# New Heterostilbene and Triazole Oximes as Potential CNS-Active and Cholinesterase-Targeted Therapeutics

**DOI:** 10.3390/biom14060679

**Published:** 2024-06-11

**Authors:** Milena Mlakić, Tena Čadež, Goran Šinko, Irena Škorić, Zrinka Kovarik

**Affiliations:** 1 Department of Organic Chemistry, Faculty of Chemical Engineering and Technology, University of Zagreb, Trg Marka Marulića 19, HR-10000 Zagreb, Croatia; mdragojev@fkit.unizg.hr; 2 Division of Toxicology, Institute for Medical Research and Occupational Health, Ksaverska cesta 2, HR-10000 Zagreb, Croatia; tcadez@imi.hr (T.Č.); gsinko@imi.hr (G.Š.); 3 Faculty of Science, University of Zagreb, Horvatovac 102a, HR-10000 Zagreb, Croatia

**Keywords:** cholinergic, HI-6, nerve agents, reactivators, Vilsmeier, Wittig reaction

## Abstract

New furan, thiophene, and triazole oximes were synthesized through several-step reaction paths to investigate their potential for the development of central nervous systems (CNS)-active and cholinesterase-targeted therapeutics in organophosphorus compound (OP) poisonings. Treating patients with acute OP poisoning is still a challenge despite the development of a large number of oxime compounds that should have the capacity to reactivate acetylcholinesterase (AChE) and butyrylcholinesterase (BChE). The activity of these two enzymes, crucial for neurotransmission, is blocked by OP, which has the consequence of disturbing normal cholinergic nerve signal transduction in the peripheral and CNS, leading to a cholinergic crisis. The oximes in use have one or two pyridinium rings and cross the brain–blood barrier poorly due to the quaternary nitrogen. Following our recent study on 2-thienostilbene oximes, in this paper, we described the synthesis of 63 heterostilbene derivatives, of which 26 oximes were tested as inhibitors and reactivators of AChE and BChE inhibited by OP nerve agents–sarin and cyclosarin. While the majority of oximes were potent inhibitors of both enzymes in the micromolar range, we identified several oximes as BChE or AChE selective inhibitors with the potential for drug development. Furthermore, the oximes were poor reactivators of AChE; four heterocyclic derivatives reactivated cyclosarin-inhibited BChE up to 70%, and *cis,trans*-**5** [2-((*Z*)-2-(5-((*E*)-(hydroxyimino)methyl)thiophen-2-yl)vinyl)benzonitrile] had a reactivation efficacy comparable to the standard oxime HI-6. *In silico* analysis and molecular docking studies, including molecular dynamics simulation, connected kinetic data to the structural features of these oximes and confirmed their productive interactions with the active site of cyclosarin-inhibited BChE. Based on inhibition and reactivation and their ADMET properties regarding lipophilicity, CNS activity, and hepatotoxicity, these compounds could be considered for further development of CNS-active reactivators in OP poisoning as well as cholinesterase-targeted therapeutics in neurodegenerative diseases such as Alzheimer’s and Parkinson’s.

## 1. Introduction

The current therapy for acute exposure to organophosphates (OPs) consists of a combination of active substances that includes an anticholinergic, a cholinesterase reactivator, and an anticonvulsant [1,2]. Oximes, reactivators of the enzyme acetylcholinesterase (AChE), possess a C=N-OH group that, upon a nucleophilic attack on the electrophilic phosphorus atom, breaks the bond between phosphorus and the catalytic serine of AChE, resulting in a free enzyme and a phosphorylated oxime. It should be emphasized that although oximes are of great importance in therapy for poisoning with OP compounds, the oximes in use today have a limited effect, which is defined by their structure and the structure of the OP-cholinesterase conjugate. Reference or standard oximes are usually specific to individual OP compounds and are not broad-spectrum [3,4,5,6,7]. In addition to the lack of universal access, another problem with reference oximes is their pharmacological properties, which are defined by their rapid dissociation in the body into ionized forms [8,9,10]. Hydrophilic oximes with a charged nitrogen atom hardly pass through the blood–brain barrier (BBB) to central synapses. The catalytic serine of BChE undergoes the same phosphorylation reactions as AChE in the presence of OP compounds. However, the different kinetic properties of cholinesterases (ChEs), due to the different structures of the active sites, affect the ability and speed of ChE to be inhibited by certain OP compounds. Previous research into therapy for poisoning with OP compounds was based on finding effective reactivators of inhibited AChE, and oxime structures were designed to achieve the best possible interaction with the phosphorylated active site of AChE, i.e., its reactivation. Consequently, most oximes developed to restore the activity of OP-AChE conjugates have been shown to be nearly ineffective in restoring OP-BChE activity.

To find new oximes that would retain excellent biochemical properties, great attention was given to the trihydroxy stilbene resveratrol and, at the same time, to heterocyclic compounds in general. *Trans*-resveratrol is known for a very wide range of biological activities [11], among which its participation in the mechanisms of the pathology of Alzheimer’s disease (AD) should be emphasized in this context [12,13]. On the other hand, most therapeutics possess at least one heterocyclic ring in their structure [14,15,16]. Applying rational design principles, we strategically incorporated the furan, thiophene, or triazole ring into the resveratrol scaffold and explored different combinations of substituents and their positions to enhance their potential for development of CNS-active and ChE-targeted therapeutics. The existence of a double bond on the (hetero)stilbene subunit opens up the possibility of different configurational isomers, which, together with a diverse configuration of the oxime group, result in different properties.

In our previous research [17], uncharged thienostilbene oximes were synthesized and characterized as reactivators of AChE and BChE inhibited by OP nerve agents. Four *trans*-derivatives reactivated cyclosarin-inhibited BChE up to 70% within two hours of reactivation. Docking studies confirmed their constructive interactions with the active site of cyclosarin-inhibited BChE, while *in silico*, evaluated ADME properties and pointed to a new class of oximes with the potential for further development of CNS-active therapeutics in OP poisoning. Building on those promising results, in this study, a number of new uncharged oxime derivatives were synthesized by a series of reactions in order to examine more deeply the influence of the heterocyclic nucleus, the position and type of substituents on the nucleus, and the position and configuration of the oxime group on their ChE reactivation potential.

**Figure 1:**
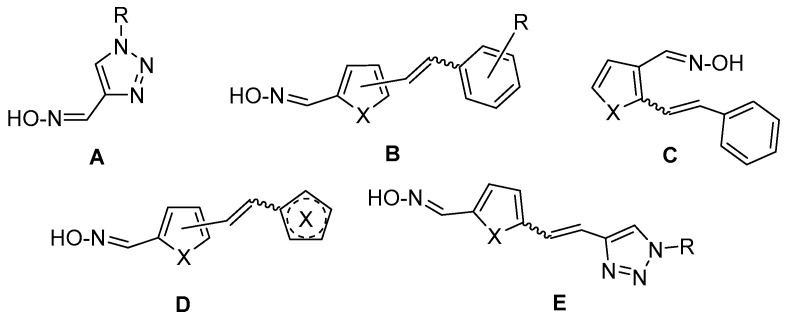
The general structures of the new oxime derivatives: triazole oximes (**A**), heterostilbene oximes with the oxime group directly attached to the furan or thiophene ring (**B**,**C**), diheterostilbene oximes (**D**) and diheterostilbene oximes with one triazole ring (**E**).

The general skeleton of the new compounds is shown in Figure 1 (structures A–E). Further on, we evaluated their physical–chemical properties important for BBB penetration *in silico* and cytotoxicity *in vitro*. Oximes were tested as inhibitors of human AChE and BChE and as reactivators of their activities upon inhibition with the OP nerve agents sarin and cyclosarin. In order to rationalize the positive interaction between the tested compounds and ChEs, we performed a molecular docking of the reversible enzyme-ligand complex and modeling of the near-attack conformation for the most potent reactivators of BChE inhibited by cyclosarin.

## 2. Results and Discussion

### 2.1. Synthesis of New Uncharged Oximes * **1**–**21** *

**Figure 2:**
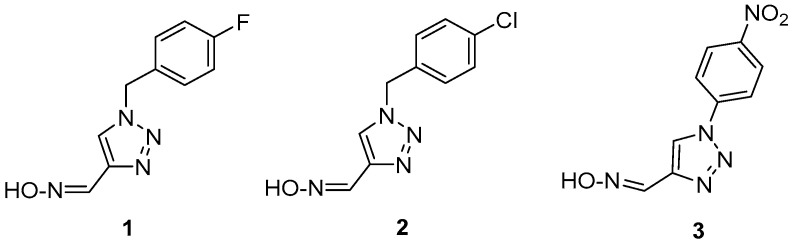
New triazole oximes **1**–**3** are the only non-stilbene type oximes with the oxime group directly attached to the triazole ring.

**Figure 3:**
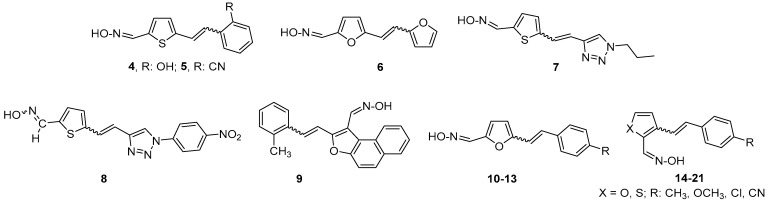
New oxime derivatives **4**–**21** with a heterostilbene skeleton.

Following the example of previously synthesized uncharged thienostilbene oximes as potential ChE reactivators [17], a large number of new oximes **1**–**21** were synthesized starting from the prepared phosphonium salts and corresponding aldehydes. The new oximes **1**–**3** (Figure 2) have their oxime group directly attached to the triazole ring and without the presence of the central double bond in the skeleton, while the other derivatives belong to heterostilbenes with an oxime group on the thiophene (**4**, **5**, **7**, **8**, **18**–**21**) or furan (**6**, **9**–**17**) ring (Figure 3).

**Scheme 1:**
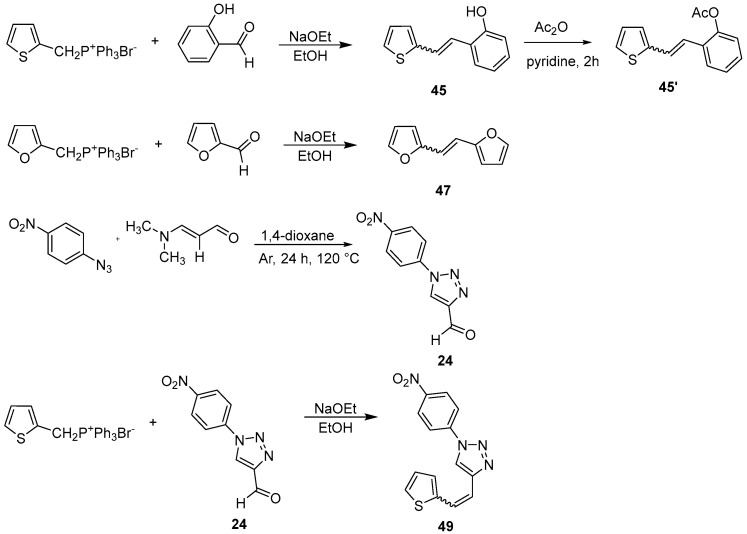
First synthetic steps towards uncharged oximes **4**–**21**.

Triazole oximes **1**–**3** were obtained (in 8–50% of isolated yields) from the triazole nitro aldehyde **24** (Scheme 1) either by direct conversion into an oxime (compound **3**) or by conversion of nitro aldehyde in a reaction with amine into new substituted triazole aldehydes **22** and **23**, and then by their conversion into an oxime (compounds **1** and **2**). This synthesis involves the corresponding starting amines in reaction with the triazole nitro aldehyde **24** [18] to produce new triazole aldehydes **22**, **23**, and **25** (see Materials and Methods) as starting substrates for the Wittig reaction used in many syntheses, especially of furan derivatives for many years [19].

**Scheme 2:**
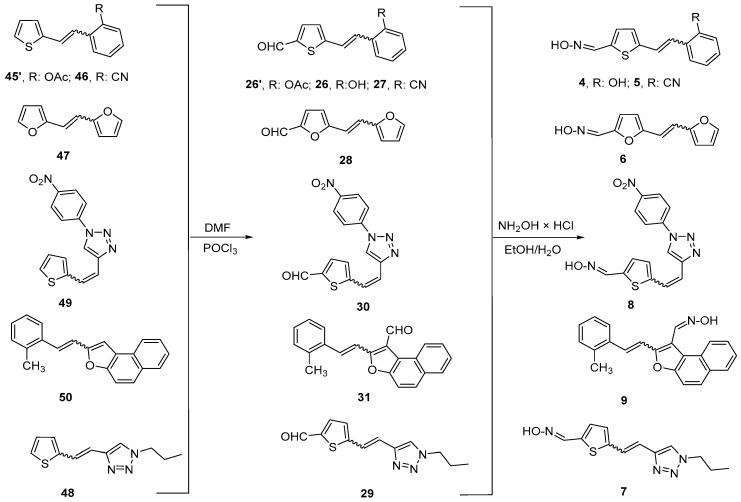
Vilsmeier formylation of new heterostilbenes and their conversion to uncharged oximes **4**–**9**.

**Scheme 3:**
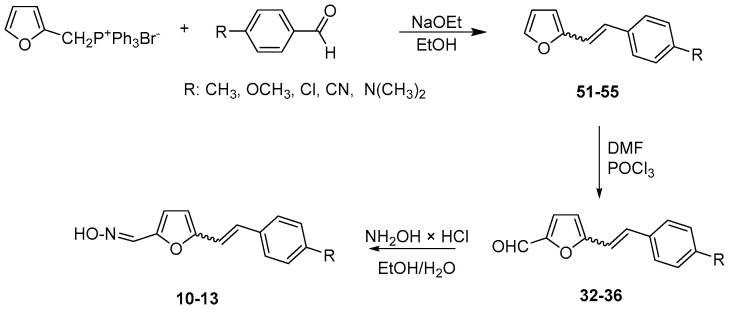
Synthetic pathway of new uncharged oximes **10**–**13** (R = CH_3_, OCH_3_, Cl, CN, N(CH_3_)_2_).

**Scheme 4:**
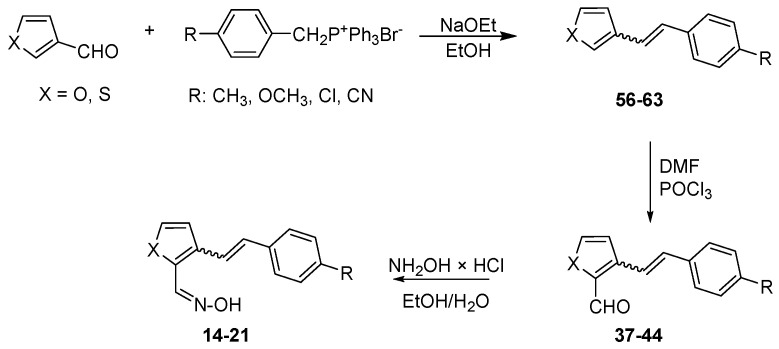
Synthetic route of new uncharged oximes **14**–**21** (R = CH_3_, OCH_3_, Cl, CN; X = O, S).

Scheme 1, Scheme 2, Scheme 3 and Scheme 4 show the synthetic route to the new uncharged oximes **4**–**21**. The preparation of the desired heterostilbene oximes **4**–**21** was achieved according to the three-step reaction path initiated by forming a double bond using the Wittig reaction to yield the starting compounds **45**–**63** as mixtures of *cis*- and *trans*-isomers (11–97%) except in the case of **45**, **45′**, **47**, **50** and **55** where only a *trans*-isomer was formed, or for the heterostilbene **49** when only a *cis*-isomer was obtained. The Wittig reaction was performed using commercially available carbaldehydes and the selected phosphonium salts prepared in our laboratory. In the second step, the obtained heterostilbenes **45**–**63** represented the basic building blocks subjected to the Vilsmeier formylation reaction, providing the corresponding aldehydes **25**–**44** (7–98%) as mixtures of isomers. In this reaction, POCl_3_ and DMF initially formed a chloriminium ion, a Vilsmeier reagent, which then reacted with the heterostilbene molecule, and an iminium ion was formed and hydrolyzed to the final aldehydes **25**–**44**. The *trans*-isomers of **45**–**63** reacted more successfully in the formylation carried out for the partially unreacted *cis*-isomers, and thus, the proportion of *trans*-isomers of aldehydes **25**–**44** was usually higher in comparison to those for the *cis*-isomers in the reaction mixture after formylation. For aldehyde *trans*-**30**, the configuration inversion occurred in the formylation reaction starting from *cis*-**49**. 

In the third step, formyl derivatives were transformed to oximes **4**–**21** as the targeted structures in a very wide range of isolated yields (2–98%) and with different proportions of individual configurational isomers. Mixtures of the geometrical isomers of oximes **4**–**21** were separated to obtain pure compounds by repeated column chromatography. The reaction of converting the synthesized aldehydes **25**–**44** into the corresponding oximes **4**–**21** involved the use of NH_2_OH × HCl and a mixture of ethanol and water as solvents and was based on the reaction described in the literature on simpler heteroaromatic systems [20]. According to ^1^H NMR spectroscopy, the nature and position of the substituent and the oxime group, as well as the nature of the heterocyclic ring, directed the ratio of configuration both on the double C=C bond and the configuration of the C=N bond of heterostilbene oximes **4**–**21** (Figure 3). The configuration of the double bonds is based exclusively on the proton chemical shift. 

After successive column chromatography, the *trans,anti*-, *trans,syn*-, *cis,anti*- and *cis,syn*-isomers of individual oxime derivatives **4**–**21** isolated at a sufficient quantity were subjected to further examination as potential ChE inhibitors and reactivators. All of the isolated oximes **1**–**21** were successfully spectroscopically characterized (See Section 3 and Supplementary Materials). Pure isolated isomers of a single oxime or a mixture of isomers were tested if there were too few pure isomers of a single oxime separated (as in the case of oximes **1**–**3**, **7**, **8**, and **13**). For some of the isolated configurational isomers, there was enough mass only for spectroscopic characterization. In cases when it was not possible to separate all of the isomers, data for individual isomers (if they were formed and clearly seen in the ^1^H NMR spectra) were listed from the spectra of oxime mixtures.

### 2.2. Prediction of ADME Properties

Chemical absorption, distribution, metabolism, and excretion (ADME), including toxicity, play key roles in drug discovery and development. A high-quality drug candidate should not only have sufficient efficacy against the therapeutic target but also show appropriate ADME properties at a therapeutic dose. For potential AChE- and BChE-targeted therapeutics, it is important to have good absorption and high potential to cross the BBB to achieve its biological activity on AChE and BChE in the brain [21,22].

**Figure 4:**
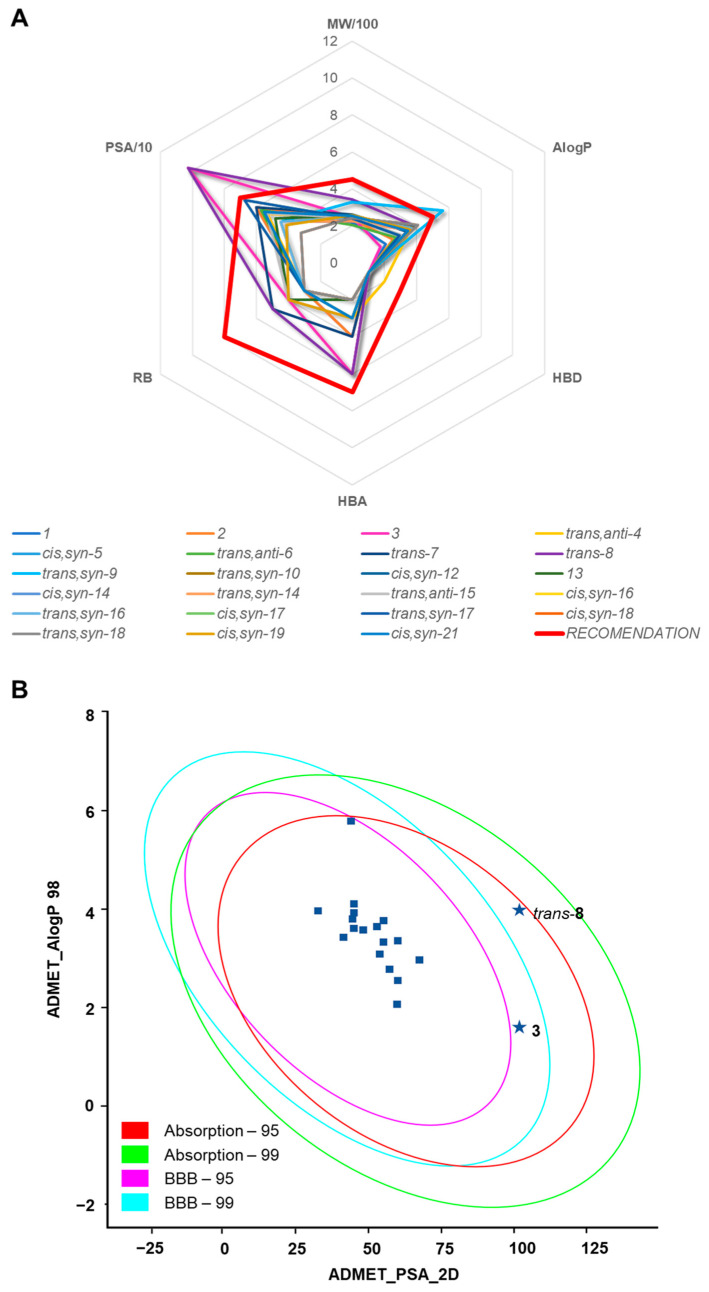
(**A**) Physico-chemical properties—molecular weight (MW), number of hydrogen bond donors and acceptors (HBDs and HBAs), number of rotating bonds (RBs), lipophilicity (AlogP), and polar surface area (PSA), of uncharged oximes in relation to the recommended values (red line). (**B**) Correlation plot for prediction of BBB penetration. The recommended values of CNS-active drugs are given by Pajouhesh and Lenz [23].

Therefore, we predicted the lipophilicity (AlogP) and polar surface area (PSA) for the 26 prepared oxime compounds based on molecular weight (MW), the number of hydrogen bond donors and acceptors (HBDs and HBAs), and number of rotating bonds (RBs). We then estimated their BBB-penetration ability and compared it with recommended values for CNS-active drugs (Figure 4) [23]. An examination of the radar plot, and AlogP and 2D PSA correlation plot reveals a high potential of the new uncharged oximes to cross the BBB. An exception is the ADME properties of compounds **3** and *trans*-**8**, which seem to have larger PSAs than is recommended for a CNS-active drug. However, as Lipinski’s rule allows one exception [24], keeping in mind their high absorption, these compounds can potentially be considered orally CNS-active compounds. 

The high lipophilicity implied low solubility in preferential phosphate buffers for ChEs, so additional attention was given to the impact of the solvent on ChE activity [25]. In our recent study, we reported natural deep eutectic solvent (NADES) as an alternative solvent for the preparation of CNS-active oximes [26] capable of not only increasing the solubility but also improving oxime properties in interactions with AChE. Indeed, whether a molecule will provide a satisfying therapeutic effect depends not only on how it interacts with the molecular target (pharmacodynamic aspect) but also on how it is processed by the body (pharmacokinetic aspect). Therefore, the search for molecules with better pharmacodynamics and pharmacokinetic characteristics is still an ongoing pursuit with a focus on CNS-active ChE-targeted therapeutics [27]. It is worth mentioning that CNS-active AChE reactivators, such as zwitterionic oximes [28] and a 3-hydroxy-2-pyridine reactivator [22,29], showed an improvement in BBB penetration compared with standard oximes. However, the challenge seems to be quick elimination, which could be solved with multiple oral applications. Therefore, it is important to note that *in silico* prediction tests showed that our new compounds possess high bioavailability and are suitable for multiple oral administration to increase and maintain a therapeutic dose of compounds, as well as to increase their residency in the brain.

### 2.3. Cytotoxicity of New Uncharged Oximes

To estimate the impact of the prepared compounds on cellular homeostasis, we evaluated 24 h cytotoxicity by measuring the succinate dehydrogenase mitochondrial activity in an exposed liver cell line (HepG2), one of the standard cell lines for metabolism and drug-safety evaluations (Figure S299). It is worth highlighting that these results also show the effects of the degradation products of compounds expected to form in a 24-h assay. The cytotoxic effect of the new uncharged oximes in terms of IC_50_ is summarized in Table 1. Out of the 26 tested compounds, the IC_50_ values of 17 oximes were estimated to be higher than 400 μM, categorizing those oximes as likely non-hepatotoxic compounds. Although nine oximes exhibited toxic effects within the tested concentration range comparable to Triton, used as a positive control, which led to 100% cytotoxicity for HepG2 cells, only compounds *cis,syn*-**16**, *trans,syn*-**10**, *cis,syn*-**21**, and *cis,syn*-**17** had IC_50_ values equal to or lower than 100 µM, demonstrating relatively high hepatotoxicity.

It seems there is a prevalence of *cis*-derivatives over *trans*-derivatives since nine out of ten *cis*-oximes exhibited hepatoxicity. In accordance with recent studies, the mechanism of toxicity depended on the structure of oximes, and variations in the structure significantly altered the toxic effect on cells [30,31,32]. It is important to mention that these studies on various cell types representing the liver, kidney, neurons, and muscles revealed that cytotoxicity was not dependent on cell type and was induced by different mechanisms/types of regulated cell death activation by caspase-dependent apoptosis or cell death [30,31,32]. More precisely, pyridinium oximes (IC_50_ 10–300 µM) induced apoptosis by an intrinsic mitochondria-dependent pathway through the activation of specific caspase 9, while imidazolium oximes (IC_50_ 120–320 µM) triggered necrotic events characterized by elevated levels of reactive oxygen species, loss of mitochondrial potential and uncontrolled intracellular lactate dehydrogenase leakage accompanied by cell burst [31]. Biological response and cell death signaling pathways modulated by uncharged 3-hydroxy-2-pyridine aldoximes with tetrahydroisoquinoline moieties (IC_50_ 8–120 µM) showed time-dependent effects and stimulated mitochondria-mediated activation of the intrinsic apoptosis pathway through ERK1/2 and p38-MAPK signaling and subsequent activation of initiator caspase 9 and executive caspase 3 accompanied with DNA damage, as observed already after 4 h of exposure. Mitochondria and fatty acid metabolism were also likely targets of 3-hydroxy-2-pyridine aldoximes with tetrahydroisoquinoline moiety due to increased phosphorylation of acetyl-CoA carboxylase [32].

### 2.4. Reversible Inhibition of Cholinesterase by the New Uncharged Oximes

We can generally emphasize that new heterocyclic oximes bind to both AChE and BChE in a reversible inhibition manner within the micromolar range. The inhibition potency in terms of inhibition dissociation constants (*K*_i_) is given in Table 2. Due to poor solubility and poor enzyme binding affinity, *K*_i_ constants for AChE could not be determined for eight compounds, while only three of them *trans*-**13**, *trans,anti*-**15**, and *cis,syn*-**19′**, were poor inhibitors of both human AChE and BChE. BChE showed the highest affinity (1/*K*_i_) for 2,5-substituted furan oximes *trans,anti*-**4** and *cis,syn*-**5**, and triazole oxime **2**. Interestingly, in the case of AChE, heterostilbenes *trans,syn*-**18**, *trans,syn*-**17**, and *cis,syn*-**12**, with the *syn* configuration on the oxime group and substituent in the *para*-position, were the most potent inhibitors, although about 10-fold lower than the most potent BChE inhibitor, *trans,anti*-**4**. 

Moreover, the most potent AChE inhibitors were poor inhibitors of BChE and vice versa (with the exception of *cis,syn*-**5** and *trans,syn*-**10**, which were potent inhibitors of both enzymes). Therefore, we can discuss these results in terms of inhibition selectivity as the *K*_i_ ratio between the two enzymes. In addition, BChE inhibitor *trans,anti*-**4**, which had the lowest *K*_i_, was identified as a potent and selective inhibitor of BChE. These results should encourage further tests of these compounds as potential therapeutics for neurological disorders, as both non-selective or BChE-selective inhibitors are highlighted as potential drugs in AD and other neuromuscular disorders [33,34,35,36]. More precisely, both AChE and BChE are targets in drug development for symptomatic treatment of Alzheimer’s disease (AD), e.g., galantamine and donepezil, which are currently in use, are AChE inhibitors [34]. However, since BChE activity in certain brain regions increased with AD progression, BChE inhibitors became additional targets in the development of drugs for AD and related dementias [33].

While in our previous paper, eight 2-thienostilbene oxime derivatives were reported as reversible inhibitors of human AChE and BChE with *K*_i_ constants also in the micromolar range, seven of the oximes were more potent inhibitors of AChE (4–80 µM) than of BChE (44–573 µM) [17]. In addition, inhibition of AChE was uncompetitive, while all of the compounds exhibited competitive binding for BChE. Along with somewhat mixed findings, this is quite opposite for the studied heterocyclic oximes—out of the twenty-six compounds, nine and eleven oximes were competitive, while six and five were uncompetitive inhibitors in the case of AChE and BChE, respectively. All BChE potent inhibitors showed an uncompetitive character of inhibition, meaning that potency of inhibition did not depend on the concentration of the substrate acetylthiocholine (Figure S300). It is also important to highlight that these uncharged heterostilbenes, which were identified as the most potent inhibitors of AChE and BChE, possessed binding affinity within the range of charged oximes [37,38,39]. Therefore, our results confirmed those from a previous study on (thiophen-2-yl)-aldoximes, which reported that the hetero atom, due to its potential for polarization, helps to stabilize the negative charge of its anionic form, similarly to the pyridinium ring of the standard oxime 2-PAM [20]. The affinity of native ChE for these compounds is still not as high as for the cholinergic agonist tacrine [21].

It is also very interesting to recapitulate our previous results on analogs of the well-known bioactive molecule resveratrol that underwent evaluation for antioxidant activity along with their potential to inhibit non-human AChE and BChE [40]. The biological tests showed that the derivative with *trans*-configuration, similar to *trans,anti*-**4**, exhibited significant antioxidative and BChE inhibitory potential, as evidenced by lower IC_50_ values compared with the established standards, *trans*-resveratrol, and galantamine, respectively.

### 2.5. Molecular Modeling of Heterostilbene Oximes−ChE Complex

**Figure 5:**
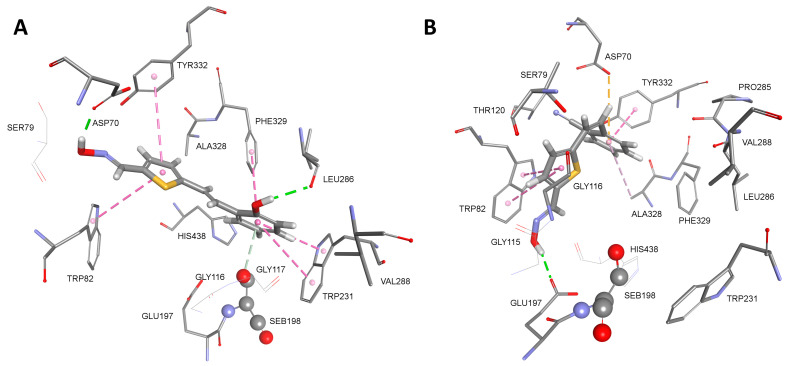
Conformation of the complex between native human BChE and *trans,anti*-**4** (**A**) and *cis,syn*-**5** (**B**). Interactions with amino acid residues are represented as dashed lines: hydrophobic (purple), hydrogen bonds (green), and electrostatic (orange). Crystal structure of human BChE was used (PDB code 2PM8) [44].

To rationalize interactions between oxime molecules and amino acids lining the active site gorge, we performed docking to visualize possible binding interactions (Figure 5 and Figure S301). Several modes of binding in the ChE active site were observed. Due to the hydrophobic nature of the active site, the main interactions for the stabilization came from aromatic residues Phe, Tyr, or Trp. Moreover, the active site of AChE preferred elongated ligands, which can simultaneously bind to the choline-binding site and peripheral site, resulting in higher inhibition potency [41]. In this study, compounds with a *trans*-configuration of double bond *trans,syn*-**18** and *trans,syn*-**17** were the most potent inhibitors of AChE (Table 2, Figure S301C,D). Molecular modeling showed stabilization of the substituted phenyl group via multiple hydrophobic interactions (Phe295, Phe297, Tyr337, Phe338, and His447), including the acyl binding site, while the oxime group attached to the heteroaromatic ring was stabilized via H-bonds from the peripheral site Asp74 and Tyr341 or Thr83, Asn87, and Tyr341.

Compounds with a *cis*-configuration of the double bond *cis,syn*-**5** and *cis,syn*-**12** in human AChE showed similar stabilization of the oxime group via multiple H-bonds from Asp74 and Tyr341 or Ser125 (Figure S301A,B). Due to the bent shape of these molecules, the phenyl group is stabilized in the choline-binding site away from the acyl-binding site. Multiple hydrophobic interactions were included in the stabilization of the cyano or chlorine-substituted phenyl group, primarily from Trp86 and additionally Tyr337 and His447.

In human BChE, both thiostilbenes *trans,anti*-**4** and *cis,syn*-**5** seemed to bind simultaneously to the choline-binding site (Trp82) and the peripheral binding site (Asp70, Trp332) (Figure 5). Strong stabilization of *trans,anti*-**4** occurs due to the formation of multiple hydrophobic interactions with Trp82, Trp231, and Phe329. Additional stabilization came from the H-bond between Asp70 and the oxime group, as well as Leu286 and the hydroxyl group. Catalytic Ser198 also stabilizes *trans,anti*-**4** via electron-π interaction from the oxygen (Oγ) of Ser198. The thiostilbene ring of *cis,syn*-**5** forms a π-π sandwich with the indole ring of Trp82 with additional stabilization in the form of an H-bond between Glu197 and the oxime group. It is worth mentioning that the formation of a π-π sandwich between thiostilbene and the indole ring of Trp82 is analogous to the crystal structure of the triazole compound III bound in the human BChE active site (PDB code 6T9P) where the imidazole ring forms a π-π sandwich with Trp82 [42]. The cyano group at the phenyl ring is stabilized in the BChE peripheral binding site, creating hydrophobic interactions with Tyr332 and Ala328 and an additional electrostatic π-anion interaction from Asp70. A similar binding mode was determined for triazole compound **2** that binds simultaneously to the choline-binding site and acyl-binding site of BChE (Figure S301E). The triazole ring bearing an oxime group is stabilized by Trp82 via hydrophobic interactions and with an additional H-bond from Gly115, a member of the oxyanion hole. Chlorine at the phenyl ring is stabilized via multiple hydrophobic interactions formed with Val288, Trp231, and Phe332, including electrostatic electron-π interactions from the oxygen (Oγ) of Ser198. Our results on docking are in agreement with the crystal structures of ligands, showing that potent inhibitors of BChE simultaneously bind to the choline-binding site and acyl pocket or peripheral binding site [43].

### 2.6. Oxime-Assisted Reactivation of AChE and BChE

**Figure 6:**
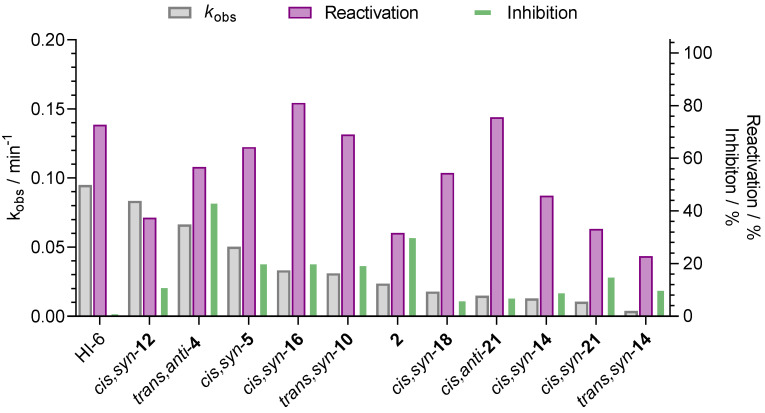
Reactivation of cyclosarin-inhibited BChE by 100 µM oximes in terms of the reactivation rate constants, *k*_obs_ (grey columns), maximal reactivation in 240 min (purple columns), and inhibition of BChE by oximes (green columns). The presented values are means of at least two experiments measured at 25 °C.

The new heterostilbene and triazole were initially tested for reactivation of sarin- and cyclosarin-inhibited AChE and BChE at the given oxime concentration of 0.1 mM. Reactivation of AChE did not exceed 20% within 5 h in both sarin and cyclosarin. While for the reactivation of sarin-inhibited BChE, no oxime had the capability to return its activity, cyclosarin-inhibited BChE was promptly reactivated, higher than 20%, with 11 uncharged oximes, and the results were sorted in terms of the observed first-order reactivation rate (*k*_obs_) (Figure 6). Oxime *cis,syn*-**16** exhibited the highest reactivation capabilities up to 80%, while *cis,anti*-**21** was also able to achieve a high reactivation percentage of 75%, but with a slow reactivation rate. Evidently, the standard oxime HI-6 demonstrated the highest observed first-order reactivation rate but not significantly higher than *cis,syn*-**12**, which reactivated with the highest *k*_obs_ of all uncharged oximes, but the maximal reactivation was low (Figure 6). Along with *cis,syn*-**16**, oximes *trans,anti*-**4**, *cis,syn*-**5**, and *trans,syn*-**10** reactivated BChE activity above 50% with moderately high reactivation rates about 4-fold higher than *k*_obs_ determined for the 2-thiostilbenes previously [17]. 

Based on initial screening, out of the 26 uncharged oximes, four oximes—two 2,5-thienstilbenes *trans,anti*-**4** and *cis,syn*-**5**, 2,3-furostilbene *cis,syn*-**16**, and 2,5-furanstilbene *trans,syn*-**10**—were singled out for further detailed kinetics reactivation of BChE inhibited by cyclosarin. The reactivation kinetic parameters were determined in a limiting concentration range (0.01–0.2 mM) due to poor solubility and compared with standard reactivator HI-6 (Table 3). The reactivation of cyclosarin-inhibited BChE with all of the selected oximes reached maximal reactivation in up to 140 min, where the highest reactivation of 80% was achieved in the presence of chlorinated 2,5-furanstilbene oxime, *cis,syn*-**16**. The thienstilbene compound containing a cyano group, *cis,syn*-**5**, exhibited the highest reactivation efficiency primarily as a result of its relatively high binding affinity, even surpassing that of standard HI-6 [29]. Indeed, cyclosarin-conjugated BChE probably had a low binding affinity for the other tested oximes, indicated by a linear relationship between *k*_obs_ and oxime concentration, enabling us to determine the overall reactivation constant only.

Nevertheless, our reactivation study confirms that although both AChE and BChE share the same mechanism of reaction, the structure of OP moiety conjugated at the catalytic serine and difference in residues in the active site direct the specificities of AChE and BChE reactivation and therefore, no single oxime reported in the literature is equally efficient against a variety of OPs or equally potent for both AChE and BChE [29,45]. It is worth mentioning here that, with the design of these uncharged oximes, we joined an endeavor undertaken by several research teams to develop a new generation of CNS-active reactivators [9,46,47,48,49,50,51,52,53,54,55,56,57].

### 2.7. Modelling of a Complex between an Oxime and Cyclosarin-Inhibited BChE

**Figure 7:**
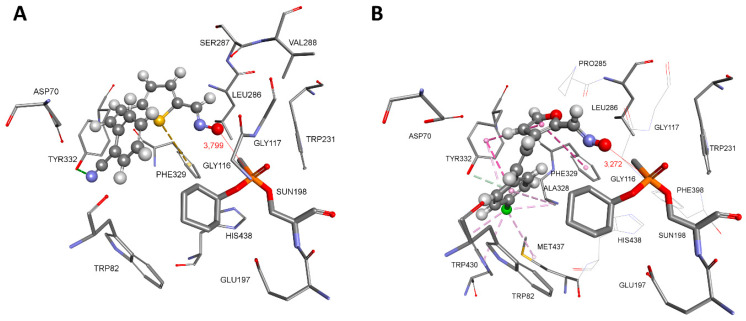
Minimized conformation of a complex in near-attack conformation between cyclosarin-inhibited BChE and *cis,syn*-**5** (**A**) and *cis,syn*-**16** (**B**). The Van der Waals distance of the oxime group from the oxygen (Oγ) of catalytic serine (Ser198) is red. Oxime interactions with active site residues are represented as dashed lines: hydrophobic (purple and yellow) and hydrogen bonds (green). The crystal structure of human BChE inhibited by tabun (PDB code 3DJY) [60] was used for the modeling of cyclosarin-inhibited BChE [58].

Since molecular docking fails in the modeling of a near-attack conformation of inhibited ChE by OPs, we performed an alternative approach using the structure of a reactivation product, a phosphorylated oxime [58]. The complex between oxime *cis,syn*-**5** or *cis,syn*-**16**, and cyclosarin-inhibited BChE representing the near-attack conformation is given in Figure 7. The following van der Waals distances were obtained from the oxime group oxygen to the phosphorus atom of the phosphoester conjugate: 3.8 Å and 3.3 Å for *cis,syn*-**5** and *cis,syn*-**16**, respectively. It is very obvious that the near-attack conformation was stabilized in a different binding pose with fewer interactions from BChE active site residues than the reversible complex representing inhibition (cf. Figure 5B and Figure S301E). Compound *cis,syn*-**5** was stabilized via electron-π interactions involving sulfur and Phe329 and an additional H-bond from Tyr332. The presence of the cyclohexyl ring in the active site blocked the interaction of *cis,syn*-**5** with Trp82 but stabilized the position of the cyano-substituted phenyl ring (cf. Figure 5B). Multiple hydrophobic interactions were formed between the *para*-chlorinated phenyl group and mainly aromatic residues of the active site (Phe329, Tyr332, and Trp430) and Ala328 and Met437. This type of binding and chlorine interactions were analogous to the crystal structure of a chlorinated tacrine derivate, a potent ChE inhibitor bound to BChE (PDB code 6I0B) [59]. Stronger stabilization of *cis,syn*-**16** may cause lower reactivation potency compared with *cis,syn*-**5** due to less freedom for the movement required for the nucleophilic attack of the oxime group.

To test the stability of near-attack conformation between cyclosarin-inhibited BChE and *cis,syn*-**5** and *cis,syn*-**16**, we performed molecular dynamics simulation (t = 20 ns) as shown in Figures S302 and S303. Analysis of dynamics trajectory for *cis,syn*-**5** showed a stable position in the active site of cyclosarin-inhibited BChE during 20 ns simulation with an average distance from oxime group oxygen (O_1_) to phosphorus atom from cyclosarin conjugate being 7.25 Å. Other monitored distances showed small fluctuations during the simulation, and the cyclohexyl ring formed a stable interaction with Trp82 from the choline-binding site. Analysis of dynamics trajectory for *cis,syn*-**16** showed some similarities to *cis,syn*-**5** trajectory, but the distance from oxime group oxygen (O_1_) to phosphorus atom from cyclosarin conjugate increased from ~7 Å to ~8 Å. Additionally, the oxime *cis,syn*-**16** distance from the C_32_ atom to the cyclohexyl ring of cyclosarin conjugate increased to 5.57 Å.

## 3. Materials and Methods

### 3.1. Chemistry

All nuclear magnetic resonance (NMR) spectroscopic data for ^1^H and ^13^C were recorded in deuterated chloroform, CDCl_3_, and deuterated methanol CD_3_OD (Sigma-Aldrich, St. Louis, MO, USA) using tetramethylsilane as standard at room temperature on Bruker Avance 300 and 600 MHz spectrometers. For the full characterization of the targeted oximes, additional techniques, 2D-CH correlation (HSQC) and 2D-HH-COSY were used. The following abbreviations were used in the NMR spectra: s (singlet), d (doublet), t (triplet), q (quartet), dd (doublet of doublets), and m (multiplet). Chemical shifts were reported in parts per million (ppm). High-resolution mass spectrometry (HRMS) analyses were carried out on a mass spectrometer (MALDI TOF/TOF analyzer) equipped with an Nd:YAG laser operating at 355 nm with a fitting rate of 200 Hz in the positive (H+) or negative (−H) ion reflector mode. All of the compounds tested for reversible inhibition and oxime-assisted reactivation of ChE were >95% pure by high-resolution mass spectrometry (HRMS) or HPLC analyses (see Supplemental Information). The results of HRMS and HPLC analyses are included in the Supporting Information. All of the used solvents (Sigma-Aldrich, St. Louis, MO, USA) for the synthesis were purified by distillation and were commercially available. Anhydrous magnesium sulfate, MgSO_4_, was used to dry the organic layers after extractions. Column chromatography was performed on columns with silica gel (0.063–0.2 nm and 60 Å, technical grade, Fluka Chemie GmbH, Buchs, Switzerland) using the appropriate solvent system. Abbreviations used in experimental procedures were ACN—acetonitrile, EtOAc—ethyl acetate, PE—petroleum ether, E—diethylether, EtOH—ethanol, DCM—dichloromethane, DMF—dimethyl formamide, POCl_3_—phosphoryl chloride, NaOH—sodium hydroxide. All solvents were removed from the solutions by rotary evaporator under reduced pressure. The initial phosphonium salts were prepared in the laboratory from the corresponding bromides, while the other starting compounds used were purchased chemicals.

Heterostilbenes **45**–**63** were obtained as mixtures of *cis*- and *trans*-isomers ((11–97%) except in the case of **45**, **45′**, **47**, **50**, and **55** where only a *trans*-isomer was formed, or for the heterostilbene **49** when only a *cis*-isomer was obtained) using the Wittig reaction. The reaction apparatus was purged with N_2_ for 15 min before adding the reactants. The reactions were carried out in three-necked flasks (100 mL) equipped with a chlorine–calcium tube and a connected N_2_ balloon. Phosphonium salts (5 mmol) were added to the 40 mL of EtOH, and the mixtures were stirred with a magnetic stirrer. A solution of sodium ethoxide (5 mmol, 1.1 eq of Na dissolved in 10 mL of absolute ethanol) was added dropwise in strictly anhydrous conditions under N_2_. The corresponding aldehydes (5 mmol) were then added to the reaction mixtures, which were allowed to stir for 24 h at room temperature. The reaction mixtures were evaporated on a vacuum evaporator and dissolved in toluene. The products were then extracted with toluene (3 × 15 mL). The organic layers were dried under anhydrous MgSO_4_. Products **45**–**63** were isolated by repeated column chromatography on silica gel using PE/E, PE/DCM, and E/EtOAc solvent systems. The first isomer to eluate was *cis*-isomer (but in some cases, it was not isolated), and the *trans*-isomer was isolated in the last fractions. The spectroscopic characterization of newly isolated heterostilbenes is given below.

Compound *trans*-**45** was converted to aldehyde by Vilsmeier formylation; however, the expected product was not produced. After that, protection of its OH group was carried out in a round flask (25 mL) using acetic anhydride (1.5 mL) at room temperature overnight in pyridine. After that, a mixture of water, toluene, and acetone (1:3:3) was added, and the reaction mixture was evaporated under reduced pressure, and a yellow solid remained in the flask (*trans*-**45′**).

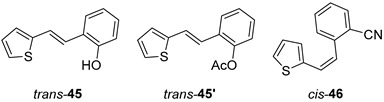

(*E*)-2-(2-(thiophen-2-yl)vinyl)phenol (*trans*-**45**) [40] 495 mg, 82% isolated yield; white powder; R*_f_* (PE/DCM (50%)) = 0.33; UV (ACN) *λ*_max_/nm (*ε*/dm^3^mol^−1^cm^−1^) 335 (27412); ^1^H NMR (CDCl_3_, 600 MHz) *δ*/ppm: 7.46 (dd, *J* = 7.7, 1.6 Hz, 1H), 7.28 (d, *J* = 16.1 Hz, 1H), 7.20–7.16 (m, 2H), 7.10 (t, *J* = 7.4 Hz, 1H), 7.07 (d, *J* = 3.7 Hz, 1H), 6.99 (dd, *J* = 5.1, 3.5 Hz, 1H), 6.93 (t, *J* = 7.5 Hz, 1H), 6.78 (dd, *J* = 8.1, 1.2 Hz, 1H), 4.97 (s, 1H); ^13^C NMR (CDCl_3_, 150 MHz) *δ*/ppm: 152.9, 143.3, 128.6, 127.6, 127.2, 126.0, 124.4, 124.3, 123.3, 122.7, 121.2, 115.9; MS (ESI) (*m*/*z*) (%, fragment): 202 (25), 105 (100).
(*E*)-2-(2-(thiophen-2-yl)vinyl)phenyl acetate (*trans*-**45′**): 286 mg, 95% isolated yield; colorless oil; R*_f_* (DCM) = 0.75; ^1^H NMR (CDCl_3_, 600 MHz) *δ*/ppm: 7.62 (dd, *J* = 7.6, 1.5 Hz, 1H), 7.28–7.20 (m, 4H), 7.08–7.06 (m, 2H), 7.00 (dd, *J* = 5.2, 3.7 Hz, 1H), 6.94 (d, *J* = 16.1 Hz, 1H), 2.37 (s, 3H); ^13^C NMR (CDCl_3_, 150 MHz) *δ*/ppm: 169.3, 148.1, 142.7, 129.6, 128.4, 127.6, 126.6, 126.3, 124.7, 124.0, 122.8, 122.8, 121.5, 20.9.
(*Z*)-2-(2-(thiophen-2-yl)vinyl)benzonitrile (*cis*-**46**): 350 mg, 50% isolated yield; colorless oil; R*_f_* (DCM) = 0.65; ^1^H NMR (CDCl_3_, 600 MHz) *δ*/ppm: 7.71 (d, *J* = 7.7 Hz, 1H), 7.57–7.53 (m, 2H), 7.42–7.39 (m, 1H), 7.12 (d, *J* = 5.8 Hz, 1H), 6.96 (d, *J* = 3.6 Hz, 1H), 6.93 (d, *J* = 12.1 Hz, 1H), 6.90 (dd, *J* = 4.9, 3.6 Hz, 1H), 6.62 (d, *J* = 12.1 Hz, 1H); ^13^C NMR (CDCl_3_, 150 MHz) *δ*/ppm: 182.9, 147.6, 143.5, 140.0, 135.9, 133.5, 133.1, 130.3, 129.7, 128.9, 128.8, 126.4, 117.4, 112.3.

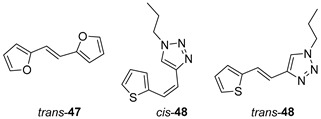

(*E*)-1,2-di(furan-2-yl)ethene (*trans*-**47**): 93 mg, 20% isolated yield; colorless powder; R*_f_* (PE) = 0.58; ^1^H NMR (CDCl_3_, 300 MHz) *δ*/ppm: 7.42 (d, *J* = 17.7 Hz, 1H), 7.34 (d, *J* = 17.7 Hz, 1H), 6.81 (t, *J* = 19.4 Hz, 2H), 6.42–6.40 (m, 2H), 6.32 (d, *J* = 3.2 Hz, 2H).
(*Z*)-1-propyl-4-(2-(thiophen-2-yl)vinyl)-1*H*-1,2,3-triazole (*cis*-**48**) [61]: 110 mg, 48% isolated, colorless oil; R*_f_* (PE/E (40%)) = 0.34; UV (ethanol, 96%) *λ*_max_/nm (*ε*/dm^3^mol^−1^cm^−1^) 296 (16418), 310 (14455, sh); ^1^H NMR (CDCl_3_, 300 MHz) *δ*/ppm: 7.53 (s, 1H), 7.25 (d, *J* = 4.7 Hz, 1H), 7.22 (d, *J* = 3.7 Hz, 1H), 6.99 (dd, *J* = 4.9, 3.4 Hz, 1H), 6.70 (d, *J* = 12.1 Hz, 1H), 6.54 (d, *J* = 12.1 Hz, 1H), 4.27 (t, *J* = 14.2 Hz, 3H), 1.93–1.87 (m, 2H), 0.94 (t, *J* = 14.6 Hz, 3H); ^13^C NMR (CDCl_3_, 75 MHz) *δ*/ppm: 143.8, 139.5, 128.5, 127.1, 126.0, 123.8, 122.1, 118.3, 51.9, 23.74, 11.1.
(*E*)-1-propyl-4-(2-(thiophen-2-yl)vinyl)-1*H*-1,2,3-triazole (*trans*-**48**) [62]: 20 mg, 10% isolated; white powder; R*_f_* (PE/E (40%)) = 0.29; UV (ethanol, 96%) *λ*_max_/nm (*ε*/dm^3^mol^−1^cm^−1^) 314 (30210); ^1^H NMR (CDCl_3_, 600 MHz) *δ*/ppm: 7.51 (s, 1H), 7,44 (d, *J* = 16.2 Hz, 1H), 7.20 (d, *J* = 8.2 Hz, 1H), 7.07 (d, *J* = 3.5 Hz, 1H), 7.00 (dd, *J* = 5.1, 3.6 Hz, 1H), 6.89 (d, *J* = 16.2 Hz, 1H), 4.33 (t, *J* = 7.2 Hz, 2H), 1.95 (m, *J* = 7.3 Hz, 2H), 0.98 (t, *J* = 7.4 Hz, 3H); ^13^C NMR (CDCl_3_, 150 MHz) *δ*/ppm: 145.2, 141.7, 127.1, 125.9, 124.2, 123.1, 119.6, 115.7, 51.4, 23.2, 10.6.

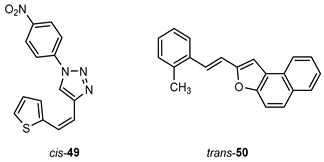

(*Z*)-1-(4-nitrophenyl)-4-(2-(thiophen-2-yl)vinyl)-1*H*-1,2,3-triazole (*cis*-**49**): 85 mg, 42% isolated yield; yellow oil; *R_f_* (E) = 0.85; ^1^H NMR (CDCl_3_, 600 MHz) *δ*/ppm: 8.41 (d, *J* = 8.7 Hz, 2H), 8.10 (s, 1H), 7.94 (d, *J* = 8.9 Hz, 2H), 7.32 (d, *J* = 5.5 Hz, 1H), 7.28 (d, *J* = 3.3 Hz, 1H), 7.06 (t, *J* = 4.1 Hz, 1H), 6.86 (d, *J* = 12.2 Hz, 1H), 6.59 (d, *J* = 12.2 Hz, 1H); ^13^C NMR (CDCl_3_, 75 MHz) *δ*/ppm: 147.2, 145.3, 141.5, 138.9, 129.2, 127.3, 126.6, 125.7, 125.6, 120.4, 119.8, 116.7.
(*E*)-2-(2-methylstyryl)naphtho[2,1-*b*]furan (*trans*-**50**): 210 mg, 95% isolated yield; yellow powder; R*_f_* (PE/E (50%)) = 0.95; ^1^H NMR (CDCl_3_, 300 MHz) *δ*/ppm: 8.12 (d, *J* = 8.5 Hz, 1H), 7.93 (d, *J* = 8.4 Hz, 1H), 7.74–7.55 (m, 5H), 7.48 (t, *J* = 7.7 Hz, 1H), 7.25–7.18 (m, 4H), 7.01 (d, *J* = 16.2 Hz, 1H), 2.50 (s, 3H); ^13^C NMR (CDCl_3_, 75 MHz) *δ*/ppm: 154.9, 152.4, 136.2, 135.6, 130.6, 130.4, 128.8, 127.9, 127.5, 127.1, 126.2, 126.2, 125.6, 125.5, 125.0, 124.6, 124.5, 123.5, 117.5, 112.1, 104.2, 19.9.

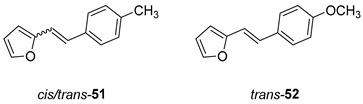

(*E*)-2-(4-methoxystyryl)furan (*trans*-**52**): 750 mg, 36% isolated yield; white powder; *R_f_* (PE/E (2%)) = 0.31; ^1^H NMR (CDCl_3_, 600 MHz) *δ*/ppm: 7.39 (d, *J* = 9.7 Hz, 2H), 6.99 (d, *J* = 15.9 Hz, 1H), 6.87 (d, *J* = 8.9 Hz, 2H), 6.75 (d, *J* = 15.9 Hz, 1H), 6.41–6.39 (m, 1H), 6.29 (d, *J* = 2.4 Hz, 1H), 3.81 (s, 3H); ^13^C NMR (CDCl_3_, 75 MHz) *δ*/ppm: 159.4, 153.7, 141.7, 129.8, 127.5, 126.7, 114.6, 114.5, 111.5, 107.7, 55.3.

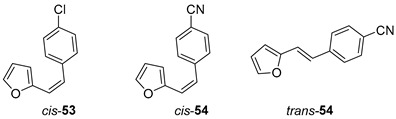

(*Z*)-2-(4-chlorostyryl)furan (*cis*-**53**): 240 mg, 23% isolated yield; colorless oil; *R_f_* (PE/E (2%)) = 0.95; ^1^H NMR (CDCl_3_, 600 MHz) *δ*/ppm: 7.38 (d, *J* = 8.5 Hz, 2H), 7.28 (d, *J* = 1.8 Hz, 1H), 7.29 (d, *J* = 8.5 Hz, 2H), 6.40 (d, *J* = 12.5 Hz, 1H), 6.35 (d, *J* = 12.5 Hz, 1H), 6.35–6.32 (m, 1H), 6.26 (d, *J* = 3.2 Hz, 1H).
(*Z*)-4-(2-(furan-2-yl)vinyl)benzonitrile (*cis*-**54**): 170 mg, 9% isolated yield; colorless oil; *R_f_* (PE/E (30%)) = 0.19; ^1^H NMR (CDCl_3_, 600 MHz) *δ*/ppm: 7.55 (d, *J* = 8.7 Hz, 2H), 7.50 (d, *J* = 8.6 Hz, 2H), 7.30 (d, *J* = 1.9 Hz, 1H), 6.44 (d, *J* = 12.4 Hz, 1H), 6.40 (d, *J* = 12.5 Hz, 1H), 6.37–6.35 (m, 1H), 6.31 (d, *J* = 3.7 Hz, 1H).
(*E*)-4-(2-(furan-2-yl)vinyl)benzonitrile (*trans*-**54**): 150 mg, 8% isolated yield; white powder; *R_f_* (PE/E (30%)) = 0.22; ^1^H NMR (CDCl_3_, 600 MHz) *δ*/ppm: 7.54 (d, *J* = 8.7 Hz, 2H), 7.51 (d, *J* = 8.7 Hz, 2H), 7.44 (d, *J* = 1.9 Hz, 1H), 7.01 (d, *J* = 16.3 Hz, 1H), 6.97 (d, *J* = 16.3 Hz, 1H), 6.45 (d, *J* = 3.3 Hz, 1H), 6.36 (dd, *J* = 1.9, 3.3 Hz, 1H).

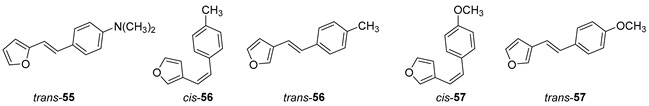

(*E*)-4-(2-(furan-2-yl)vinyl)-*N*,*N*-dimethylaniline (*trans*-**55**): 79 mg, 11% isolated yield; yellow powder; *R_f_* (PE/E (5%)) = 0.36; ^1^H NMR (CDCl_3_, 600 MHz) *δ*/ppm: 7.44 (d, *J* = 8.1 Hz, 2H), 7.39 (d, *J* = 1.6 Hz, 1H), 6.73–6.68 (m, 2H), 6.42 (d, *J* = 12.9 Hz, 1H), 6.33 (d, *J* = 2.5 Hz, 2H), 6.18 (d, *J* = 12.9 Hz, 1H), 2.98 (s, 6H).
(*Z*)-3-(4-methylstyryl)furan (*cis*-**56**): 214 mg, 40% isolated yield; yellow oil; *R_f_* (PE/E (10%)) = 0.86; ^1^H NMR (CDCl_3_, 600 MHz) *δ*/ppm: 7.36 (s, 1H), 7.22 (d, *J* = 7.9 Hz, 2H), 7.23 (d, *J* = 1.4 Hz, 1H), 7.11 (d, *J* = 7.9 Hz, 2H), 6.51 (d, *J* = 12.2 Hz, 1H), 6.33 (d, *J* = 12.2 Hz, 1H), 6.16 (d, *J* = 1.4 Hz, 1H), 2.35 (s, 3H); ^13^C NMR (CDCl_3_, 150 MHz) *δ*/ppm: 142.5, 141.9, 136.9, 134.9, 129.5, 128.9, 128.6, 122.4, 119.5, 110.3, 21.2.
(*E*)-3-(4-methylstyryl)furan (*trans*-**56**): 219 mg, 41% isolated yield; white powder; *R_f_* (PE/E (10%)) = 0.81; ^1^H NMR (CDCl_3_, 600 MHz) *δ*/ppm: 7.51 (s, 1H), 7.40 (t, *J* = 1.7 Hz, 1H), 7.34 (d, *J* = 8.1 Hz, 2H), 7.14 (d, *J* = 8.1 Hz, 2H), 6.92 (d, *J* = 16.2 Hz, 1H), 6.78 (d, *J* = 16.2 Hz, 1H), 6.65 (d, *J* = 1.7 Hz, 1H), 2.34 (s, 3H); ^13^C NMR (CDCl_3_, 150 MHz) *δ*/ppm: 143.6, 140.7, 137.2, 134.6, 129.3, 128.4, 126.0, 124.7, 117.4, 104.4, 21.2.
(*Z*)-3-(4-methoxylstyryl)furan (*cis*-**57**): 160 mg, 50% isolated yield; white oil; *R_f_* (PE) = 0.80; ^1^H NMR (CDCl_3_, 600 MHz) *δ*/ppm: 7.36 (s, 1H), 7.27 (d, *J* = 8.8 Hz, 2H), 6.85 (d, *J* = 8.8 Hz, 2H), 6.48 (d, *J* = 12.8 Hz, 1H), 6.31 (d, *J* = 12.8 Hz, 1H), 6.18 (s, 1H), 3.81 (s, 3H).
(*E*)-3-(4-methoxylstyryl)furan (*trans*-**57**): 217 mg, 30% isolated yield; white powder; *R_f_* (PE) = 0.75; ^1^H NMR (CDCl_3_, 600 MHz) *δ*/ppm: 7.50 (s, 1H), 7.38 (d, *J* = 9.1 Hz, 3H), 6.88 (d, *J* = 8.8 Hz, 2H), 6.84 (d, *J* = 16.1 Hz, 1H), 6.76 (d, *J* = 16.1 Hz, 1H), 6.64 (s, 1H), 3.82 (s, 3H); ^13^C NMR (CDCl_3_, 75 MHz) *δ*/ppm: 143.6, 140.5, 131.3, 130.2, 127.9, 127.3, 124.7, 116.4, 114.2, 107.4, 55.3.

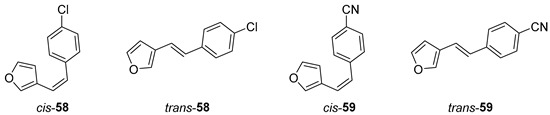

(*Z*)-3-(4-chlorostyryl)furan (*cis*-**58**): 256 mg, 63% isolated yield; colorless oil; *R_f_* (PE) = 0.64; ^1^H NMR (CDCl_3_, 600 MHz) *δ*/ppm: 7.36 (s, 1H), 7.30–7.26 (m, 4H), 7.25 (d, *J* = 1.3 Hz, 1H), 6.47 (d, *J* = 12.1 Hz, 1H), 6.39 (d, *J* = 12.1 Hz, 1H), 6.11 (d, *J* = 1.3 Hz, 1H); ^13^C NMR (CDCl_3_, 150 MHz) *δ*/ppm: 142.7, 142.2, 136.3, 132.9, 130.0, 128.4, 128.1, 121.9, 120.8, 110.0.
(*E*)-3-(4-chlorostyryl)furan (*trans*-**58**): 95 mg, 21% isolated yield; white powder; *R_f_* (PE) = 0.43; ^1^H NMR (CDCl_3_, 600 MHz) *δ*/ppm: 7.54 (s, 1H), 7.41 (d, *J* = 1.1 Hz, 1H), 7.37 (d, *J* = 8.5 Hz, 2H), 7.29 (d, *J* = 8.5 Hz, 2H), 6.94 (d, *J* = 16.3 Hz, 1H), 6.75 (d, *J* = 16.3 Hz, 1H), 6.64 (d, *J* = 1.1 Hz, 1H).
(*Z*)-4-(2-(furan-3-yl)vinyl)benzonitrile (*cis*-**59**): 278 mg, 61% isolated yield; colorless oil; *R_f_* (PE/E (5%)) = 0.54; ^1^H NMR (CDCl_3_, 600 MHz) *δ*/ppm: 7.60 (d, *J* = 8.4 Hz, 2H), 7.44 (d, *J* = 8.4 Hz, 2H), 7.39 (d, *J* = 1.2 Hz, 1H), 7.27 (s, 1H), 6.53 (d, *J* = 12.4 Hz, 1H), 6.48 (d, *J* = 12.4 Hz, 1H), 6.06 (d, *J* = 1.2 Hz, 1H); ^13^C NMR (CDCl_3_, 150 MHz) *δ*/ppm: 143.1, 142.7, 132.5, 132.1, 129.5, 127.4, 126.5, 124.0, 122.7, 121.6, 109.8.
(*E*)-4-(2-(furan-3-yl)vinyl)benzonitrile (*trans*-**59**): 134 mg, 30% isolated yield; white powder; *R_f_* (PE/E (5%)) = 0.33; ^1^H NMR (CDCl_3_, 600 MHz) *δ*/ppm: 7.61 (d, *J* = 8.5 Hz, 2H), 7.51 (d, *J* = 8.5 Hz, 2H), 7.60 (s, 1H), 7.44 (d, *J* = 1.3 Hz, 1H), 7.09 (d, *J* = 16.2 Hz, 1H), 6.79 (d, *J* = 16.2 Hz, 1H), 6.66 (d, *J* = 1.3 Hz, 1H); ^13^C NMR (CDCl_3_, 150 MHz) *δ*/ppm: 144.1, 142.1, 141.9, 134.5, 126.5, 124.0, 122.2, 119.1, 110.3, 107.2.

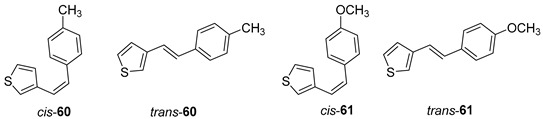

(*Z*)-3-(4-methylstyryl)thiophene (*cis*-**60**): 377 mg, 44% isolated yield; colorless oil; *R_f_* (PE) = 0.32; ^1^H NMR (CDCl_3_, 300 MHz) *δ*/ppm: 7.19 (d, *J* = 8.1 Hz, 2H), 7.14–7.11 (m, 2H), 7.09 (d, *J* = 8.1 Hz, 2H), 6.89 (d, *J* = 4.8 Hz, 1H), 6.54 (d, *J* = 12.3 Hz, 1H), 6.49 (d, *J* = 12.3 Hz, 1H), 2.34 (s, 3H); ^13^C NMR (CDCl_3_, 150 MHz) *δ*/ppm: 138.4, 136.9, 134.8, 129.5, 129.0, 128.6, 128.1, 124.8, 123.8, 123.8, 21.3.
(*E*)-3-(4-methylstyryl)thiophene (*trans*-**60**): 289 mg, 33% isolated yield; white powder; *R_f_* (PE) = 0.21; ^1^H NMR (CDCl_3_, 300 MHz) *δ*/ppm: 7.37 (d, *J* = 8.1 Hz, 2H), 7.33–7.29 (m, 2H), 7.24–7.22 (m, 1H), 7.15 (d, *J* = 8.0 Hz, 2H), 7.08 (d, *J* = 16.3 Hz, 1H), 6.92 (d, *J* = 16.3 Hz, 1H), 2.35 (s, 3H); ^13^C NMR (CDCl_3_, 150 MHz) *δ*/ppm: 140.3, 137.3, 134.6, 129.4, 128.6, 126.2, 126.1, 124.9, 122.0, 121.9, 21.2.
(*Z*)-3-(4-methoxystyryl)thiophene (*cis*-**61**): 244 mg, 40% isolated yield; colorless oil; *R_f_* (PE) = 0.80; ^1^H NMR (CDCl_3_, 600 MHz) *δ*/ppm: 7.37 (s, 1H), 7.26 (d, *J* = 9.2 Hz, 2H), 7.25 (t, *J* = 1.7 Hz, 1H), 6.85 (d, 1H, *J* = 8.8 Hz, 2H), 6.48 (d, *J* = 11.8 Hz, 1H), 6.31 (d, *J* = 11.8 Hz, 1H), 6.18 (d, *J* = 5.1 Hz, 1H), 3.81 (s, 3H); ^13^C NMR (CDCl_3_, 150 MHz) *δ*/ppm: 158.7, 142.5, 141.8, 130.3, 129.8, 129.2, 122.4, 118.9, 113.6, 110.3, 100.0, 55.2.
(*E*)-3-(4-methoxystyryl)thiophene (*trans*-**61**): 248 mg, 41% isolated yield; white powder; *R_f_* (PE) = 0.76; ^1^H NMR (CDCl_3_, 600 MHz) *δ*/ppm: 7.41 (d, *J* = 8.7 Hz, 2H), 7.34–7.29 (m, 2H), 7.22–7.20 (m, 1H), 7.00 (d, *J* = 16.2 Hz, 1H), 6.91 (d, *J* = 16.2 Hz, 1H), 6.89 (d, *J* = 8.7 Hz, 2H), 3.82 (s, 3H); ^13^C NMR (CDCl_3_, 75 MHz) *δ*/ppm: 159.2, 140.4, 130.2, 128.2, 127.5, 126.1, 124.9, 121.5, 120.9, 114.2, 55.3.

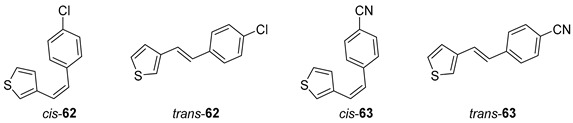

(*Z*)-3-(4-chlorostyryl)thiophene (*cis*-**62**): 222 mg, 41% isolated yield; colorless oil; *R_f_* (PE) = 0.64; ^1^H NMR (CDCl_3_, 600 MHz) *δ*/ppm: 7.23 (d, *J* = 8.7 Hz, 2H), 7.21 (d, *J* = 8.4 Hz, 2H), 7.15 (dd, *J* = 5.1, 2.9 Hz, 1H), 7.11 (d, *J* = 2.9 Hz, 1H), 6.85 (d, *J* = 5.1 Hz, 1H), 6.56 (d, *J* = 12.1 Hz, 1H), 6.48 (d, *J* = 12.1 Hz, 1H); ^13^C NMR (CDCl_3_, 150 MHz) *δ*/ppm: 137.9, 136.2, 132.9, 130.1, 128.5, 128.2, 127.8, 125.2, 125.1, 124.3.
(*E*)-3-(4-chlorostyryl)thiophene (*trans*-**62**): 186.1 mg, 34% isolated yield; white powder; *R_f_* (PE) = 0.49; ^1^H NMR (CDCl_3_, 300 MHz) *δ*/ppm: 7.40 (d, *J* = 8.7 Hz, 2H), 7.33–7.27 (m, 5H), 7.09 (d, *J* = 16.3 Hz, 1H), 6.89 (d, *J* = 16.3 Hz, 1H); ^13^C NMR (CDCl_3_, 150 MHz) *δ*/ppm: 139.8, 135.9, 133.0, 128.8, 127.4, 127.3, 126.3, 124.8, 123.5, 122.7.
(*Z*)-4-(2-(thiophen-3-yl)vinyl)benzonitrile (*cis*-**63**): 474 mg, 73% isolated yield; colorless oil; *R_f_* (PE/E (15%)) = 0.69; ^1^H NMR (CDCl_3_, 300 MHz) *δ*/ppm: 7.56 (d, *J* = 8.3 Hz, 2H), 7.39 (d, *J* = 8.1 Hz, 2H), 7.18 (dd, *J* = 4.9, 3.0 Hz, 1H), 7.13 (d, *J* = 2.9 Hz, 1H), 6.81 (d, *J* = 5.0 Hz, 1H), 6.68 (d, *J* = 12.1 Hz, 1H), 5.52 (d, *J* = 12.1 Hz, 1H); ^13^C NMR (CDCl_3_, 75 MHz) *δ*/ppm: 142.6, 137.3, 132.0, 132.1, 129.5, 127.5, 127.1, 125.6, 125.0, 118.9, 110.6.
  (*E*)-4-(2-(thiophen-3-yl)vinyl)benzonitrile (*trans*-**63**): 158 mg, 23% isolated yield; white powder; *R_f_* (PE/E (15%)) = 0.48; ^1^H NMR (CDCl_3_, 300 MHz) *δ*/ppm: 7.62 (d, *J* = 8.4 Hz, 2H), 7.54 (d, *J* = 8.4 Hz, 2H), 7.36 (s, 2H), 7.26 (s, 1H), 7.23 (d, *J* = 16.3 Hz, 1H), 6.93 (d, *J* = 16.3 Hz, 1H); ^13^C NMR (CDCl_3_, 75 MHz) *δ*/ppm: 142.0, 139.2, 132.5, 126.7, 126.6, 126.6, 126.4, 124.8, 124.3, 119.1, 110.4.
The corresponding amines (1.2 eq) were added to a solution of triazole nitro aldehyde **24** [18,19] (1 eq) in dry dioxane and purged with argon, Ar. The reaction mixtures were stirred at 130 °C. After being left overnight, the reaction mixtures were cooled to room temperature and evaporated to dryness to obtain crude products **22**, **23**, and **25**. The crude products were purified using column chromatography with an E/EtOAc solvent system.

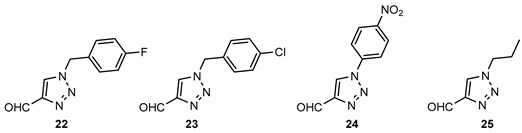

1-(4-fluorobenzyl)-1*H*-1,2,3-triazole-4-carbaldehyde (**22**): 128 mg, 78% isolated yield; yellow oil; *R_f_* (DCM/EtOAc (2%)) = 0.53; ^1^H NMR (CDCl_3_, 600 MHz) *δ*/ppm: 10.13 (s, 1H), 7.99 (s, 1H), 7.33–7.30 (m, 2H), 7.13–7.08 (m, 2H), 5.57 (s, 2H).
1-(4-chlorobenzyl)-1*H*-1,2,3-triazole-4-carbaldehyde (**23**): 85 mg, 68% isolated yield; yellow oil; *R_f_* (DCM/EtOAc (2%)) = 0.50; ^1^H NMR (CDCl_3_, 600 MHz) *δ*/ppm: 10.13 (s, 1H), 8.01 (s, 1H), 7.41–7.38 (m, 4H), 5.57 (s, 2H).
1-(4-nitrophenyl)-1*H*-1,2,3-triazole-4-carbaldehyde (**24**) [12]: 720 mg, 72% isolated yield; yellow powder; *R_f_* (DCM/EtOAc (2%)) = 0.52; ^1^H NMR (CDCl_3_, 600 MHz) *δ*/ppm: 10.20 (s, 1H), 8.66 (s, 1H), 8.49 (d, *J* = 8.8 Hz, 2H), 8.04 (d, *J* = 9.0 Hz, 2H).
  1-propyl-1*H*-1,2,3-triazole-4-carbaldehyde (**25**): 100 mg, 40% isolated yield; yellow oil; *R_f_* (DCM/EtOAc (2%)) = 0.55; ^1^H NMR (CDCl_3_, 600 MHz) *δ*/ppm: 10.14 (s, 1H), 8.15 (s, 1H), 4.95–4.88 (m, 1H), 1.65 (s, 3H), 1.64 (s, 3H).
The obtained heterostilbenes **45**–**63** were subjected to the Vilsmeier formylation reaction. The selected heterostilbenes, as mixtures of isomers, were dissolved in 2 mL of DMF and stirred for 10 min at 10 °C. This temperature was achieved using a water bath with a few ice cubes and monitored with a thermometer. The weighed amount of POCl_3_ was slowly added dropwise. After 30 min, the water bath was removed, and the reaction mixture was allowed to stir. Upon completion of the reaction, the reaction mixture was neutralized using a 10% NaOH solution. When neutralization was achieved, extraction was carried out using E and water. The combined organic layer was washed with water and dried over MgSO_4_, filtered, and the solvent was evaporated. The dry reaction mixture was purified by column chromatography on silica gel using a PE/E or PE/DCM variable polarity eluent. In the first fractions, the unreacted substrates were isolated (as *cis*-isomers), while in the last fractions, the desired formyl derivatives **26**–**44** were obtained (mainly as *trans*-isomers). They were further used in the preparation of oximes **4**–**21**.

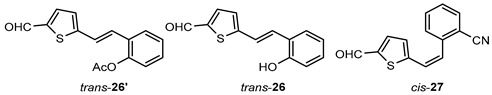

(*E*)-2-(2-(5-formylthiophen-2-yl)vinyl)phenyl acetate (*trans*-**26′**): 128 mg, 78% isolated yield; yellow oil; *R_f_* (DCM) = 0.36; ^1^H NMR (CDCl_3_, 600 MHz) *δ*/ppm: 9.86 (s, 1H), 7.65–7.64 (m, 2H), 7.33 (t, *J* = 7.9 Hz, 1H), 7.27–7.24 (m, 1H), 7.21 (d, *J* = 16.1 Hz, 1H), 7.17 (d, *J* = 16.1 Hz, 1H), 7.14 (d, *J* = 3.9 Hz, 1H), 7.11 (d, *J* = 8.0 Hz, 1H), 2.39 (s, 3H); ^13^C NMR (CDCl_3_, 150 MHz) *δ*/ppm: 182.7, 169.2, 152.1, 148.5, 141.9, 137.2, 129.6, 128.5, 127.2, 126.4, 125.9, 123.0, 122.9, 99.9, 20.9.
(*E*)-5-(2-hydroxystyryl)thiophene-2-carbaldehyde (*trans*-**26**): 60 mg, 50% isolated yield; yellow powder; *R_f_* (E) = 0.26; ^1^H NMR (CDCl_3_, 600 MHz) *δ*/ppm: 9.85 (s, 1H), 7.67 (d, *J* = 3.7 Hz, 1H), 7.50 (dd, *J* = 7.7, 1.4 Hz, 1H), 7.47 (d, *J* = 16.2 Hz, 1H), 7.31 (d, *J* = 16.2 Hz, 1H), 7.19 (t, *J* = 7.6 Hz, 1H), 7.16 (d, *J* = 3.8 Hz, 1H), 6.96 (t, *J* = 7.4 Hz, 1H), 6.82 (d, *J* = 8.1 Hz, 1H), 5.38 (s, 1H); ^13^C NMR (CDCl_3_, 150 MHz) *δ*/ppm: 182.8, 153.7, 153.5, 141.3, 137.5, 129.8, 128.0, 127.7, 126.4, 123.3, 121.8, 121.2, 116.2.
(*Z*)-2-(2-(5-formylthiophen-2-yl)vinyl)benzonitrile (*cis*-**27**): 10 mg, 20% isolated yield; yellow oil; *R_f_* (DCM) = 0.40; ^1^H NMR (CDCl_3_, 600 MHz) *δ*/ppm: 9.79 (s, 1H), 7.75 (d, *J* = 7.7 Hz, 1H), 7.61 (t, *J* = 8.1 Hz, 1H), 7.57 (d, *J* = 4.6 Hz, 1H), 7.51–7.47 (m, 2H), 7.05 (d, *J* = 4.1 Hz, 1H), 6.96 (d, *J* = 11.9 Hz, 1H), 6.87 (d, *J* = 11.9 Hz, 1H); ^13^C NMR (CDCl_3_, 150 MHz) *δ*/ppm: 182.9, 147.6, 143.5, 140.1, 135.9, 133.5, 130.3, 129.6, 128.8, 126.4, 117.4, 112.3.

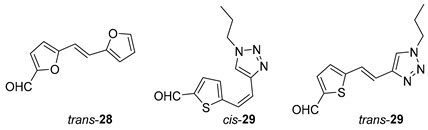

(*E*)-5-(2-(furan-2-yl)vinyl)furan-2-carbaldehyde (*trans*-**28**): 74 mg, 60% isolated yield; red oil; *R_f_* (PE/E (20%)) = 0.85; ^1^H NMR (CDCl_3_, 300 MHz) *δ*/ppm: 9.57 (s, 1H), 7.44 (d, *J* = 1.2 Hz, 1H), 7.26–7.23 (m, 1H), 7.16 (d, *J* = 16.2 Hz, 1H), 6.82 (d, *J* = 16.2 Hz, 1H), 6.49–6.45 (m, 3H).
(*Z*)-5-(2-(1-propyl-1*H*-1,2,3-triazol-4-yl)vinyl)thiophene-2-carbaldehyde (*cis*-**29**): 25 mg, 25% isolated yield; yellow oil; *R_f_* (PE/E (9%)) = 0.25; ^1^H NMR (CDCl_3_, 600 MHz) *δ*/ppm: 9.89 (s, 1H), 7.69 (d, *J* = 4.1 Hz, 1H), 7.63 (s, 1H), 7.51 (d, *J* = 4.1 Hz, 1H), 6.72 (d, *J* = 12.4 Hz, 1H), 6.69 (d, *J* = 12.4 Hz, 1H), 4.36–4.33 (m, 2H), 2.00–1.93 (m, 2H), 1.00–0.96 (m, 3H).
(*E*)-5-(2-(1-propyl-1*H*-1,2,3-triazol-4-yl)vinyl)thiophene-2-carbaldehyde (*trans*-**29**): 25 mg, 25% isolated yield; yellow oil; *R_f_* (PE/E (90%)) = 0.23; ^1^H NMR (CDCl_3_, 600 MHz) *δ*/ppm: 9.87 (s, 1H), 7.67 (d, *J* = 4.1 Hz, 1H), 7.57 (s, 1H), 7.51 (d, *J* = 15.9 Hz, 1H), 7.17 (d, *J* = 4.8 Hz, 1H), 7.11 (d, *J* = 15.9 Hz, 1H), 4.36–4.33 (m, 2H), 2.00–1.93 (m, 2H), 1.00–0.96 (m, 3H).

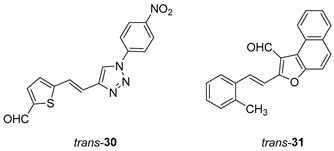

(*E*)-5-(2-(1-(4-nitrophenyl)-1*H*-1,2,3-triazol-4-yl)vinyl)thiophene-2-carbaldehyde (*trans*-**30**): 12 mg, 10% isolated yield; yellow oil; *R_f_* (DCM) = 0.15; ^1^H NMR (CDCl_3_, 600 MHz) *δ*/ppm: 9.82 (s, 1H), 8.45 (d, *J* = 9.1 Hz, 2H), 8.11 (s, 1H), 8.00 (d, *J* = 9.6 Hz, 2H), 7.69 (d, *J* = 1.7 Hz, 1H), 7.68 (d, *J* = 14.1 Hz, 1H), 7.23 (d, *J* = 3.6 Hz, 1H), 7.68 (d, *J* = 14.1 Hz, 1H).
(*E*)-2-(2-methylstyryl)naphtho[2,1-*b*]furan-1-carbaldehyde (*trans*-**31**): 75 mg, 75% isolated yield; yellow powder; *R_f_* (PE/E (50%)) = 0.75; ^1^H NMR (CDCl_3_, 600 MHz) *δ*/ppm: 10.67 (s, 1H), 9.30 (d, *J* = 8.3 Hz, 1H), 7.97 (d, *J* = 15.9 Hz, 1H), 7.95 (d, *J* = 5.9 Hz, 1H), 7.86 (d, *J* = 9.3 Hz, 1H), 7.75–7.63 (m, 4H), 7.58–7.54 (m, 2H), 7.31–7.29 (m, 2H), 2.55 (s, 3H).

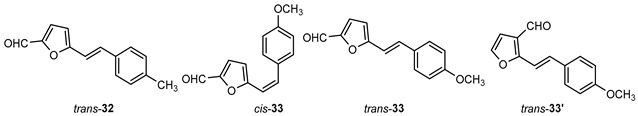

(*E*)-5-(4-methylstyryl)furan-2-carbaldehyde (*trans*-**32**): 693 m, 76% isolated yield; white powder; *R_f_* (PE/E (2%)) = 0.15; ^1^H NMR (CDCl_3_, 600 MHz) *δ*/ppm: 9.58 (s, 1H), 7.40 (d, *J* = 8.2 Hz, 2H), 7.36 (d, *J* = 16.5 Hz, 1H), 7.24 (d, *J* = 3.8 Hz, 1H), 7.18 (d, *J* = 7.7 Hz, 2H), 6.88 (d, *J* = 16.5 Hz, 1H), 6.50 (d, *J* = 3.8 Hz, 1H), 2.36 (s, 3H).
(*Z*)-5-(4-methylstyryl)furan-2-carbaldehyde (*cis*-**33**): 17 mg, 4% isolated yield; colorless oil; *R_f_* (PE/E (1%)) = 0.27; ^1^H NMR (CDCl_3_, 600 MHz) *δ*/ppm: 9.54 (s, 1H), 7.42 (d, *J* = 8.5 Hz, 2H), 7.13 (d, *J* = 3.8 Hz, 1H), 6.89 (d, *J* = 8.7 Hz, 2H), 6.73 (d, *J* = 12.4 Hz, 1H), 6.41 (d, *J* = 3.8 Hz, 1H), 6.36 (d, *J* = 12.4 Hz, 1H), 3.84 (s, 3H); ^13^C NMR (CDCl_3_, 600 MHz) *δ*/ppm: 177.4, 159.7, 157.8, 151.1, 134.3, 130.2, 128.7, 115.9, 113.9, 111.6, 55.3.
(*E*)-5-(4-methoxystyryl)furan-2-carbaldehyde (*trans*-**33**): 90 mg, 17% isolated yield; white powder; *R_f_* (PE/E (1%)) = 0.22; ^1^H NMR (CDCl_3_, 600 MHz) *δ*/ppm: 9.56 (s, 1H), 7.44 (d, *J* = 8.6 Hz, 2H), 7.34 (d, *J* = 16.1 Hz, 1H), 7.24 (d, *J* = 4.0 Hz, 1H), 6.90 (d, *J* = 9.1 Hz, 2H), 6.78 (d, *J* = 16.1 Hz, 1H), 6.47 (d, *J* = 4.0 Hz, 1H), 3.82 (s, 3H); ^13^C NMR (CDCl_3_, 600 MHz) *δ*/ppm: 176.7, 160.3, 159.3, 151.4, 133.1, 133.2, 128.4, 114.3, 112.9, 109.9, 55.4.
(*E*)-2-(4-methoxystyryl)furan-3-carbaldehyde (*trans*-**33′**): 60 mg, 26% isolated yield; white powder; *R_f_* (PE/E (1%)) = 0.15; ^1^H NMR (CDCl_3_, 600 MHz) *δ*/ppm: 9.91 (s, 1H), 7.57 (d, *J* = 1.9 Hz, 1H), 7.49 (d, *J* = 9.1 Hz, 2H), 7.47 (d, *J* = 16.9 Hz, 1H), 7.09 (d, *J* = 16.9 Hz, 1H), 6.91 (d, *J* = 8.8 Hz, 2H), 6.83 (d, *J* = 1.5 HZ, 1H), 3.84 (s, 3H).

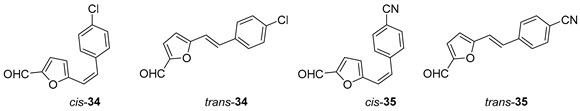

(*Z*)-5-(4-chlorostyryl)furan-2-carbaldehyde (*cis*-**34**): 128 mg, 21% isolated yield; yellow oil; *R_f_* (PE/E (1%)) = 0.23; ^1^H NMR (CDCl_3_, 600 MHz) *δ*/ppm: 9.54 (s, 1H), 7.38 (d, *J* = 8.4 Hz, 2H), 7.34 (d, *J* = 8.4 Hz, 2H), 7.13 (d, *J* = 3.6 Hz, 1H), 6.73 (d, *J* = 13.1 Hz, 1H), 6.47 (d, *J* = 13.1 Hz, 1H), 6.34 (d, *J* = 3.6 Hz, 1H); ^13^C NMR (CDCl_3_, 600 MHz) *δ*/ppm: 177.3, 156.9, 151.4, 134.7, 134.2, 132.9, 129.9, 128.7, 117.9, 112.3.
(*E*)-5-(4-chlorostyryl)furan-2-carbaldehyde (*trans*-**34**): 62.7 mg, 11% isolated yield; white powder; *R_f_* (PE/E (1%)) = 0.18; ^1^H NMR (CDCl_3_, 600 MHz) *δ*/ppm: 9.57 (s, 1H), 7.41 (d, *J* = 8.7 Hz, 2H), 7.32 (d, *J* = 8.7 Hz, 2H), 7.31 (d, *J* = 15.9 Hz, 1H), 7.24 (d, *J* = 3.5 Hz, 1H), 6.88 (d, *J* = 15.9 Hz, 1H), 6.53 (d, *J* = 3.7 Hz, 1H); ^13^C NMR (CDCl_3_, 600 MHz) *δ*/ppm: 176.9, 158.2, 151.8, 134.6, 134.3, 131.8, 129.1, 128.1, 115.5, 111.0.
(*Z*)-4-(2-(5-formylfuran-2-yl)vinyl)benzonitrile (*cis*-**35**): 21 mg, 3% isolated yield; white oil; *R_f_* (PE/E (20%)) = 0.33; ^1^H NMR (CDCl_3_, 600 MHz) *δ*/ppm: 9.54 (s, 1H), 7.67 (d, *J* = 7.7 Hz, 2H), 7.55 (d, *J* = 7.8 Hz, 2H), 7.16 (d, *J* = 3.8 Hz, 1H), 6.76 (d, *J* = 12.3 Hz, 1H), 6.56 (d, *J* = 12.3 Hz, 1H), 6.37 (d, *J* = 3.8 Hz, 1H).
(*E*)-4-(2-(5-formylfuran-2-yl)vinyl)benzonitrile (*trans*-**35**): 62 mg, 9% isolated yield; white powder; *R_f_* (PE/E (20%)) = 0.23; ^1^H NMR (CDCl_3_, 600 MHz) *δ*/ppm: 9.64 (s, 1H), 7.67 (d, *J* = 7.9 Hz, 2H), 7.57 (d, *J* = 8.3 Hz, 2H), 7.37 (d, *J* = 16.5 Hz, 1H), 7.28 (d, *J* = 3.8 Hz, 1H), 7.03 (d, *J* = 16.5 Hz, 1H), 6.64 (d, *J* = 3.8 Hz, 1H).

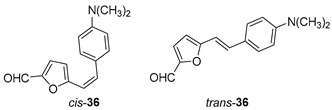

(*Z*)-5-(4-(dimethylamino)styryl)furan-2-carbaldehyde (*cis*-**36**): 10 mg; 2% isolated yield; yellow oil; *R_f_* (PE/E (5%)) = 0.16; ^1^H NMR (CDCl_3_, 600 MHz) *δ*/ppm: 9.55 (s, 1H), 7.45 (d, *J =* 8.5 Hz, 2H), 7.17 (d, *J* = 3.7 Hz, 1H), 6.70 (d, *J* = 8.7 Hz, 2H), 6.69 (d, *J* = 12.4 Hz, 1H), 6.52 (d, *J* = 3.6 Hz, 1H), 6.23 (d, *J* = 12.4 Hz, 1H), 3.01 (s, 6H).
(*E*)-5-(4-(dimethylamino)styryl)furan-2-carbaldehyde (*trans*-**36**): 5 mg; 5% isolated yield; yellow powder; *R_f_* (PE/E (5%)) = 0.15; ^1^H NMR (CDCl_3_, 600 MHz) *δ*/ppm: 9.53 (s, 1H), 7.41 (d, *J* = 8.6 Hz, 2H), 7.33 (d, *J* = 16.2 Hz, 1H), 7.24 (d, *J* = 3.6 Hz, 1H), 7.20 (d, *J* = 16.2 Hz, 1H), 6.70 (d, *J* = 8.7 Hz, 2H), 6.43 (d, *J* = 3.7 Hz, 1H), 3.01 (s, 6H).

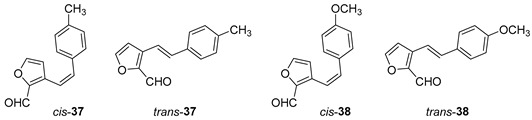

(*Z*)-3-(4-methylstyryl)furan-2-carbaldehyde (*cis*-**37**): 151 mg, 43% isolated yield; orange oil; *R_f_* (PE/E (5%)) = 0.40; ^1^H NMR (CDCl_3_, 600 MHz) *δ*/ppm: 9.81 (s, 1H), 7.43 (d, *J* = 1.2 Hz, 1H), 7.18 (d, *J* = 8.1 Hz, 2H), 7.11 (d, *J* = 8.1 Hz, 2H), 6.85 (d, *J* = 12.4 Hz, 1H), 6.85 (d, *J* = 12.4 Hz, 1H), 6.27 (d, *J* = 1.2 Hz, 1H), 2.35 (s, 3H); ^13^C NMR (CDCl_3_, 150 MHz) *δ*/ppm: 178.2, 148.5, 146.8, 138.0, 135.7, 133.6, 129.1, 128.7, 127.0, 117.4, 112.9, 21.3.
(*E*)-3-(4-methylstyryl)furan-2-carbaldehyde (*trans*-**37**): 103 mg, 51% isolated yield; yellow powder; *R_f_* (PE/E (5%)) = 0.26; ^1^H NMR (CDCl_3_, 600 MHz) *δ*/ppm: 9.91 (s, 1H), 7.58 (d, *J* = 1.3 Hz, 1H), 7.50 (d, *J* = 16.2 Hz, 1H), 7.45 (d, *J* = 7.8 Hz, 2H), 7.19 (d, *J* = 7.8 Hz, 2H), 7.11 (d, *J* = 16.2 Hz, 1H), 6.85 (d, *J* = 1.3 Hz, 1H), 2.37 (s, 3H); ^13^C NMR (CDCl_3_, 150 MHz) *δ*/ppm: 178.5, 147.7, 147.5, 139.0, 135.3, 133.4, 129.5, 129.1, 128.7, 127.1, 109.8, 21.3.
(*Z*)-3-(4-methoxystyryl)furan-2-carbaldehyde (*cis*-**38**): 90 mg, 30% isolated yield; yellow oil; *R_f_* (PE/DCM (30%)) = 0.30; ^1^H NMR (CDCl_3_, 300 MHz) *δ*/ppm: 9.81 (s, 1H), 7.44 (s, 1H), 7.26–7.21 (m, 2H), 6.85–6.76 (m, 4H), 6.31 (d, *J* = 1.6 Hz, 1H), 3.82 (s, 3H).
(*E*)-3-(4-methoxystyryl)furan-2-carbaldehyde (*trans*-**38**): 135 mg, 56% isolated yield; yellow powder; *R_f_* (PE/DCM (30%)) = 0.25; ^1^H NMR (CDCl_3_, 300 MHz) *δ*/ppm: 9.90 (s, 1H), 7.57–7.44 (m, 4H), 7.09 (d, *J* = 15.0 Hz, 1H), 6.93–6.83 (m, 3H), 3.84 (s, 3H).

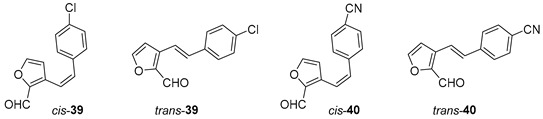

(*Z*)-3-(4-chlorostyryl)furan-2-carbaldehyde (*cis*-**39**): 187 mg, 67% isolated yield; orange oil; *R_f_* (PE/E (10%)) = 0.30; ^1^H NMR (CDCl_3_, 600 MHz) *δ*/ppm: 9.83 (s, 1H), 7.44 (d, *J* = 1.6 Hz, 1H), 7.29 (d, *J* = 8.6 Hz, 2H), 7.22 (d, *J* = 8.6 Hz, 2H), 6.94 (d, *J* = 12.0 Hz, 1H), 7.82 (d, *J* = 12.0 Hz, 1H), 6.22 (d, *J* = 1.6 Hz, 1H).
(*E*)-3-(4-chlorostyryl)furan-2-carbaldehyde (*trans*-**39**): 58 mg, 23% isolated yield; yellow powder; *R_f_* (PE/E (5%)) = 0.20; ^1^H NMR (CDCl_3_, 600 MHz) *δ*/ppm: 9.92 (s, 1H), 7.62 (d, *J* = 16.3 Hz, 1H), 7.59 (d, *J* = 1.5 Hz, 1H), 7.48 (d, *J* = 8.5 Hz, 2H), 7.35 (d, *J* = 8.5 Hz, 2H), 7.08 (d, *J* = 16.3 Hz, 1H), 6.85 (d, *J* = 1.5 Hz, 1H); ^13^C NMR (CDCl_3_, 150 MHz) *δ*/ppm: 178.5, 147.6, 146.9, 134.2, 133.8, 130.1, 129.0, 128.7, 128.2, 112.8, 109.9.
(*Z*)-4-(2-(2-formylfuran-3-yl)vinyl)benzonitrile (*cis*-**40**): 175 mg, 68% isolated yield; yellow oil; *R_f_* (PE/E (20%)) = 0.55; ^1^H NMR (CDCl_3_, 600 MHz) *δ*/ppm: 9.85 (s, 1H), 7.61 (d, *J* = 8.3 Hz, 2H), 7.47 (d, *J* = 1.6 Hz, 1H), 7.40 (d, *J* = 8.3 Hz, 2H), 7.07 (d, *J* = 12.3 Hz, 1H), 6.85 (d, *J* = 12.3 Hz, 1H), 6.15 (d, *J* = 1.6 Hz, 1H); ^13^C NMR (CDCl_3_, 150 MHz) *δ*/ppm: 178.7, 148.9, 147.0, 133.2, 132.6, 132.3, 129.5, 127.4, 121.1, 112.6, 111.6, 109.9.
(*E*)-4-(2-(2-formylfuran-3-yl)vinyl)benzonitrile (*trans*-**40**): 119 mg, 45% isolated yield; yellow powder; *R_f_* (PE/E (20%)) = 0.46; ^1^H NMR (CDCl_3_, 600 MHz) *δ*/ppm: 9.94 (s, 1H), 7.76 (d, *J* = 16.5 Hz, 1H), 7.66 (d, *J* = 8.5 Hz, 2H), 7.63 (d, *J* = 8.5 Hz, 2H), 7.62 (d, *J* = 1.9 Hz, 1H), 7.12 (d, *J* = 16.5 Hz, 1H), 6.87 (d, *J* = 1.9 Hz, 1H); ^13^C NMR (CDCl_3_, 150 MHz) *δ*/ppm: 179.4, 148.4, 147.6, 140.6, 132.9, 132.6, 127.4, 120.6, 118.8, 111.7, 109.9.

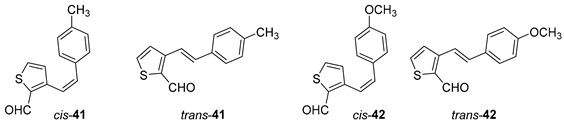

(*Z*)-3-(4-methylstyryl)thiophene-2-carbaldehyde (*cis*-**41**): 110 mg, 27% isolated yield; *R_f_* (PE/E (10%)) = 0.57; ^1^H NMR (CDCl_3_, 300 MHz) *δ*/ppm: 9.99 (s, 1H), 7.56 (d, *J* = 4.9 Hz, 1H), 7.06 (t, *J* = 9.4 Hz, 4H), 6.92 (d, *J* = 5.0 Hz, 1H), 6.84 (d, *J* = 12.1 Hz, 1H), 6.78 (d, *J* = 12.1 Hz, 1H), 2.31 (s, 3H).
(*E*)-3-(4-methylstyryl)thiophene-2-carbaldehyde (*trans*-**41**): 186 mg, 64% isolated yield; white powder; *R_f_* (PE/E (10%)) = 0.42; ^1^H NMR (CDCl_3_, 300 MHz) *δ*/ppm: 10.21 (s, 1H), 7.65 (d, *J* = 5.6 Hz, 1H), 7.63 (d, *J* = 16.2 Hz, 1H), 7.43 (d, *J* = 6.6 Hz, 3H), 7.20 (d, *J* = 8.0 Hz, 2H), 7.14 (d, *J* = 16.2 Hz, 1H), 2.37 (s, 3H).
(*Z*)-3-(4-methoxystyryl)thiophene-2-carbaldehyde (*cis*-**42**): 45 mg, 20% isolated yield; white oil; *R_f_* (PE/DCM (60%)) = 0.55; ^1^H NMR (CDCl_3_, 300 MHz) *δ*/ppm: 9.99 (s, 1H), 7.57 (d, *J* = 5.2 Hz, 1H), 7.12 (d, *J* = 9.1 Hz, 2H), 6.94 (d, *J* = 4.6 Hz, 1H), 6.82–6.71 (m, 4H), 3.78 (s, 3H).
(*E*)-3-(4-methoxystyryl)thiophene-2-carbaldehyde (*trans*-**42**): 130 mg, 52% isolated yield; white powder; *R_f_* (PE/DCM (60%)) = 0.50; ^1^H NMR (CDCl_3_, 300 MHz) *δ*/ppm: 10.21 (s, 1H), 7.66 (d, *J* = 5.2 Hz, 1H), 7.59–7.42 (m, 7H), 3.85 (s, 3H).

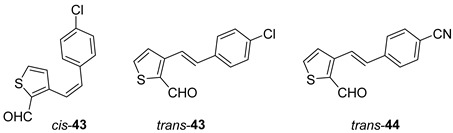

(*Z*)-3-(4-chlorostyryl)thiophene-2-carbaldehyde (*cis*-**43**): 98 mg, 41% isolated yield; white oil; *R_f_* (PE/E (10%)) = 0.19; ^1^H NMR (CDCl_3_, 300 MHz) *δ*/ppm: 9.99 (s, 1H), 7.58 (d, *J* = 4.9 Hz, 1H), 7.22 (d, *J* = 8.5 Hz, 2H), 7.10 (d, *J* = 8.5 Hz, 2H), 6.90–6.87 (m, 3H), 6.86 (d, *J* = 5.0 Hz, 1H), 6.80 (d, *J* = 12.2 Hz, 1H).
(*E*)-3-(4-chlorostyryl)thiophene-2-carbaldehyde (*trans*-**43**): 96 mg, 40% isolated yield; white powder; *R_f_* (PE/E (10%)) = 0.15; ^1^H NMR (CDCl_3_, 300 MHz) *δ*/ppm: 10.19 (s, 1H), 7.85 (d, *J* = 16.5 Hz, 1H), 7.72–7.60 (m, 5H), 7.48 (d, *J* = 4.7 Hz, 1H), 7.15 (d, *J* = 16.5 Hz, 1H).
(*E*)-4-(2-(2-formylthiophen-3-yl)vinyl)benzonitrile (*trans*-**44**): 31 mg, 86% isolated yield; white powder; *R_f_* (PE/E (10%)) = 0.24; ^1^H NMR (CDCl_3_, 300 MHz) *δ*/ppm: 10.19 (s, 1H), 7.86 (d, *J* = 16.2 Hz, 1H), 7.71 (d, *J* = 5.2 Hz, 1H), 7.68 (d, *J* = 8.5 Hz, 2H), 7.63 (d, *J* = 8.5 Hz, 2H), 7.48 (d, *J* = 5.2 Hz, 1H), 7.16 (d, *J* = 16.2 Hz, 1H); ^13^C NMR (CDCl_3_, 150 MHz) *δ*/ppm: 181.9, 149.7, 140.8, 134.5, 139.5, 133.0, 132.6, 132.3, 129.5, 127.3, 123.1, 118.7.
The obtained aldehydes **23**–**25** were converted into oximes **1**–**3**, while aldehydes **26**–**44** produced the corresponding oximes **4**–**21**, according to the literature [20]. Crystals of NH_2_OH × HCl were dissolved in a prepared mixture of 10 mL of EtOH and 3 mL of distilled water. After a homogeneous solution was obtained, the corresponding prepared heterostilbene aldehydes **26**–**44** were added. The reaction mixture was stirred at room temperature for 24 h. When the reaction was completed, the solvent was evaporated on a rotavapor under reduced pressure. The reaction mixture was purified by repeated column chromatography on silica gel using PE/DCM and DCM/methanol variable polarity eluents. The targeted oximes **1**–**21** were isolated, and their spectroscopic data and the yields of their pure isomers are given below.

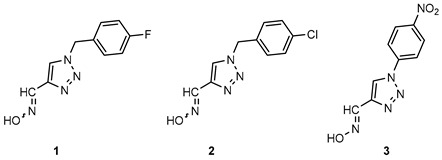

1-(4-fluorobenzyl)-1*H*-1,2,3-triazole-4-carbaldehyde oxime (**1**): 30 mg, 16% isolated yield; yellow oil; *R_f_* (DCM/MeOH (30%)) = 0.22; ^1^H NMR (CD_3_OD, 600 MHz) *δ*/ppm: 8.48 (s, 1H), 8.44 (s, 1H), 8.12 (d, *J* = 9.3 Hz, 2H), 6.87 (d, *J* = 8.2 Hz, 2H), 5.68 (s, 2H). In the mixture, the ratio of the two isomers is approximately 1:1.
1-(4-chlorobenzyl)-1*H*-1,2,3-triazole-4-carbaldehyde oxime (**2**): 30 mg, 8% isolated yield; yellow oil; *R_f_* (DCM/MeOH (30%)) = 0.21; ^1^H NMR (CD_3_OD, 600 MHz) *δ*/ppm: 8.11 (s, 1H), 8.10 (s, 1H), 6.86 (d, *J* = 8.7 Hz, 2H), 6.60 (d, *J* = 8.7 Hz, 2H), 4.81 (s, 2H). In the mixture, the ratio of the *syn*- and *anti*-isomers is approximately 2:1.
1-(4-nitrophenyl)-1*H*-1,2,3-triazole-4-carbaldehyde oxime (**3**): 70 mg, 50% isolated yield; yellow oil; *R_f_* (DCM/MeOH (30%)) = 0.25; ^1^H NMR (CD_3_OD, 600 MHz) *δ*/ppm: 9.29 (s, 1H), 8.48 (d, *J* = 9.4 Hz, 2H), 8.24 (d, *J* = 9.2 Hz, 2H), 7.75 (s, 1H). The majority *syn*-isomer is formed. MS (ESI) *m*/*z* (%, fragment): 234 (5); 121 (100); HRMS (*m*/*z*) for C_9_H_7_N_5_O_3_: [M + H]^+^_calcd_ = 233.0548, and [M + H]^+^_measured_ = 233.0549.

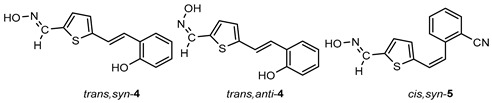

(*E*)-5-((*E*)-2-hydroxystyryl)thiophene-2-carbaldehyde oxime (*trans,syn*-**4**): 18 mg, 25%; *R_f_* (DCM/MeOH (10%)) = 0.52; ^1^H NMR (CDCl_3_, CD_3_OD, 600 MHz) *δ*/ppm: 8.20 (s, 1H), 7.46 (d, *J* = 7.2 Hz, 1H), 7.29 (d, *J* = 16.1 Hz, 2H), 7.25 (d, *J* = 16.1 Hz, 1H), 7.11 (t, *J* = 7.3 Hz, 1H), 7.05 (d, *J* = 3.8 Hz, 1H), 6.97 (d, *J* = 4.2 Hz, 1H), 6.87 (t, *J* = 7.8 Hz, 1H), 6.81 (d, *J* = 8.2 Hz, 1H); ^13^C NMR (CDCl_3_, 600 MHz) *δ*/ppm: 153.9, 149.1, 145.6, 133.9, 132.4, 130.2, 128.9, 127.3, 125.9, 125.1, 122.2, 120.7, 116.0.
(*Z*)-5-((*E*)-2-hydroxystyryl)thiophene-2-carbaldehyde oxime (*trans,anti*-**4**): 41 mg, 52% isolated yield; white powder; m.p. = 173–175 °C; *R_f_* (DCM/MeOH (10%)) = 0.45; ^1^H NMR (CDCl_3_, CD_3_OD, 600 MHz) *δ*/ppm: 7.61 (s, 1H), 7.47 (dd, *J* = 7.6, 1.5 Hz, 1H), 7.38 (d, *J* = 16.2 Hz, 1H), 7.30 (d, *J* = 16.2 Hz, 1H), 7.24 (t, *J* = 3.7 Hz, 1H), 7.13–7.10 (m, 1H), 7.04 (d, *J* = 4.4 Hz, 1H), 6.88 (t, *J* = 7.5 Hz, 1H), 6.81 (d, *J* = 8.1 Hz, 1H); ^13^C NMR (CDCl_3_, 600 MHz) *δ*/ppm: 158.6, 152.9, 144.8, 135.9, 133.1, 132.8, 130.8, 129.3, 128.5, 127.8, 125.5, 123.8, 119.6.
MS (ESI) *m*/*z* (%, fragment): 242 (100); 200 (30); HRMS (*m*/*z*) for C_13_H_9_NO_2_S: [M + H]^+^_calcd_ = 243.0354, and [M + H]^+^_measured_ = 243.0355 (for the mixture of isomers).
2-((*Z*)-2-(5-((*E*)-(hydroxyimino)methyl)thiophen-2-yl)vinyl)benzonitrile (*cis,syn*-**5**): 38 mg, 33% isolated yield; white powder; m.p. = 171–173 °C; *R_f_* (DCM/MeOH (5%)) = 0.65; ^1^H NMR (CDCl_3_, 600 MHz) *δ*/ppm: 8.12 (s, 1H), 7.72 (d, *J* = 7.9 Hz, 1H), 7.60–7.55 (m, 2H), 7.43 (t, *J* = 7.1 Hz, 1H), 7.09 (s, 1H), 6.99 (d, *J* = 4.1 Hz, 1H), 6.91 (d, *J* = 4.1 Hz, 1H), 6.88 (d, *J* = 12.1 Hz, 1H), 6.69 (d, *J* = 12.1 Hz, 1H); MS (ESI) *m*/*z* (%, fragment): 255 (100); HRMS (*m*/*z*) for C_14_H_10_N_2_OS: [M + H]^+^_calcd_ = 254.0513, and [M + H]^+^_measured_ = 254.0514.

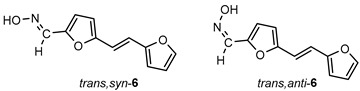

(*E*)-5-((*E*)-2-(furan-2-yl)vinyl)furan-2-carbaldehyde oxime (*trans,syn*-**6**): 15 mg, 23%; *R_f_* (DCM/MeOH (10%)) = 0.56; ^1^H NMR (CDCl_3_, 600 MHz) *δ*/ppm: 7.97 (s, 1H), 7.45 (s, 1H), 6.97 (d, *J* = 16.2 Hz, 1H), 6.77 (d, *J* = 16.2 Hz, 1H), 6.63 (d, *J* = 3.6 Hz, 1H), 6.42–6.41 (m, 1H), 6.38 (d, *J* = 3.7 Hz, 1H), 6.37 (d, *J* = 3.2 Hz, 1H).
(*Z*)-5-((*E*)-2-(furan-2-yl)vinyl)furan-2-carbaldehyde oxime (*trans,anti*-**6**): 34 mg, 48% isolated yield; white powder; m.p. = 150–153 °C; *R_f_* (DCM/MeOH (10%)) = 0.53; ^1^H NMR (CDCl_3_, 600 MHz) *δ*/ppm: 7.51 (s, 1H), 7.41 (d, *J* = 1.8 Hz, 1H), 7.31 (d, *J* = 3.7 Hz, 1H), 6.93 (d, *J* = 16.2 Hz, 1H), 6.79 (d, *J* = 16.2 Hz, 1H), 6.46 (d, *J* = 3.6 Hz, 1H), 6.44–6.43 (m, 1H), 6.40 (d, *J* = 3.4 Hz, 1H).
MS (ESI) *m*/*z* (%, fragment): 204 (100); HRMS (*m*/*z*) for C_11_H_10_NO_3_: [M + H]^+^_calcd_ = 203.0582, and [M + H]^+^_measured_ = 203.0586.

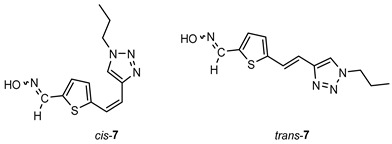

(*E*)-5-((*Z*)-2-(1-propyl-1*H*-1,2,3-triazol-4-yl)vinyl)thiophene-2-carbaldehyde oxime (*cis,syn*-**7**): 17 mg; 9%; *R_f_* (DCM/MeOH (5%)) = 0.22; ^1^H NMR (CDCl_3_, 600 MHz) *δ*/ppm: 8.21 (s, 1H), 7.53 (s, 1H), 7.08–7.06 (m, 2H), 6.69 (d, *J* = 12.4 Hz, 1H), 6.63 (d, *J* = 12.4 Hz, 1H), 4.35–4.29 (m, 2H), 1.98–1.91 (m, 2H), 0.99–0.95 (m, 3H).
(*Z*)-5-((*Z*)-2-(1-propyl-1*H*-1,2,3-triazol-4-yl)vinyl)thiophene-2-carbaldehyde oxime (*cis,anti*-**7**): 20 mg; 11%; *R_f_* (DCM/MeOH (5%)) = 0.22; ^1^H NMR (CDCl_3_, 600 MHz) *δ*/ppm: 7.64 (s, 1H), 7.63 (s, 1H), 7.10 (d, *J* = 3.2 Hz, 1H), 7.08–7.06 (m, 1H), 6.65 (d, *J* = 12.1 Hz, 1H), 6.58 (d, *J* = 12.1 Hz, 1H), 4.35–4.29 (m, 2H), 1.98–1.91 (m, 2H), 0.99–0.95 (m, 3H).
(*E*)-5-((*E*)-2-(1-propyl-1*H*-1,2,3-triazol-4-yl)vinyl)thiophene-2-carbaldehyde oxime (*trans,syn*-**7**): 22 mg; 12%; *R_f_* (DCM/MeOH (5%)) = 0.21; ^1^H NMR (CDCl_3_, 600 MHz) *δ*/ppm: 8.22 (s, 1H), 7.53 (s, 1H), 7.47 (d, *J* = 15.6 Hz, 1H), 7.31 (d, *J* = 3.8 Hz, 1H), 7.29 (d, *J* = 3.8 Hz, 1H), 7.01 (d, *J* = 15.6 Hz, 1H), 4.35–4.29 (m, 2H), 1.98–1.91 (m, 2H), 0.99–0.95 (m, 3H).
(*Z*)-5-((*E*)-2-(1-propyl-1*H*-1,2,3-triazol-4-yl)vinyl)thiophene-2-carbaldehyde oxime (*trans,anti*-**7**): 19 mg; 10%; *R_f_* (DCM/MeOH (5%)) = 0.21; ^1^H NMR (CDCl_3_, 600 MHz) *δ*/ppm: 7.65 (s, 1H), 7.61 (s, 1H), 7.42 (d, *J* = 15.4 Hz, 1H), 7.08 (d, *J* = 3.7 Hz, 1H), 7.08 (d, *J* = 4.2 Hz, 1H), 6.92 (d, *J* = 15.4 Hz, 1H), 4.35–4.29 (m, 2H), 1.98–1.91 (m, 2H), 0.99–0.95 (m, 3H).
  MS (ESI) *m*/*z* (%, fragment): 263 (100); HRMS (*m*/*z*) for C_12_H_15_N_4_OS: [M + H]^+^_calcd_ = 262.0888, and [M + H]^+^_measured_ = 262.0890 (for the mixture of isomers).

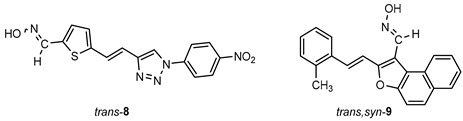

5-(2-(1-(4-nitrophenyl)-1*H*-1,2,3-triazol-4-yl)vinyl)thiophene-2-carbaldehyde oxime (*trans*-**8**): 6 mg, 2%; *R_f_* (DCM/MeOH (5%)) = 0.12; ^1^H NMR (CDCl_3_, 600 MHz) *δ*/ppm: 8.82 (s, 1H), 8.79 (s, 1H), 8.48 (d, *J* = 9.8 Hz, 2H), 8.19 (d, *J* = 9.6 Hz, 2H), 7.67 (d, *J* = 16.6 Hz, 1H), 7.65 (d, *J* = 4.7 Hz, 1H), 7.63 (d, *J* = 5.1 Hz, 1H), 7.61 (d, *J* = 16.6 Hz, 1H); MS (ESI) *m*/*z* (%, fragment): 342 (30); 121 (100); HRMS (*m*/*z*) for C_15_H_11_N_5_O_3_S: [M + H]^+^_calcd_ = 341.0583, and [M + H]^+^_measured_ = 341.0574 (for the mixture of isomers).
(*E*)-2-((*E*)-2-methylstyryl)naphtho[2,1-*b*]furan-1-carbaldehyde oxime (*trans,syn*-**9**): 38 mg, 40% isolated yield; yellow powder; m.p. = 148–152 °C; *R_f_* (DCM) = 0.35; ^1^H NMR (CDCl_3_, 600 MHz) *δ*/ppm: 7.60 (s, 1H), 7.50–7.45 (m, 1H), 7.40 (d, *J* = 16.6 Hz, 1H), 7.30 (d, *J* = 16.6 Hz, 1H) 7.25 (d, *J* = 3.7 Hz, 1H), 7.11 (t, *J* = 7.9 Hz, 1H), 7.04 (d, *J* = 3.7 Hz, 1H), 6.87 (t, *J* = 7.9 Hz, 1H), 6.83 (d, *J* = 8.5 Hz, 1H), 3.22 (s, 3H); MS (ESI) *m*/*z* (%, fragment): 328 (100); 121 (30); HRMS (*m*/*z*) for C_22_H_17_NO_2_: [M + H]^+^_calcd_ = 327.1259, and [M + H]^+^_measured_ = 327.1254 (for the mixture of isomers).

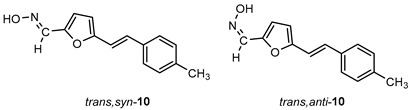

(*E*)-5-((*E*)-4-methylstyryl)furan-2-carbaldehyde oxime (*trans,syn*-**10**): 203 mg, 28% isolated yield; white powder; m.p. = 153–154 °C; *R_f_* (DCM/MeOH (1%)) = 0.35; ^1^H NMR (CDCl_3_, 600 MHz) *δ*/ppm: 7.99 (s, 1H), 7.59 (s, 1H), 7.37 (d, *J* = 8.2 Hz, 2H), 7.17–7.15 (m, 3H), 6.83 (d, *J* = 16.3 Hz, 1H), 6.68 (d, *J* = 3.5 Hz, 1H), 6.38 (d, *J* = 3.5 Hz, 1H), 2.35 (s, 3H); ^13^C NMR (CDCl_3_, 600 MHz) *δ*/ppm: 155.3, 146.2, 140.2, 138.1, 133.8, 129.5, 129.5, 126.5, 115.4, 114.7, 109.9, 21.3; MS (ESI) *m*/*z* (%, fragment): 228 (100); HRMS (*m*/*z*) for C_14_H_13_NO_2_: [M + H]^+^_calcd_ = 227.0946, and [M + H]^+^_measured_ = 227.0947.
(*Z*)-5-((*E*)-4-methylstyryl)furan-2-carbaldehyde oxime (*trans,anti*-**10**): 168 mg, 23% isolated yield; yellow oil; *R_f_* (DCM/MeOH (1%)) = 0.26; ^1^H NMR (CDCl_3_, 600 MHz) *δ*/ppm: 7.53 (s, 1H), 7.38 (d, *J* = 7.1 Hz, 2H), 7.31 (d, *J* = 3.1 Hz, 1H), 7.16 (d, *J* = 7.7 Hz, 2H), 7.15 (d, *J* = 16.5 Hz, 1H), 6.84 (d, *J* = 16.5 Hz, 1H), 6.46 (d, *J* = 3.3 Hz, 1H), 2.36 (s, 3H); ^13^C NMR (CDCl_3_, 600 MHz) *δ*/ppm: 153.9, 144.0, 138.2, 137.4, 133.7, 129.7, 126.5, 120.3, 114.9, 110.8, 21.3.

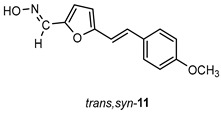

(*E*)-5-((*E*)-4-methoxystyryl)furan-2-carbaldehyde oxime (*trans,syn*-**11**): 7 mg, 6% isolated yield; yellow powder; m.p. = 160–162 °C; *R_f_* (DCM/E (1%)) = 0.26; ^1^H NMR (CDCl_3_, 600 MHz) *δ*/ppm: 8.20 (s, 1H), 7.861 (s, 1H), 7.43–7.41 (m, 3H), 7.03 (d, *J* = 15.8 Hz, 1H), 6.91–6.84 (m, 3H), 6.71 (d, *J* = 1.2 Hz, 1H), 3.82 (s, 3H); ^13^C NMR (CDCl_3_, 600 MHz) *δ*/ppm: 159.1, 143.9, 142.4, 139.2, 130.3, 129.1, 127.3, 126.2, 114.4, 113.7, 108.4, 54.8.

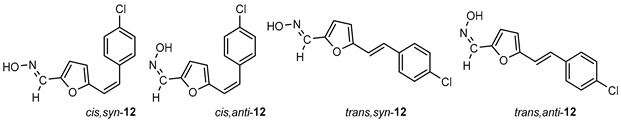

(*E*)-5-((*Z*)-4-chlorostyryl)furan-2-carbaldehyde oxime (*cis,syn*-**12**): 39 mg, 42% isolated yield; white powder; m.p. = 178–180 °C; *R_f_* (DCM/MeOH (1%)) = 0.73; ^1^H NMR (CDCl_3_, 600 MHz) *δ*/ppm: 8.0 (s, 1H), 7.91 (s, 1H), 7.40 (d, *J* = 7.6 Hz, 2H), 7.31 (d, *J* = 7.6 Hz, 2H), 6.56 (d, *J* = 3.4 Hz, 1H), 6.50 (d, *J* = 12.6 Hz, 1H), 6.38 (d, *J* = 12.6 Hz, 1H), 6.28 (d, *J* = 3.5 Hz, 1H); ^13^C NMR (CDCl_3_, 600 MHz) *δ*/ppm: 153.4, 146.0, 140.1, 135.3, 133.5, 130.0, 128.9, 128.5, 118.0, 114.2, 111.9; MS (ESI) *m*/*z* (%, fragment): 248 (100); HRMS (*m*/*z*) for C_13_H_10_ClNO_2_: [M + H]^+^_calcd_ = 247.0400, and [M + H]^+^_measured_ = 247.0401.
(*Z*)-5-((*Z*)-4-chlorostyryl)furan-2-carbaldehyde oxime (*cis,anti*-**12**): 15 mg, 14%; *R_f_* (DCM/MeOH (1%)) = 0.50; ^1^H NMR (CDCl_3_, 600 MHz) *δ*/ppm: 8.63 (s, 1H), 7.39 (s, 1H), 7.38–7.28 (m, 4H), 7.23 (d, *J* = 3.2 Hz, 1H), 6.54 (d, *J* = 12.8 Hz, 1H), 6.38 (d, *J* = 3.4 Hz, 1H), 6.37 (d, *J* = 12.8 Hz, 1H); ^13^C NMR (CDCl_3_, 600 MHz) *δ*/ppm: 152.2, 143.8, 137.0, 135.3, 133.5, 130.1 129.3, 128.3, 119.7, 117.8, 113.0.
(*E*)-5-((*E*)-4-chlorostyryl)furan-2-carbaldehyde oxime (*trans,syn*-**12**): 19 mg, 20%; *R_f_* (DCM/MeOH (1%)) = 0.44; ^1^H NMR (CDCl_3_, 600 MHz) *δ*/ppm: 7.98 (s, 1H), 7.49 (s, 1H), 7.39 (d, *J* = 8.4 Hz, 2H), 7.31 (d, *J* = 8.5 Hz, 2H), 7.13 (d, *J* = 16.3 Hz, 1H), 6.84 (d, *J* = 16.2 Hz, 1H), 6.65 (d, *J* = 3.5 Hz, 1H), 6.42 (d, *J* = 3.5 Hz, 1H); ^13^C NMR (CDCl_3_, 600 MHz) *δ*/ppm: 154.7, 146.6, 140.2, 135.1, 133.7, 128.9, 128.0, 127.6, 116.1, 115.3, 110.7.
(*Z*)-5-((*E*)-4-chlorostyryl)furan-2-carbaldehyde oxime (*trans,anti*-**12**): 5 mg, 5%; *R_f_* (DCM/MeOH (1%)) = 0.34; ^1^H NMR (CDCl_3_, 600 MHz) *δ*/ppm: 8.10 (s, 1H), 7.99 (s, 1H), 7.38 (d, *J* = 8.4 Hz, 2H), 7.30 (d, *J* = 8.9 Hz, 2H), 7.14 (d, *J* = 16.1 Hz, 1H), 6.83 (d, *J* = 16.1 Hz, 1H), 6.65 (d, *J* = 3.1 Hz, 1H), 6.42 (d, *J* = 3.4 Hz, 1H).

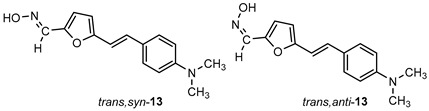

(*E*)-5-((*E*)-4-(dimethylamino)styryl)furan-2-carbaldehyde oxime (*trans,syn*-**13**): 10 mg, 5%; *R_f_* (DCM/MeOH (5%)) = 0.65; ^1^H NMR (CDCl_3_, 600 MHz) *δ*/ppm: 7.96 (s, 1H), 7.38 (d, *J* = 8.8 Hz, 2H), 7.37 (d, *J* = 8.8 Hz, 2H), 7.09 (d, *J* = 16.5 Hz, 1H), 6.60 (d, *J* = 16.5 Hz, 1H), 6.62 (d, *J* = 3.5 Hz, 1H), 6.31 (d, *J* = 3.5 Hz, 1H), 2.99 (s, 6H).
(*Z*)-5-((*E*)-4-(dimethylamino)styryl)furan-2-carbaldehyde oxime (*trans,anti*-**13**): 11 mg, 7%; *R_f_* (DCM/MeOH (5%)) = 0.59; ^1^H NMR (CDCl_3_, 600 MHz) *δ*/ppm: 7.51 (s, 1H), 7.38 (d, *J* = 8.8 Hz, 2H), 7.37 (d, *J* = 8.7 Hz, 2H), 7.31 (d, *J* = 3.7 Hz, 1H), 7.13 (d, *J* = 16.4 Hz, 1H), 7.09 (d, *J* = 16.4 Hz, 1H), 6.39 (d, *J* = 3.6 Hz, 1H), 2.99 (s, 6H).
Data for the isomers written from the ^1^H NMR of the mixture of isomers. MS (ESI) *m*/*z* (%, fragment): 239 (100); HRMS (*m*/*z*) for C_14_H_10_N_2_O_2_: [M + H]^+^_calcd_ = 238.0742, and [M + H]^+^_measured_ = 238.0741.

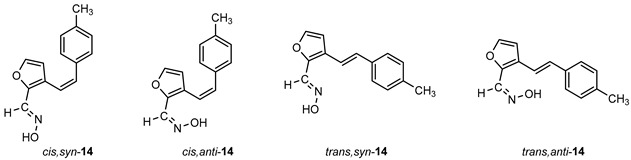

(*E*)-3-((*Z*)-4-methylstyryl)furan-2-carbaldehyde oxime (*cis,syn*-**14**): 113 mg, 38% isolated yield; white powder; m.p. = 145–148 °C; *R_f_* (PE/E (15%)) = 0.27; ^1^H NMR (CDCl_3_, 600 MHz) *δ*/ppm: 9.08 (s, 1H), 8.11 (s, 1H), 7.27 (d, *J* = 1.6 Hz, 1H), 7.19 (d, *J* = 8.0, 2H), 7.09 (d, *J* = 8.0, 2H), 6.65 (d, *J* = 12.2 Hz, 1H), 6.44 (d, *J* = 12.2 Hz, 1H), 6.17 (d, *J* = 1.6 Hz, 1H), 2.33 (s, 3H); ^13^C NMR (CDCl_3_, 150 MHz) *δ*/ppm: 144.1, 143.6, 139.4, 137.5, 134.0, 132.4, 129.0, 128.8, 124.3, 117.9, 111.9, 21.3. MS (ESI) *m*/*z* (%, fragment): 228 (100); HRMS (*m*/*z*) for C_14_H_13_NO_2_: [M + H]^+^_calcd_ = 227.0952, and [M + H]^+^_measured_ = 227.0946.
(*Z*)-3-((*Z*)-4-methylstyryl)furan-2-carbaldehyde oxime (*cis,anti*-**14**): 2 mg, 1%; *R_f_* (PE/E (15%)) = 0.22; ^1^H NMR (CDCl_3_, 600 MHz) *δ*/ppm: 7.43 (d, *J* = 1.7 Hz, 1H), 7.26 (s, 1H), 7.18 (d, *J* = 7.9 Hz, 2H), 7.09 (d, *J* = 7.9 Hz, 2H), 6.70 (d, *J* = 11.8 Hz, 1H), 6.45 (d, *J* = 11.8 Hz, 1H), 6.22 (d, *J* = 1.7 Hz, 1H), 2.34 (s, 3H).
(*E*)-3-((*E*)-4-methylstyryl)furan-2-carbaldehyde oxime (*trans,syn*-**14**): 70 mg, 34% isolated yield; white powder; m.p. = 146–147 °C; *R_f_* (PE/E (10%)) = 0.17; ^1^H NMR (CDCl_3_, 600 MHz) *δ*/ppm: 8.23 (s, 1H), 7.45 (s, 1H), 7.43 (d, *J* = 1.9 Hz, 1H), 7.38 (d, *J* = 8.0 Hz, 2H), 7.17 (d, *J* = 8.0 Hz, 2H), 7.13 (d, *J* = 16.2 Hz, 1H), 6.89 (d, *J* = 16.2 Hz, 1H), 6.72 (d, *J* = 1.9 Hz, 1H), 2.36 (s, 3H); ^13^C NMR (CDCl_3_, 150 MHz) *δ*/ppm: 144.4, 143.2, 140.1, 138.0, 134.1, 131.2, 129.5, 126.5, 126.4, 116.1, 109.0, 21.3.
(*Z*)-3-((*E*)-4-methylstyryl)furan-2-carbaldehyde oxime (*trans,anti*-**14**): 5 mg, 2%; *R_f_* (PE/E (10%)) = 0.10; ^1^H NMR (CDCl_3_, 600 MHz) *δ*/ppm: 7.49 (d, *J* = 1.7 Hz, 1H), 7.44 (s, 1H), 7.32 (d, *J* = 8.0 Hz, 2H), 7.19 (s, 1H), 7.10 (d, *J* = 8.0 Hz, 2H), 7.05 (d, *J* = 16.1 Hz, 1H), 6.84 (d, *J* = 16.1 Hz, 1H), 6.67 (d, *J* = 1.7 Hz, 1H), 2.29 (s, 3H).

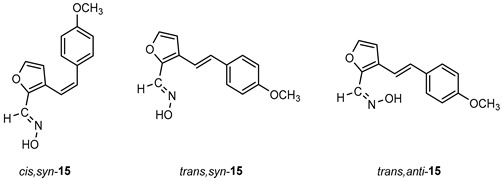

(*E*)-3-((*Z*)-4-methoxystyryl)furan-2-carbaldehyde oxime (*cis,syn*-**15**): 26 mg, 15%; *R_f_* (PE/DCM (60%)) = 0.25; ^1^H NMR (CDCl_3_, 600 MHz) *δ*/ppm: 8.10 (s, 1H), 7.55 (s, 1H), 7.30 (d, *J* = 1.8 Hz, 1H), 7.24 (d, *J* = 8.5 Hz, 2H), 6.83 (d, *J* = 9.2 Hz, 2H), 6.63 (d, *J* = 12.3 Hz, 1H), 6.42 (d, *J* = 12.3 Hz, 1H), 6.21 (d, *J* = 1.9 Hz, 1H), 3.81 (s, 3H); ^13^C NMR (CDCl_3_, 150 MHz) *δ*/ppm: 159.1, 143.9, 143.6, 140.1, 132.0, 130.2, 129.4, 124.8, 117.2, 113.7, 111.9, 55.2.
(*E*)-3-((*E*)-4-methoxystyryl)furan-2-carbaldehyde oxime (*trans,syn*-**15**): 36 mg, 15%; *R_f_* (PE/DCM (60%)) = 0.15; ^1^H NMR (CDCl_3_, 600 MHz) *δ*/ppm: 8.23 (s, 1H), 7.84 (s, 1H), 7.42 (d, *J* = 8.6 Hz, 3H), 7.03 (d, *J* = 16.6 Hz, 1H), 6.89 (d, *J* = 8.6 Hz, 2H), 6.87 (d, *J* = 16.6 Hz, 1H), 6.71 (d, *J* = 2.1 Hz, 1H), 3.83 (s, 3H); ^13^C NMR (CDCl_3_, 150 MHz) *δ*/ppm: 159.6, 144.4, 142.9, 139.9, 130.8, 129.7, 127.8, 126.7, 114.9, 114.2, 108.9, 55.3; MS (ESI) *m*/*z* (%, fragment): 244 (100); HRMS (*m*/*z*) for C_14_H_13_NO_3_: [M + H]^+^_calcd_ = 243.0895, and [M + H]^+^_measured_ = 243.0901.
(*Z*)-3-((*E*)-4-methoxystyryl)furan-2-carbaldehyde oxime (*trans,anti*-**15**): 70 mg, 26% isolated yield; white powder; m.p. = 158–164 °C; *R_f_* (PE/DCM (60%)) = 0.13; ^1^H NMR (CDCl_3_, 600 MHz) *δ*/ppm: 8.23 (s, 1H), 7.93 (s, 1H), 7.42 (d, *J* = 9.1 Hz, 3H), 7.03 (d, *J* = 15.9 Hz, 1H), 6.89 (d, *J* = 9.1 Hz, 2H), 6.87 (d, *J* = 15.9 Hz, 1H), 6.71 (d, *J* = 1.9 Hz, 1H), 3.83 (s, 3H); ^13^C NMR (CDCl_3_, 150 MHz) *δ*/ppm: 159.6, 144.4, 142.9, 139.9, 130.8, 129.7, 127.8, 126.7, 114.9, 114.2, 108.9, 55.3.

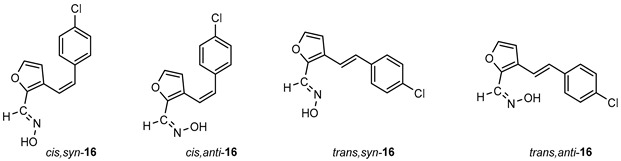

(*E*)-3-((*Z*)-4-chlorostyryl)furan-2-carbaldehyde oxime (*cis,syn*-**16**): 114 mg, 24% isolated yield; white powder; m.p. = 189–191 °C; *R_f_* (PE/E (15%)) = 0.47; ^1^H NMR (CDCl_3_, 600 MHz) *δ*/ppm: 8.10 (s, 1H), 7.99 (s, 1H), 7.28 (d, *J* = 8.5 Hz, 2H), 7.23 (d, *J* = 8.5 Hz, 2H), 6.63 (d, *J* = 12.0 Hz, 1H), 6.55 (d, *J* = 12.0 Hz, 1H), 6.12 (d, *J* = 1.2 Hz, 1H); ^13^C NMR (CDCl_3_, 150 MHz) *δ*/ppm: 144.4, 143.7, 139.8, 135.5, 133.4, 130.9, 130.2, 128.5, 124.0, 119.4, 111.7; MS (ESI) *m*/*z* (%, fragment): 248 (100); HRMS (*m*/*z*) for C_13_H_10_ClNO_2_: [M + H]^+^_calcd_ = 247.0400, and [M + H]^+^_measured_ = 247.0405.
(*Z*)-3-((*Z*)-4-chlorostyryl)furan-2-carbaldehyde oxime (*cis,anti*-**16**): 5 mg, 1%; *R_f_* (PE/E (15%)) = 0.28; ^1^H NMR (CDCl_3_, 600 MHz) *δ*/ppm: 7.44 (d, *J* = 1.6 Hz, 1H), 7.35 (s, 1H), 7.26 (d, *J* = 8.6 Hz, 2H), 7.21 (d, *J* = 8.6 Hz, 2H), 6.66 (d, *J* = 16.2 Hz, 2H), 6.53 (d, *J* = 16.2 Hz, 2H), 6.16 (d, *J* = 1.8 Hz, 1H).
(*E*)-3-((*E*)-4-chlorostyryl)furan-2-carbaldehyde oxime (*trans,syn*-**16**): 48 mg, 20% isolated yield; white powder; m.p. = 181–183 °C; *R_f_* (PE/E (15%)) = 0.40; ^1^H NMR (CDCl_3_, 600 MHz) *δ*/ppm: 8.22 (s, 1H), 7.48 (s, 1H), 7.48 (s, 1H), 7.44 (d, *J* = 1.6 Hz, 1H), 7.41 (d, *J* = 8.5 Hz, 2H), 7.32 (d, *J* = 8.5 Hz, 1H), 7.19 (d, *J* = 16.1 Hz, 1H), 6.86 (d, *J* = 16.1 Hz, 1H), 6.72 (d, *J* = 1.7 Hz, 1H); ^13^C NMR (CDCl_3_, 150 MHz) *δ*/ppm: 144.4, 143.7, 140.2, 135.4, 133.5, 129.8, 128.9, 127.6, 125.8, 118.0, 108.9.
(*Z*)-3-((*E*)-4-chlorostyryl)furan-2-carbaldehyde oxime (*trans,anti*-**16**): 12 mg, 3%; *R_f_* (PE/E (15%)) = 0.25; ^1^H NMR (CDCl_3_, 600 MHz) *δ*/ppm: 7.61 (d, *J* = 1.6 Hz, 1H), 7.50 (s, 1H), 7.42 (d, *J* = 8.5 Hz, 2H), 7.33 (d, *J* = 8.5 Hz, 2H), 7.17 (d, *J* = 16.1 Hz, 1H), 6.88 (d, *J* = 16.1 Hz, 1H), 6.74 (d, *J* = 1.6 Hz, 1H); ^13^C NMR (CDCl_3_, 150 MHz) *δ*/ppm: 144.9, 144.5, 140.1, 135.4, 134.3, 130.3, 129.8, 129.0, 127.9, 117.9, 108.7.

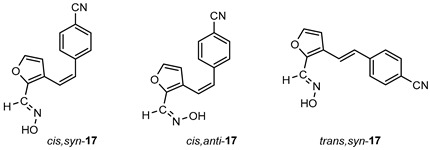

4-((*Z*)-2-(2-((*E*)-(hydroxyimino)methyl)furan-3-yl)vinyl)benzonitrile (*cis,syn*-**17**): 141 mg, 43% isolated yield; white powder; m.p. = 198–201 °C; *R_f_* (PE/E (20%)) = 0.56; ^1^H NMR (CDCl_3_, 600 MHz) *δ*/ppm: 8.10 (s, 1H), 7.59 (d, *J* = 8.3 Hz, 2H), 7.41 (d, *J* = 8.3 Hz, 2H), 7.32 (d, *J* = 1.9 Hz, 1H), 6.71 (d, *J* =12.1 Hz, 1H), 6.64 (d, *J* =12.1 Hz, 1H), 6.07 (d, *J* = 1.9 Hz, 1H); ^13^C NMR (CDCl_3_, 150 MHz) *δ*/ppm: 143.9, 140.2, 132.5, 132.2, 130.0, 129.9, 129.5, 126.8, 123.1, 121.7, 117.7, 111.6; MS (ESI) *m*/*z* (%, fragment): 239 (100); HRMS (*m*/*z*) for C_14_H_10_N_2_O_2_: [M + H]^+^_calcd_ = 247.0400, and [M + H]^+^_measured_ = 247.0405.
4-((*Z*)-2-(2-((*Z*)-(hydroxyimino)methyl)furan-3-yl)vinyl)benzonitrile (*cis,anti*-**17**): 17 mg, 6%; *R_f_* (PE/E (20%)) = 0.34; ^1^H NMR (CDCl_3_, 600 MHz) *δ*/ppm: 7.58 (d, *J* = 8.3 Hz, 2H), 7.45 (d, *J* = 1.9 Hz, 1H), 7.39 (d, *J* = 8.3 Hz, 2H), 7.33 (s, 1H), 6.69 (d, *J* = 12.3 Hz, 1H), 6.67 (d, *J* = 12.3 Hz, 1H), 6.11 (d, *J* = 1.9 Hz, 1H); ^13^C NMR (CDCl_3_, 150 MHz) *δ*/ppm: 144.5, 141.4, 134.2, 132.1, 130.7, 129.5, 125.5, 121.9, 118.7, 111.5, 111.1, 103.0.
4-((*E*)-2-(2-((E)-(hydroxyimino)methyl)furan-3-yl)vinyl)benzonitrile (*trans,syn*-**17**): 47 mg, 16% isolated yield; white powder; m.p. = 193–195 °C; *R_f_* (PE/E (30%)) = 0.53; ^1^H NMR (CDCl_3_, 600 MHz) *δ*/ppm: 8.23 (s, 1H), 7.76 (s, 1H), 7.63 (d, *J* = 8.2 Hz, 2H), 7.56 (d, *J* = 8.2 Hz, 2H), 7.47 (d, *J* = 1.8 Hz, 1H), 7.37 (d, *J* = 16.3 Hz, 1H), 6.90 (d, *J* = 16.3 Hz, 1H), 6.74 (d, *J* = 1.8 Hz, 1H); ^13^C NMR (CDCl_3_, 150 MHz) *δ*/ppm: 144.6, 144.5, 141.4, 140.3, 132.6, 132.5, 126.9, 125.0, 121.2, 118.9, 110.8, 108.9.

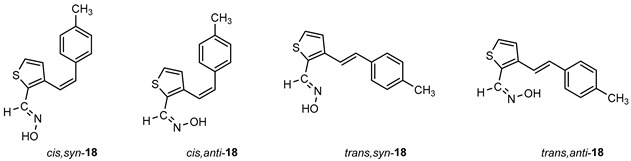

(*E*)-3-((*Z*)-4-methylstyryl)thiophene-2-carbaldehyde oxime (*cis,syn*-**18**): 72 mg, 28% isolated yield; white powder; m.p. = 158–160 °C; R*_f_* (PE/E (10%)) = 0.53; ^1^H NMR (CDCl_3_, 300 MHz) *δ*/ppm: 8.32 (s, 1H), 7.15 (d, *J* = 5.2 Hz, 2H), 7.09 (d, *J* = 8.2 Hz, 2H), 7.04 (d, *J* = 8.2 Hz, 2H), 6.79 (d, *J* = 5.3 Hz, 1H), 6.68 (d, *J* = 12.1 Hz, 1H), 6.50 (d, *J* = 12.1 Hz, 1H), 2.31 (s, 3H); ^13^C NMR (CDCl_3_, 150 MHz) *δ*/ppm: 144.5, 140.0, 137.6, 132.8, 130.4, 129.0, 128.9, 128.8, 126.4, 121.6, 121.1, 21.2; MS (ESI) *m*/*z* (%, fragment): 244 (100); HRMS (*m*/*z*) for C_14_H_13_NOS: [M + H]^+^_calcd_ = 243.0718, and [M + H]^+^_measured_ = 243.0717.
(*Z*)-3-((*Z*)-4-methylstyryl)thiophene-2-carbaldehyde oxime (*cis,anti*-**18**): 58 mg, 23% isolated yield; white powder; m.p. = 155–156 °C; *R_f_* (PE/E (10%)) = 0.34; ^1^H NMR (CDCl_3_, 300 MHz) *δ*/ppm: 7.81 (s, 1H), 7.42 (d, *J* = 5.1 Hz, 1H), 7.05 (d, *J* = 8.7 Hz, 2H), 7.01 (d, *J* = 8.7 Hz, 2H), 6.87 (d, *J* = 5.2 Hz, 1H), 6.73 (d, *J* = 12.1 Hz, 1H), 6.58 (d, *J* = 12.1 Hz, 1H), 2.29 (s, 3H); ^13^C NMR (CDCl_3_, 150 MHz) *δ*/ppm: 141.5, 140.1, 137.6, 133.5, 133.5, 130.4, 129.0, 128.9, 127.6, 125.8, 121.5, 99.9, 21.2.
  (*E*)-3-((*E*)-4-methylstyryl)thiophene-2-carbaldehyde oxime (*trans*,*syn*-**18**): 87 mg, 34% isolated yield; white powder; m.p. = 151–153 °C; *R_f_* (PE/E (10%)) = 0.40; ^1^H NMR (CDCl_3_, 300 MHz) *δ*/ppm: 8.55 (s, 1H), 7.39 (d, *J* = 8.0 Hz, 2H), 7.34 (s, 1H), 7.30 (k, 2H), 7.18 (d, *J* = 6.6 Hz, 3H), 6.98 (d, *J* = 16.1 Hz, 1H), 2.37 (s, 3H); ^13^C NMR (CDCl_3_, 150 MHz) *δ*/ppm: 144.0, 140.6, 138.2, 134.1, 131.4, 129.8, 129.5, 127.2, 126.5, 125.6, 118.9, 21.3.
(*Z*)-3-((*E*)-4-methylstyryl)thiophene-2-carbaldehyde oxime (*trans*,*anti*-**18**): 43 mg, 13%; *R_f_* (PE/E (10%)) = 0.23; ^1^H NMR (CDCl_3_, 300 MHz) *δ*/ppm: 8.02 (s, 1H), 7.52 (d, *J* = 5.4 Hz, 1H), 7.42 (d, *J* = 8.0 Hz, 2H), 7.37 (d, *J* = 5.4 Hz, 1H), 7.29 (d, *J* = 8.7 Hz, 1H), 7.19 (d, *J* = 8.1 Hz, 2H), 7.04 (d, *J* = 16.1 Hz, 1H), 2.37 (s, 3H).

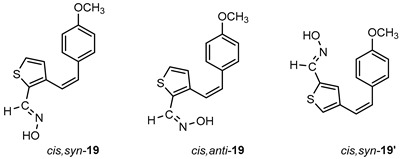

(*E*)-3-((*Z*)-4-methoxystyryl)thiophene-2-carbaldehyde oxime (*cis,syn*-**19**): 7 mg, 6%; *R_f_* (DCM) = 0.55; ^1^H NMR (CDCl_3_, 600 MHz) *δ*/ppm: 8.32 (s, 1H), 7.69 (s, 1H), 7.16 (d, *J* = 4.3 Hz, 1H), 7.13 (d, *J* = 8.6 Hz, 2H), 6.81 (d, *J* = 5.1 Hz, 1H), 6.80 (d, *J* = 8.8 Hz, 2H), 6.64 (d, *J* = 11.8 Hz, 1H), 6.45 (d, *J* = 11.8 Hz, 1H), 3.78 (s, 3H).
(*Z*)-3-((*Z*)-4-methoxystyryl)thiophene-2-carbaldehyde oxime (*cis,anti*-**19**): 16 mg, 13%; *R_f_* (DCM) = 0.45; ^1^H NMR (CDCl_3_, 600 MHz) *δ*/ppm: 7.81 (s, 1H), 7.44 (d, *J* = 5.2 Hz, 1H), 7.08 (d, *J* = 8.7 Hz, 2H), 6.89 (d, *J* = 5.2 Hz, 1H), 6.74 (d, *J* = 8.7 Hz, 2H), 6.69 (d, *J* = 11.9 Hz, 1H), 6.52 (d, *J* = 11.9 Hz, 1H), 3.77 (s, 3H).
(*E*)-4-((*Z*)-4-methoxystyryl)thiophene-2-carbaldehyde oxime (*cis,syn*-**19′**): 100 mg, 78% isolated yield; white powder; m.p. = 159–160 °C; *R_f_* (DCM) = 0.60; ^1^H NMR (CDCl_3_, 600 MHz) *δ*/ppm: 9.26 (s, 1H), 8.11 (s, 1H), 7.30 (d, *J* = 1.7 Hz, 1H), 7.23 (d, *J* = 8.6 Hz, 2H), 6.82 (d, *J* = 9.2 Hz, 2H), 6.61 (d, *J* = 12.0 Hz, 1H), 6.39 (d, *J* = 12.0 Hz, 1H), 6.19 (d, *J* = 1.8 Hz, 1H), 3.79 (s, 3H); ^13^C NMR (CDCl_3_, 75 MHz) *δ*/ppm: 159.2, 144.7, 143.1, 138.7, 131.4, 129.8, 123.7, 117.3, 113.7, 111.4, 54.3.

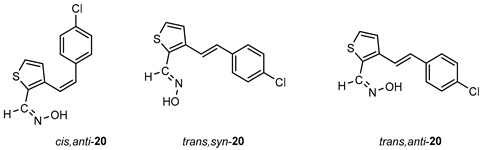

(*Z*)-3-((*Z*)-4-chlorostyryl)thiophene-2-carbaldehyde oxime (*cis,anti*-**20**): 8 mg, 29%; *R_f_* (PE/E (20%)) = 0.35; ^1^H NMR (CDCl_3_, 300 MHz) *δ*/ppm: 8.29 (s, 1H), 7.21 (d, *J* = 8.5 Hz, 2H), 7.17 (d, *J* = 5.3 Hz, 1H), 7.12 (d, *J* = 8.5 Hz, 2H), 6.74 (d, *J* = 5.3 Hz, 1H), 6.65 (d, *J* = 12.1 Hz, 1H), 6.58 (d, *J* = 12.1 Hz, 1H).
(*E*)-3-((*E*)-4-chlorostyryl)thiophene-2-carbaldehyde oxime (*trans,syn*-**20**): 6 mg, 21%; *R_f_* (PE/E (20%)) = 0.62; ^1^H NMR (CDCl_3_, 300 MHz) *δ*/ppm: 8.54 (s, 1H), 7.42 (d, *J* = 8.5 Hz, 2H), 7.33 (d, *J* = 8.6 Hz, 2H), 7.30 (s, 2H) 7.22 (d, *J* = 16.1 Hz, 1H), 6.95 (d, *J* = 16.1 Hz, 1H); ^13^C NMR (CDCl_3_, 150 MHz) *δ*/ppm: 143.8, 139.9, 135.4, 133.7, 130.0, 129.0, 127.7, 127.4, 125.5, 124.3, 120.5.
(*Z*)-3-((*E*)-4-chlorostyryl)thiophene-2-carbaldehyde oxime (*trans,anti*-**20**): 8 mg, 29%; *R_f_* (PE/E (20%)) = 0.27; ^1^H NMR (CDCl_3_, 300 MHz) *δ*/ppm: 7.93 (s, 1H), 7.53 (s, 2H), 7.42–7.38 (m, 2H), 7.35 (d, *J* = 16.1 Hz, 1H), 7.30 (t, *J* = 8.4 Hz, 1H), 7.24–7.17 (m, 2H), 6.96 (d, *J* = 16.1 Hz, 1H).

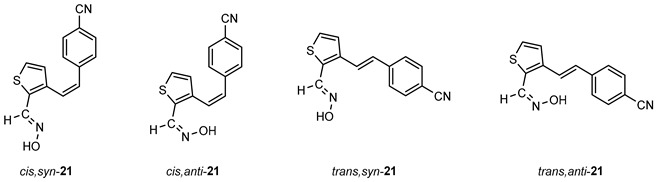

4-((*Z*)-2-(2-((*E*)-(hydroxyimino)methyl)thiophen-3-yl)vinyl)benzonitrile (*cis,syn*-**21**): 62 mg, 34% isolated yield; yellow powder; m.p. = 169–171 °C; *R_f_* (PE/E (25%)) = 0.58; ^1^H NMR (CDCl_3_, 300 MHz) *δ*/ppm: 8.25 (s, 1H), 7.53 (d, *J* = 8.3 Hz, 2H), 7.34 (s, 1H), 7.29 (d, *J* = 8.3 Hz, 2H), 7.20 (d, *J* = 5.2 Hz, 1H), 6.74–6.66 (m, 3H); ^13^C NMR (CDCl_3_, 150 MHz) *δ*/ppm: 138.6, 136.0, 133.0, 126.9, 126.7, 125.5, 124.2, 123.0, 121.9, 119.9, 113.5, 105.8; MS (ESI) *m*/*z* (%, fragment): 255 (100); HRMS (*m*/*z*) for C_14_H_10_N_2_OS: [M + H]^+^_calcd_ = 254.0514, and [M + H]^+^_measured_ = 254.0512.
4-((*Z*)-2-(2-((*Z*)-(hydroxyimino)methyl)thiophen-3-yl)vinyl)benzonitrile (*cis,anti*-**21**): 52 mg, 27% isolated yield; yellow powder; m.p. = 170–171 °C; *R_f_* (PE/E (25%)) = 0.24; ^1^H NMR (CDCl_3_, 300 MHz) *δ*/ppm: 7.75 (s, 1H), 7.50 (d, *J* = 8.3 Hz, 2H), 7.47 (d, *J* = 5.4 Hz, 1H), 7.24 (d, *J* = 8.3 Hz, 2H), 6.83 (d, *J* = 12.2 Hz, 1H), 6.79 (d, *J* = 5.3 Hz, 1H), 6.75 (d, *J* = 12.2 Hz, 1H); ^13^C NMR (CDCl_3_, 150 MHz) *δ*/ppm: 143.8, 141.1, 139.8, 138.3, 132.1, 130.8, 129.5, 128.3, 127.1, 125.7, 118.7, 111.0.
4-((*E*)-2-(2-((*E*)-(hydroxyimino)methyl)thiophen-3-yl)vinyl)benzonitrile (*trans,syn*-**21**): 11 mg, 5%; *R_f_* (PE/E (25%)) = 0.47; ^1^H NMR (CDCl_3_, 300 MHz) *δ*/ppm: 8.53 (s, 1H), 7.65 (d, *J* = 8.3 Hz, 2H), 7.57 (d, *J* = 8.3 Hz, 2H), 7.36–7.35 (m, 4H), 6.98 (d, *J* = 16.1 Hz, 1H); ^13^C NMR (CDCl_3_, 150 MHz) *δ*/ppm: 148.7, 143.6, 141.4, 132.6, 129.2, 127.6, 126.9, 125.5, 123.4, 121.2, 118.9, 111.1.
4-((*E*)-2-(2-((*Z*)-(hydroxyimino)methyl)thiophen-3-yl)vinyl)benzonitrile (*trans,anti*-**21**): 15 mg, 7%; *R_f_* (PE/E (25%)) = 0.22; ^1^H NMR (CDCl_3_, 300 MHz) *δ*/ppm: 8.05 (s, 1H), 7.72 (d, *J* = 8.8 Hz, 2H), 7.69 (d, *J* = 8.8 Hz, 2H), 7.64 (s, 1H), 7.60 (s, 1H), 7.49 (d, *J* = 16.0 Hz, 1H), 7.46 (d, *J* = 5.4 Hz, 1H), 7.14 (d, *J* = 16.0 Hz, 1H).

### 3.2. Oxime ADME Properties and ChE Docking

Physical–chemical properties were calculated using the Calculate molecule properties protocol in BioVia Discovery Studio v21.1 (BioVia, San Diego, CA, USA) and ADMET descriptors calculation to determine the probability of crossing the BBB. Molecular docking of ligands in the active site of AChE and BChE was performed using CDOCKER protocol in BioVia Discovery Studio v21.1 (BioVia, San Diego, CA, USA). The protocol based on a CHARMm force field generated 20 binding poses for each ligand, and scoring functions CDOCKER Energy and CDOCKER Interaction Energy were used to rank the obtained binding poses [63,64]. After an evaluation of poses and comparison to known crystal structures of AChE or BChE inhibitor complexes, we selected the final ones [65]. The crystal structures of human BChE and human AChE deposited in the Protein Data Bank, PDB code 2PM8 [42] and 4PQE [66], respectively, were used. The detailed ligand docking protocol used was described previously [5]. Modeling of the near-attack conformation of oxime reactivators in cyclosarin-inhibited BChE was performed using the structure of phosphorylated *cis,syn*-**5** and *cis,syn*-**12** as described previously [58]. The structure of the cyclosarin-inhibited BChE was generated from the crystal structure of the tabun-inhibited BChE (PDB code 3DJY) [60] using molecular replacement of matching cyclosarin moiety. The final minimized structure of the near-attack conformation of oxime in the cyclosarin-inhibited BChE was submitted to full minimization using a CHARMm force field with an RMS gradient of 1.0 kcal/(mol⋅Å).

Molecular dynamics simulation in BioVia Discovery Studio v21.1 (BioVia, San Diego, CA, USA) was performed using protocol Standard Dynamic Cascade performing full system minimization using Steepest Decent algorithm with RMS gradient 1.0, followed by Adopted Basis NR algorithm with RMS gradient 0.1, heating step for 4 ps T = 310 K, equilibration step 20 ps T = 310 K, and production step for 20 ns at 310 K. For more details about molecular dynamics simulation of near-attack conformation of oximes please see [58].

### 3.3. AChE and BChE Activity Assays

Stock solutions of oximes (100 mM) were dissolved in dimethyl sulfide solvent (DMSO, Kemika, Zagreb, Croatia), stored at 4 °C and diluted just before use. Acetylthiocholine iodide (ATCh), 5,5′-dithiobis(2-nitrobenzoic acid) (DTNB), and bovine serum albumin (BSA) were purchased from Sigma-Aldrich (St. Louis, MO, USA). Stock solutions (5000 μg/mL) of sarin and cyclosarin purchased from NC Laboratory (Spiez, Switzerland) were made in isopropyl alcohol and diluted in water just before use. Recombinant human AChE was a generous gift from Dr. Zoran Radić, Skaggs School of Pharmacy and Pharmaceutical Sciences, University of California at San Diego, La Jolla, USA, while human BChE derived from purified plasma was a generous gift from Dr. Xavier Brazzolotto and Dr. Florian Nachon, Institut de Recherche Biomédicale des Armées, Bretigny-sur-Orge, France. Enzyme activity was measured using the Ellman method [67] at 25 °C. Enzyme inhibition was assessed using various oxime concentrations ranging from 50 to 150 μM for AChE and 5 to 250 μM for BChE in the presence of ATCh (0.02–0.7 mM) and DTNB (0.3 mM) diluted in 0.1 mM sodium phosphate buffer, pH 7.4 in 96-well plates. The tested concentration of DMSO did not significantly inhibit enzymes. The enzyme–oxime dissociation constant of complex (*K*_i_) was derived using the Hunter–Downs equation as previously described [38]. Measurements were conducted on a Tecan Infinite M200PRO microplate reader (Tecan Austria GmbH, Salzburg, Austria) at 25 °C for 5 min.

For reactivation assays, the enzyme was inhibited almost completely (95–100%) during the incubation with a 10-fold excess of the OP for 60 min, and the inhibited enzyme was separated from the excess of unconjugated OP by filtration using ProbeQuant^TM^ G-50 Micro Columns (Cytiva, Buckinghamshire, UK). For assessing the recovery of enzyme activity, the inhibited enzyme was added to a reactivation mixture containing an oxime (100 μM in screenings and 10–200 μM in detailed kinetics). Aliquots of the reactivation mixture were diluted at designated intervals in sodium phosphate buffer containing DTNB (0.3 mM), and enzyme activity was measured upon the addition of ATCh (1 mM) on a CARY 300 spectrophotometer (Varian Inc., Mulgrave, Australia) at 25 °C for 2 min. An equivalent prepared sample of the uninhibited enzyme, whose activity was measured in the presence of an oxime, served as the 100% activity control. Spontaneous reactivation was not observed. If the maximal percentage of reactivation (*React*_max_) was higher than 40%, the first-order reactivation rate constant (*k*_obs_) was determined from the one-phase exponential curve of reactivation% over time. The selected oximes were then evaluated in a wide concentration range (10–200 μM) in order to determine the maximal reactivation rate constant (*k*_2_), dissociation constant (*K*_OX_), and overall second-order reactivation rate constant (*k*_r_) as described previously [68]. Results were analyzed using Prism 9 software (GraphPad by Dotmatics, Boston, MA, USA).

### 3.4. Cytotoxicity Assay

Certified human liver cell culture, HepG2 (ECACC 85011430; European Collection of Authenticated Cell Cultures, Salisbury, England, UK) were cultured as adherent cultures under controlled conditions (5% pCO_2_ and a temperature of 37 °C) in Eagle’s Minimum Essential Medium supplemented with a 1% solution of penicillin-streptomycin (Pen-Strep, Sigma-Aldrich, Steinheim, Germany), 2 mM of glutamine, 10% (*v*/*v*) FBS, and 1% non-essential amino acids. The MTS detection reagent assay (CellTiter 96^®^ AQueous One Solution Cell Proliferation Assay, Promega, Madison, WI, USA) was used to evaluate *in vitro* cytotoxicity. Triton X-100 (Sigma-Aldrich, Steinheim, Germany), in 1% final concentration, was used as a positive control.

HepG2 cells were subjected to concentrations of the tested oximes spanning from 3 to 400 μM in durations of 24 h. For utilization of the commercially procured MTS detection reagent assay, cells were cultivated in 96-well plates at a density of 20,000 cells/well and treated with oxime compounds, as in a previously established protocol [37]. After incubation of 24 h at 37 °C in an environment infused with 5% CO_2_, the cells underwent a PBS buffer rinse, and each well received 120 μL of the MTS reagent (diluted with PBS at a 1:6 ratio). In intervals of 0.5 to 3-h color incubation, optical density readings were captured at 492 nm using an Infinite M200PRO plate reader (Tecan Austria GmbH, Salzburg, Austria). Inhibitory concentration (IC_50_) values represent the oxime concentration responsible for a 50% cell mortality rate. The DMSO content within the assay remained consistent at 0.4% and posed no perceptible impact on cell vitality. Data were evaluated using predefined equations from Prism 9 software (GraphPad by Dotmatics, Boston, MA, USA).

## 4. Conclusions

**Figure 8:**
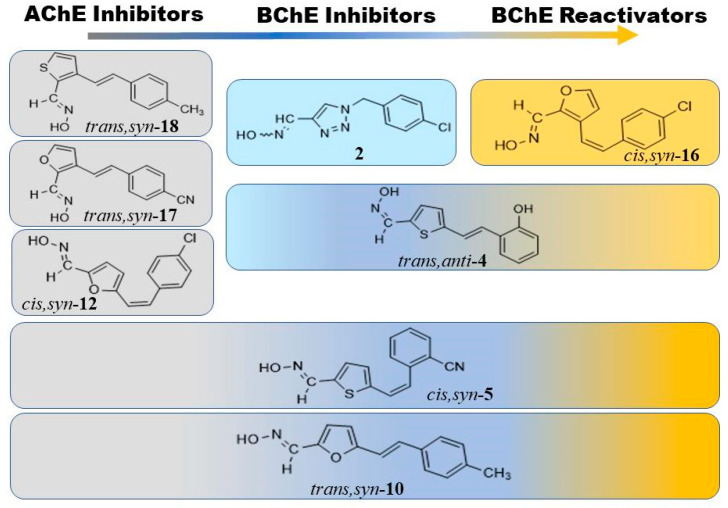
New uncharged heterocyclic oximes are potent human AChE (grey) and human BChE (blue) inhibitors and reactivators of human BChE that are inhibited by cyclosarin (yellow).

This paper presents the design and synthesis of uncharged furan, thiophene, and triazole oximes using an economical synthesis using the Wittig reaction and Vilsmeier formylation in high yields. The expected targeted products, 26 compounds, were pure *cis*- and *trans*-isomers of *syn*- and *anti*-oximes containing different substituents bound in the *para*- or *ortho*-position of the benzene ring. Based on their binding affinity, reactivation potency, estimated ADME, and hepatotoxicity properties, several compounds should be highlighted as potential CNS-active and ChE-targeted therapeutics (Figure 8). The list is headed by two selective BChE inhibitors—*trans,anti*-**4** and **2**, three selective AChE inhibitors—*trans,syn*-**18**, *trans,syn*-**17**, and *cis,syn*-**12**, and two non-selective inhibitors of ChE–*cis,syn*-**5** and *trans,syn*-**10**. These compounds present a new scaffold for drug development because the current therapy in AD, which basically increases acetylcholine levels in synapses, are selective AChE inhibitors. In Parkinson’s and all stages of AD, non-selective inhibitors of ChE are preferred [21], while selective BChE inhibitors are promising therapeutics for later stages of AD since BChE activity in certain brain regions increases up to 120% of its normal values during AD progression [33,61]. Even more importantly, in this study, we identified CNS-active BChE reactivators–among which *cis*-derivatives *cis,syn*-**5** and *cis,syn*-**16** showed an exceptional potential for reactivation of cyclosarin-inhibited BChE. The productive and energy-efficient positioning in the active site, as reported with the near-attack modeling, along with an optimal geometry of the reactivation transition state, returns BChE activity within a short amount of time. Furthermore, as uncharged ligands, these leading compounds are predicted to cross the BBB efficiently, and therefore, they could potentially achieve significant concentrations both at the neuromuscular junction and in the brain and, as such, could result in an overall improved therapeutic outcome after OP poisonings or for use in neuromuscular diseases and neurodegenerative disorders.

## Figures and Tables

**Table 1:** The cytotoxicity of new uncharged oximes **1**–**21** evaluated by MTS assay after 24-h liver cell line (HepG2) exposure. Data were calculated from at least three experiments.

Oxime	IC_50_/μM
	HepG2
**1** ^a^	>400
**2** ^a^	>400
**3** ^a^	>400
*trans,anti*-**4**	191 ± 21
*cis,syn*-**5**	≥400
*trans,anti*-**6**	≥400
*trans*-**7** ^a^	>400
*trans*-**8** ^a^	>400
*trans,syn*-**9**	>400
*trans,syn*-**10**	105 ± 4
*trans,anti*-**10**	>400
*cis,syn*-**12**	226 ± 10
**13** ^a^	>400
*cis,syn*-**14**	182 ± 32
*trans,syn*-**14**	>400
*trans,anti*-**15**	>400
*cis,syn*-**16**	65 ± 13
*trans,syn*-**16**	>400
*cis,syn*-**17**	120 ± 6
*trans,syn*-**17**	>400
*cis,syn*-**18**	269 ± 38
*cis,anti*-**18**	>400
*trans,syn*-**18**	>400
*cis,syn*-**19′**	>400
*cis,syn*-**21**	118 ± 46
*cis,anti*-**21**	182 ± 13

^a^ mixture of configurational isomers.

**Table 2:** Dissociation constants (*K*_i_ ± S.E.) for reversible inhibition of human AChE and BChE with uncharged oximes at 25 °C from at least three experiments.

Oxime	*K*_i_/μM	
	AChE	BChE
**1** ^a^	271 ± 29	503 ± 32
**2** ^a^	267 ± 92	24 ± 2.9
**3** ^a^	280 ± 30	303 ± 66
*trans,anti*-**4**	258 ± 90	6.5 ± 0.5
*cis,syn*-**5**	112 ± 21	17 ± 2.0
*trans,anti*-**6**	nd ^b^	123 ± 23
*trans*-**7** ^a^	147 ± 43	49 ± 11
*trans*-**8** ^a^	199 ± 38	143 ± 22
*trans,syn*-**9**	172 ± 29	303 ± 65
*trans,syn*-**10**	111 ± 45	38 ± 9.8
*trans,anti*-**10**	180 ± 40	nd
*cis,syn*-**12**	68 ± 37	147 ± 37
*trans*-**13** ^a^	nd	nd
*cis,syn*-**14**	627 ± 245	93 ± 30
*trans,syn*-**14**	nd	122 ± 47
*trans,anti*-**15**	nd	nd
*cis,syn*-**16**	nd	119 ± 44
*trans,syn*-**16**	217 ± 160	nd
*cis,syn*-**17**	544 ± 105	nd
*trans,syn*-**17**	66 ± 24	nd
*cis,syn*-**18**	nd	86 ± 32
*cis,anti*-**18**	nd	760 ± 122
*trans,syn*-**18**	59 ± 39	nd
*cis,syn*-**19′**	nd	nd
*cis,syn*-**21**	171 ± 68	59 ± 8.0
*cis,anti*-**21**	187 ± 54	53 ± 10
tacrine ^c^	0.040 ± 0.006	0.0063 ± 0.0010

^a^ mixture of configurational isomers; ^b^ nd—not determined due to enzyme activity was higher than 80% at the highest oxime concentration; ^c^ [21].

**Table 3:** Reactivation of cyclosarin-inhibited BChE by the selected uncharged oximes. The determined parameters (mean ± S.E.)-maximal reactivation rate constant (*k*_2_), dissociation constant (*K*_OX_), overall reactivation rate constant (*k*_r_), maximal percentage of reactivation (React_max_) and time (t) in which maximal reactivation was achieved were determined from at least three experiments at 25 °C.

Oxime	*k*_2/_min^−1^	*K*_OX/_µM	*k*_r/_M^−1^ min^−1^	React_max/_%	t/min
*trans,anti*-**4**	- ^a^	-	592 ± 79	50	50
*cis,syn*-**5**	0.11 ± 0.02	105.2 ± 47.3	1082 ± 270	70	60
*trans,syn*-**10**	-	-	240 ± 14	70	140
*cis,syn*-**16**	-	-	424 ± 39	80	105
HI-**6** ^b^	-	-	780 ± 30	90	30

^a^ Not evaluated due to a linear dependence of *k*_obs_ and oxime concentration (0.01–0.2 mM selected oximes and 0.1–1 mM HI-6). ^b^ from [29].

## Data Availability

The data presented in this study are available on request from the corresponding author. The data are not publicly available due to privacy.

## Supplementary Materials

The following supporting information can be downloaded at: https://www.mdpi.com/article/10.3390/biom14060679/s1, NMR spectra (Figures S1–S282), mass spectra and HRMS analyses (Figures S283–S298), evaluation of cytotoxicity (Figure S299), inhibition of AChE and BChE with selected oximes (Figure S300), molecular docking of human AChE and BChE (Figure S301), molecular dynamics of oxime *cis,syn*-**5** (Figure S302), molecular dynamics of oxime *cis,syn*-**16** (Figure S303).
