# Supplementary material for: New Heterostilbene and Triazole Oximes as Potential CNS-Active and Cholinesterase-Targeted Therapeutics
Source: Biomolecules. 2024 Jun 11;14(6):679. doi: 10.3390/biom14060679 (PMC11201660; doi:10.3390/biom14060679)
Supplement: Supplementary file 1 [file biomolecules-14-00679-s001.zip › biomolecules-3029633-supplementary.pdf]

New heterostilbene and triazole oximes as potential CNS-active and cholinesterase-targeted therapeutics

Milena Mlakić[1,£], Tena Čadež[2,£], Goran Šinko[2], Irena Škorić[1,*] and Zrinka Kovarik[2,3,*]

[1] Department of Organic Chemistry, Faculty of Chemical Engineering and Technology,
University of Zagreb, Trg Marka Marulića 19, HR-10 000 Zagreb, Croatia
[2] Division of Toxicology, Institute for Medical Research and Occupational Health, Ksaverska cesta 2, HR-10 000 Zagreb, Croatia
[3] University of Zagreb, Faculty of Science, Horvatovac 102a, 10000 Zagreb, Croatia

* Correspondance: zkovarik@imi.hr; iskoric@fkit.unizg.hr

£ These authors contributed equally.

Supplementary Data

Contents

1. NMR spectra of compounds 1-63 2
2. Mass spectra and HRMS analyses of oximes 1-21 284
3. Evaluation of cytotoxicity 300
4. Inhibition of AChE and BChE by selected oximes 301
5. Molecullar docking of human AChE and BChE 302
6. Molecular dynamics simulation of oxime near-attack conformation........ 303

1. NMR spectra of compounds 1-63

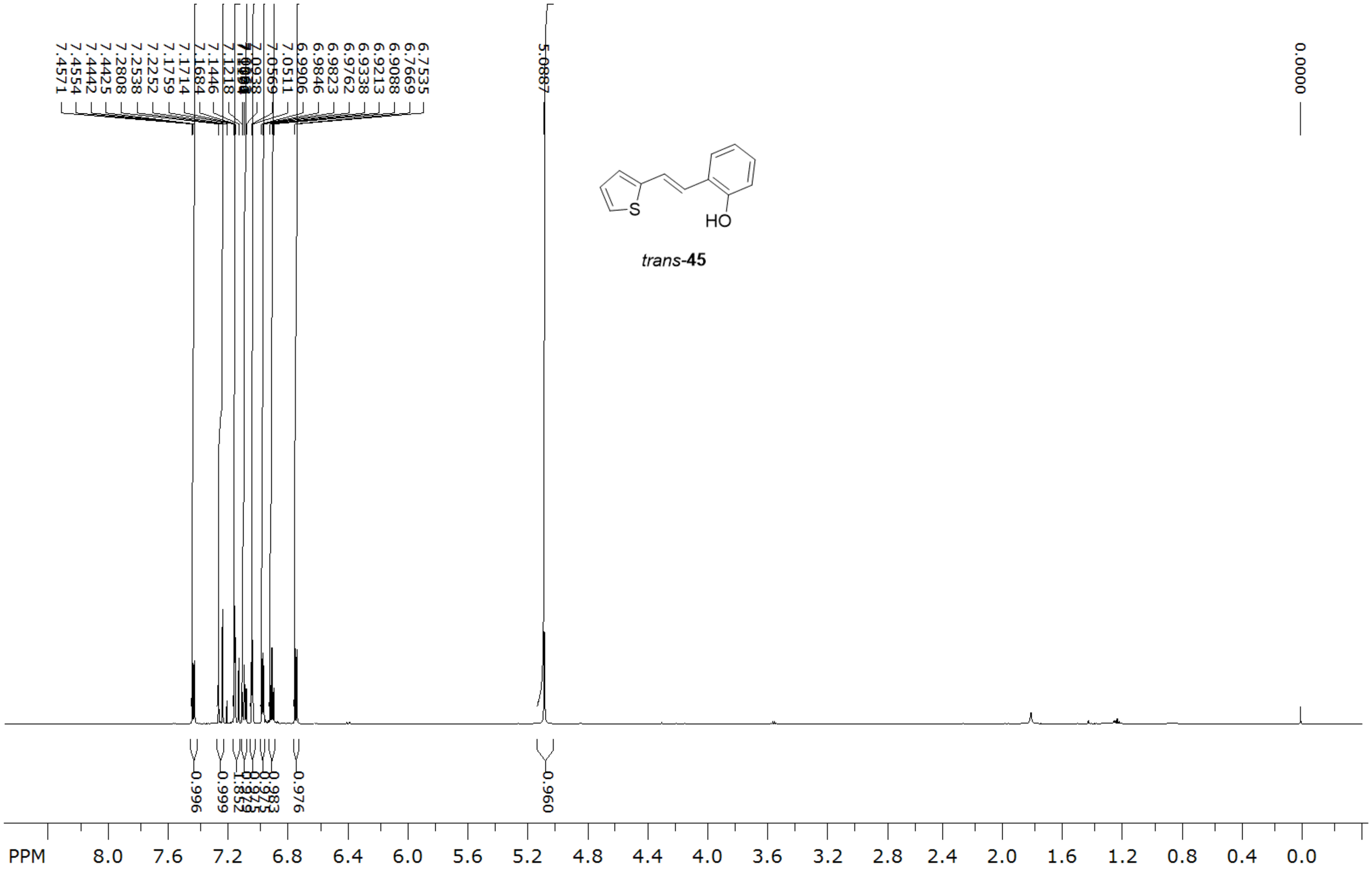

Figure S1. ^{1}H NMR ($CDCl_3$) spectrum of *trans*-**45**.

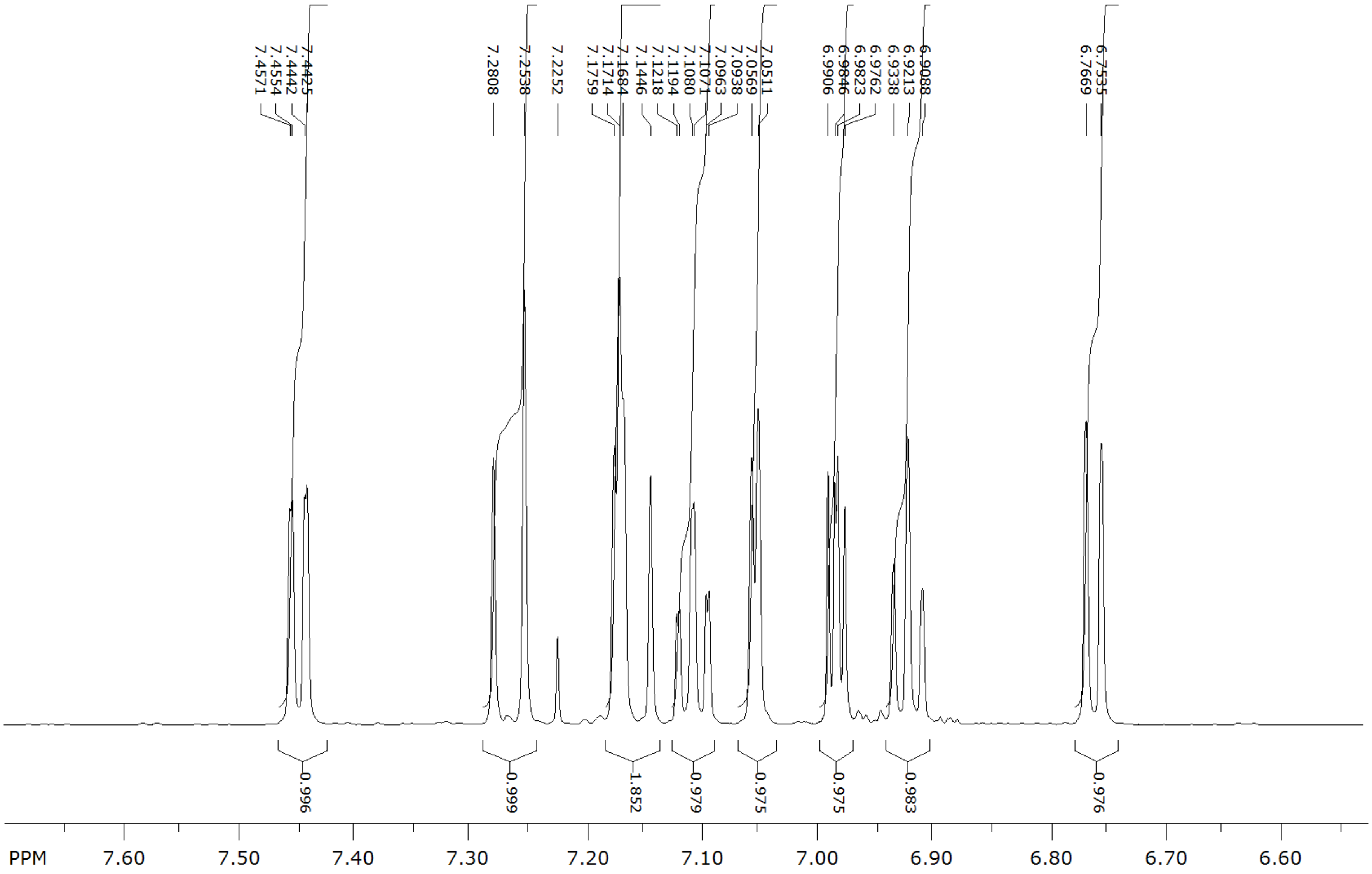

Figure S2. ^{1}H NMR ($CDCl_3$) spectrum of aromatic part of *trans*-**45**.

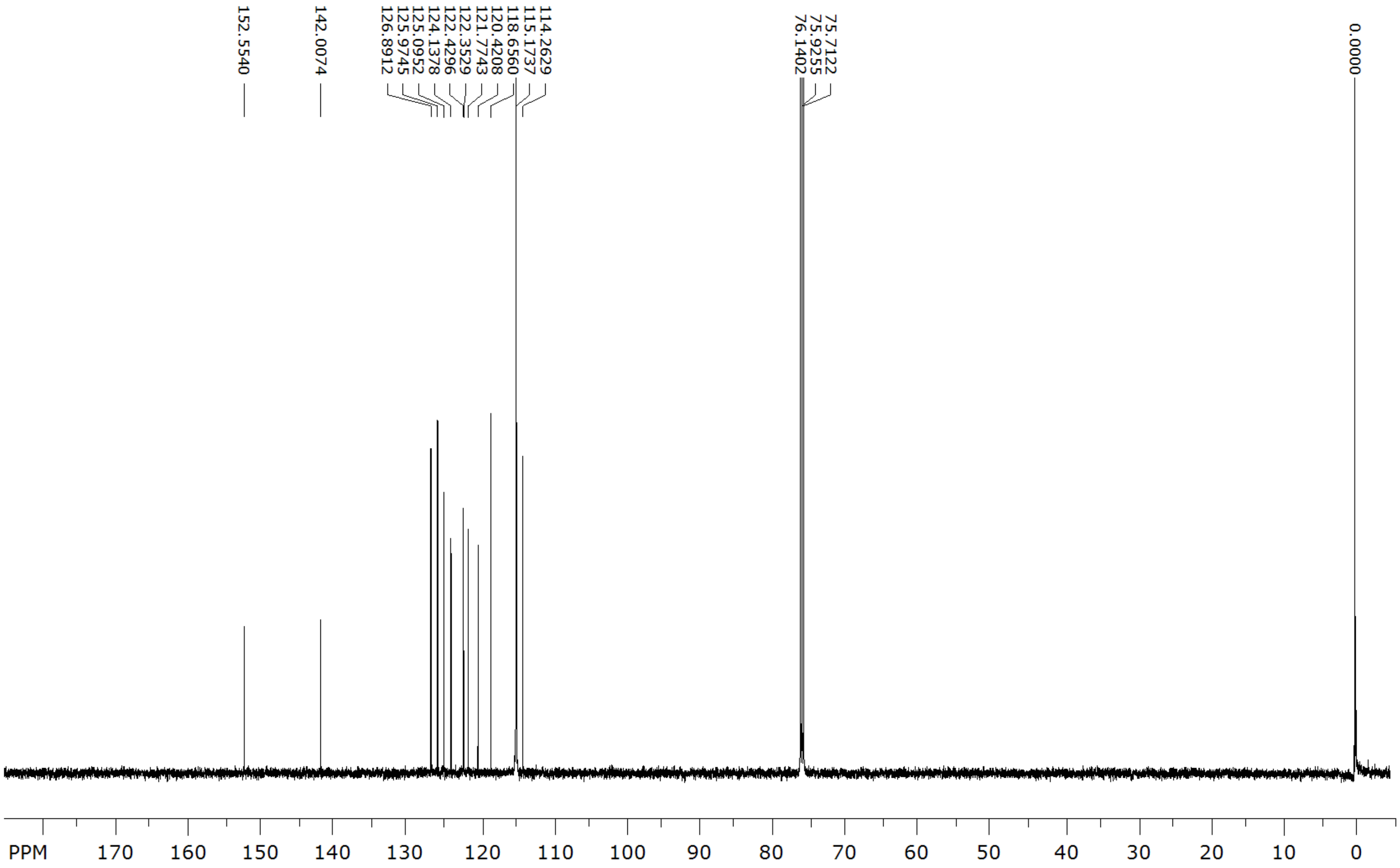

Figure S3. ^{13}C NMR ($CDCl_3$) spectrum of *trans*-**45**.

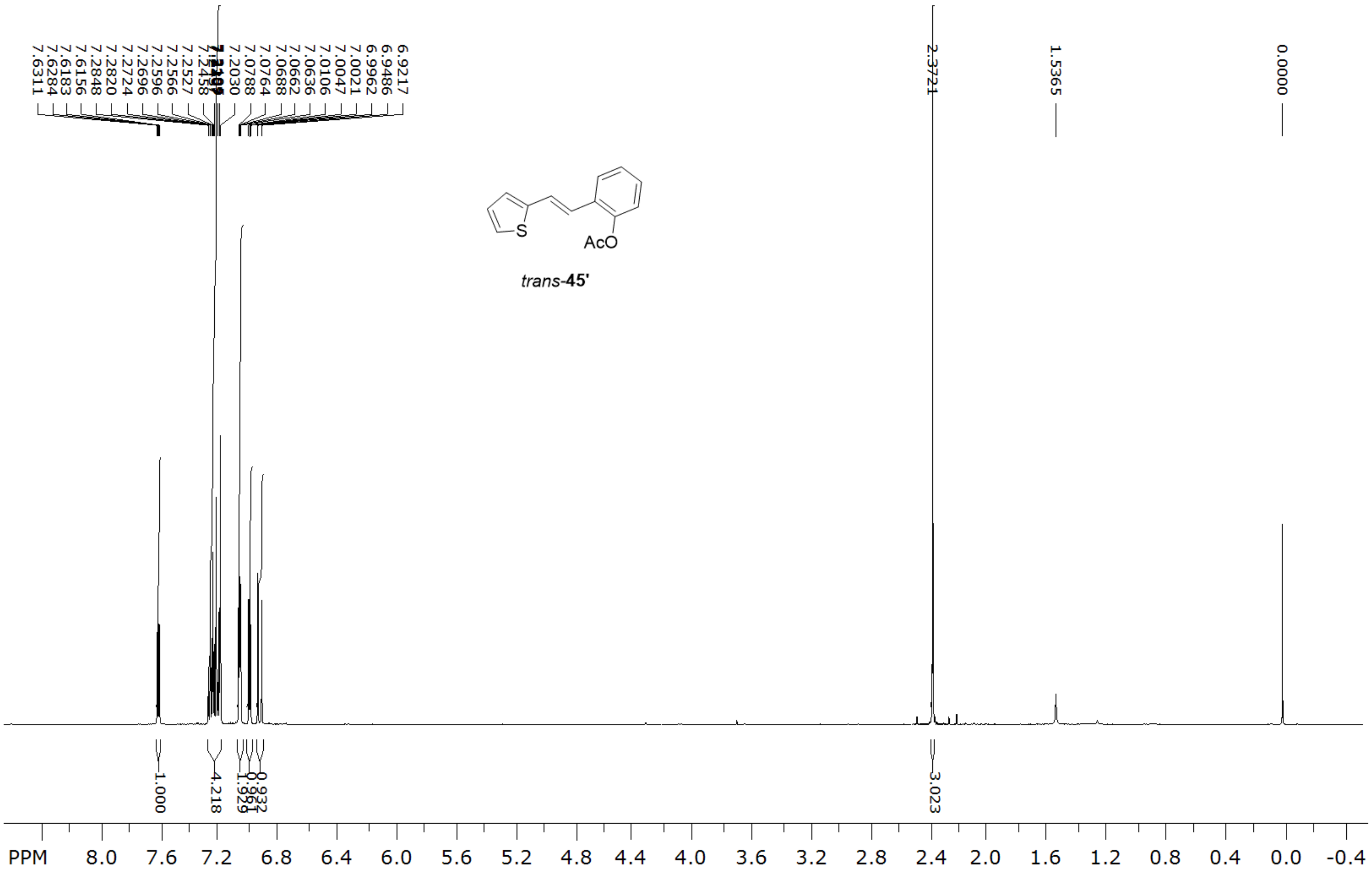

Figure S4. ^{1}H NMR ($CDCl_3$) spectrum of *trans*-**45'**.

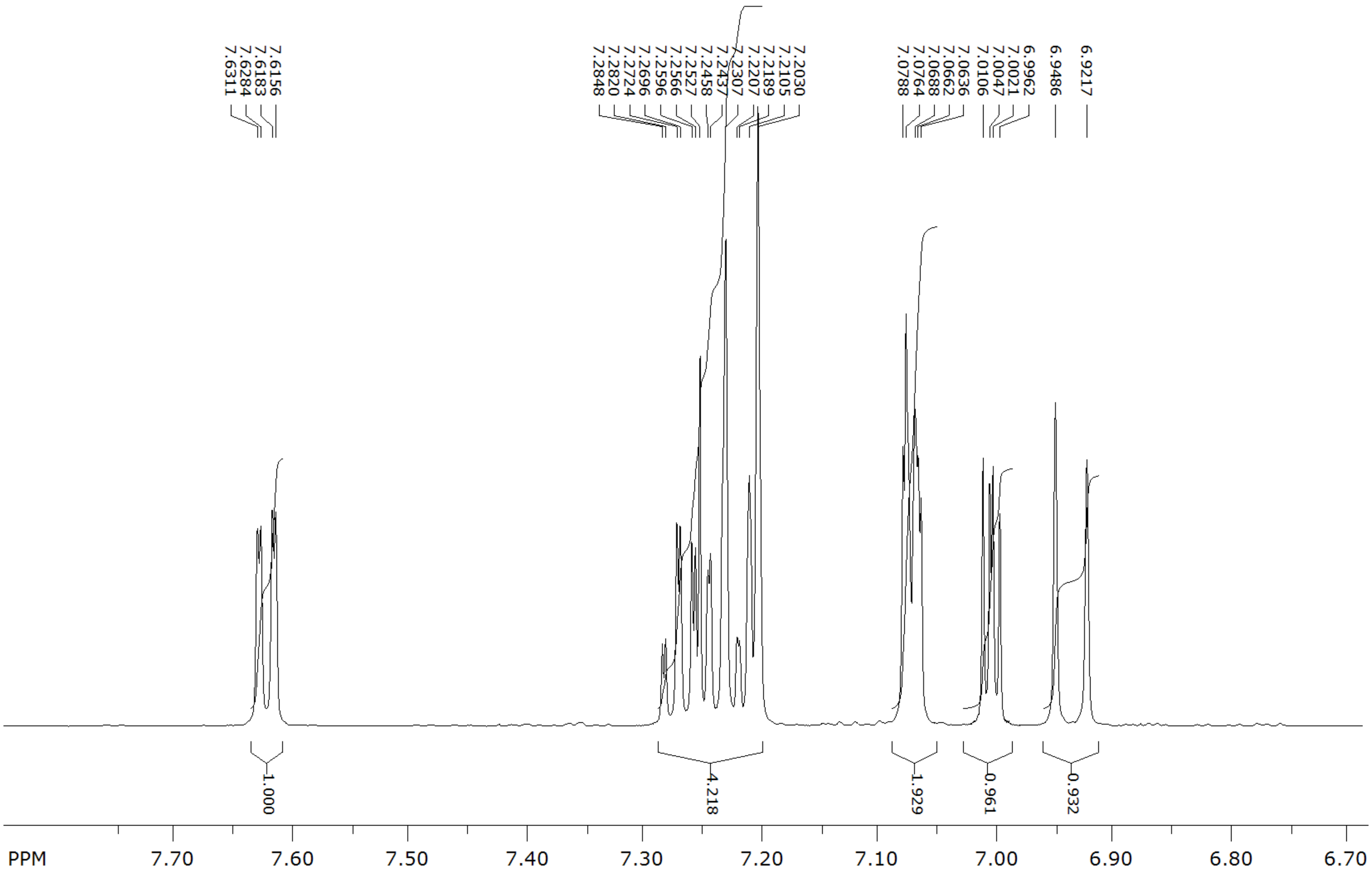

Figure S5. ^{1}H NMR ($CDCl_3$) spectrum of aromatic part of *trans*-**45'**.

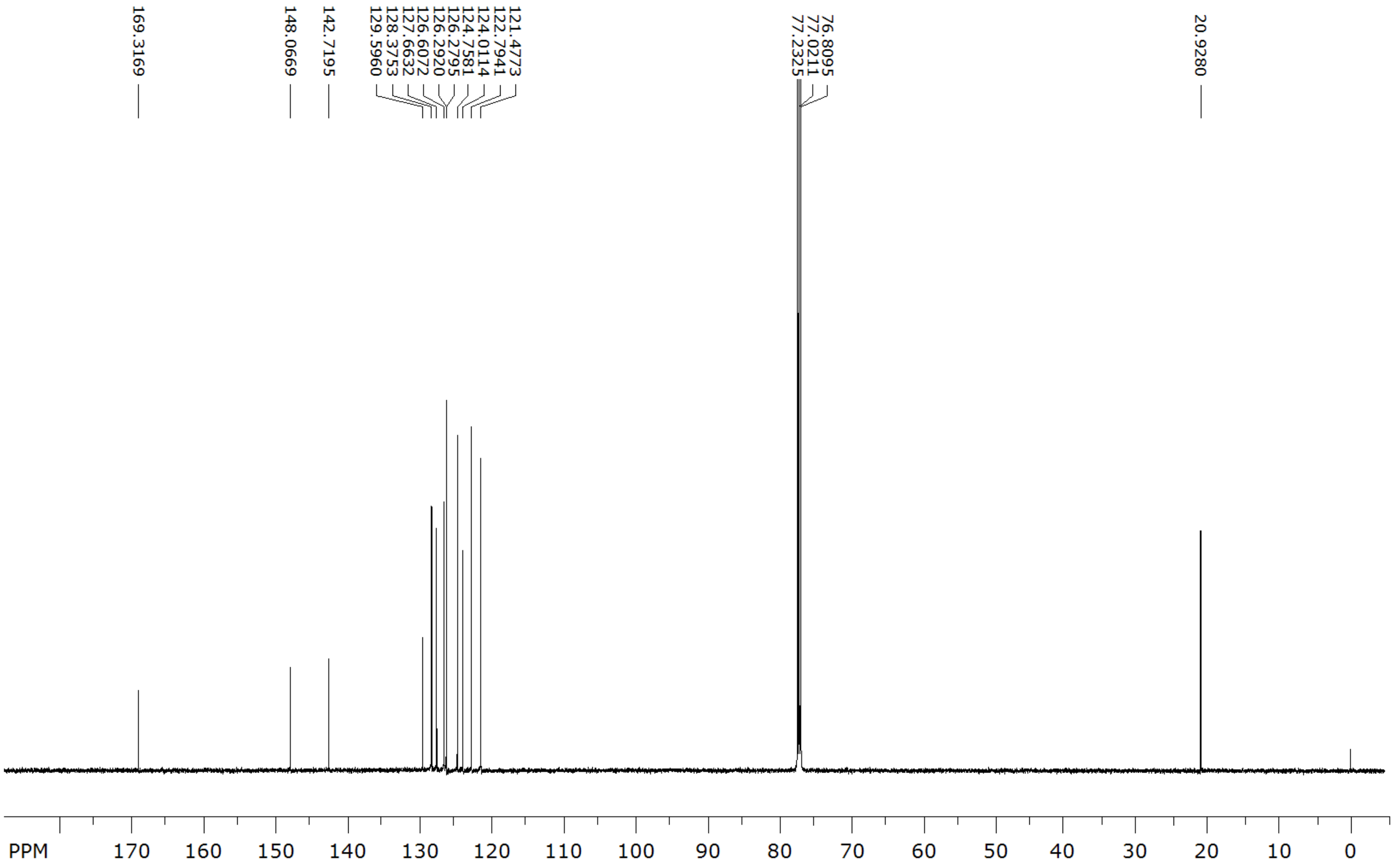

Figure S6. ^{13}C NMR ($CDCl_3$) spectrum of *trans*-**45'**.

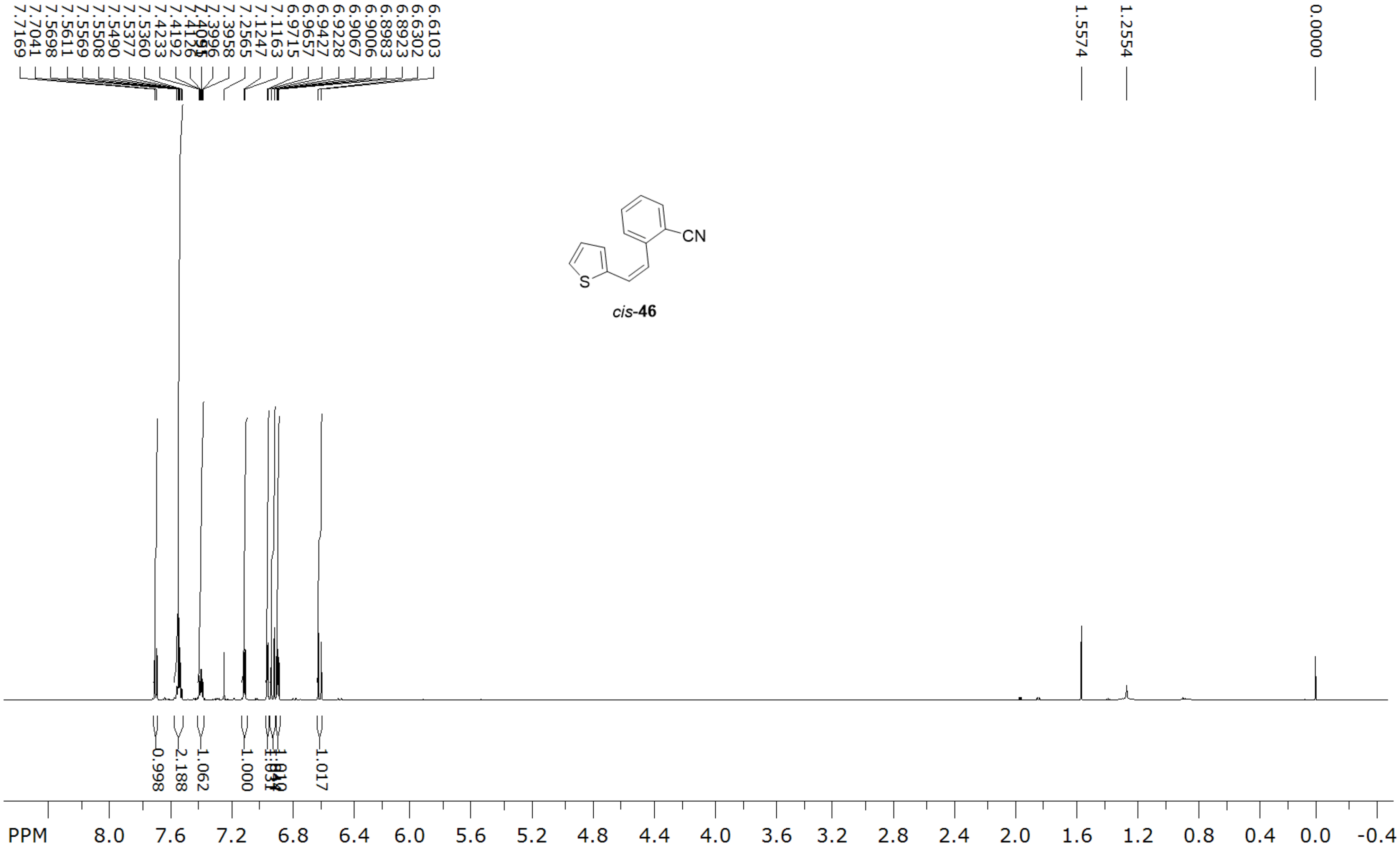

Figure S7. ^{1}H NMR ($CDCl_3$) spectrum of *cis*-**46**.

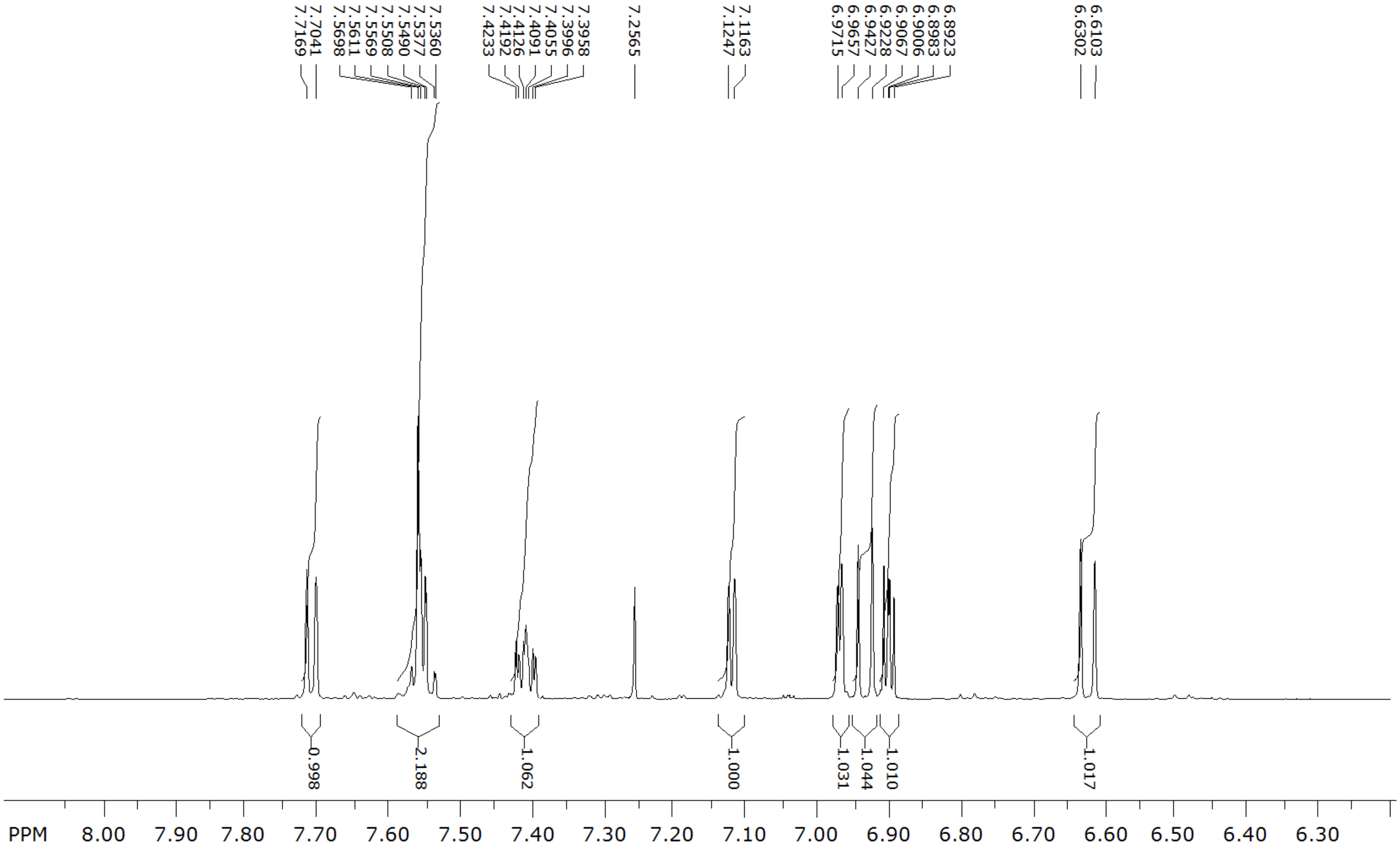

Figure S8. ^{1}H NMR ($CDCl_3$) spectrum of aromatic part of *cis*-**46**.

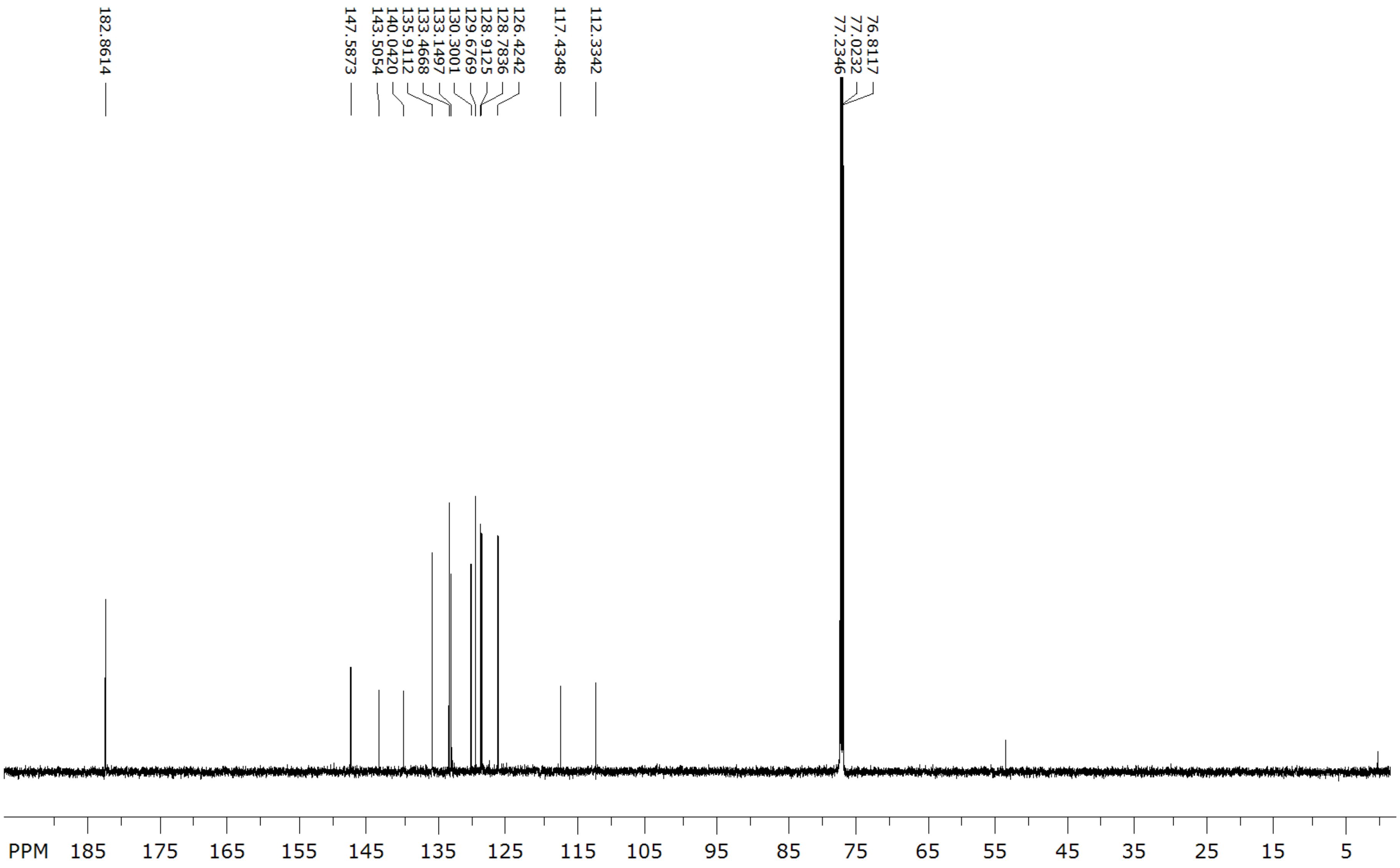

Figure S9. ^{13}C NMR ($CDCl_3$) spectrum of *cis*-**46**.

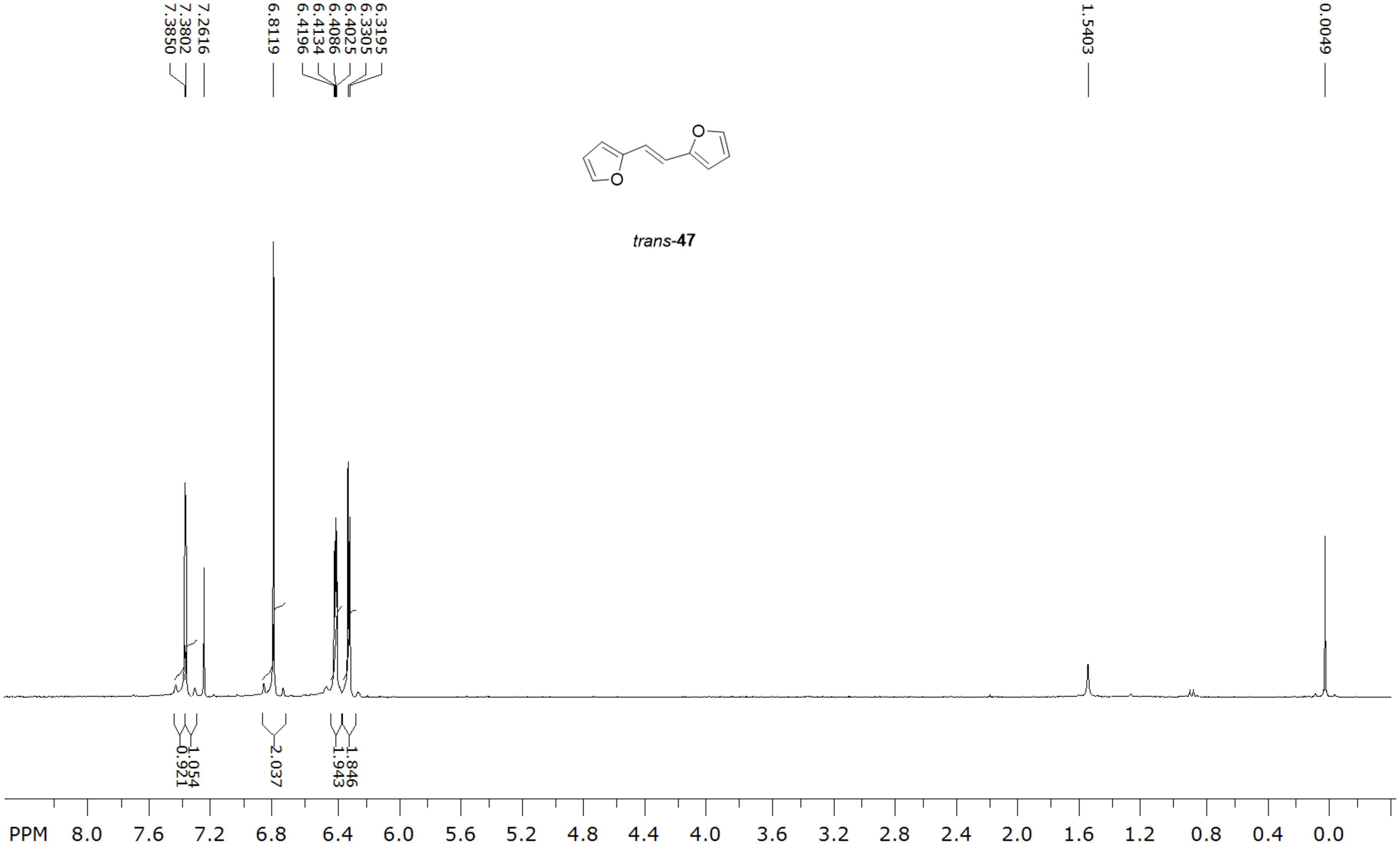

Figure S10. ^{1}H NMR ($CDCl_3$) spectrum of *trans*-**47**.

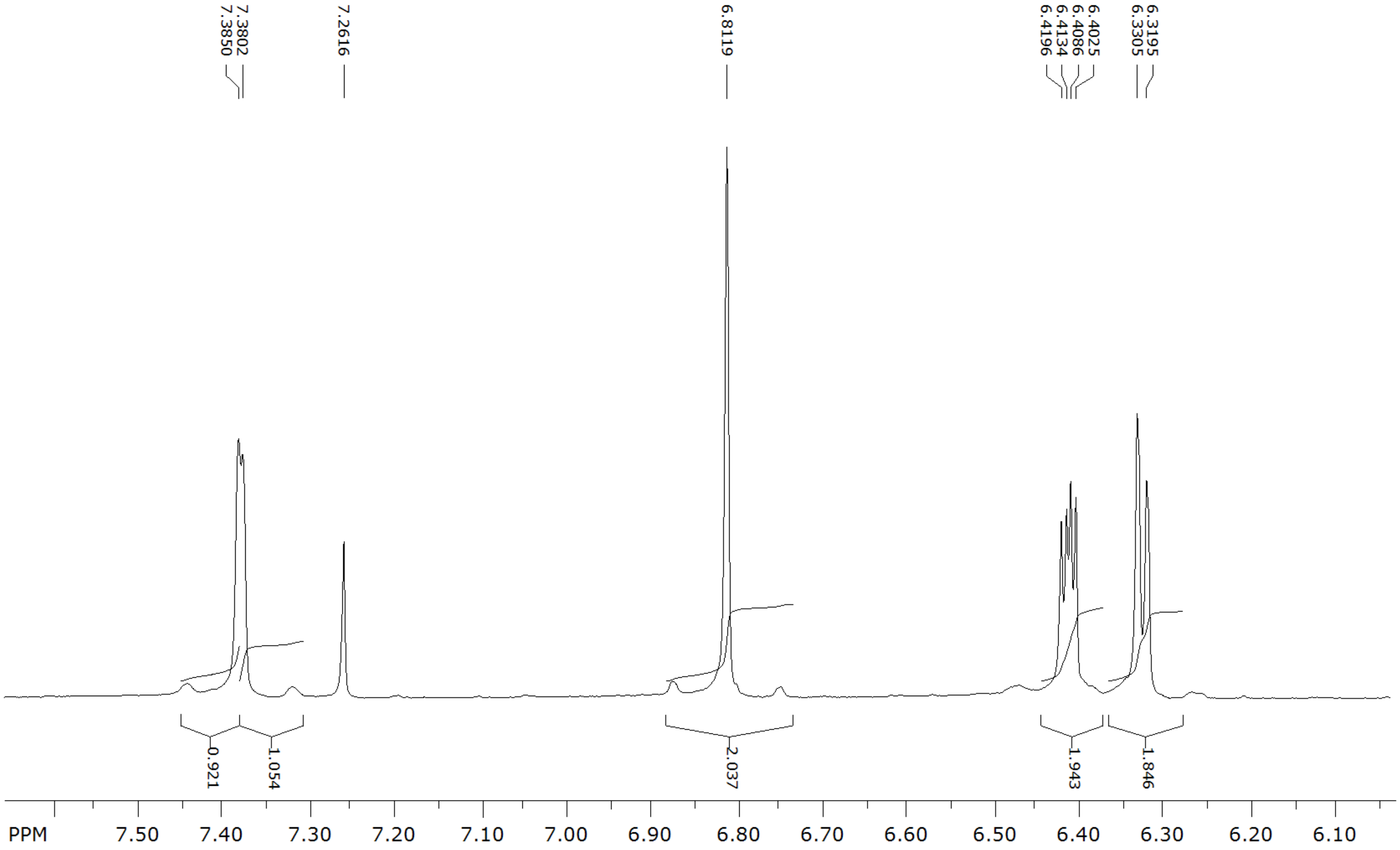

Figure S11. ^{1}H NMR ($CDCl_3$) spectrum of aromatic part of *trans*-**47**.

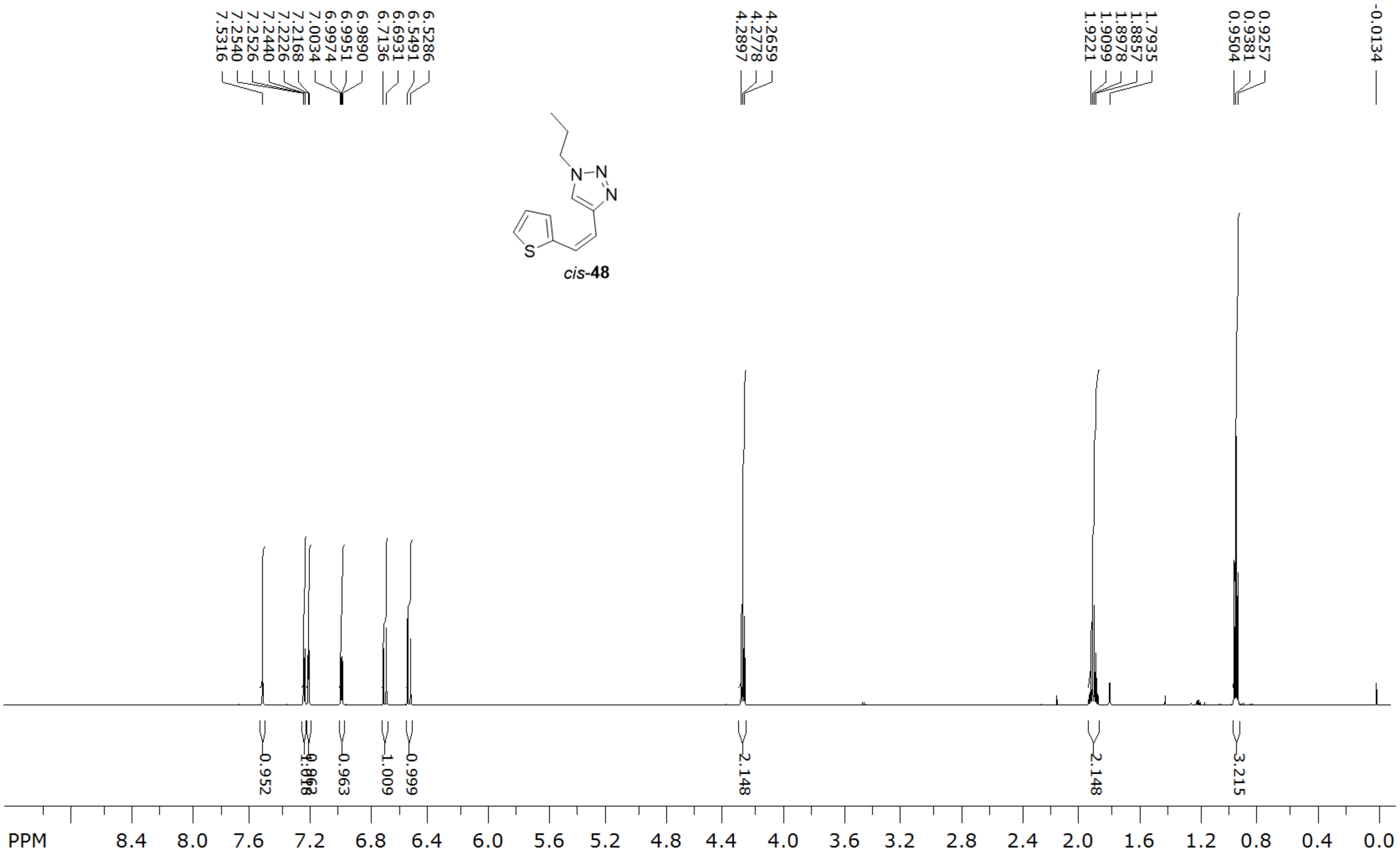

Figure S12. ^{1}H NMR ($CDCl_3$) spectrum of *cis*-**48**.

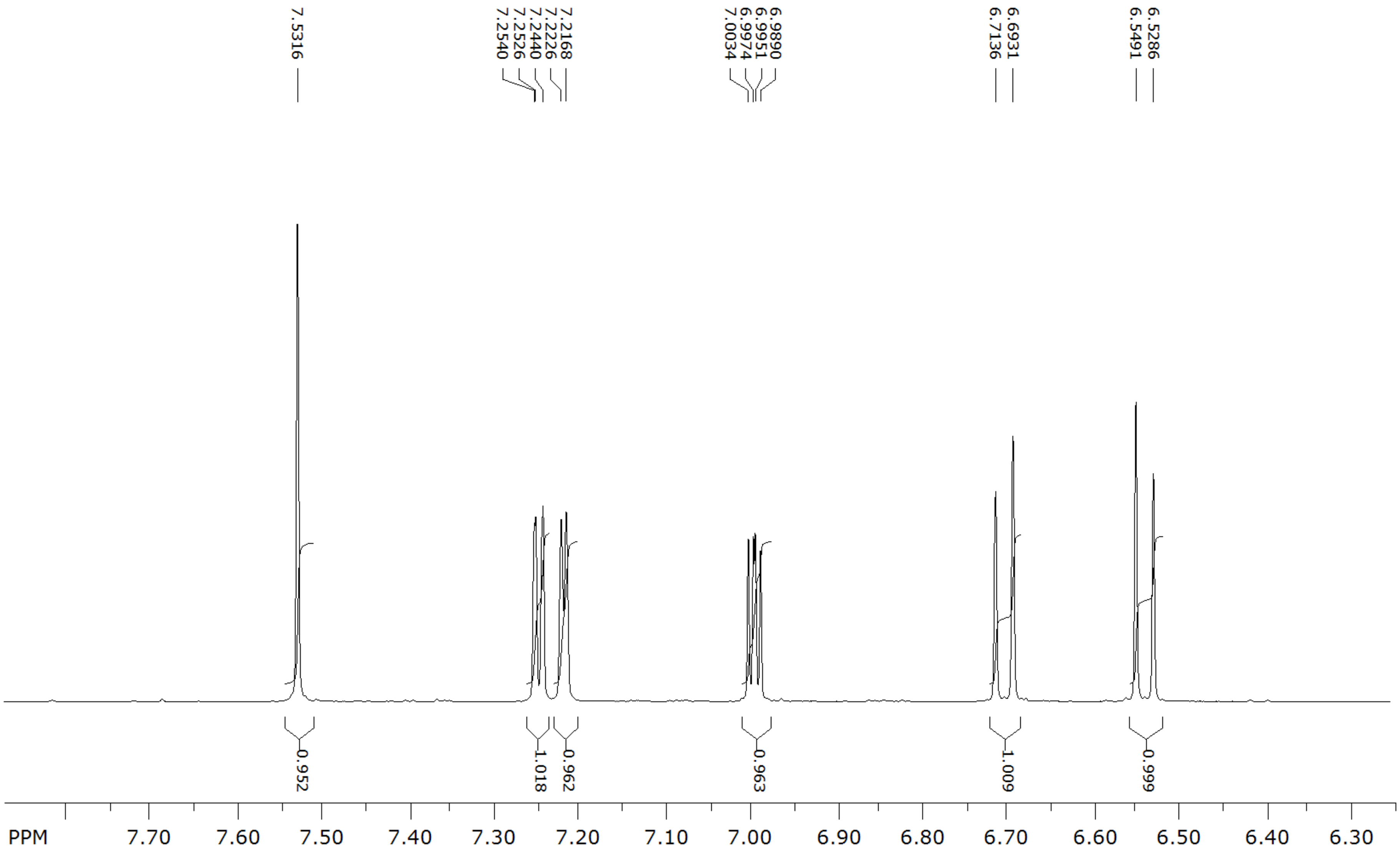

Figure S13. ^{1}H NMR ($CDCl_3$) spectrum of aromatic part of *cis*-**48**.

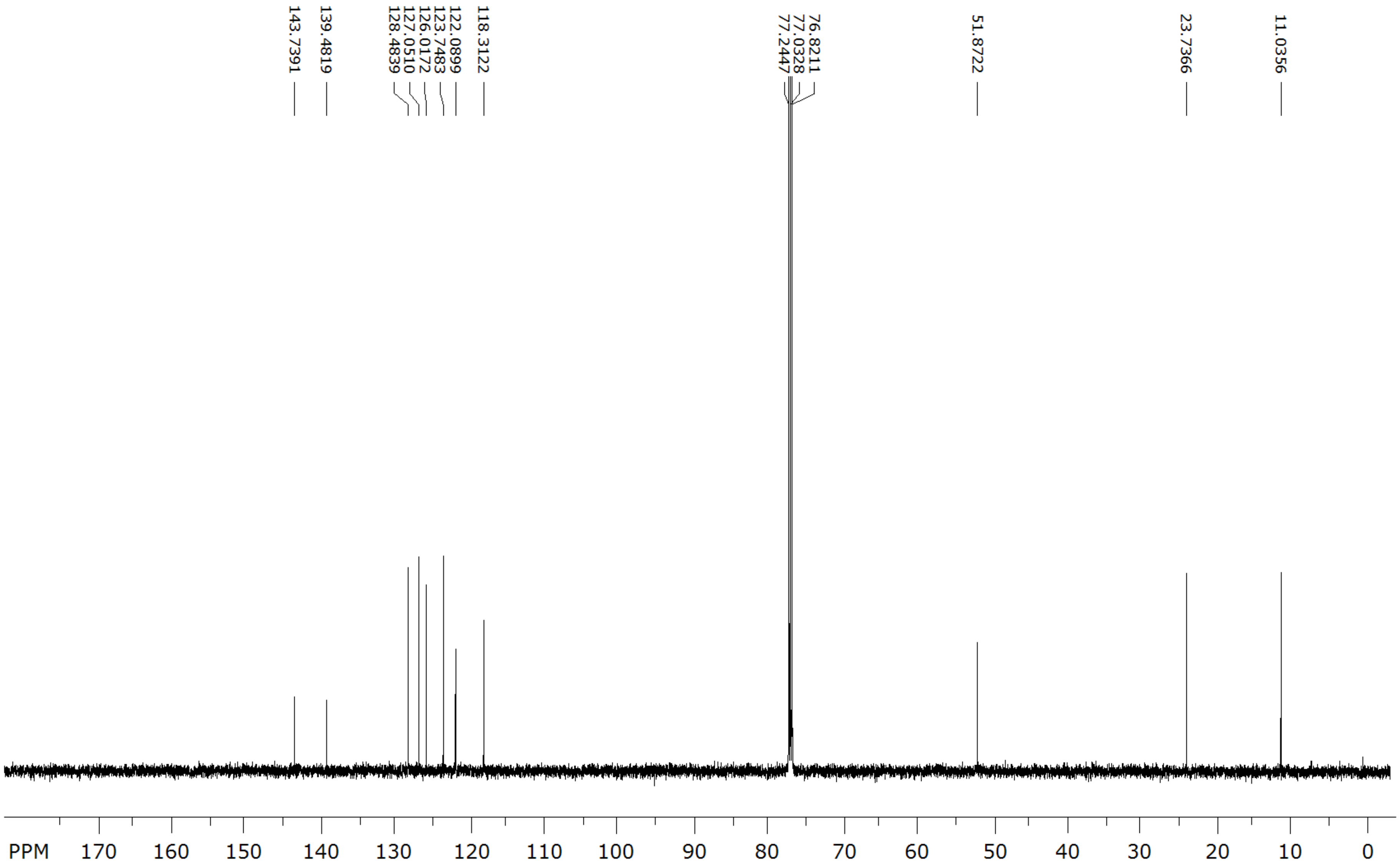

Figure S14. ^{13}C NMR ($CDCl_3$) spectrum of *cis*-**48**.

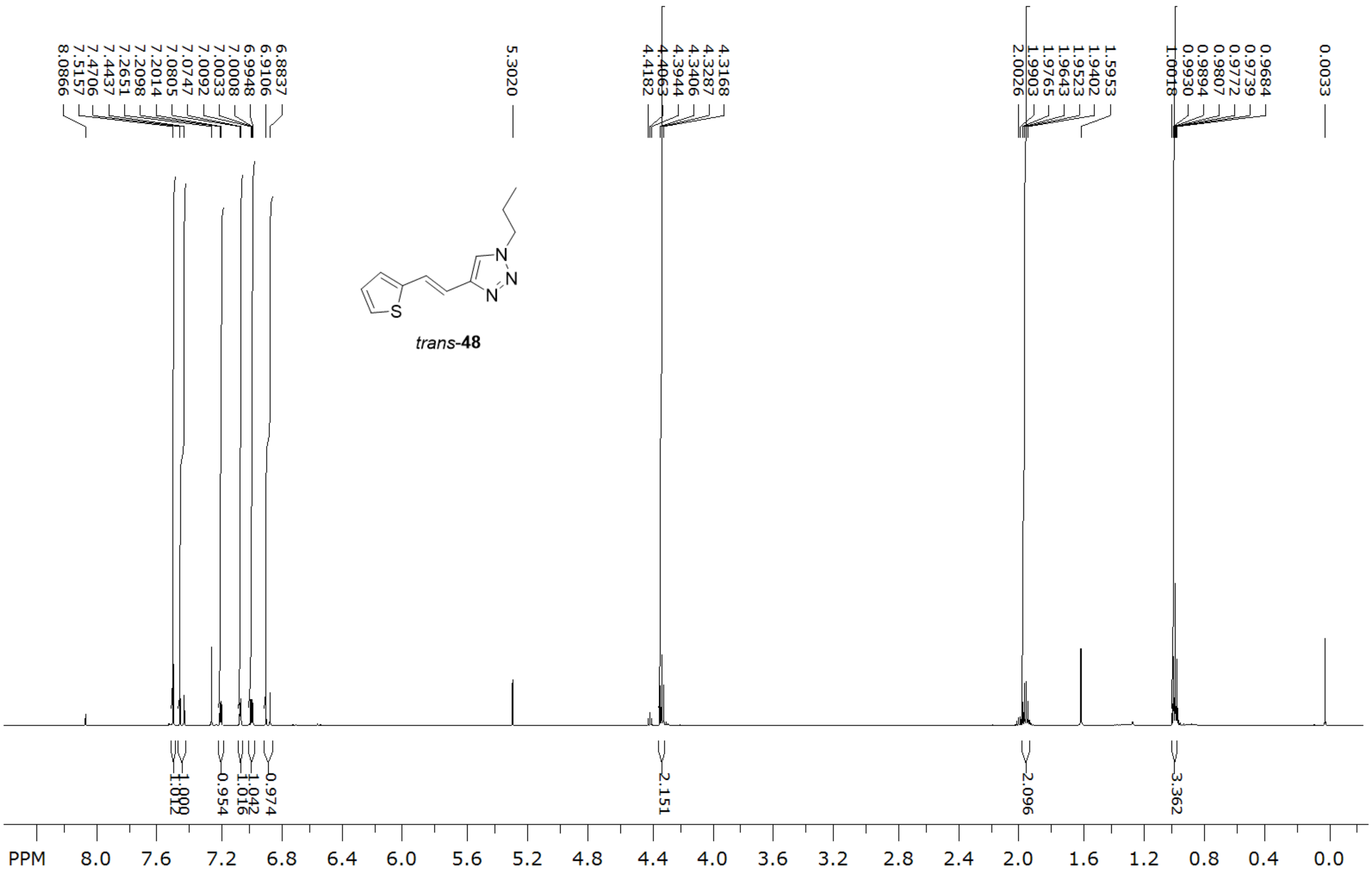

Figure S15. ^1H NMR ($CDCl_3$) spectrum of *trans*-**48**.

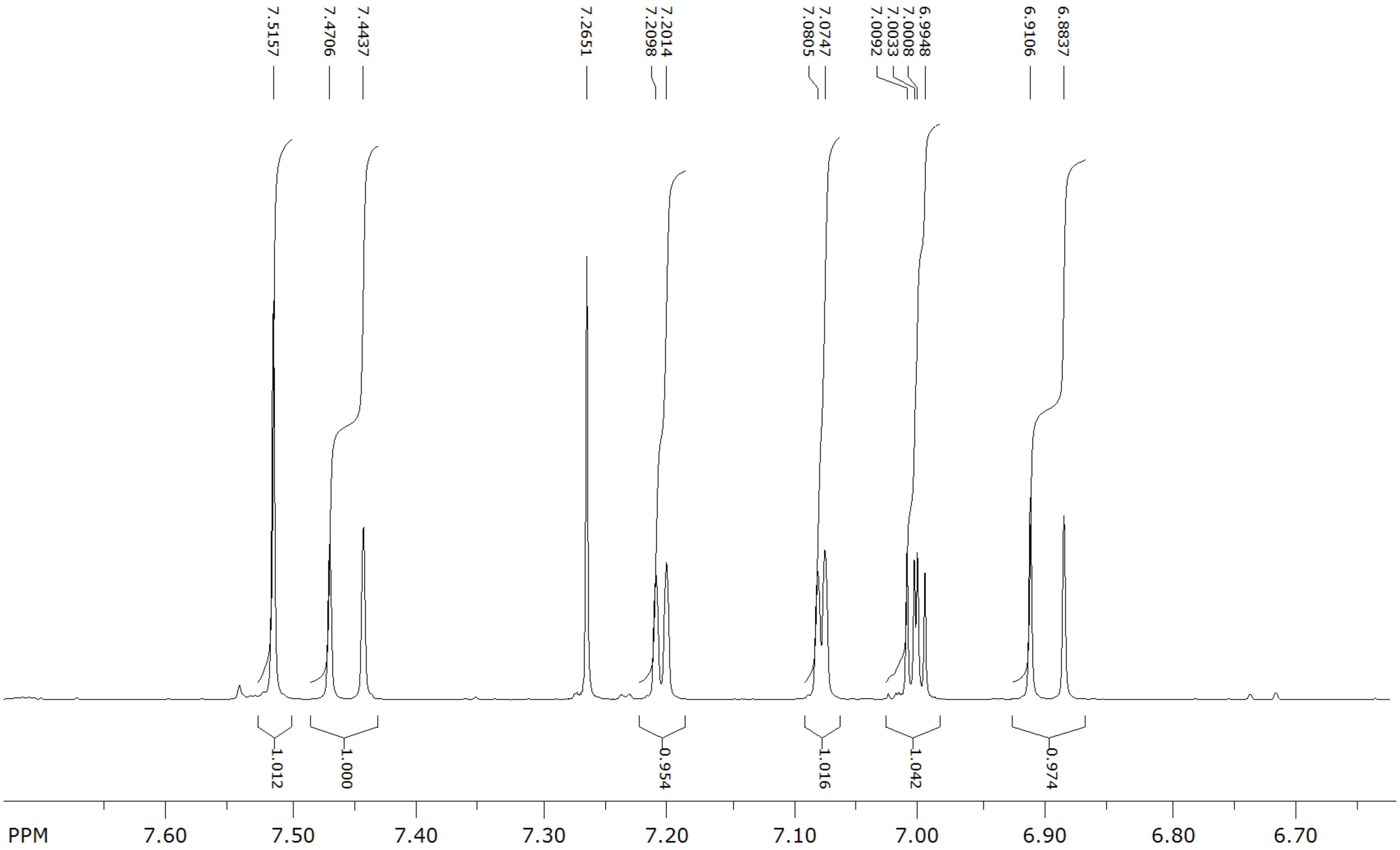

Figure S16. ^{1}H NMR ($CDCl_3$) spectrum of aromatic part of *trans*-**48**.

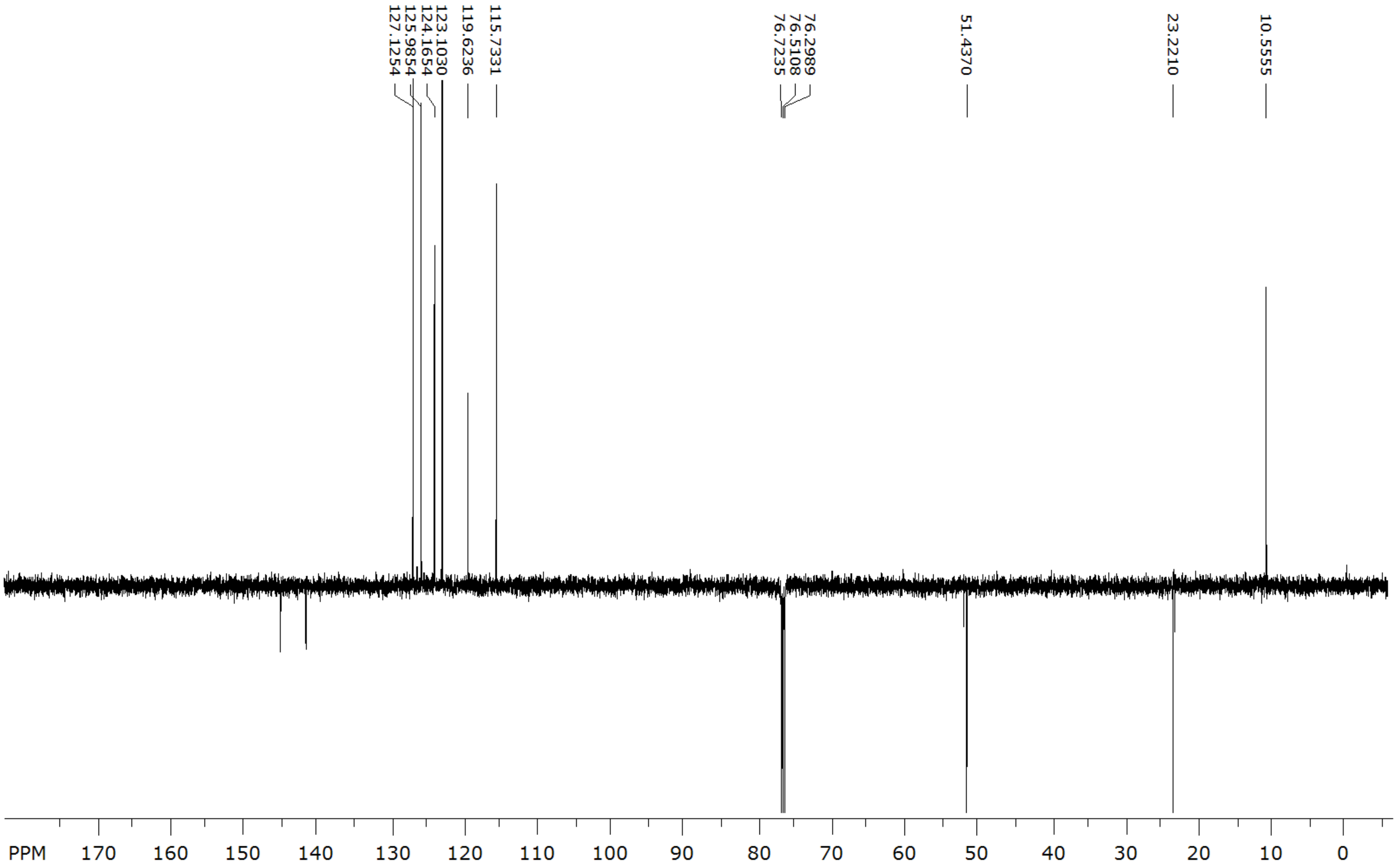

Figure S17. ^{13}C NMR ($CDCl_3$) spectrum of *trans*-**48**.

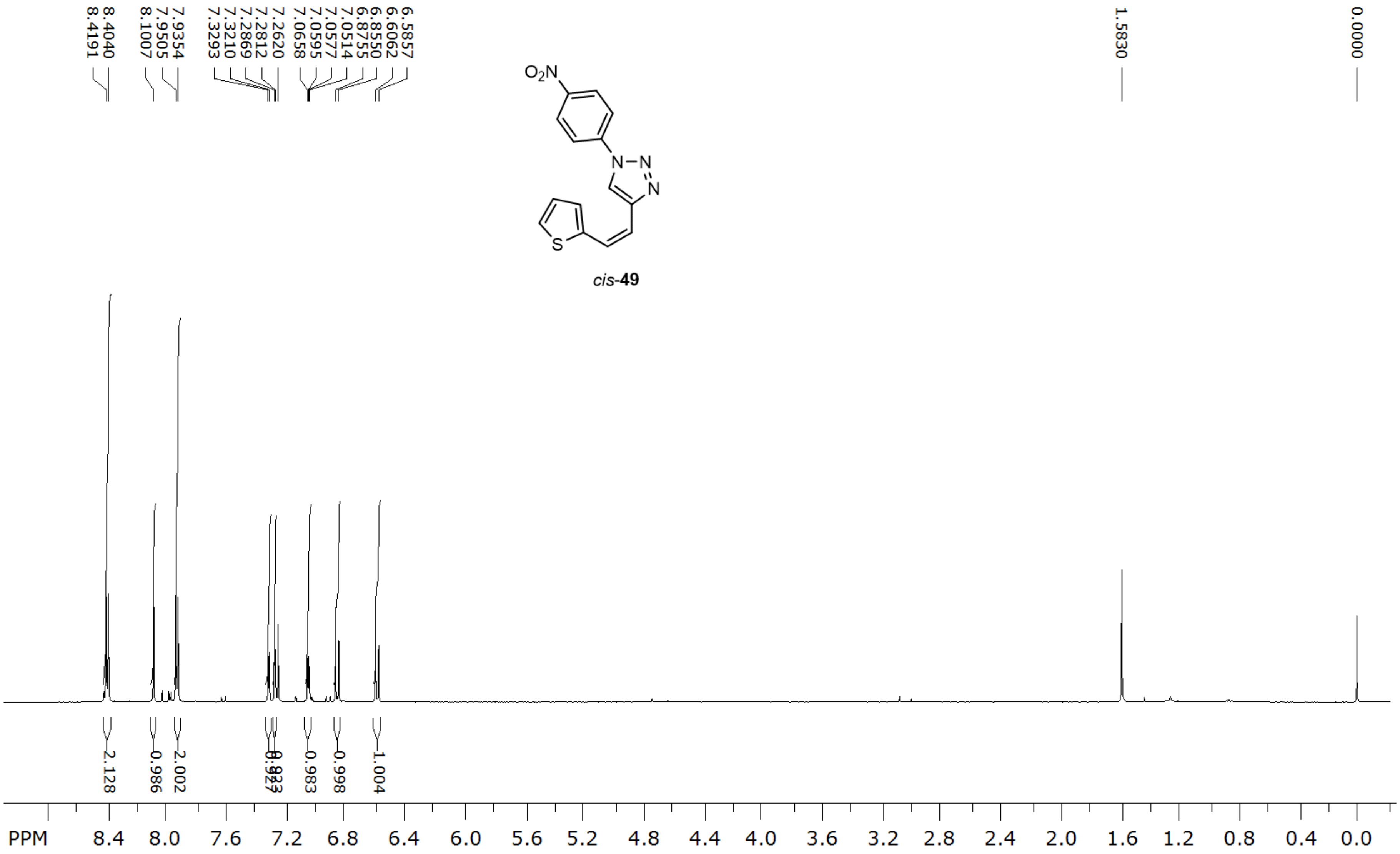

Figure S18. ^{1}H NMR ($CDCl_3$) spectrum of *cis*-**49**.

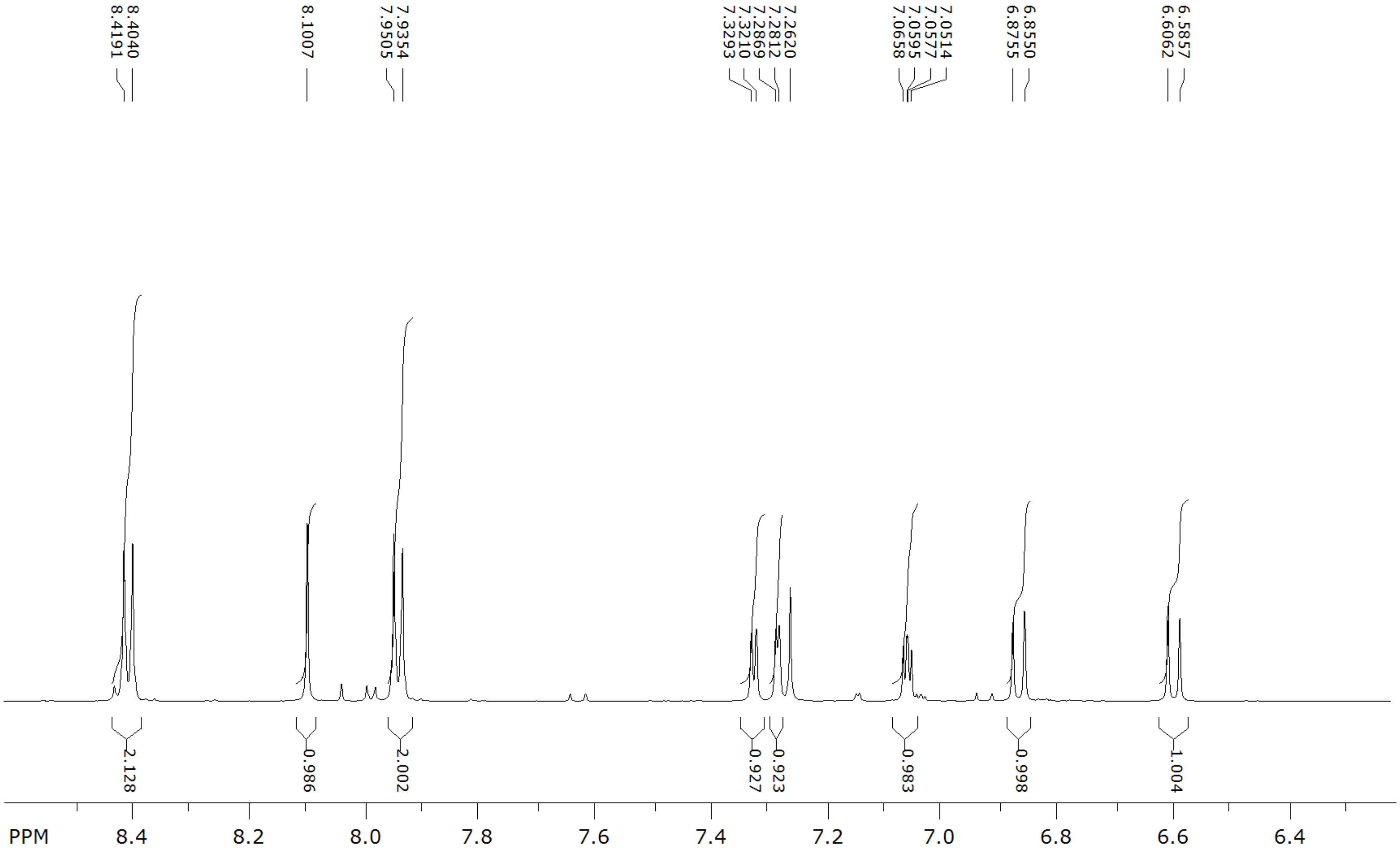

Figure S19. ^{1}H NMR ($CDCl_3$) spectrum of aromatic part of *cis*-**49**.

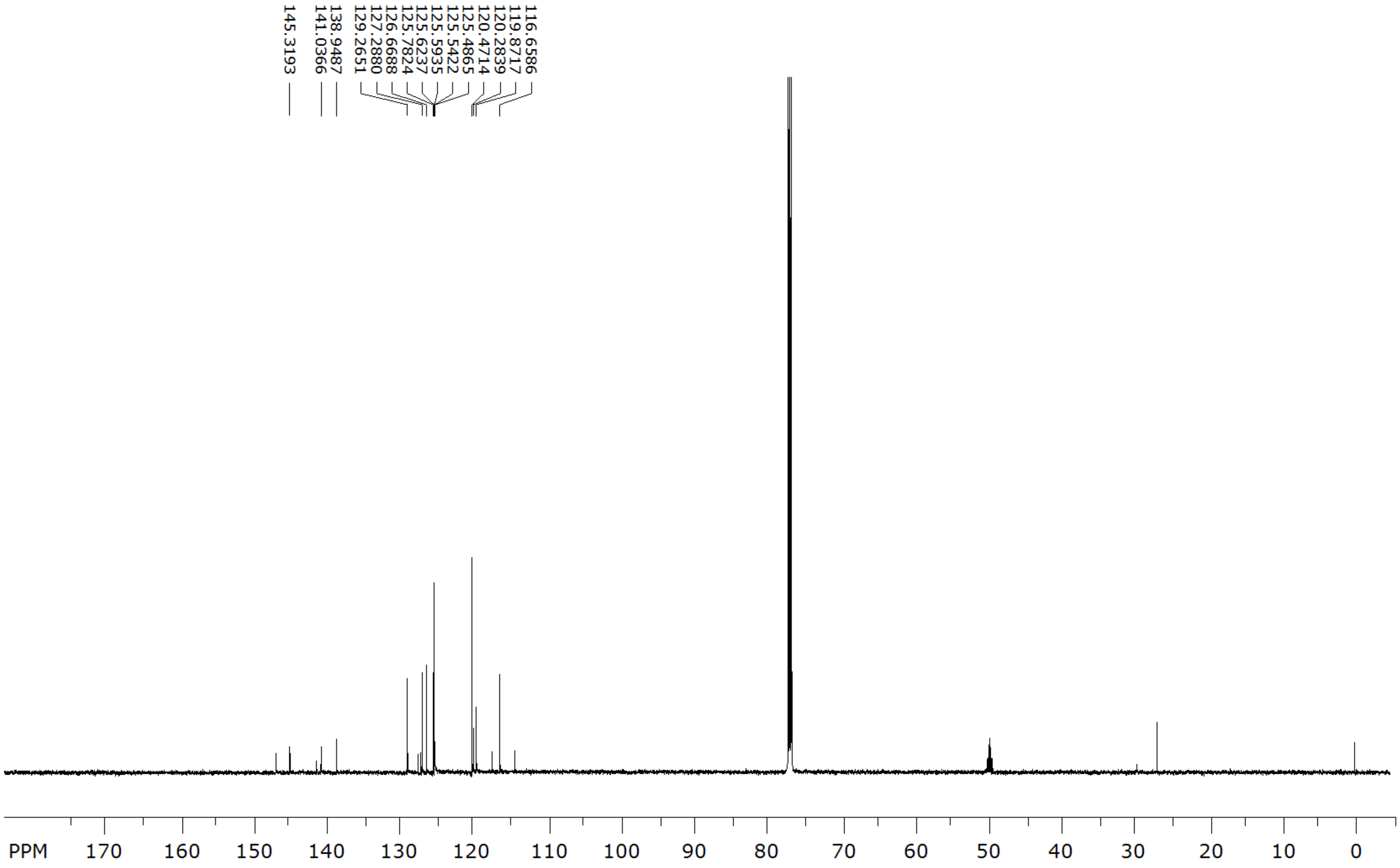

Figure S20. ^{13}C NMR ($CDCl_3$) spectrum of *cis*-**49**.

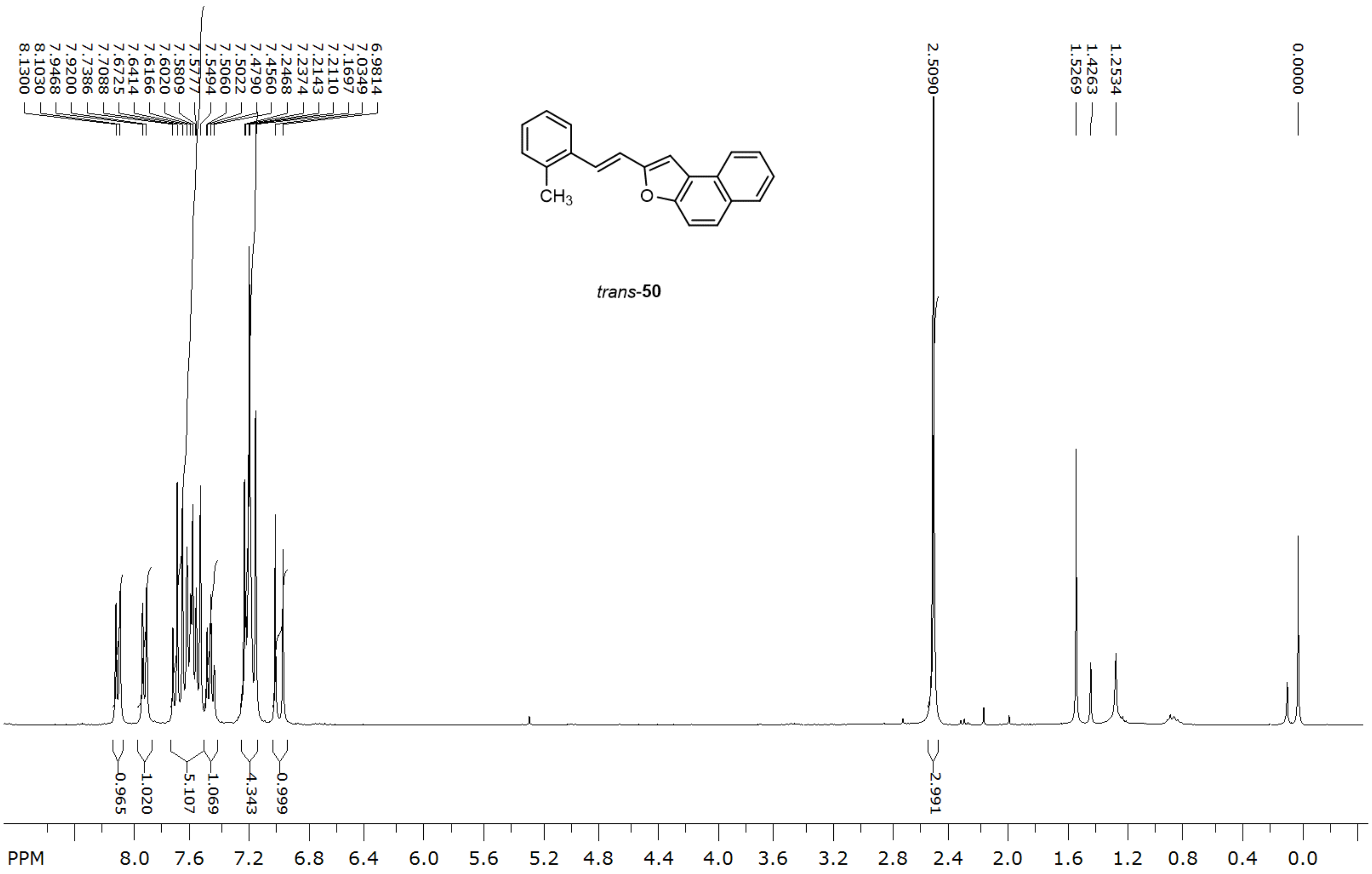

Figure S21. ^{1}H NMR ($CDCl_3$) spectrum of *trans*-**50**.

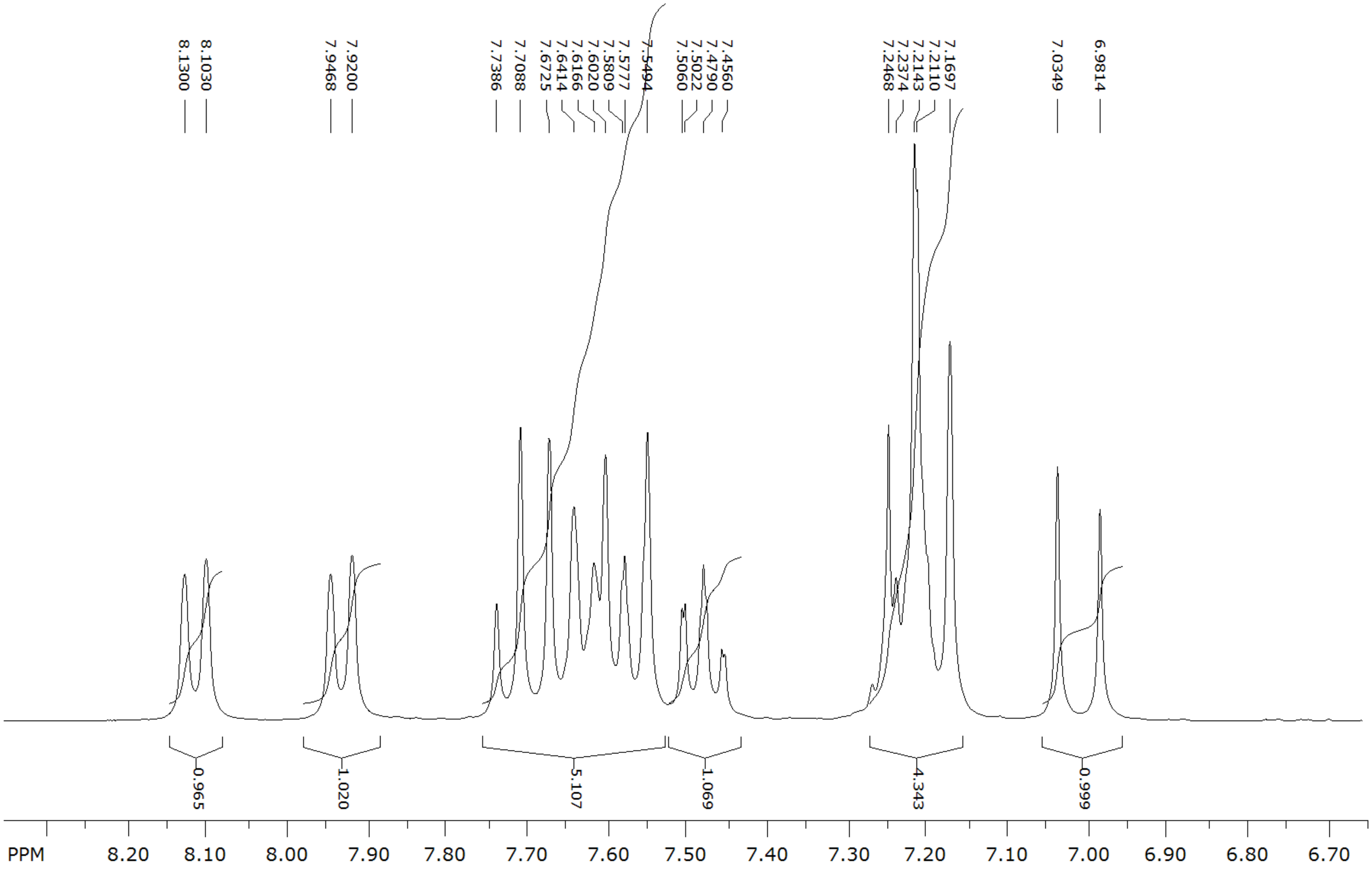

Figure S22. ^{1}H NMR ($CDCl_3$) spectrum of aromatic part of *trans*-**50**.

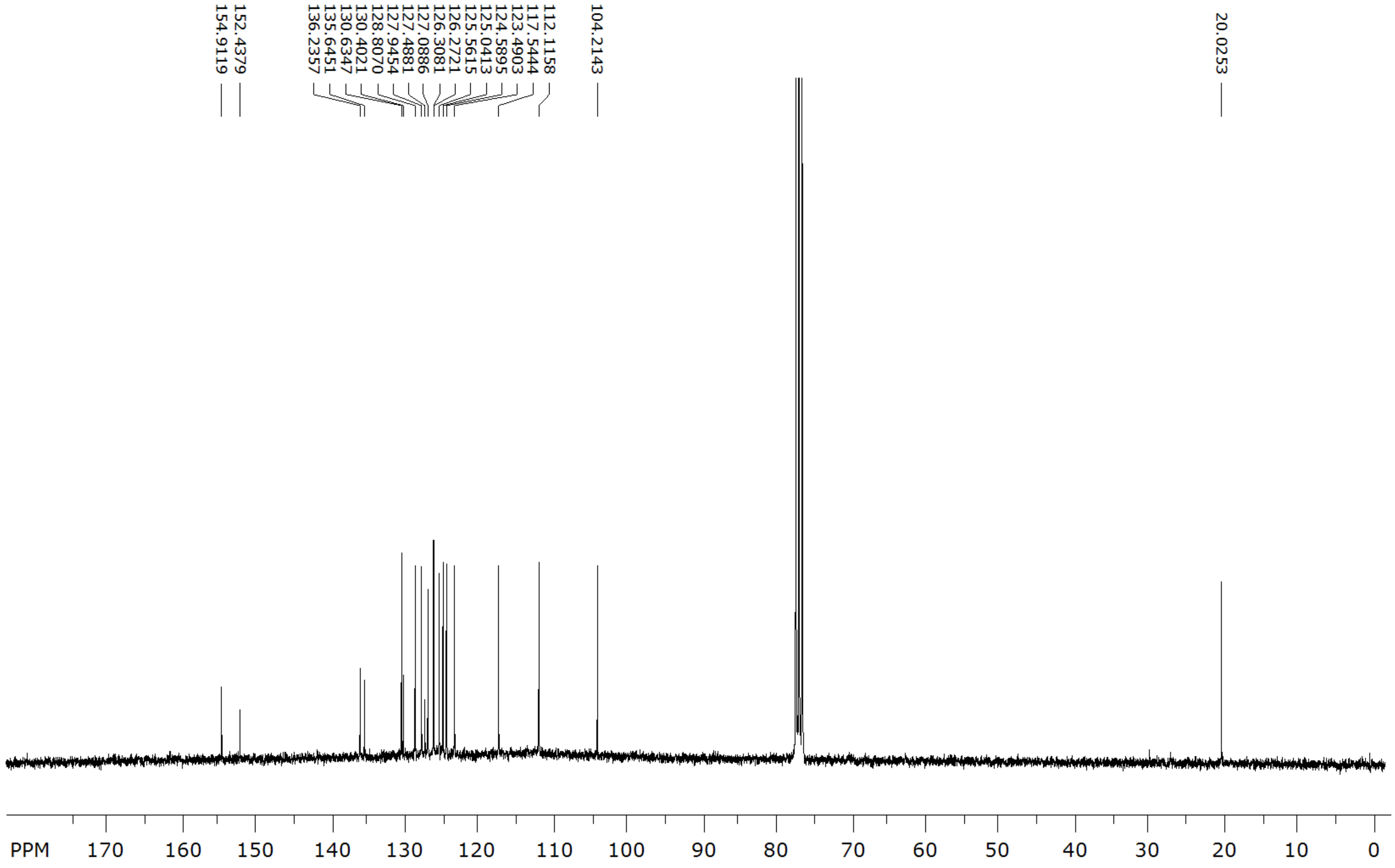

Figure S23. ^{13}C NMR ($CDCl_3$) spectrum of *trans*-**50**.

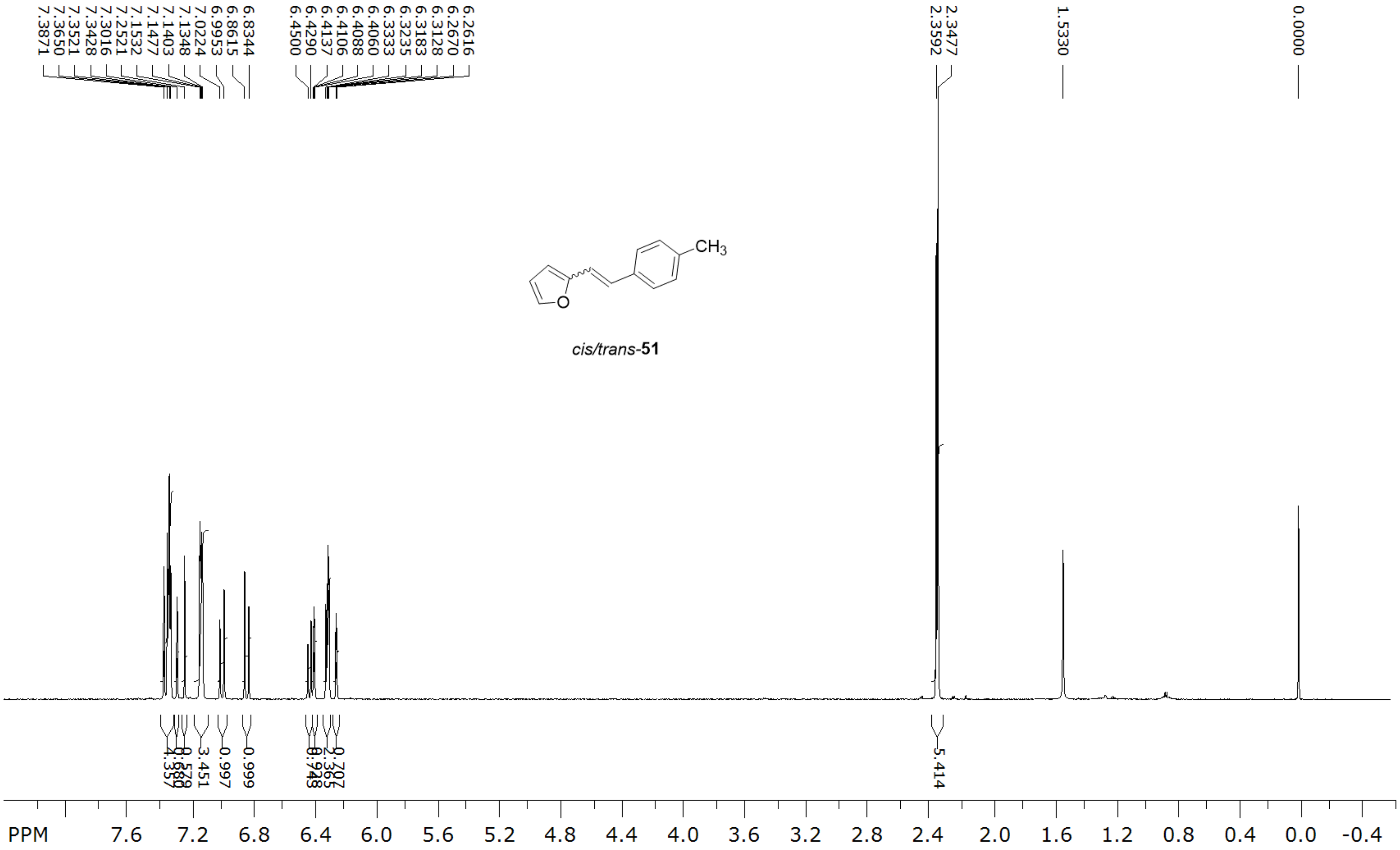

Figure S24. ^{1}H NMR ($CDCl_3$) spectrum of *cis/trans*-**51**.

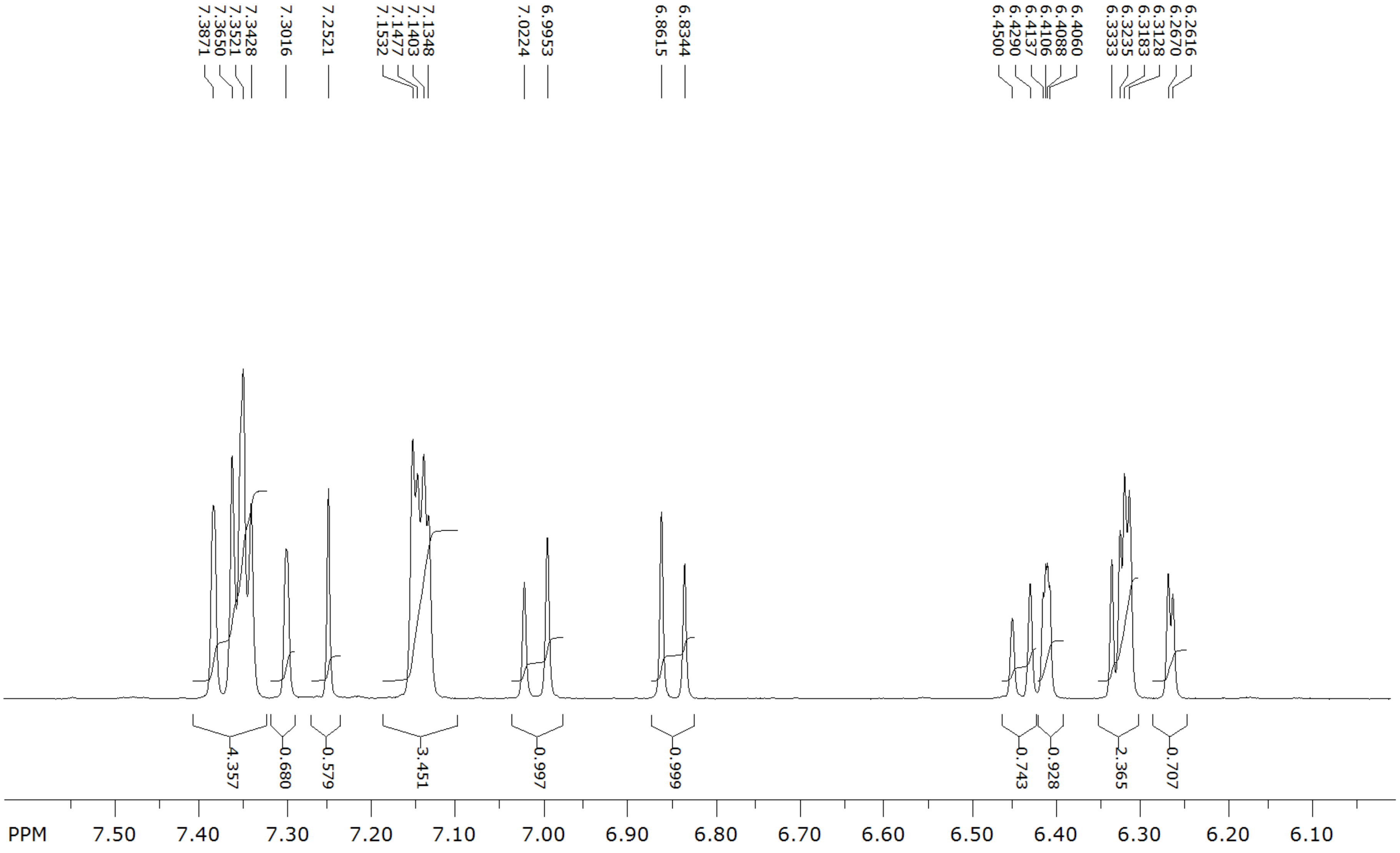

Figure S25. ^{1}H NMR ($CDCl_3$) spectrum of aromatic part of *cis/trans*-**51**.

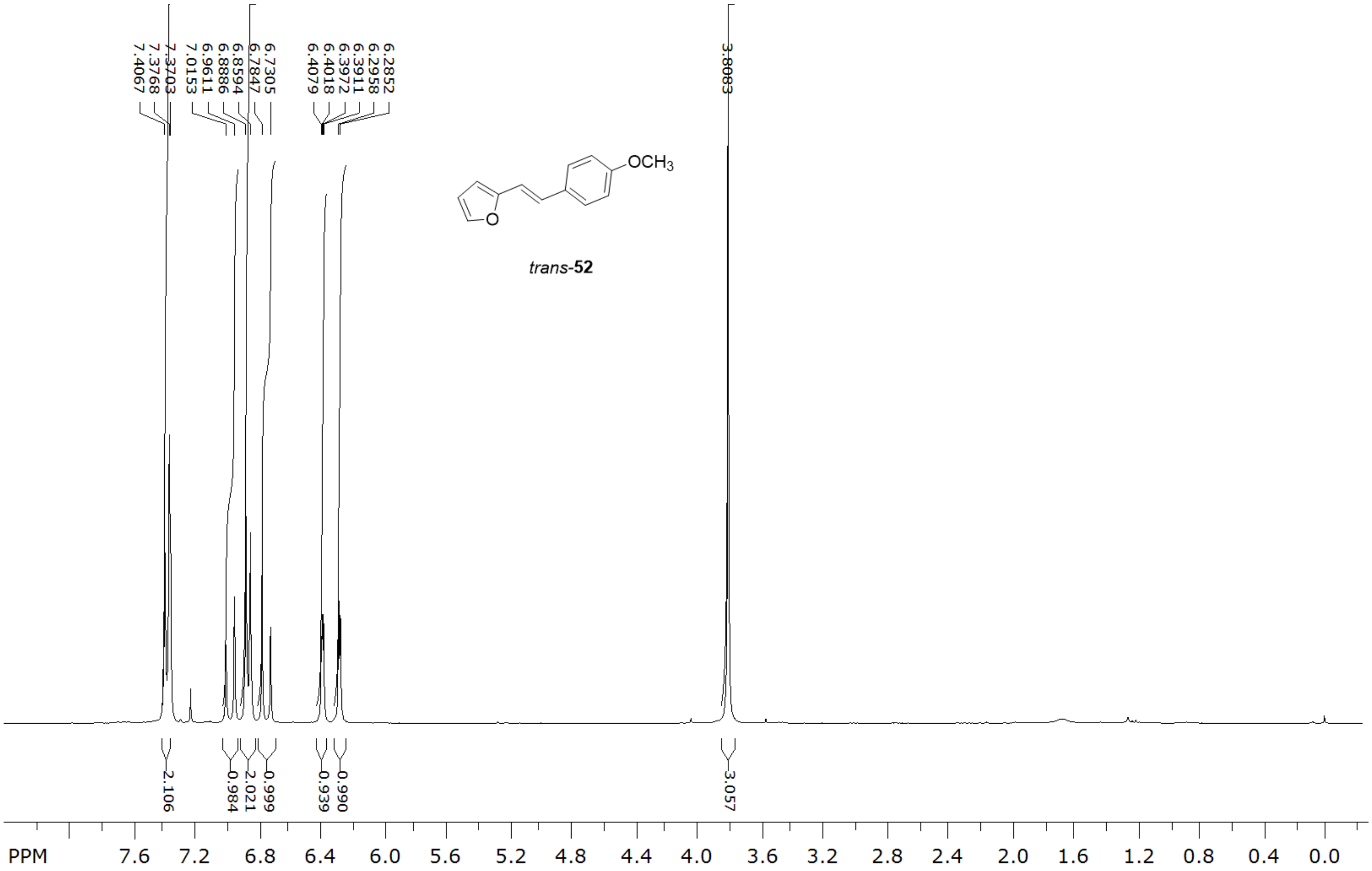

Figure S26. ^{1}H NMR ($CDCl_3$) spectrum of *trans*-**52**.

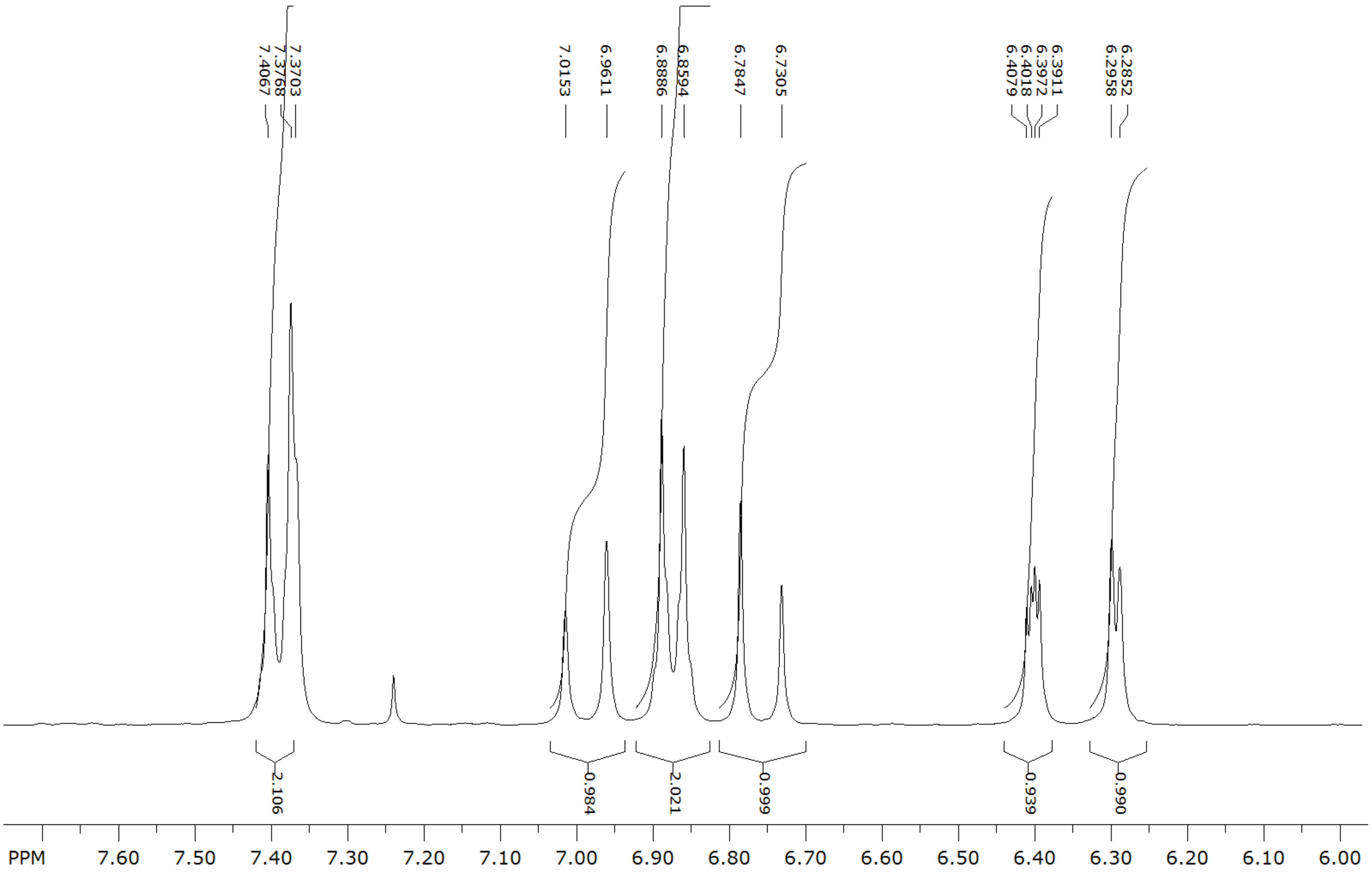

Figure S27. ^{1}H NMR ($CDCl_3$) spectrum of aromatic part of *trans*-**52**.

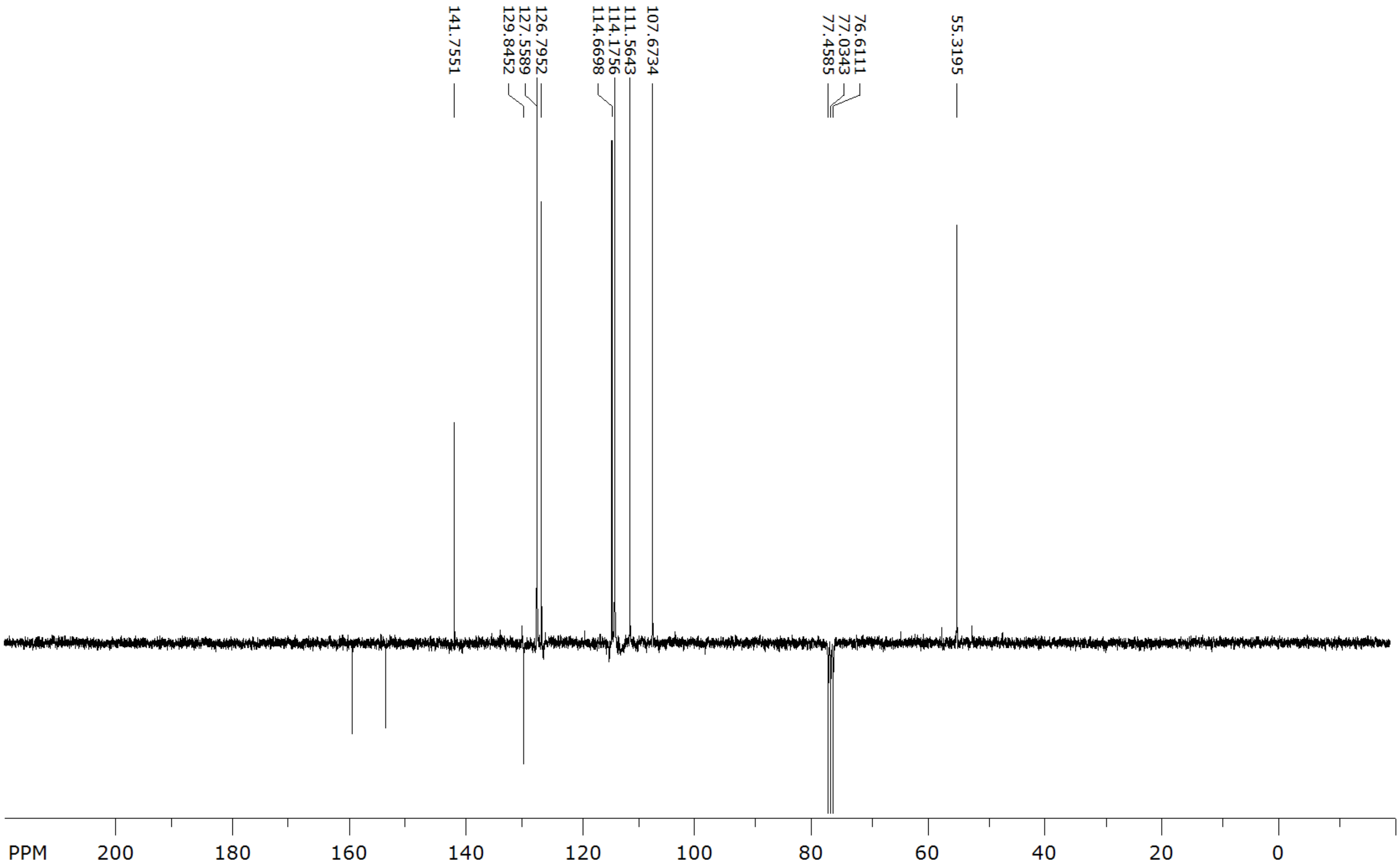

Figure S28. ^{13}C NMR ($CDCl_3$) spectrum of *trans*-**52**.

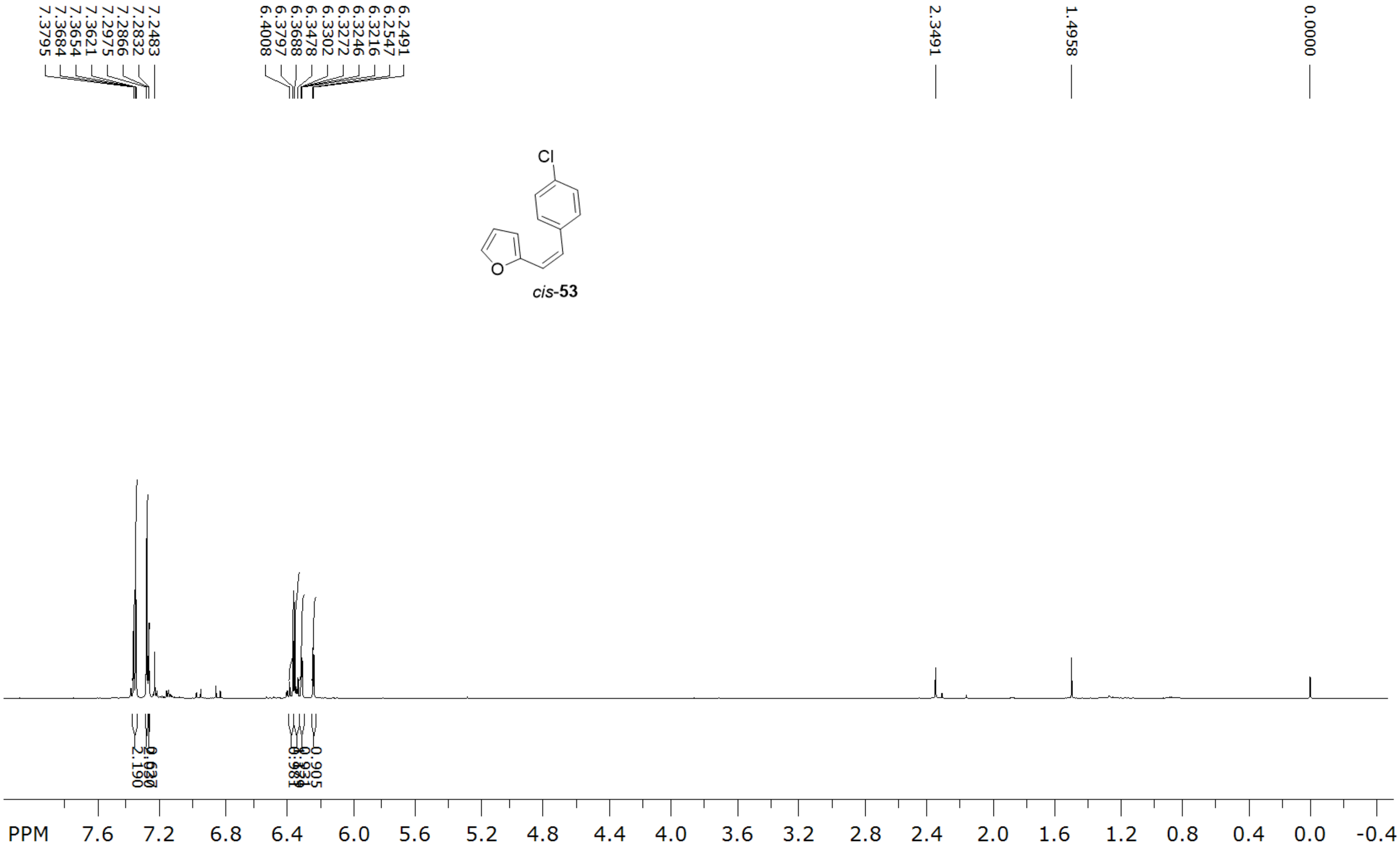

Figure S29. ^{1}H NMR ($CDCl_3$) spectrum of *cis*-**53**.

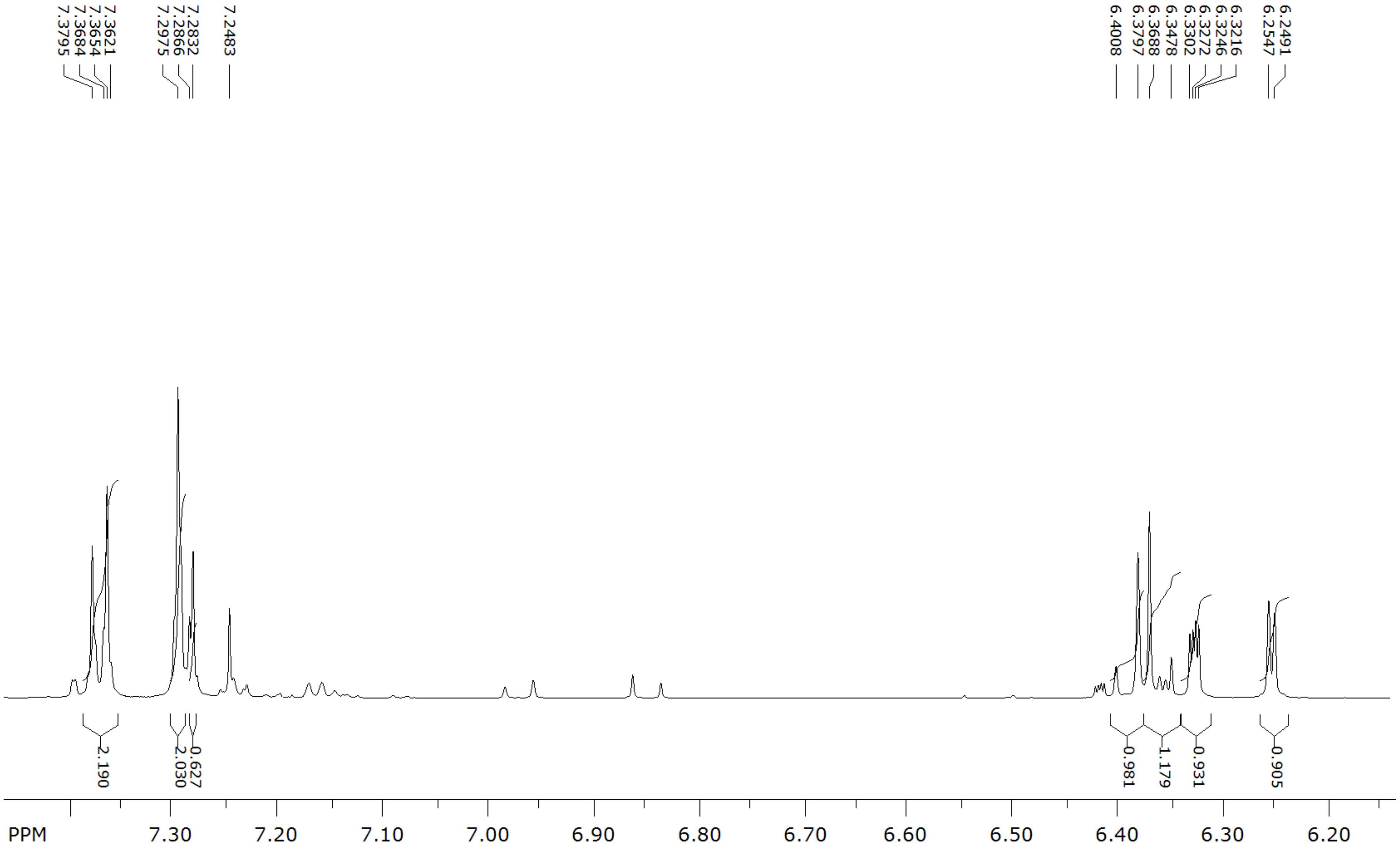

Figure S30. ^{1}H NMR ($CDCl_3$) spectrum of aromatic part of *cis*-**53**.

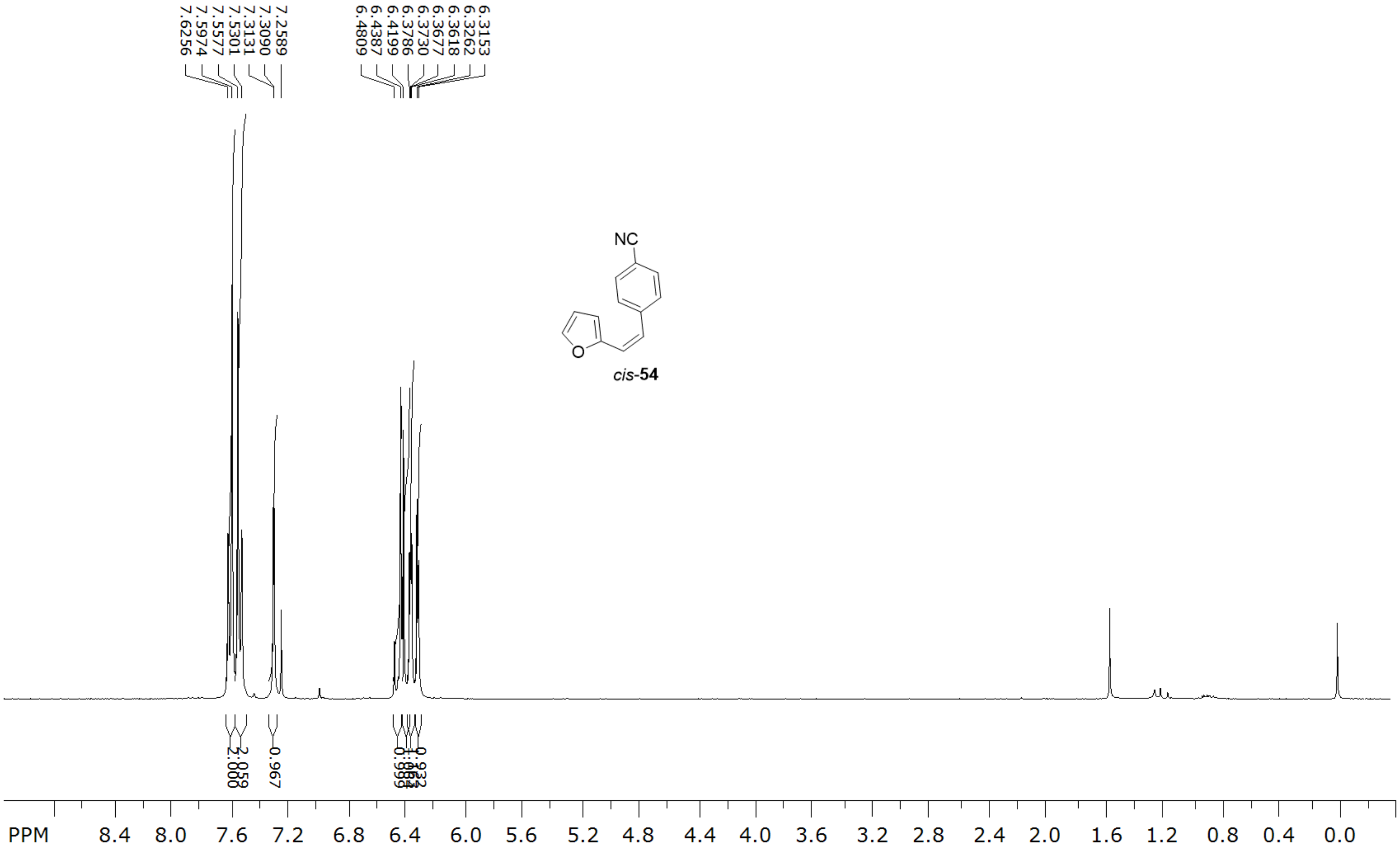

Figure S31. ^{1}H NMR ($CDCl_3$) spectrum of *cis*-**54**.

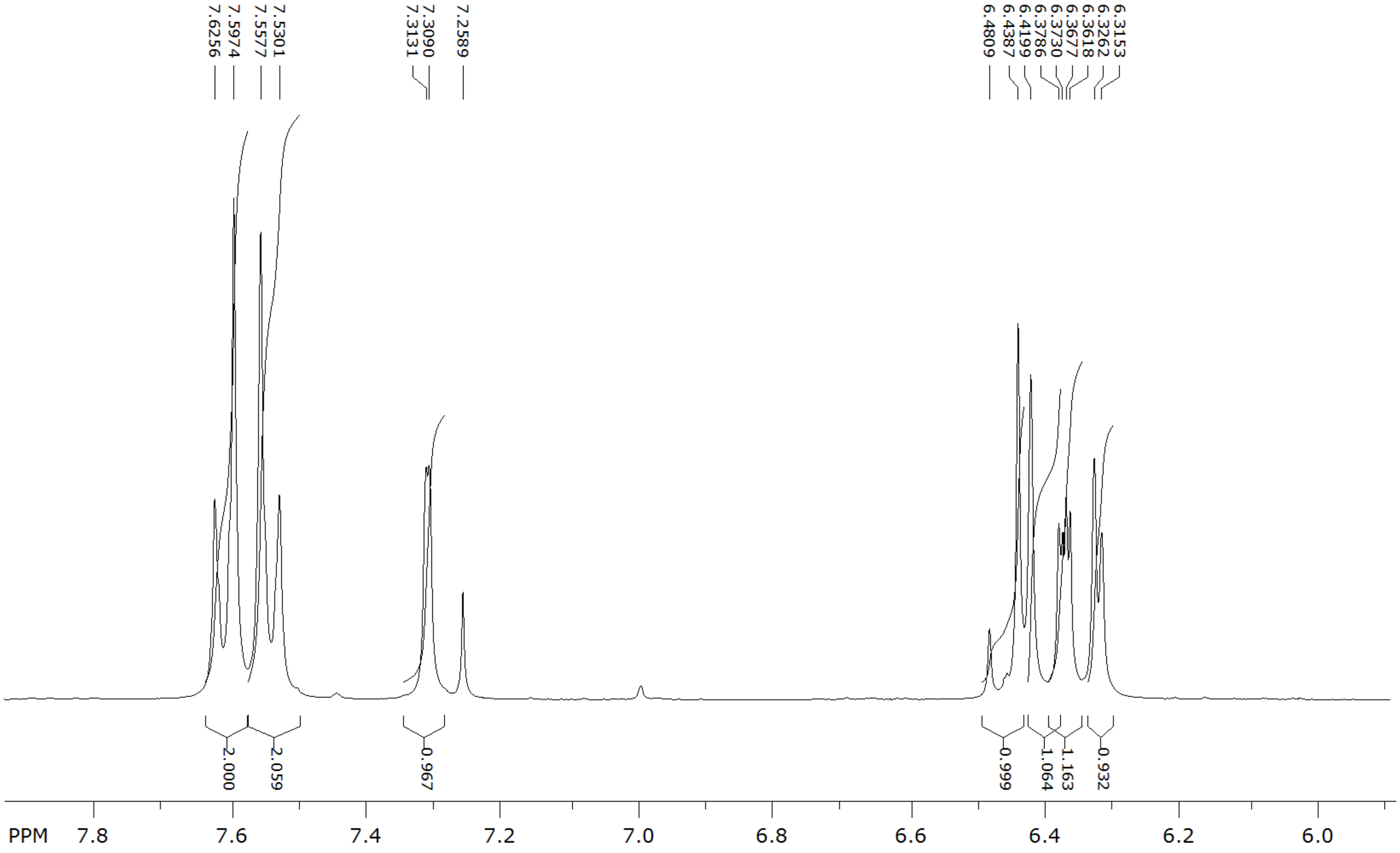

Figure S32. ^{1}H NMR ($CDCl_3$) spectrum of aromatic part of *cis*-**54**.

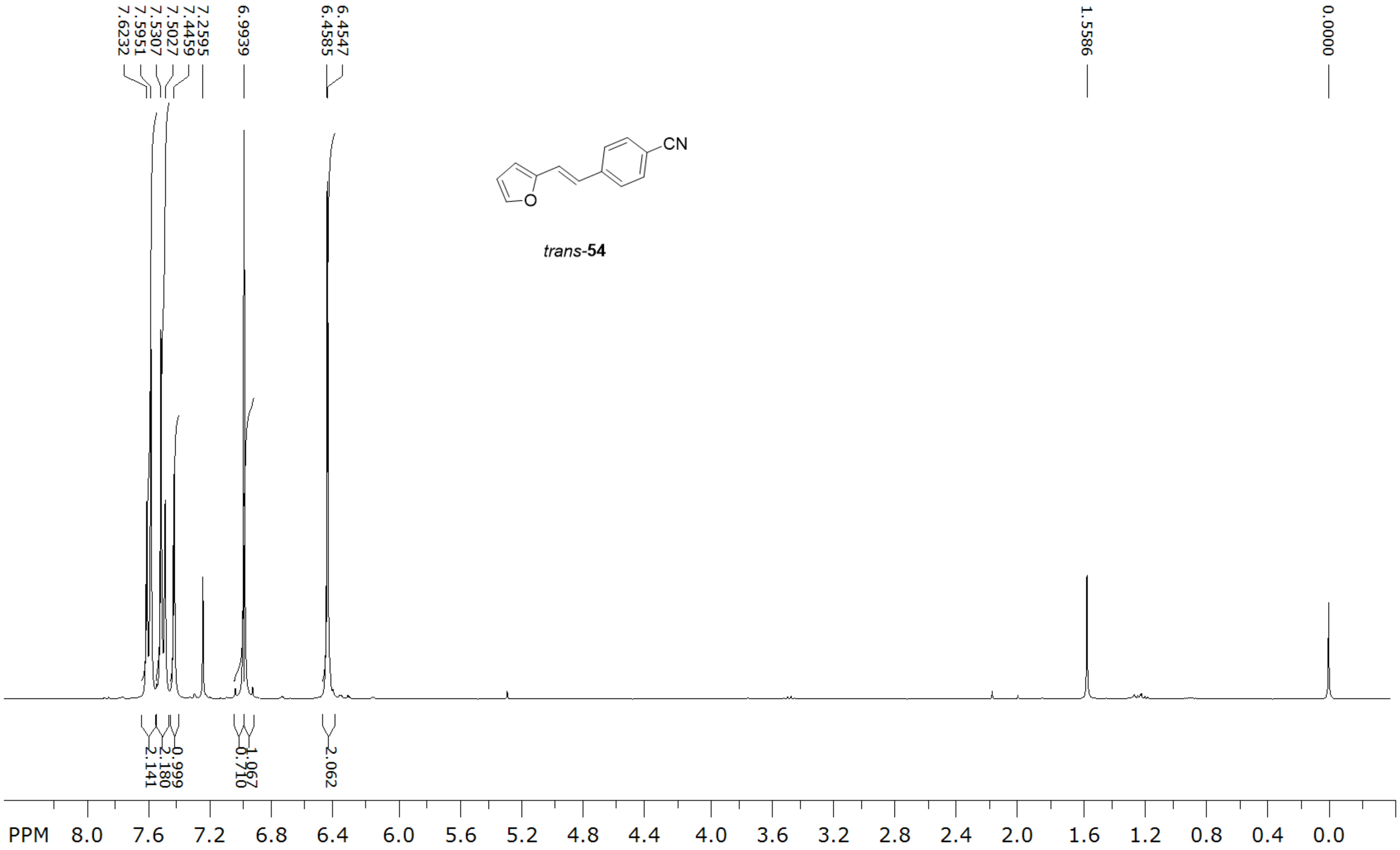

Figure S33. ^{1}H NMR ($CDCl_3$) spectrum of *cis*-**54**.

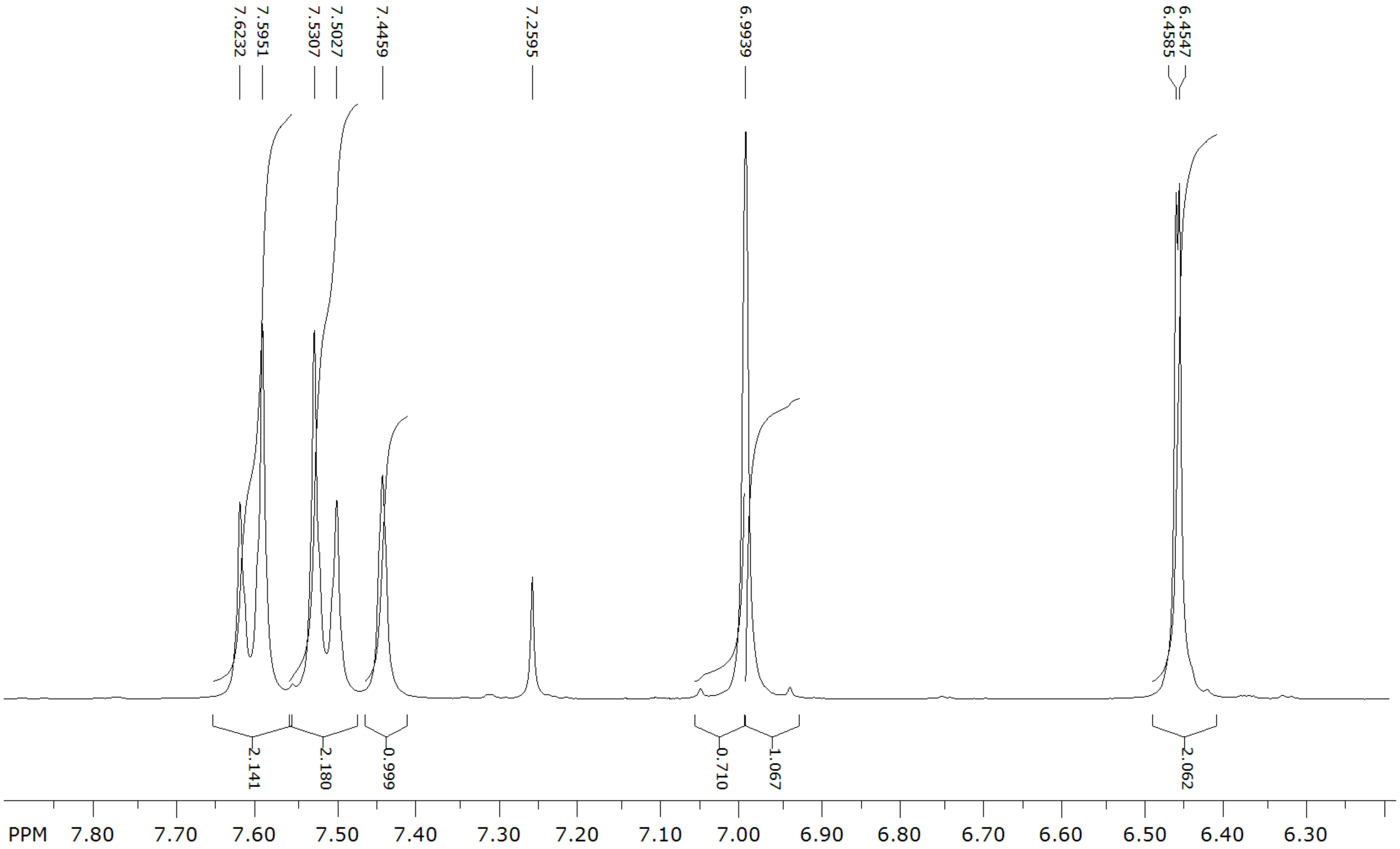

Figure S34. ^{1}H NMR ($CDCl_3$) spectrum of aromatic part of *cis*-**54**.

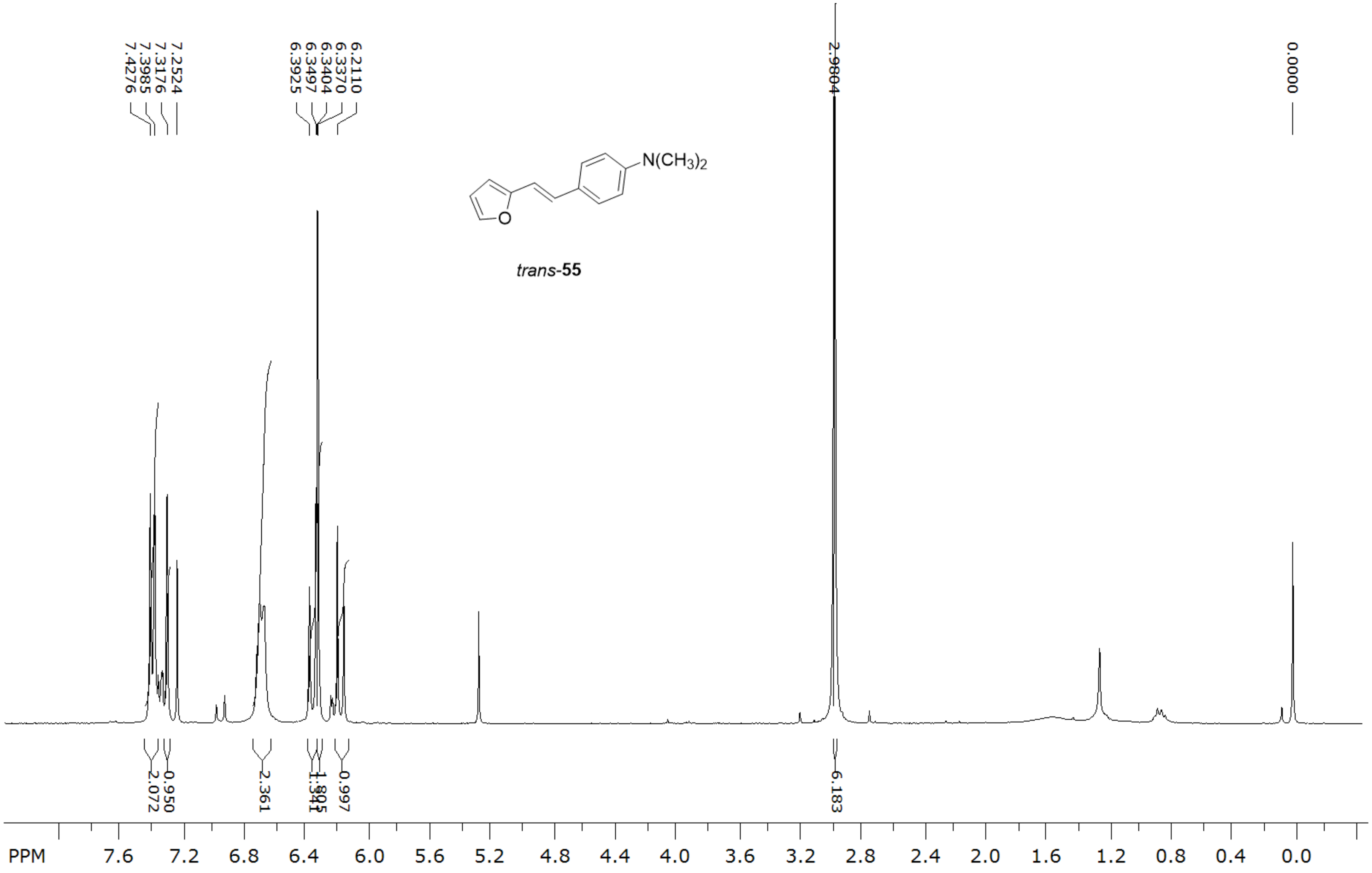

Figure S35. ^{1}H NMR ($CDCl_3$) spectrum of *trans*-**55**.

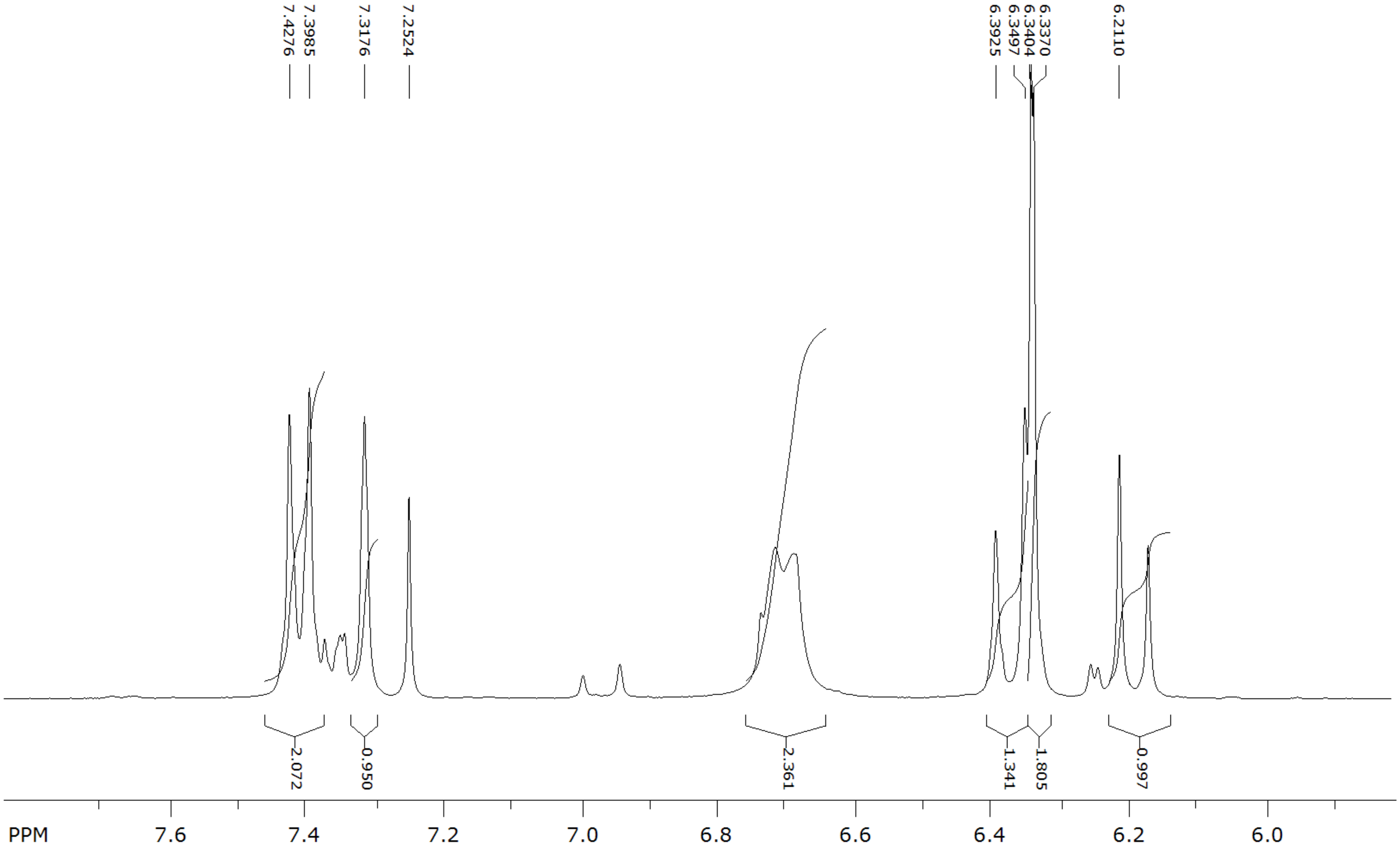

Figure S36. ^{1}H NMR ($CDCl_3$) spectrum of aromatic part of *trans*-**55**.

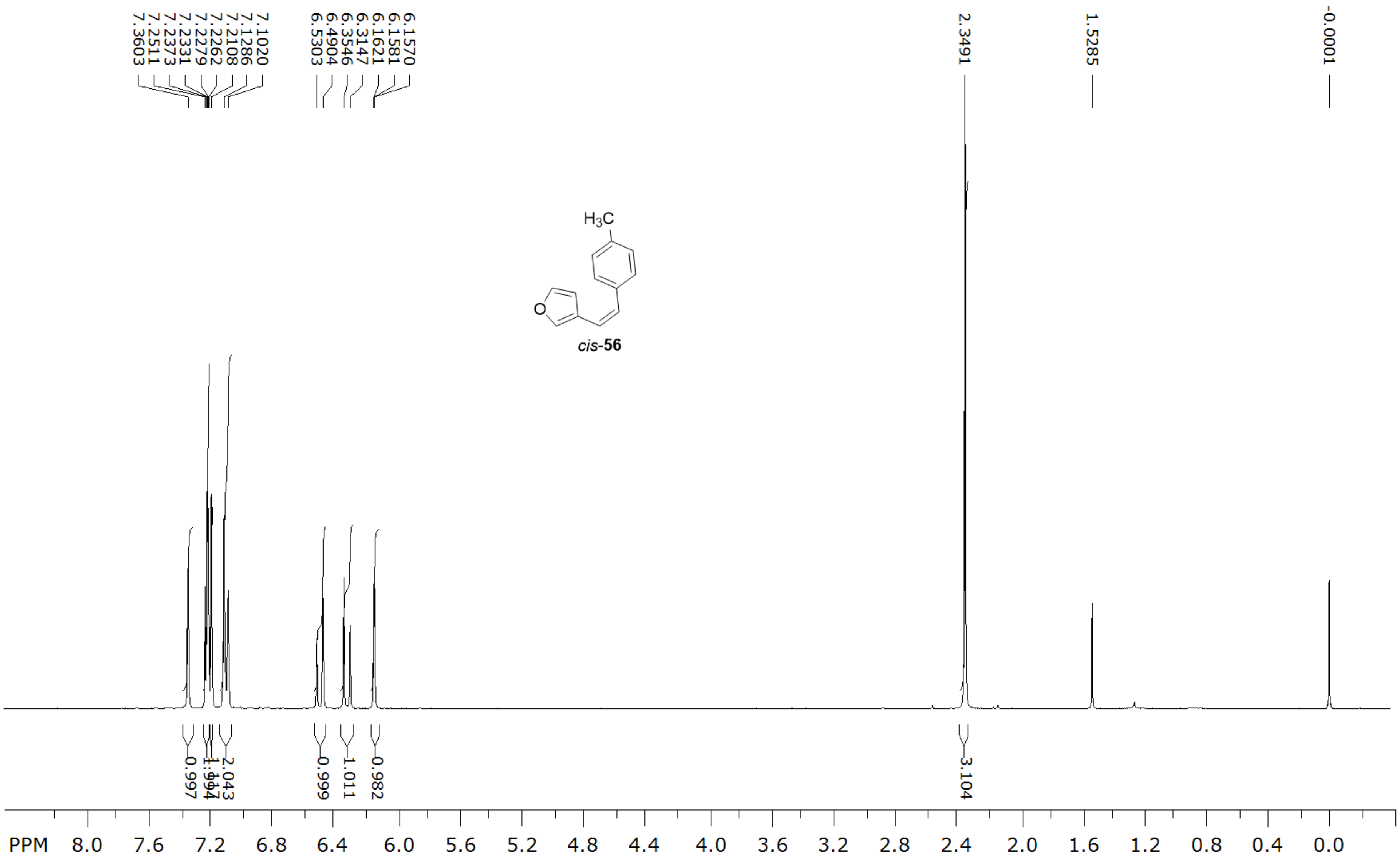

Figure S37. ^{1}H NMR ($CDCl_3$) spectrum of *cis*-**56**.

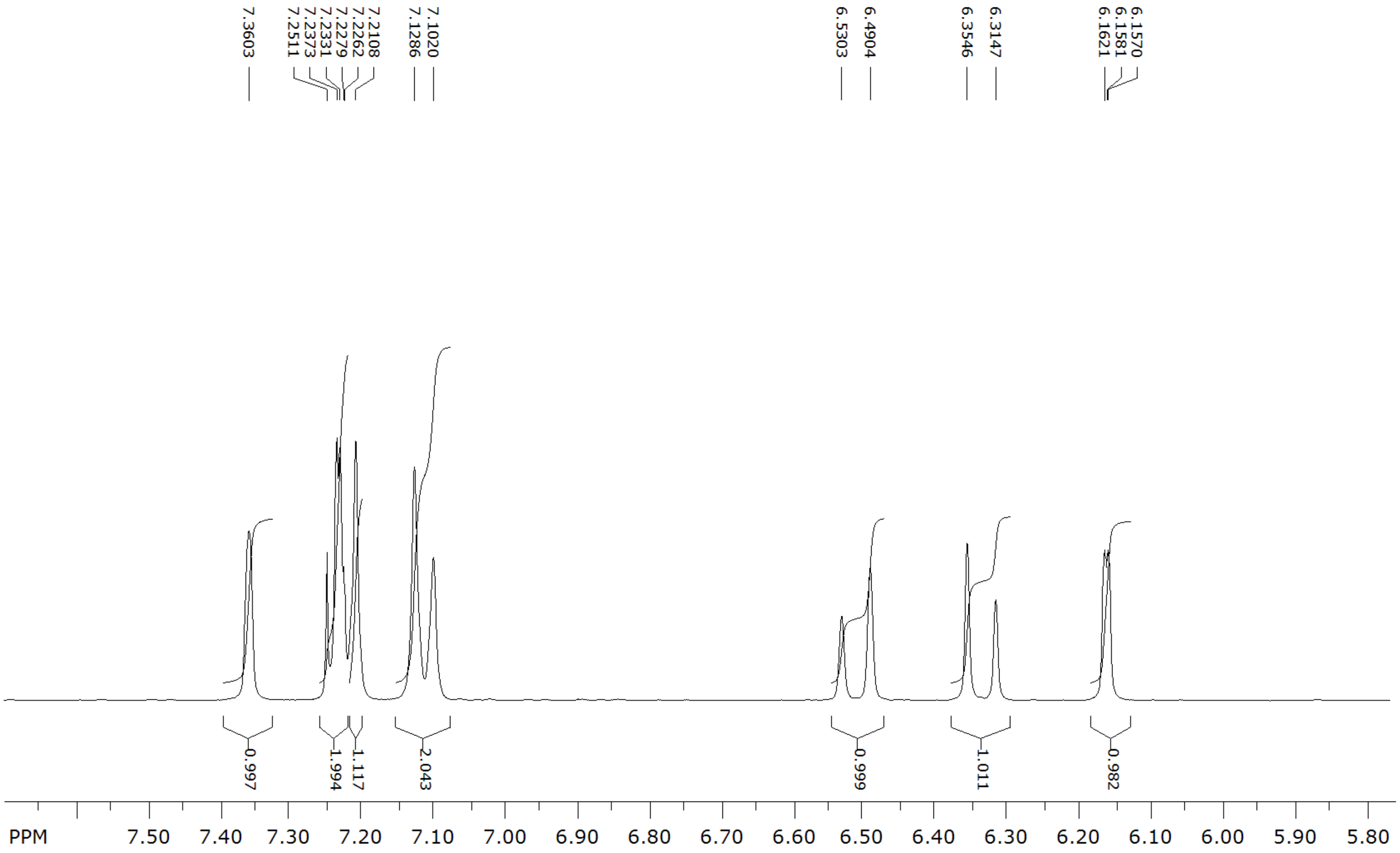

Figure S38. ^{1}H NMR ($CDCl_3$) spectrum of aromatic part of *cis*-**56**.

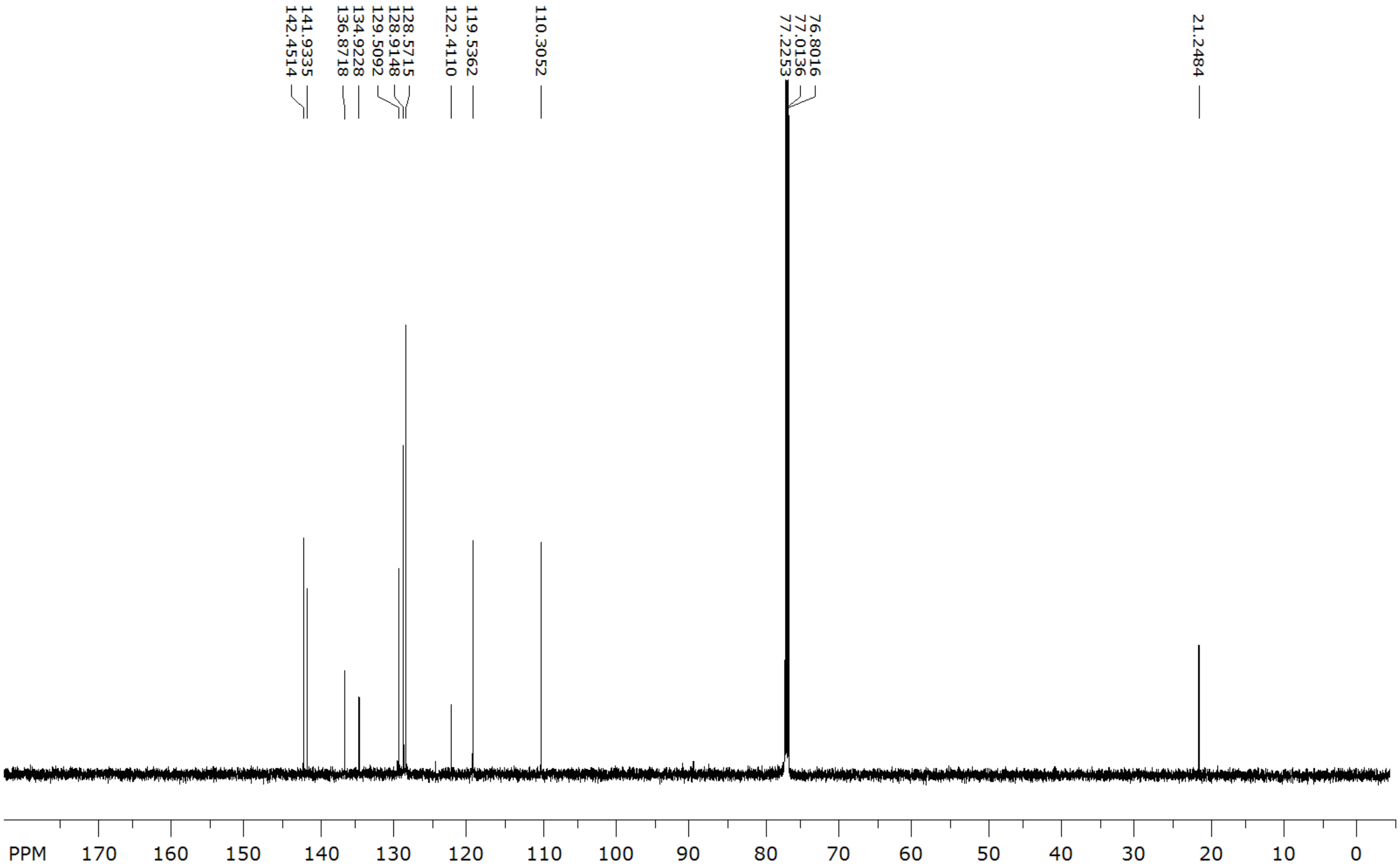

Figure S39. ^{13}C NMR ($CDCl_3$) spectrum of *cis*-**56**.

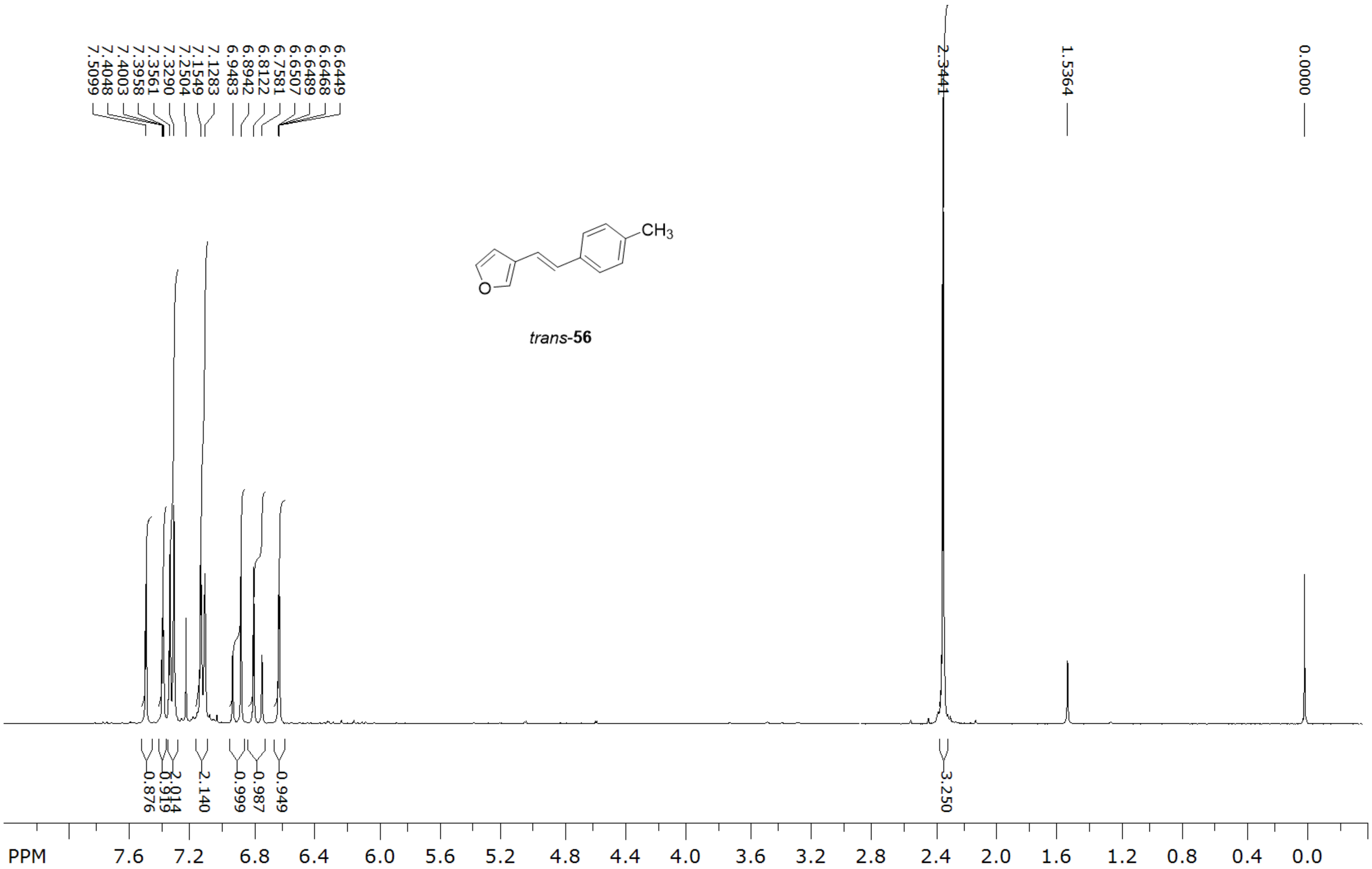

Figure S40. ^{1}H NMR ($CDCl_3$) spectrum of *trans*-**56**.

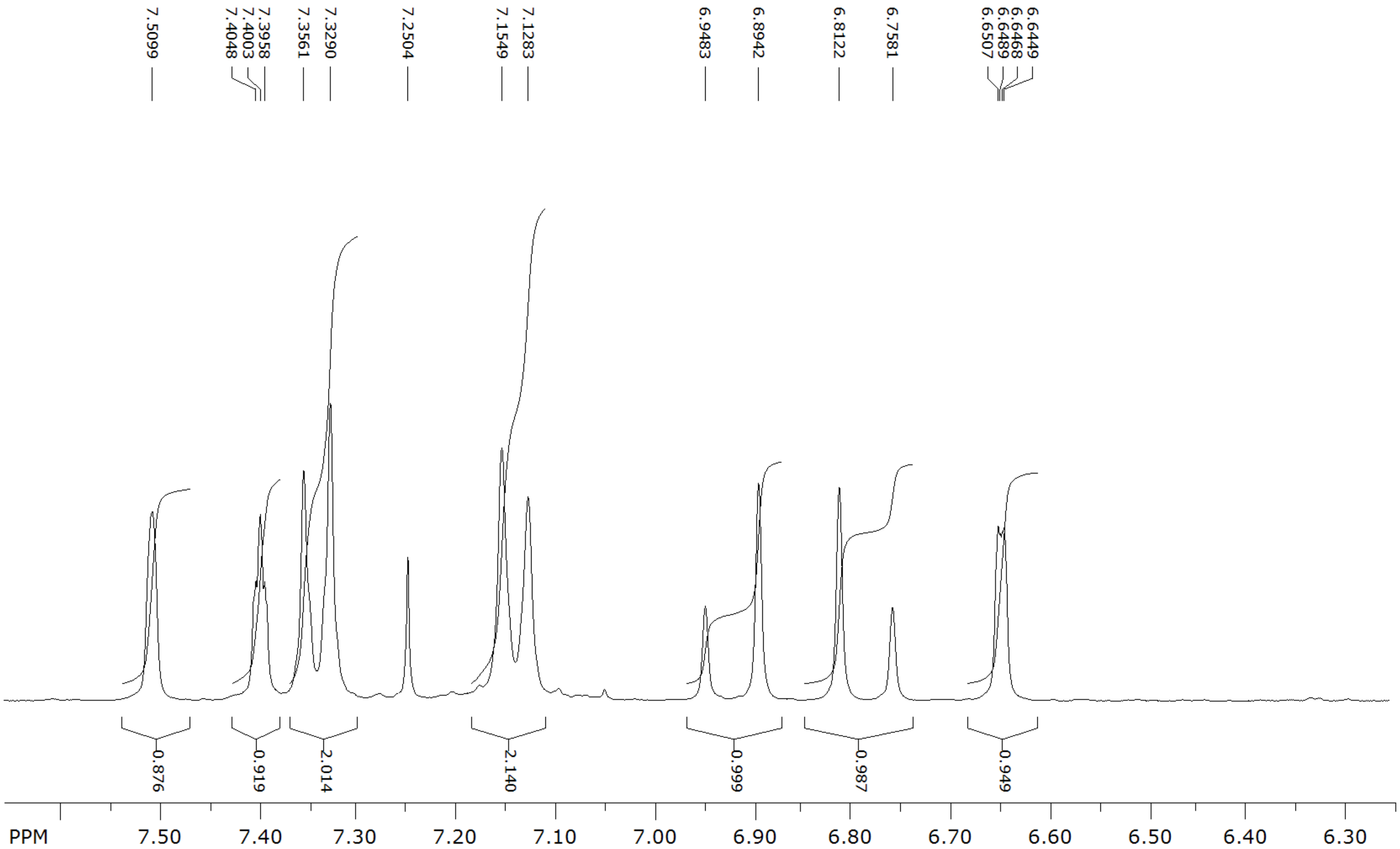

Figure S41. ^{1}H NMR ($CDCl_3$) spectrum of aromatic part of *trans*-**56**.

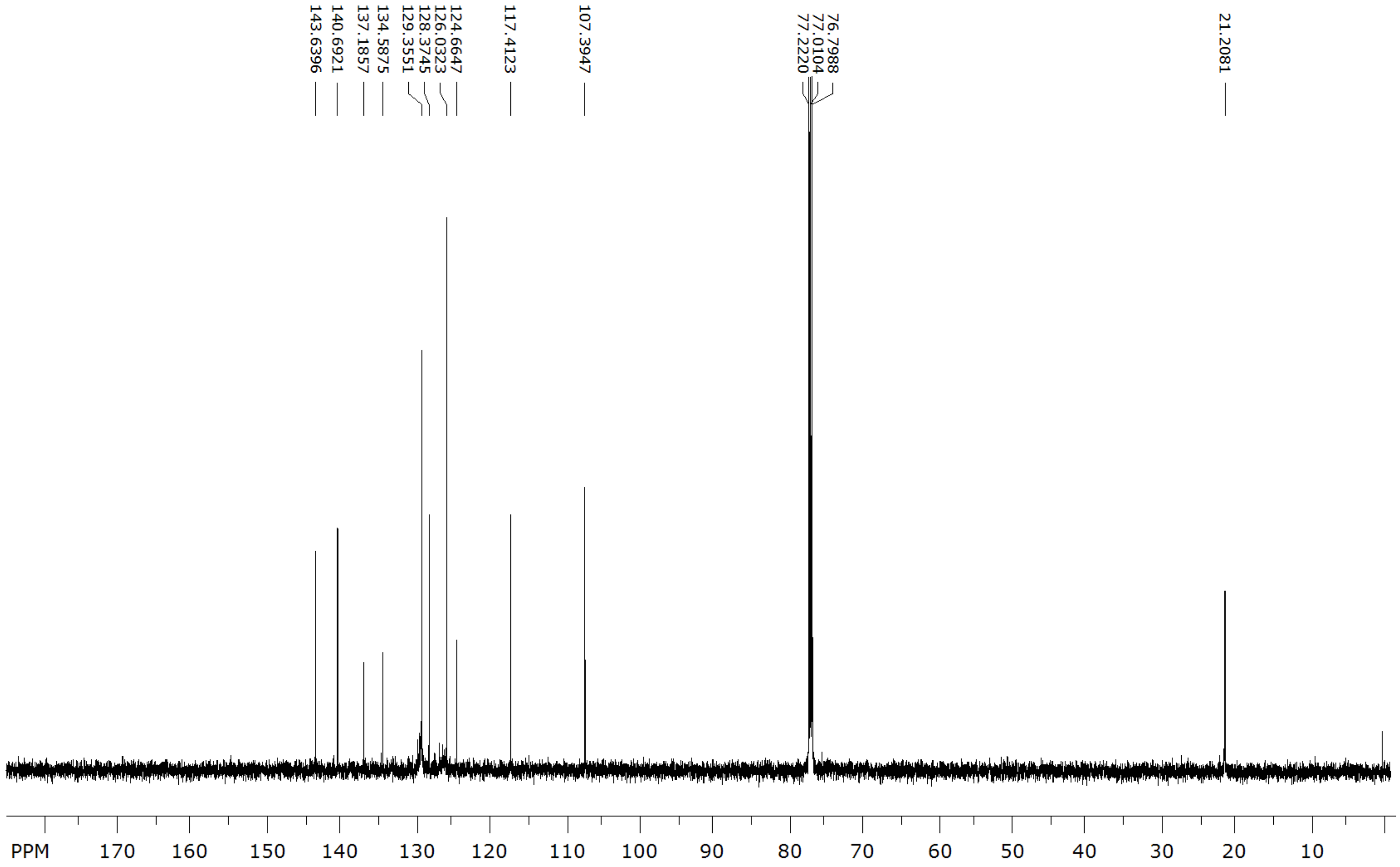

Figure S42. ^{13}C NMR ($CDCl_3$) spectrum of *trans*-**56**.

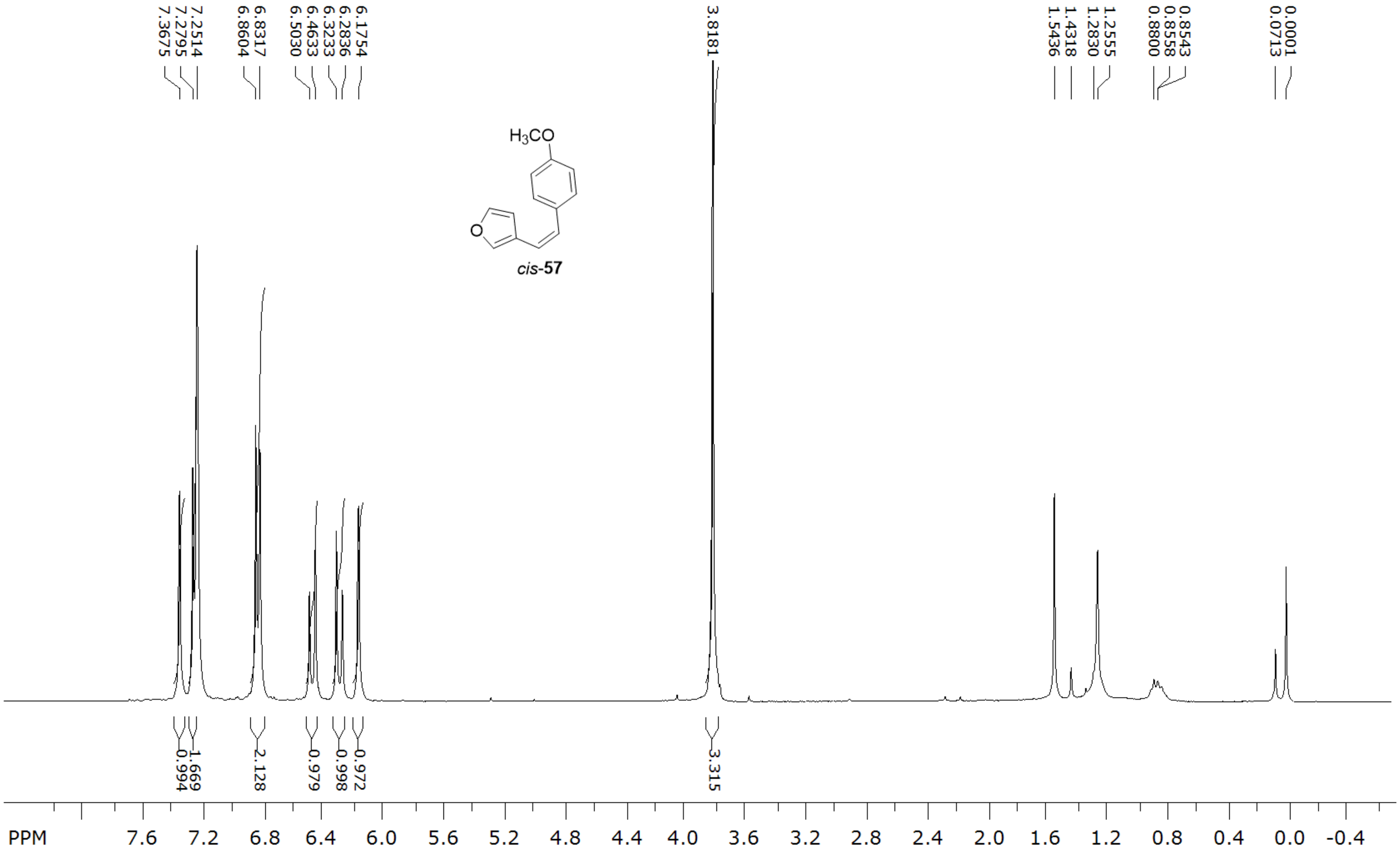

Figure S43. ^{1}H NMR ($CDCl_3$) spectrum of *cis*-**57**.

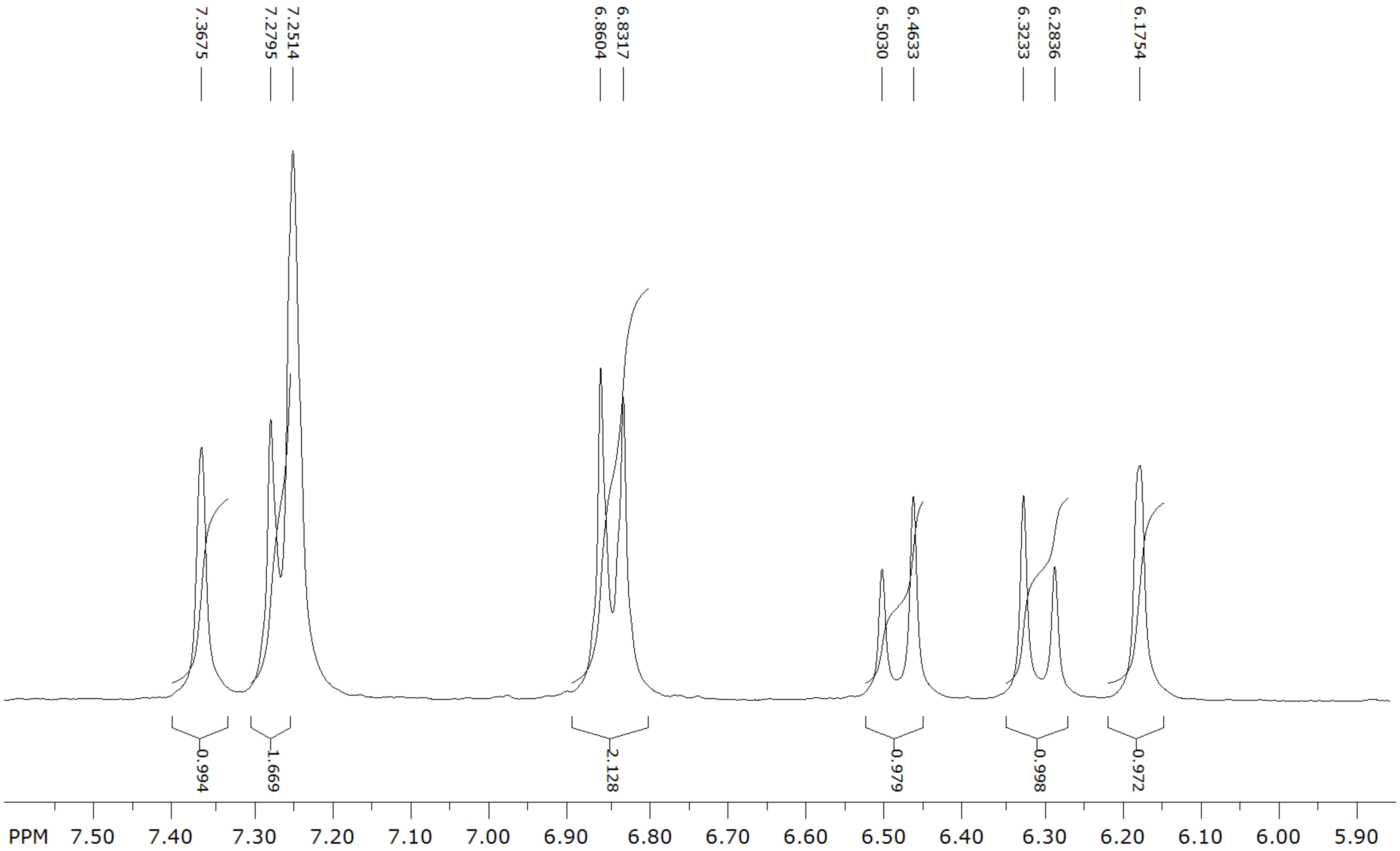

Figure S44. ^{1}H NMR ($CDCl_3$) spectrum of aromatic part of *cis*-**57**.

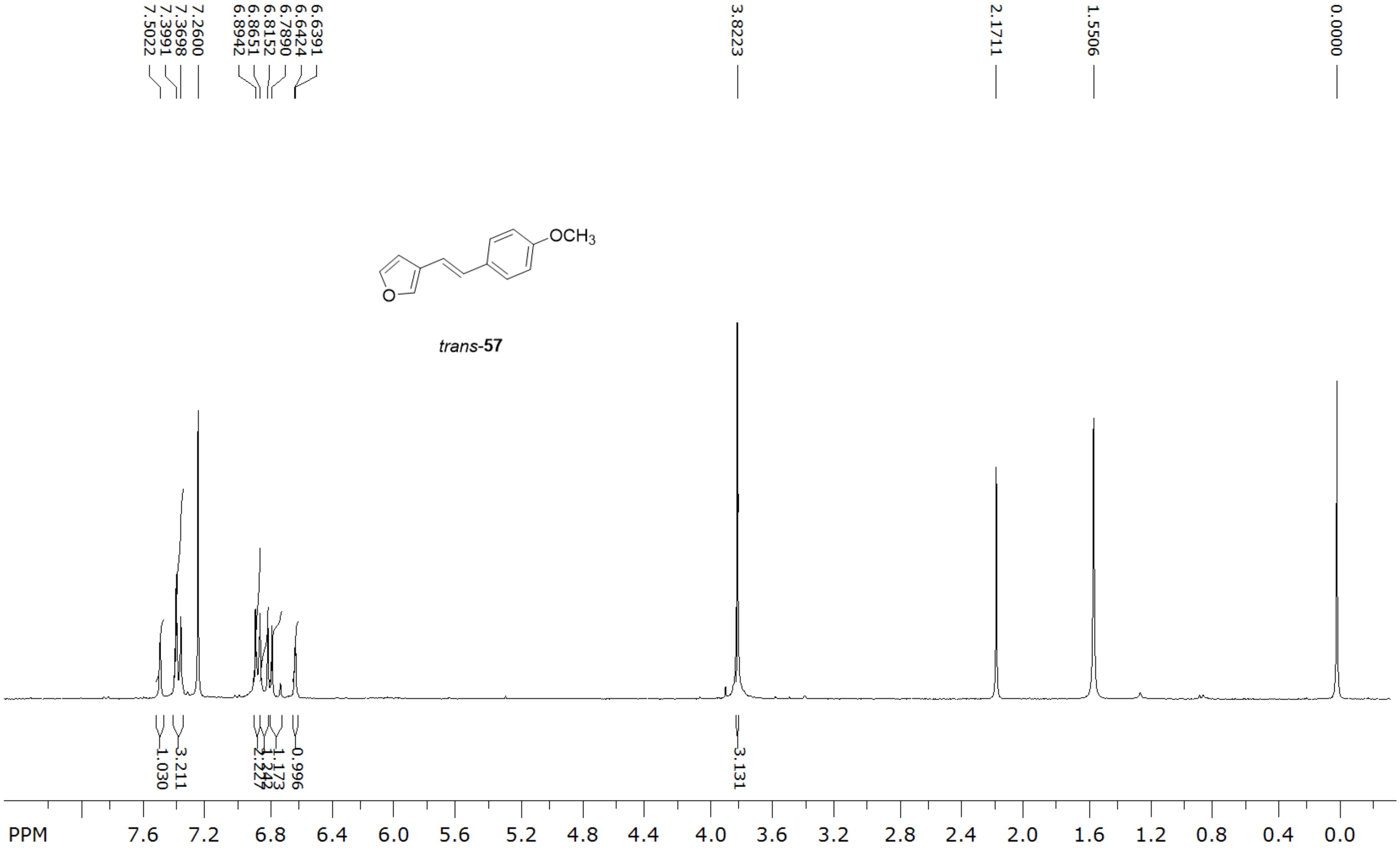

Figure S45. ^{1}H NMR ($CDCl_3$) spectrum of *trans*-**57**.

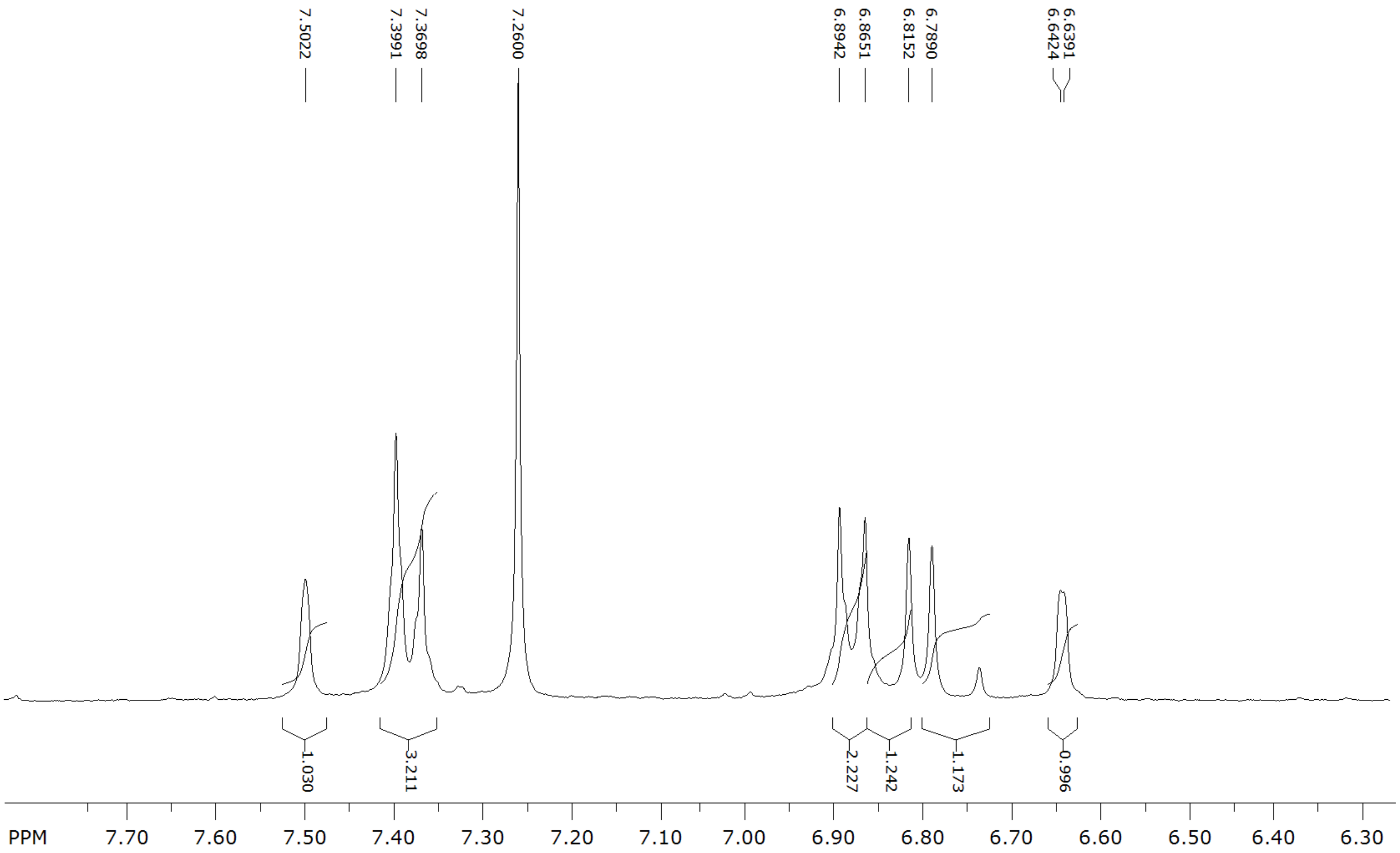

Figure S46. ^{1}H NMR ($CDCl_3$) spectrum of aromatic part of *trans*-**57**.

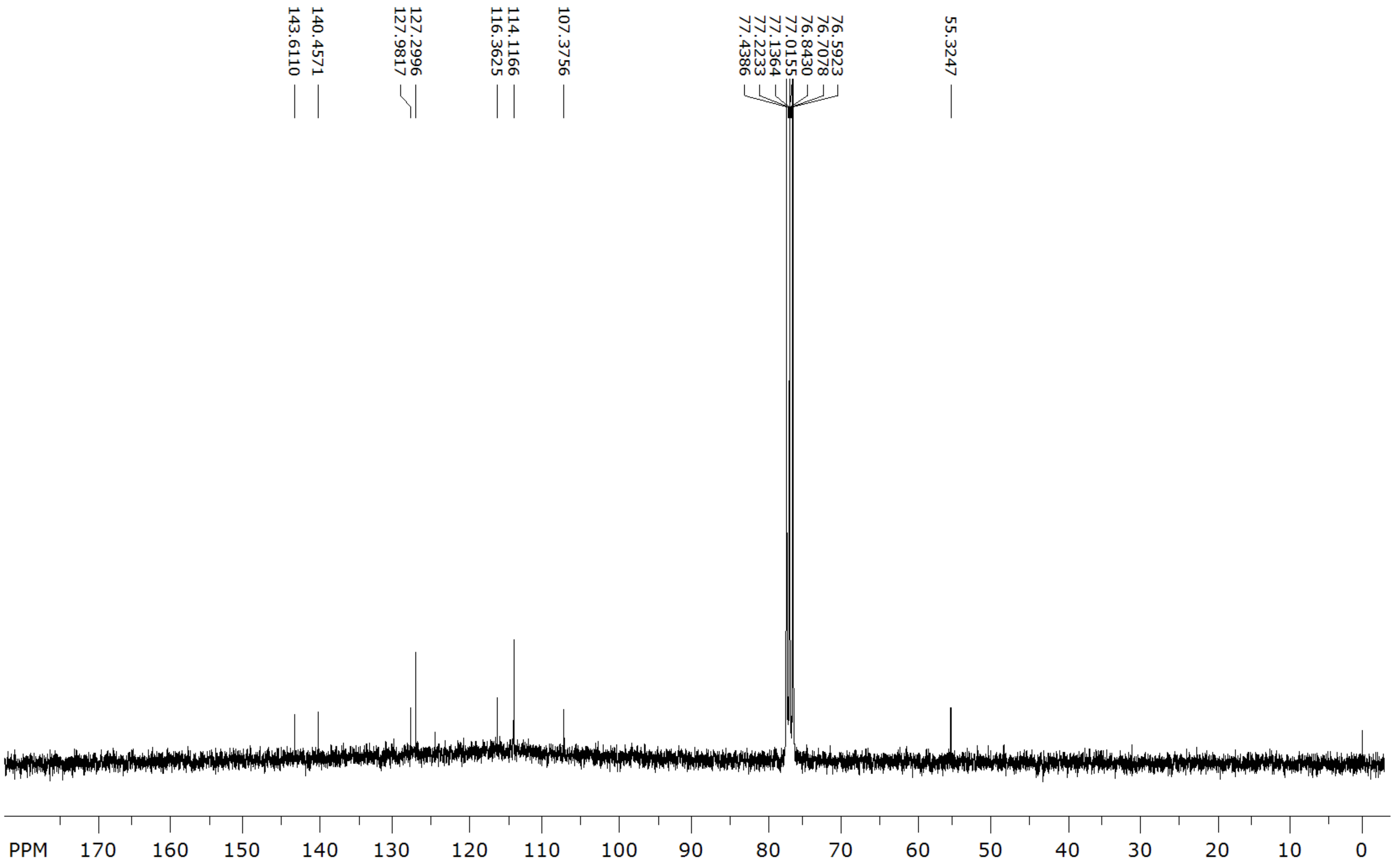

Figure S47. ^{13}C NMR ($CDCl_3$) spectrum of *trans*-**57**.

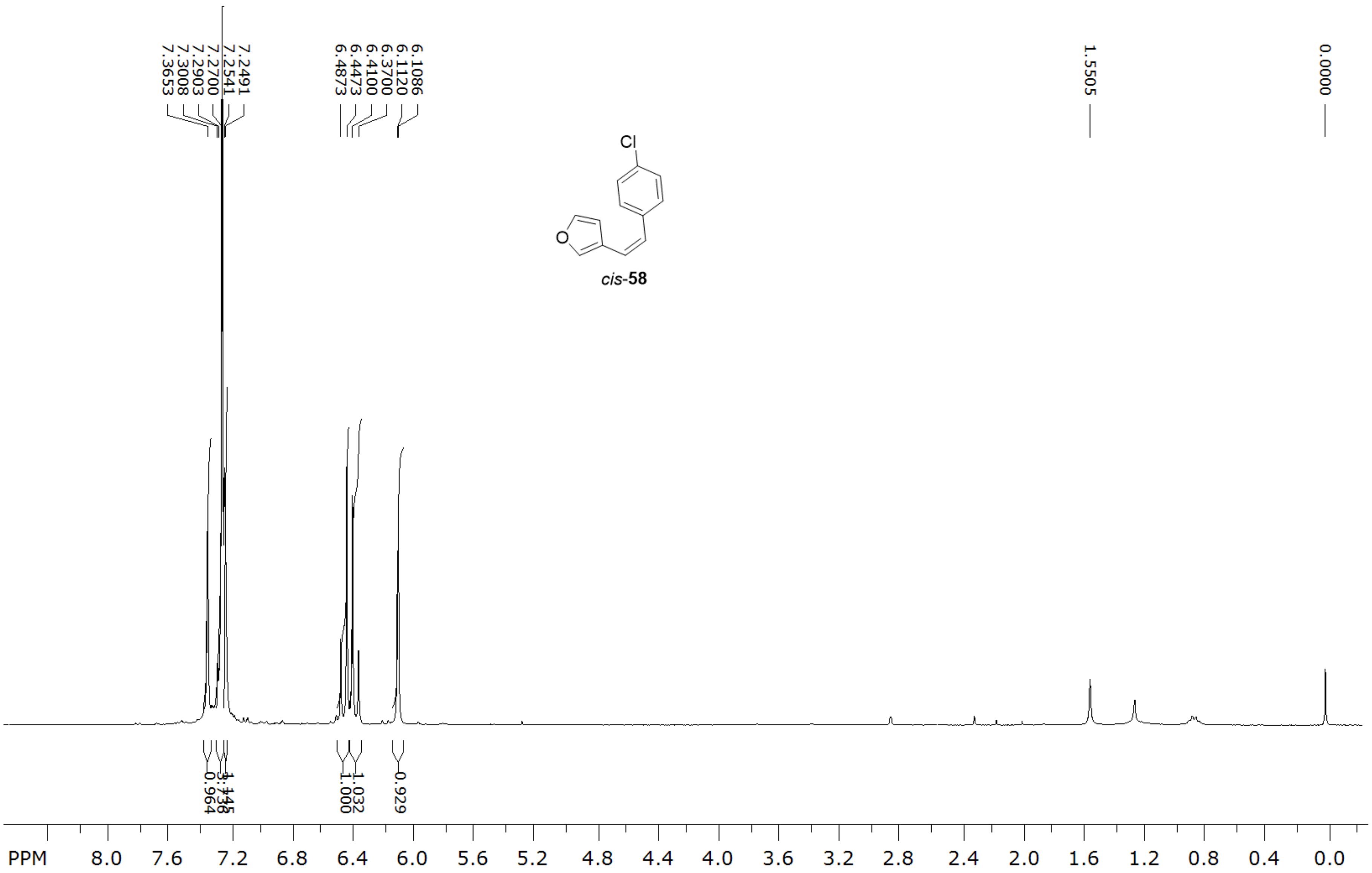

Figure S48. ^{1}H NMR ($CDCl_3$) spectrum of *cis*-**58**.

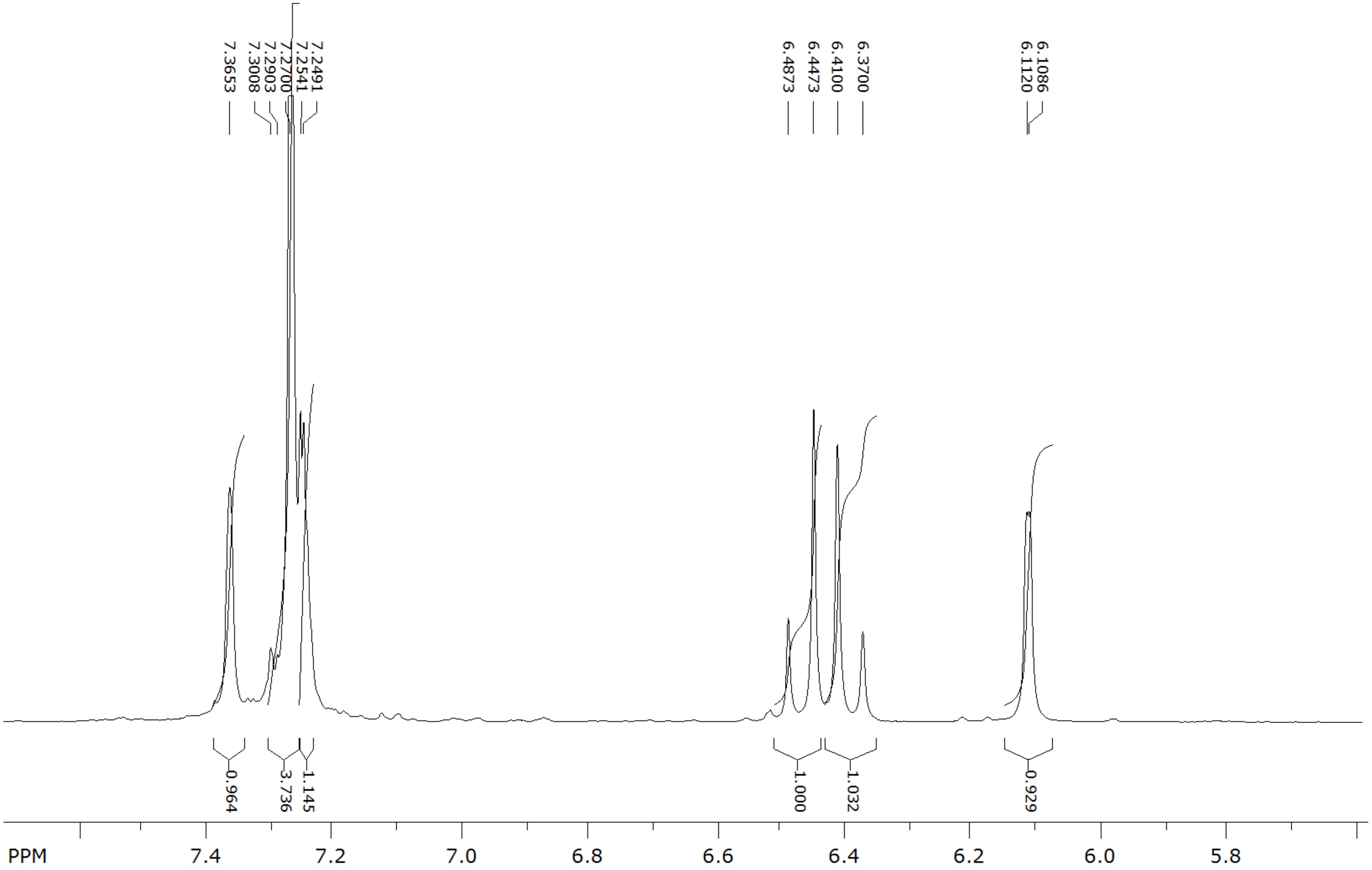

Figure S49. ^{1}H NMR ($CDCl_3$) spectrum of aromatic part of *cis*-**58**.

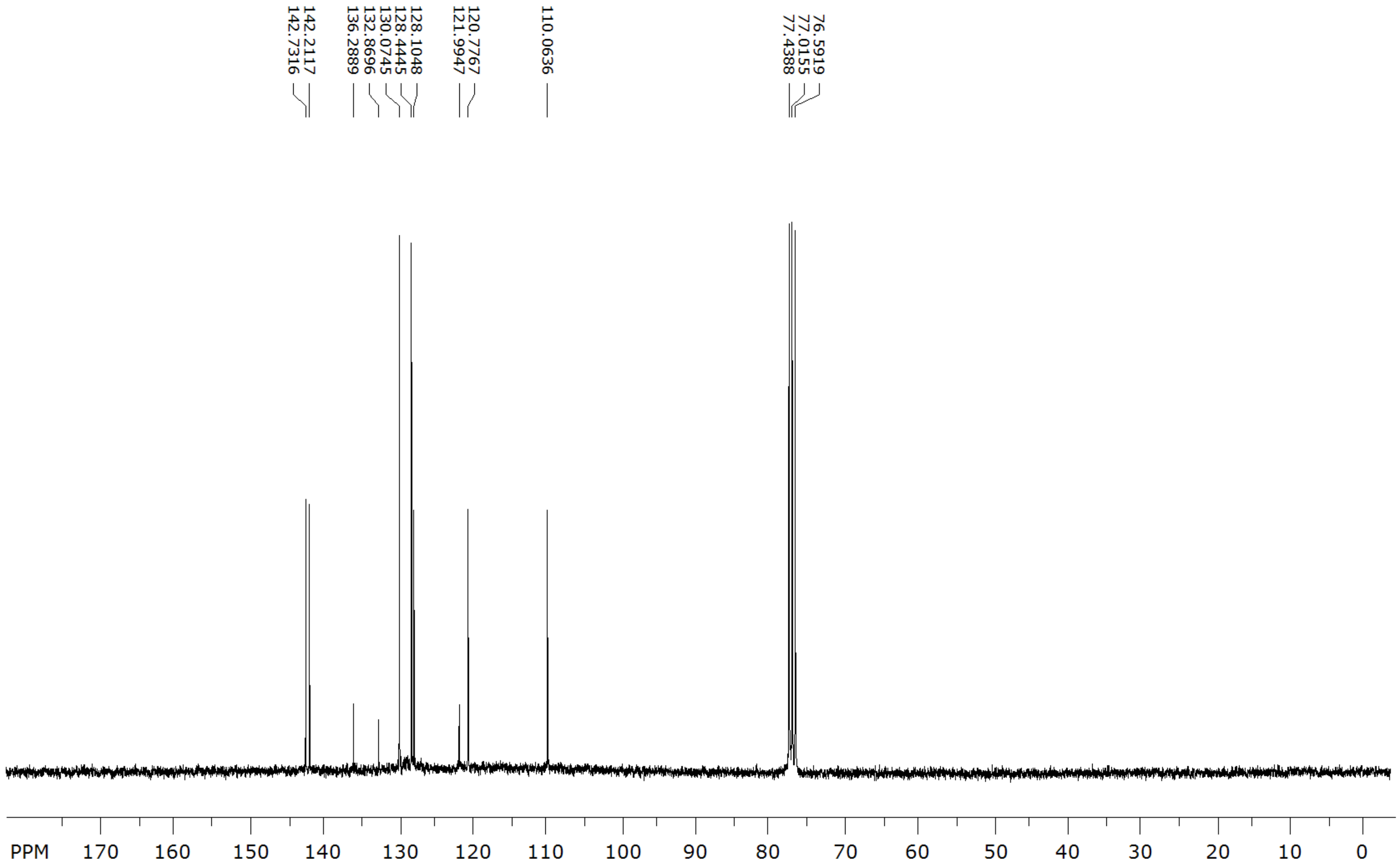

Figure S50. ^{1}H NMR ($CDCl_3$) spectrum of *cis*-**58**.

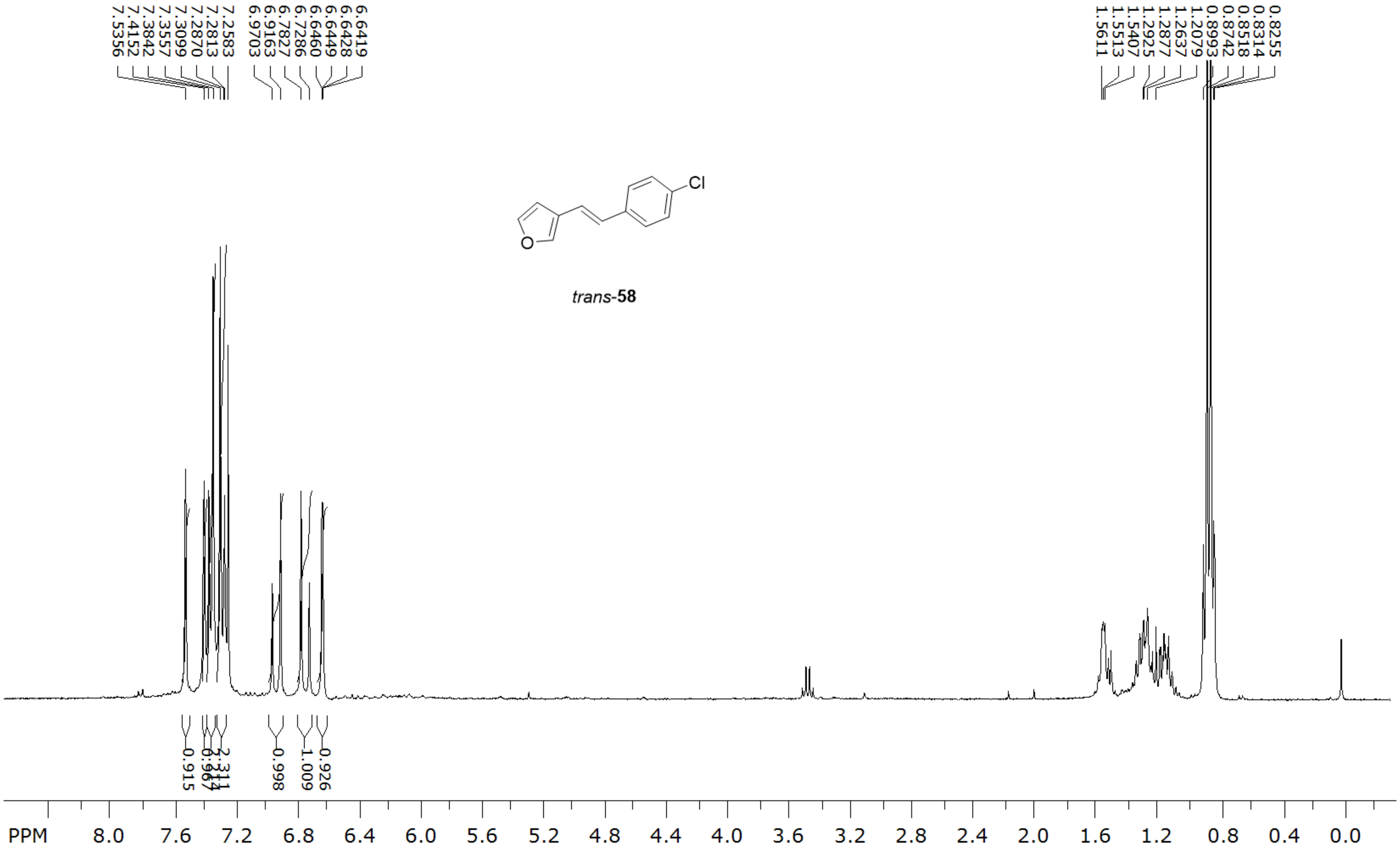

Figure S51. ^{1}H NMR ($CDCl_3$) spectrum of *trans*-**58**.

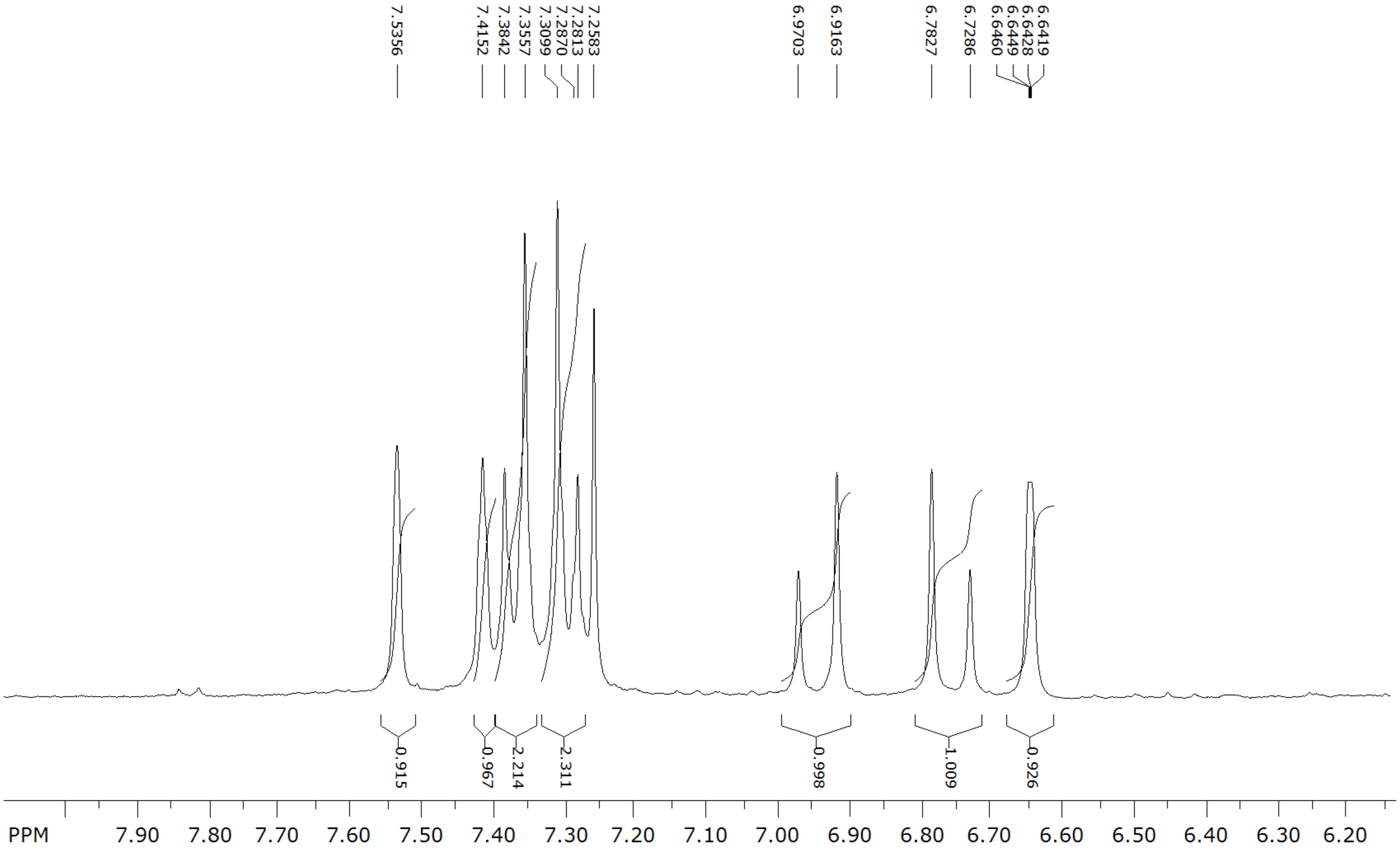

Figure S52. ^{1}H NMR ($CDCl_3$) spectrum of aromatic part of *trans*-**58**.

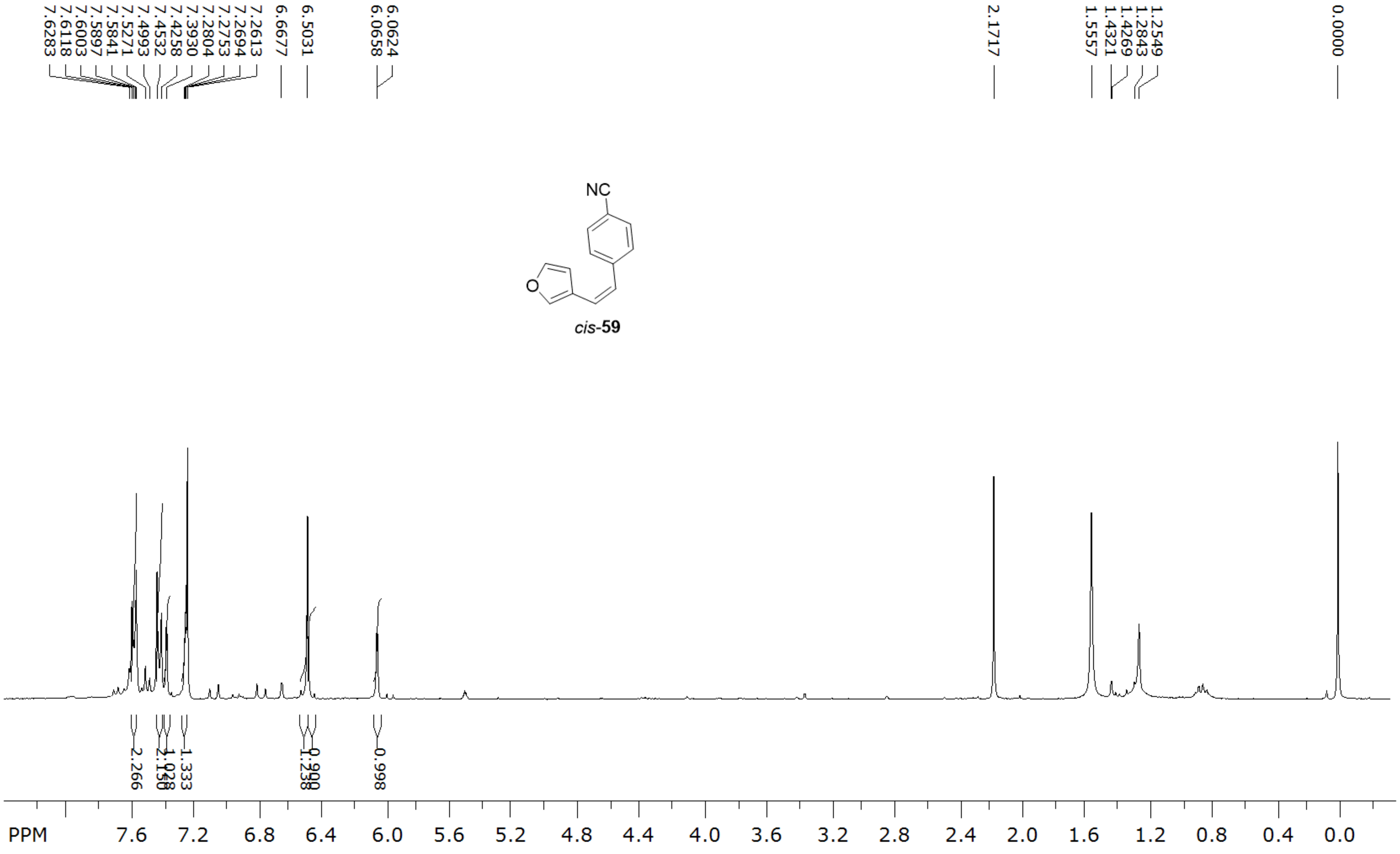

Figure S53. ^{1}H NMR ($CDCl_3$) spectrum of *cis*-**59**.

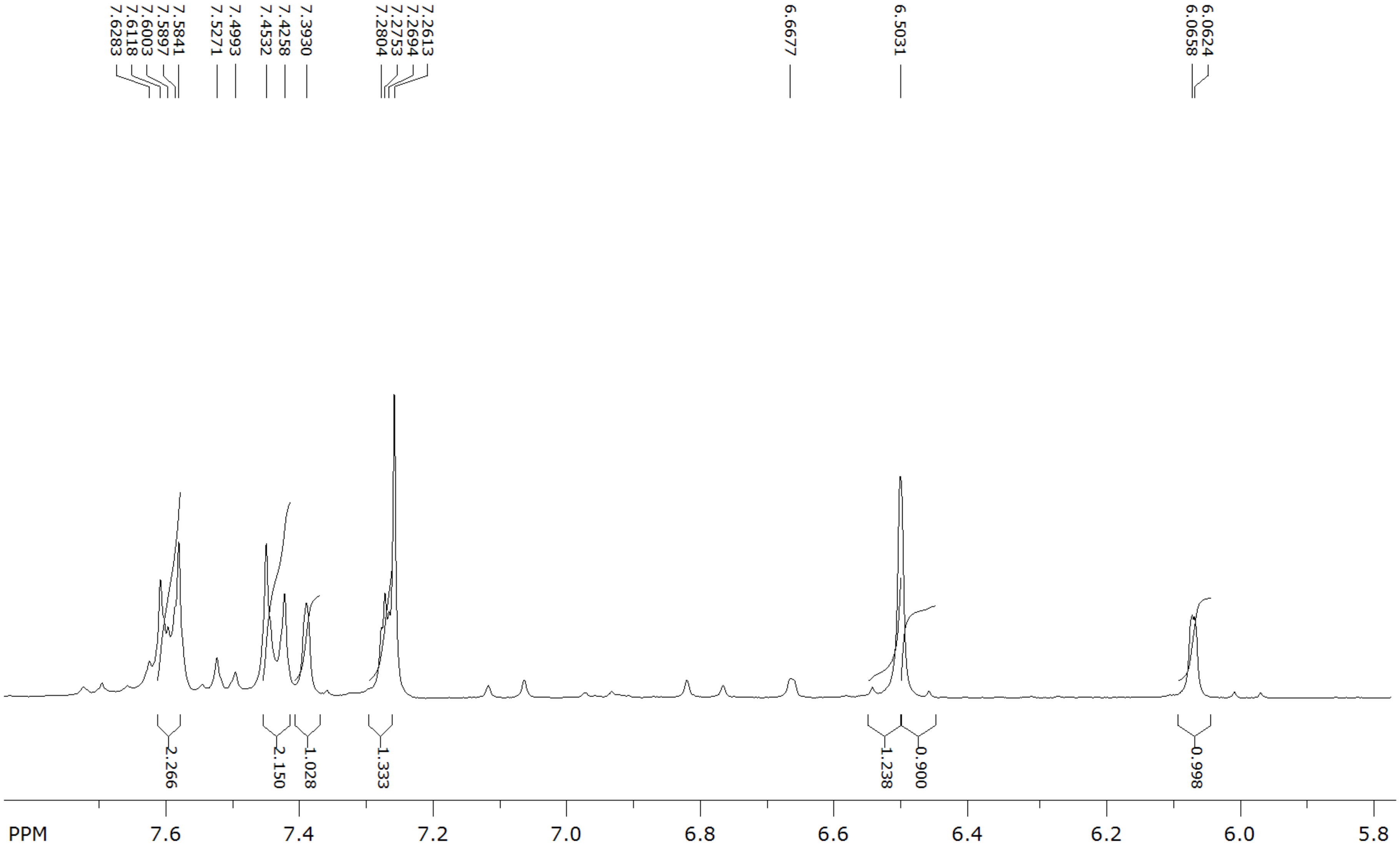

Figure S54. ^{1}H NMR ($CDCl_3$) spectrum of aromatic part of *cis*-**59**.

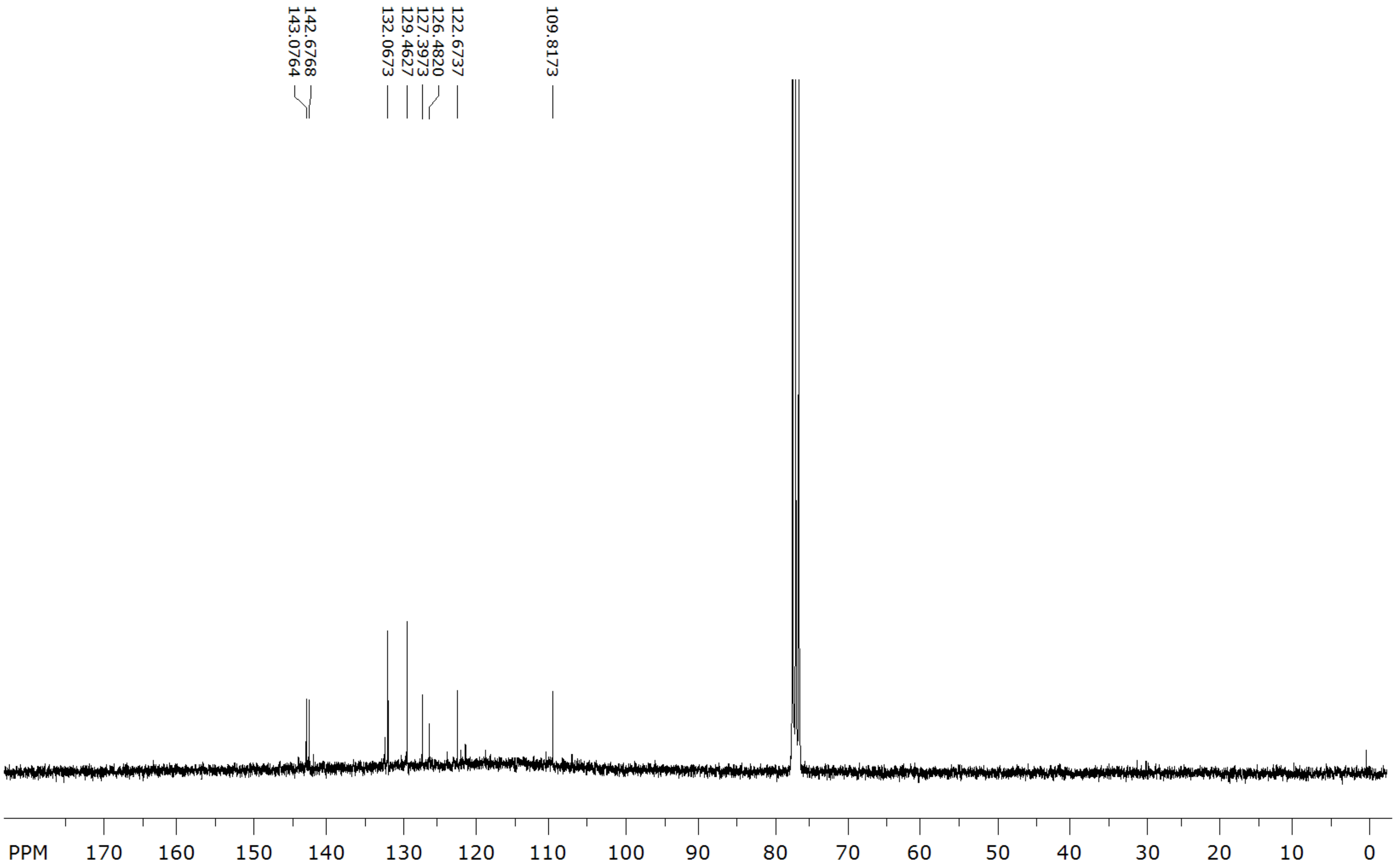

Figure S55. ^{13}C NMR ($CDCl_3$) spectrum of *cis*-**59**.

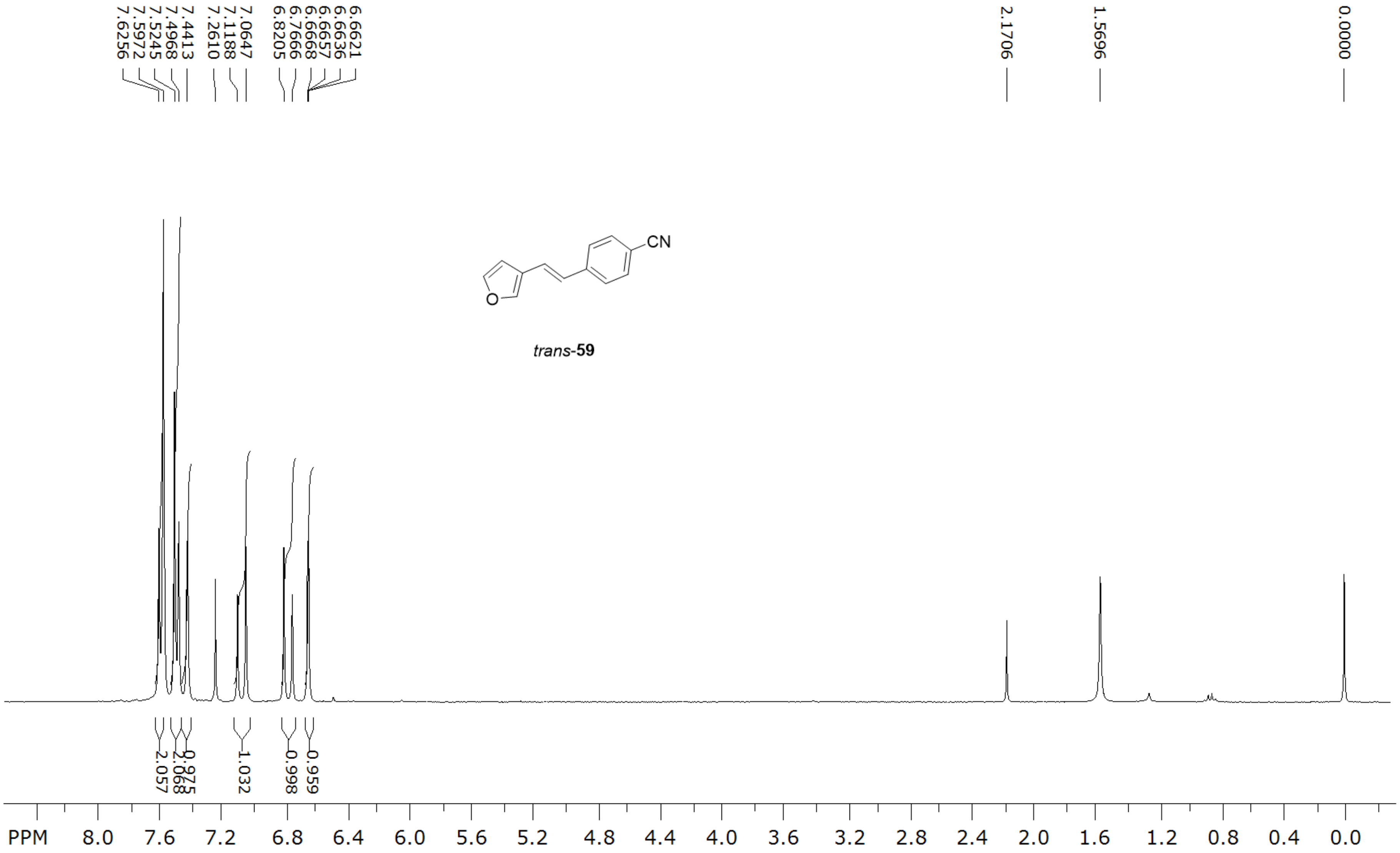

Figure S56. ^{1}H NMR ($CDCl_3$) spectrum of *trans*-**59**.

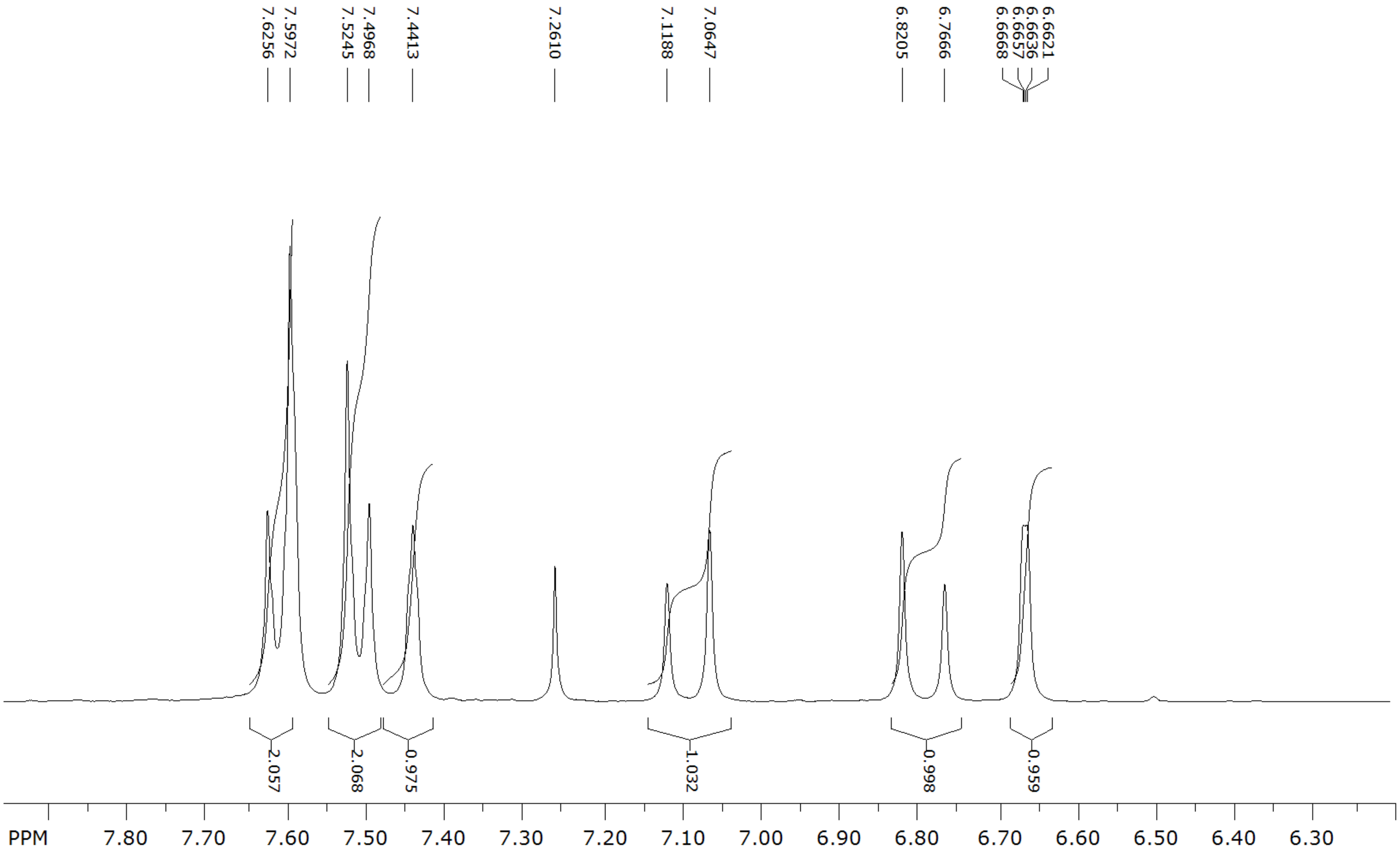

Figure S57. ^{1}H NMR ($CDCl_3$) spectrum of aromatic part of *trans*-**59**.

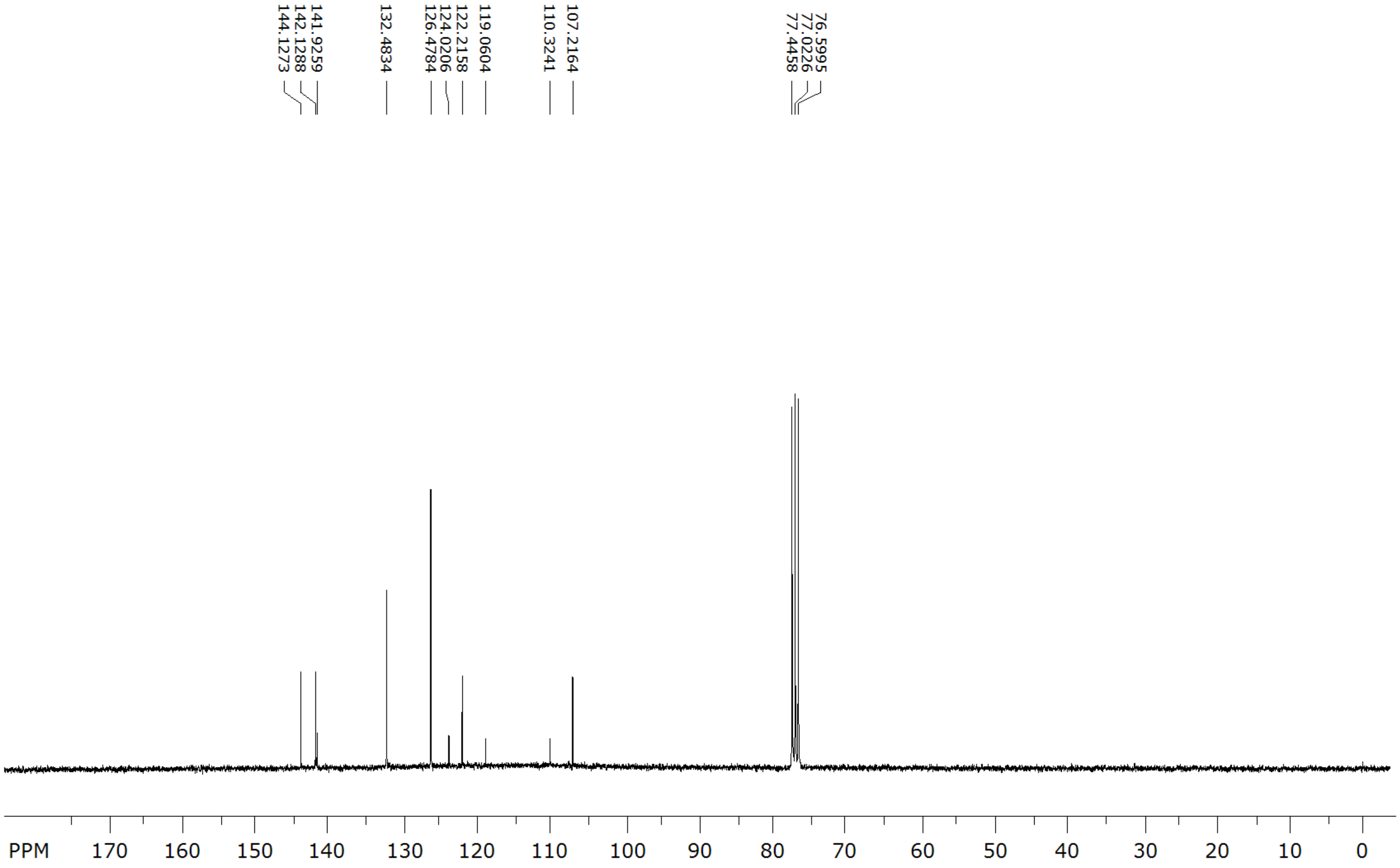

Figure S58. ^{13}C NMR ($CDCl_3$) spectrum of *trans*-**59**.

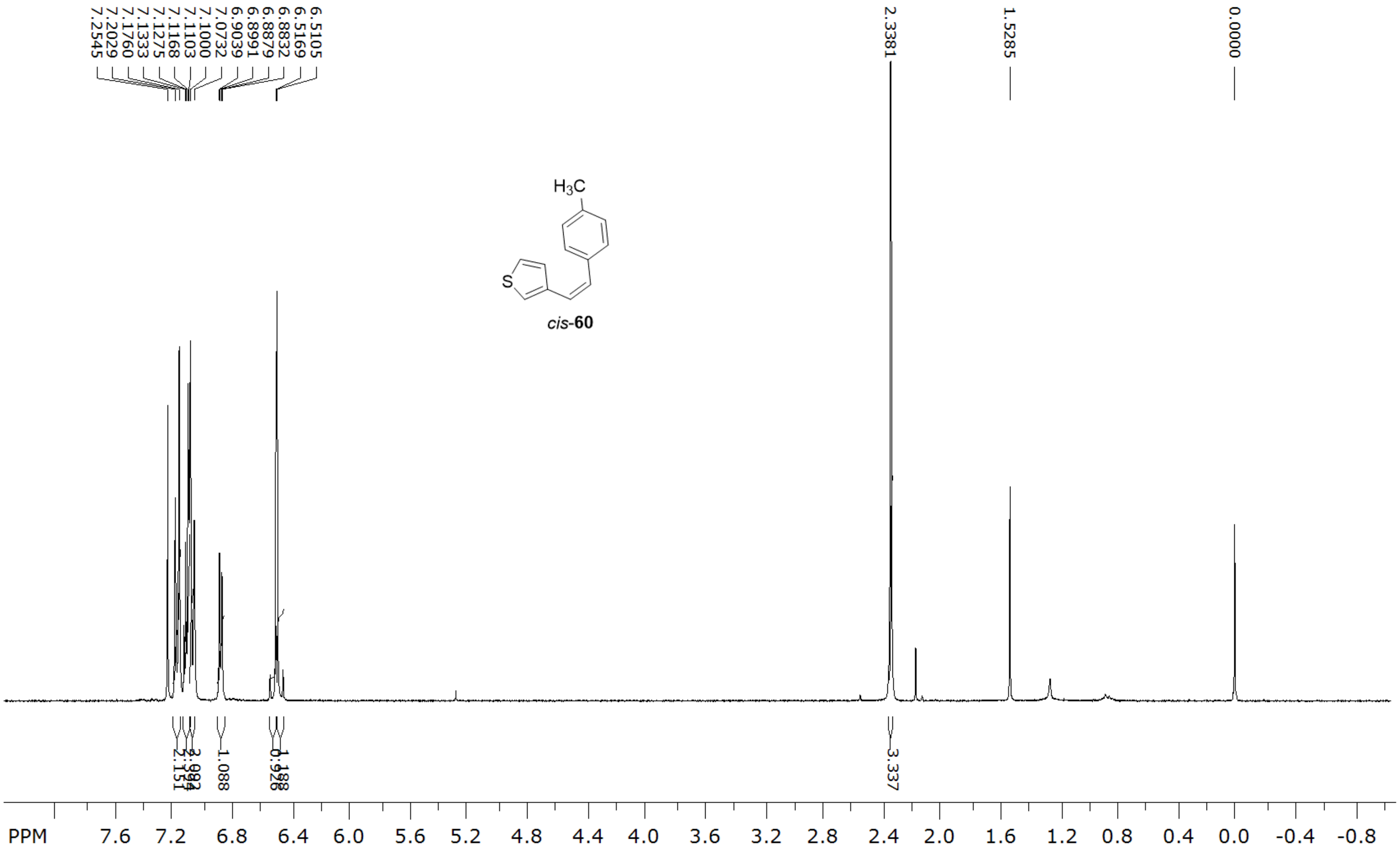

Figure S59. ^{1}H NMR ($CDCl_3$) spectrum of *cis*-**60**.

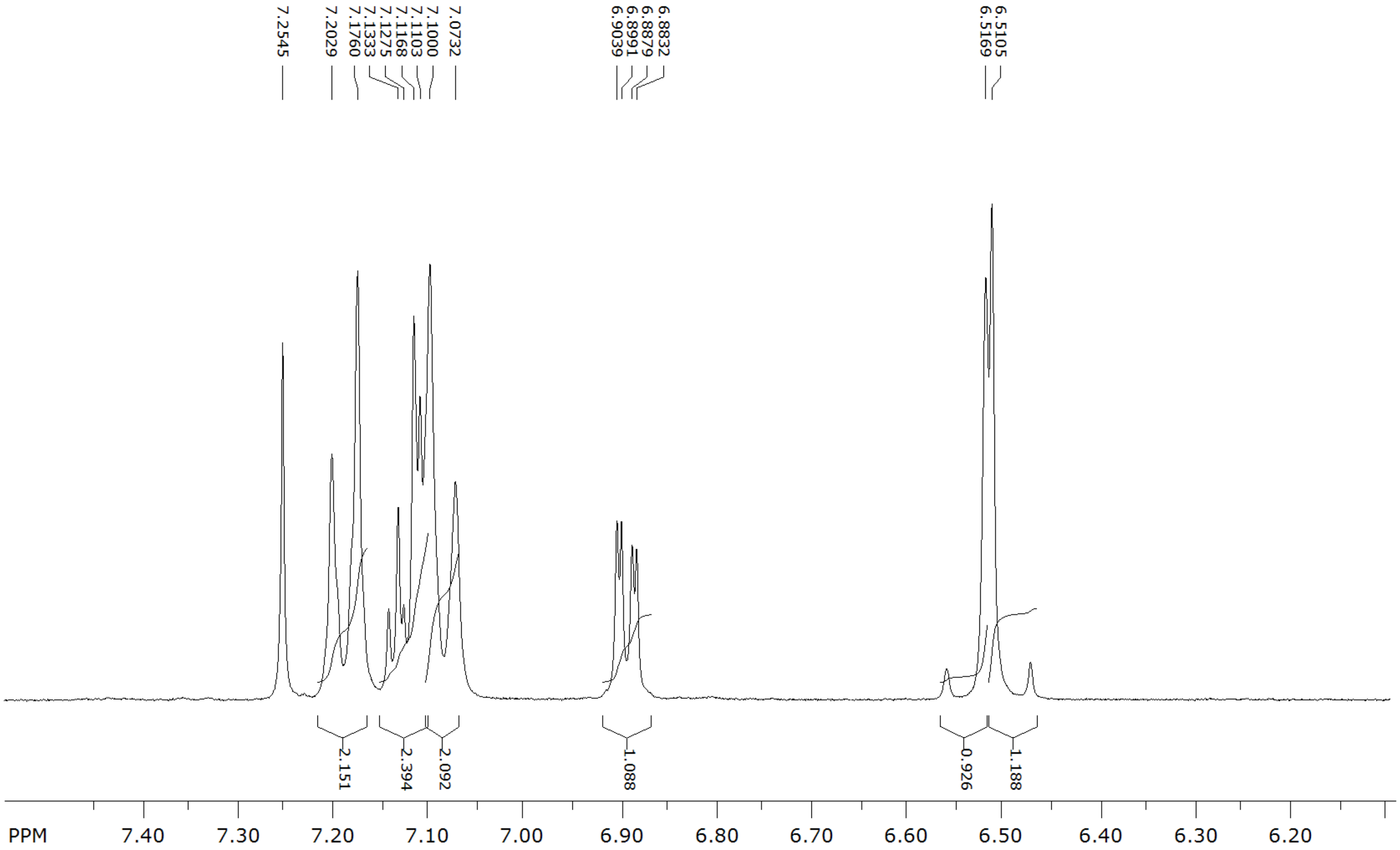

Figure S60. ^{1}H NMR ($CDCl_3$) spectrum of aromatic part of *cis*-**60**.

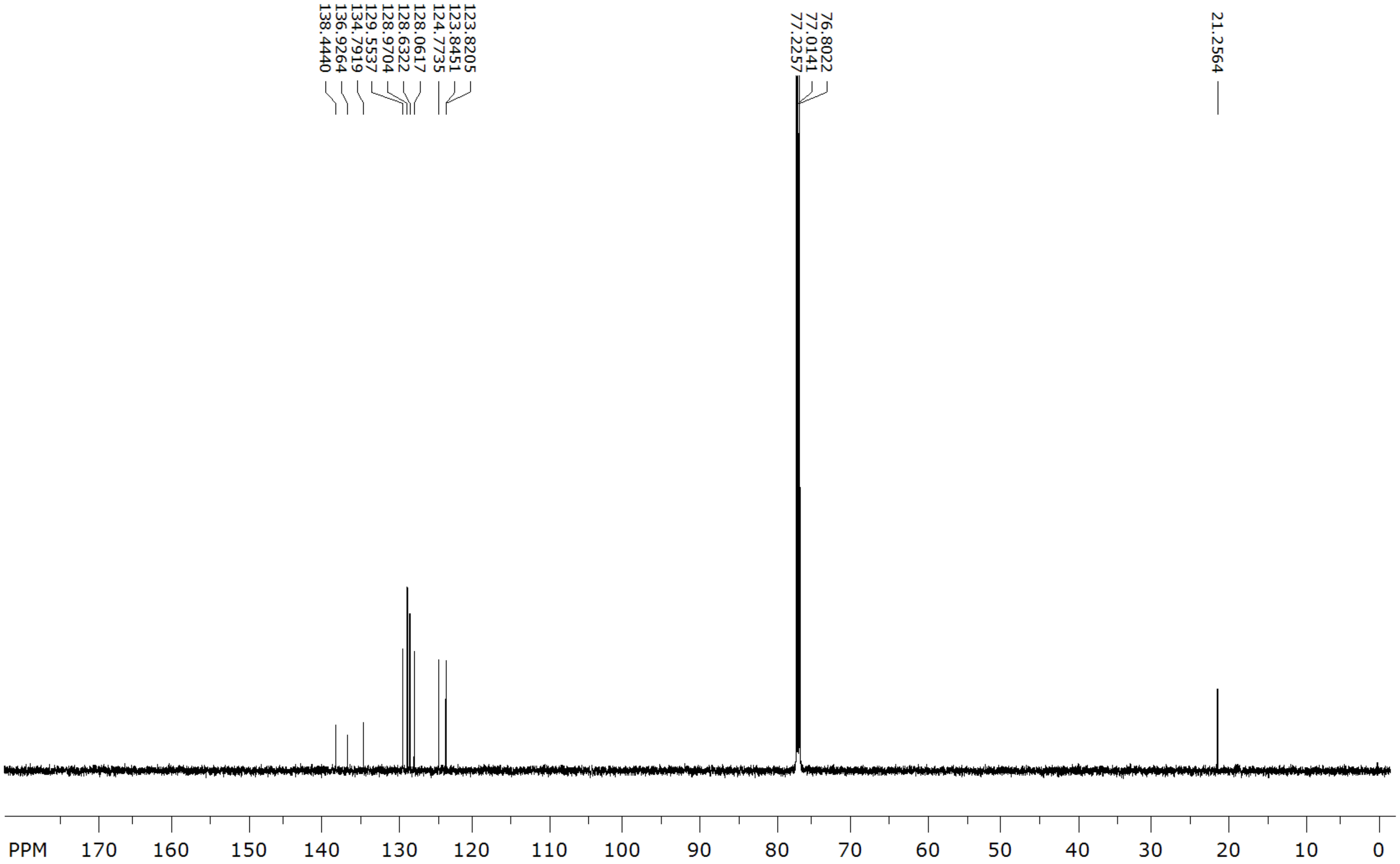

Figure S61. ^{13}C NMR ($CDCl_3$) spectrum of *cis*-**60**.

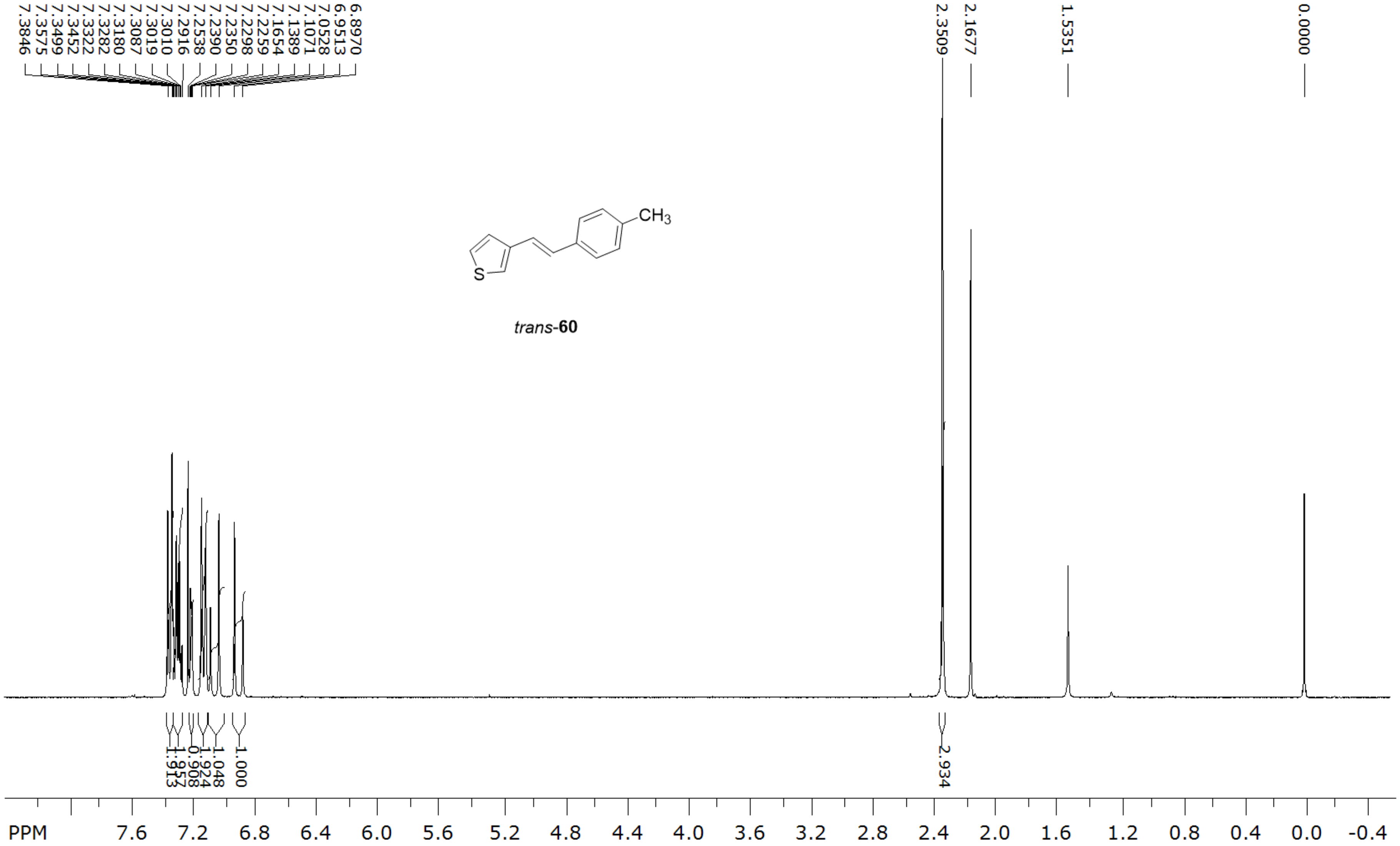

Figure S62. ^{1}H NMR ($CDCl_3$) spectrum of *trans*-**60**.

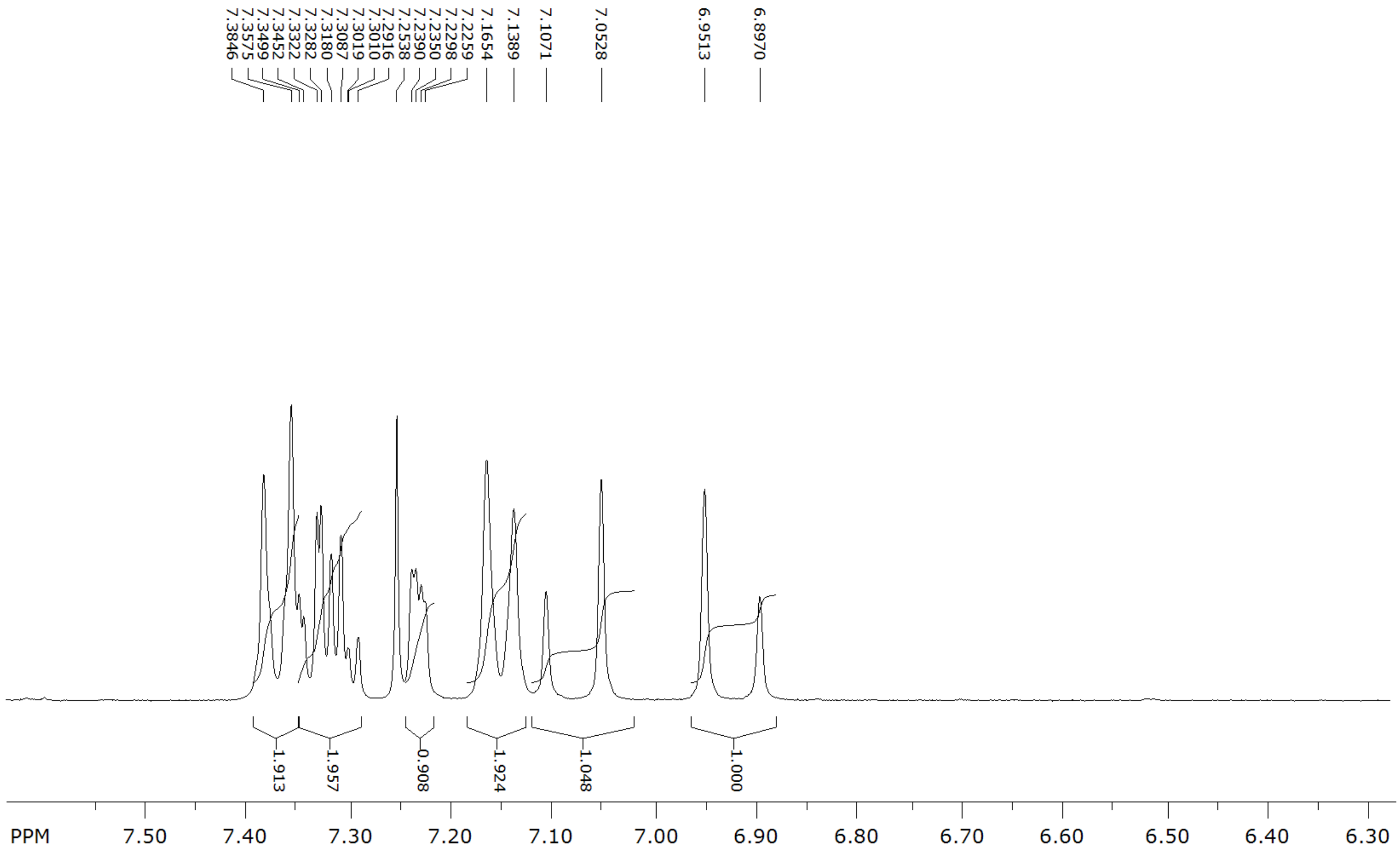

Figure S63. ^{1}H NMR ($CDCl_3$) spectrum of aromatic part of *trans*-**60**.

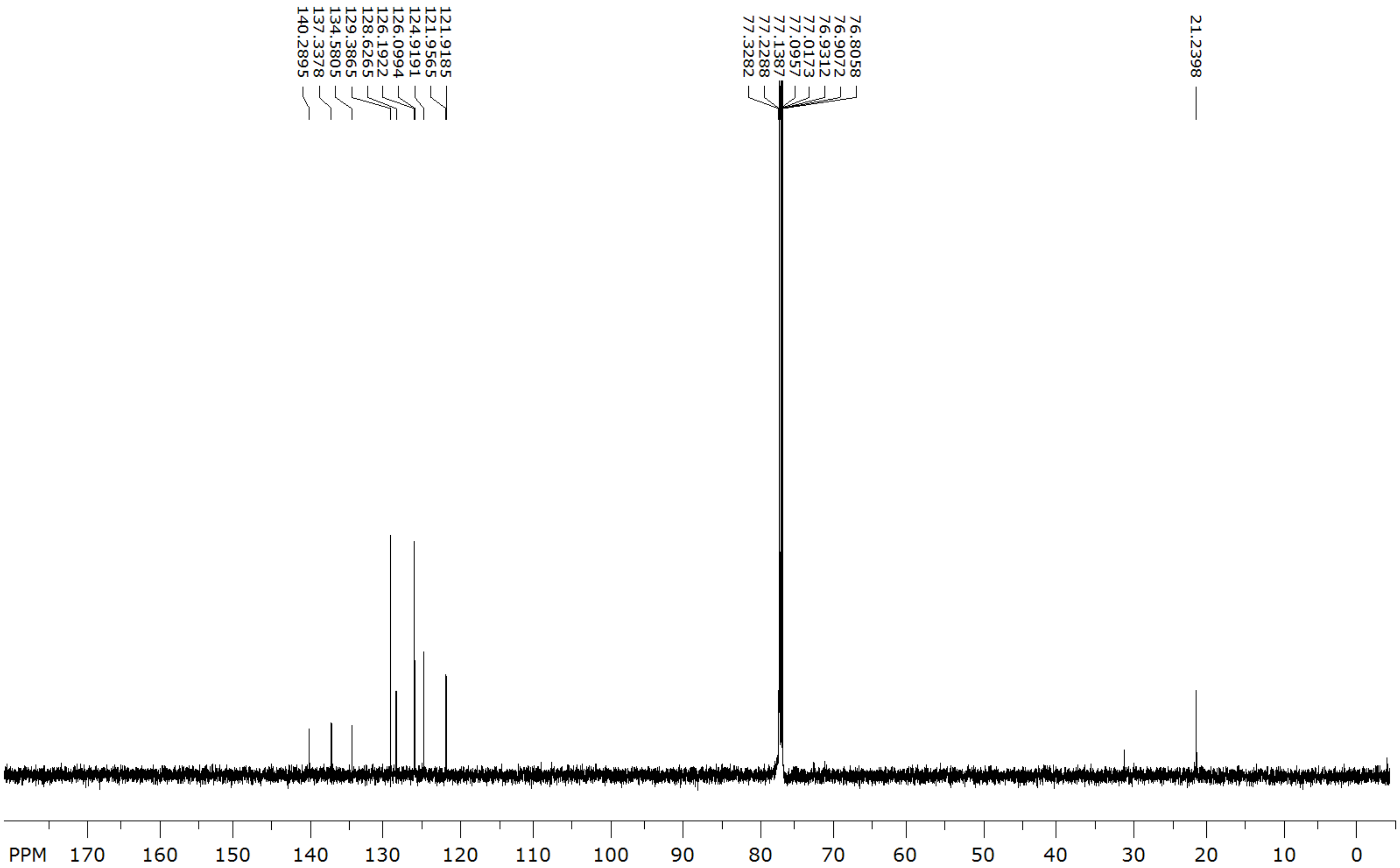

Figure S64. ^{13}C NMR ($CDCl_3$) spectrum of *trans*-**60**.

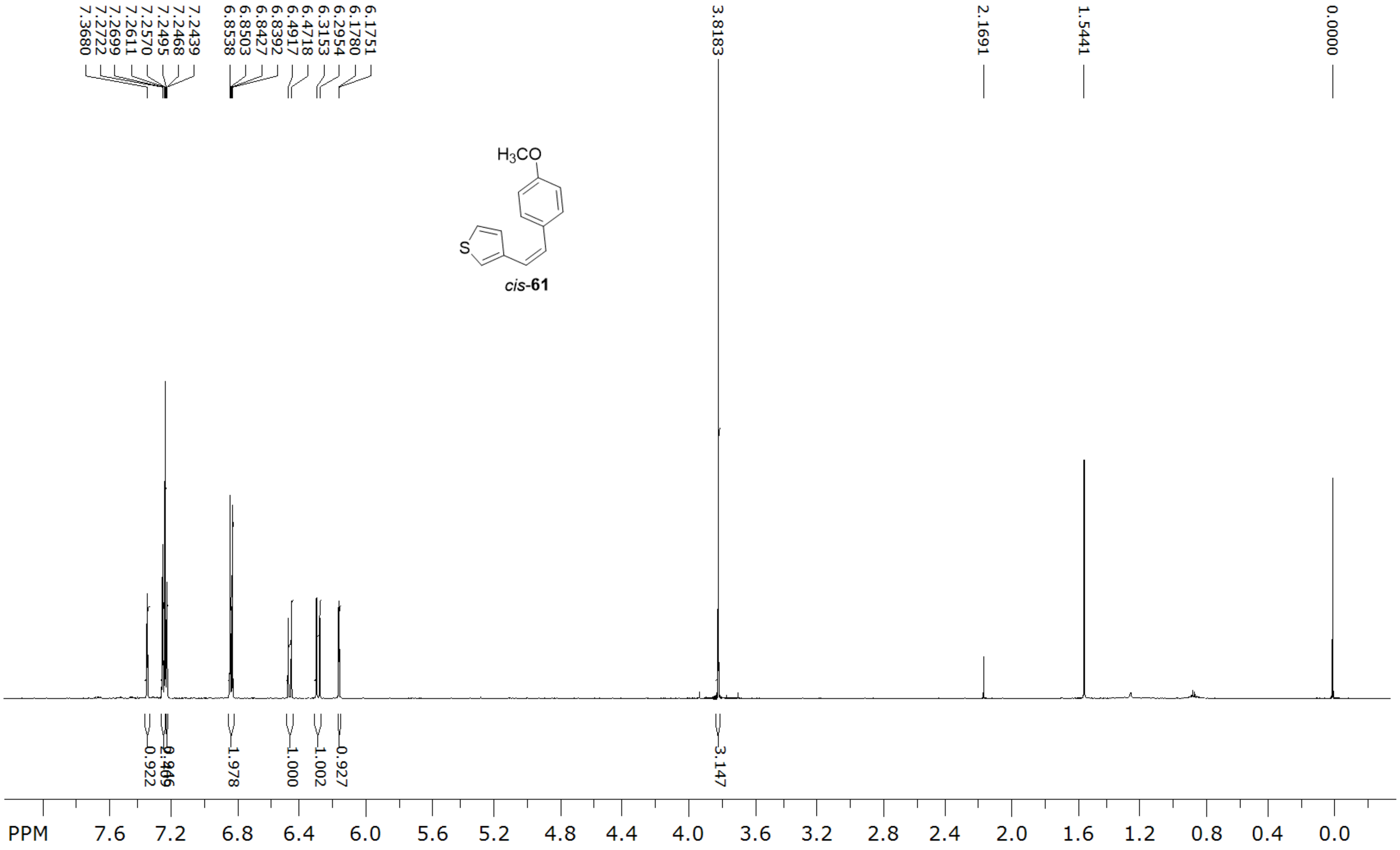

Figure S65. ^{1}H NMR ($CDCl_3$) spectrum of *cis*-**61**.

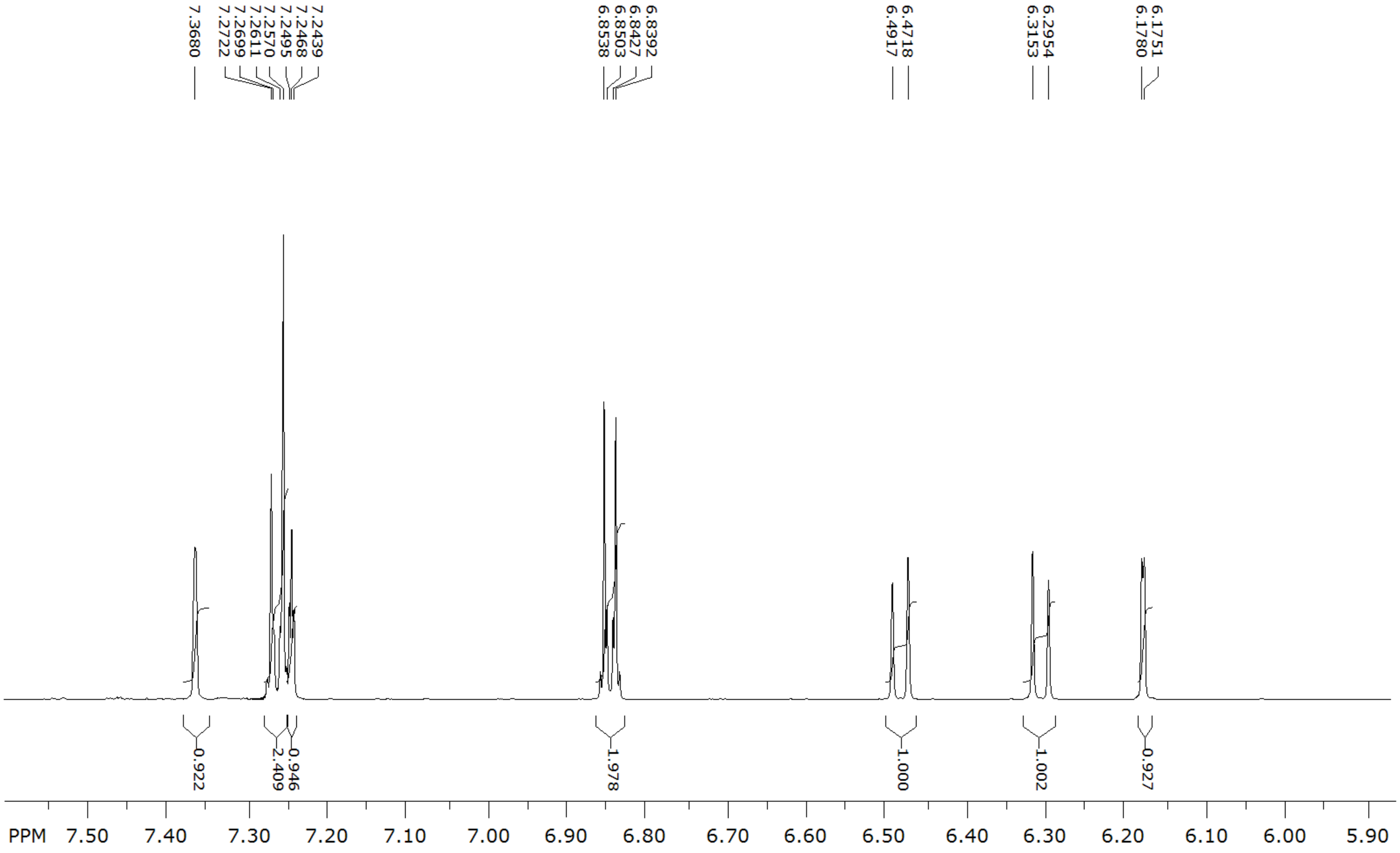

Figure S66. ^{1}H NMR ($CDCl_3$) spectrum of aromatic part of *cis*-**61**.

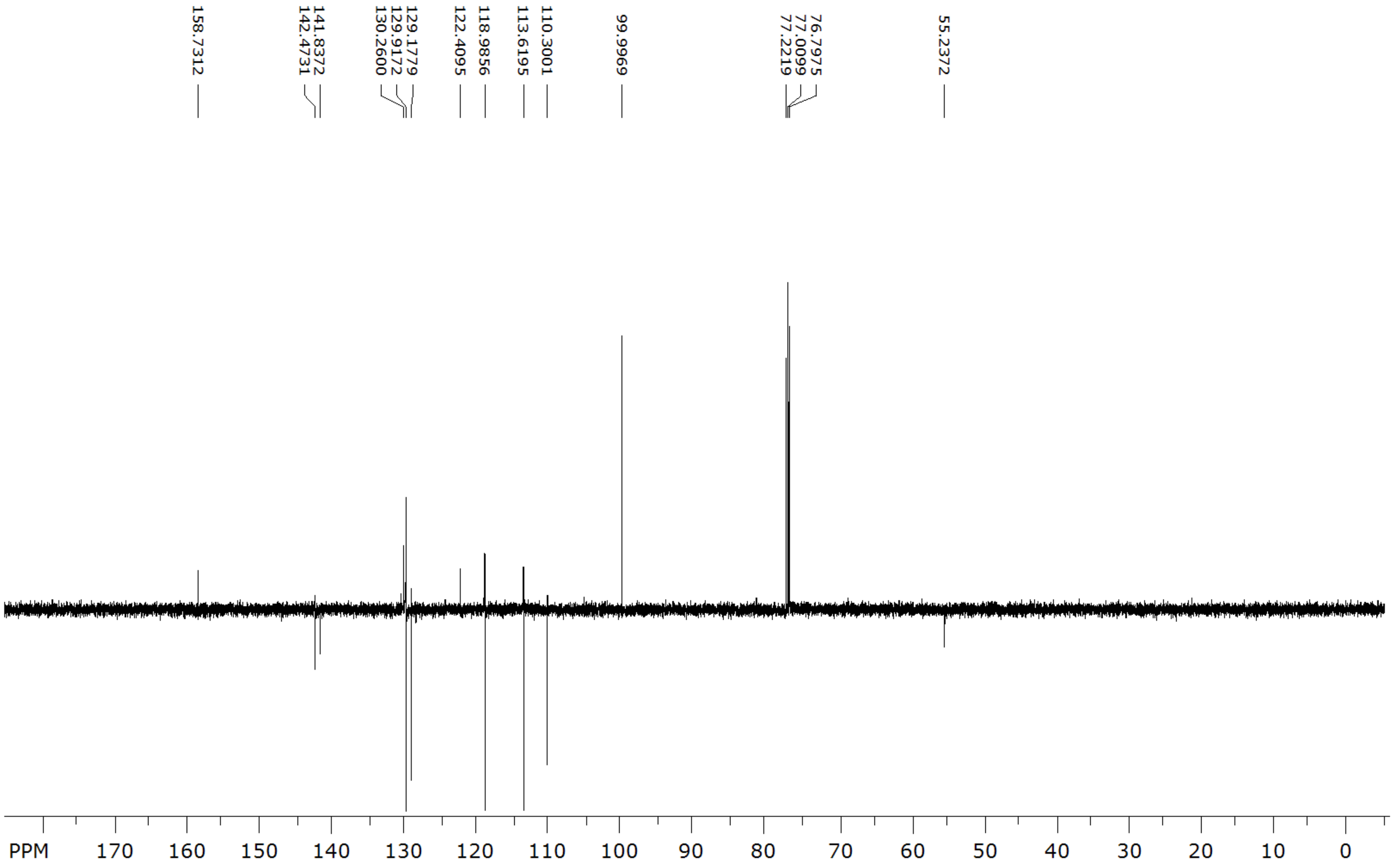

Figure S67. ^{13}C NMR ($CDCl_3$) spectrum of *cis*-**61**.

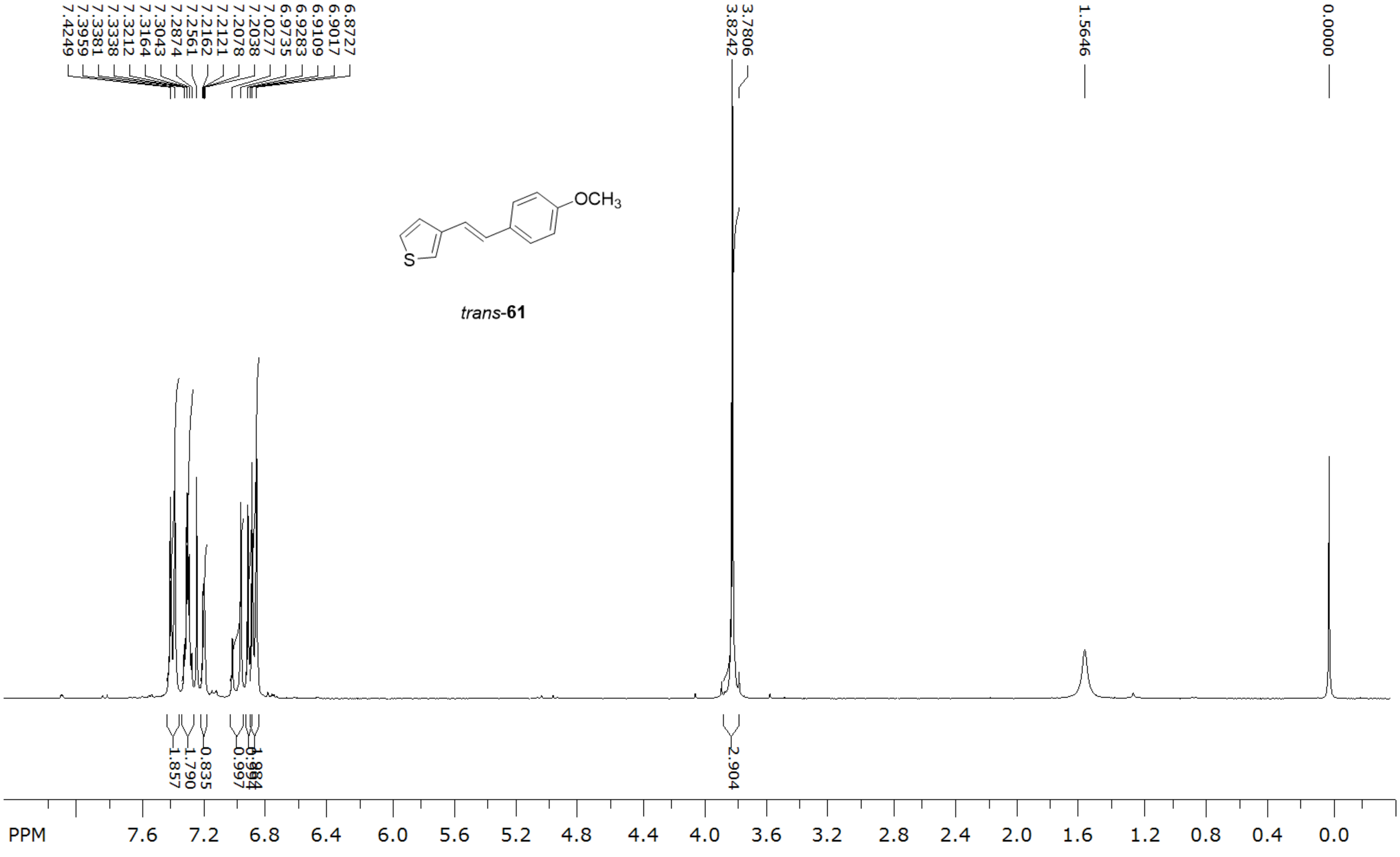

Figure S68. ¹H NMR ($CDCl_3$) spectrum of *trans*-**61**.

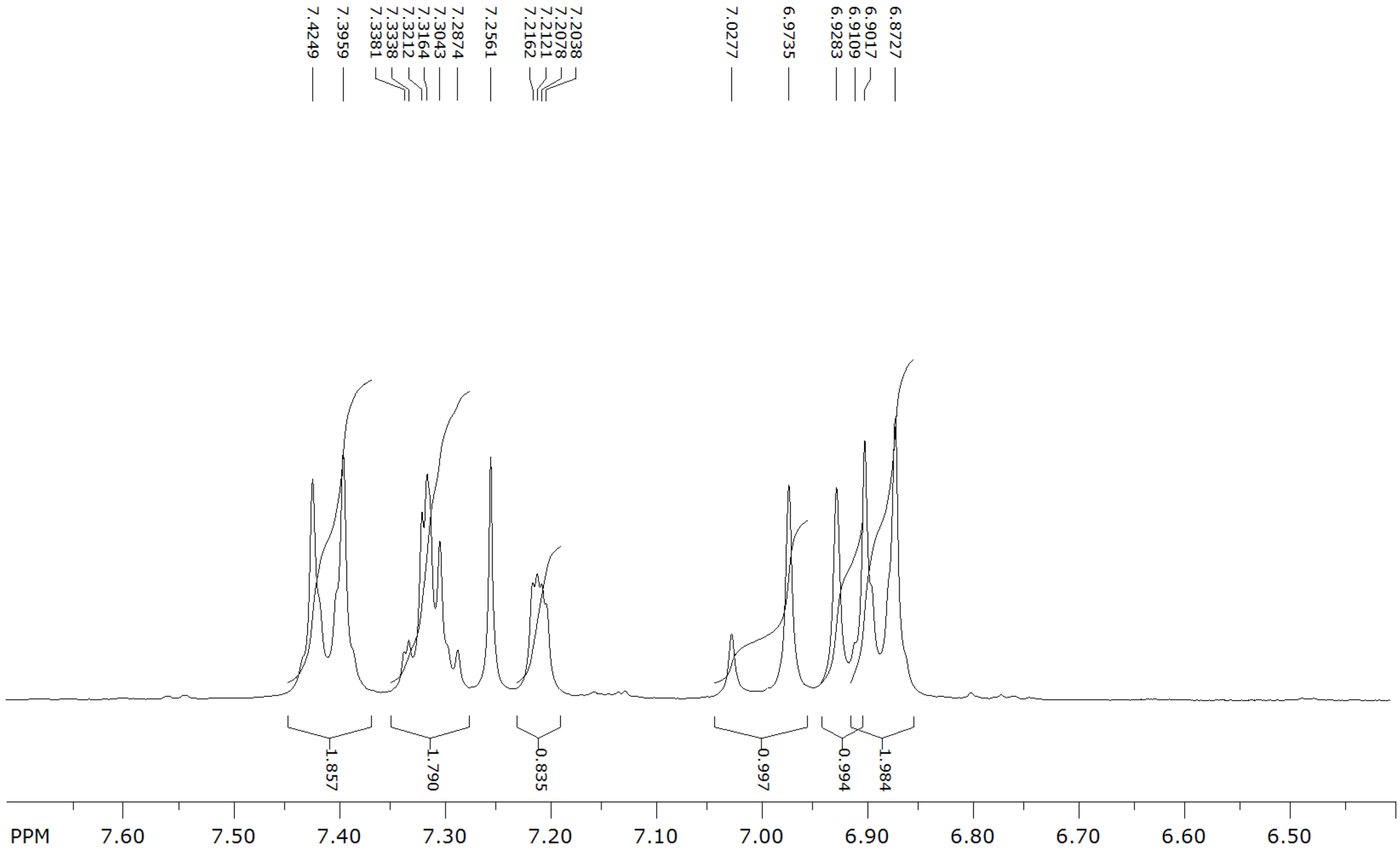

Figure S69. ^{1}H NMR ($CDCl_3$) spectrum of aromatic part of *trans*-**61**.

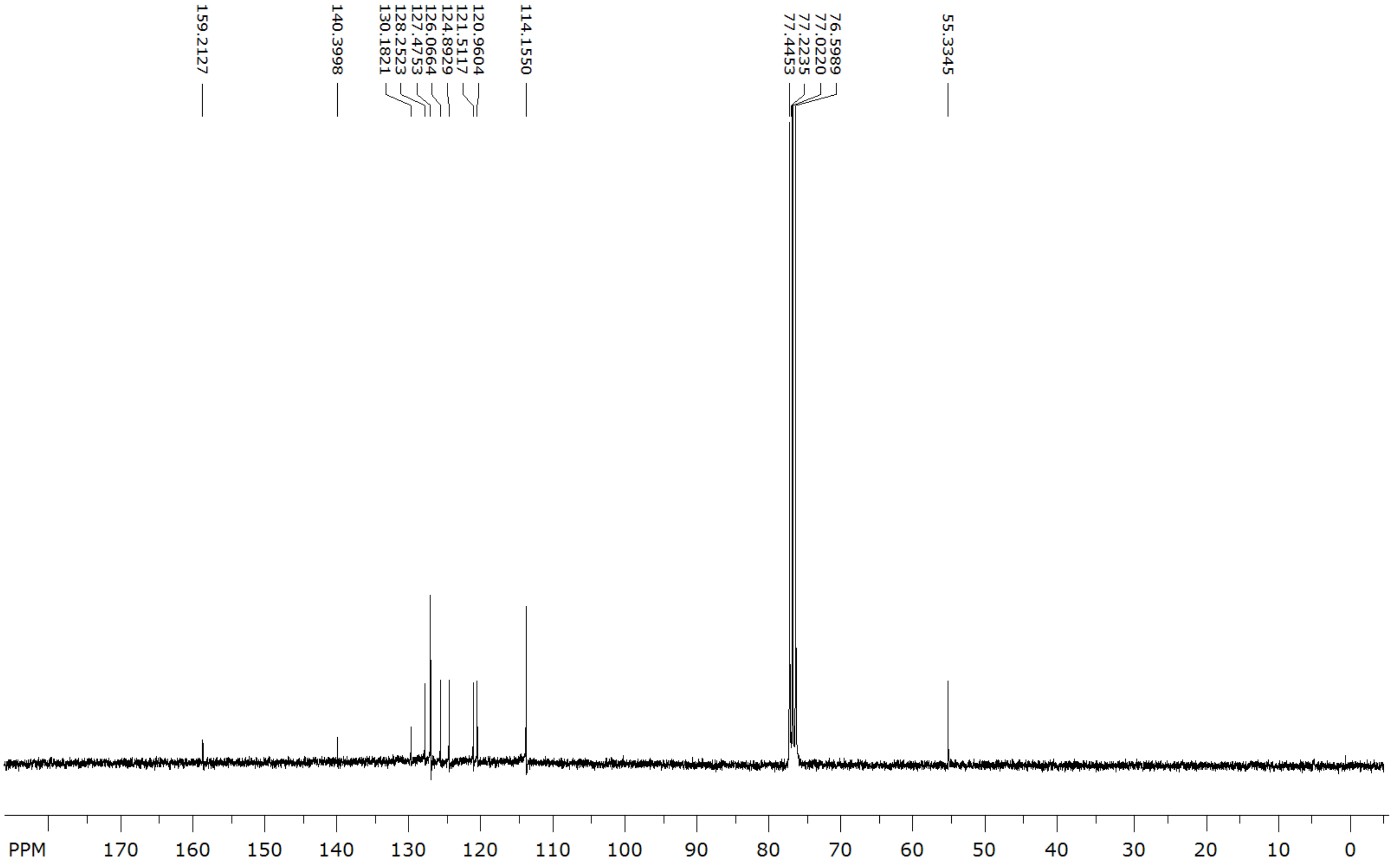

Figure S70. ^{13}C NMR ($CDCl_3$) spectrum of *trans*-**61**.

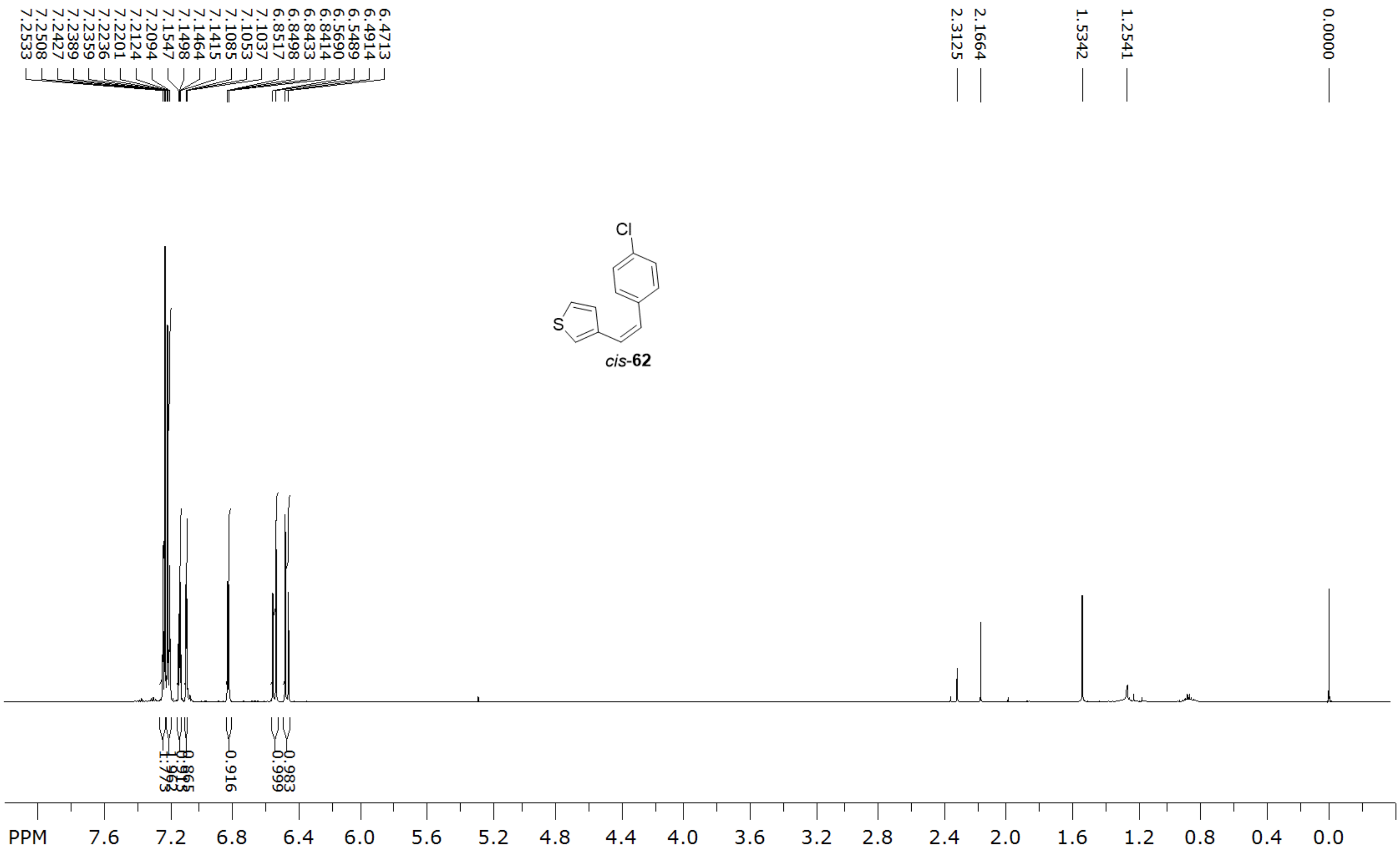

Figure S71. ^{1}H NMR ($CDCl_3$) spectrum of *cis*-**62**.

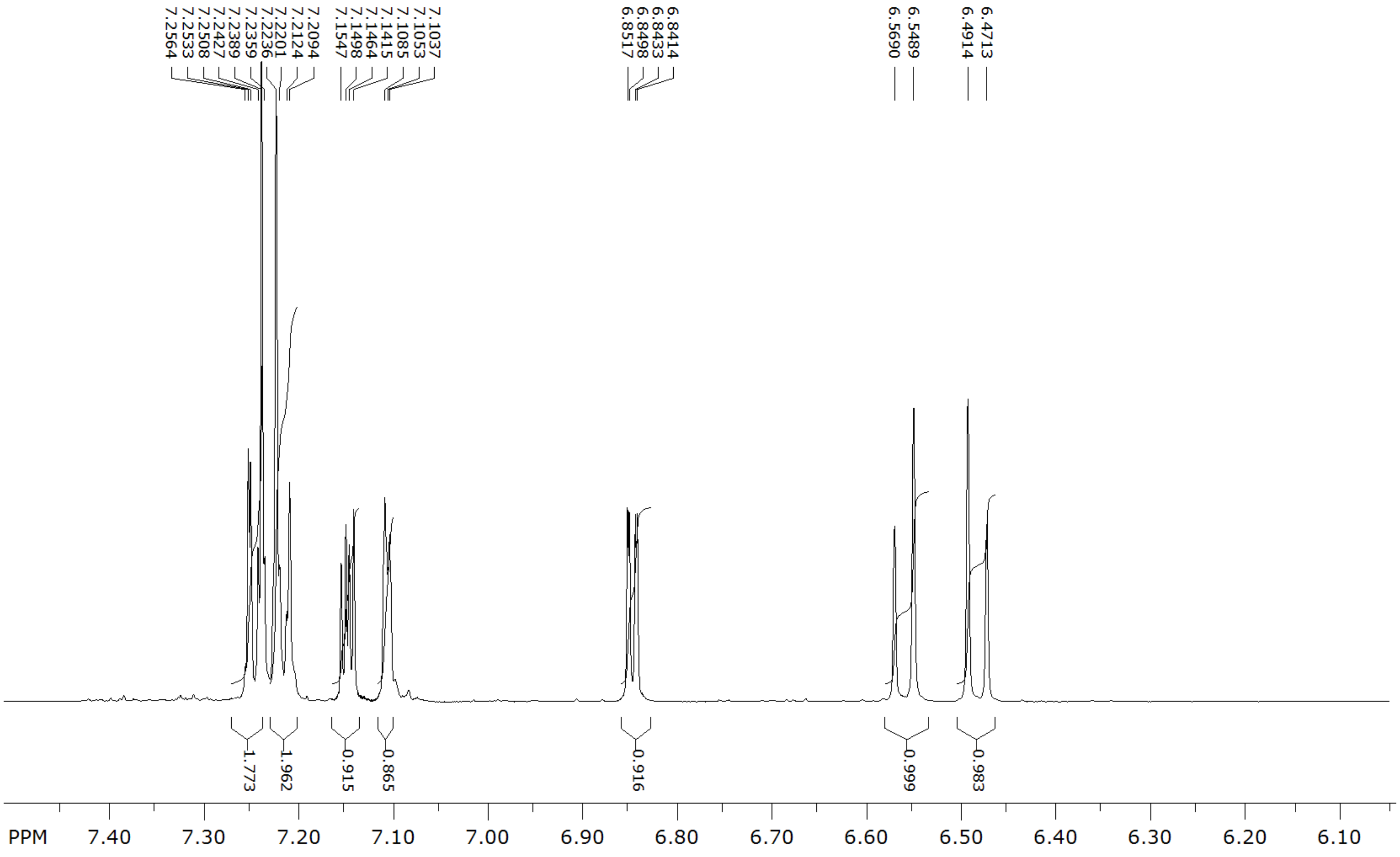

Figure S72. ^{1}H NMR ($CDCl_3$) spectrum of aromatic part of *cis*-**62**.

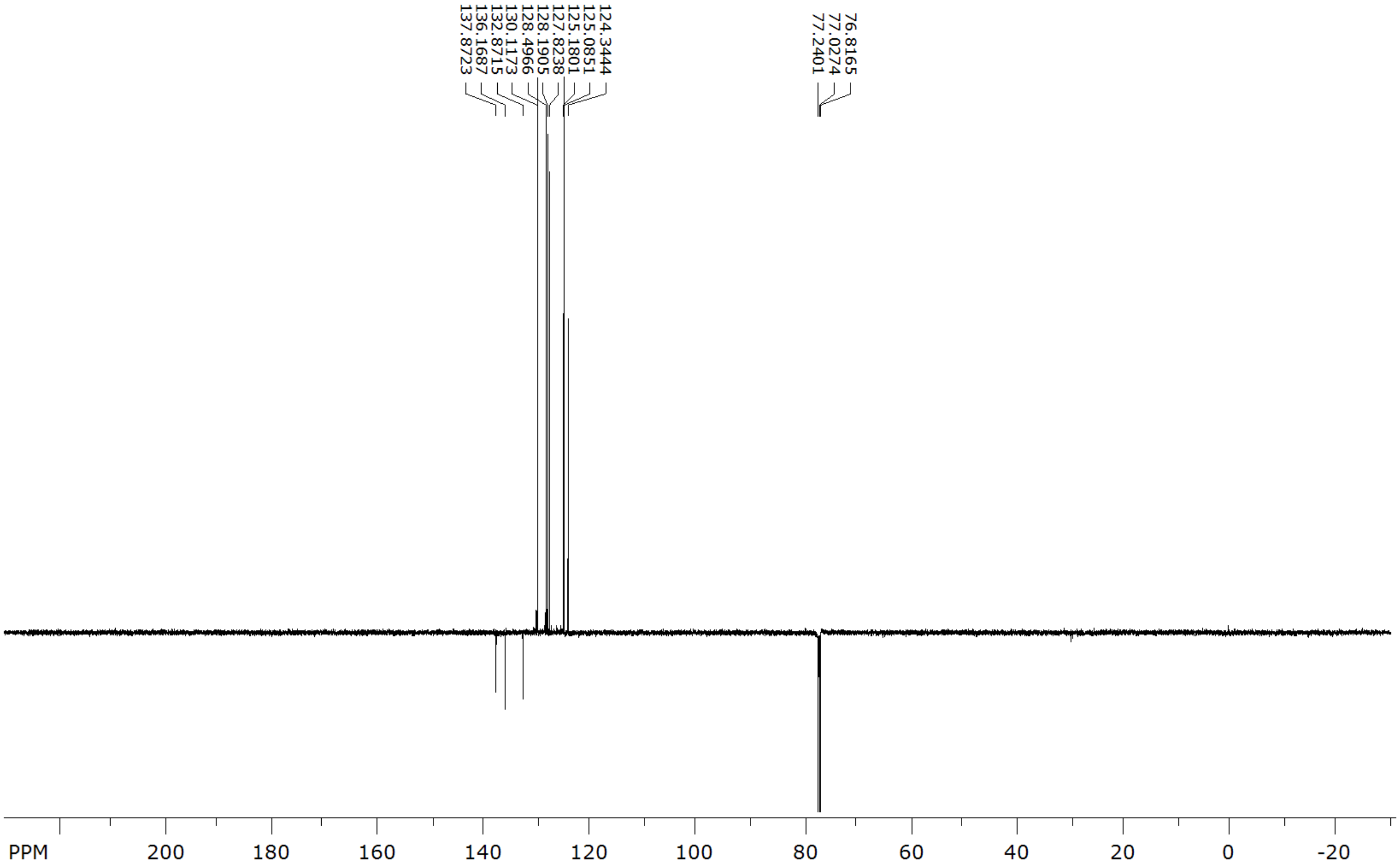

Figure S73. ^{13}C NMR ($CDCl_3$) spectrum of *cis*-**62**.

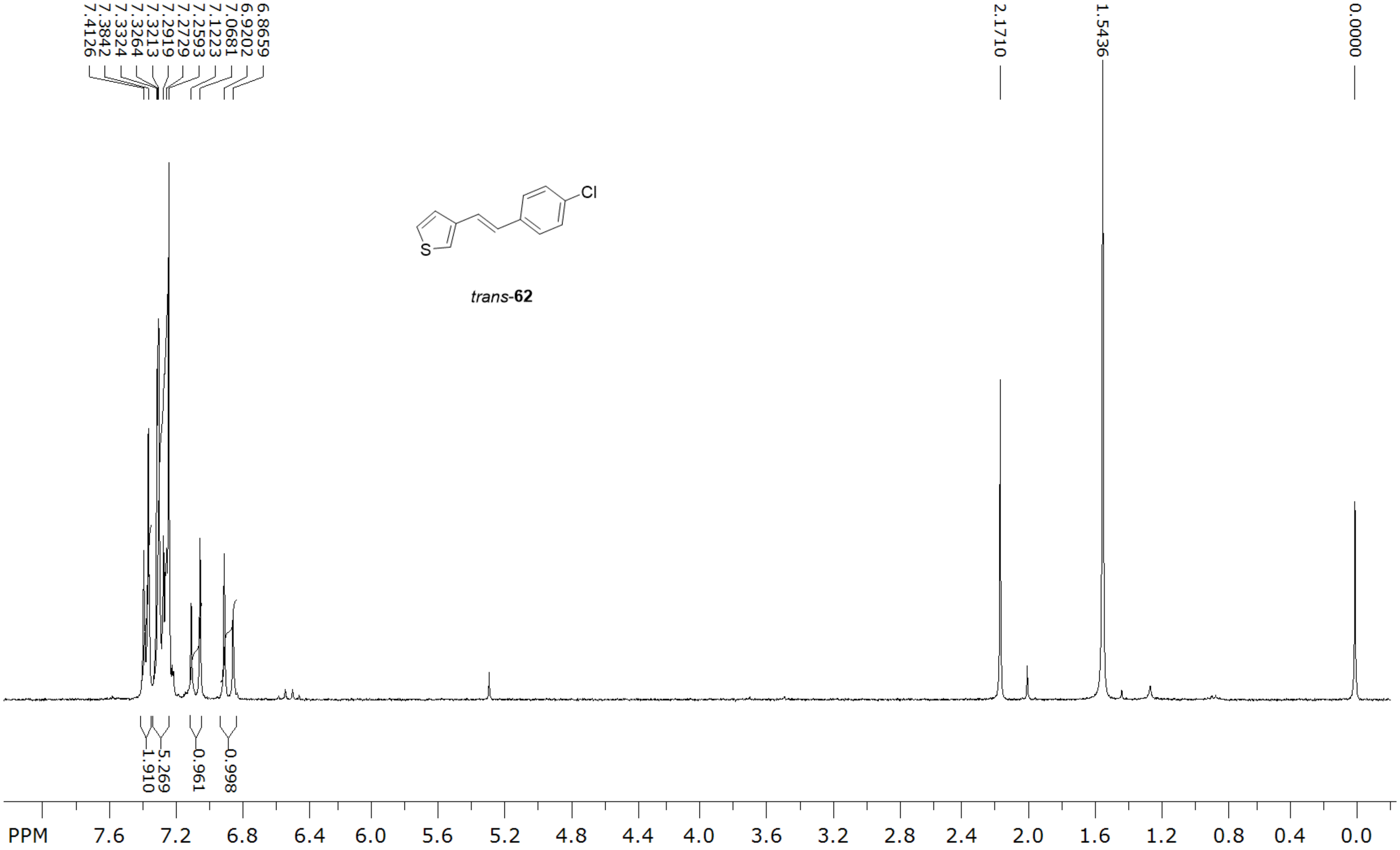

Figure S74. ^{1}H NMR ($CDCl_3$) spectrum of *trans*-**62**.

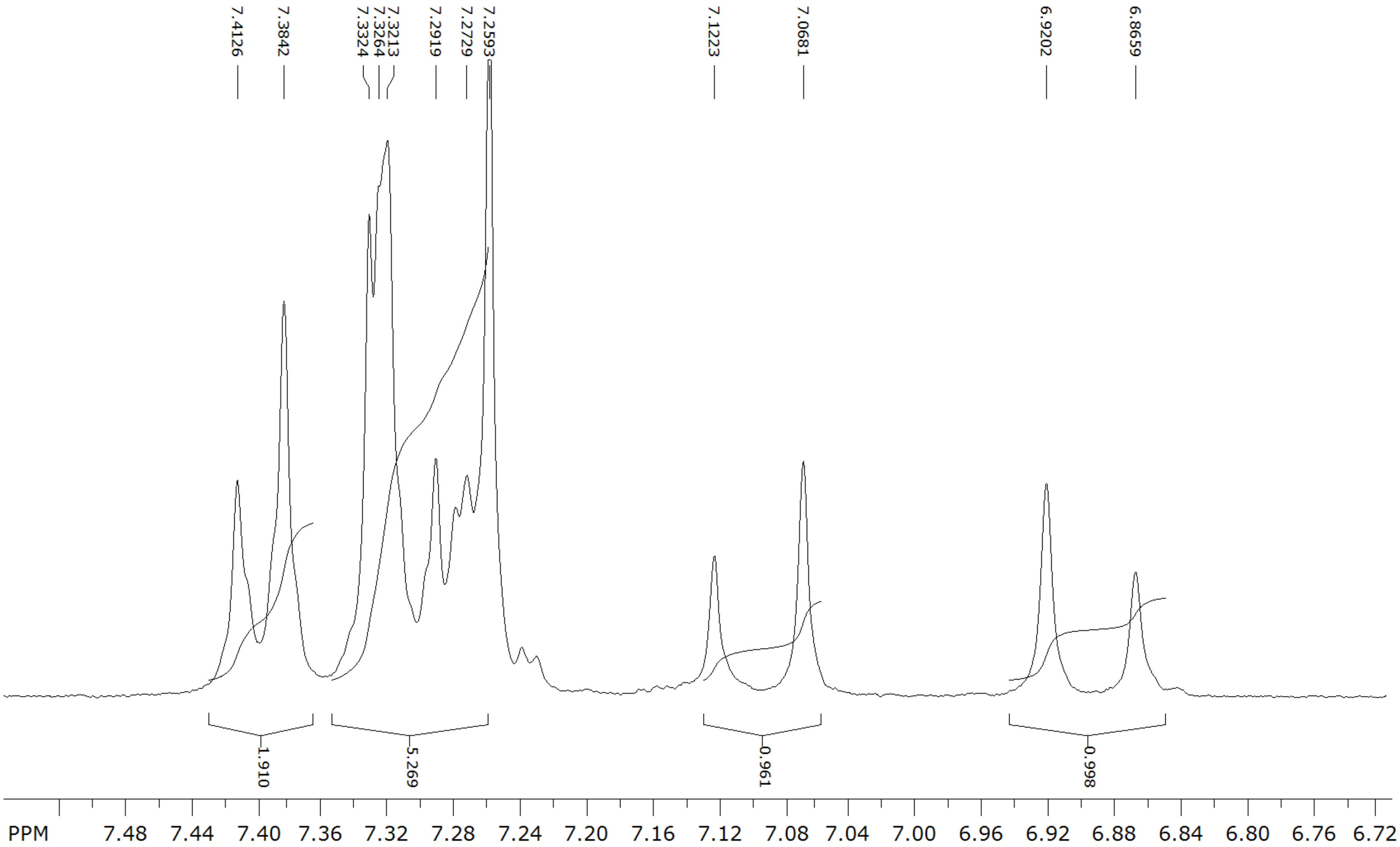

Figure S75. ^{1}H NMR ($CDCl_3$) spectrum of aromatic part of *trans*-**62**.

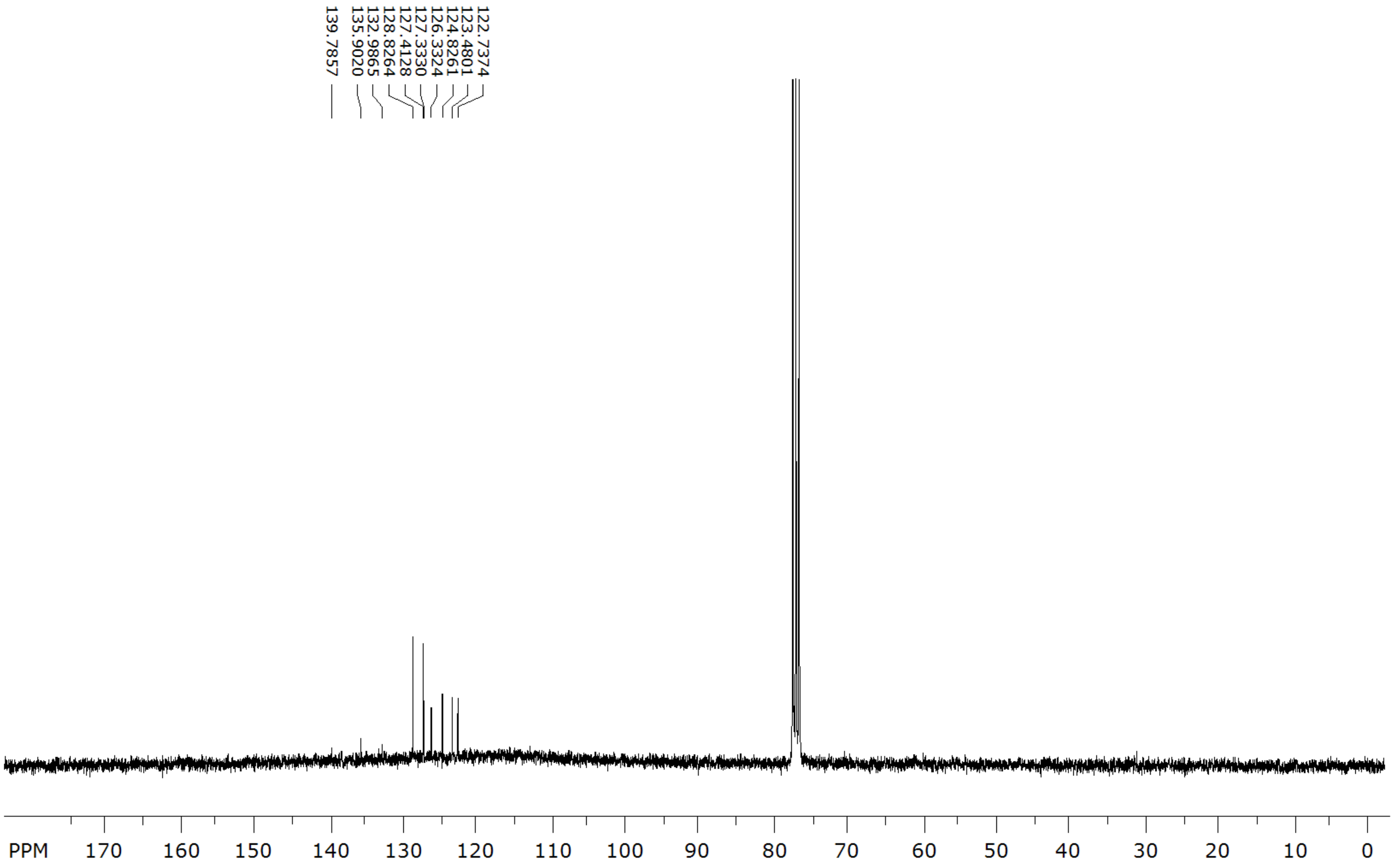

Figure S76. ^{13}C NMR ($CDCl_3$) spectrum of *trans*-**62**.

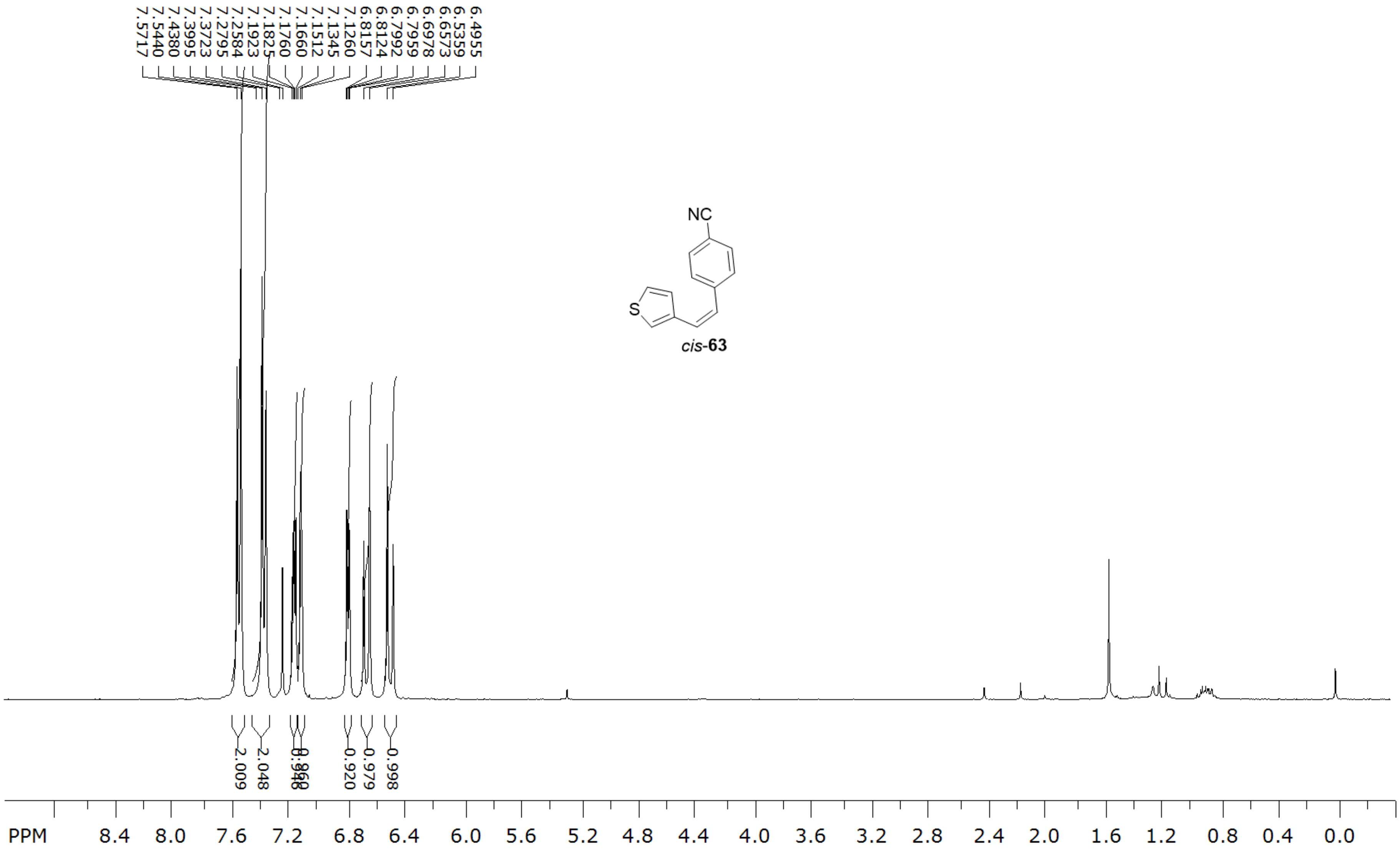

Figure S77. ^{1}H NMR ($CDCl_3$) spectrum of *cis*-**63**.

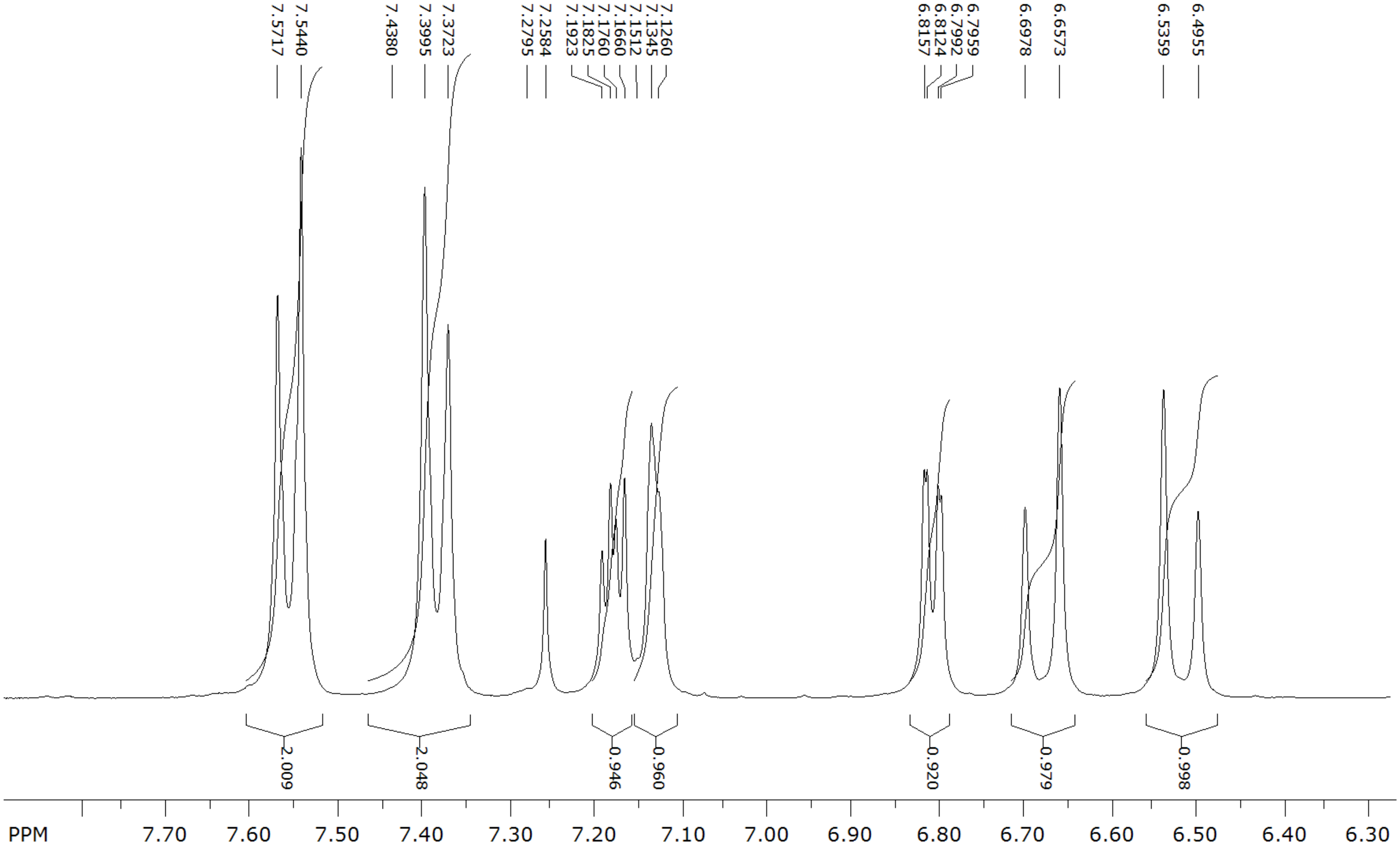

Figure S78. ^{1}H NMR ($CDCl_3$) spectrum of aromatic part of *cis*-**63**.

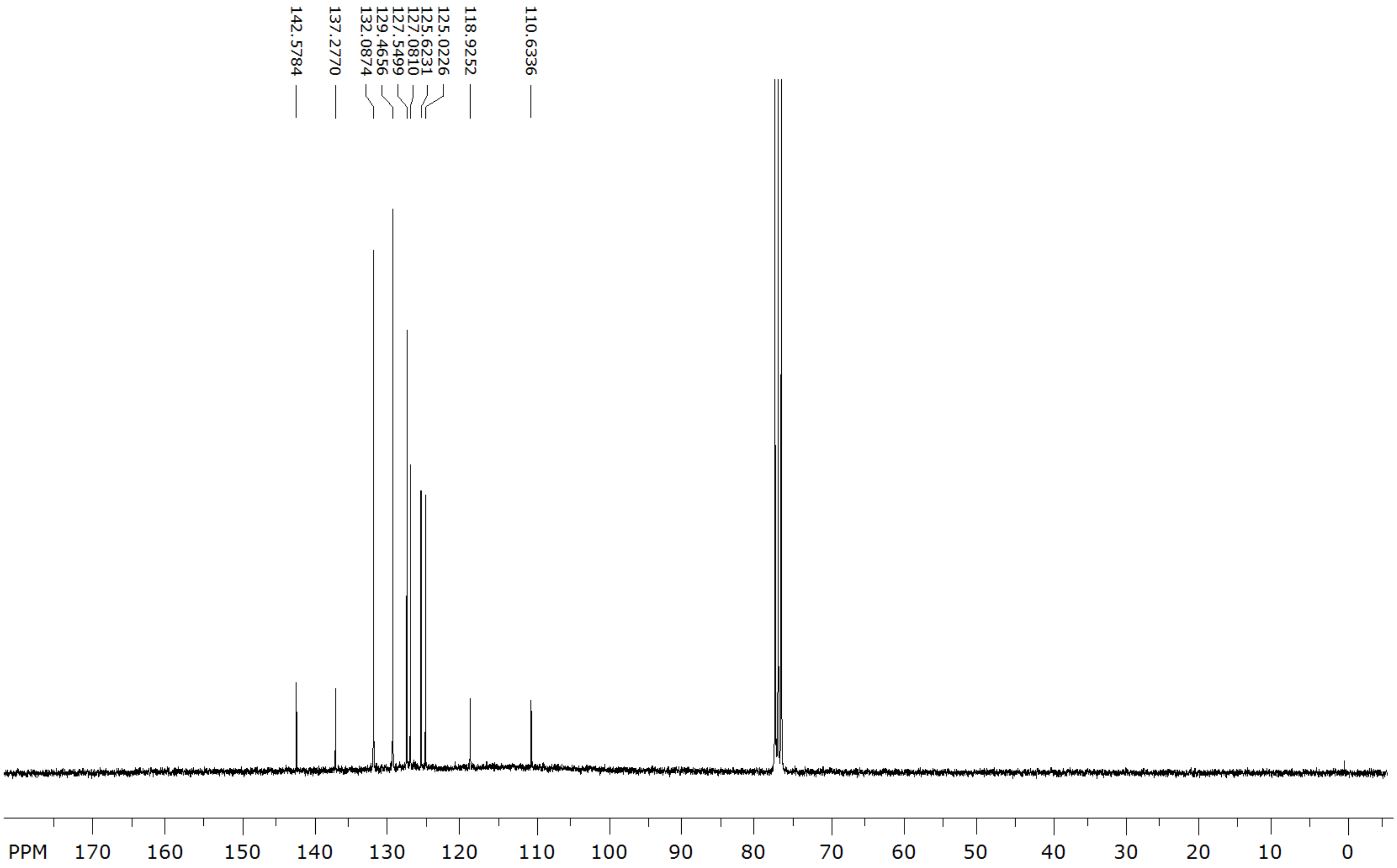

Figure S79. ^{13}C NMR ($CDCl_3$) spectrum of *cis*-**63**.

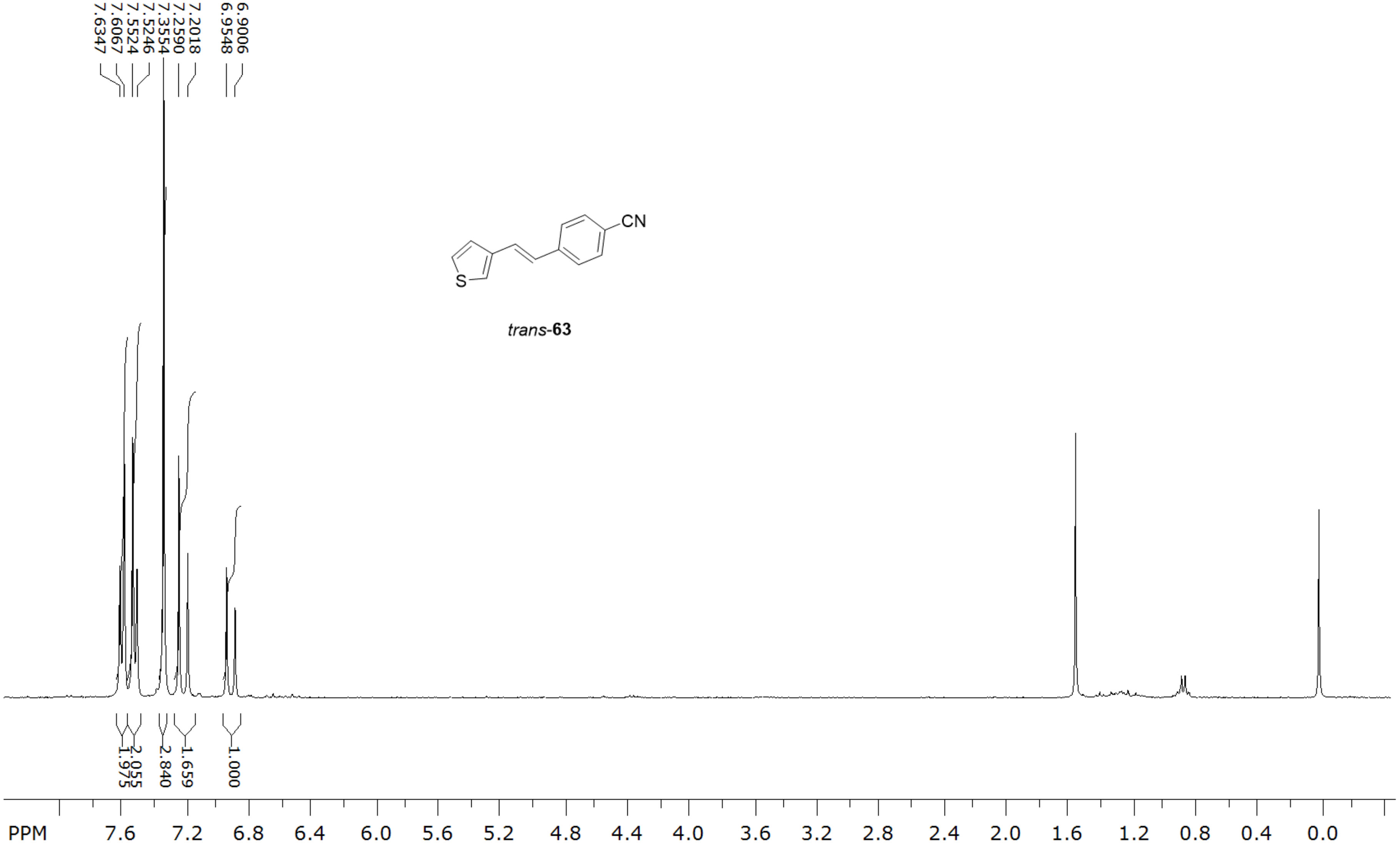

Figure S80. ^{1}H NMR ($CDCl_3$) spectrum of *trans*-**63**.

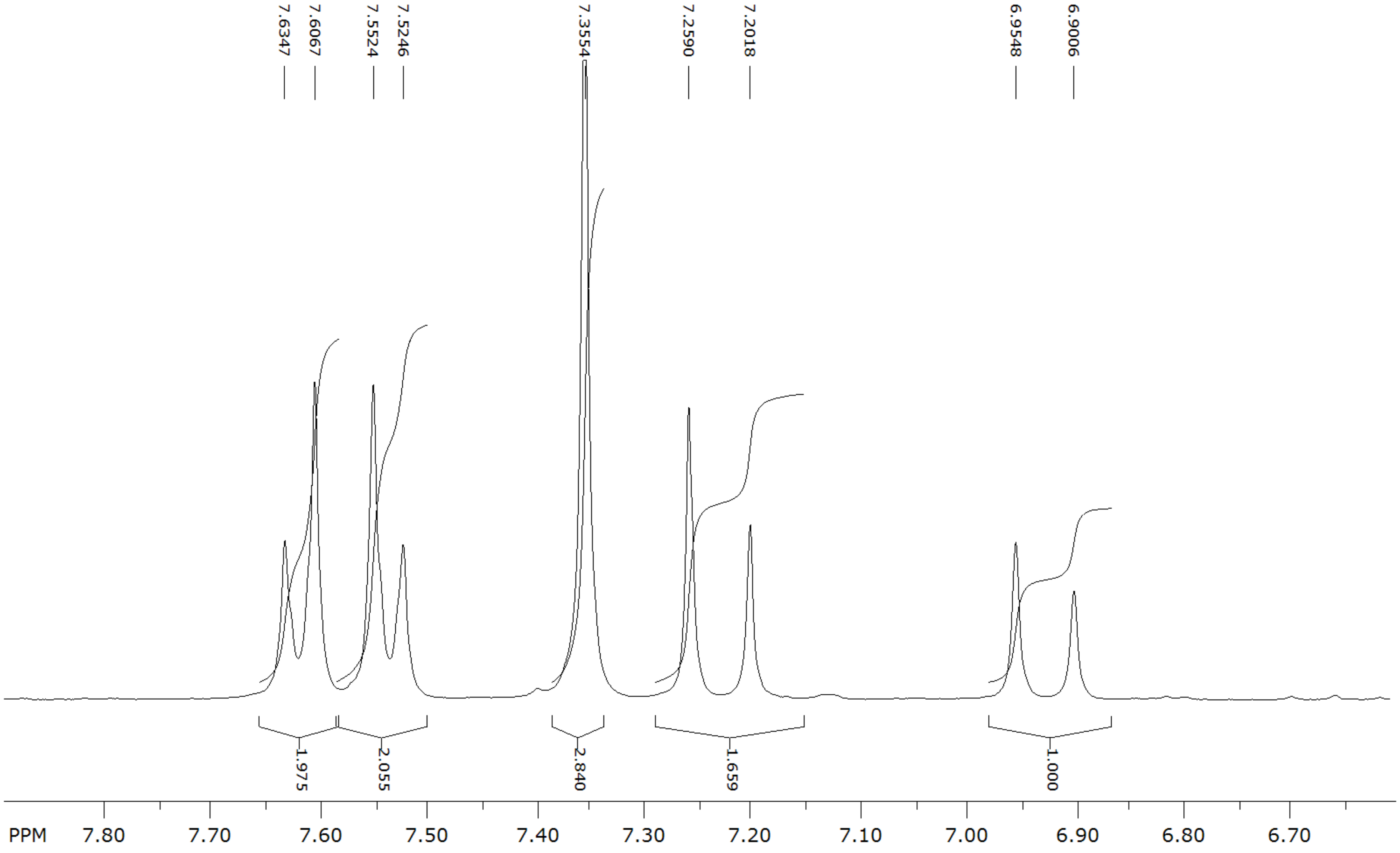

Figure S81. ^{1}H NMR ($CDCl_3$) spectrum of aromatic part of *trans*-**63**.

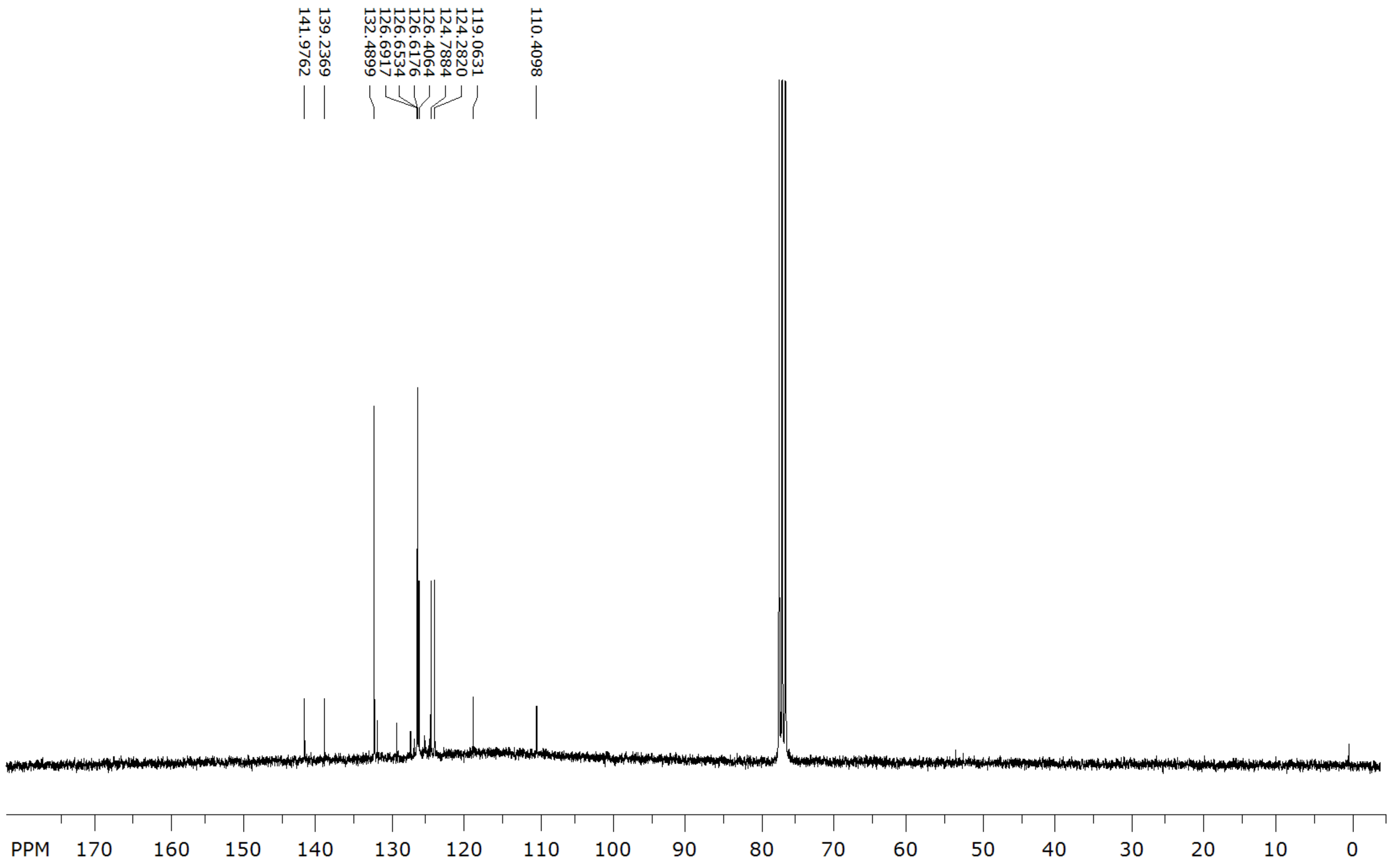

Figure S82. ^{13}C NMR ($CDCl_3$) spectrum of *trans*-**63**.

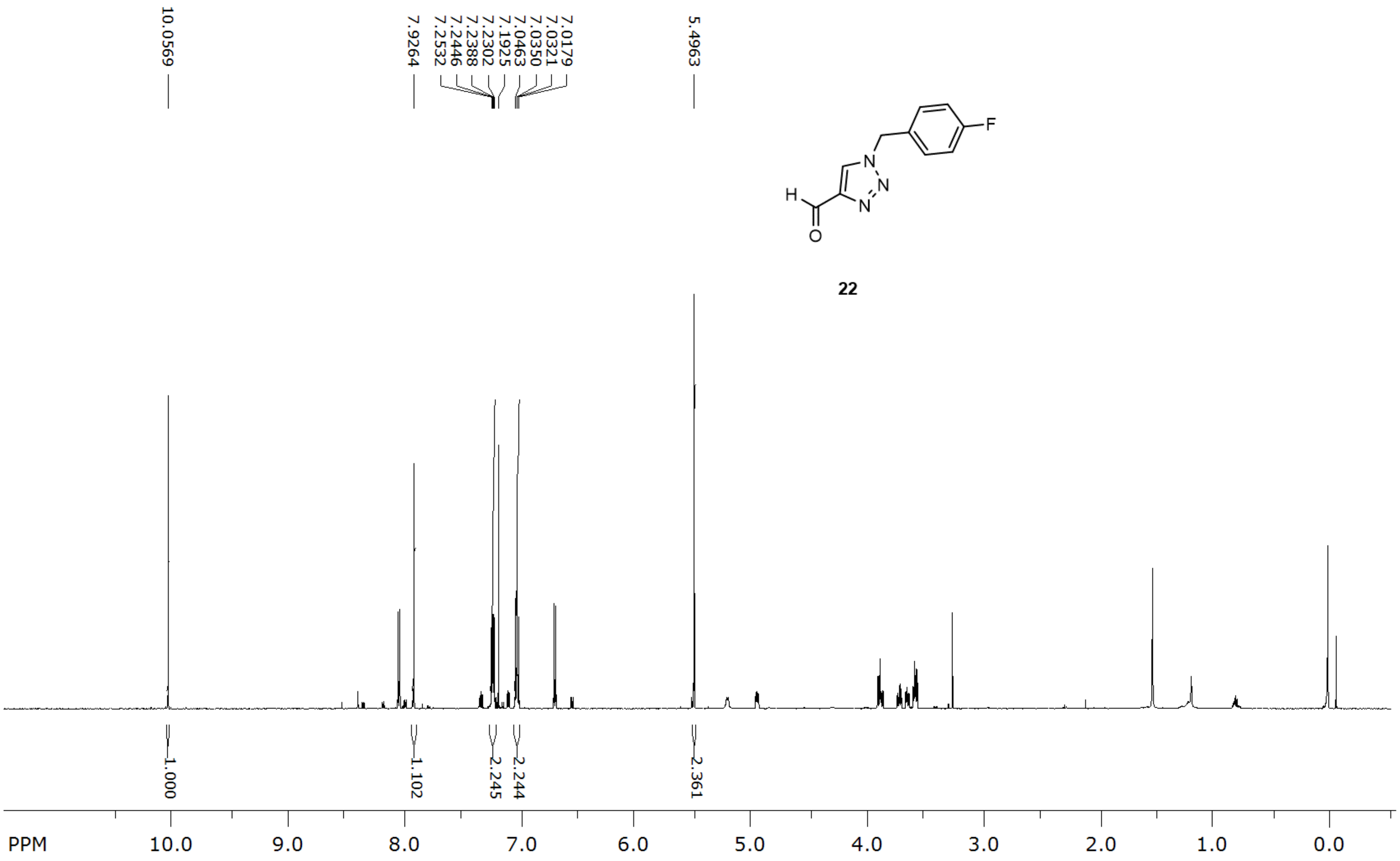

Figure S83. ^{1}H NMR ($CDCl_3$) spectrum of **22**.

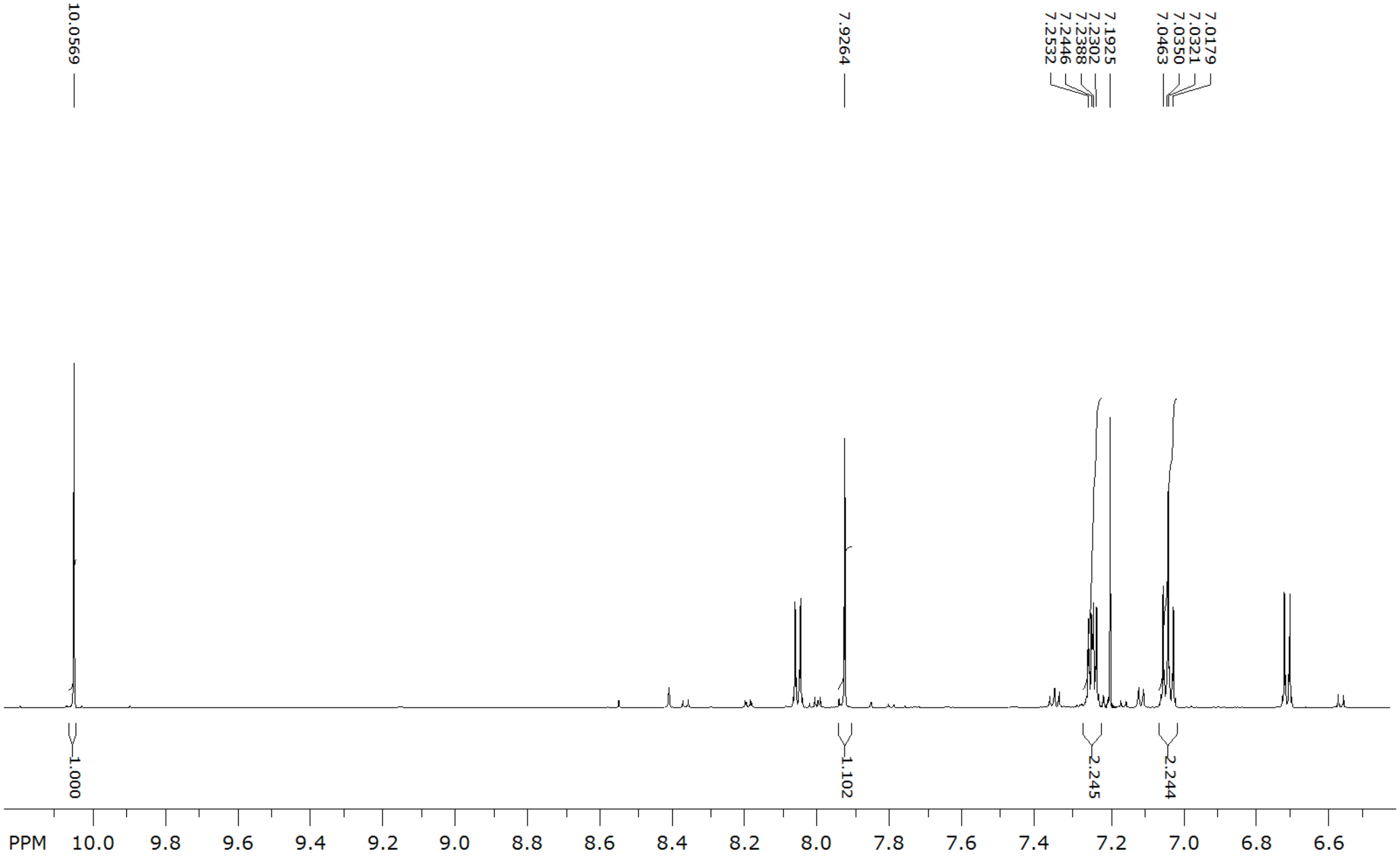

Figure S84. ^{1}H NMR ($CDCl_3$) spectrum of aromatic part of **22**.

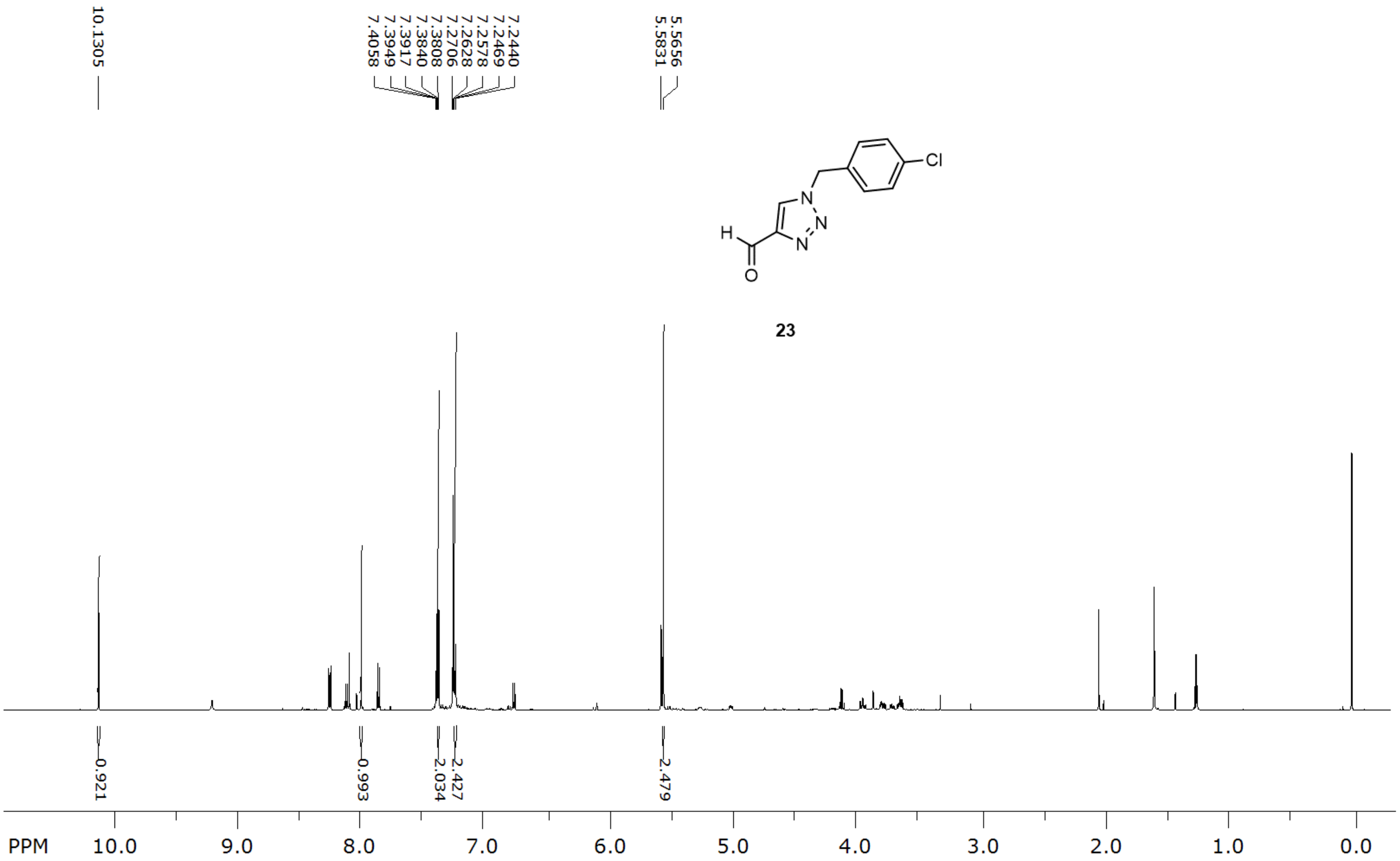

Figure S85. ^{1}H NMR ($CDCl_3$) spectrum of **23**.

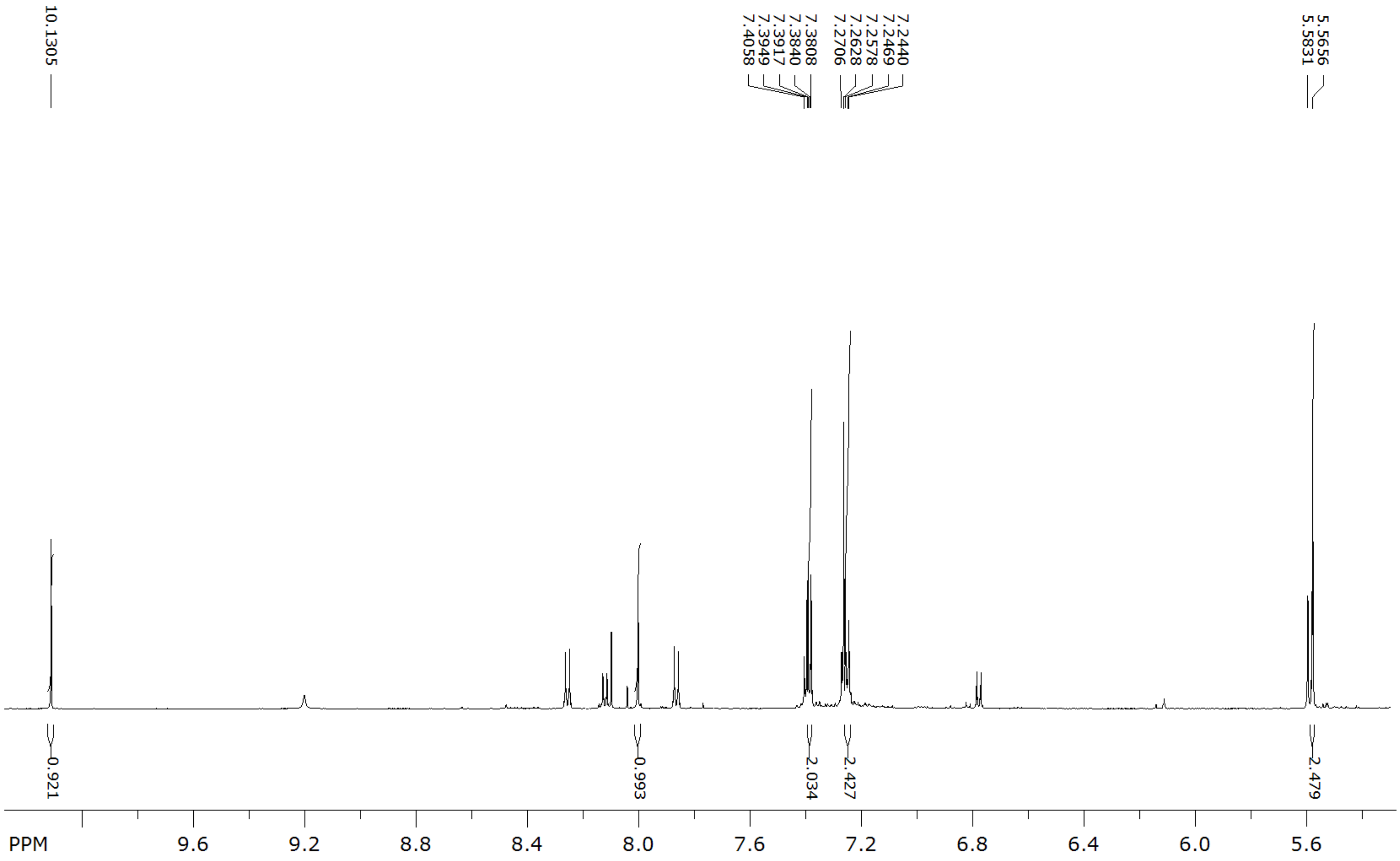

Figure S86. ^{1}H NMR ($CDCl_3$) spectrum of aromatic part of **23**.

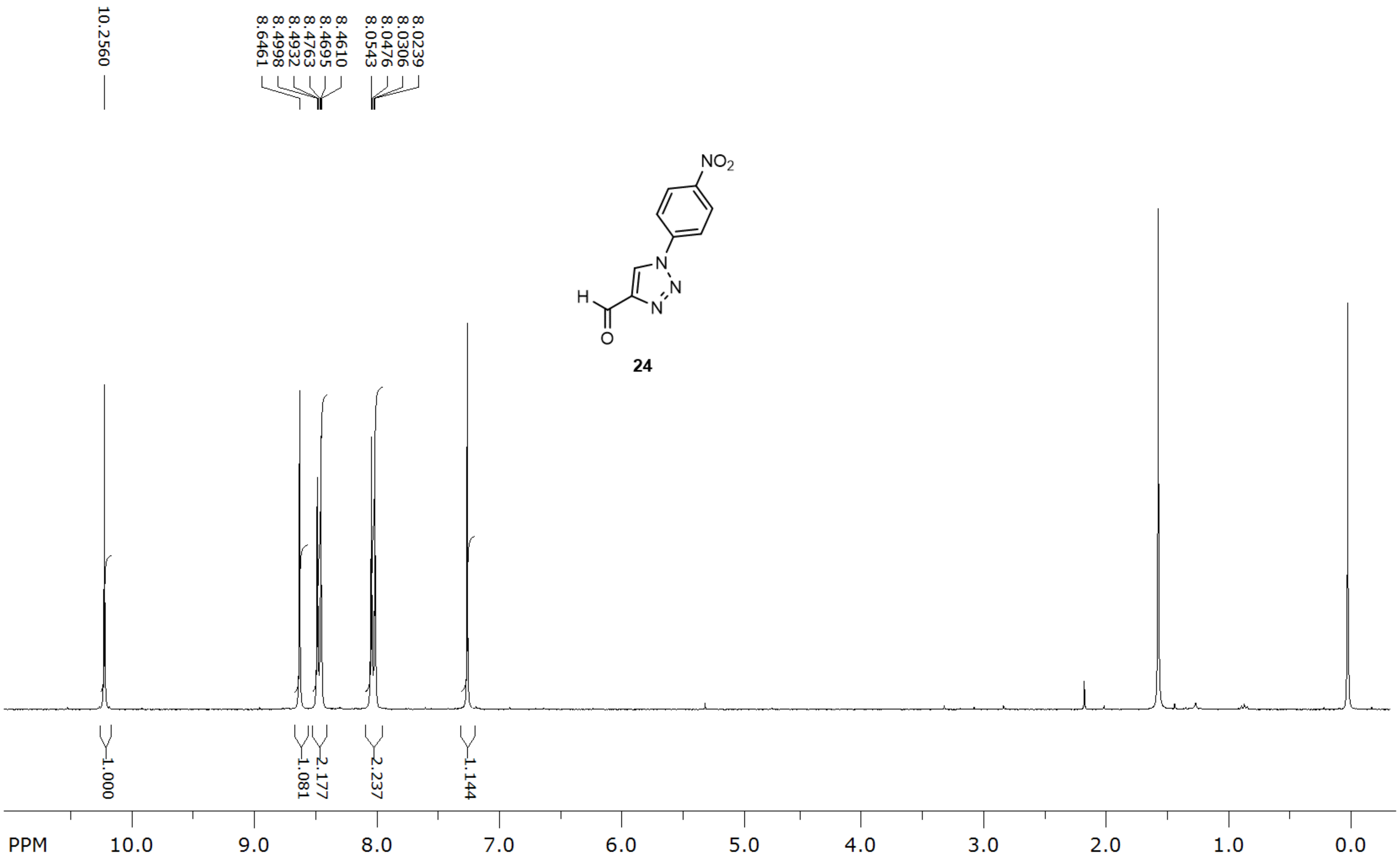

Figure S87. ^{1}H NMR ($CDCl_3$) spectrum of **24**.

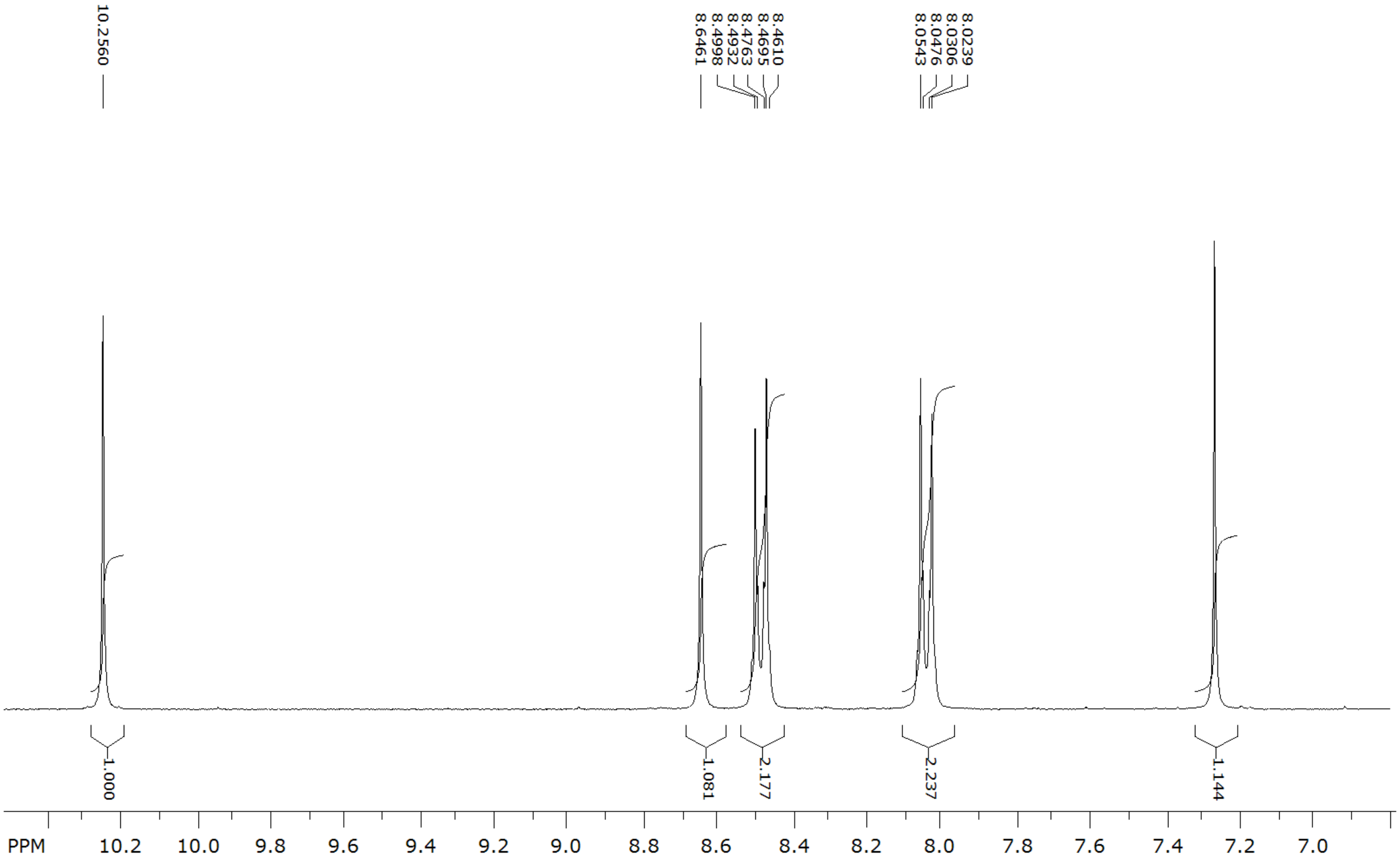

Figure S88. ^{1}H NMR ($CDCl_3$) spectrum of aromatic part of **24**.

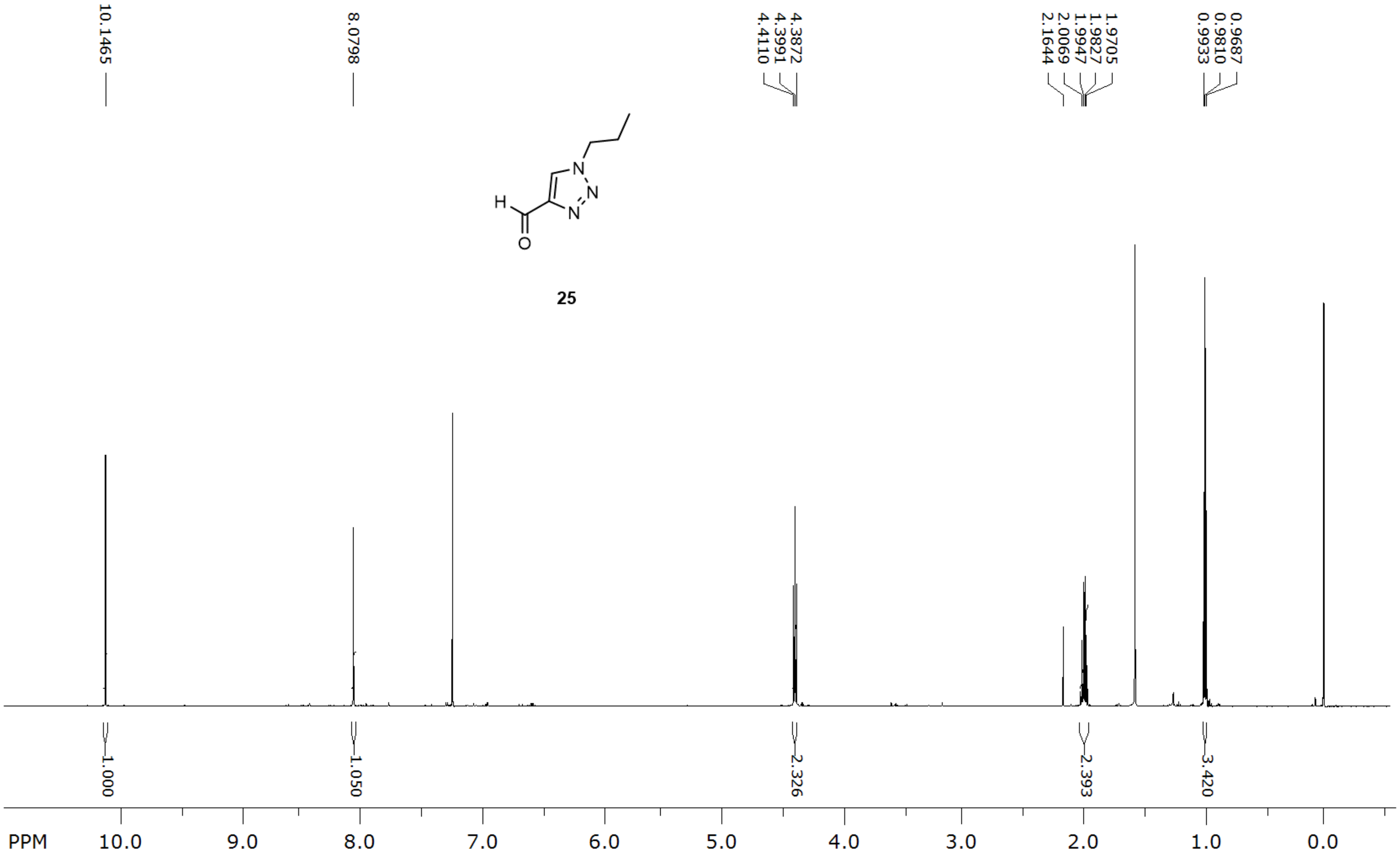

Figure S89. ^{1}H NMR ($CDCl_3$) spectrum of **25**.

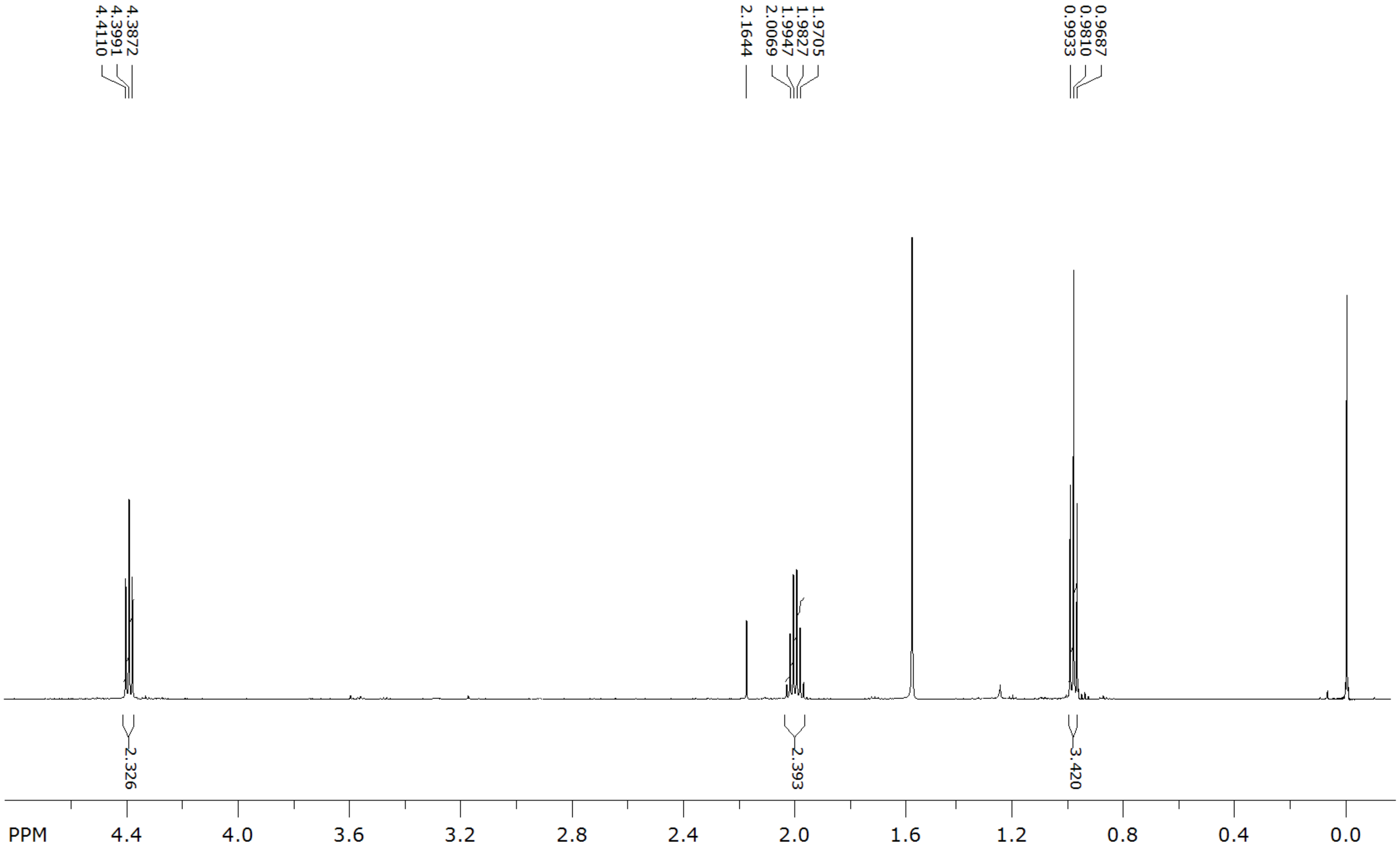

Figure S90. ^{1}H NMR ($CDCl_3$) spectrum of aliphatic part of **25**.

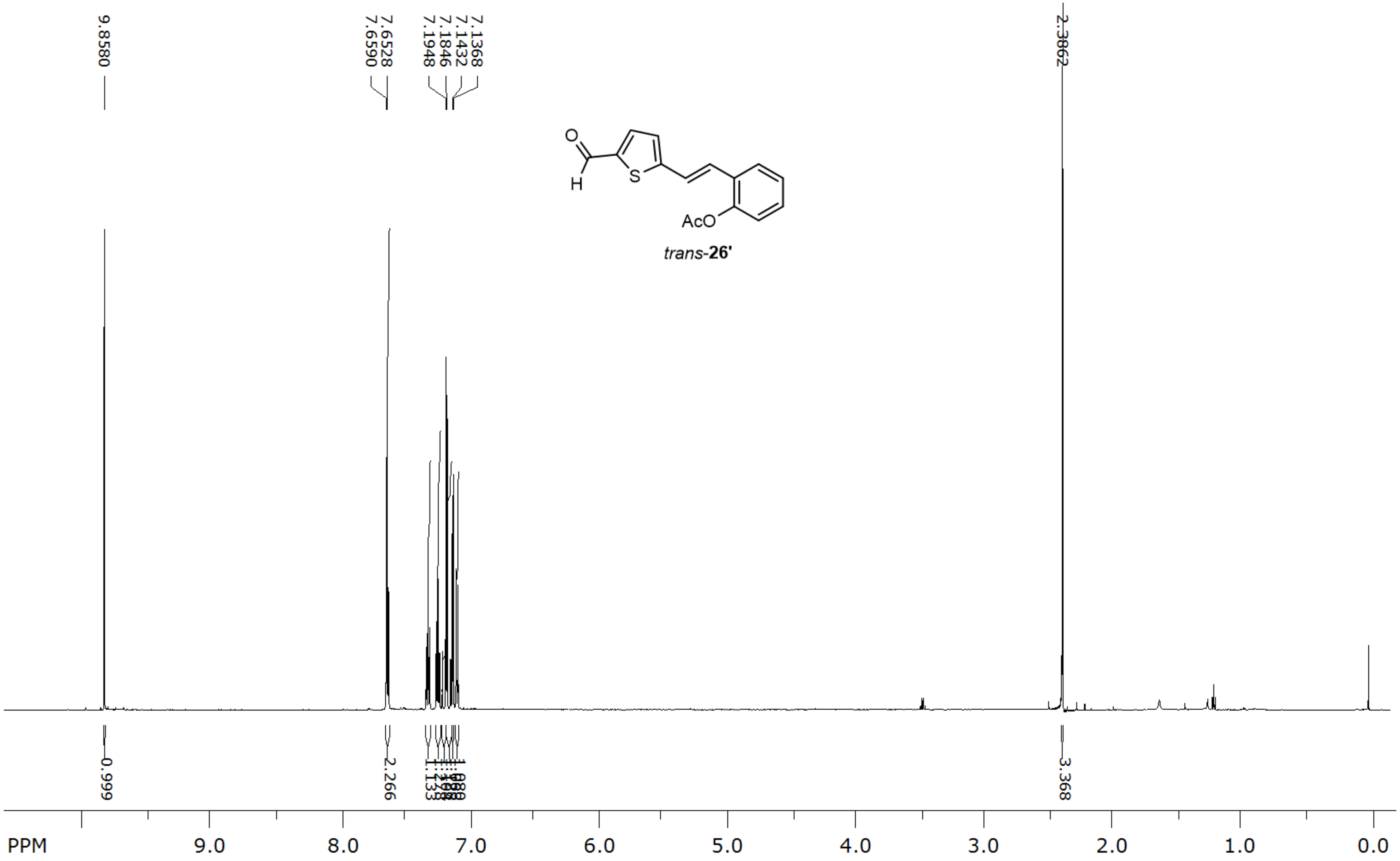

Figure S91. ^{1}H NMR ($CDCl_3$) spectrum of *trans*-**26'**.

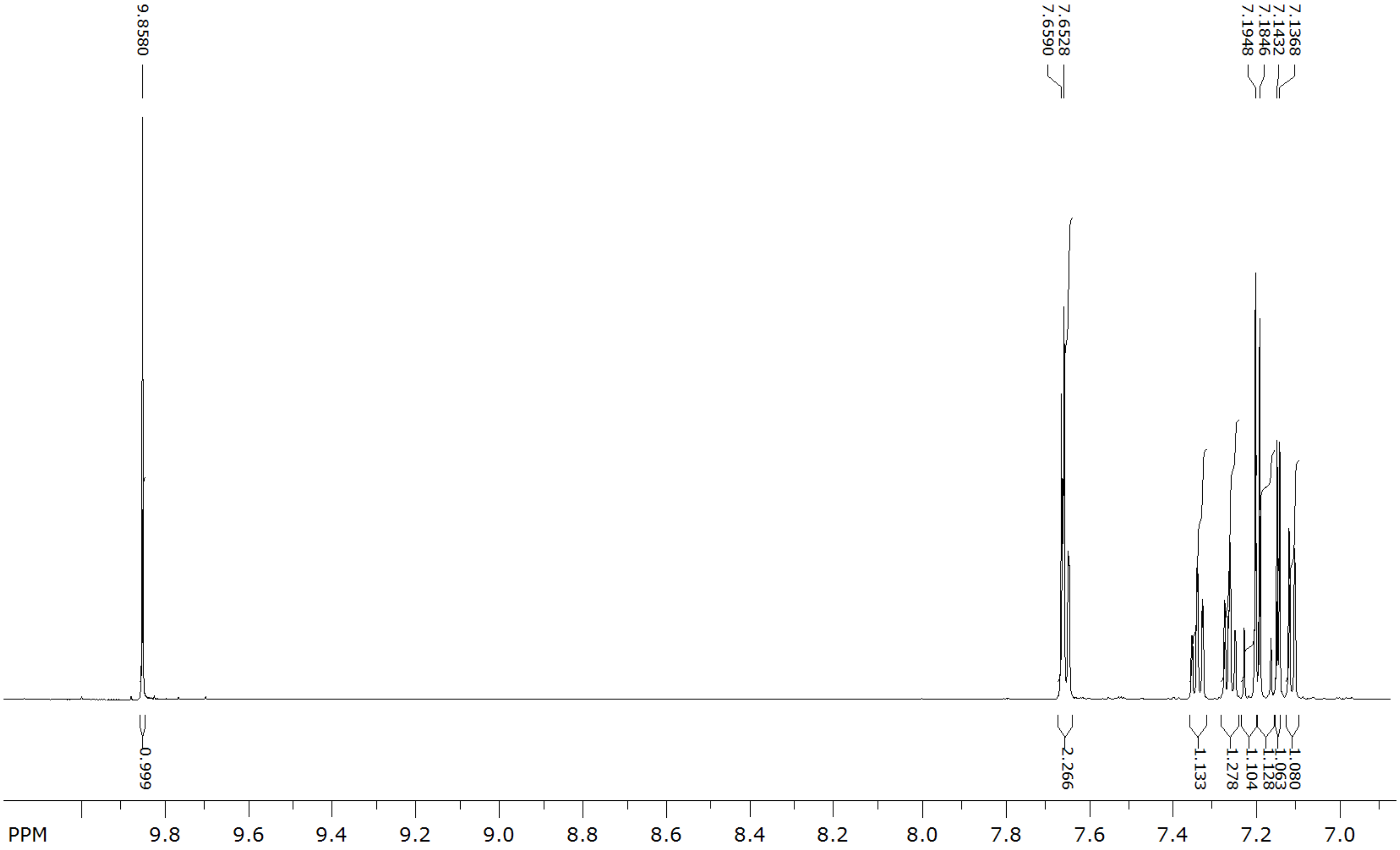

Figure S92. ^{1}H NMR ($CDCl_3$) spectrum of aromatic part of *trans*-**26'**.

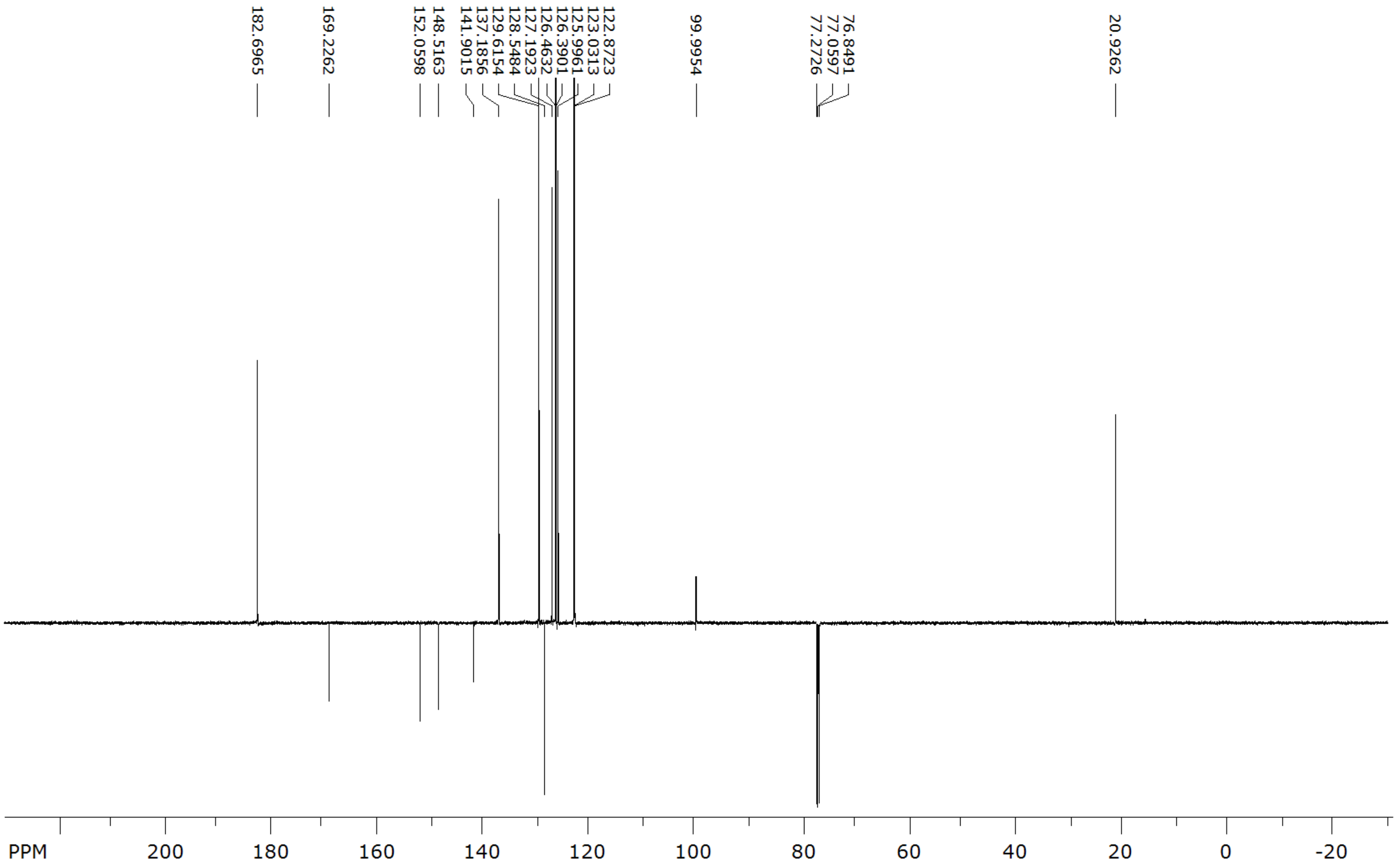

Figure S93. ^{13}C NMR ($CDCl_3$) spectrum of *trans*-**26'**.

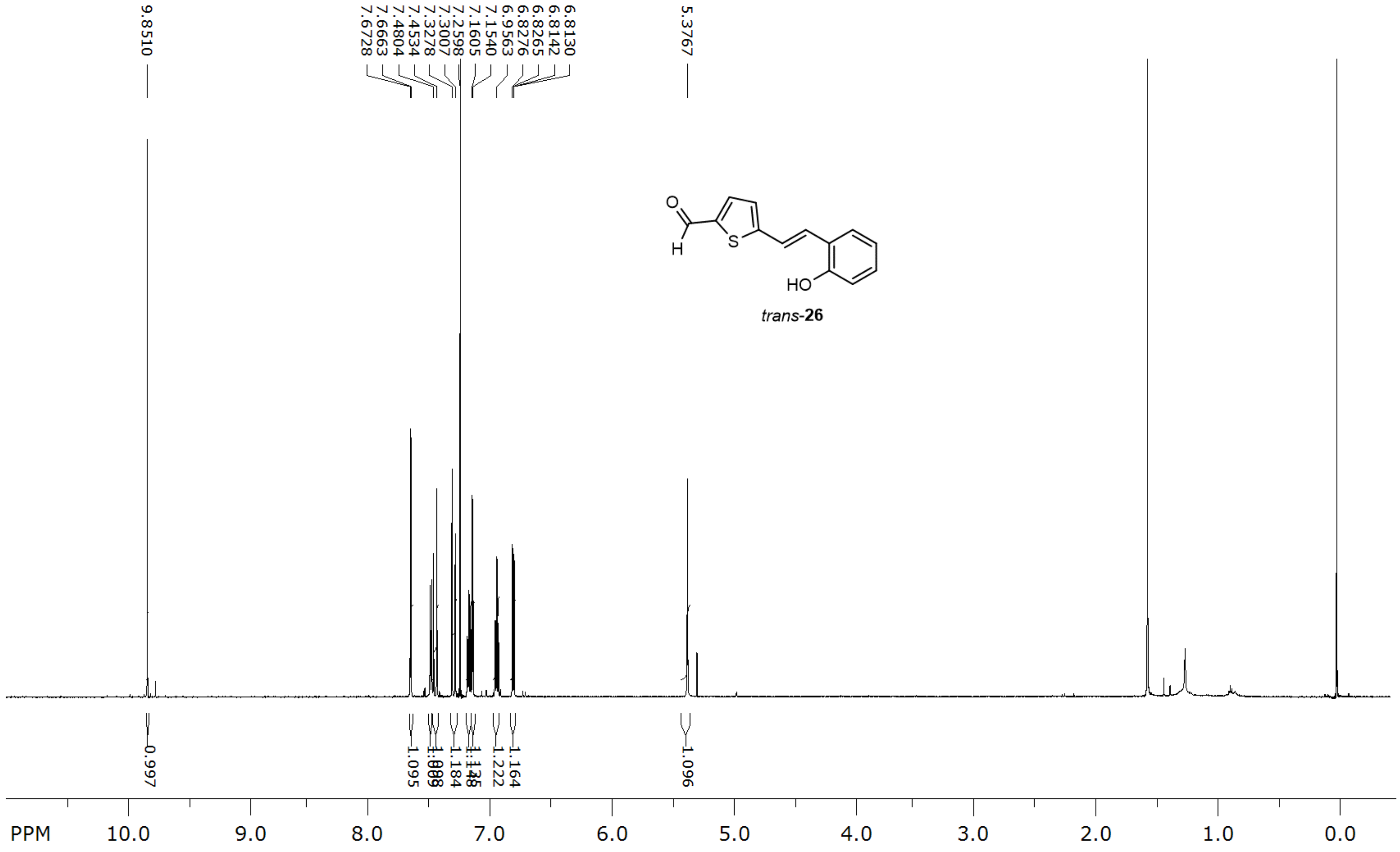

Figure S94. ^{1}H NMR ($CDCl_3$) spectrum of *trans*-**26**.

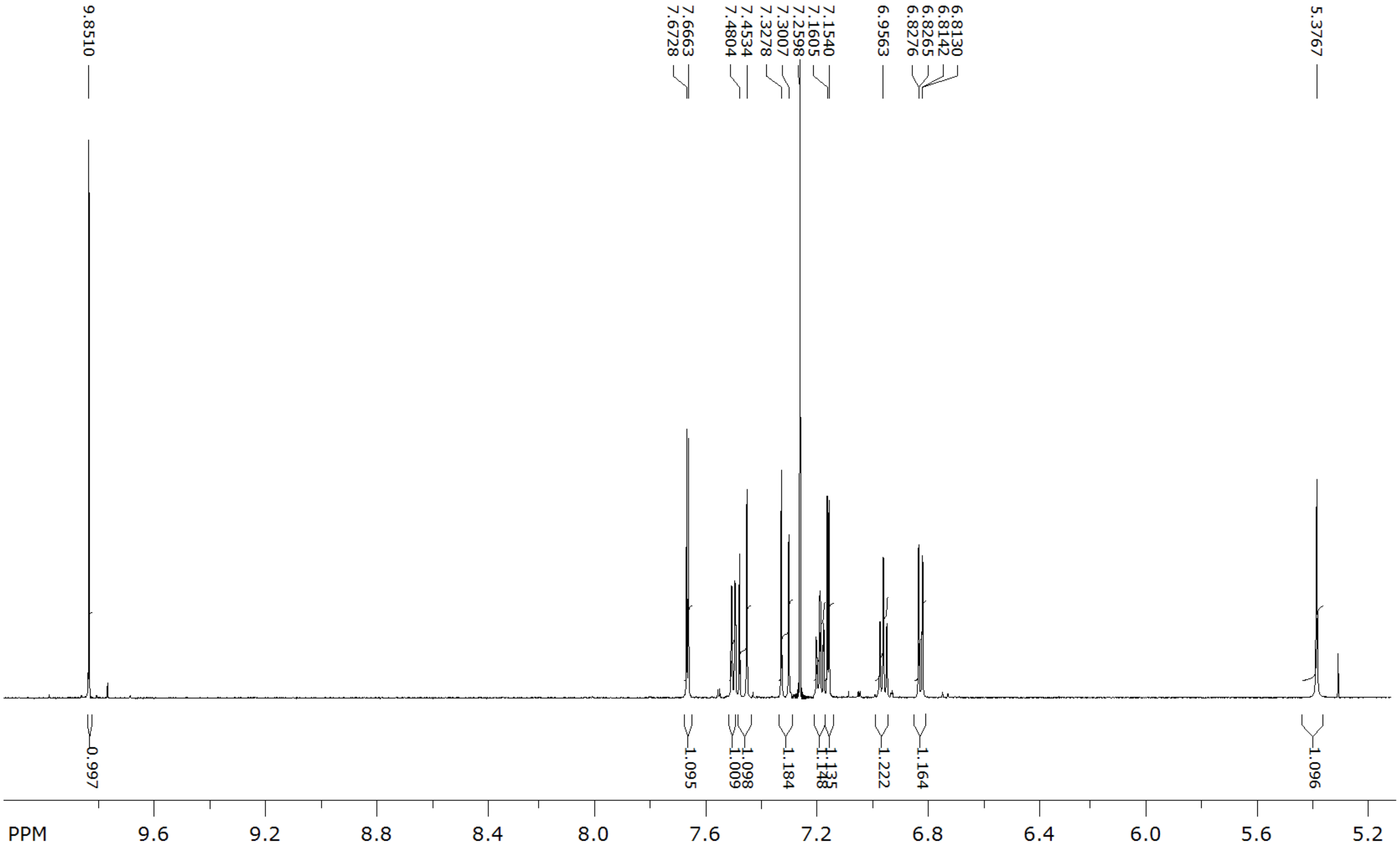

Figure S95. ^{1}H NMR ($CDCl_3$) spectrum of aromatic part of *trans*-**26**.

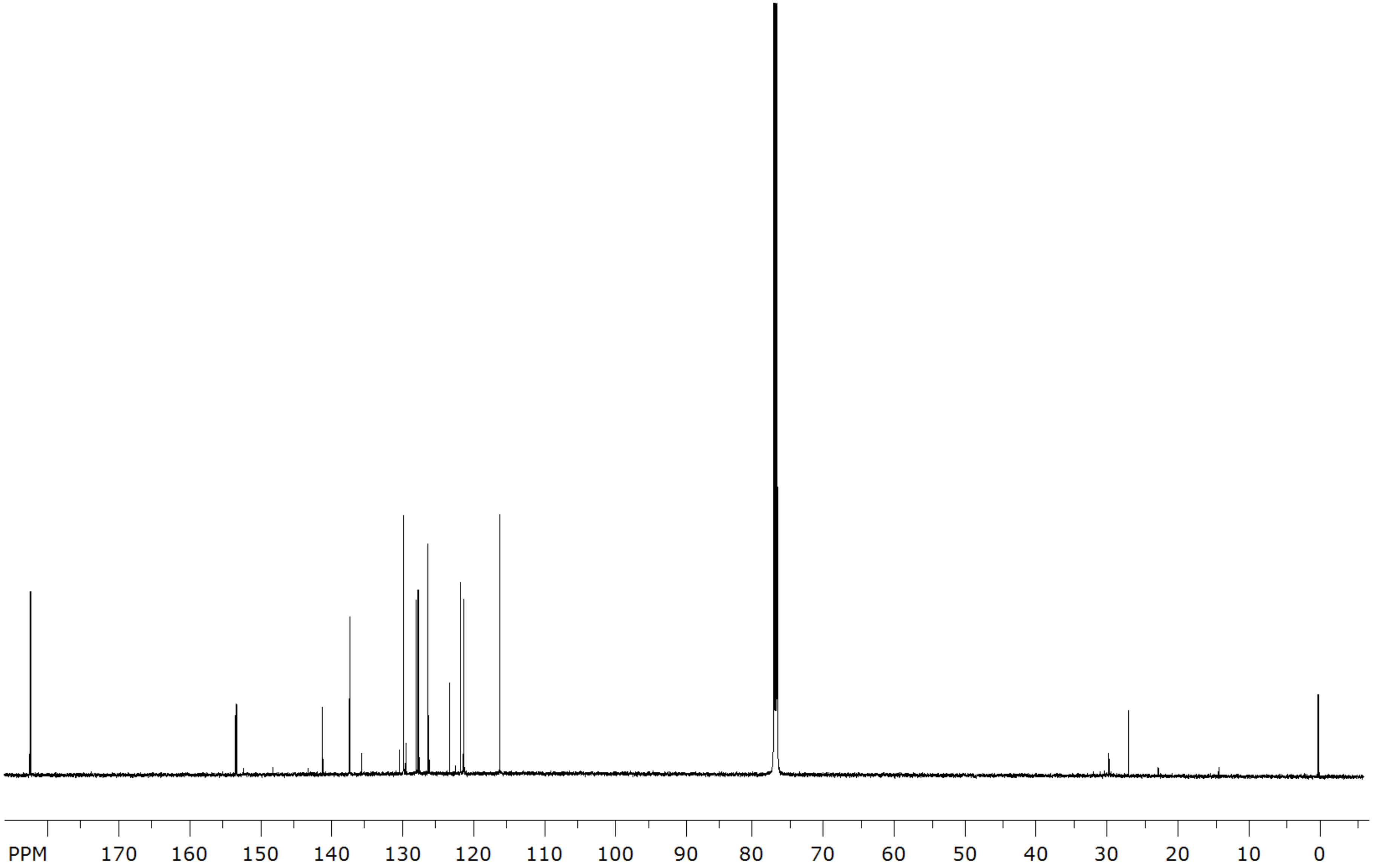

Figure S96. ^{13}C NMR ($CDCl_3$) spectrum of *trans*-**26**.

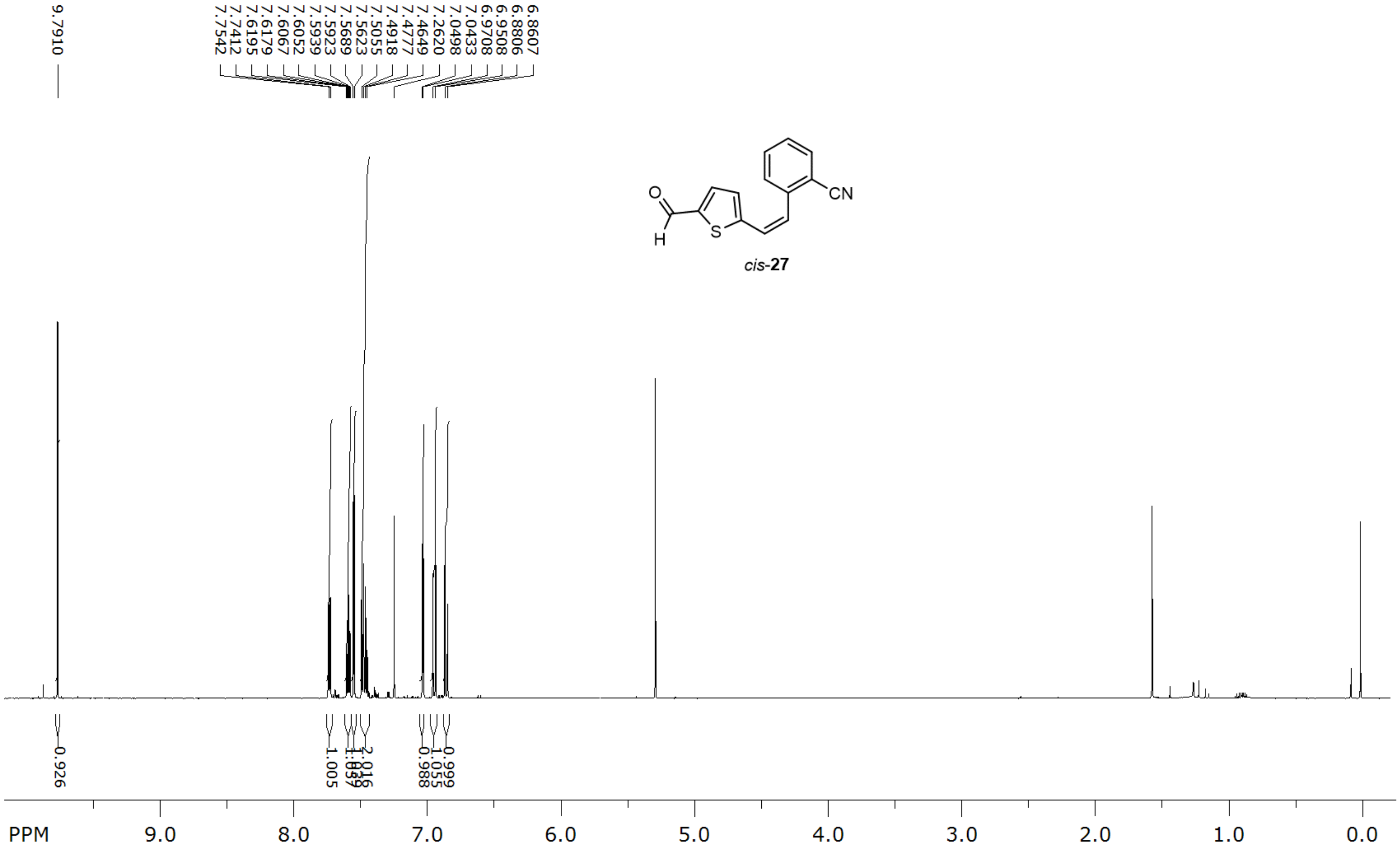

Figure S97. ^1H NMR ($CDCl_3$) spectrum of *cis*-**27**.

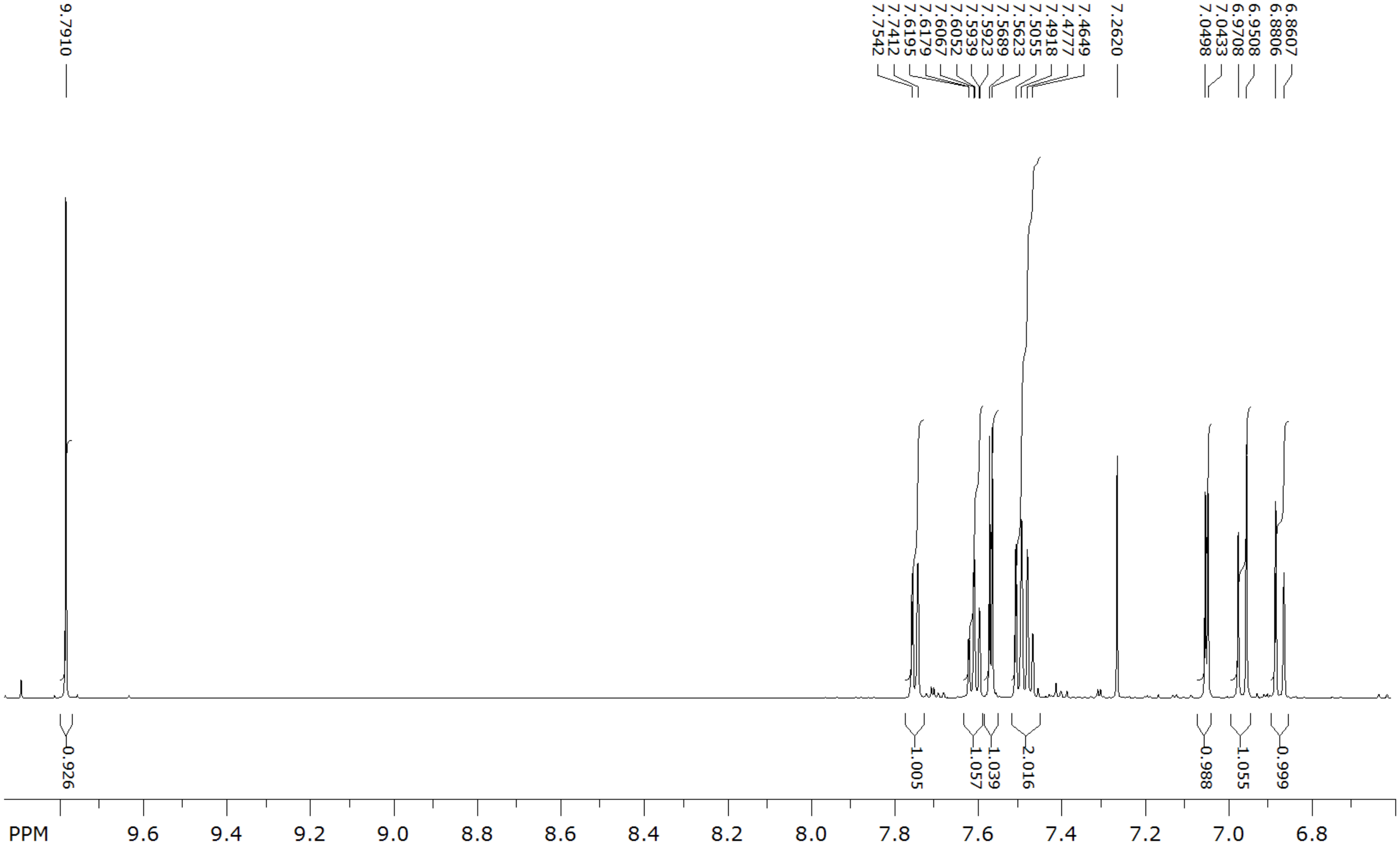

Figure S98. ^{1}H NMR ($CDCl_3$) spectrum of aromatic part of *cis*-**27**.

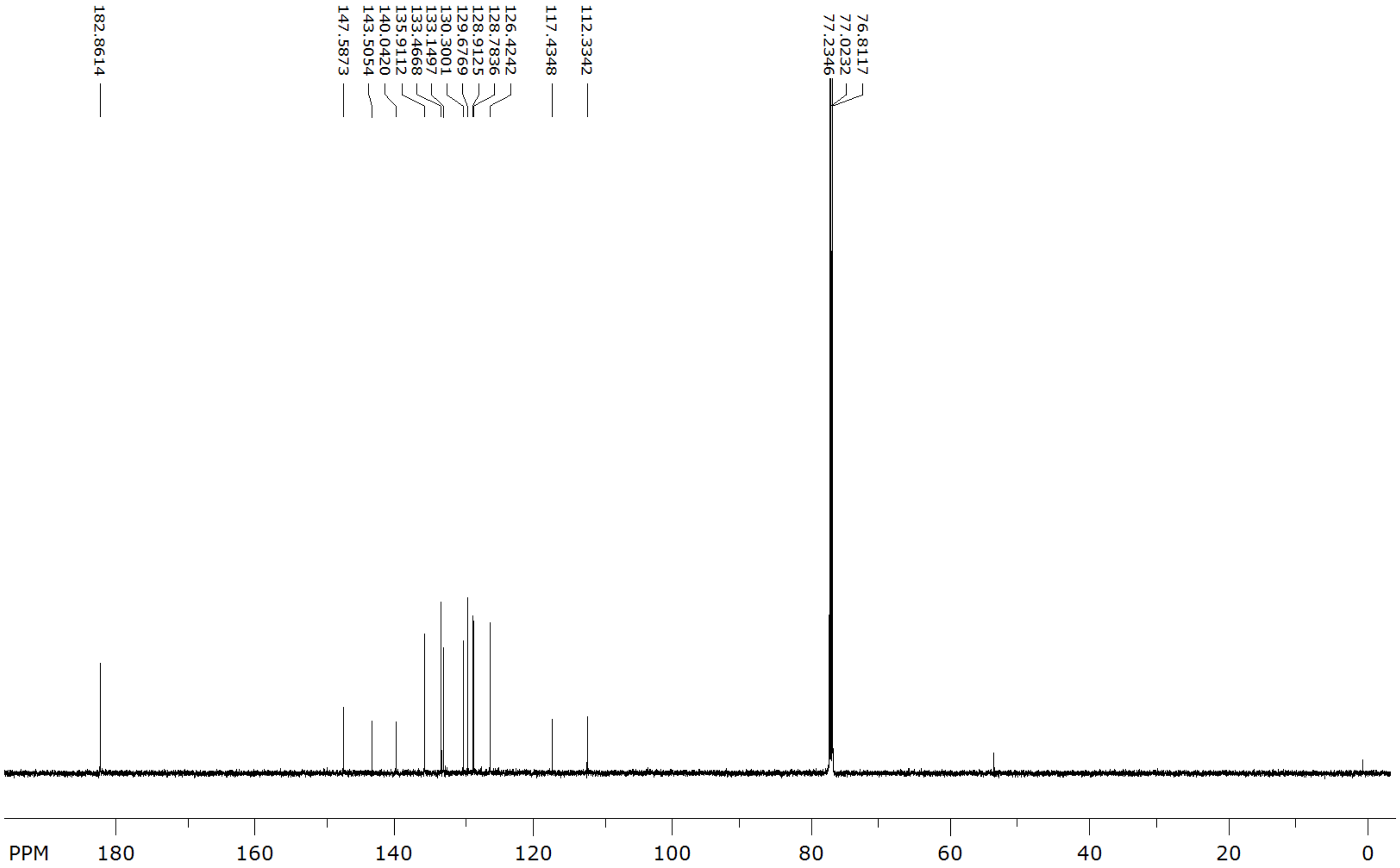

Figure S99. ^{13}C NMR ($CDCl_3$) spectrum of *cis*-**27**.

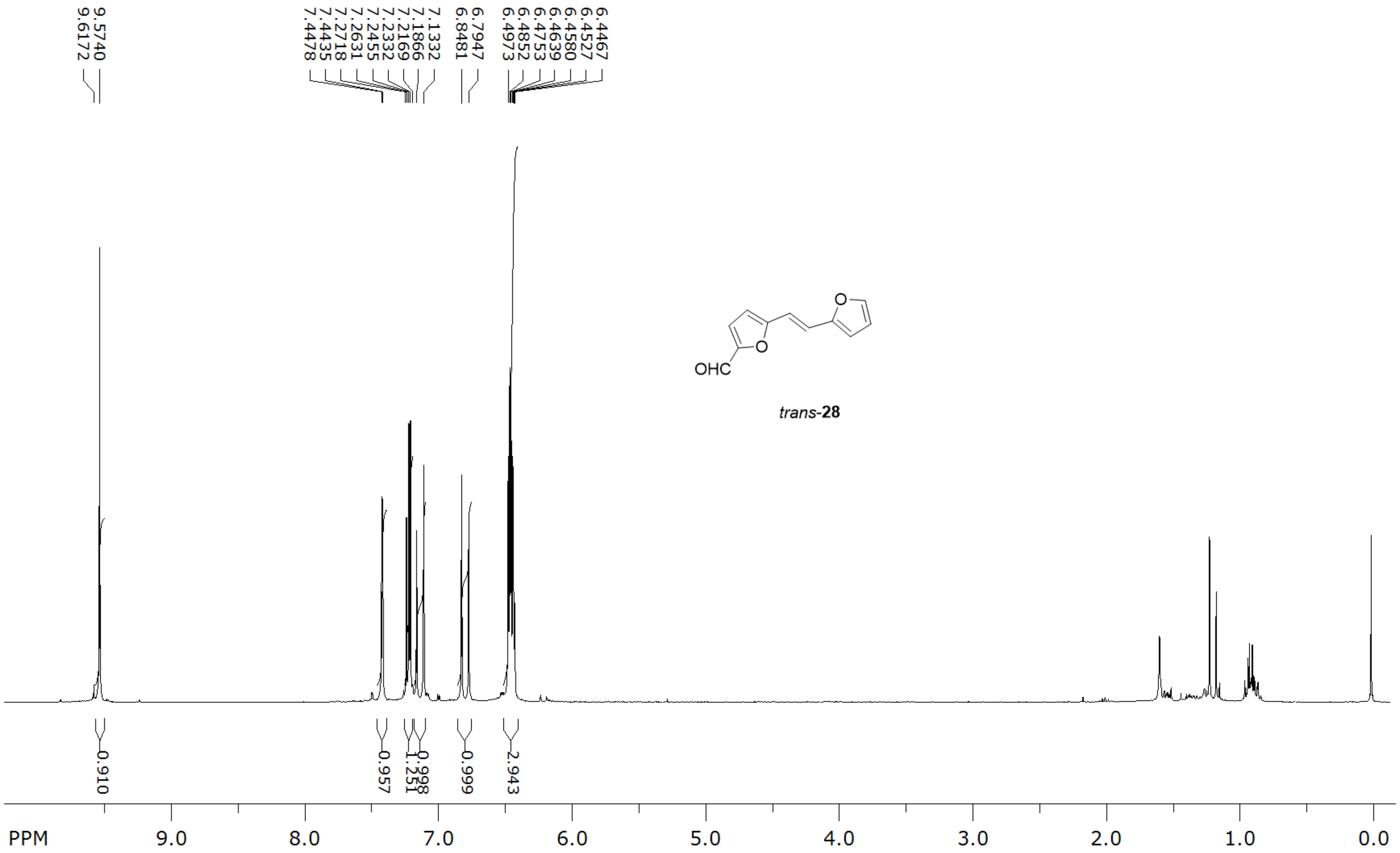

Figure S100. ^{1}H NMR ($CDCl_3$) spectrum of *trans*-**28**.

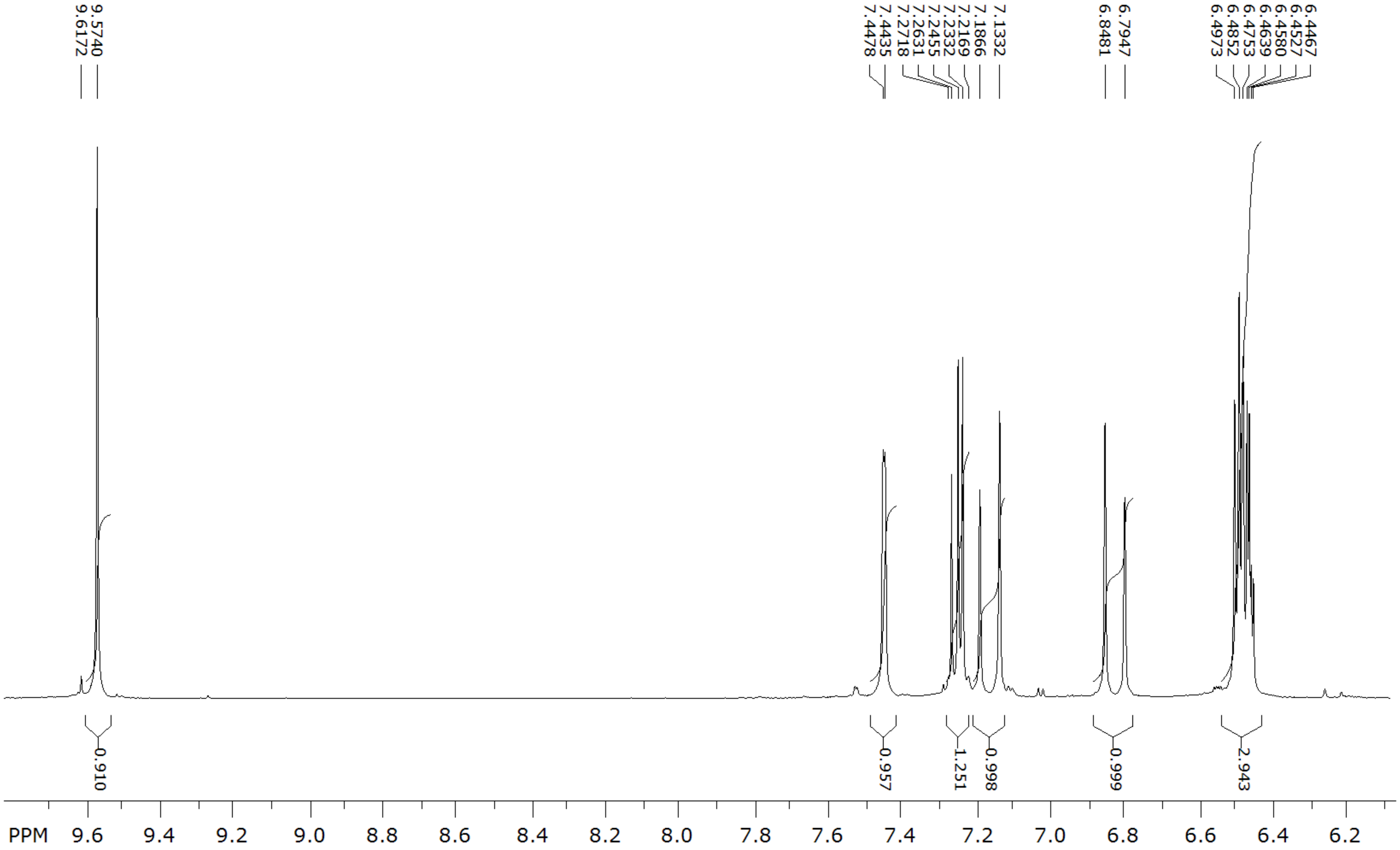

Figure S101. ^{1}H NMR ($CDCl_3$) spectrum of aromatic part of *trans*-**28**.

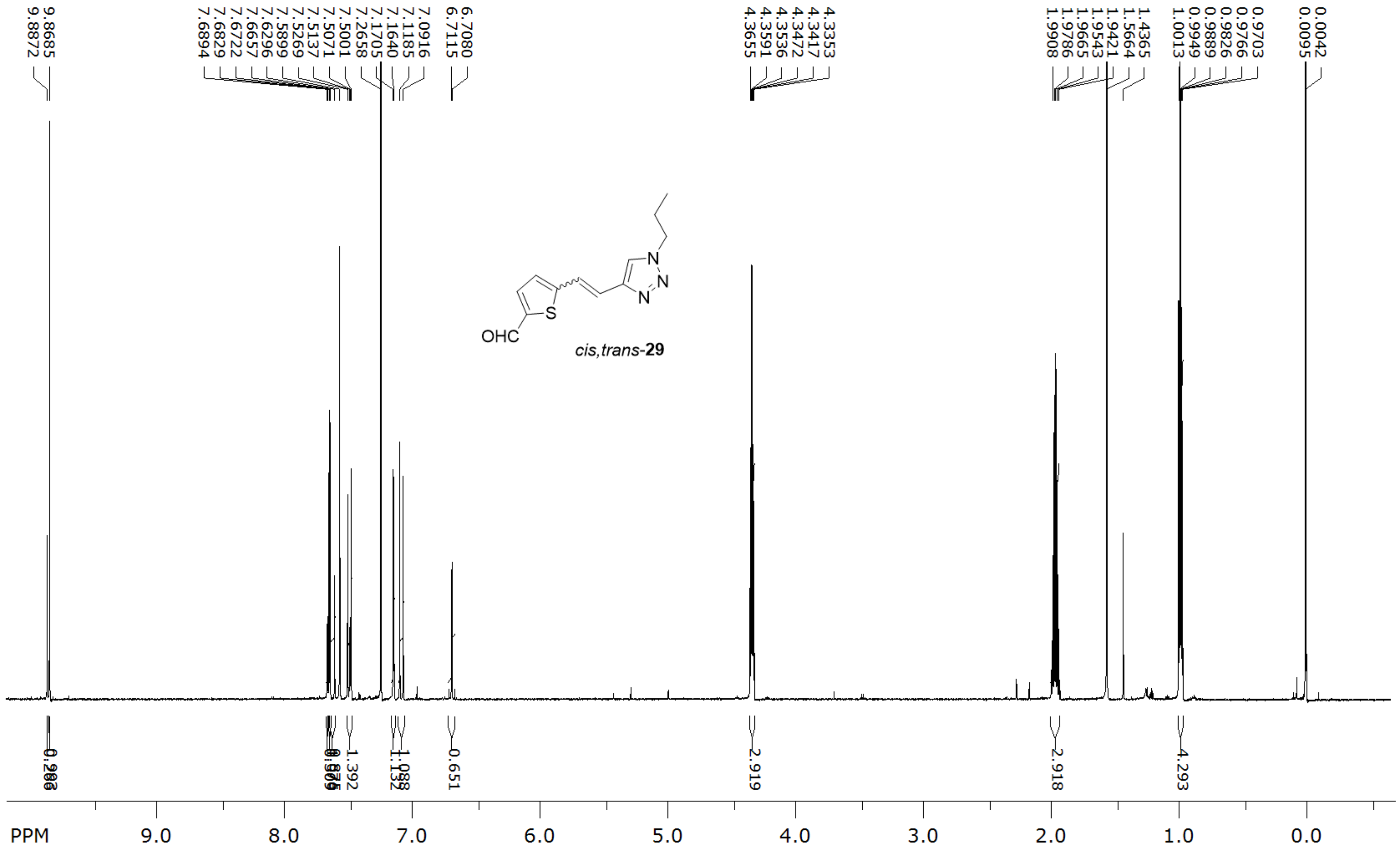

Figure S102. ^{1}H NMR ($CDCl_3$) spectrum of *cis,trans*-**29**.

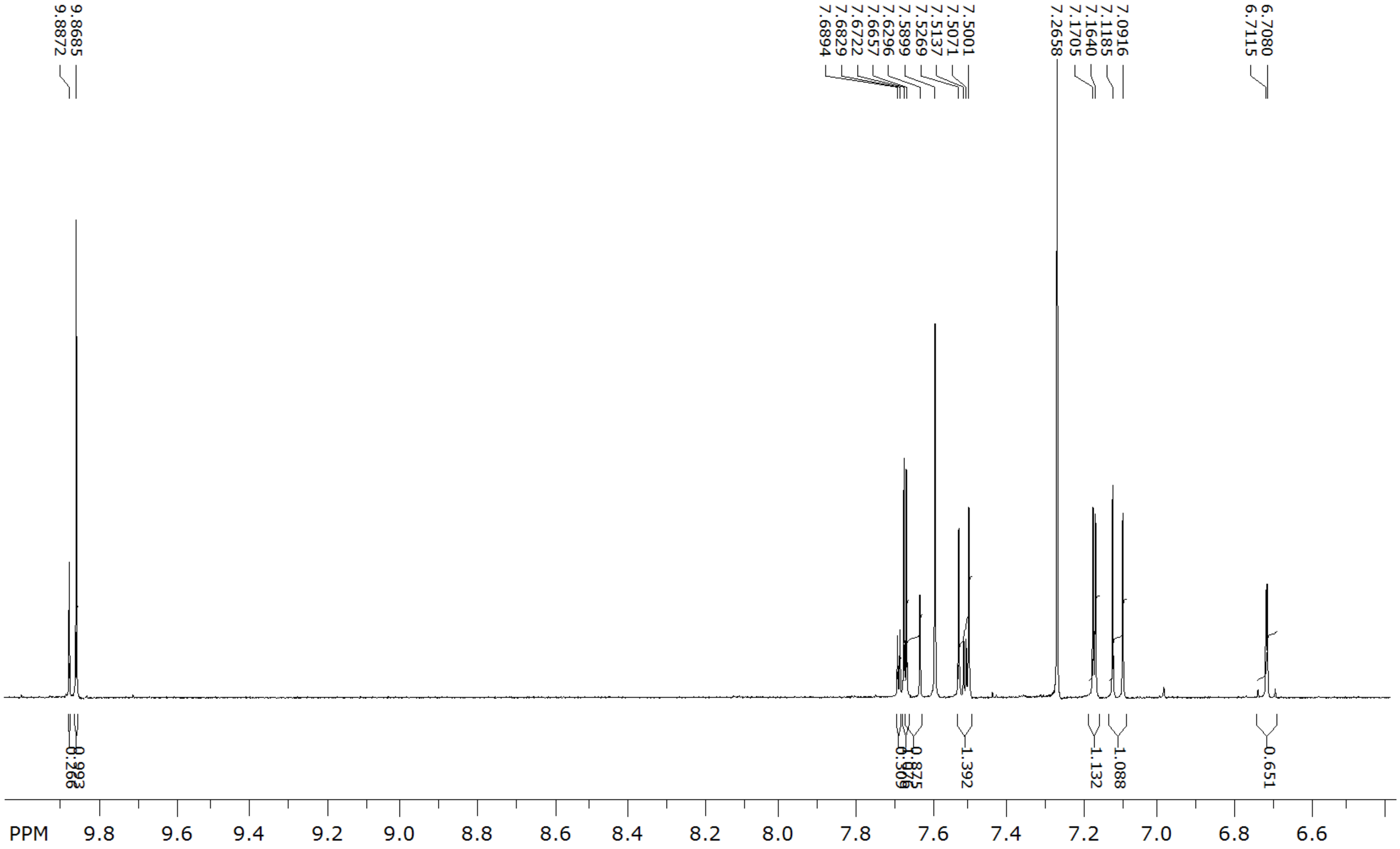

Figure S103. ^{1}H NMR ($CDCl_3$) spectrum of aromatic part of *cis,trans*-**29**.

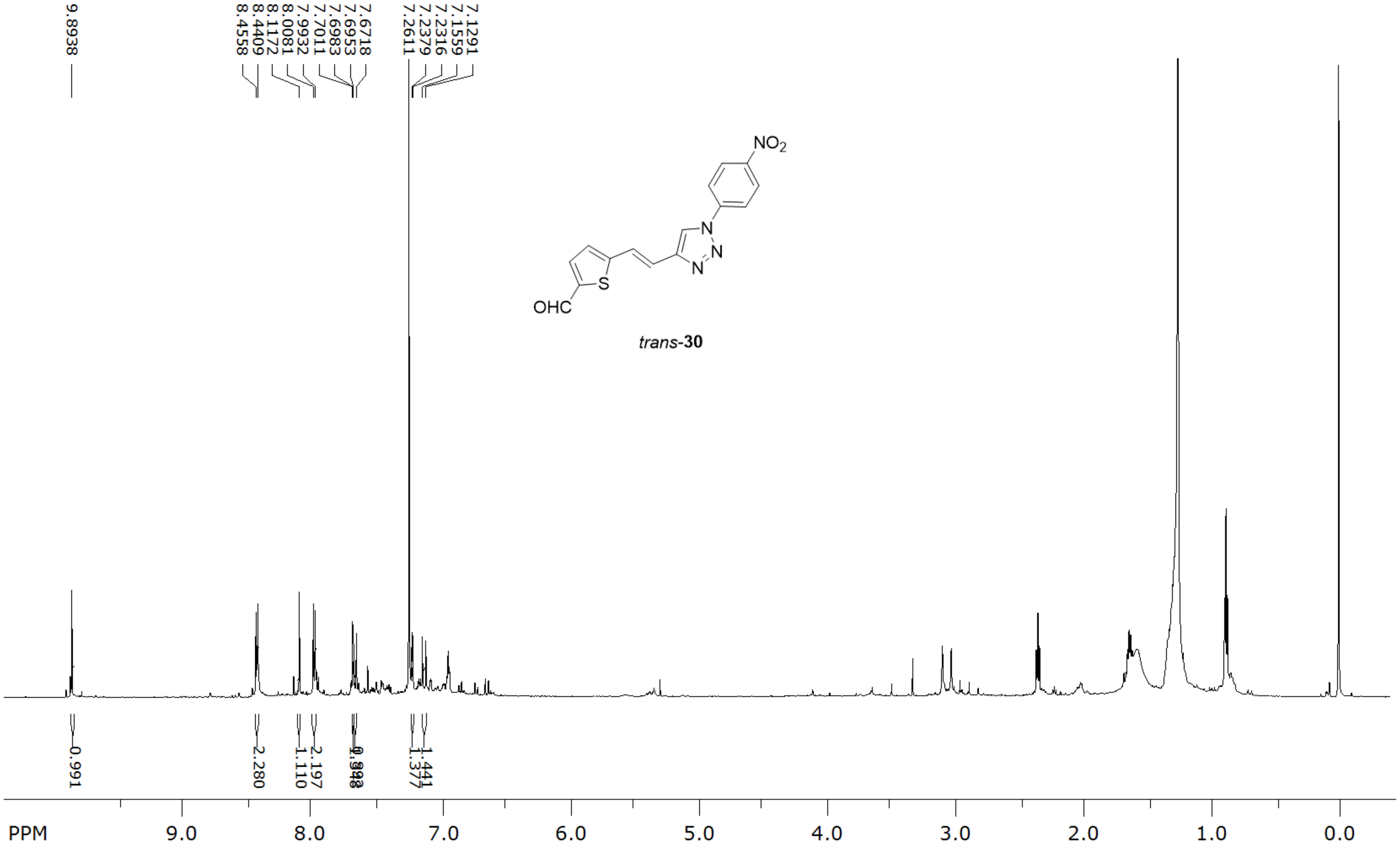

Figure S104. ^{1}H NMR ($CDCl_3$) spectrum of *trans*-**30**.

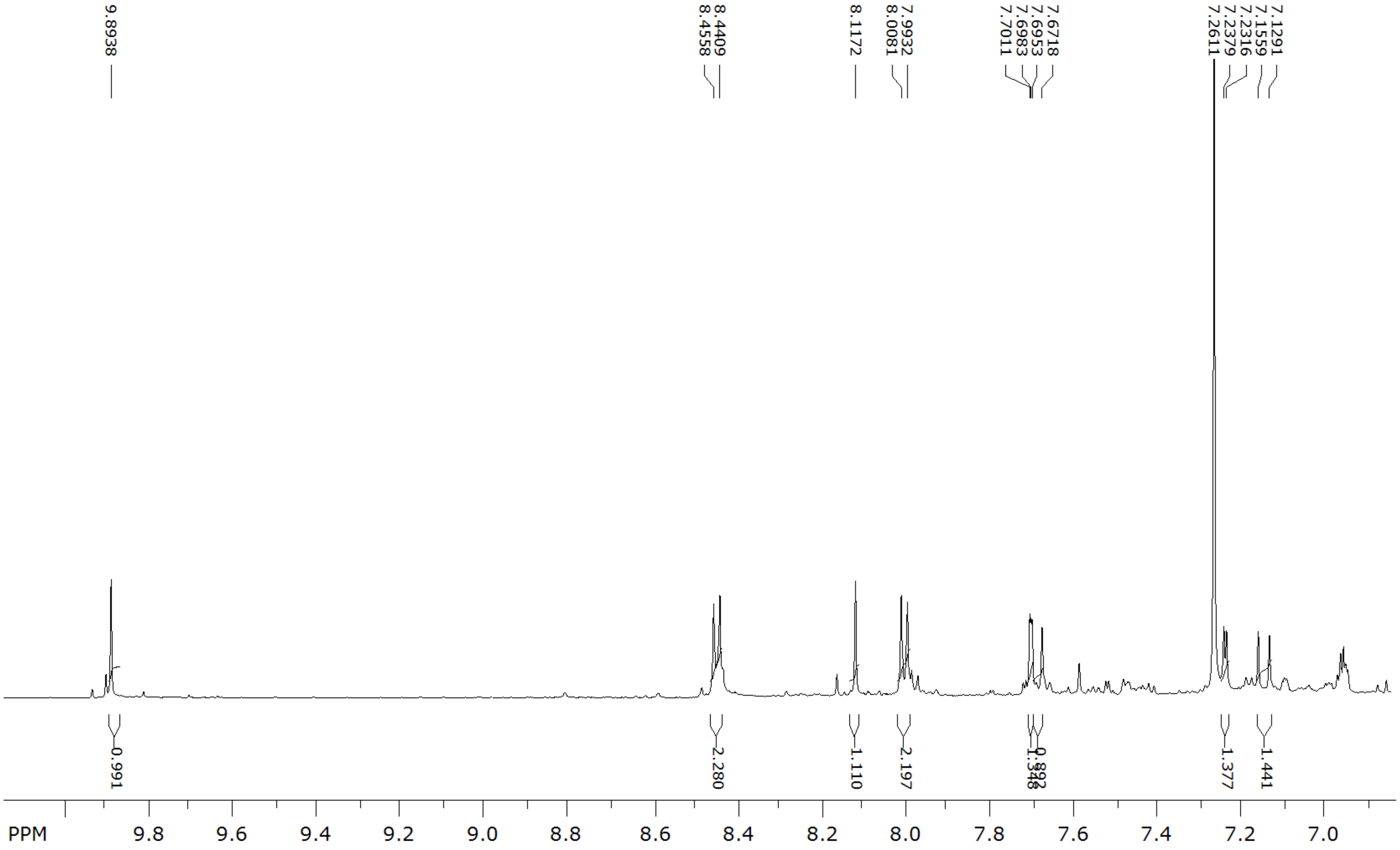

Figure S105. ^{1}H NMR ($CDCl_3$) spectrum of aromatic part of *trans*-**30**.

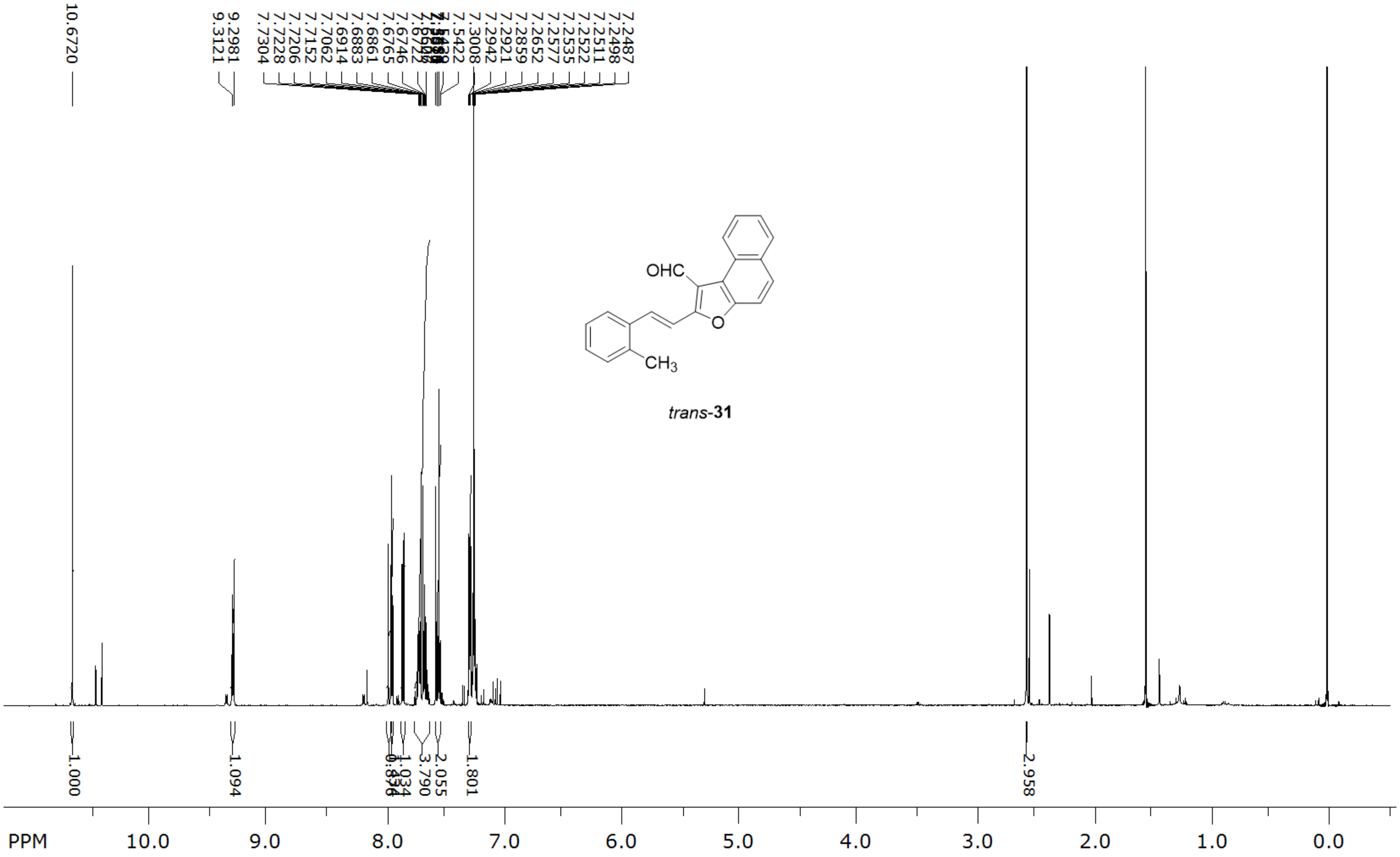

Figure S106. ^{1}H NMR ($CDCl_3$) spectrum of *trans*-**31**.

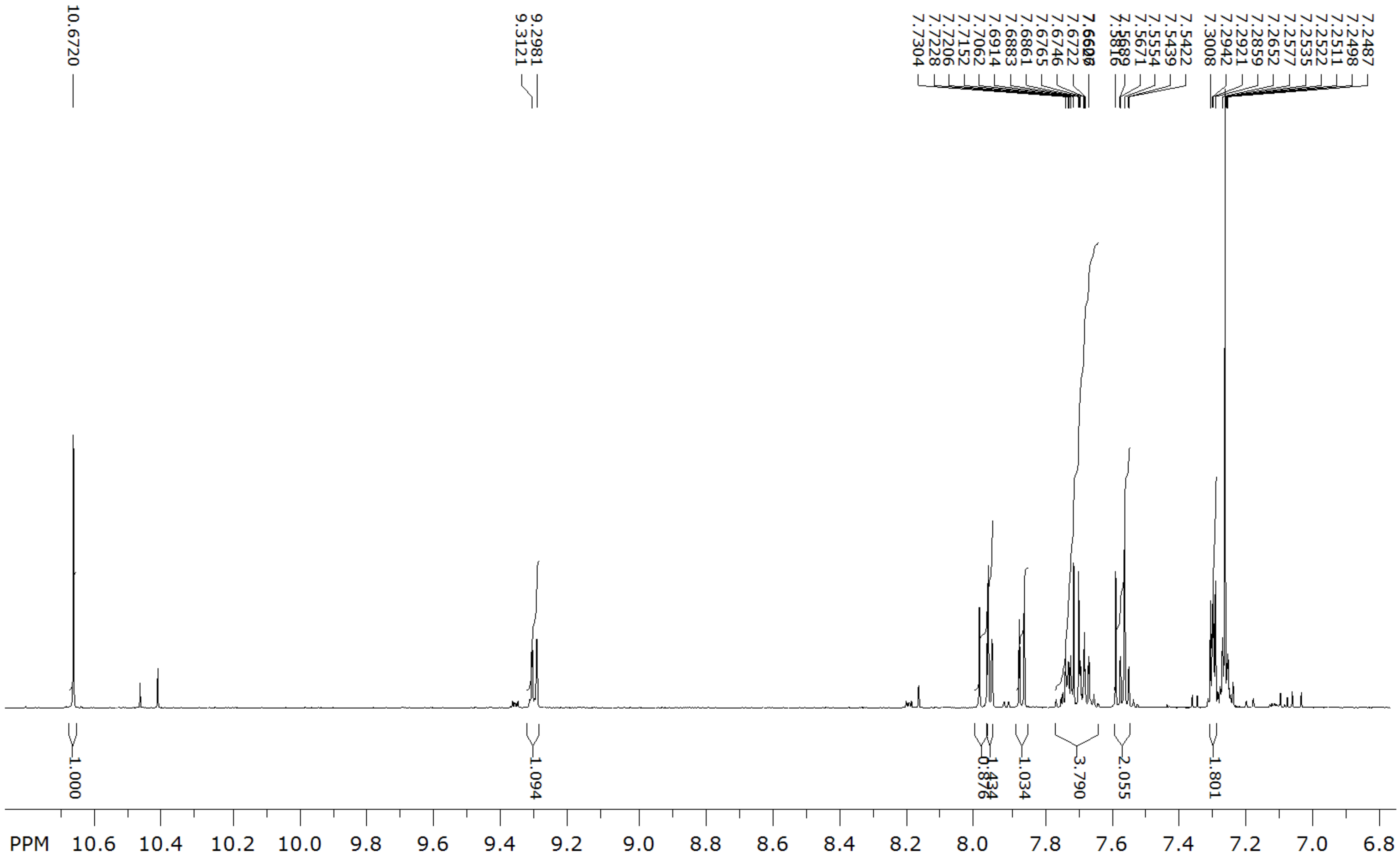

Figure S107. ^{1}H NMR ($CDCl_3$) spectrum of aromatic part of *trans*-**31**.

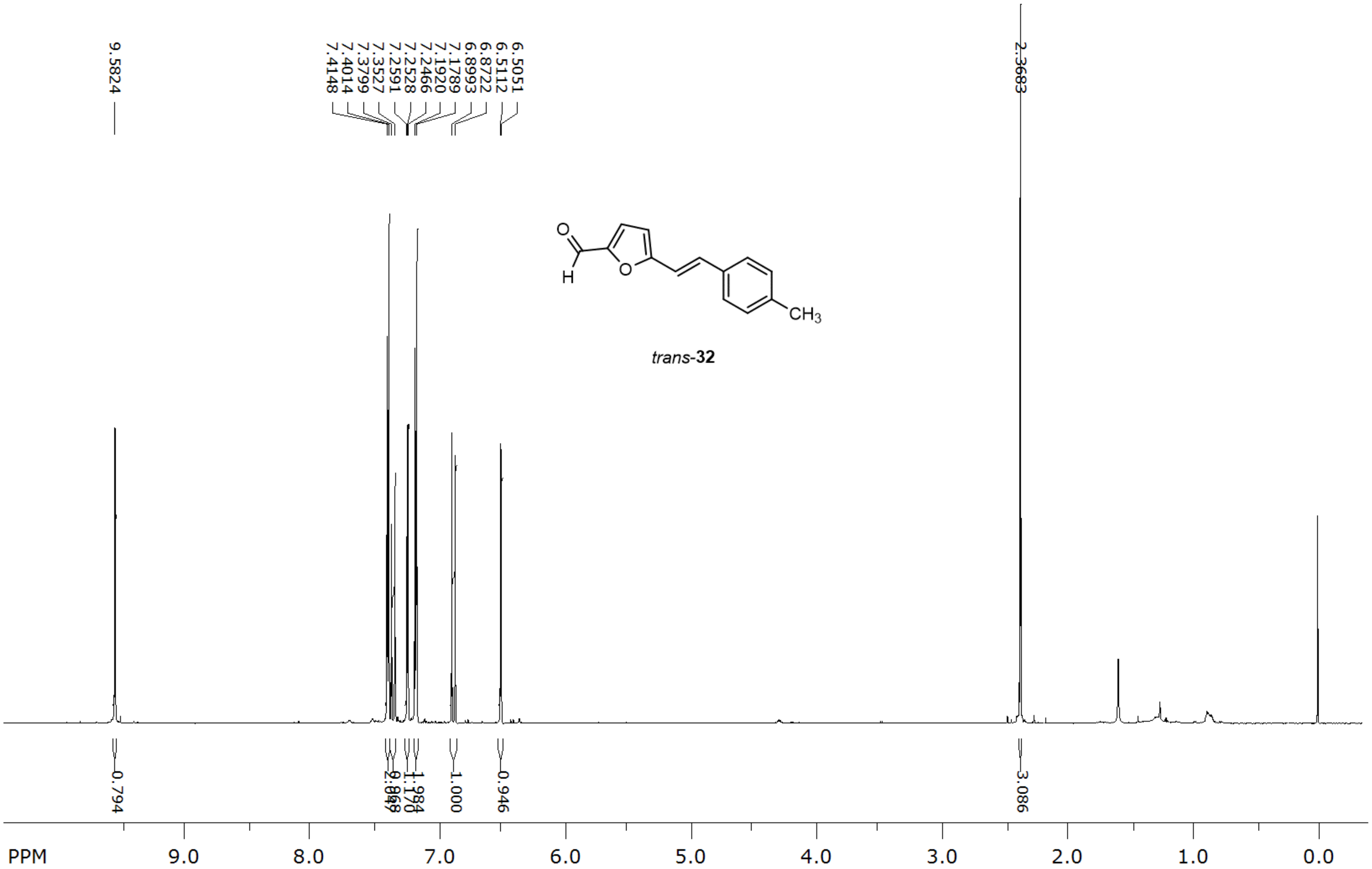

Figure S108. ^{1}H NMR ($CDCl_3$) spectrum of *trans*-**32**.

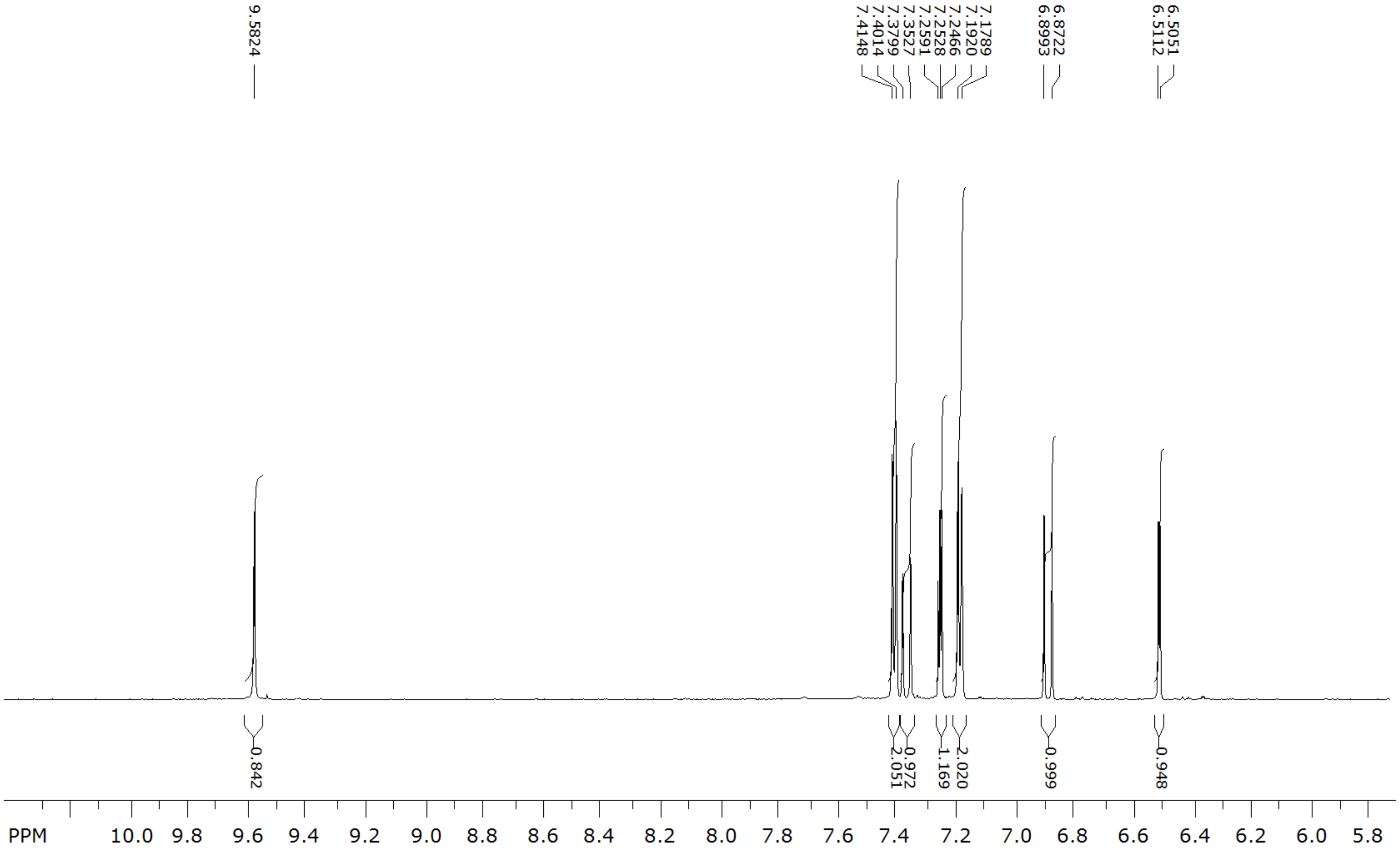

Figure S109. ^{1}H NMR ($CDCl_3$) spectrum of aromatic part of *trans*-**32**.

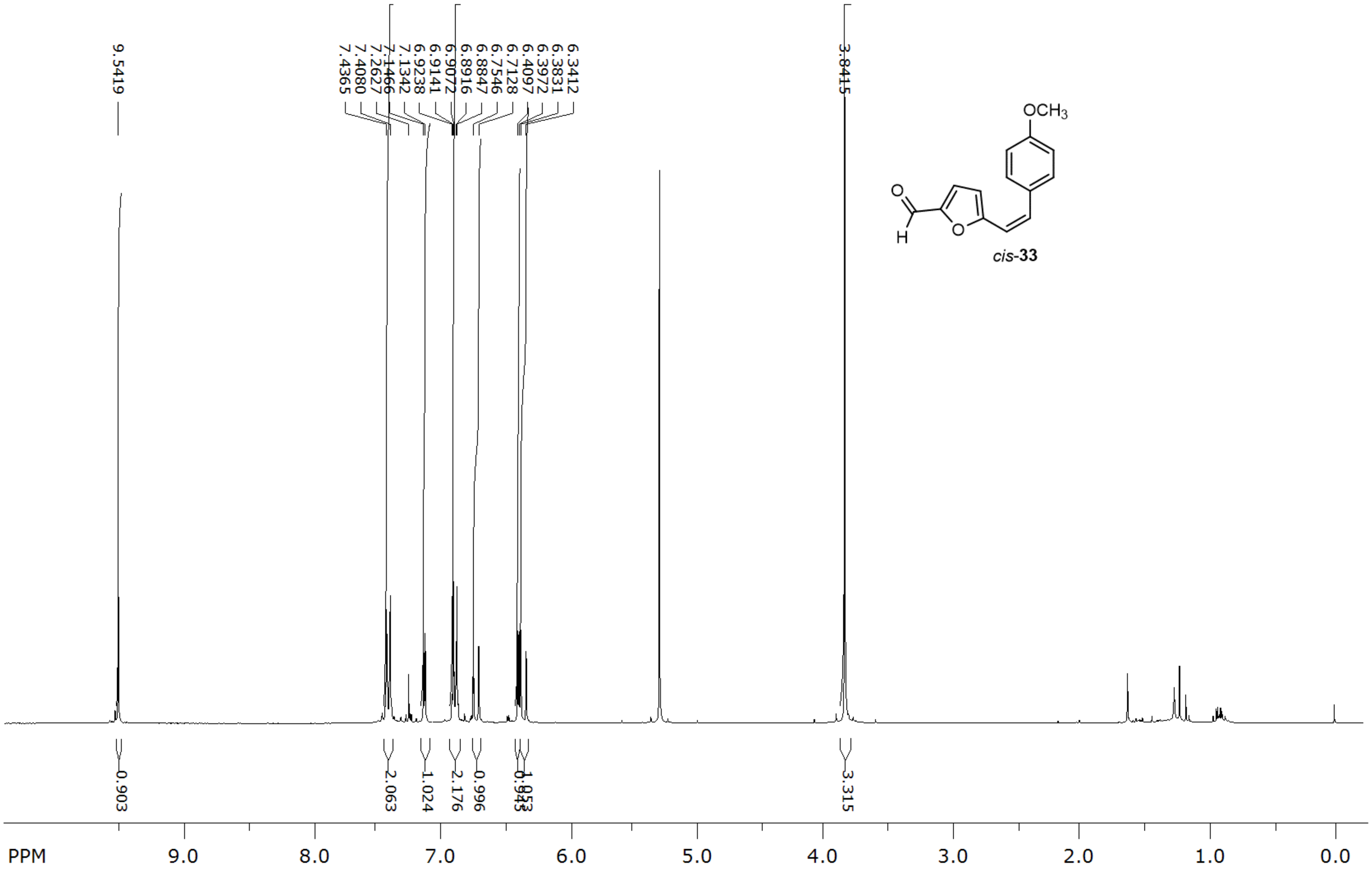

Figure S110. ^{1}H NMR ($CDCl_3$) spectrum of *cis*-**33**.

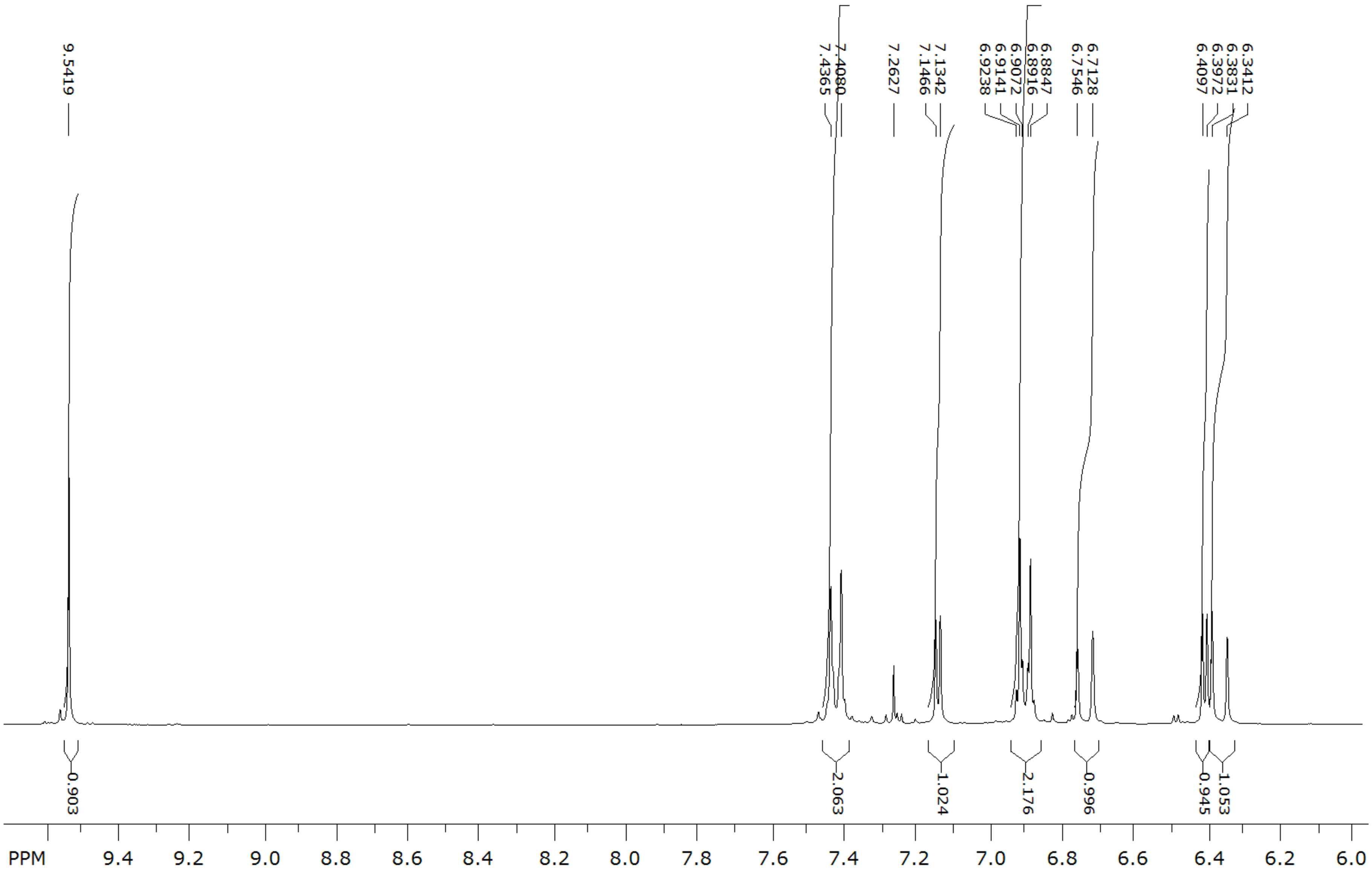

Figure S111. ^{1}H NMR ($CDCl_3$) spectrum of aromatic part of *cis*-**33**.

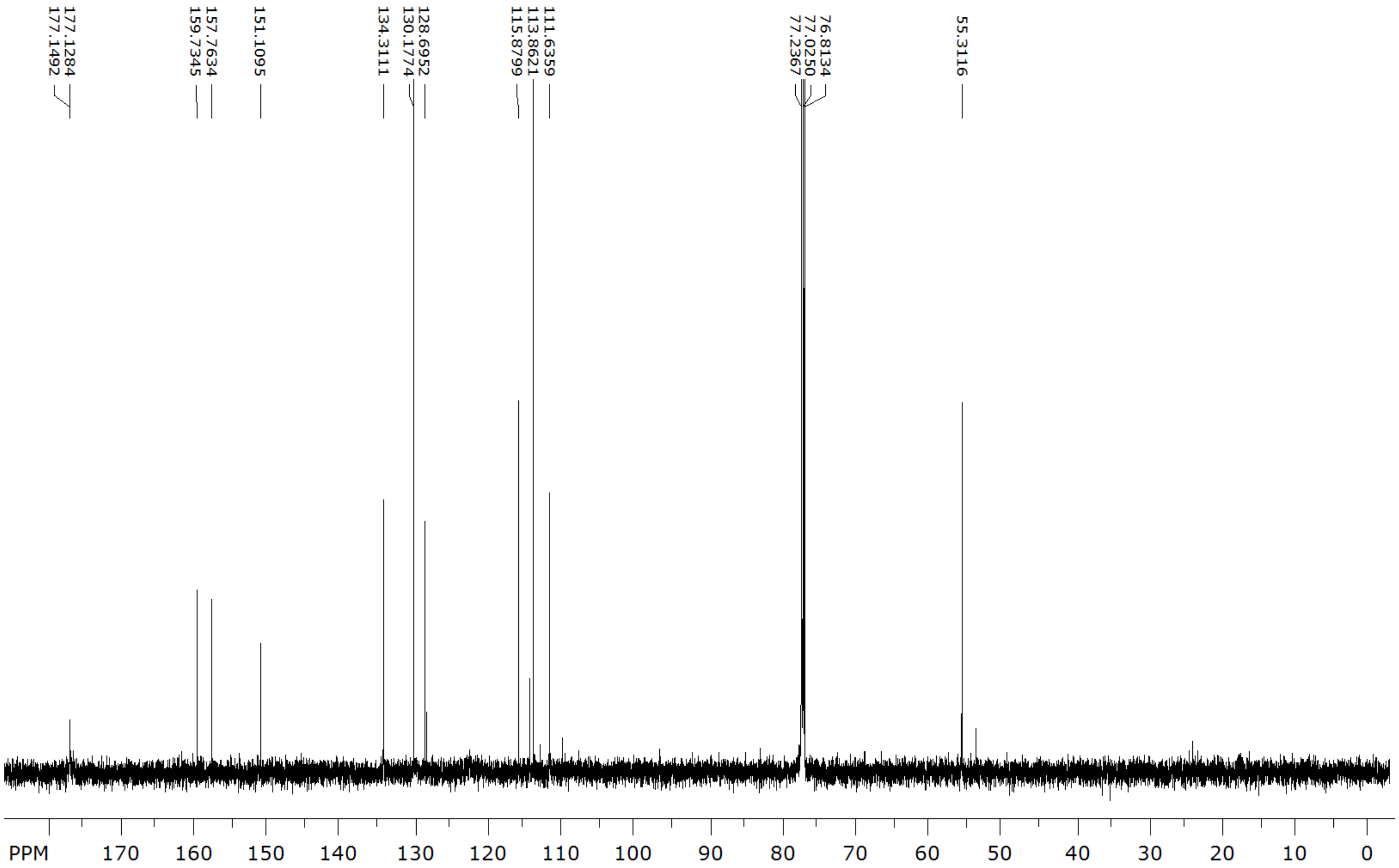

Figure S112. ^{1}H NMR ($CDCl_3$) spectrum of *cis*-**33**.

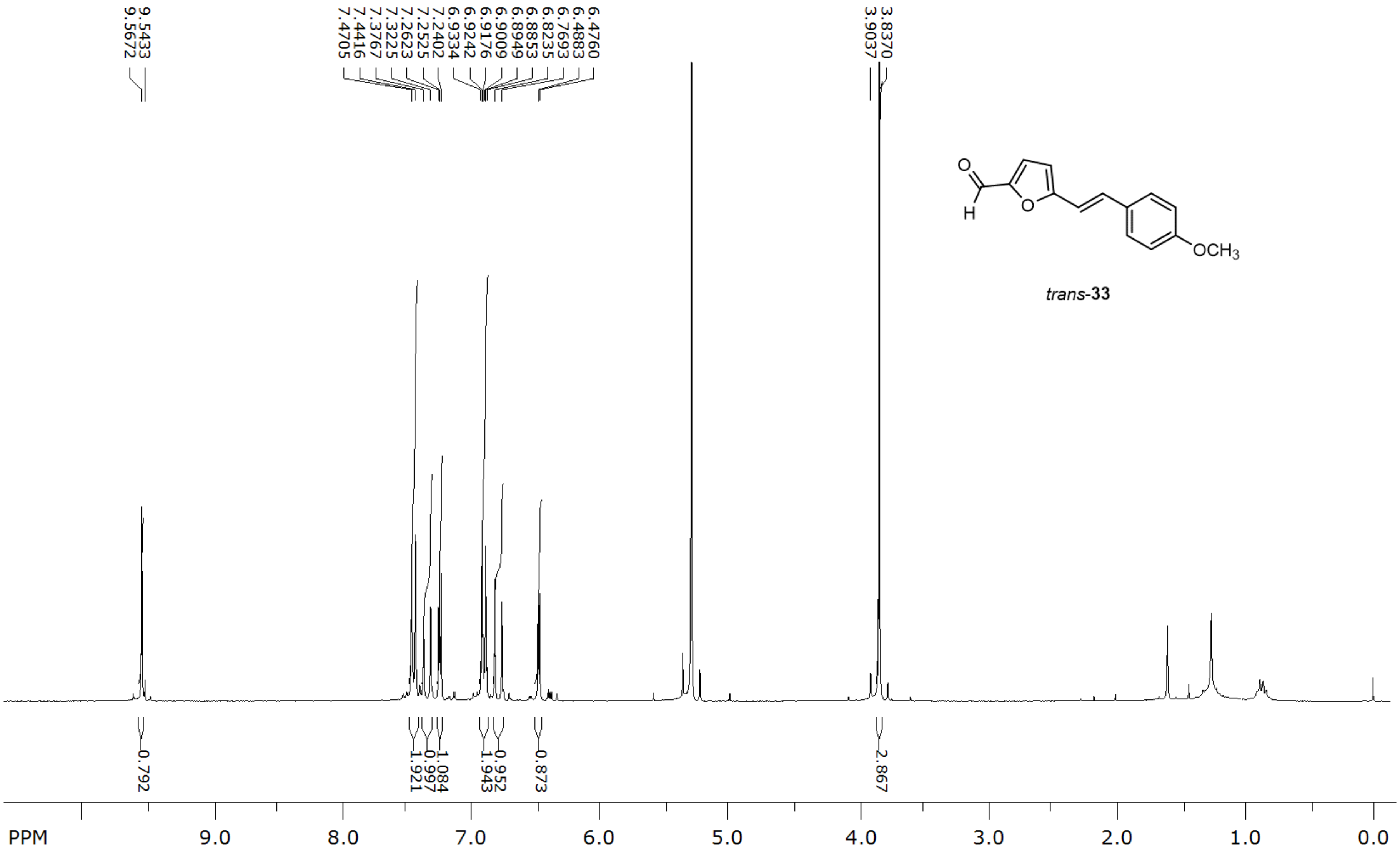

Figure S113. ^{1}H NMR ($CDCl_3$) spectrum of *trans*-**33**.

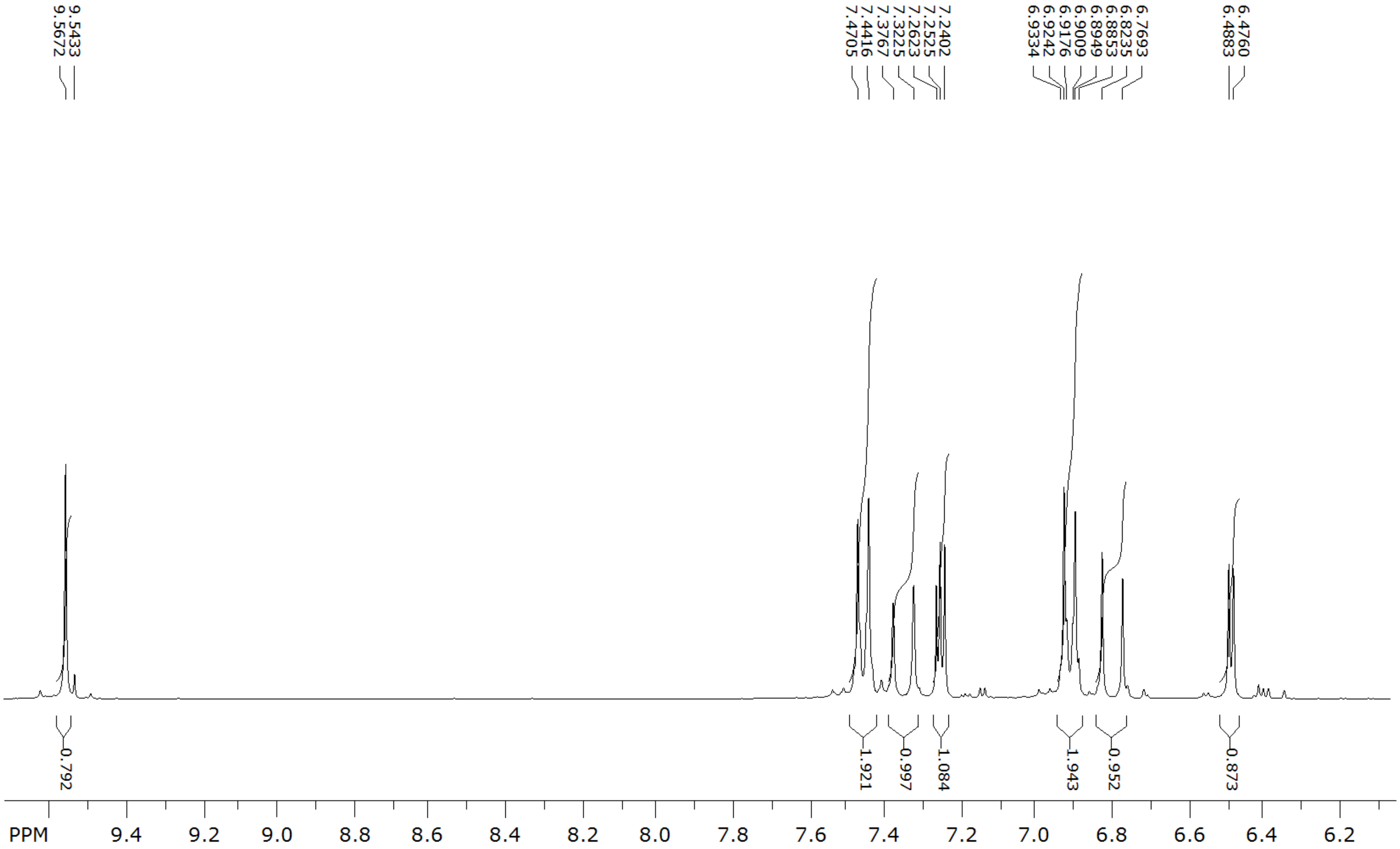

Figure S114. ^{1}H NMR ($CDCl_3$) spectrum of aromatic part of *trans*-**33**.

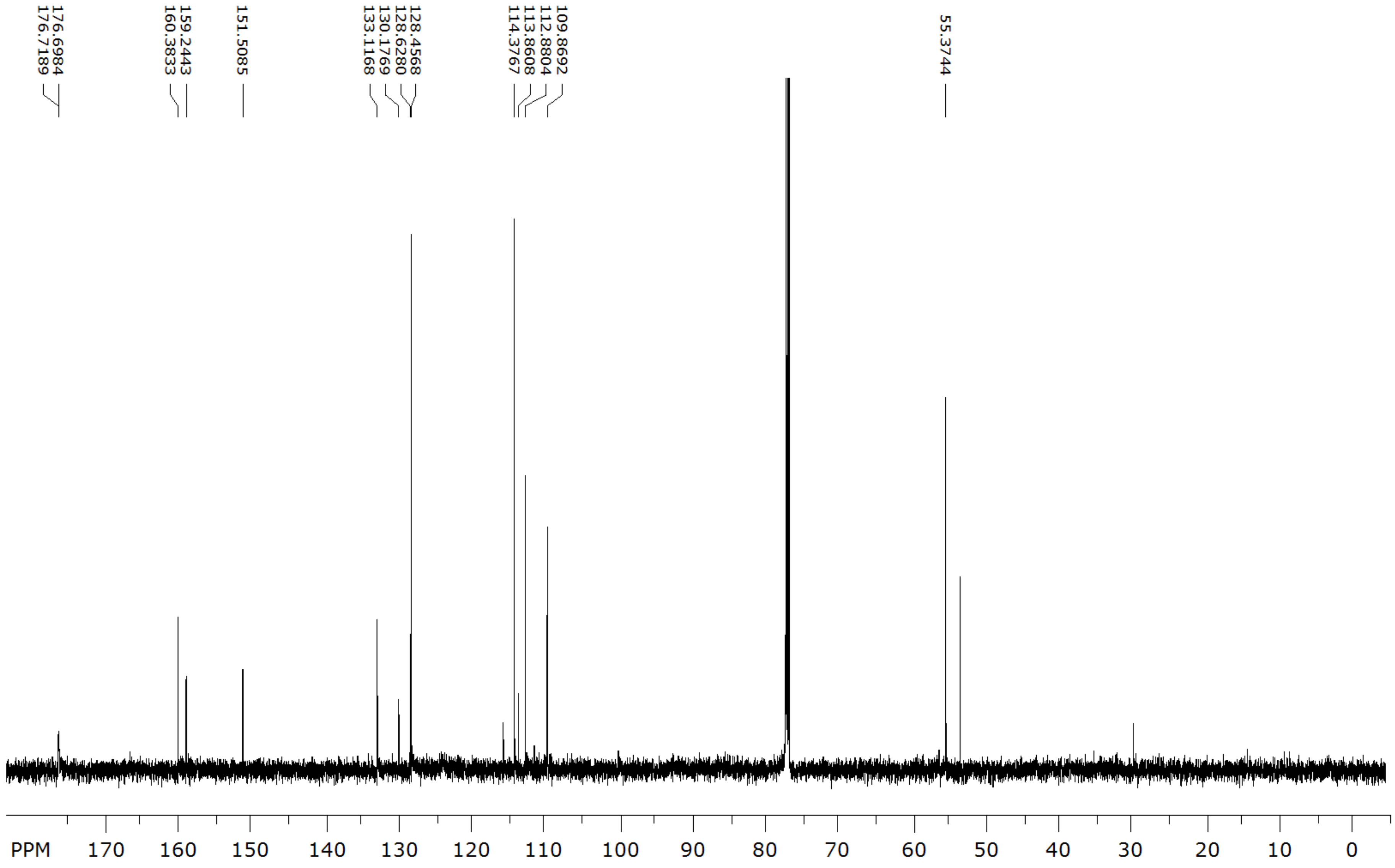

Figure S115. ^{13}C NMR ($CDCl_3$) spectrum of *trans*-**33**.

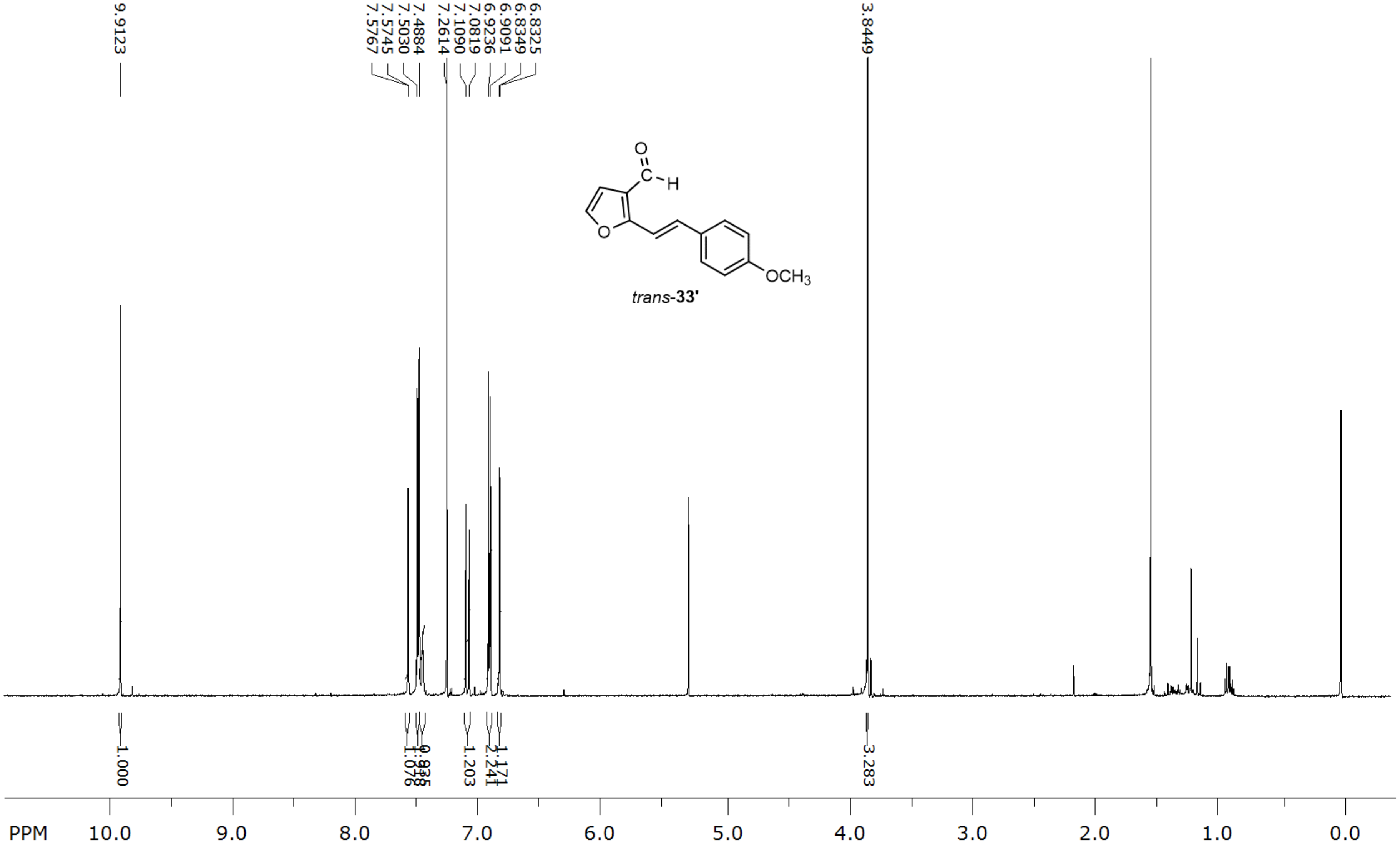

Figure S116. ^{1}H NMR ($CDCl_3$) spectrum of *trans*-**33'**.

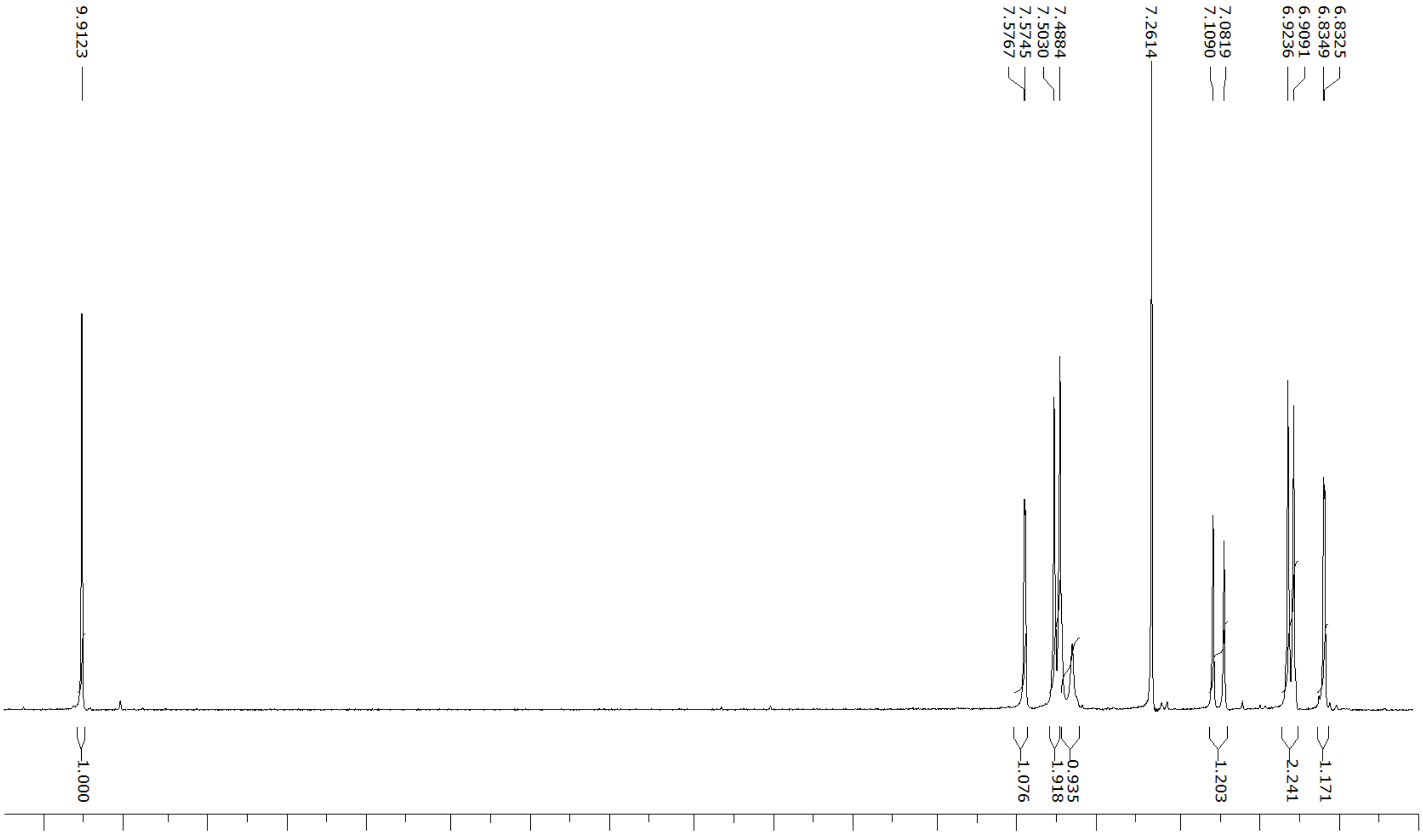

Figure S117. ^{1}H NMR ($CDCl_3$) spectrum of aromatic part of *trans*-**33'**.

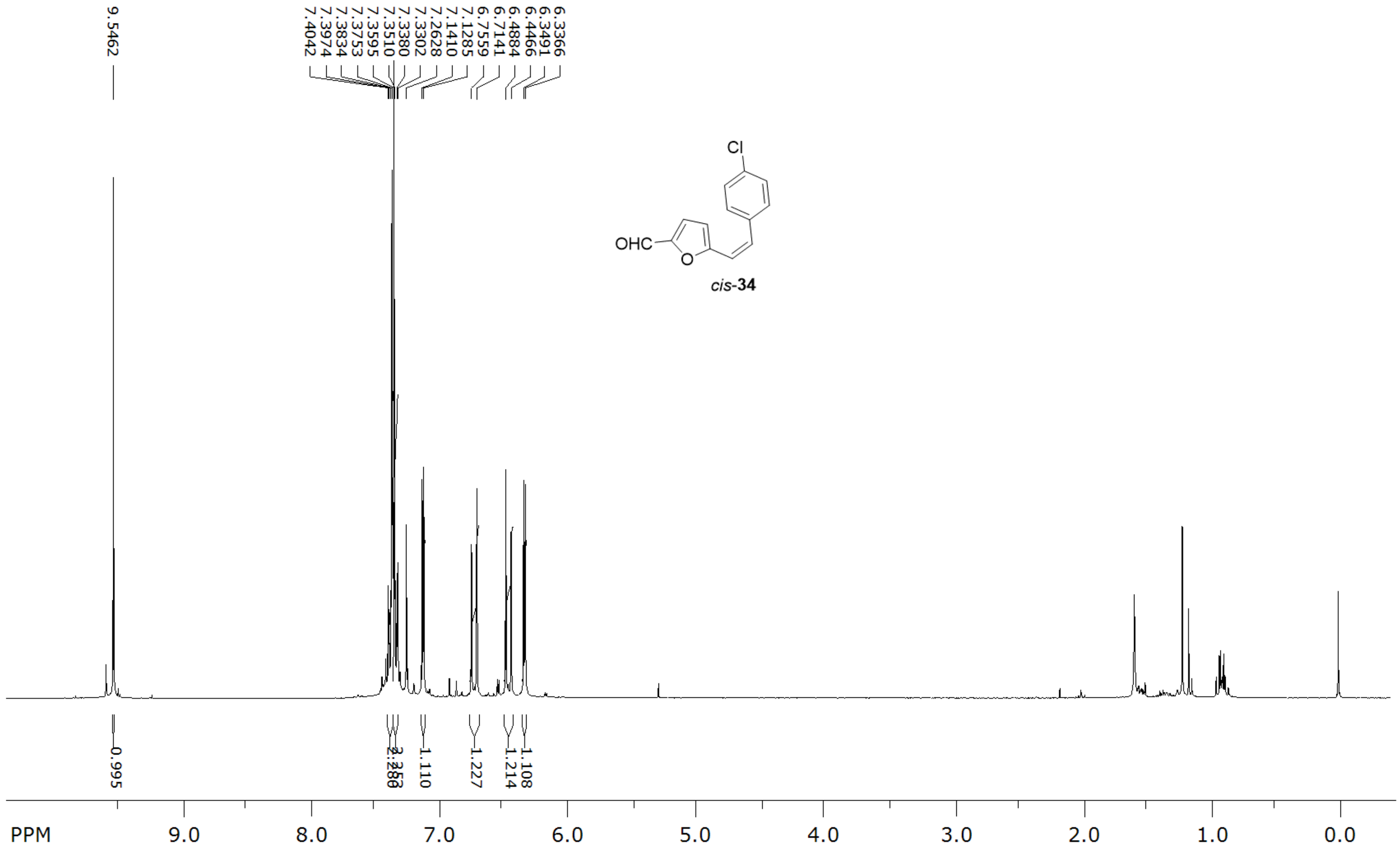

Figure S118. ^{1}H NMR ($CDCl_3$) spectrum of *cis*-**34**.

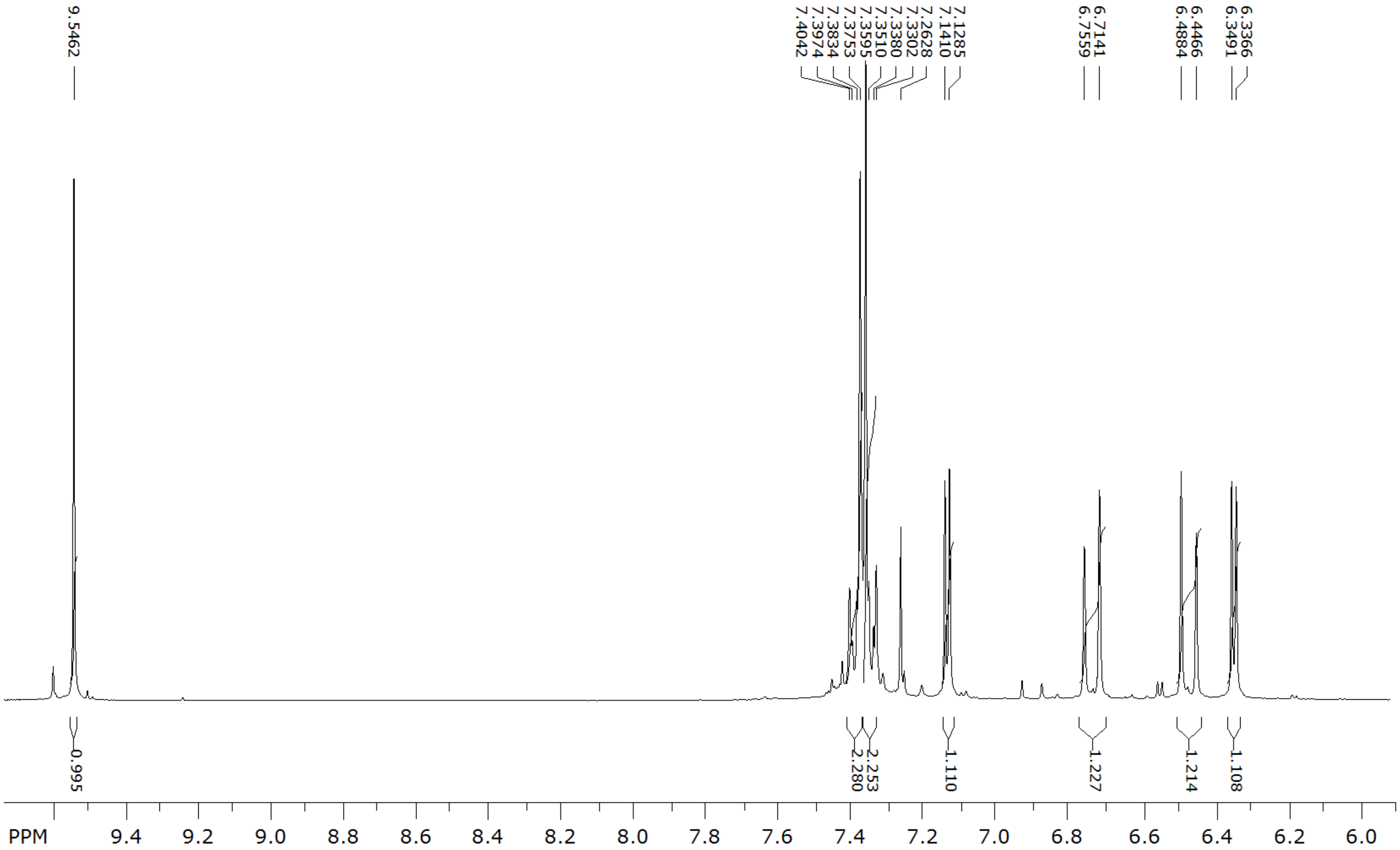

Figure S119. ^{1}H NMR ($CDCl_3$) spectrum of aromatic part of *cis*-**34**.

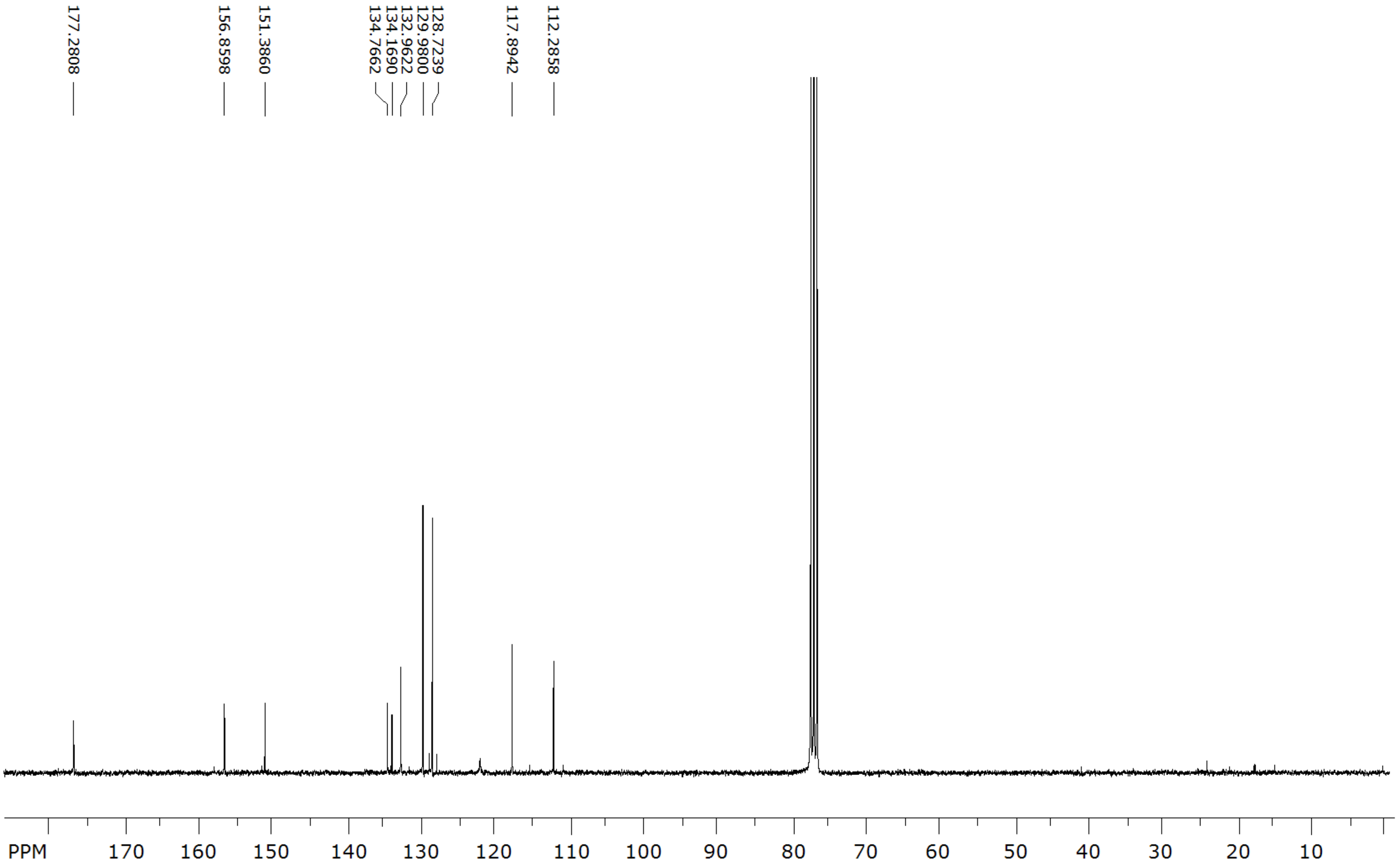

Figure S120. ^{13}C NMR ($CDCl_3$) spectrum of *cis*-**34**.

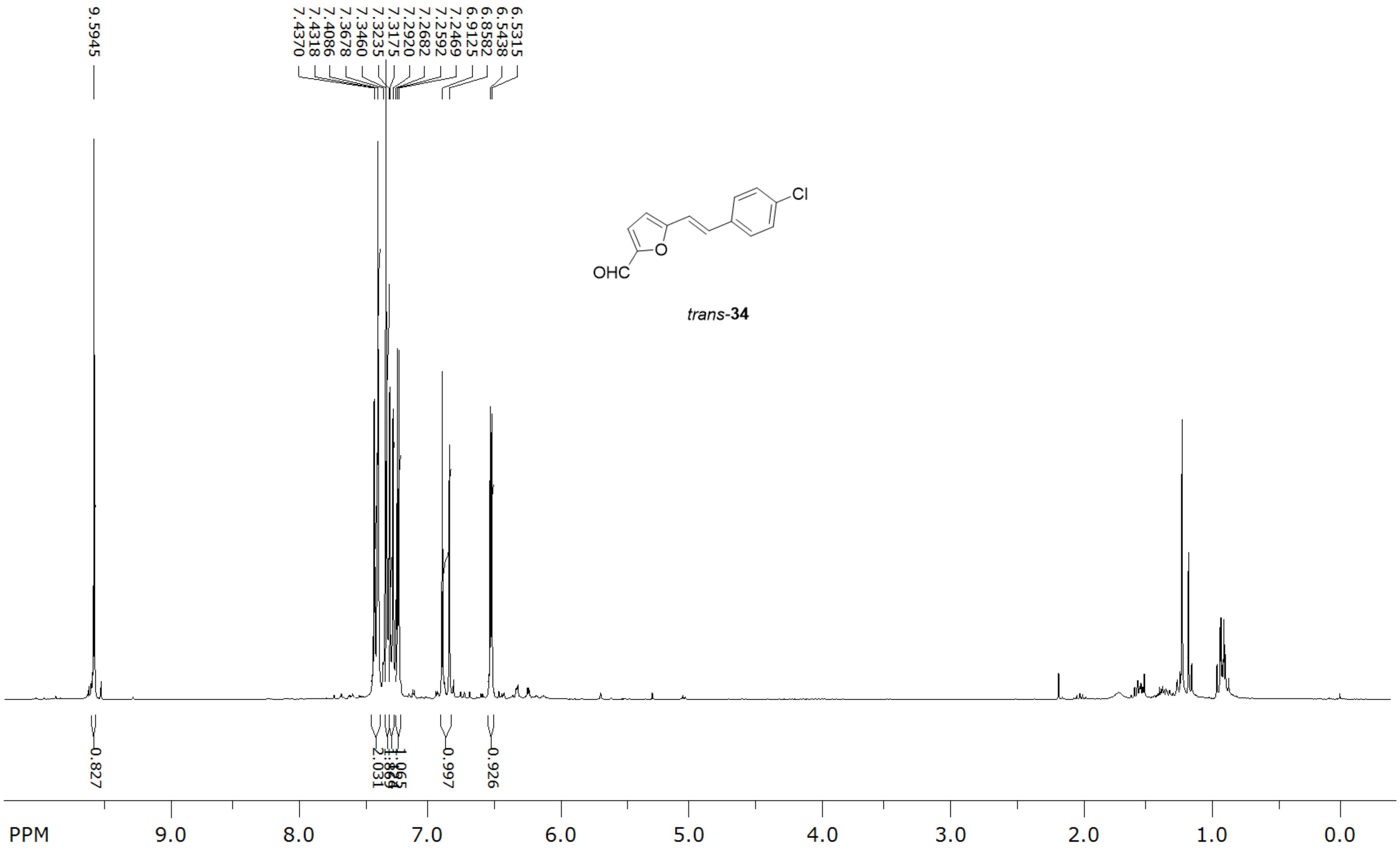

Figure S121. ^{1}H NMR ($CDCl_3$) spectrum of *trans*-**34**.

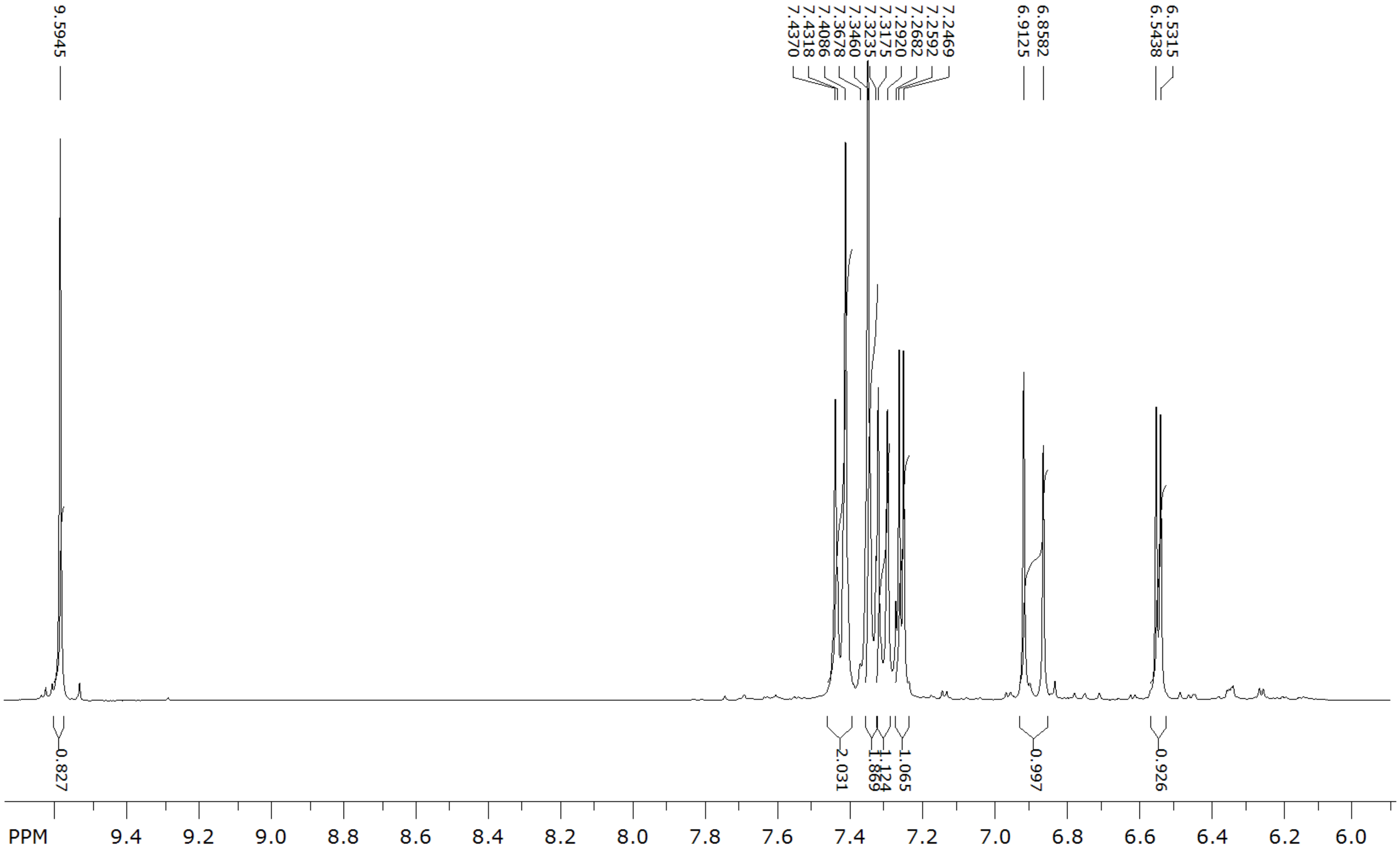

Figure S122. ^{1}H NMR ($CDCl_3$) spectrum of aromatic part of *trans*-**34**.

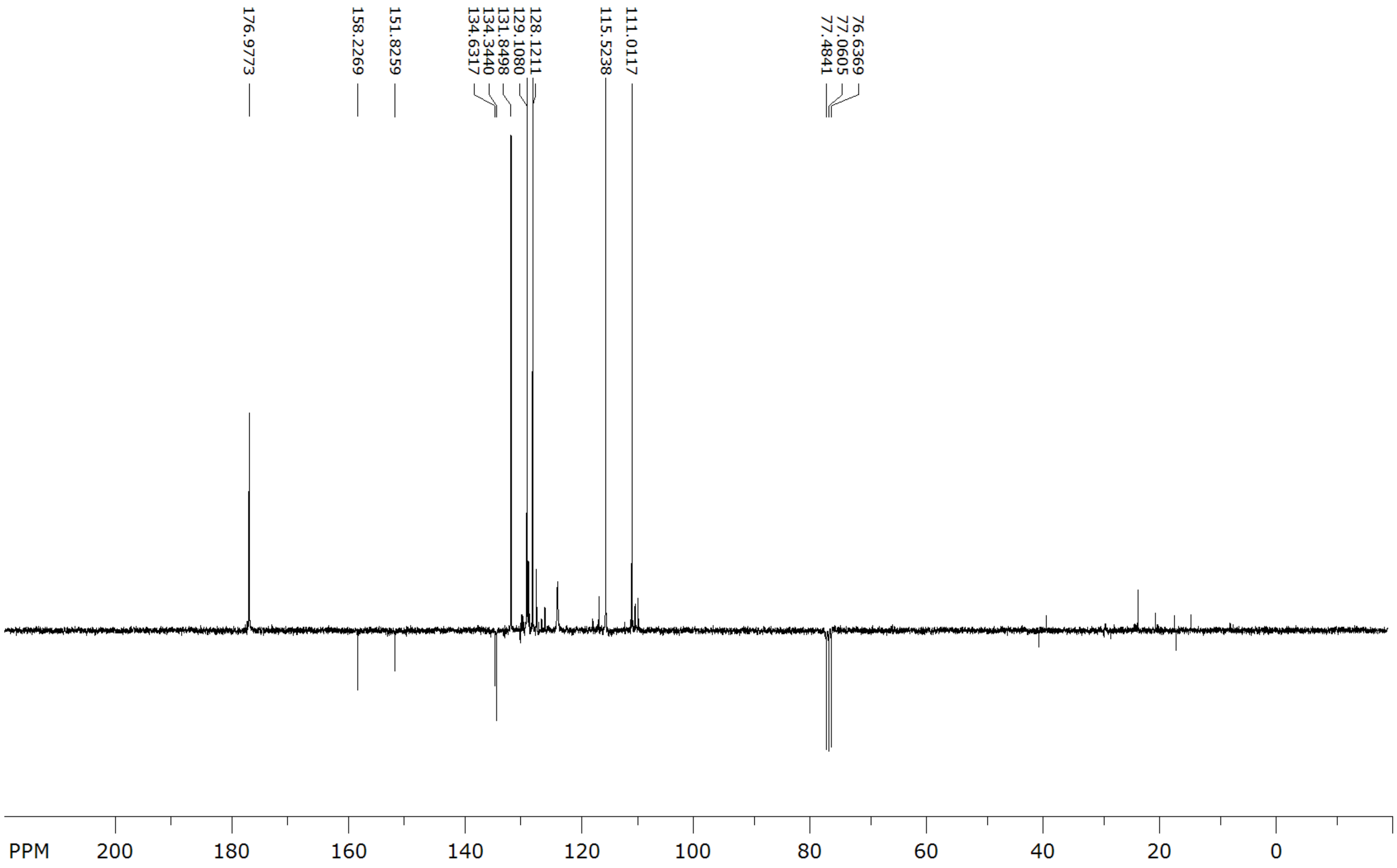

Figure S123. ^{13}C NMR ($CDCl_3$) spectrum of *trans*-**34**.

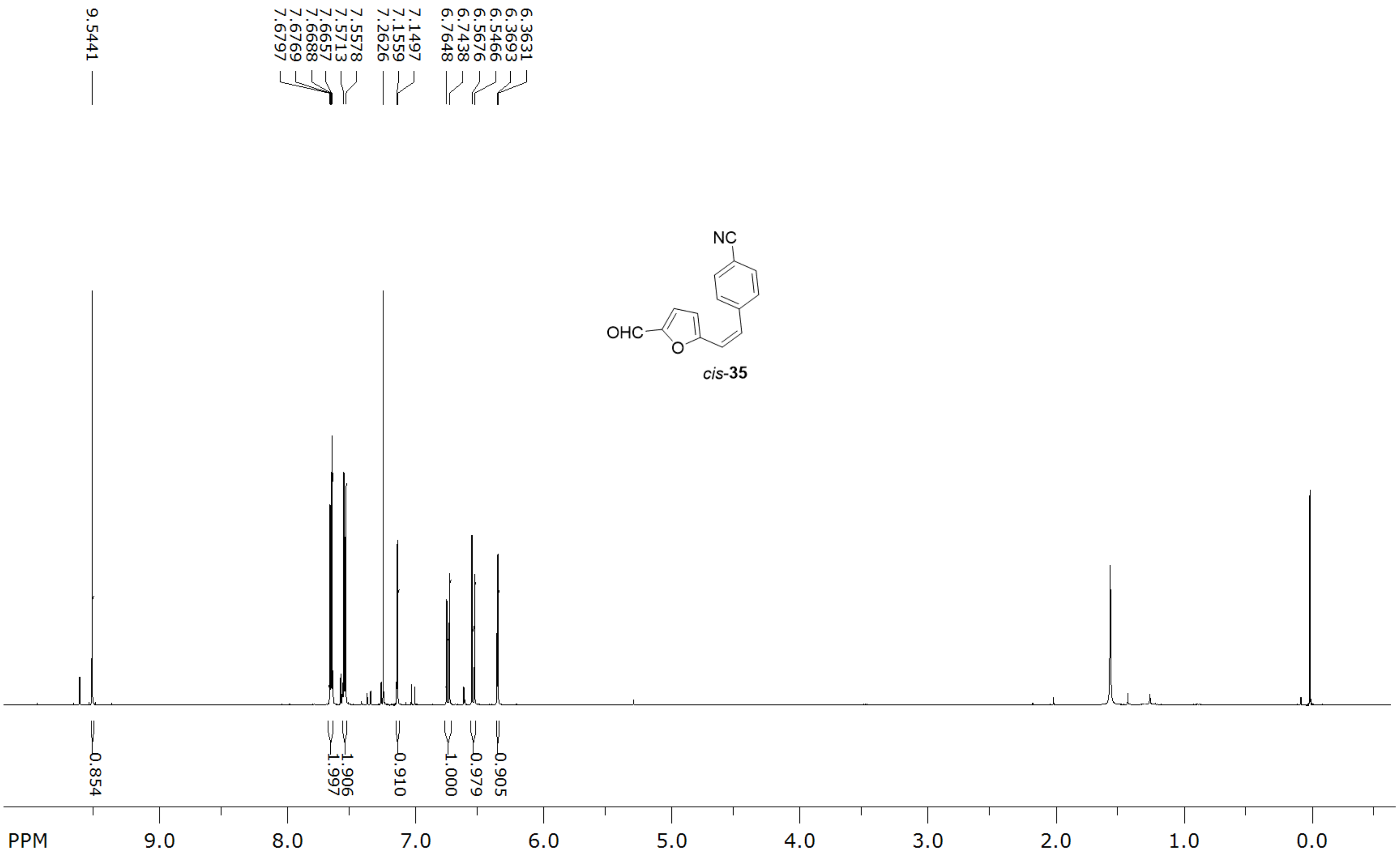

Figure S124. ^{1}H NMR ($CDCl_3$) spectrum of *cis*-**35**.

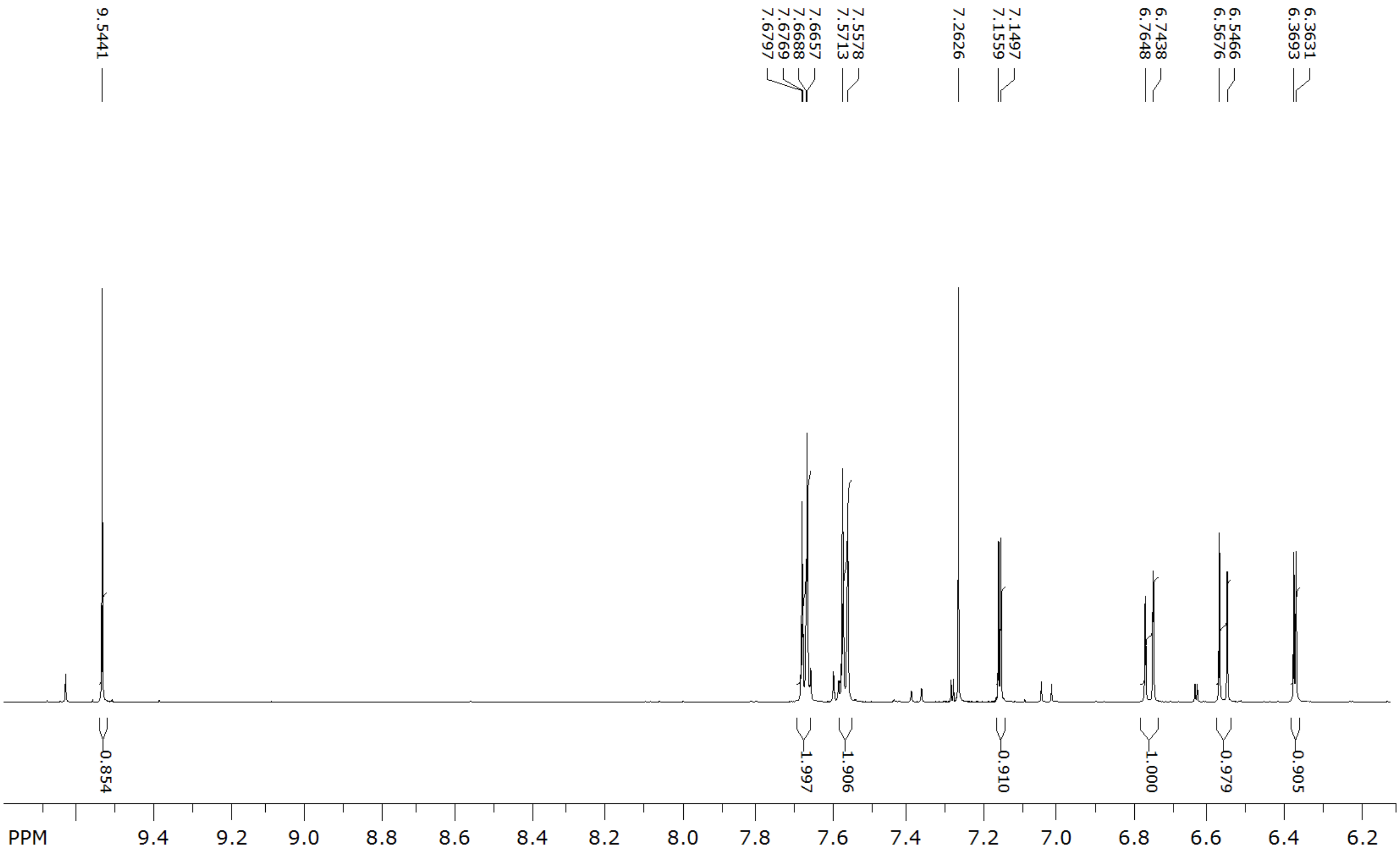

Figure S125. ^{1}H NMR ($CDCl_3$) spectrum of aromatic part of *cis*-**35**.

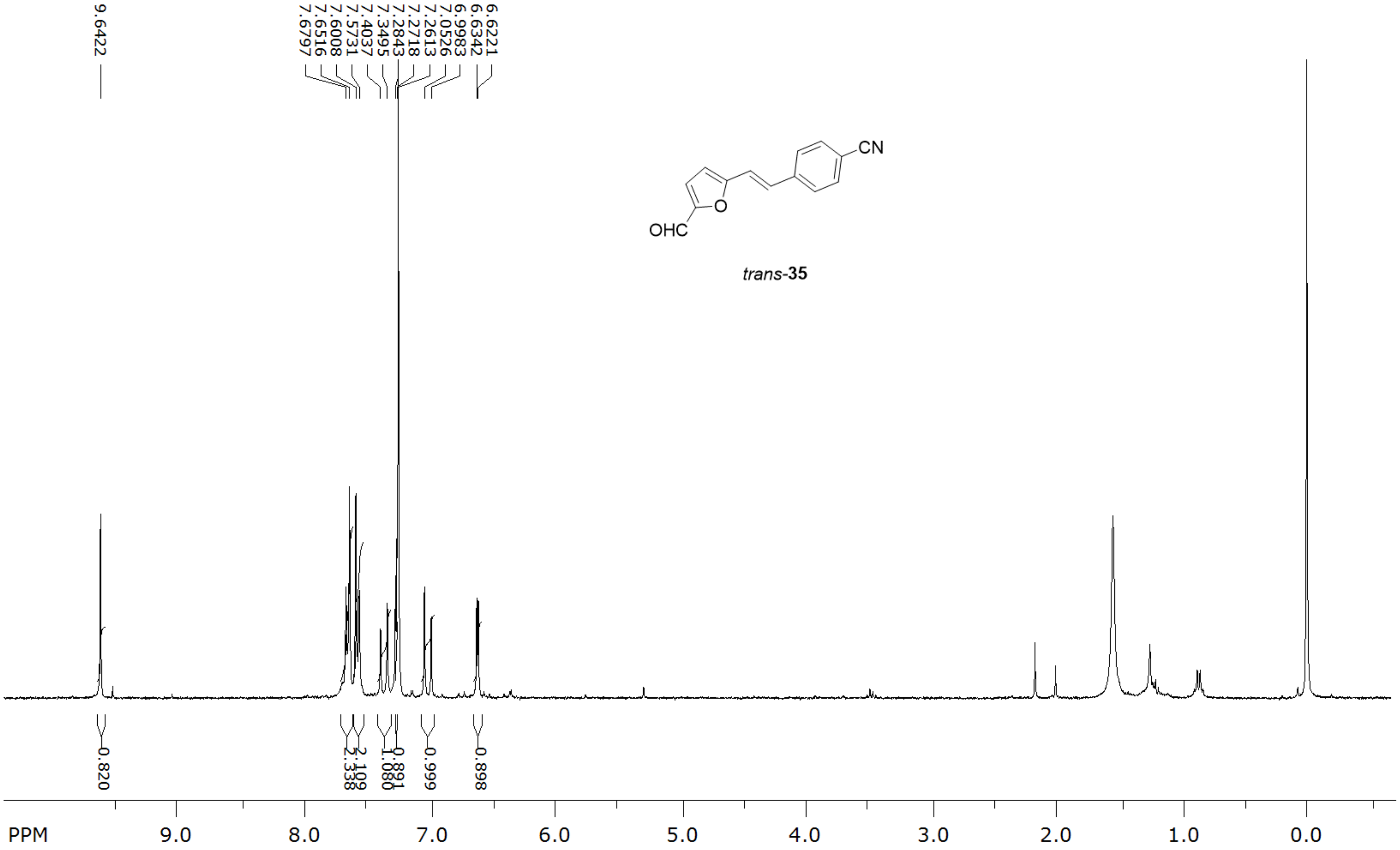

Figure S126. ^{1}H NMR ($CDCl_3$) spectrum of *trans*-**35**.

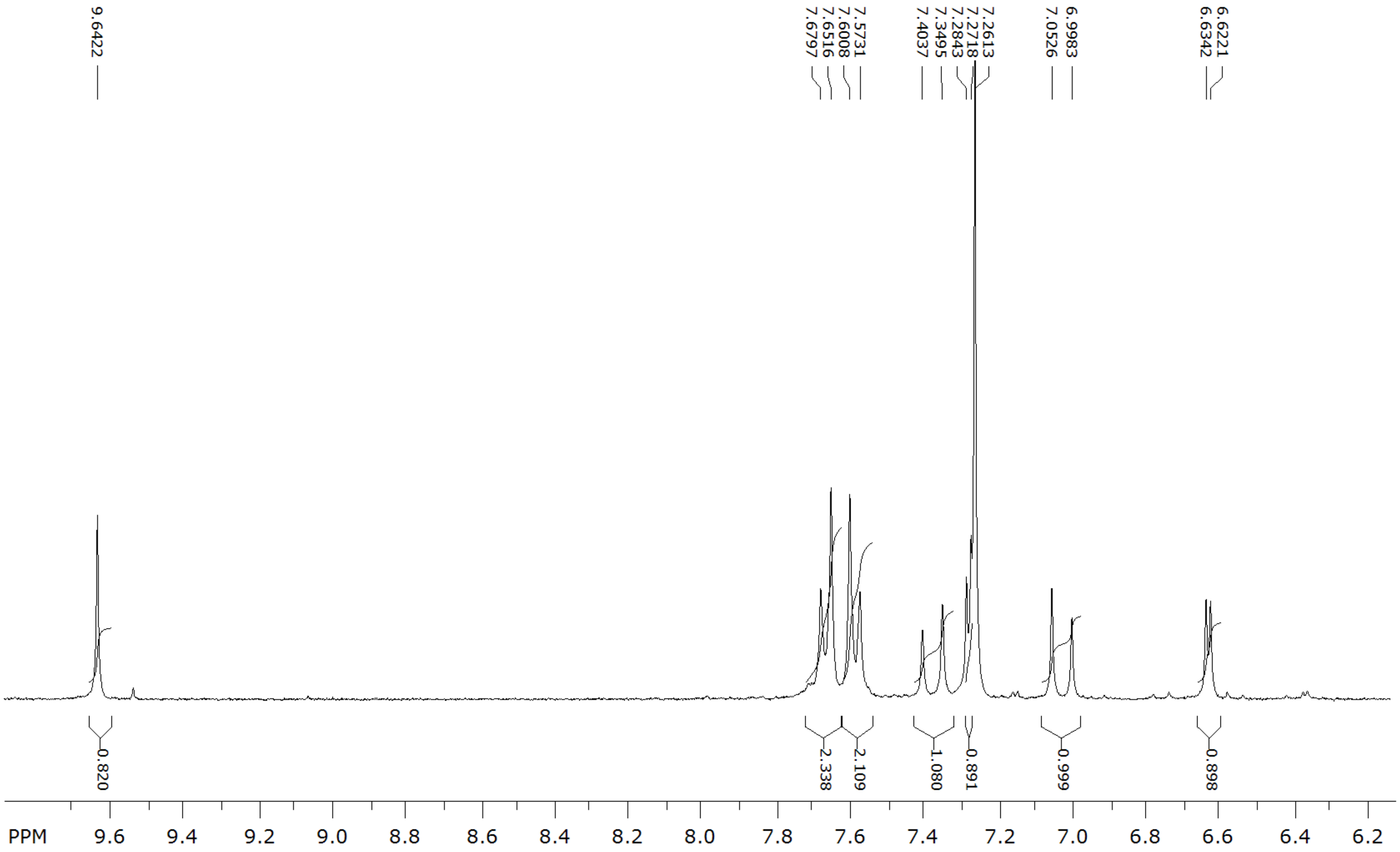

Figure S127. ^{1}H NMR ($CDCl_3$) spectrum of aromatic part of *trans*-**35**.

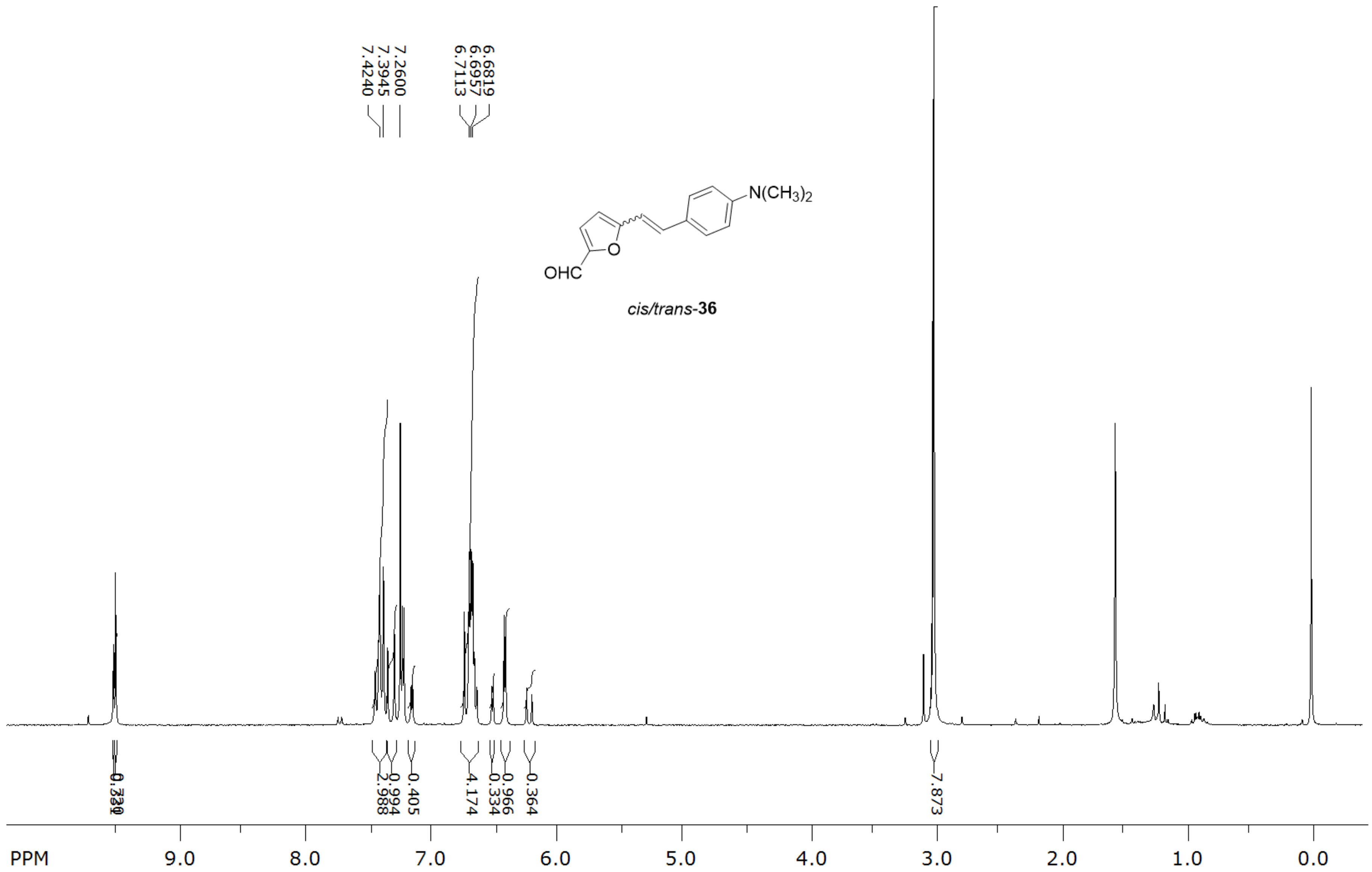

Figure S128. ^{1}H NMR ($CDCl_3$) spectrum of *cis/trans*-**36**.

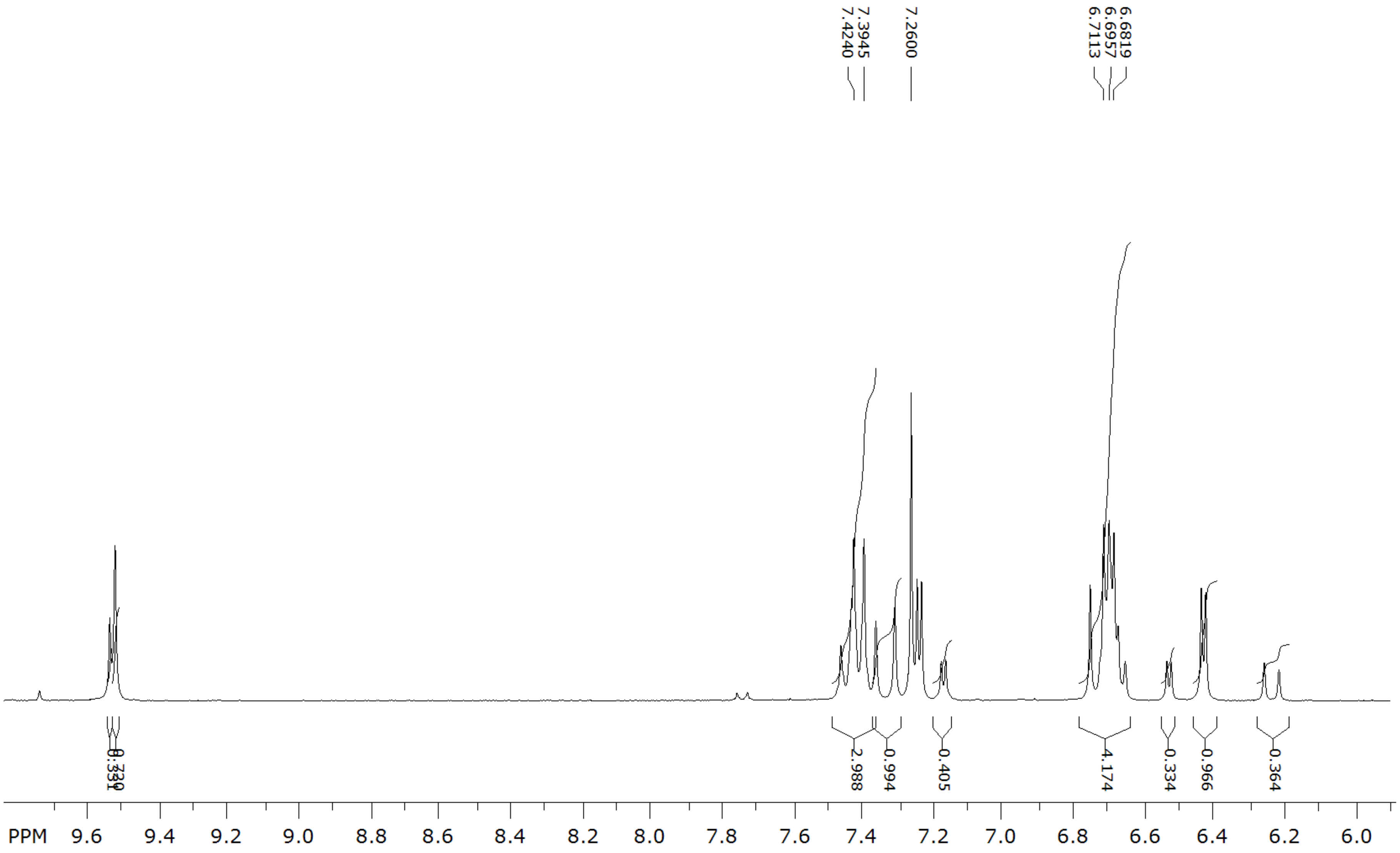

Figure S129. ^{1}H NMR ($CDCl_3$) spectrum of aromatic part of *cis/trans*-**36**.

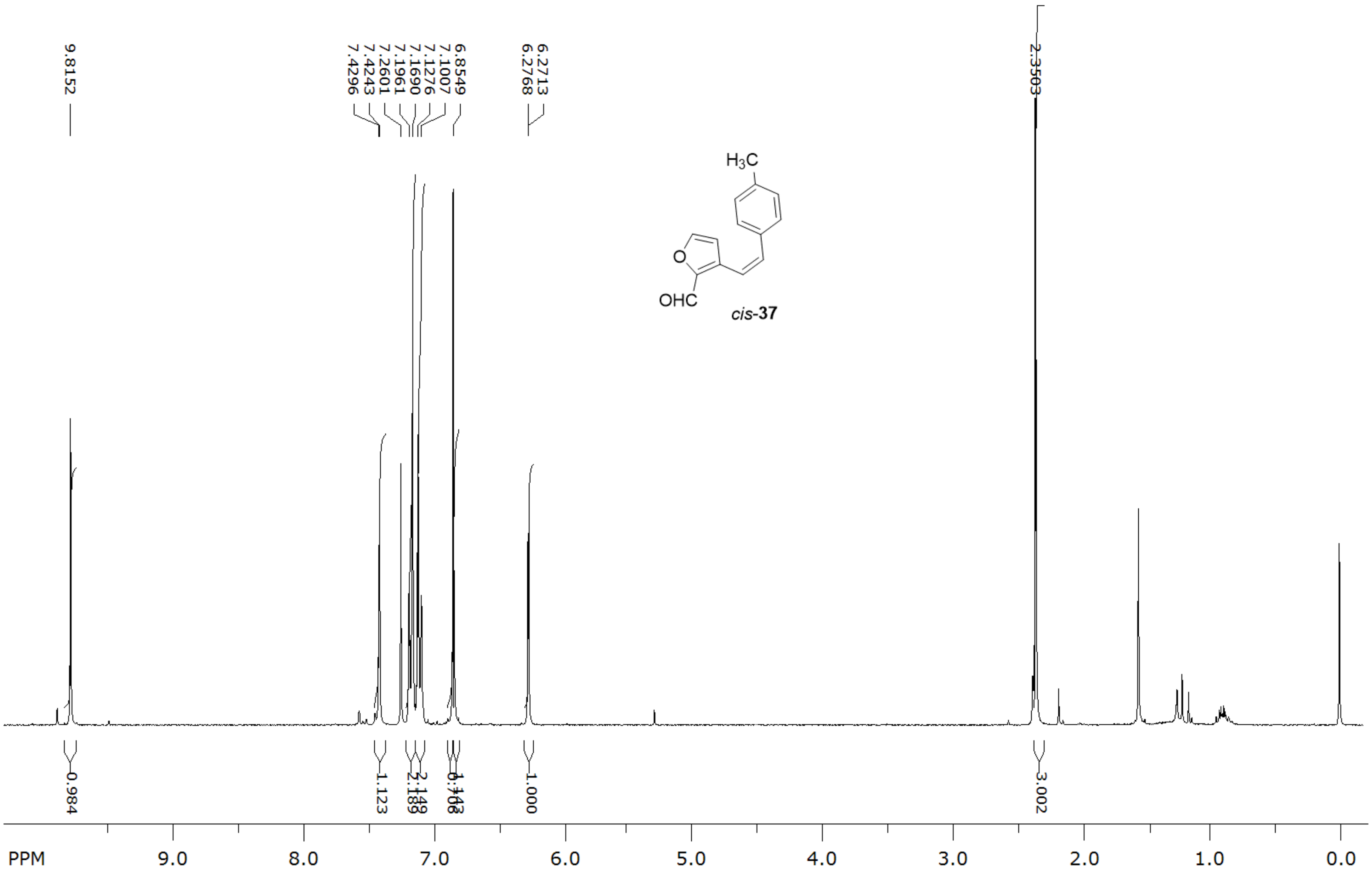

Figure S130. ^{1}H NMR ($CDCl_3$) spectrum of *cis*-**37**.

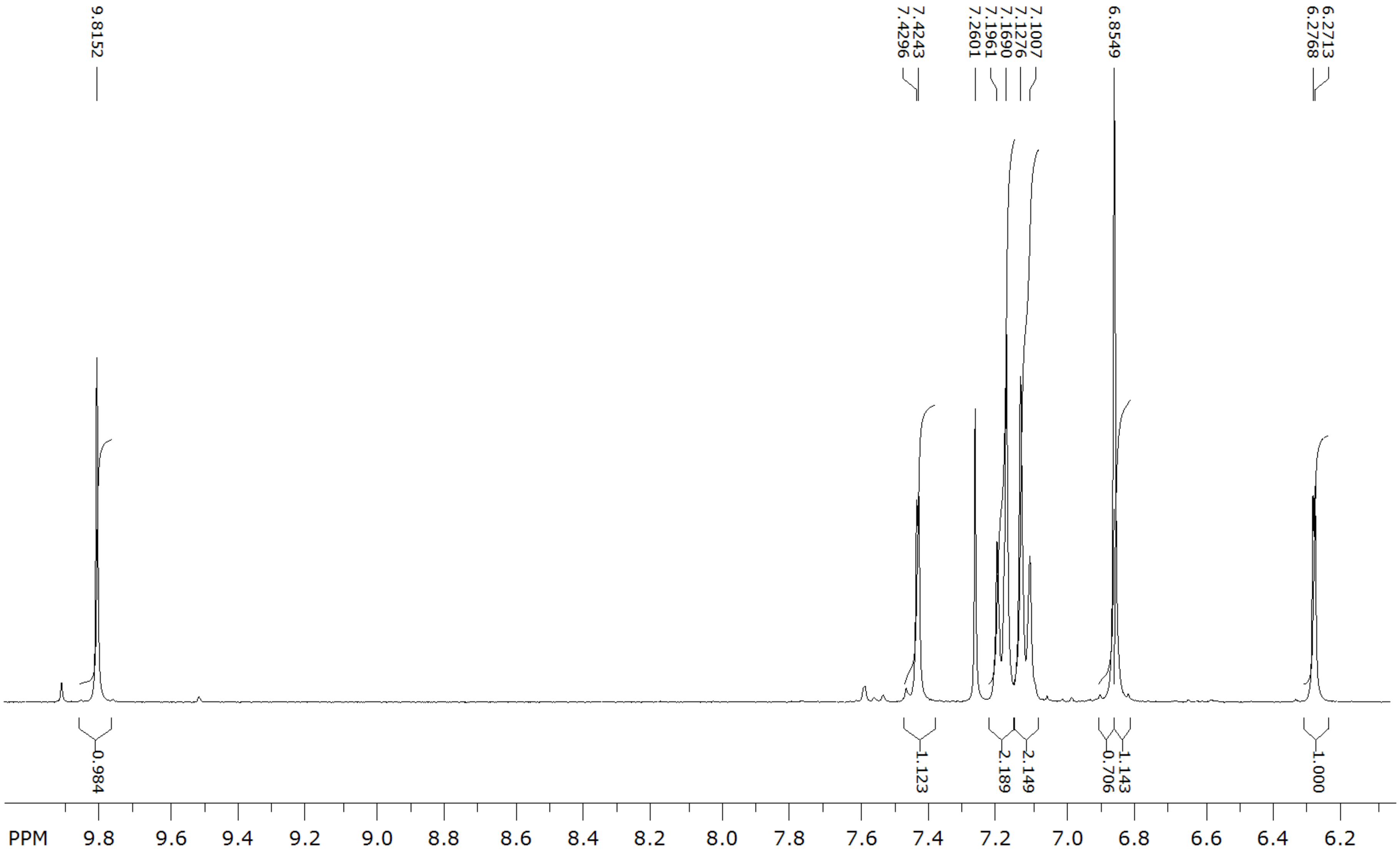

Figure S131. ^{1}H NMR ($CDCl_3$) spectrum of aromatic part of *cis*-**37**.

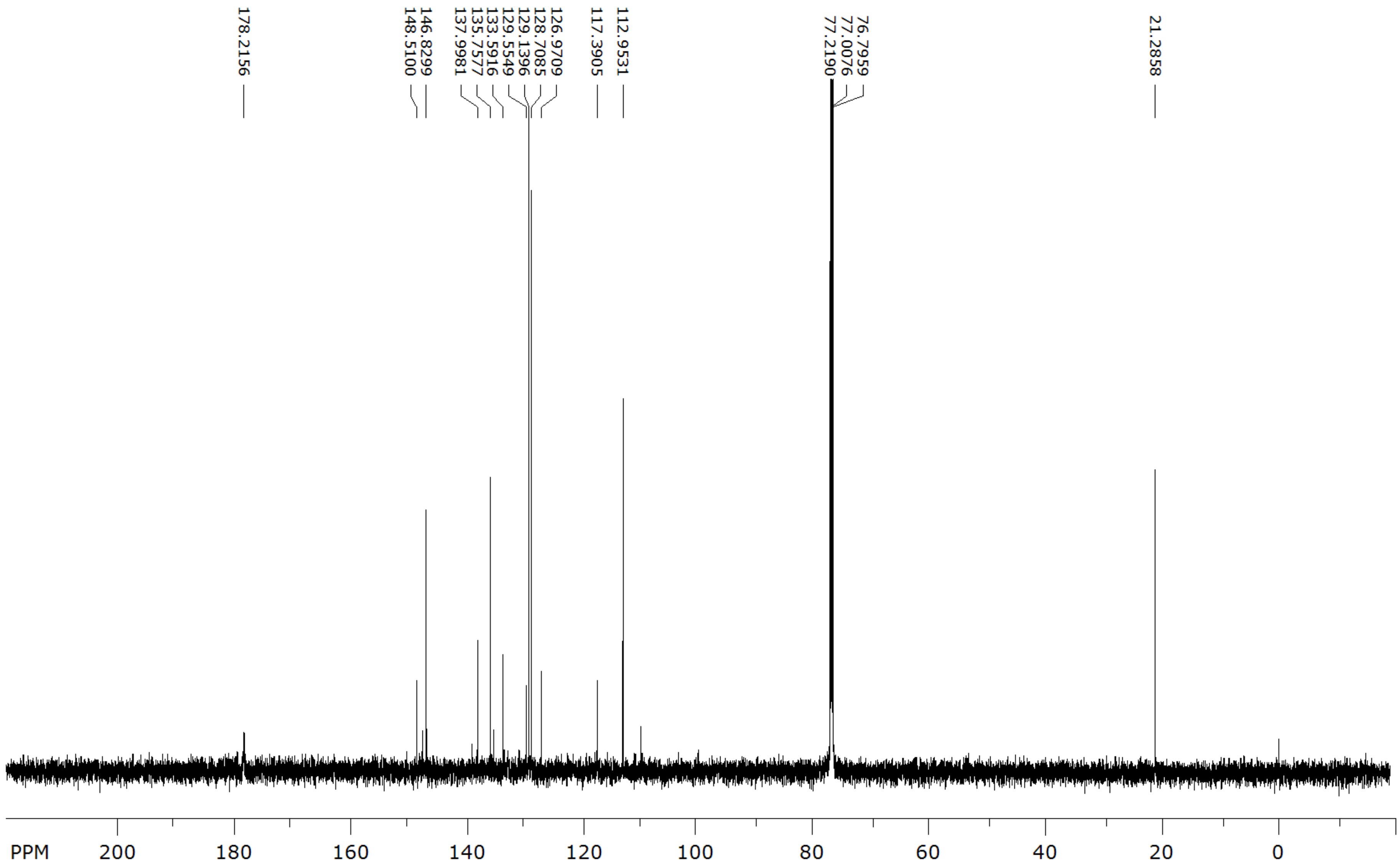

Figure S132. ^{13}C NMR ($CDCl_3$) spectrum of *cis*-**37**.

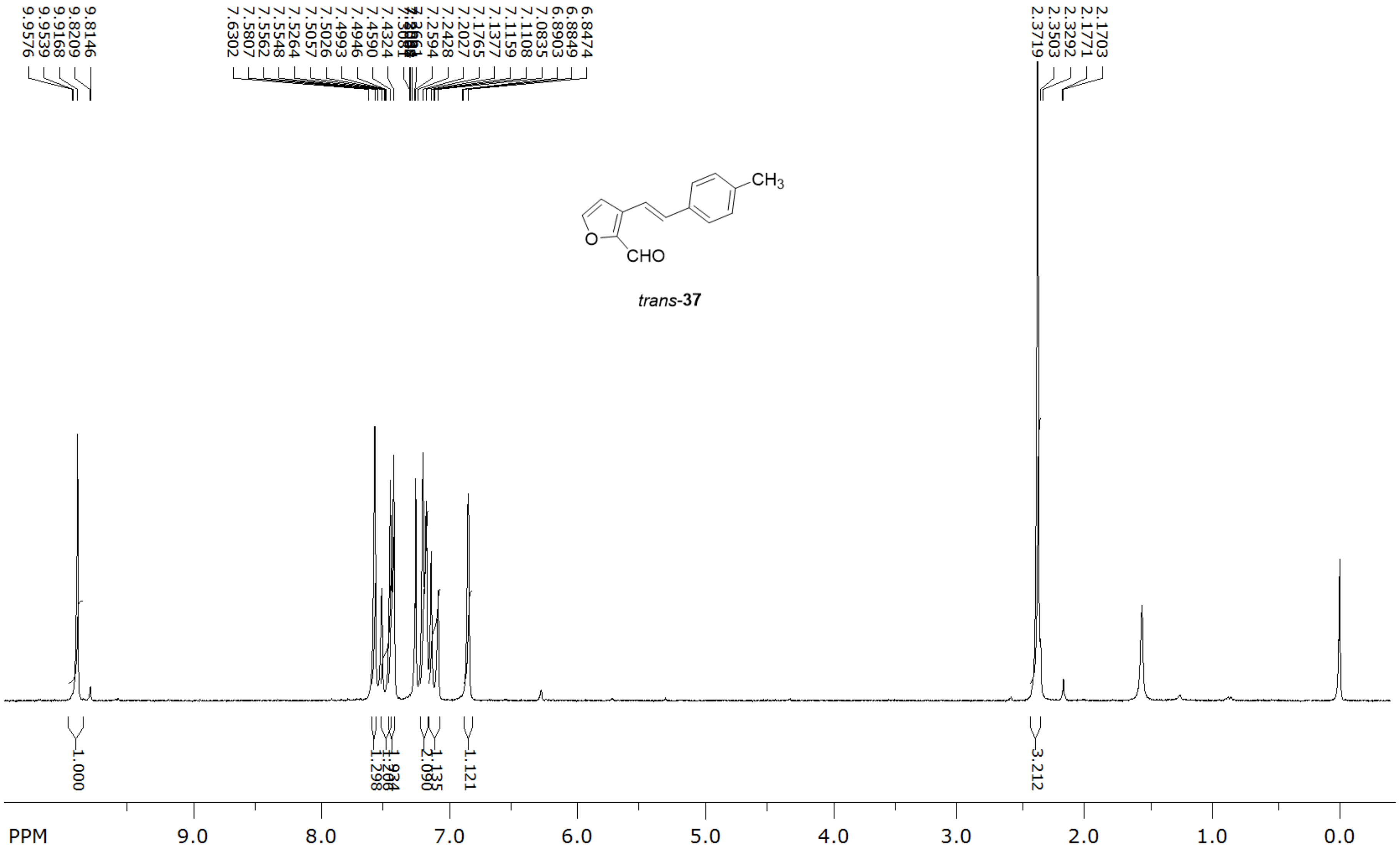

Figure S133. ^{1}H NMR ($CDCl_3$) spectrum of *trans*-**37**.

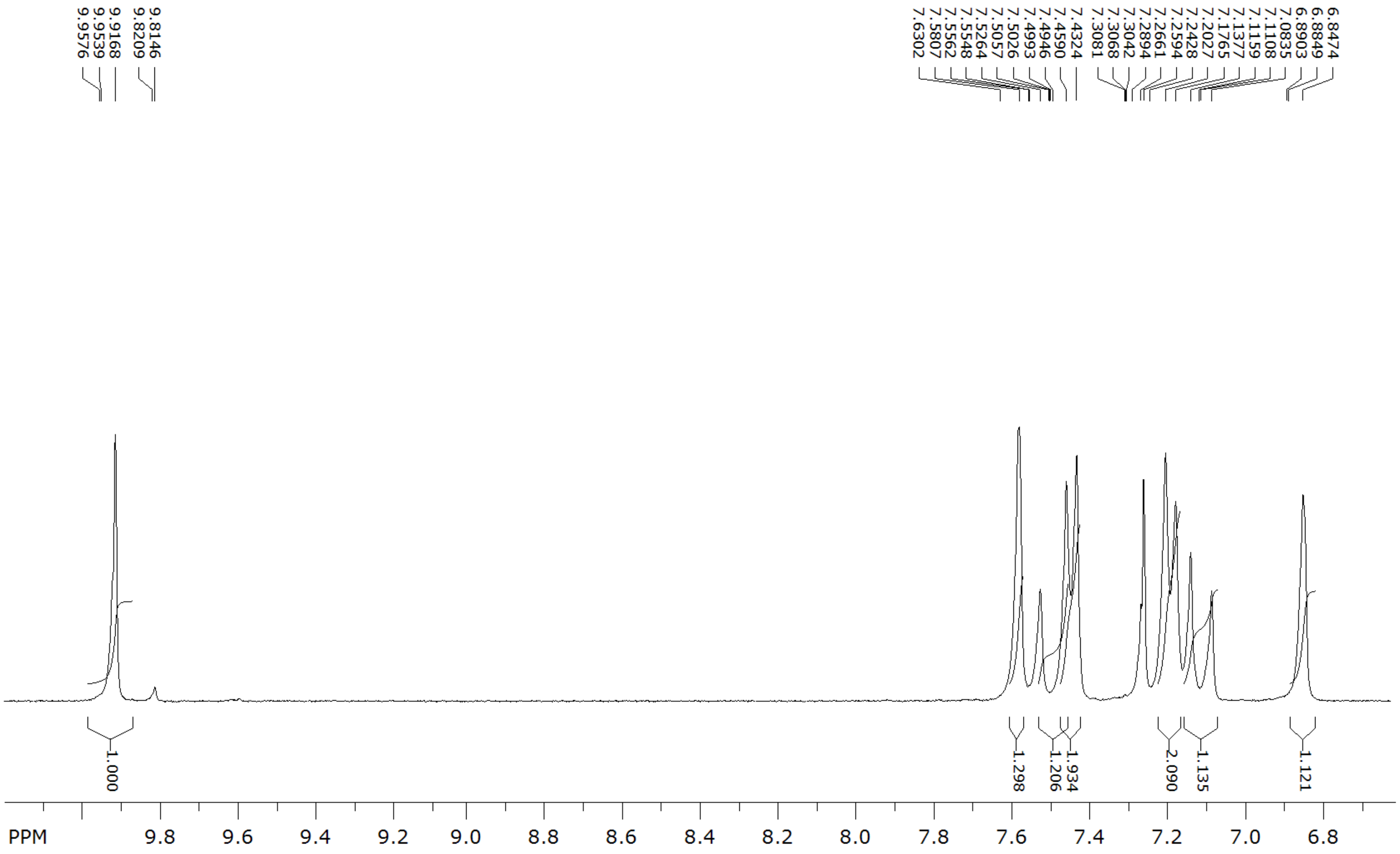

Figure S134. ^{1}H NMR ($CDCl_3$) spectrum of aromatic part of *trans*-**37**.

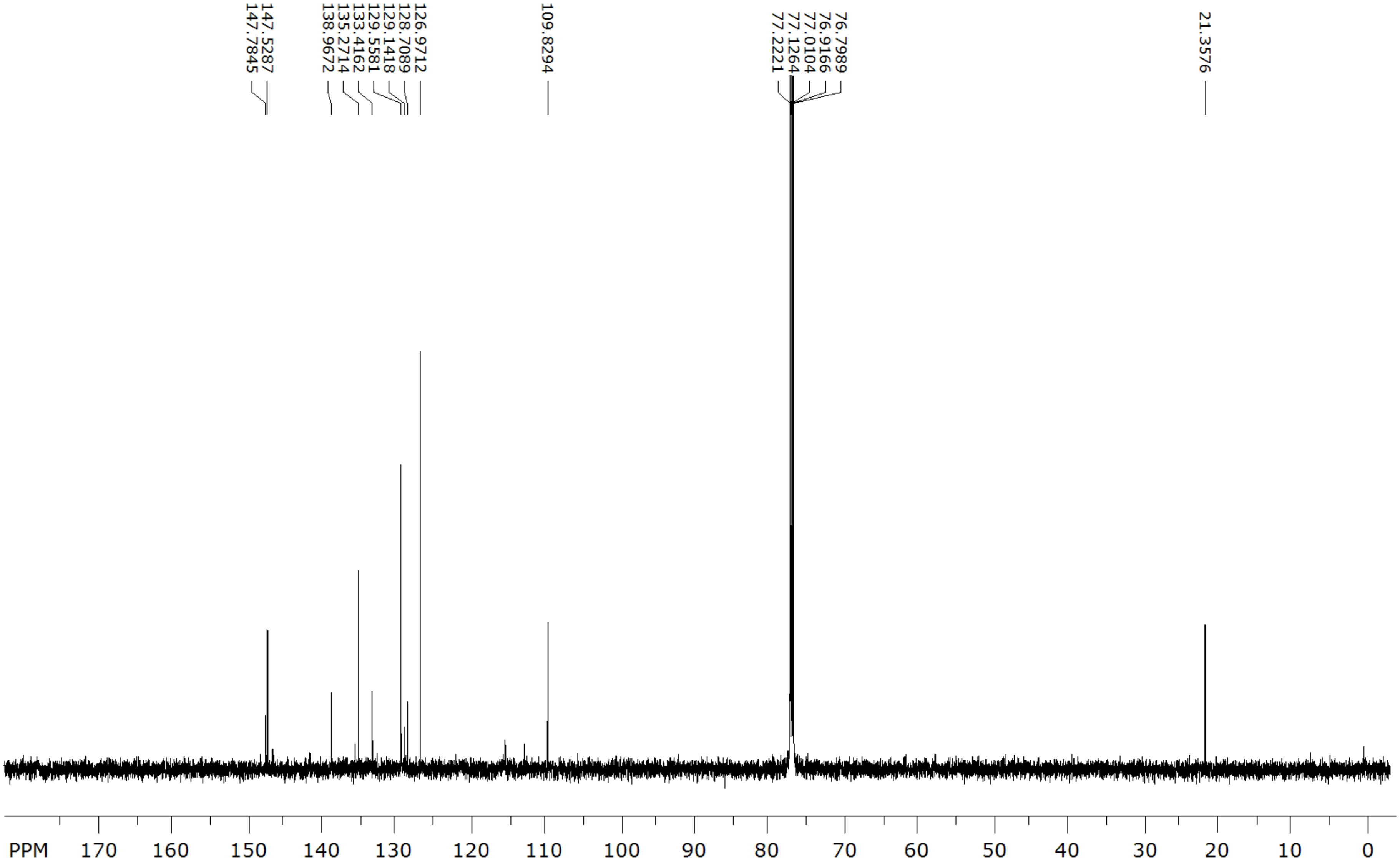

Figure S135. ^{13}C NMR ($CDCl_3$) spectrum of *trans*-**37**.

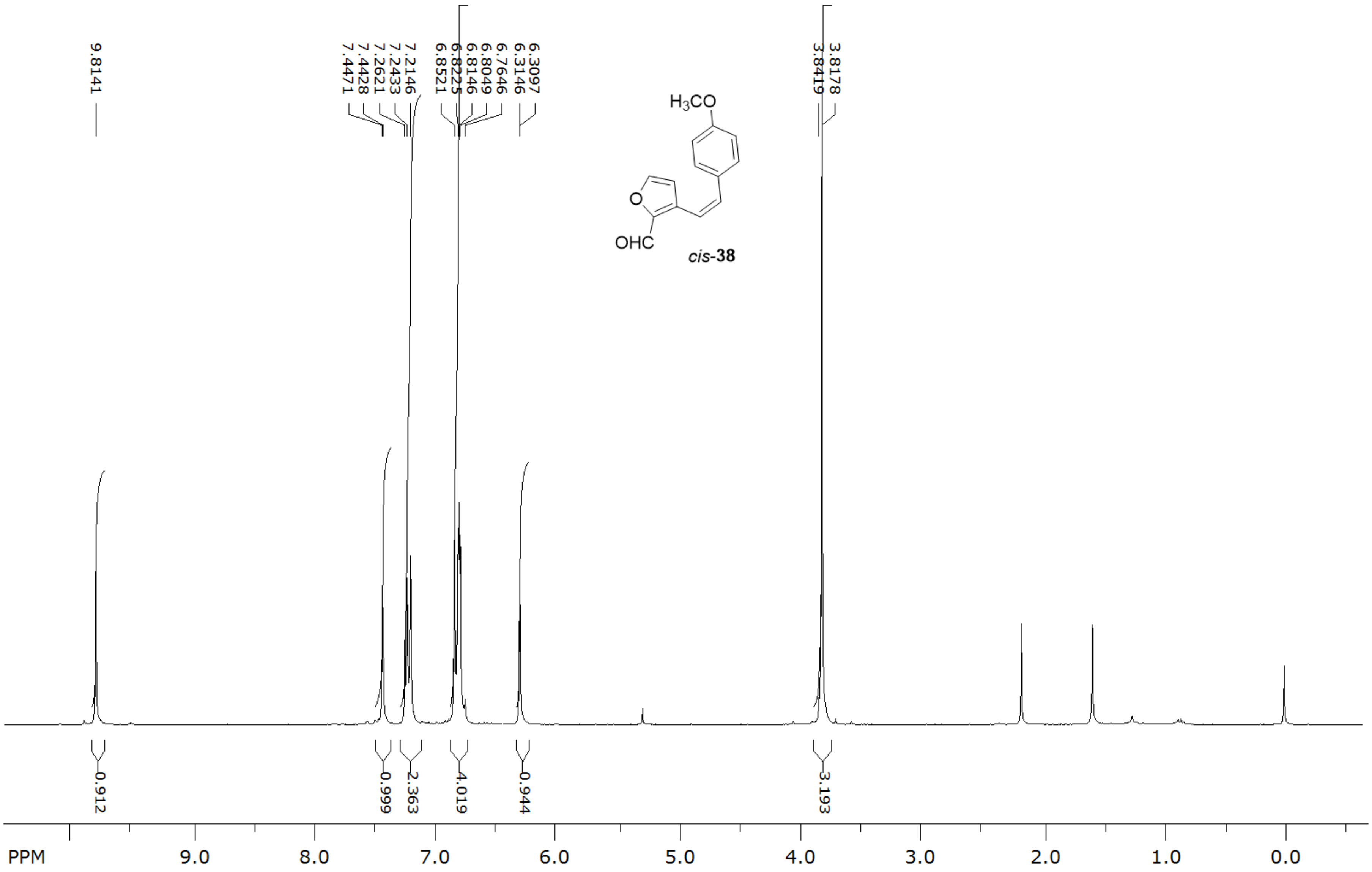

Figure S136. ^{1}H NMR ($CDCl_3$) spectrum of *cis*-**38**.

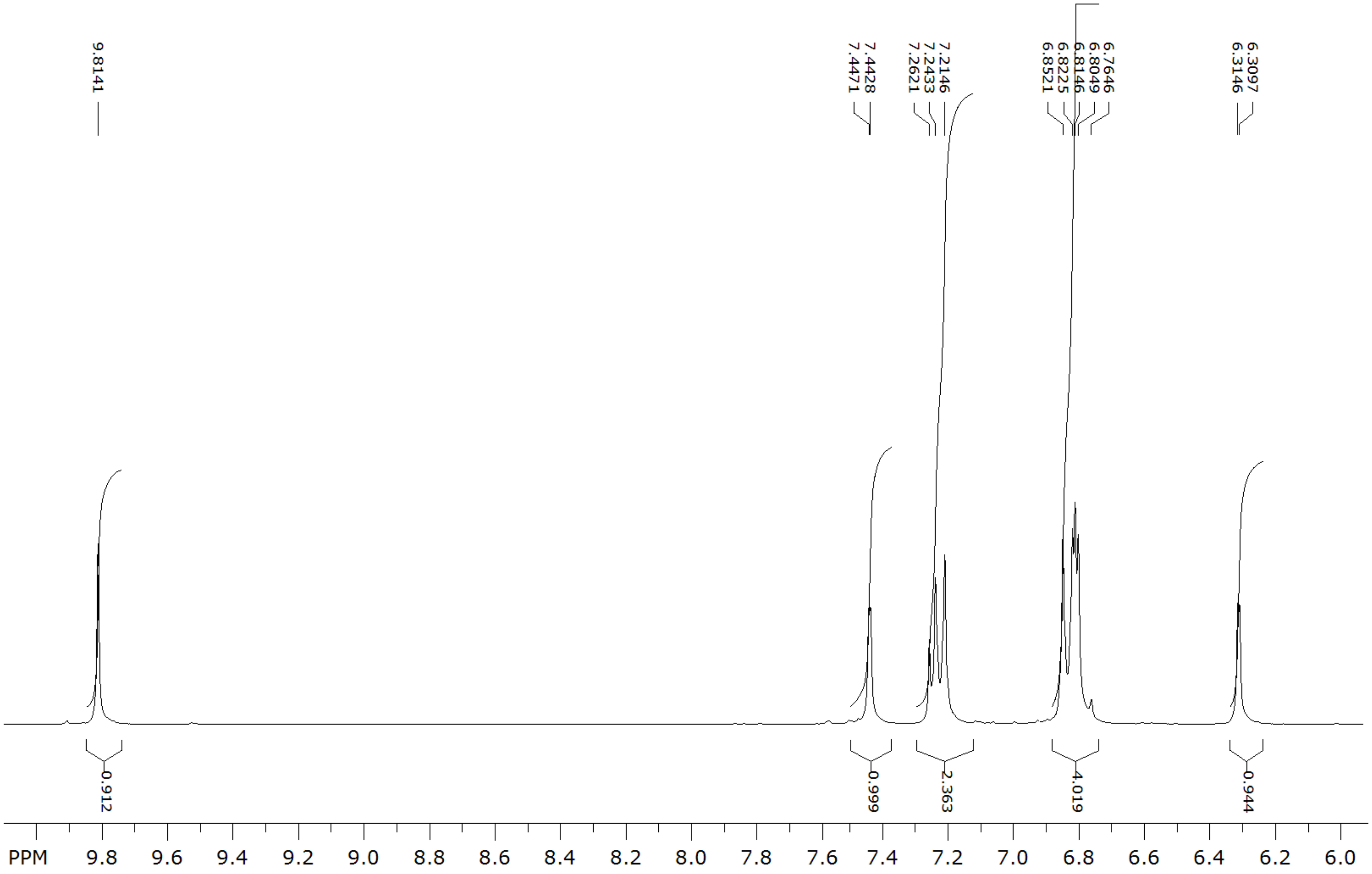

Figure S137. ^{1}H NMR ($CDCl_3$) spectrum of aromatic part of *cis*-**38**.

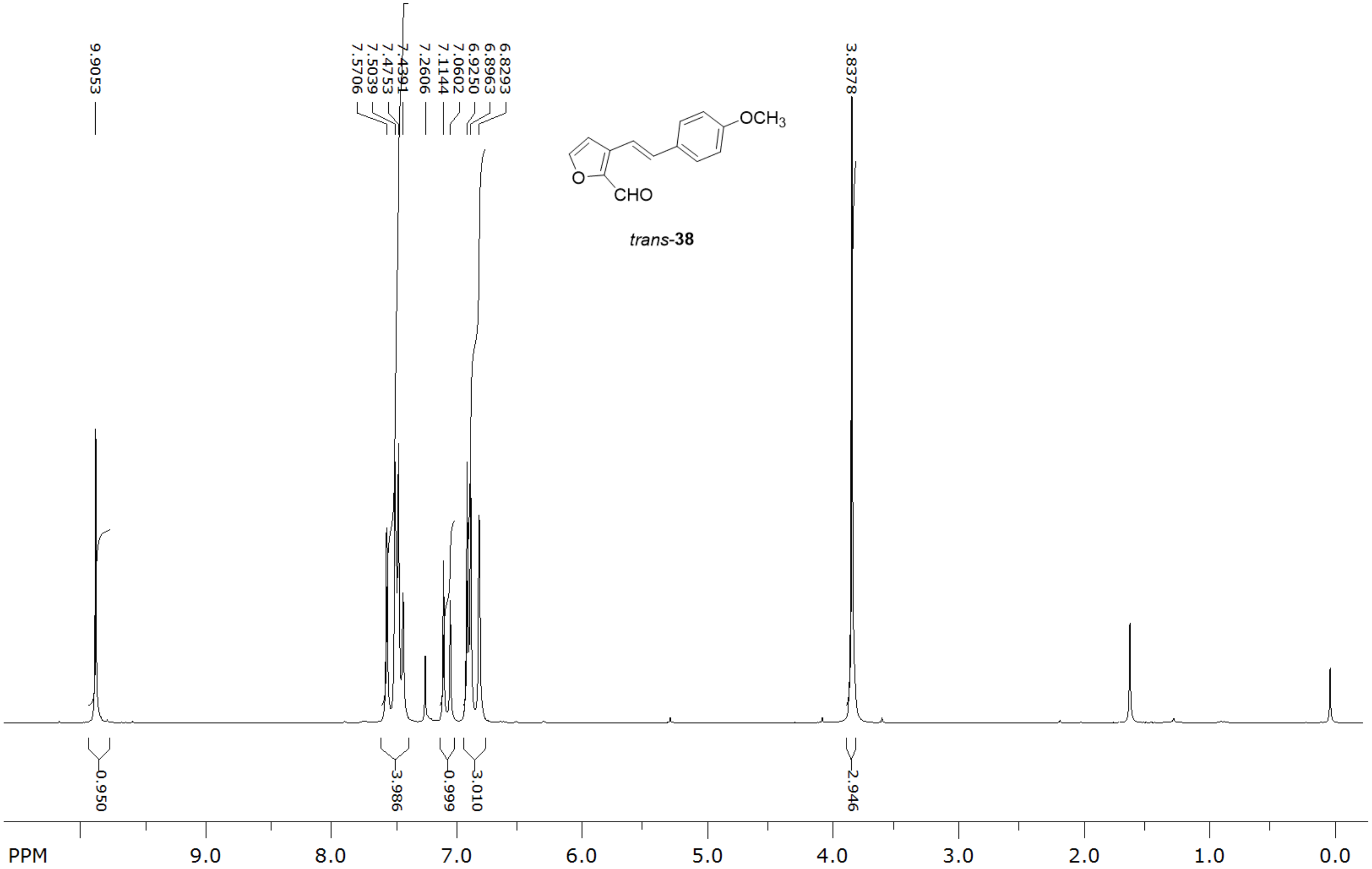

Figure S138. ^{1}H NMR ($CDCl_3$) spectrum of *trans*-**38**.

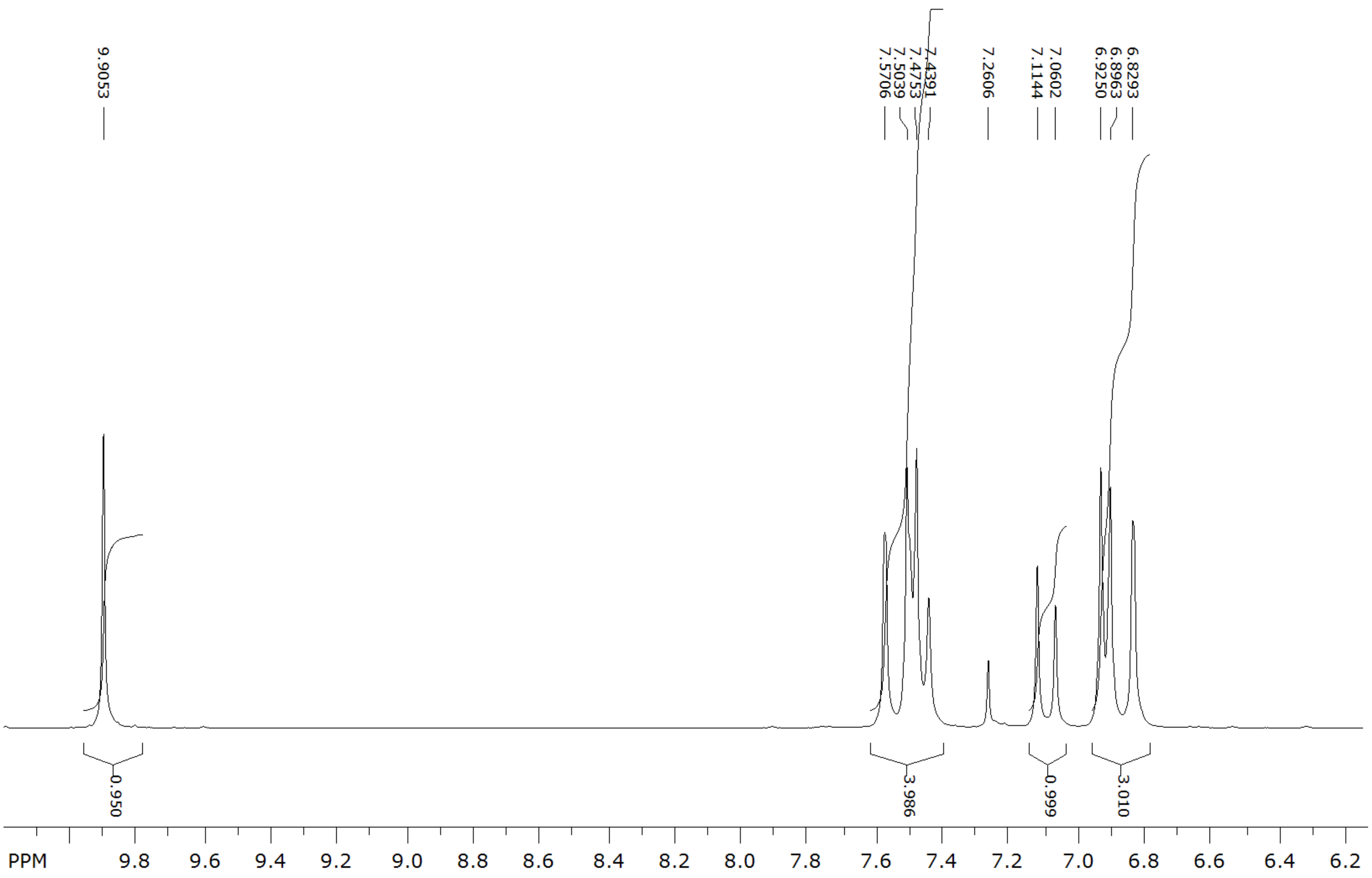

Figure S139. ^{1}H NMR ($CDCl_3$) spectrum of aromatic part of *trans*-**38**.

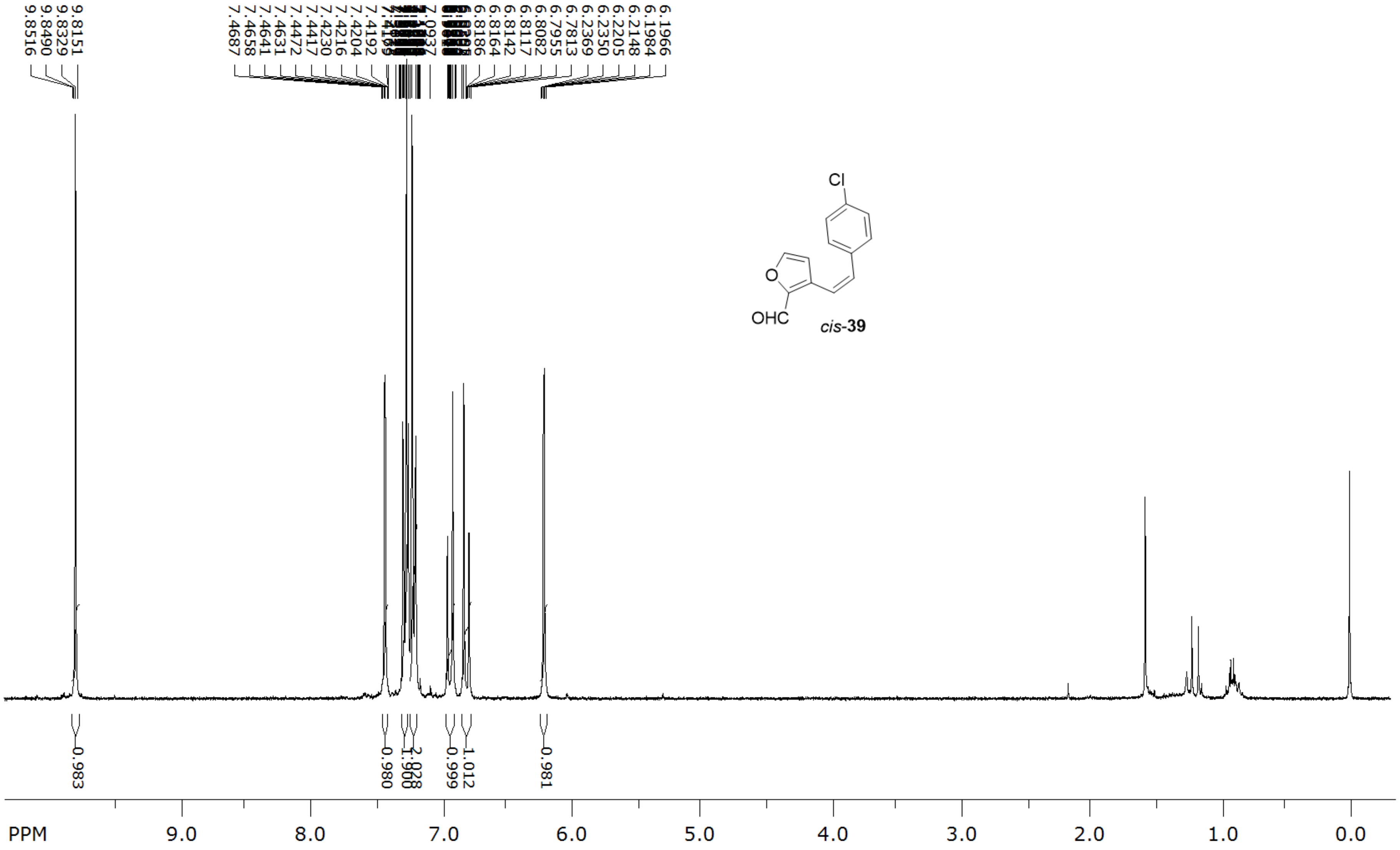

Figure S140. ^{1}H NMR ($CDCl_3$) spectrum of *cis*-**39**.

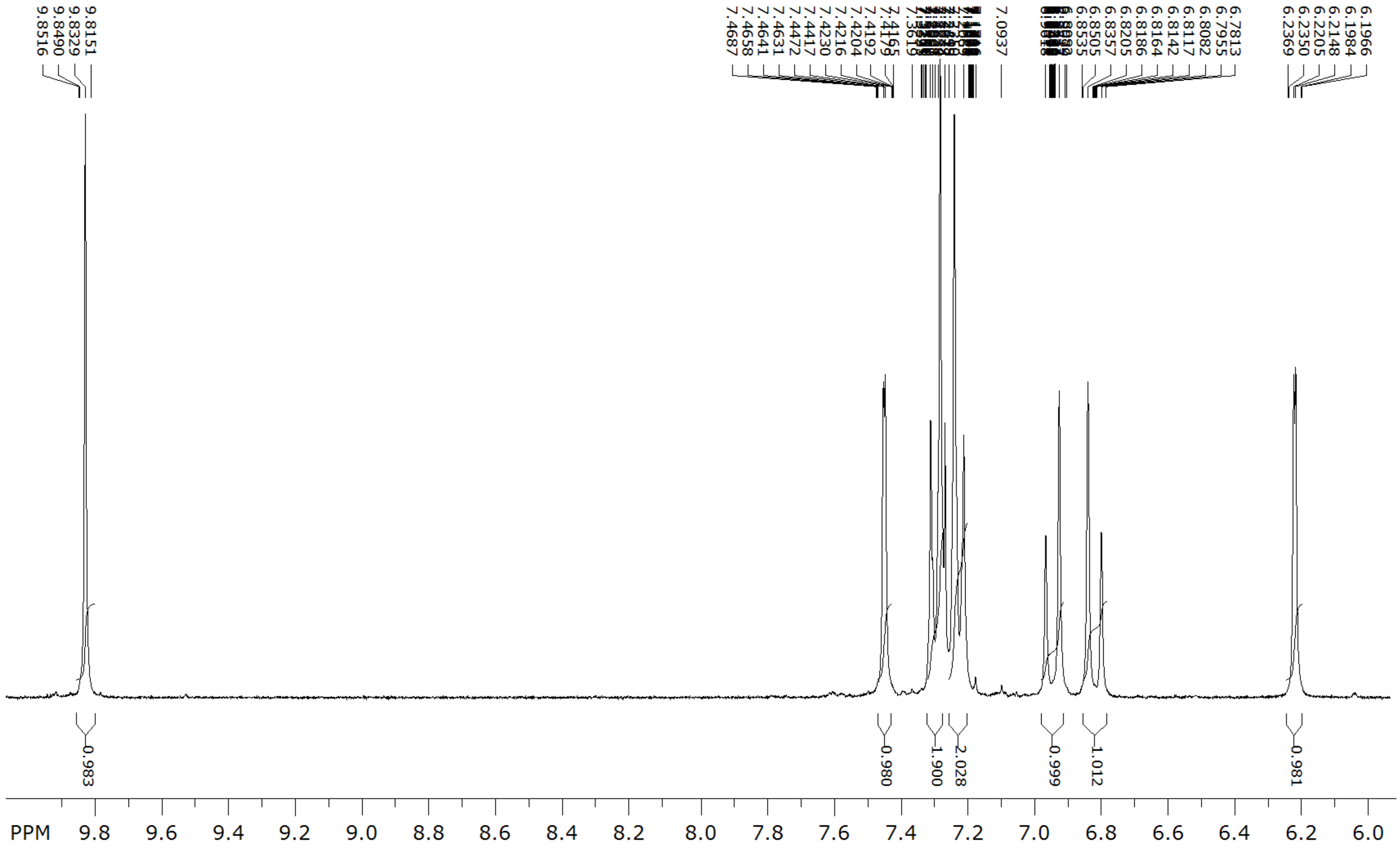

Figure S141. ^{1}H NMR ($CDCl_3$) spectrum of aromatic part of *cis*-**39**.

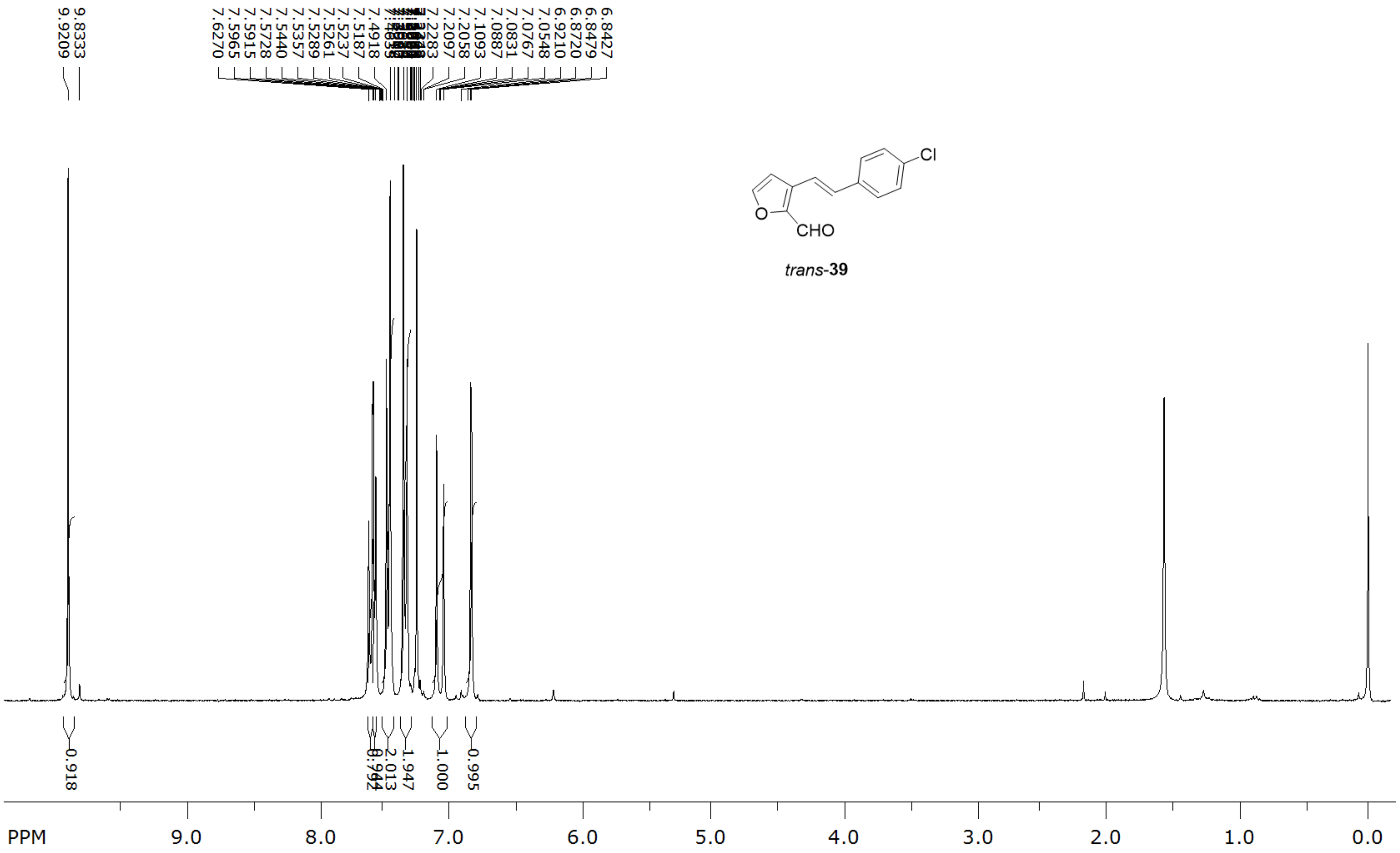

Figure S142. ^{1}H NMR ($CDCl_3$) spectrum of *trans*-**39**.

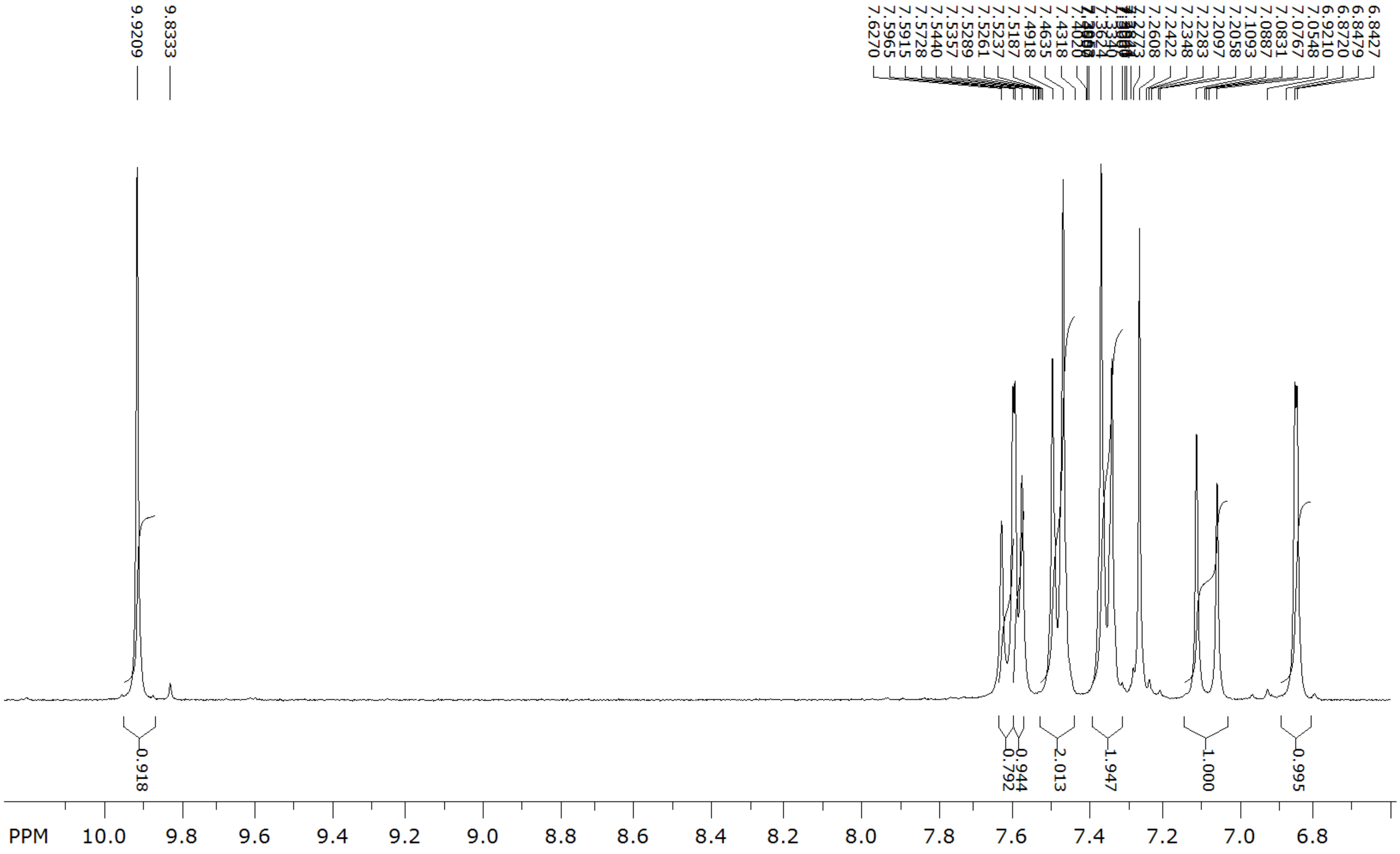

Figure S143. ^{1}H NMR ($CDCl_3$) spectrum of aromatic part of *trans*-**39**.

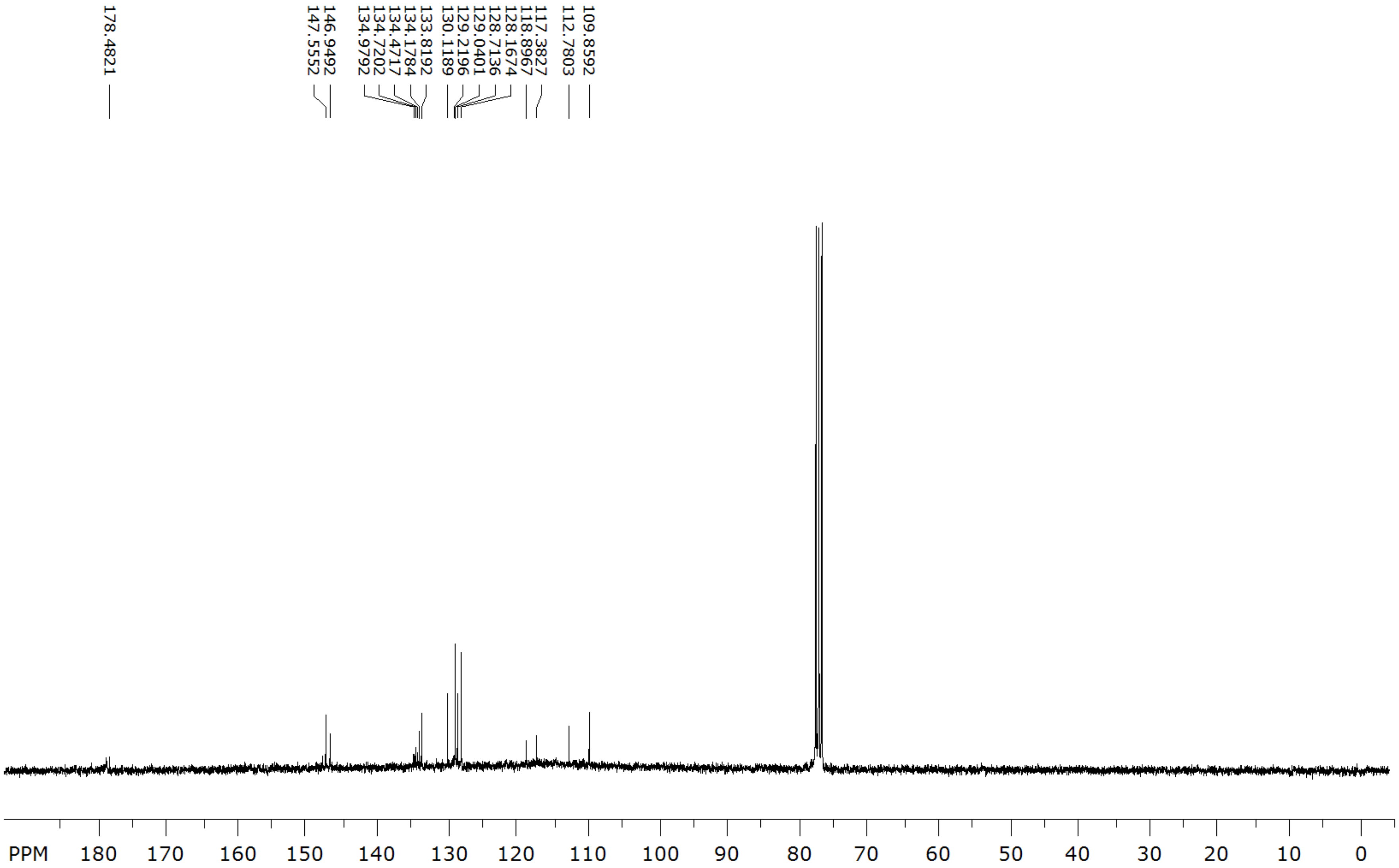

Figure S144. ^{13}H NMR ($CDCl_3$) spectrum of *trans*-**39**.

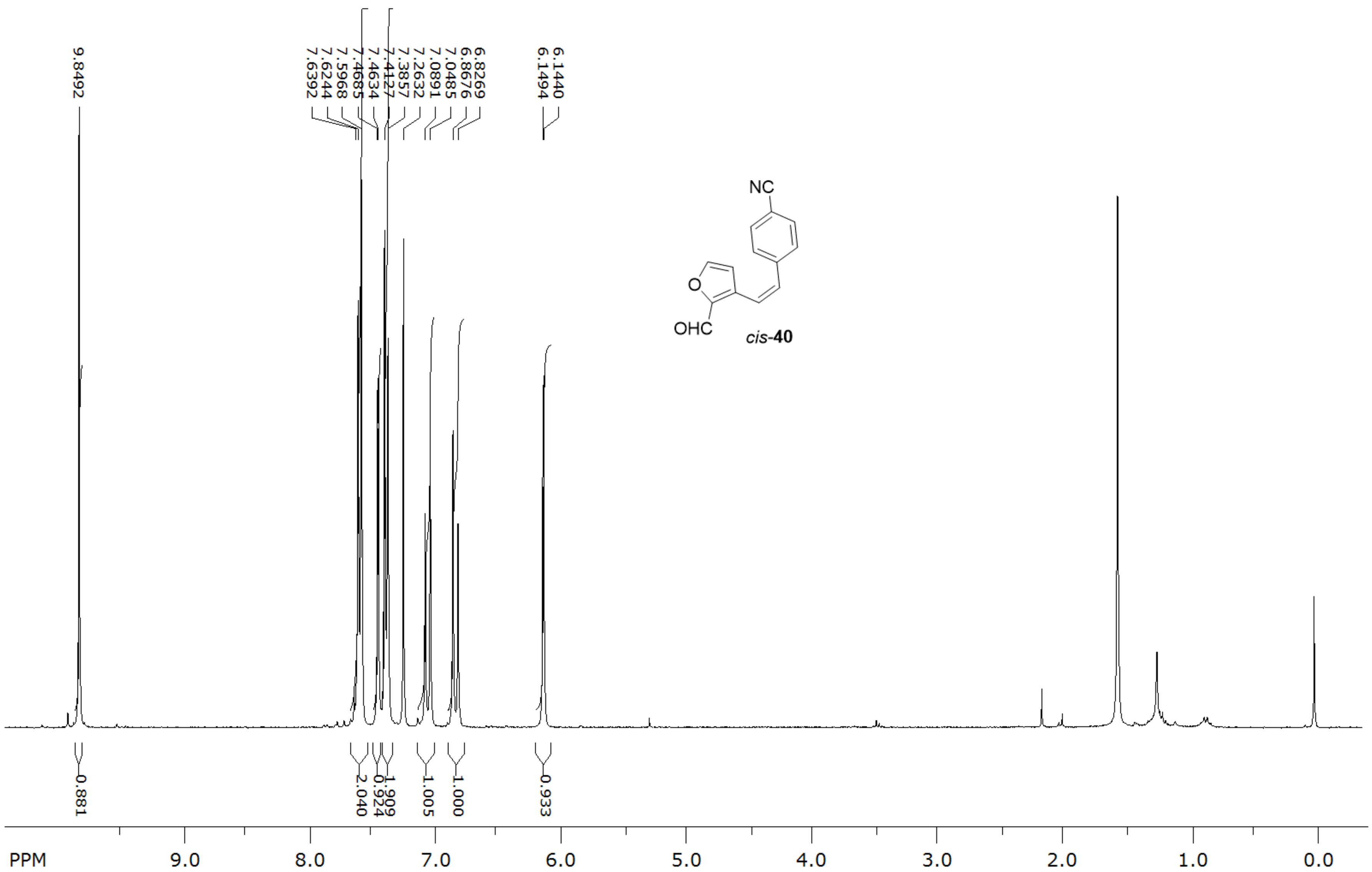

Figure S145. ^{1}H NMR ($CDCl_3$) spectrum of *cis*-**40**.

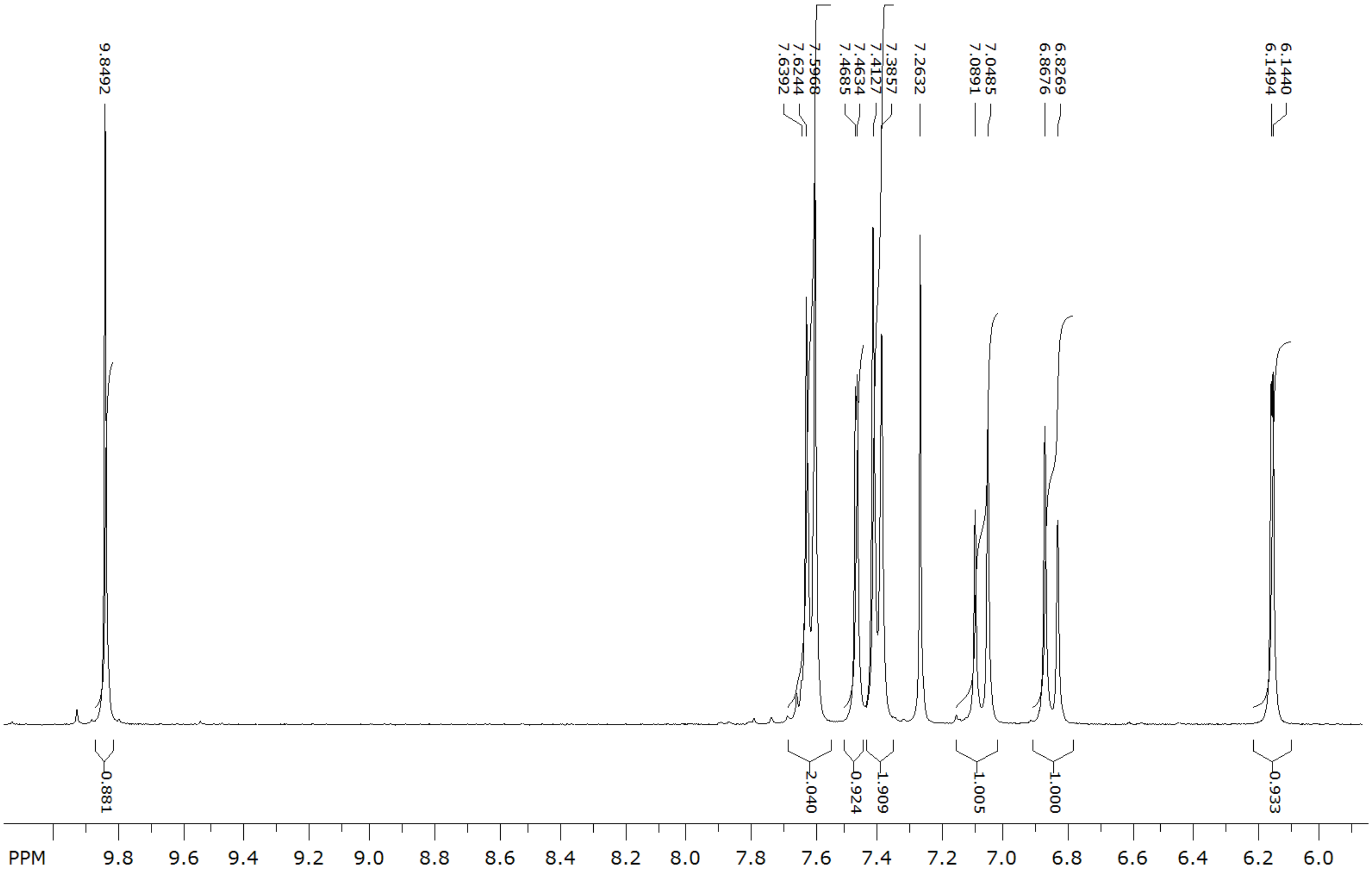

Figure S146. ^{1}H NMR ($CDCl_3$) spectrum of aromatic part of *cis*-**40**.

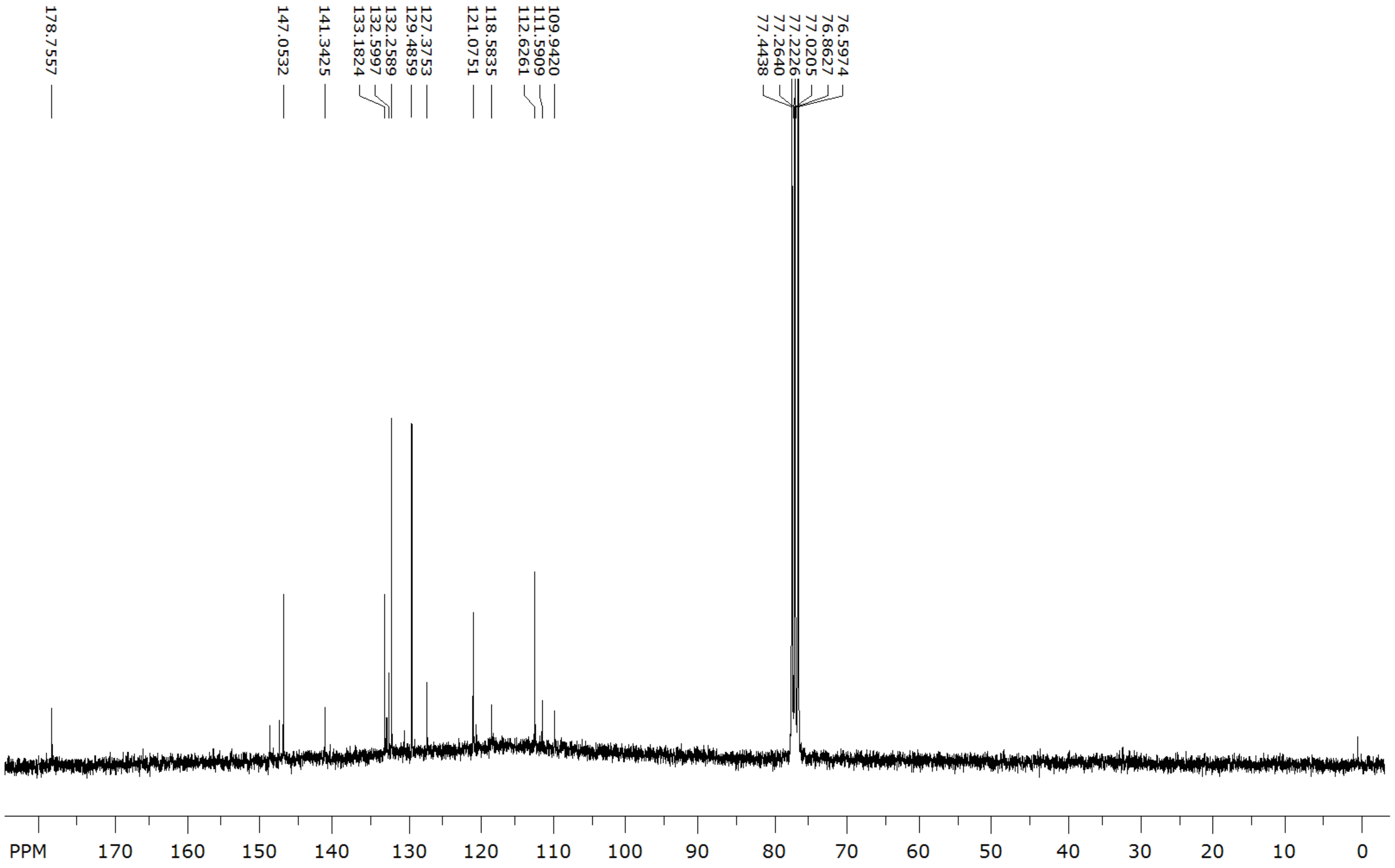

Figure S147. ^{13}C NMR ($CDCl_3$) spectrum of *cis*-**40**.

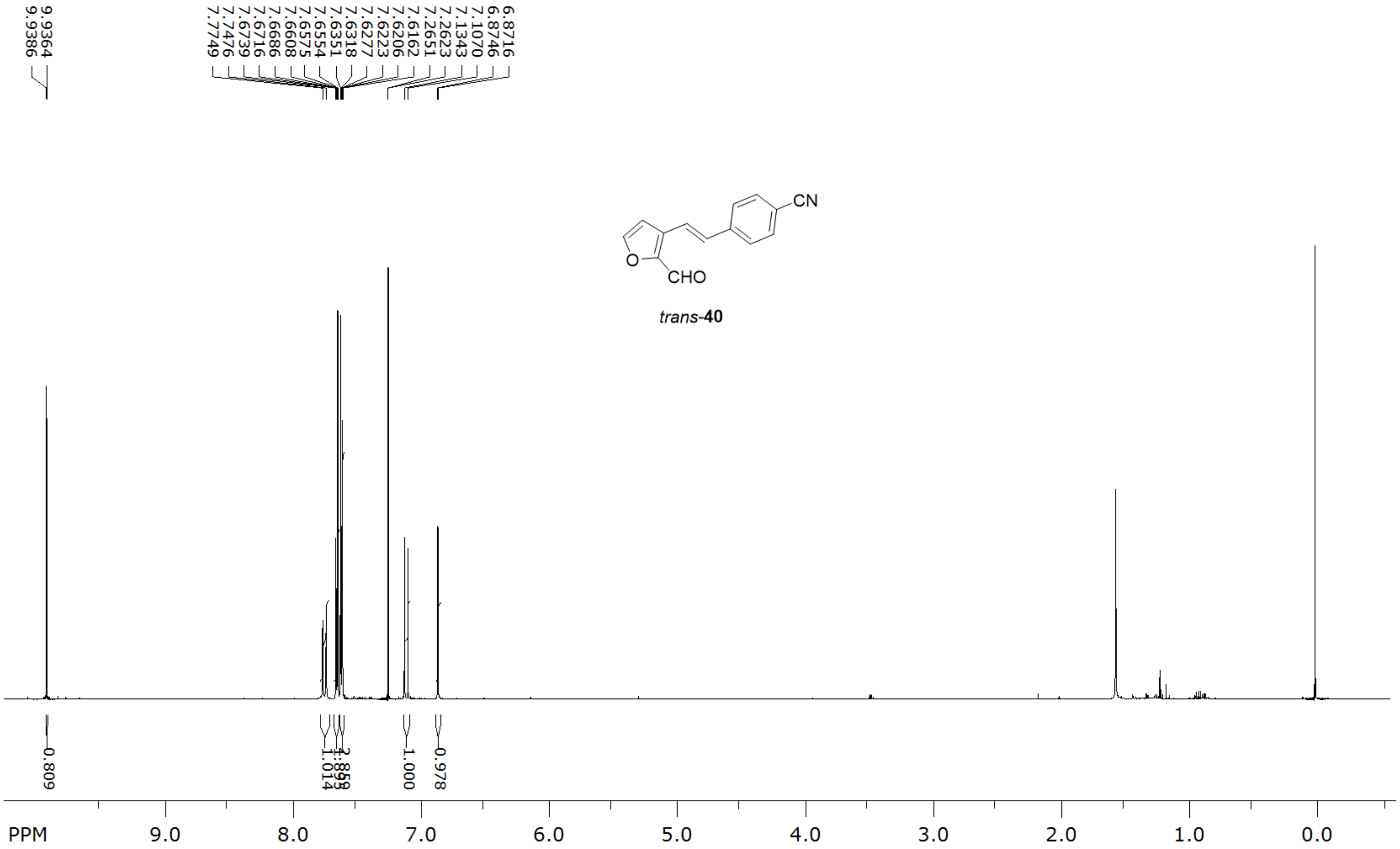

Figure S148. ^{1}H NMR ($CDCl_3$) spectrum of *trans*-**40**.

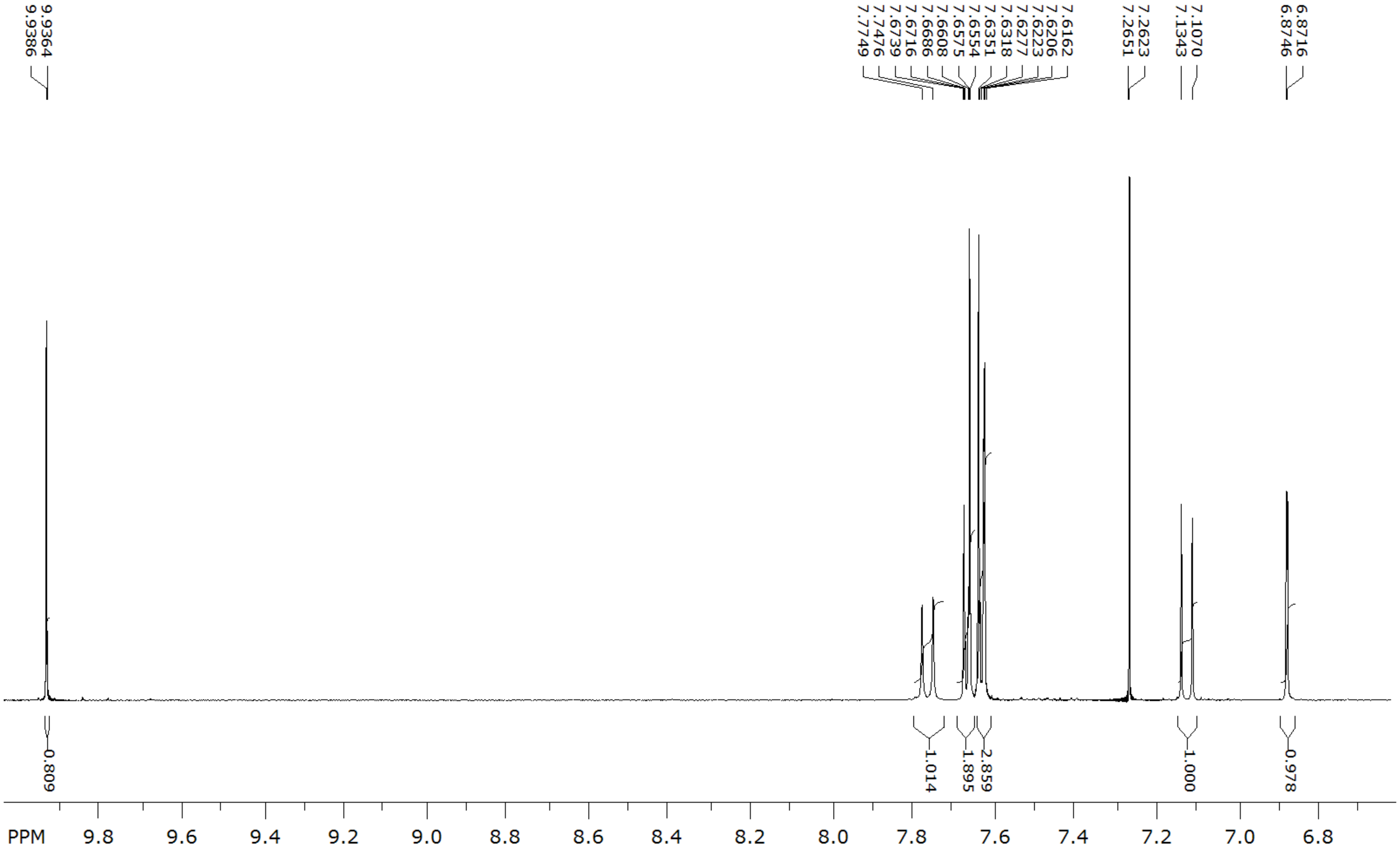

Figure S149. ^{1}H NMR ($CDCl_3$) spectrum of aromatic part of *trans*-**40**.

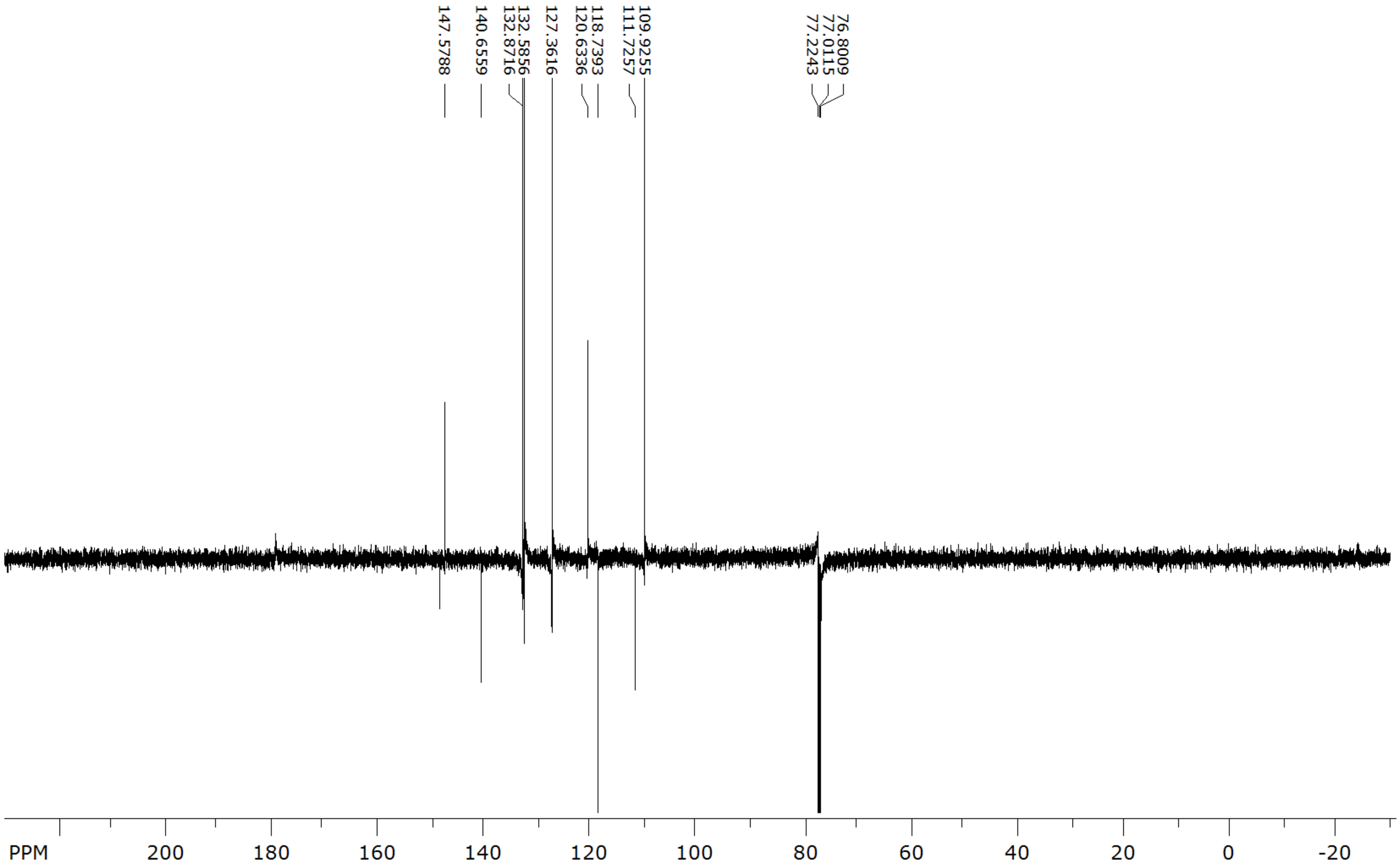

Figure S150. ^{13}C NMR ($CDCl_3$) spectrum of *trans*-**40**.

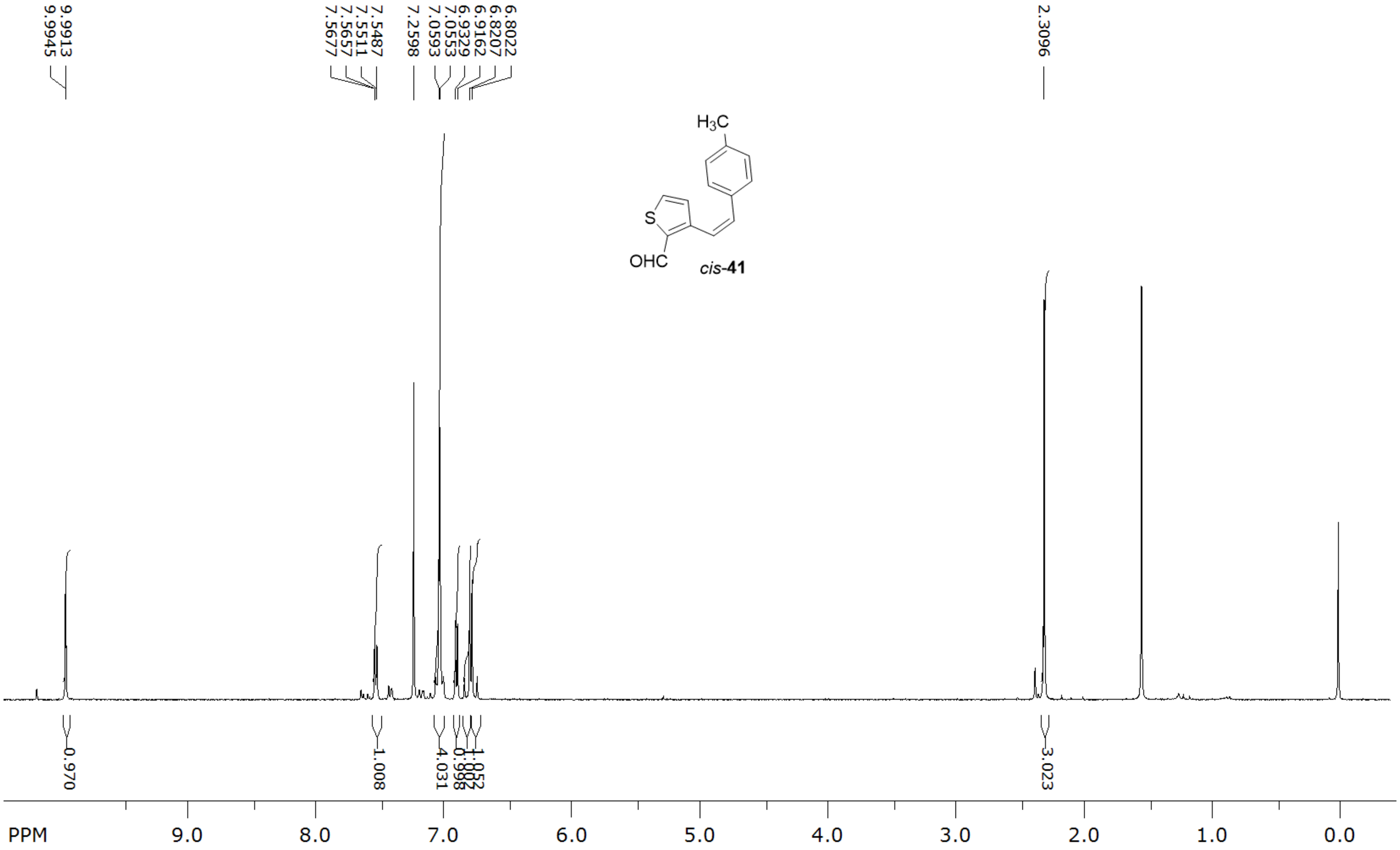

Figure S151. ^{1}H NMR ($CDCl_3$) spectrum of *cis*-**41**.

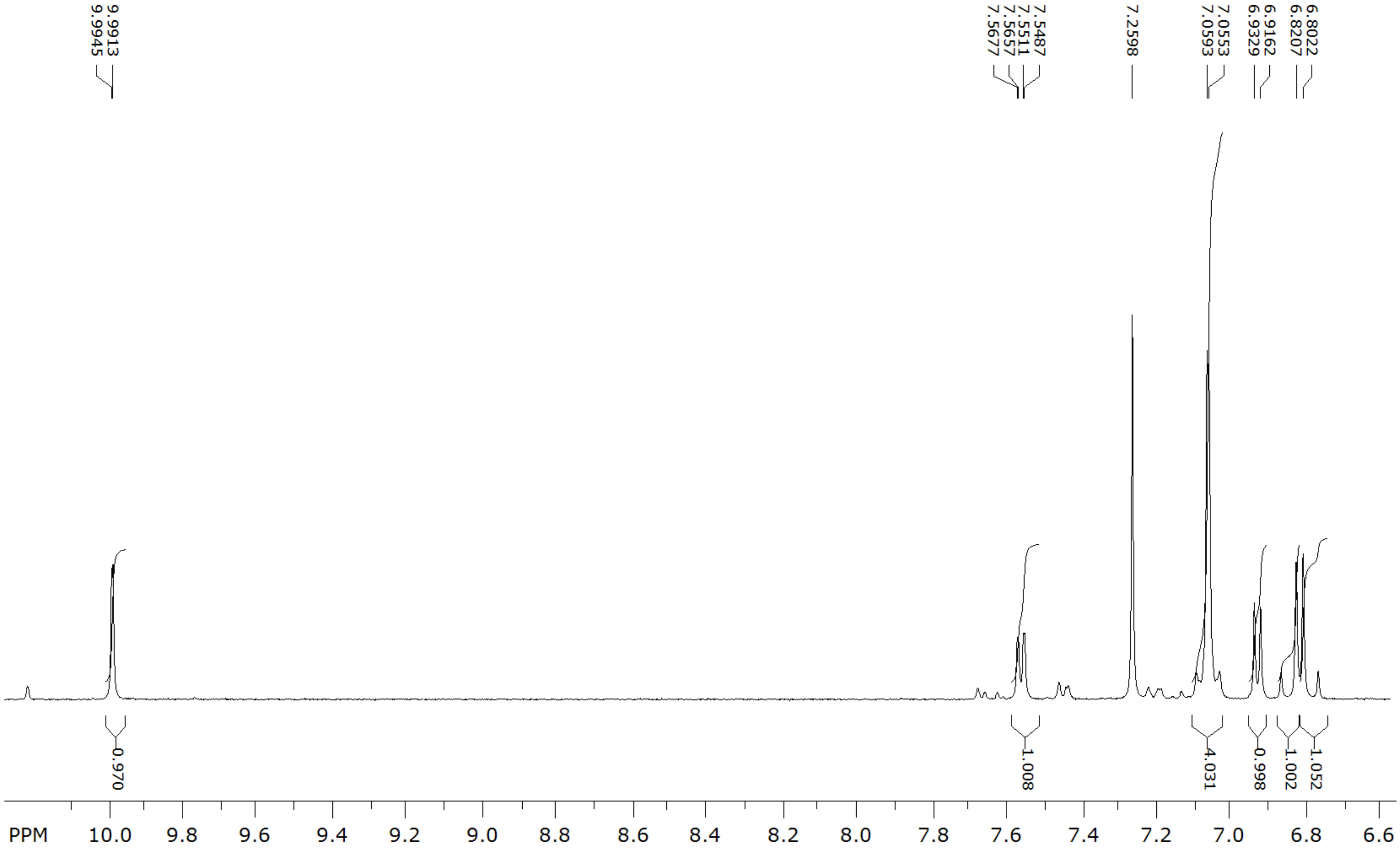

Figure S152. ¹H NMR ($CDCl_3$) spectrum of aromatic part of *cis*-**41**.

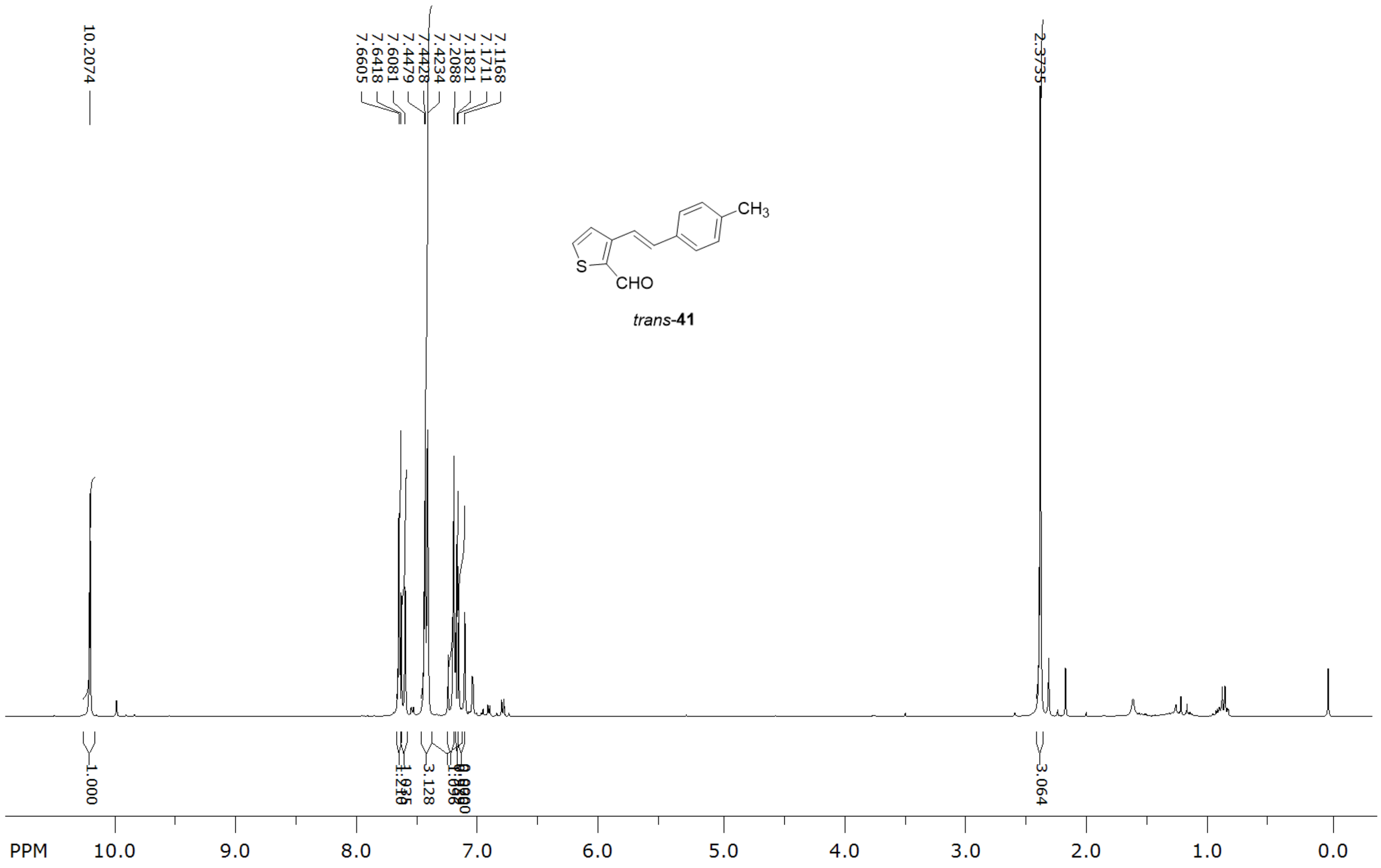

Figure S153. ^{1}H NMR ($CDCl_3$) spectrum of *trans*-**41**.

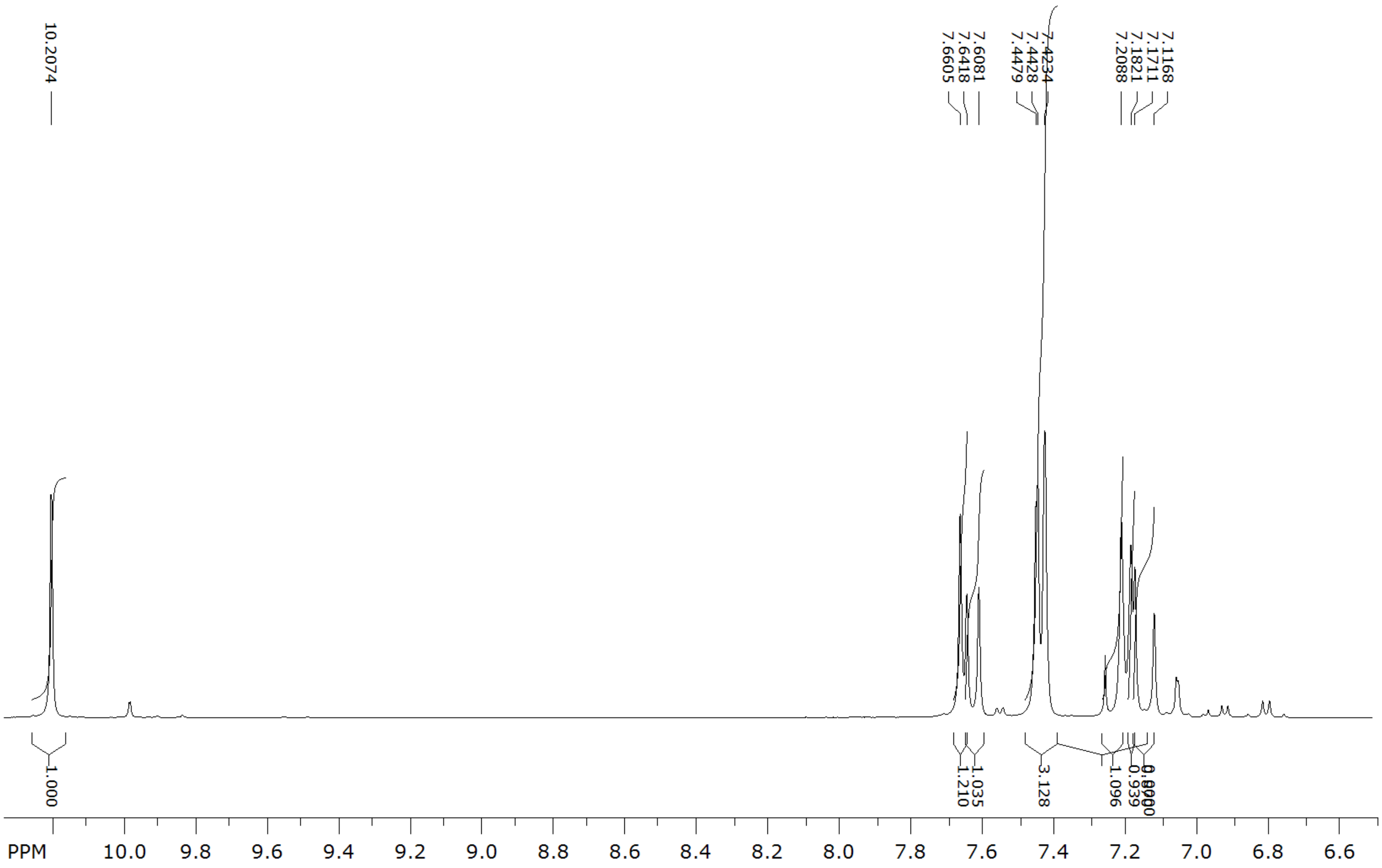

Figure S154. ^{1}H NMR ($CDCl_3$) spectrum of aromatic part of *trans*-**41**.

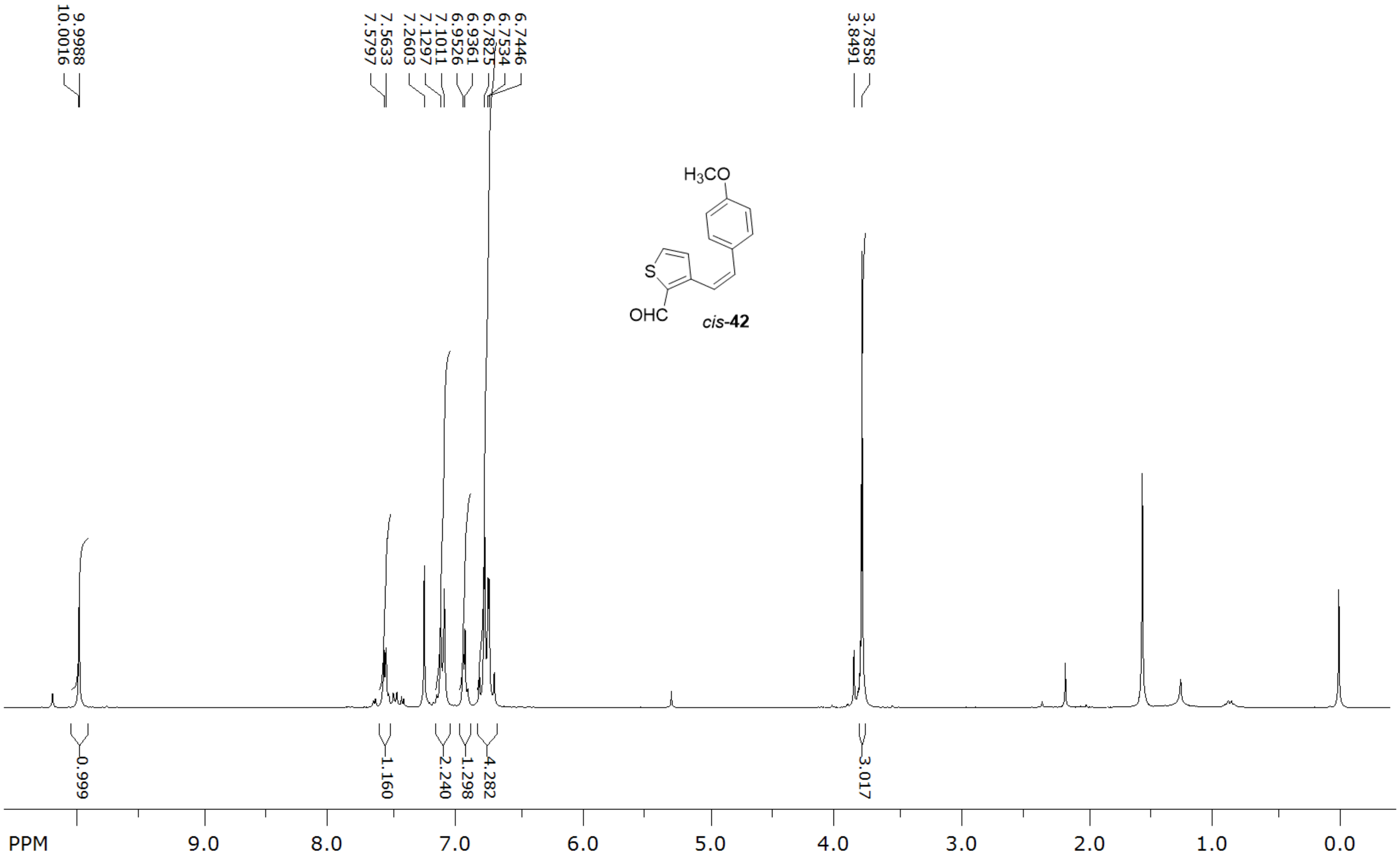

Figure S155. ^{1}H NMR ($CDCl_3$) spectrum of *cis*-**42**.

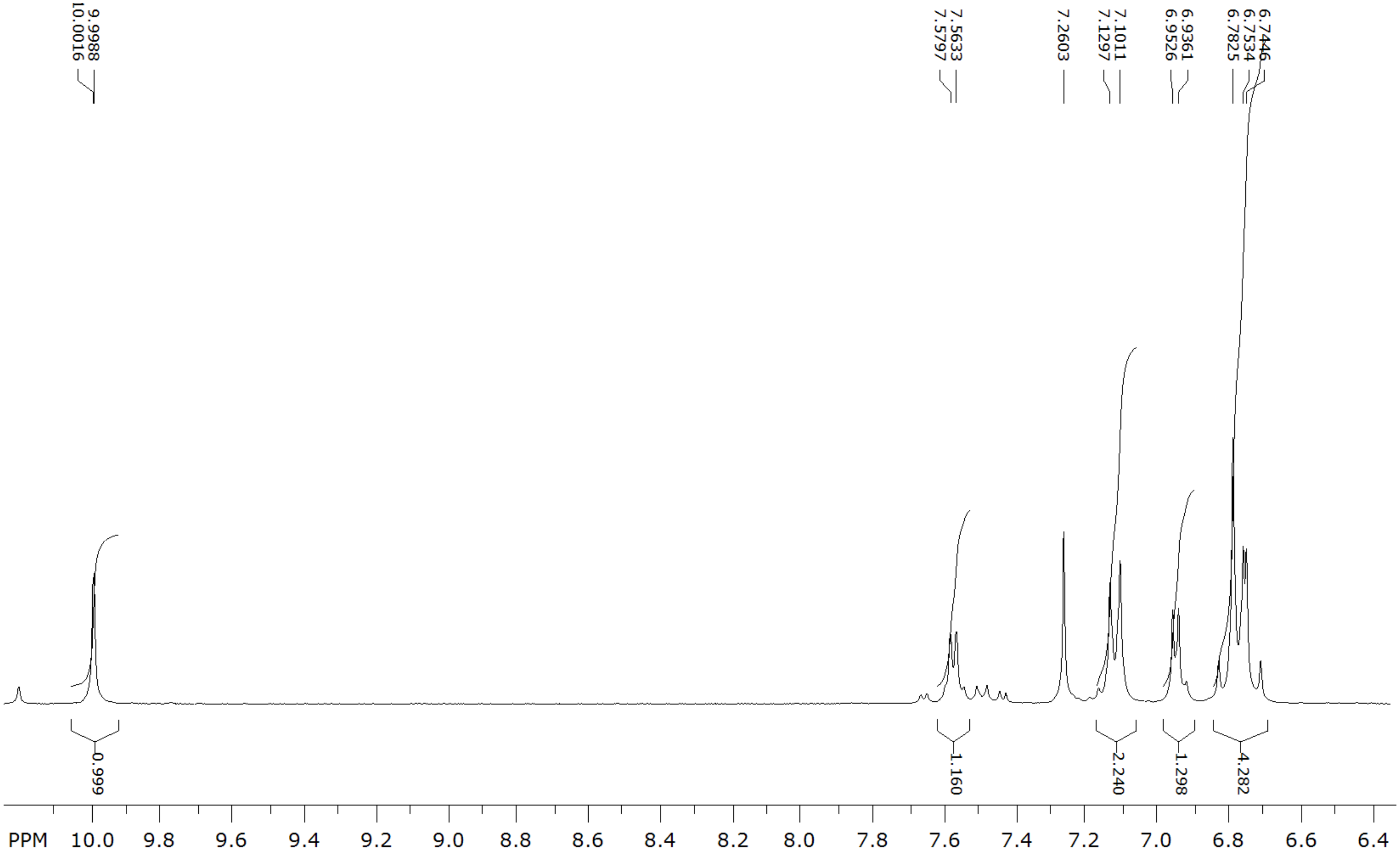

Figure S156. ^{1}H NMR ($CDCl_3$) spectrum of aromatic part of *cis*-**42**.

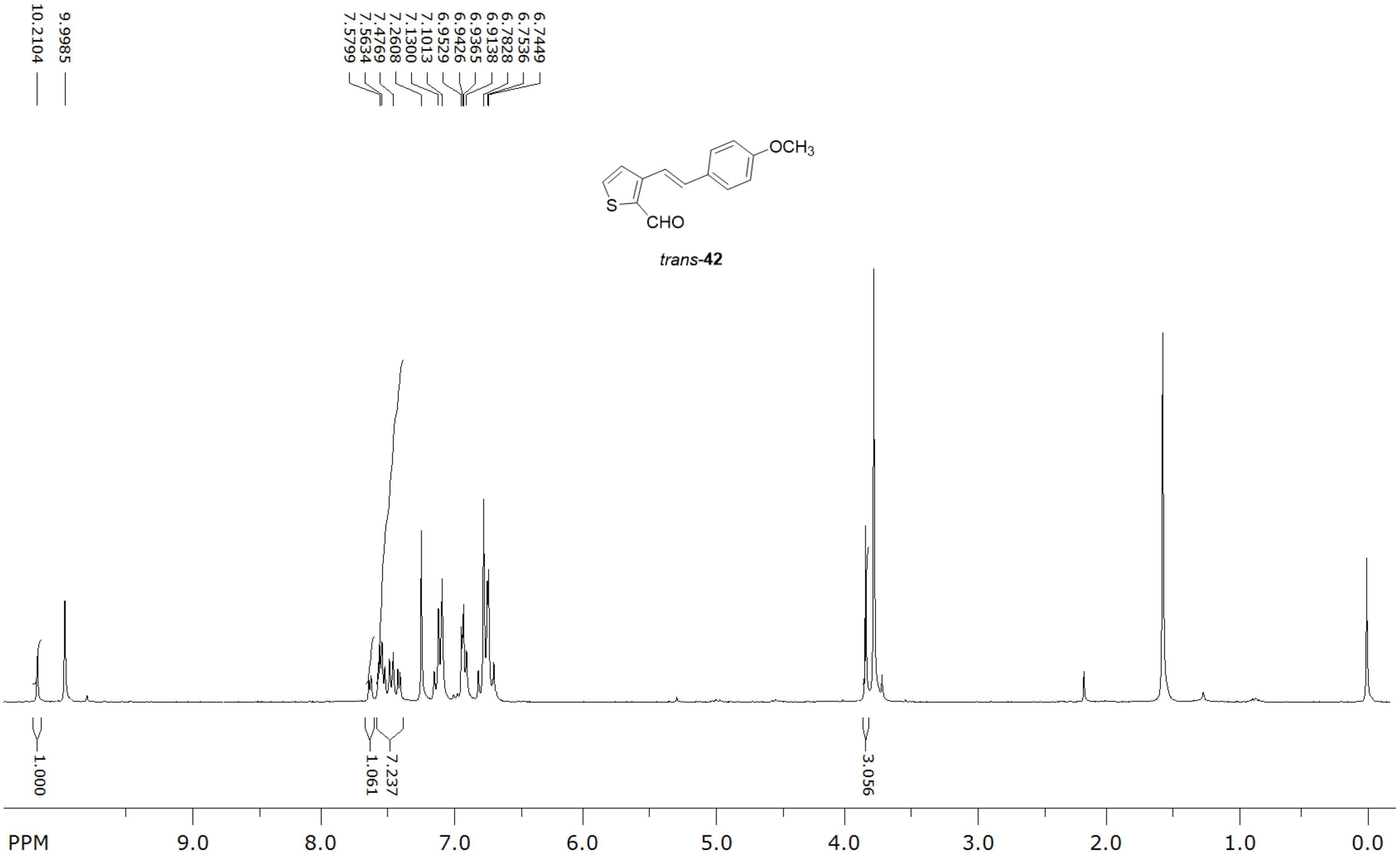

Figure S157. ^{1}H NMR ($CDCl_3$) spectrum of *trans*-**42**.

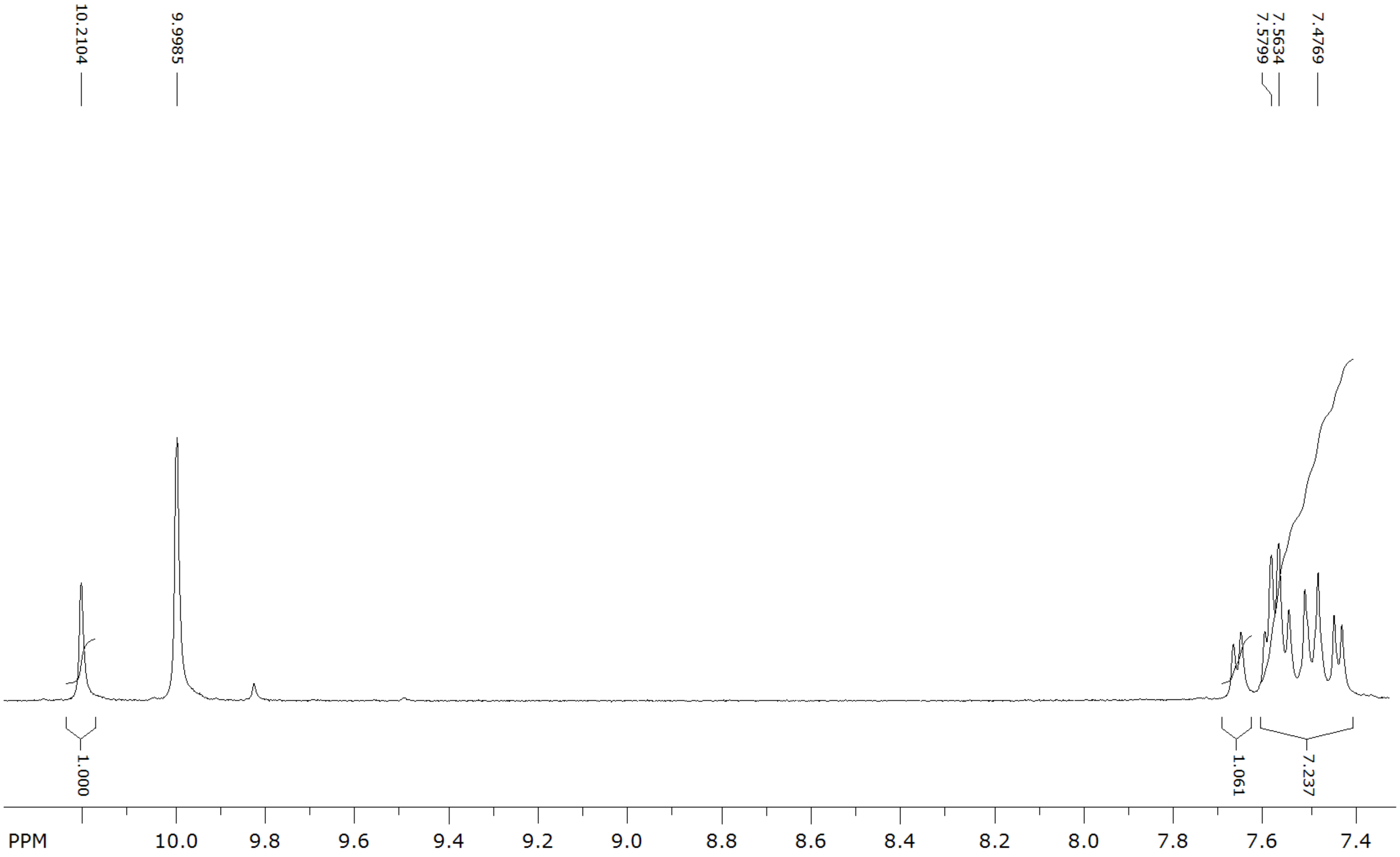

Figure S158. ^{1}H NMR ($CDCl_3$) spectrum of aromatic part of *trans*-**42**.

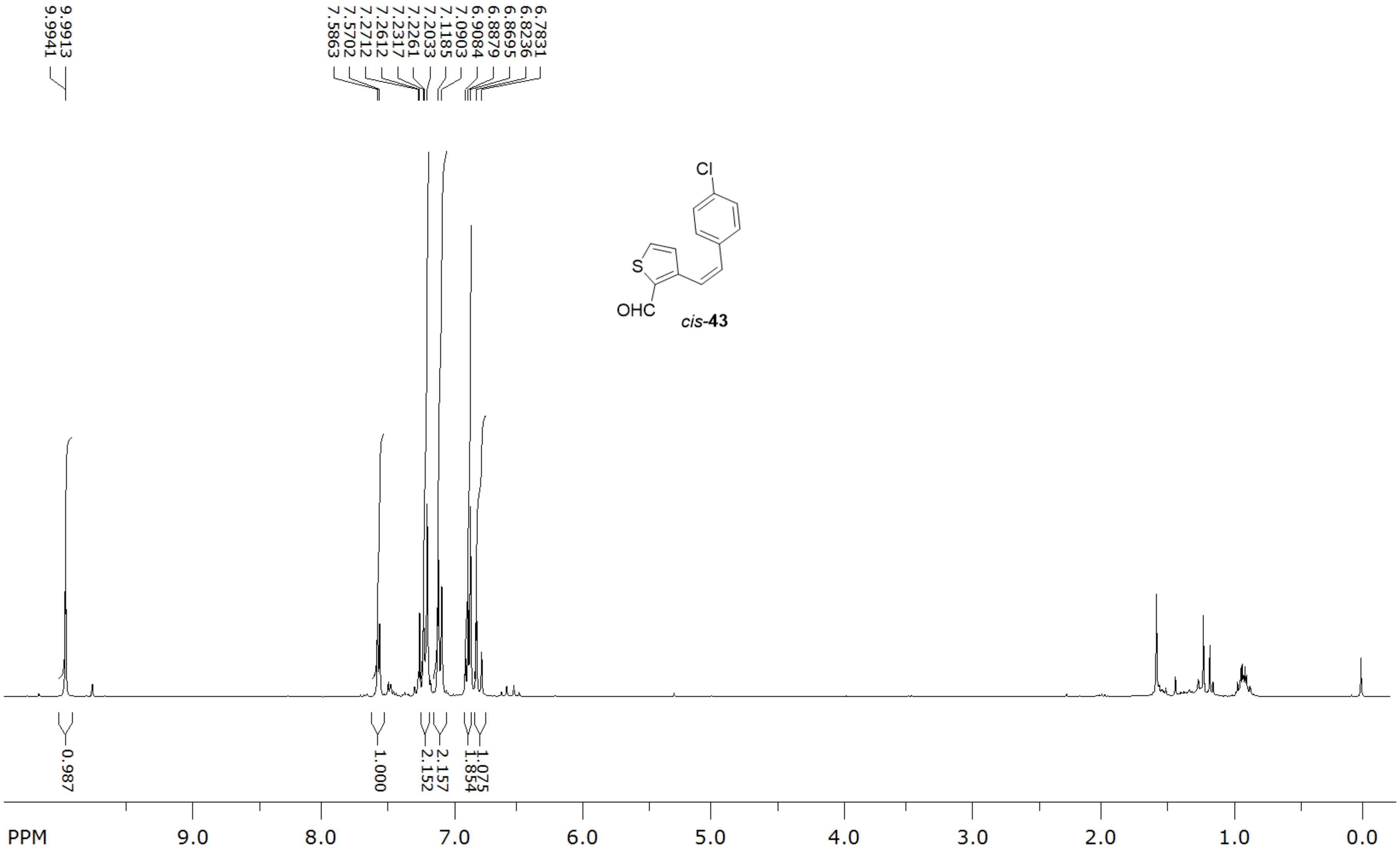

Figure S159. ^{1}H NMR ($CDCl_3$) spectrum of *cis*-**43**.

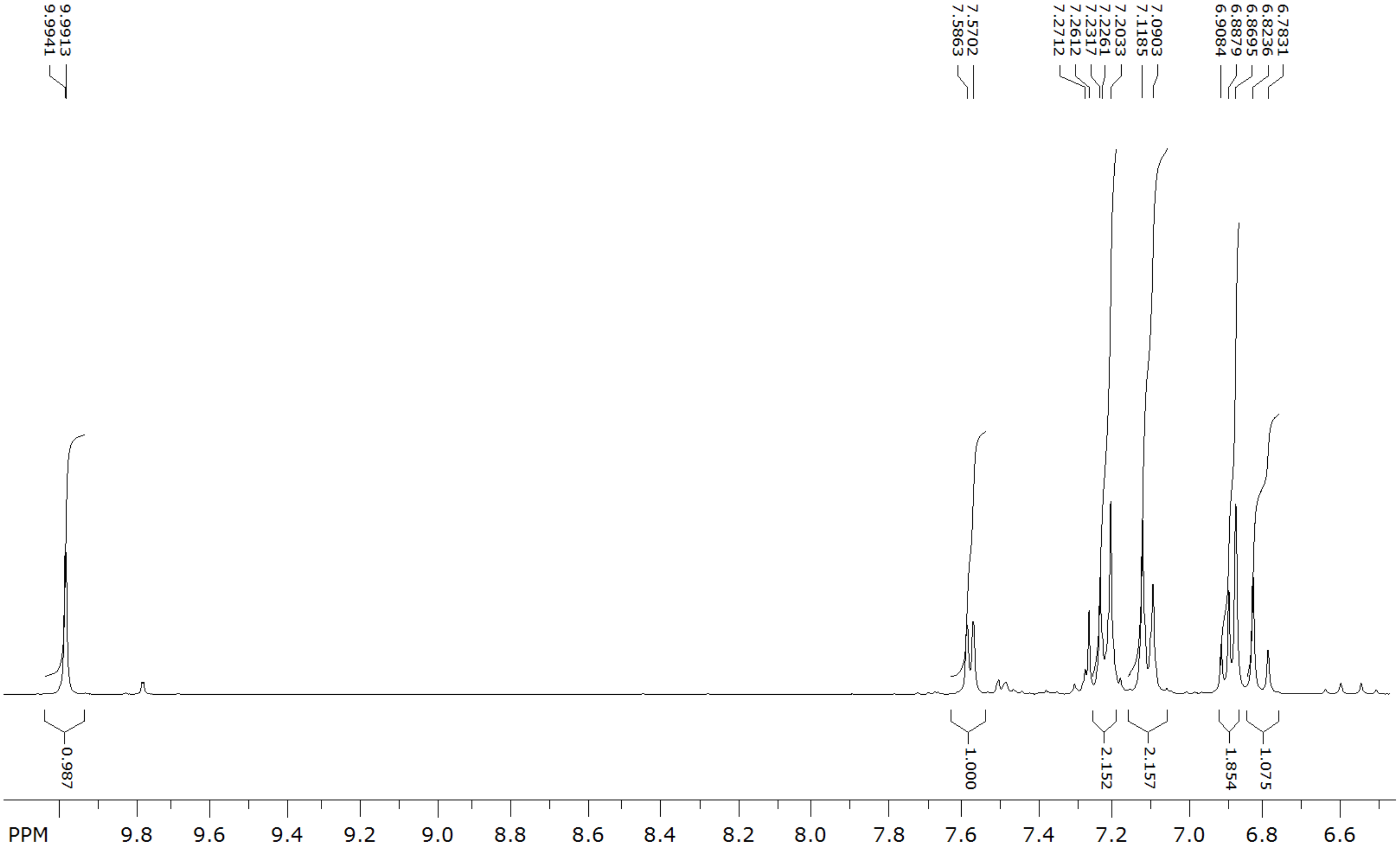

Figure S160. ^{1}H NMR ($CDCl_3$) spectrum of aromatic part of *cis*-**43**.

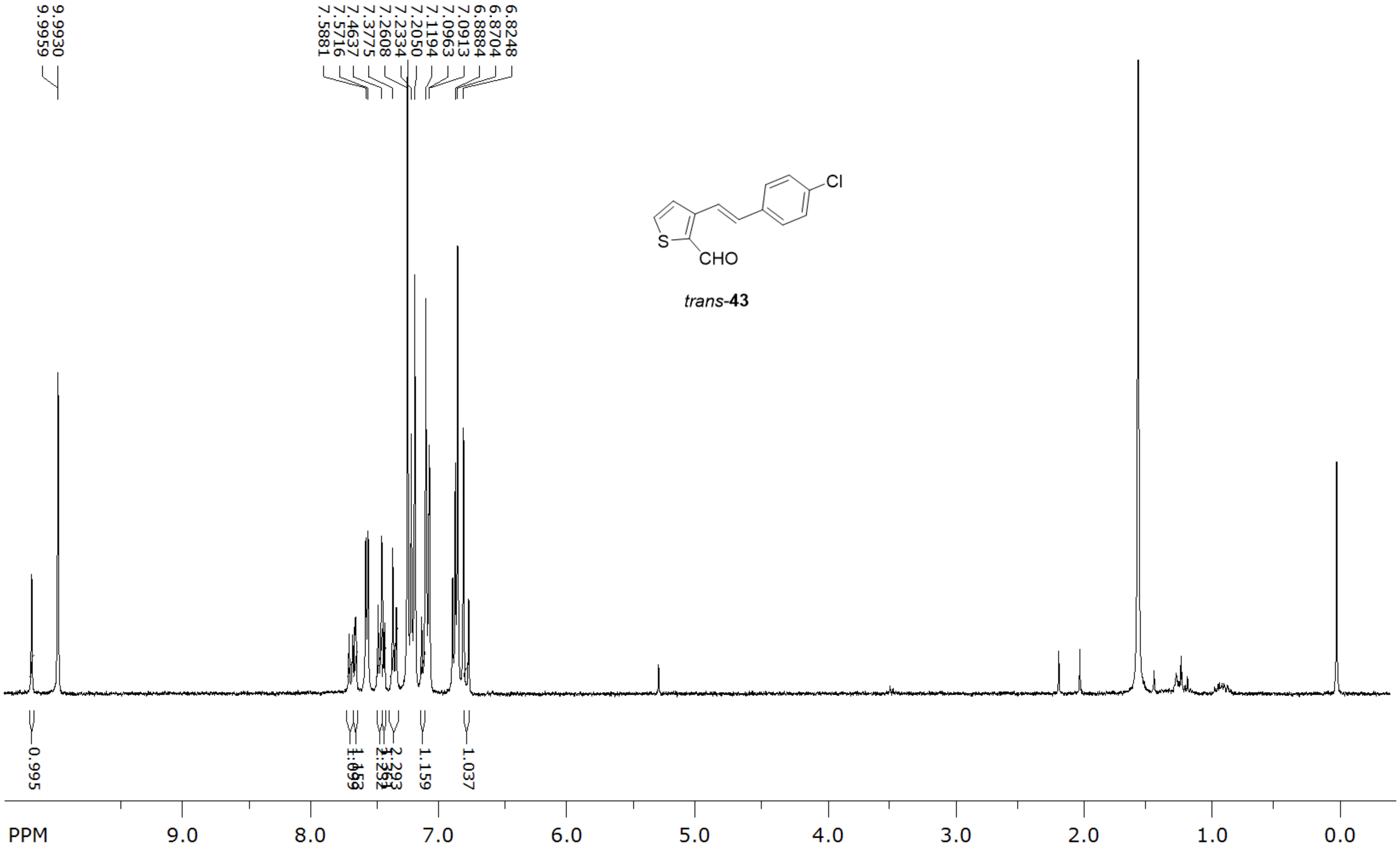

Figure S161. ^{1}H NMR ($CDCl_3$) spectrum of *trans*-**43** (with *cis*-**43**).

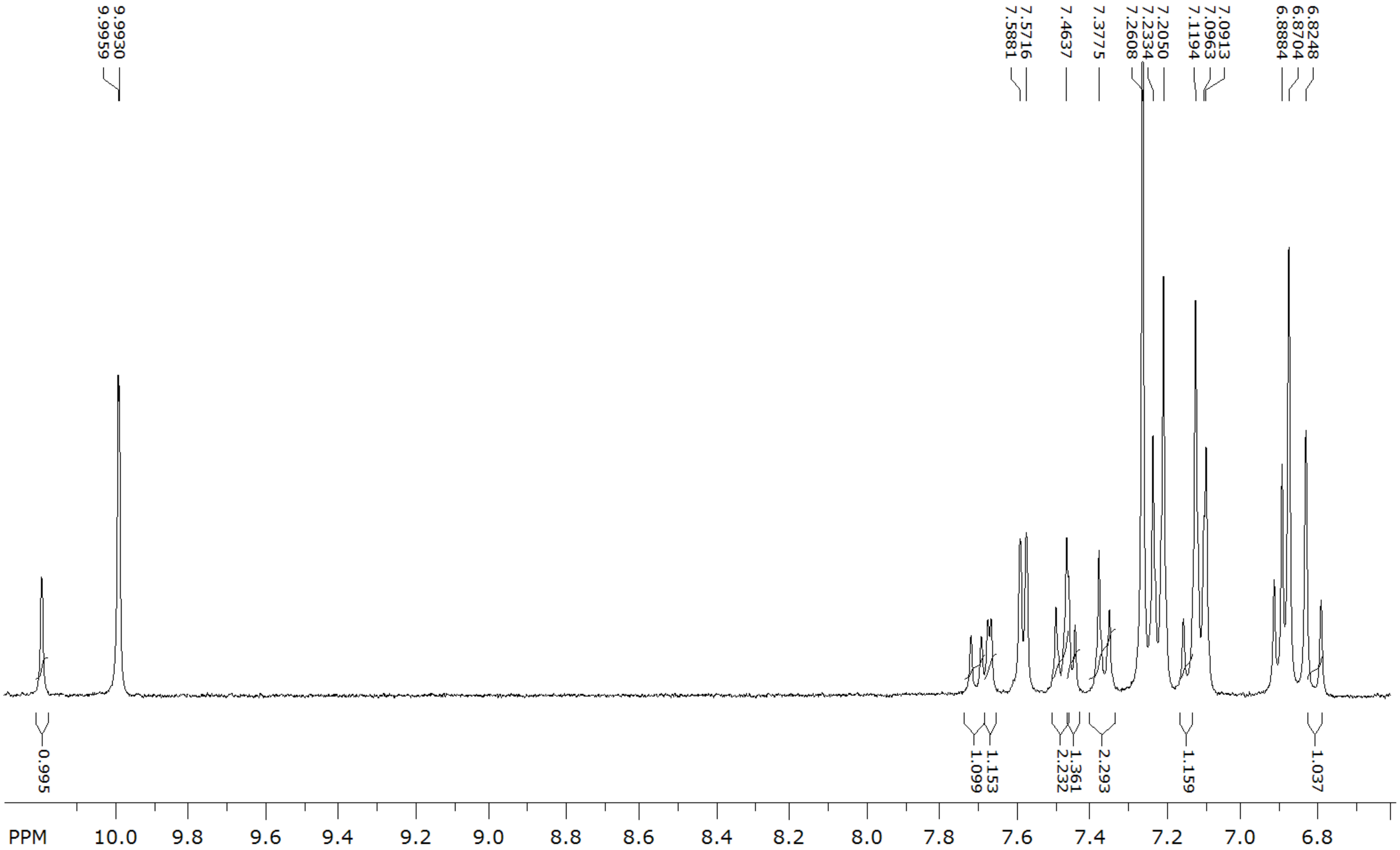

Figure S162. ^{1}H NMR ($CDCl_3$) spectrum of aromatic part of *trans*-**43** (with *cis*-**43**).

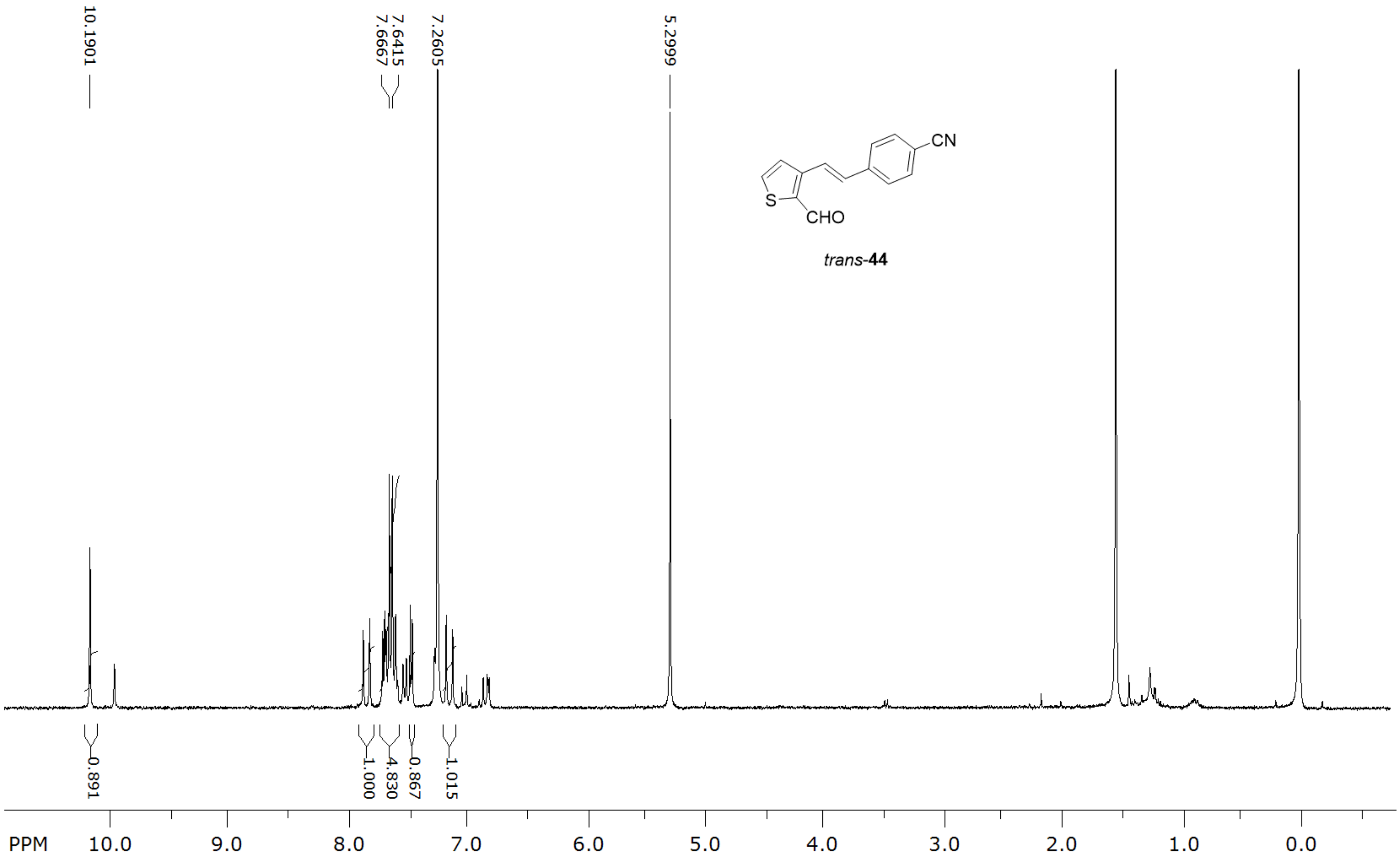

Figure S163. ^{1}H NMR ($CDCl_3$) spectrum of *trans*-**44**.

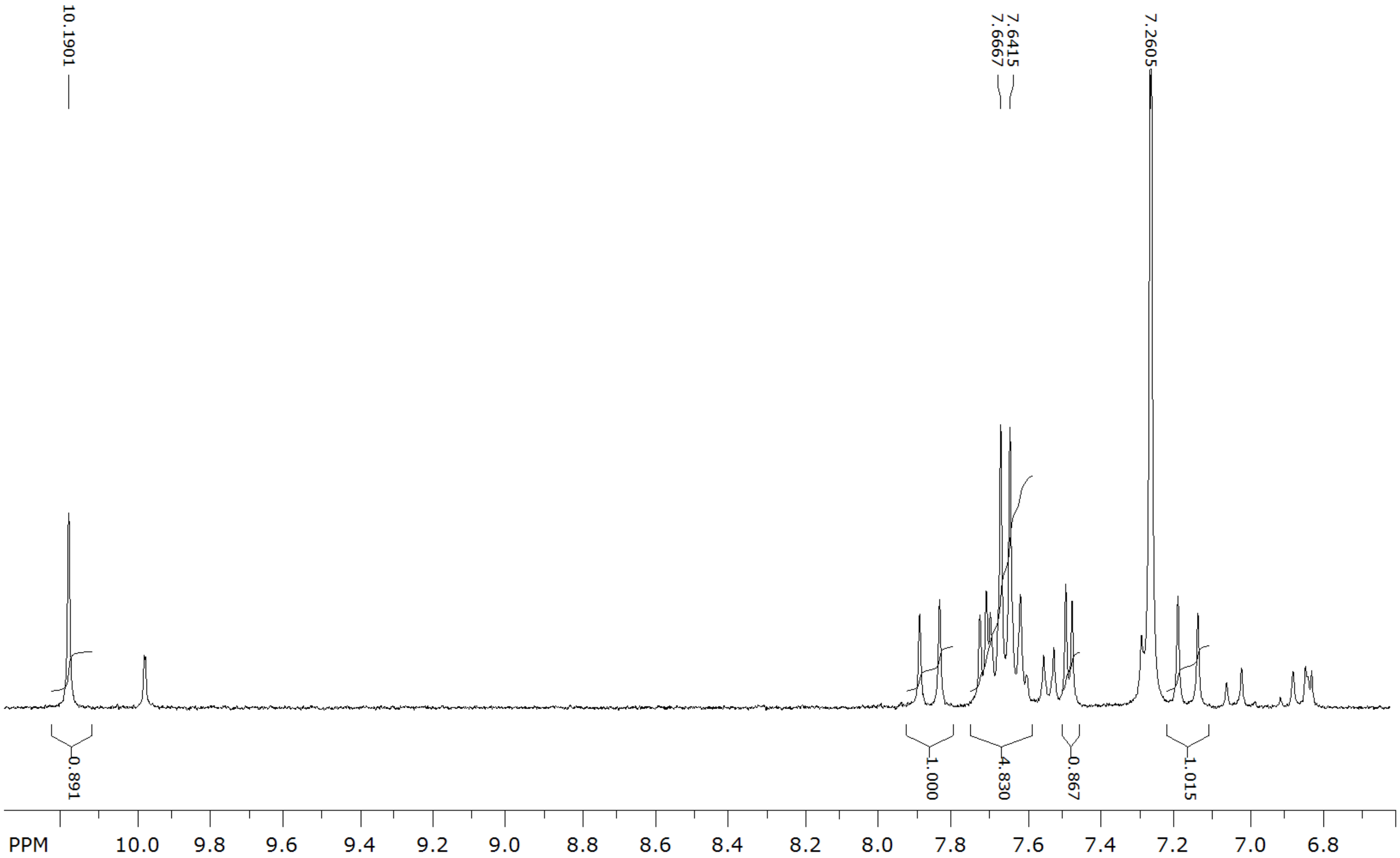

Figure S164. ^1H NMR ($CDCl_3$) spectrum of aromatic part of *trans*-**44**.

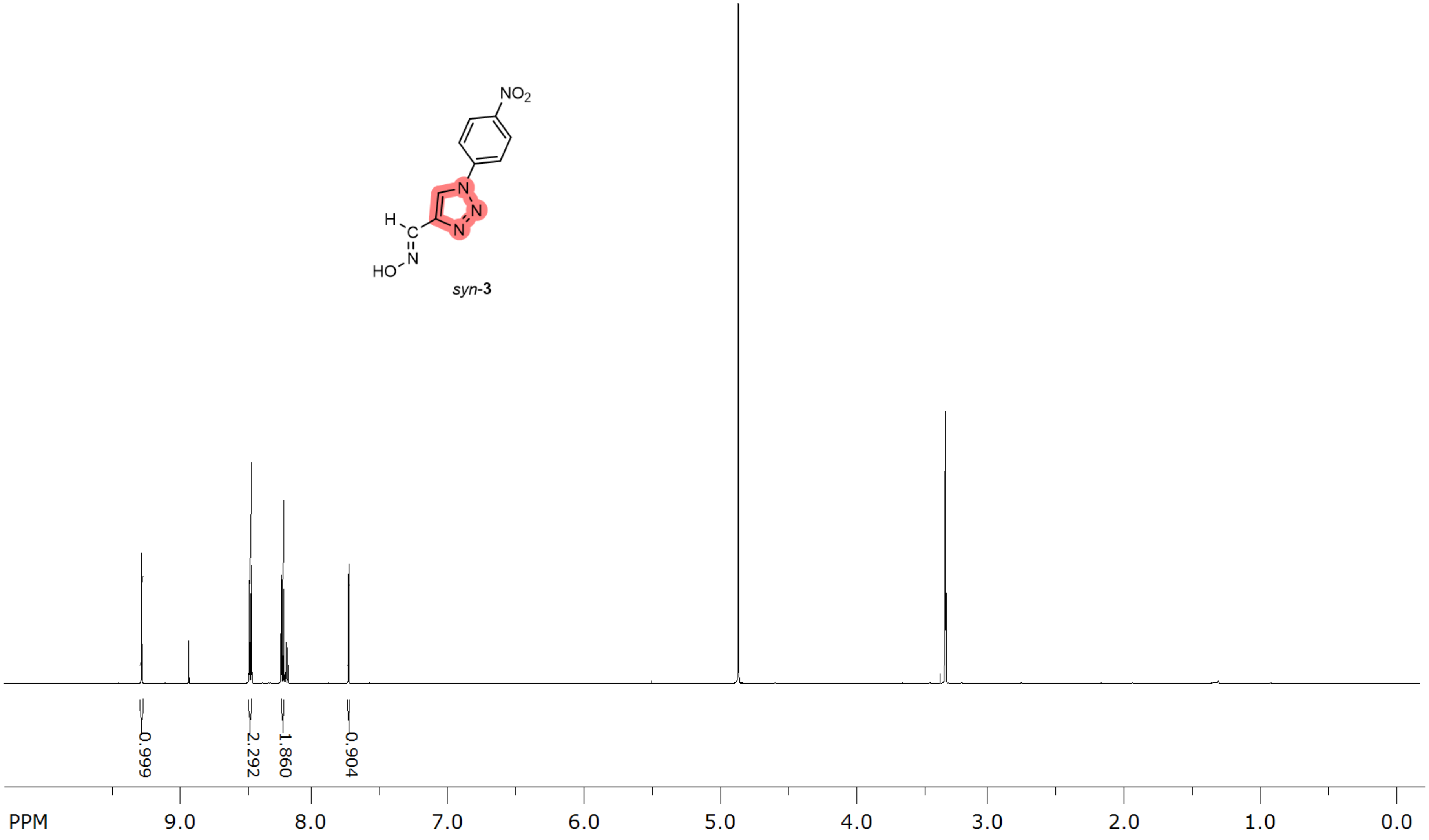

Figure S165. ^{1}H NMR ($CDCl_3$) spectrum of **3**.

Figure S166. ^1H NMR ($CDCl_3$) spectrum of aromatic part of *syn*-**3**.

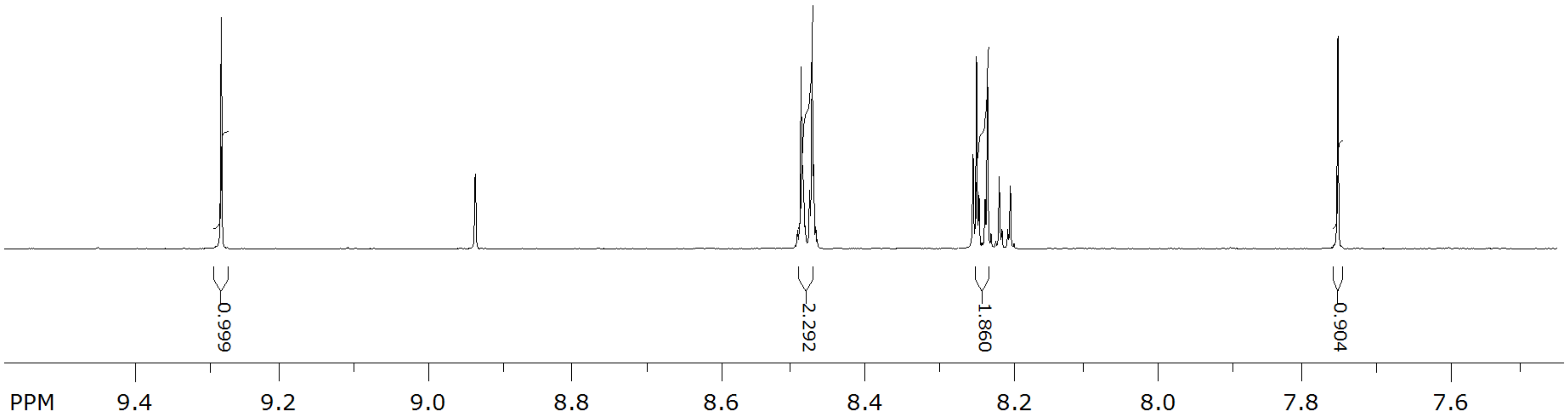

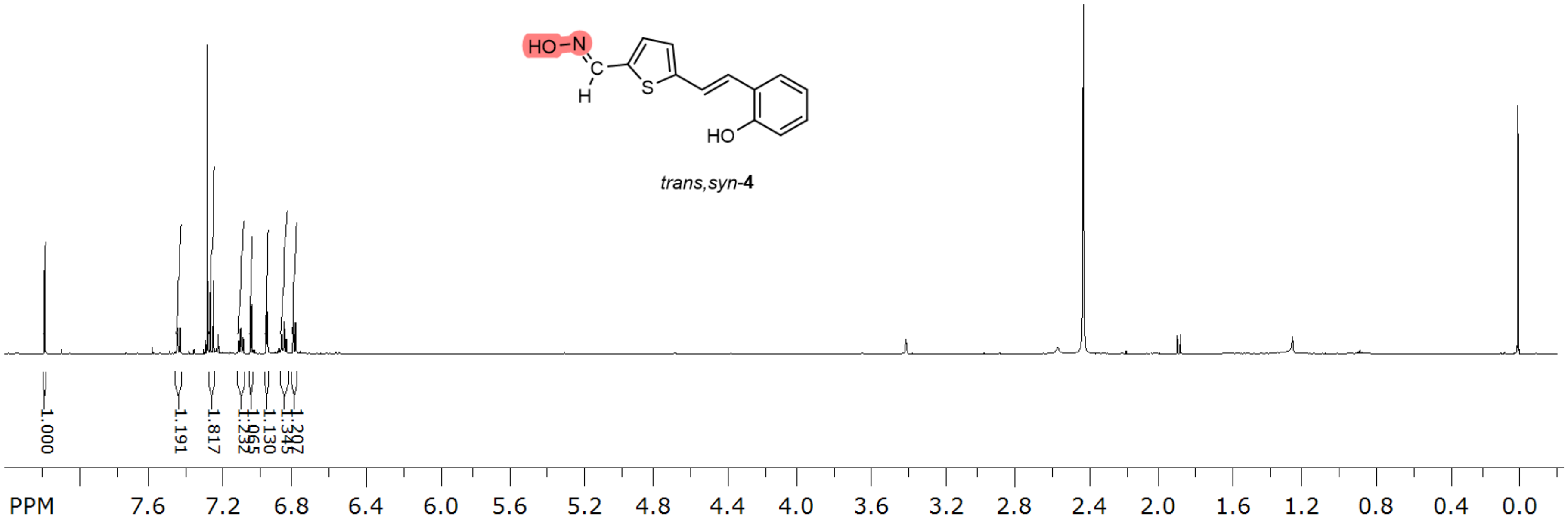

Figure S167. ^{1}H NMR ($CDCl_3$) spectrum of *trans,syn*-**4**.

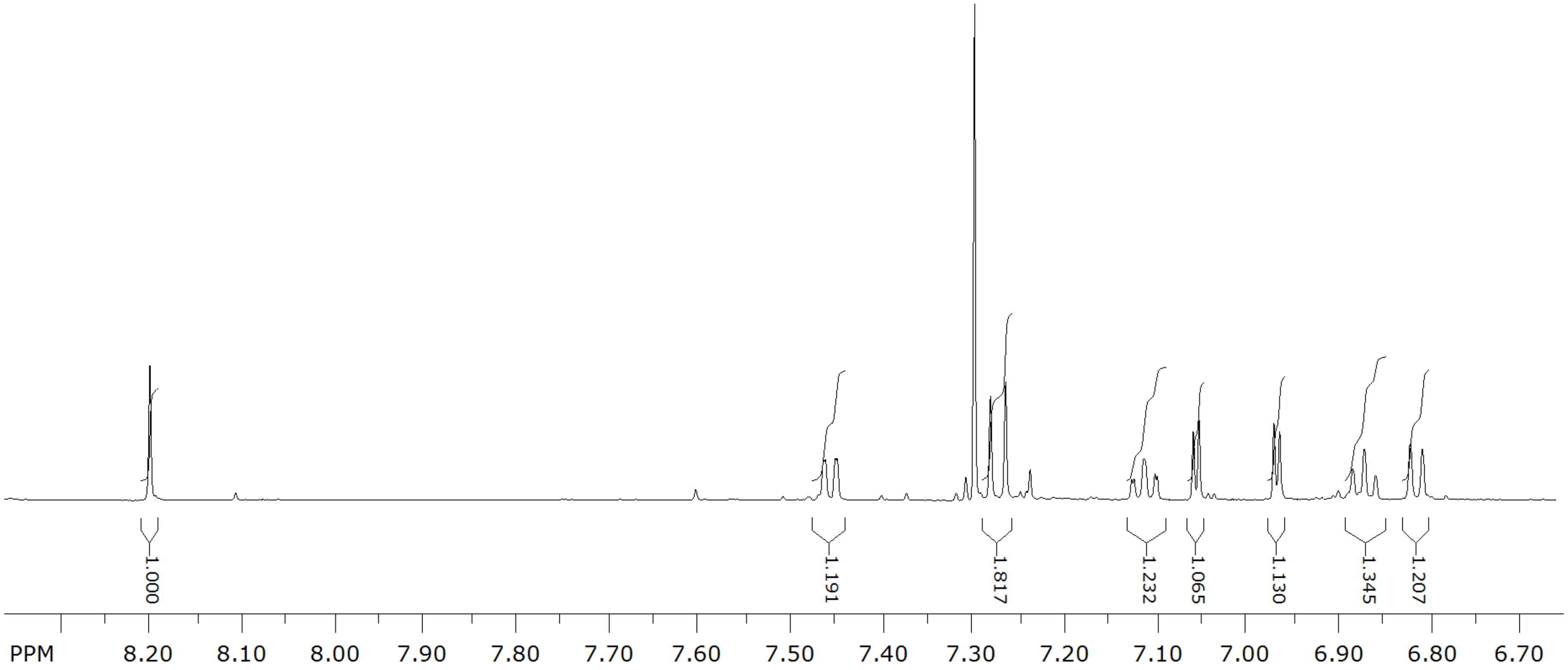

Figure S168. ^{1}H NMR ($CDCl_3$) spectrum of aromatic part of *trans,syn*-**4**.

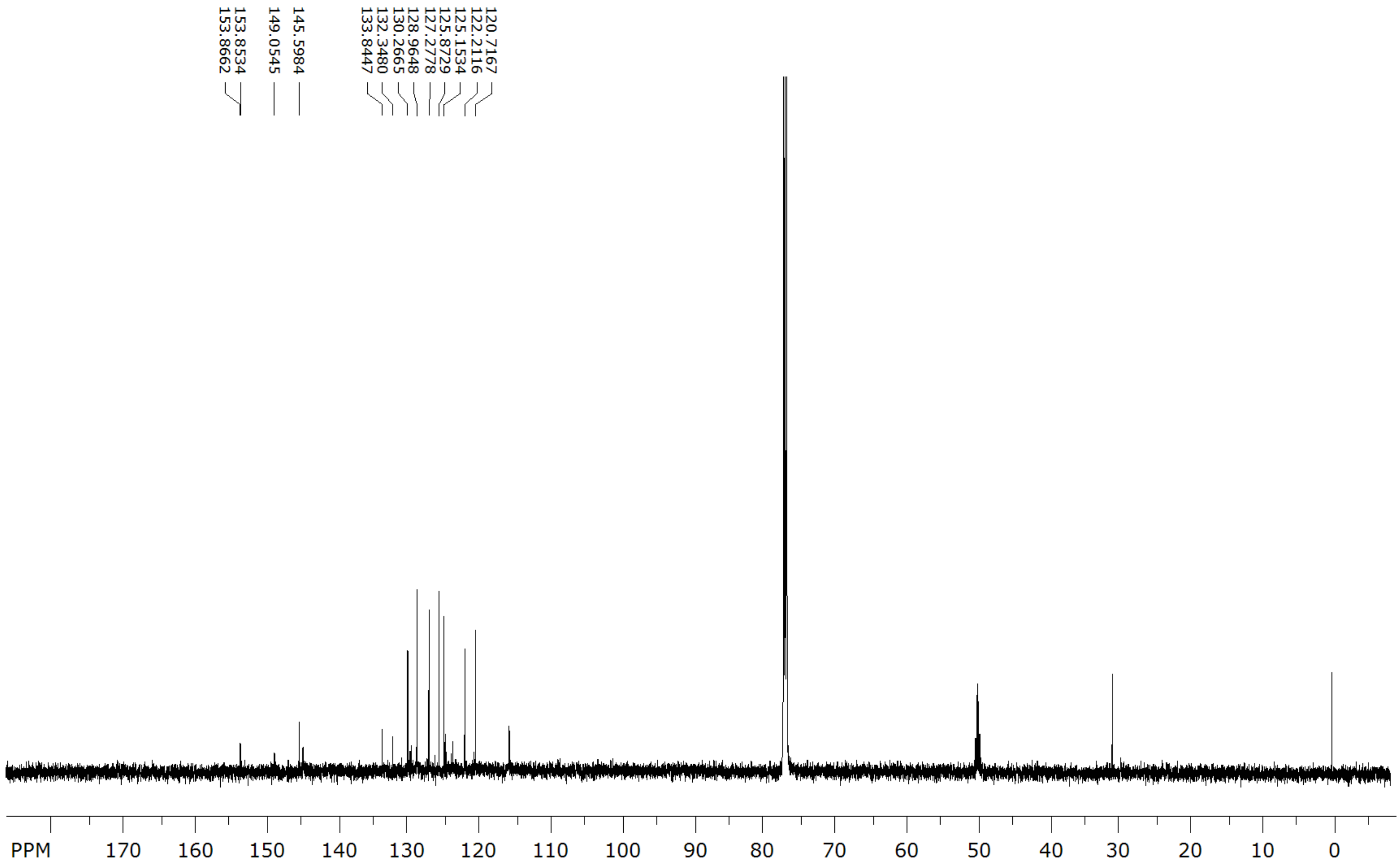

Figure S169. ^{13}C NMR ($CDCl_3$) spectrum of *trans,syn*-**4**.

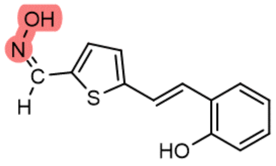

trans,anti-**4**

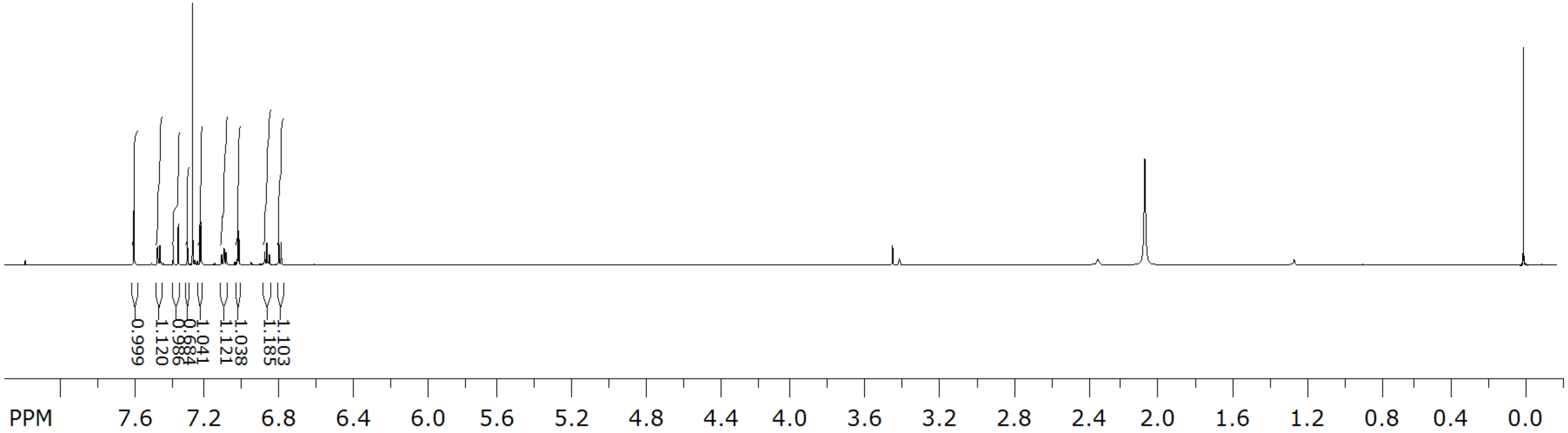

Figure S170. ^{1}H NMR ($CDCl_3$) spectrum of *trans,anti*-**4**.

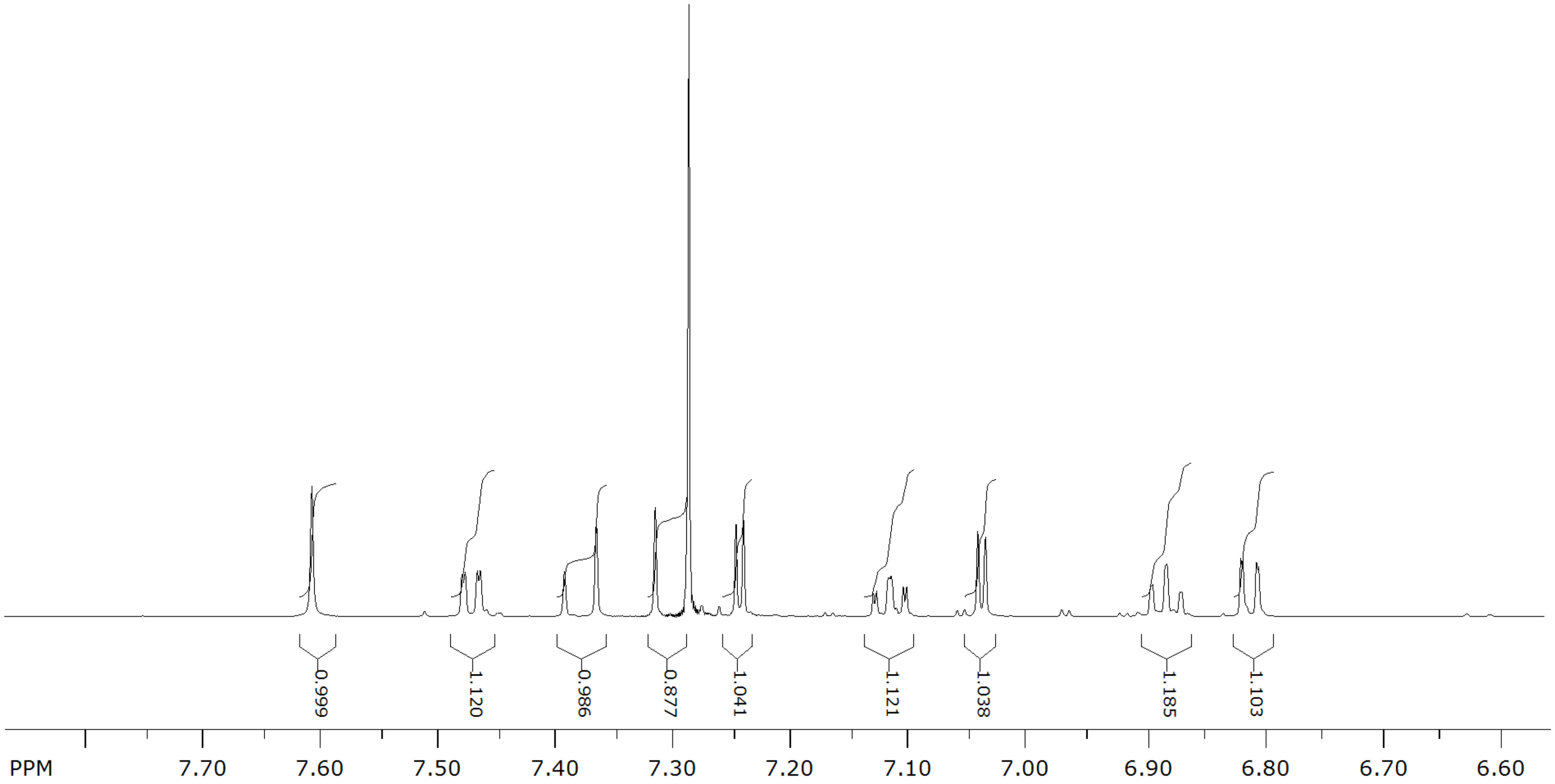

Figure S171. ^{1}H NMR ($CDCl_3$) spectrum of aromatic part of *trans,anti*-**4**.

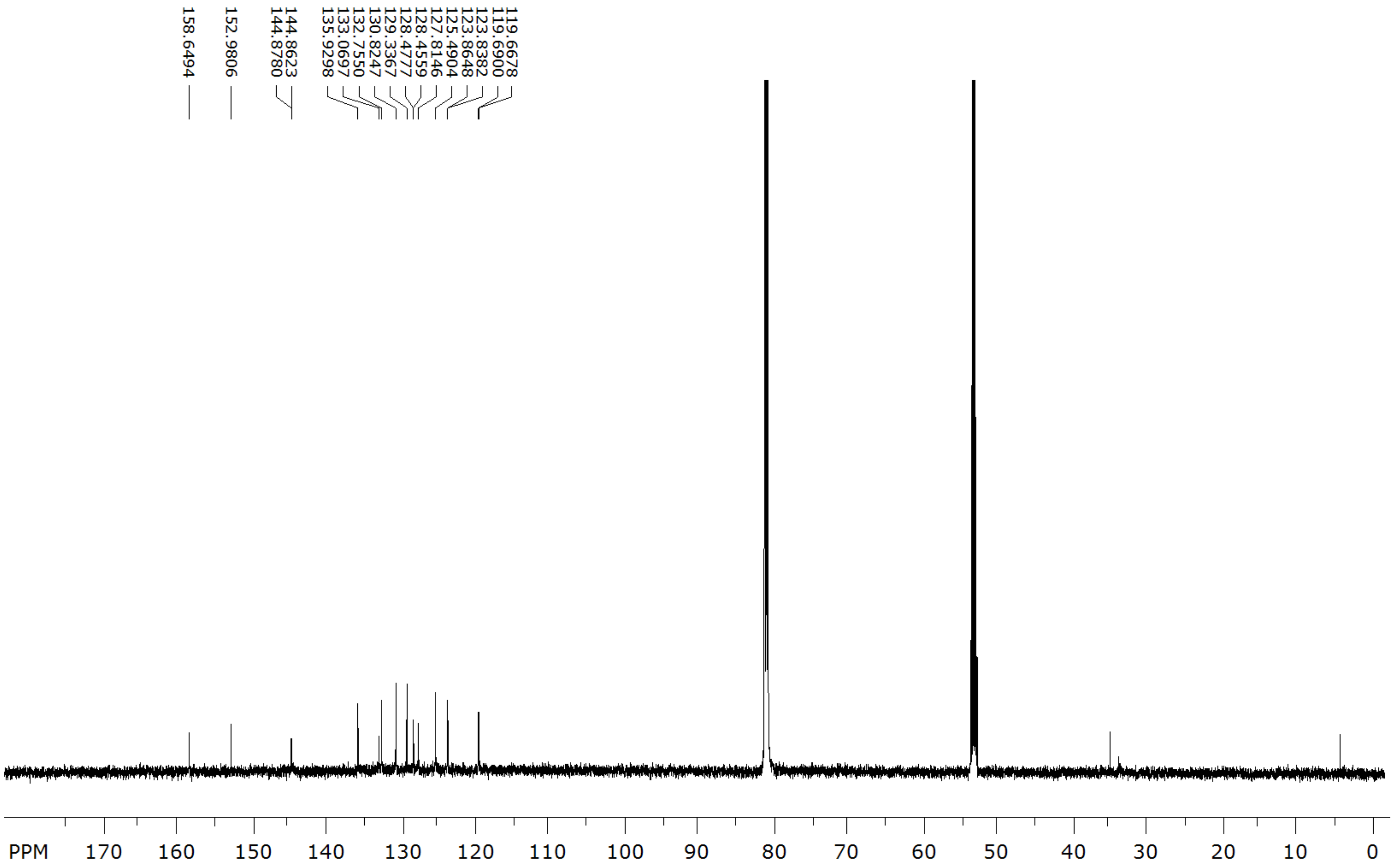

Figure S172. ^{13}C NMR ($CDCl_3$+CD_3OD) spectrum of *trans,anti*-**4**.

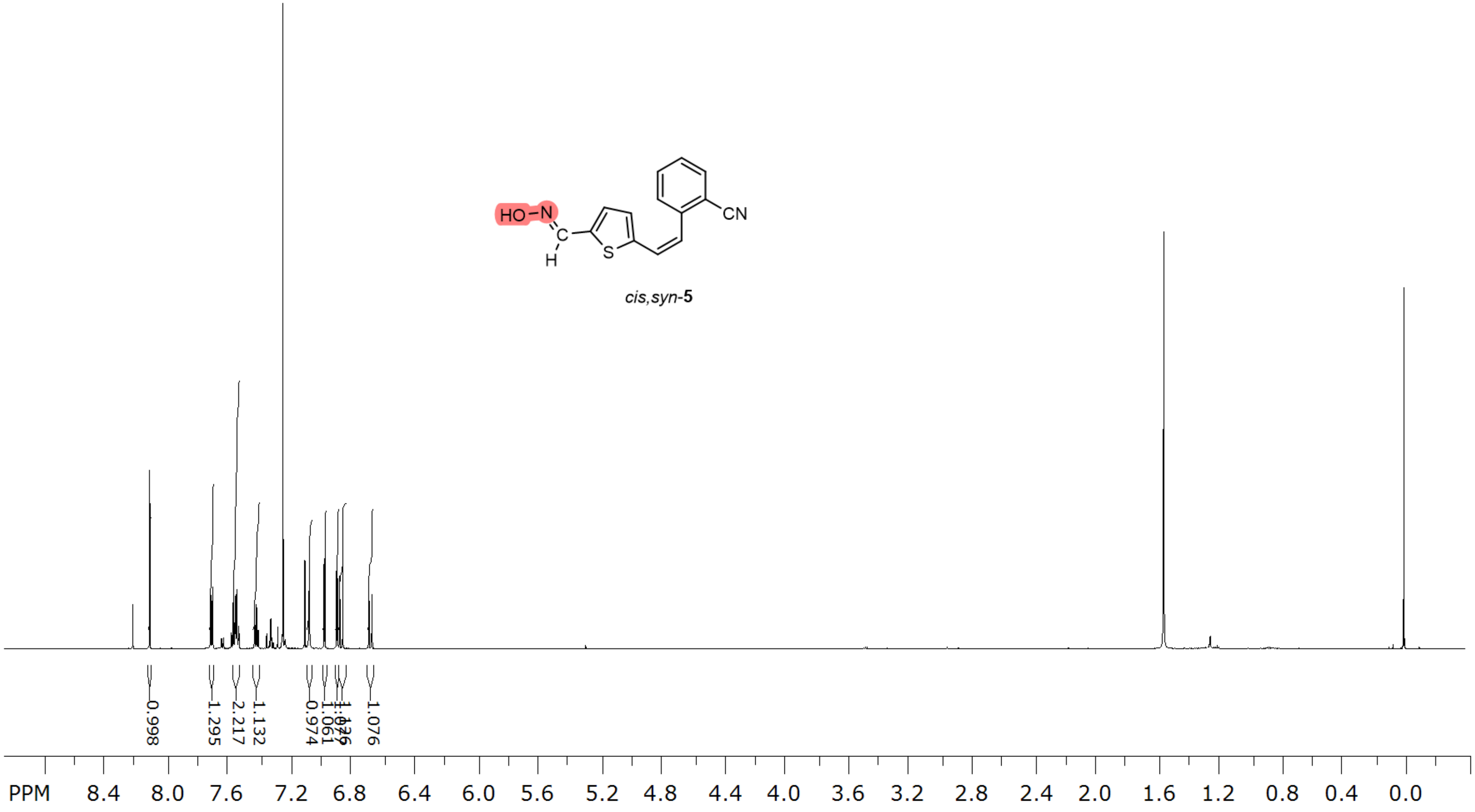

Figure S173. ^{1}H NMR ($CDCl_3$) spectrum of *cis,syn*-**5**.

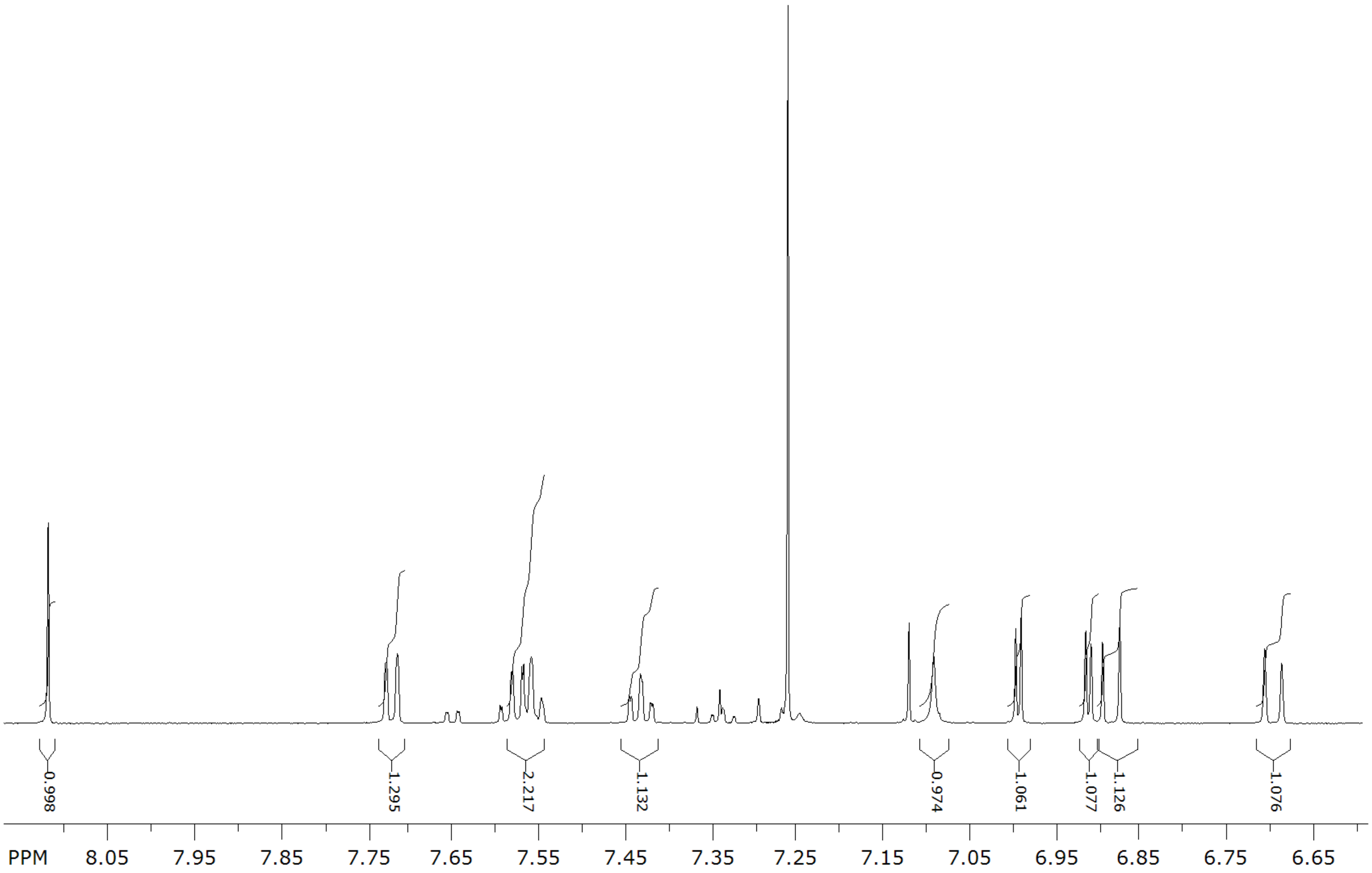

Figure S174. ^{1}H NMR ($CDCl_3$) spectrum of aromatic part of *cis,syn*-**5**.

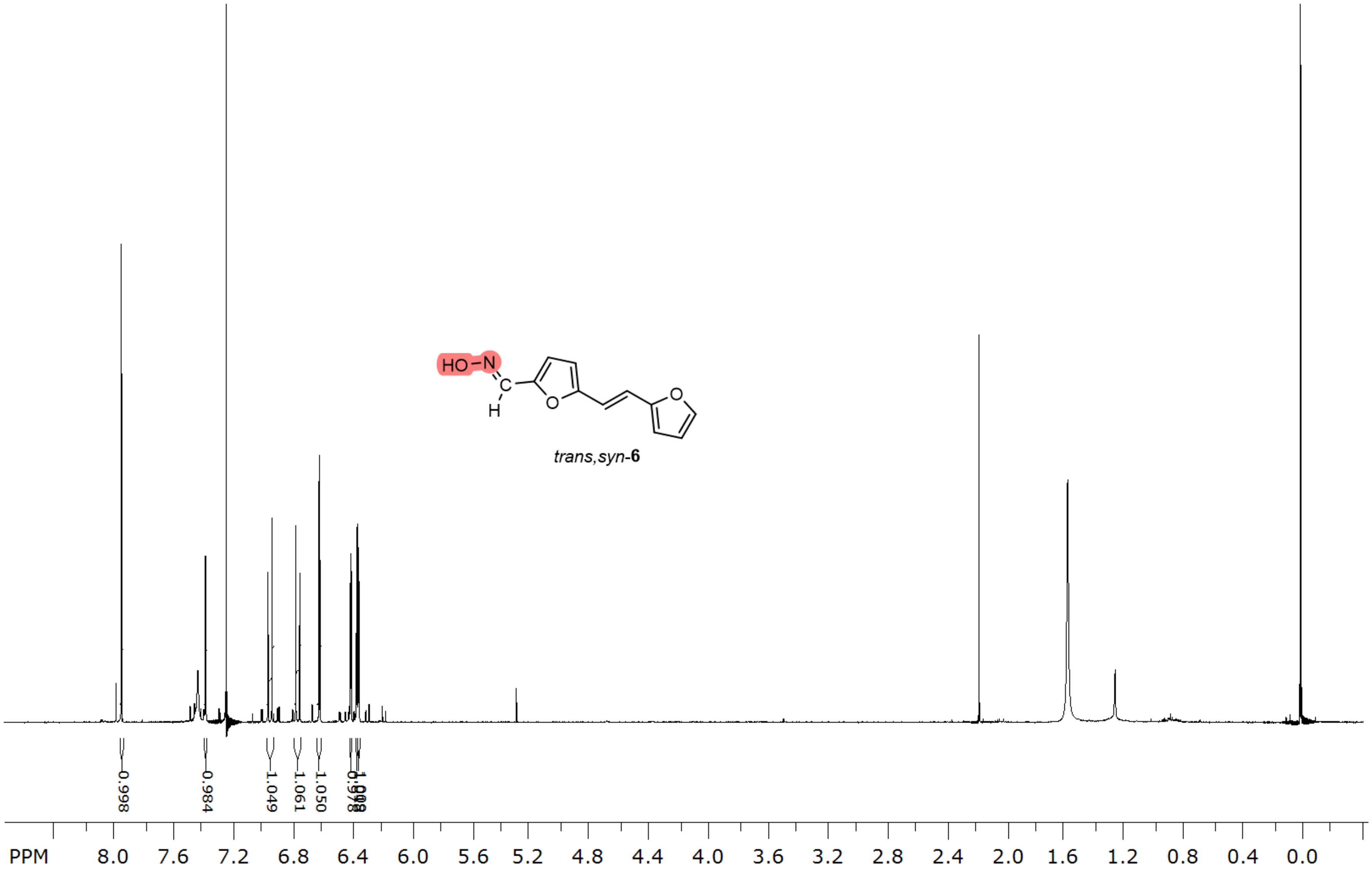

Figure S175. ^1H NMR ($CDCl_3$) spectrum of *trans,syn*-**6**.

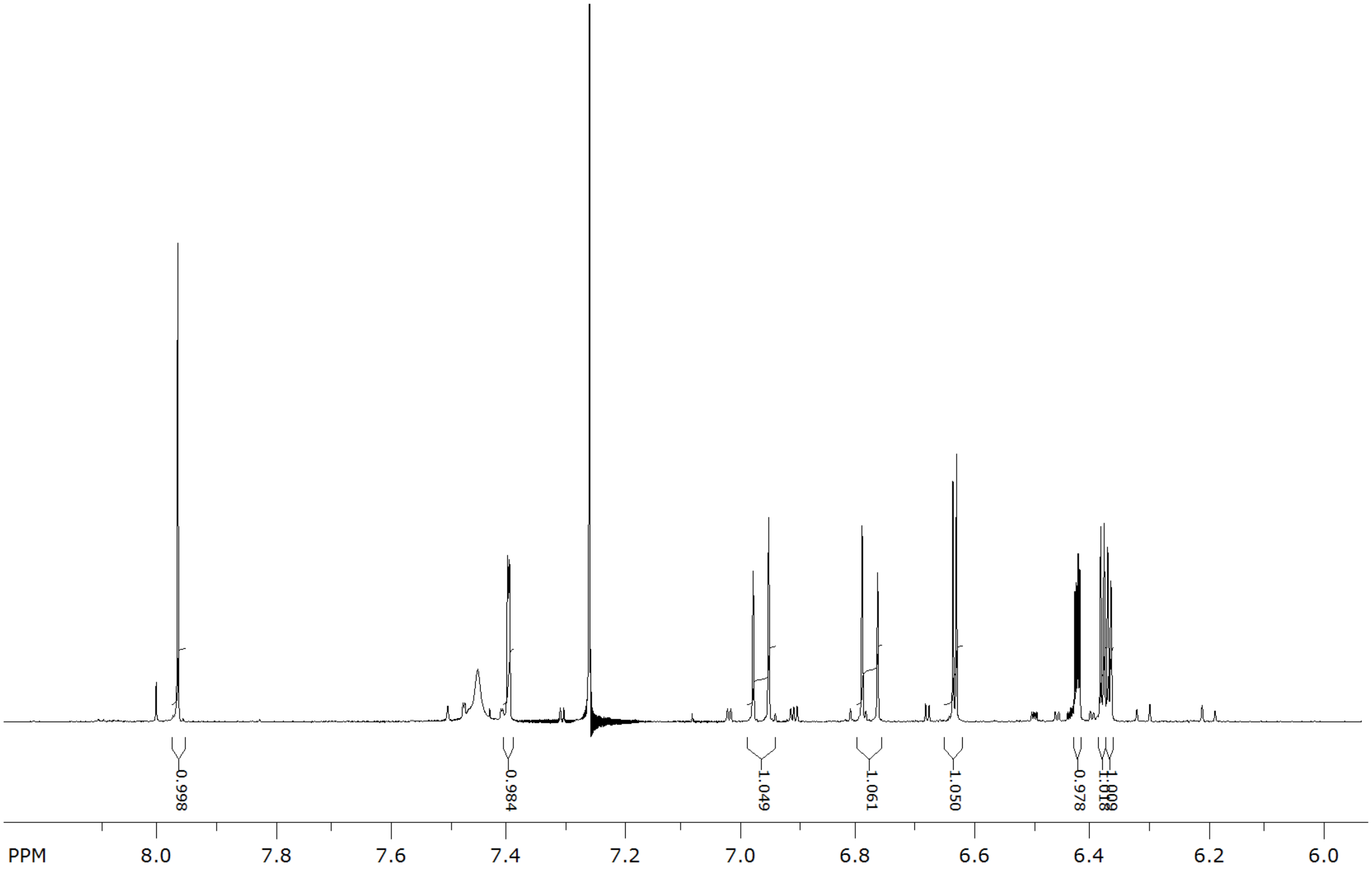

Figure S176. ^{1}H NMR ($CDCl_3$) spectrum of aromatic part of *trans,syn*-**6**.

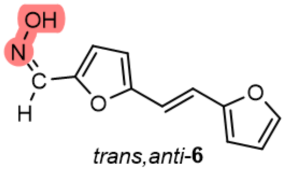

trans,anti-**6**

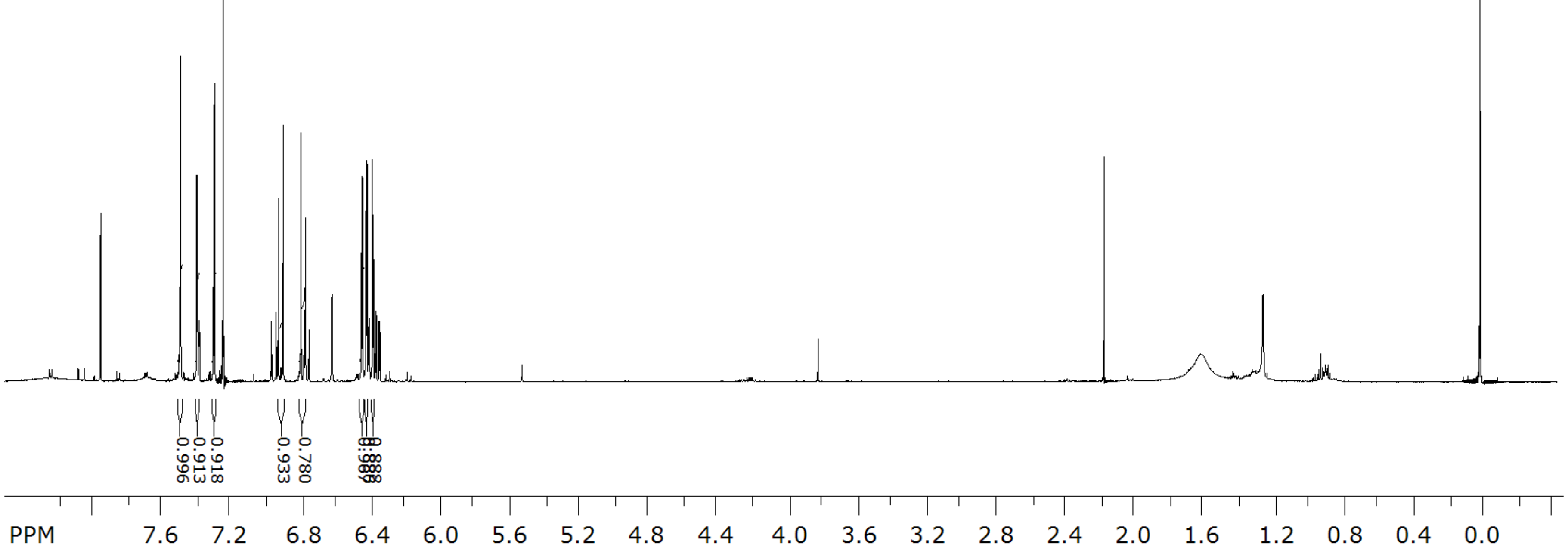

Figure S177. ^{1}H NMR ($CDCl_3$) spectrum of *trans,anti*-**6**.

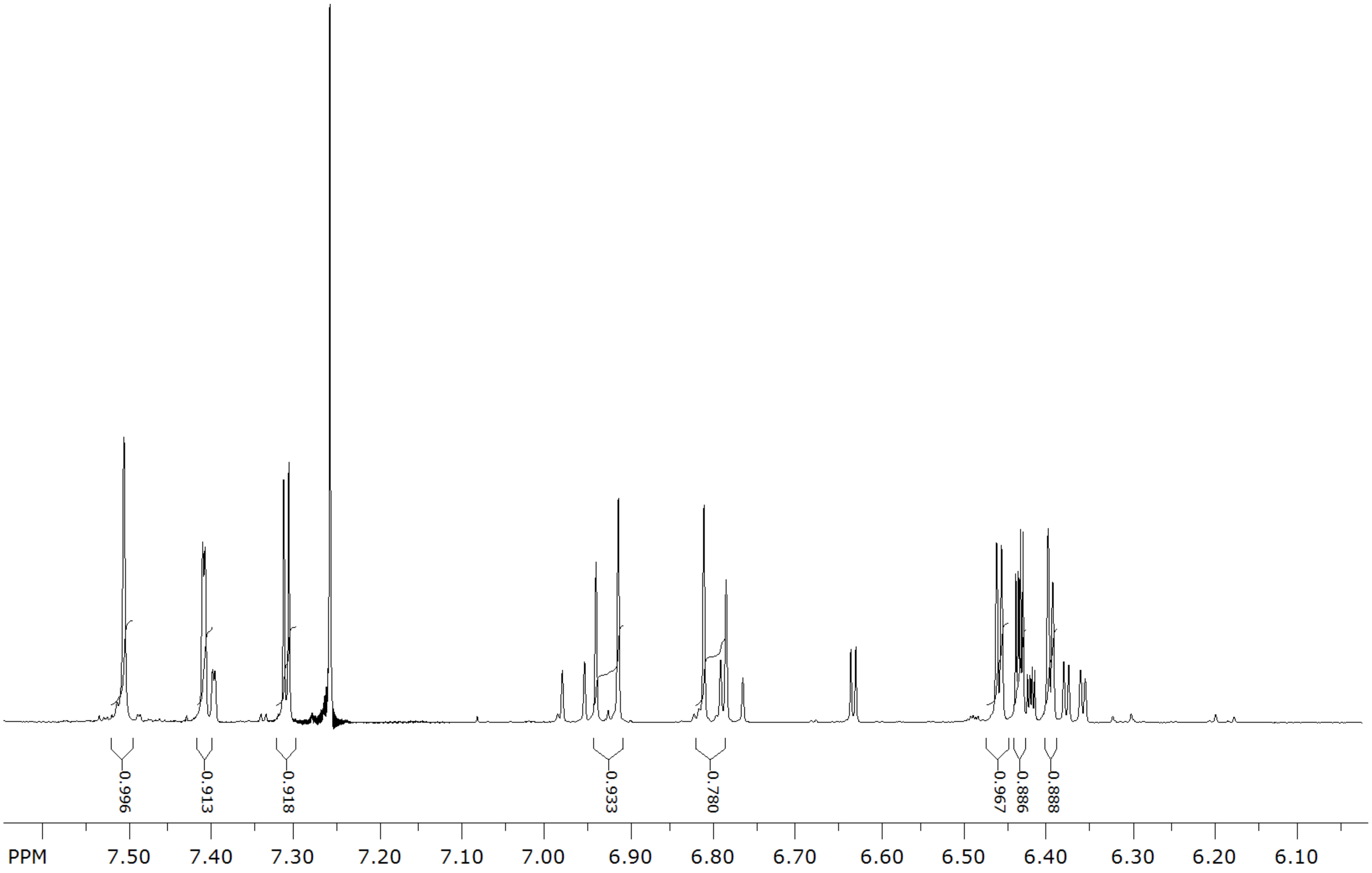

Figure S178. ^{1}H NMR ($CDCl_3$) spectrum of aromatic part of *trans,anti*-**6** (with *trans,syn*-**6**).

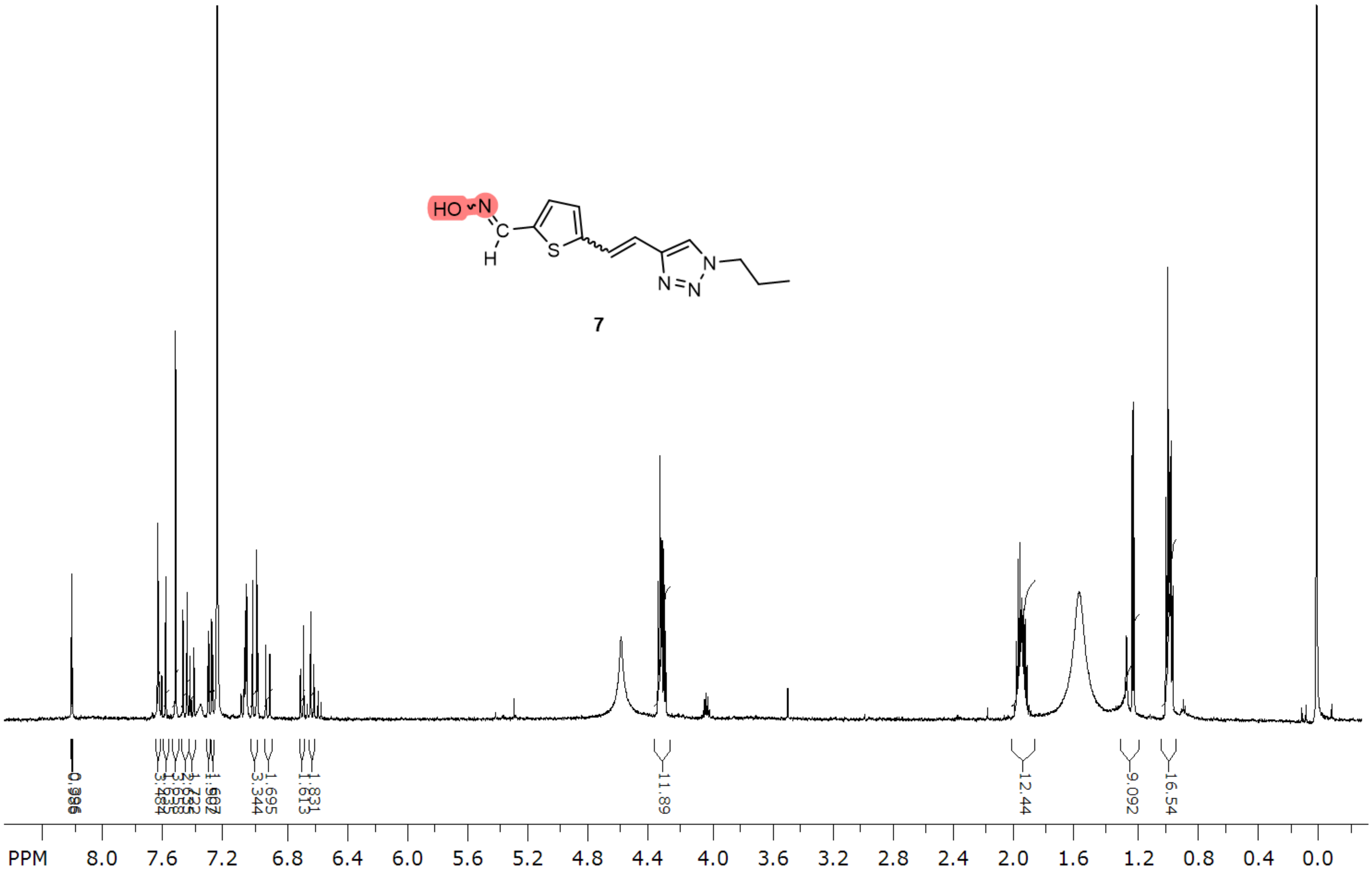

Figure S179. ^{1}H NMR ($CDCl_3$) spectrum of *trans*-**7**.

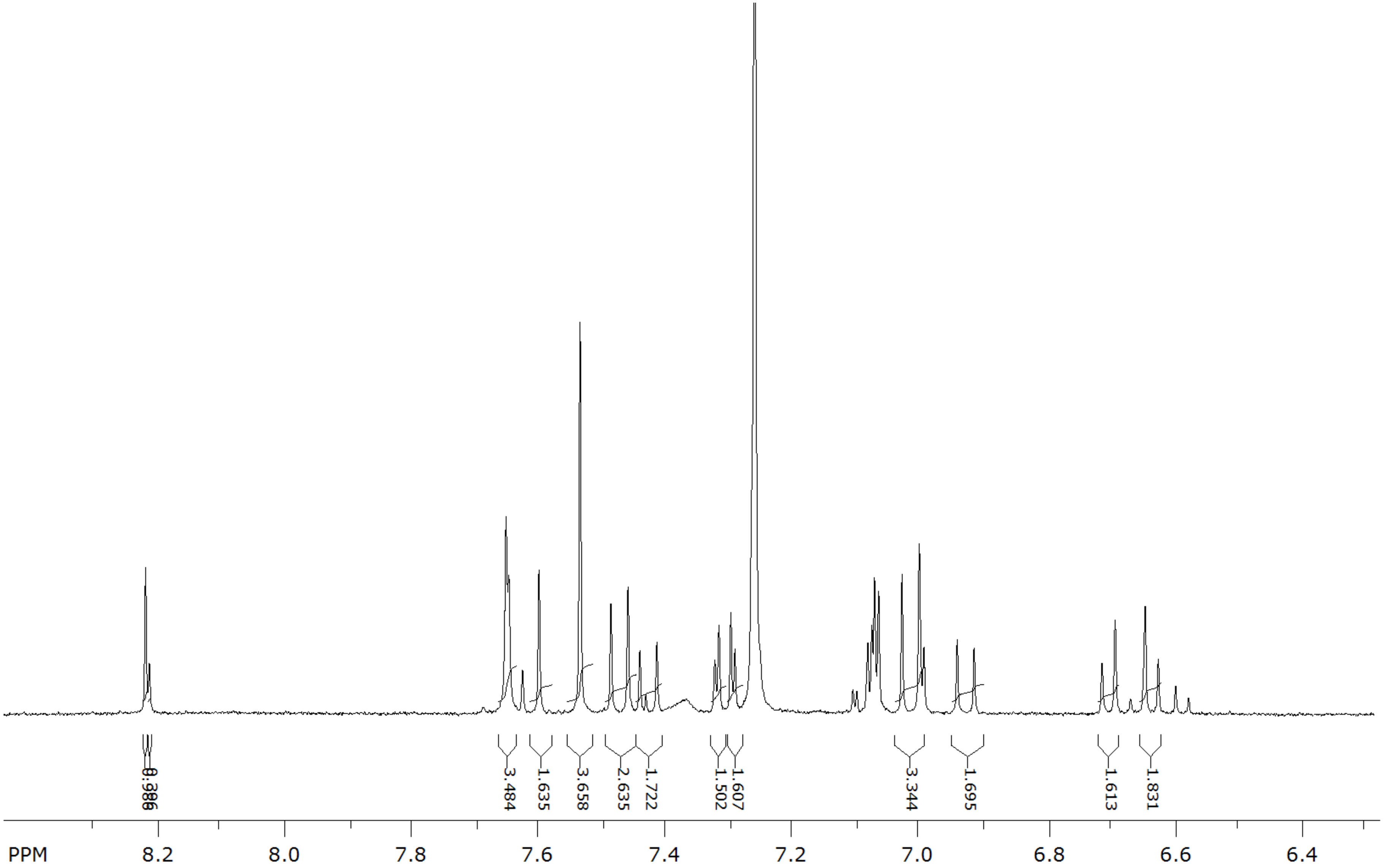

Figure S180. ^{1}H NMR ($CDCl_3$) spectrum of aromatic part of *trans*-**7**.

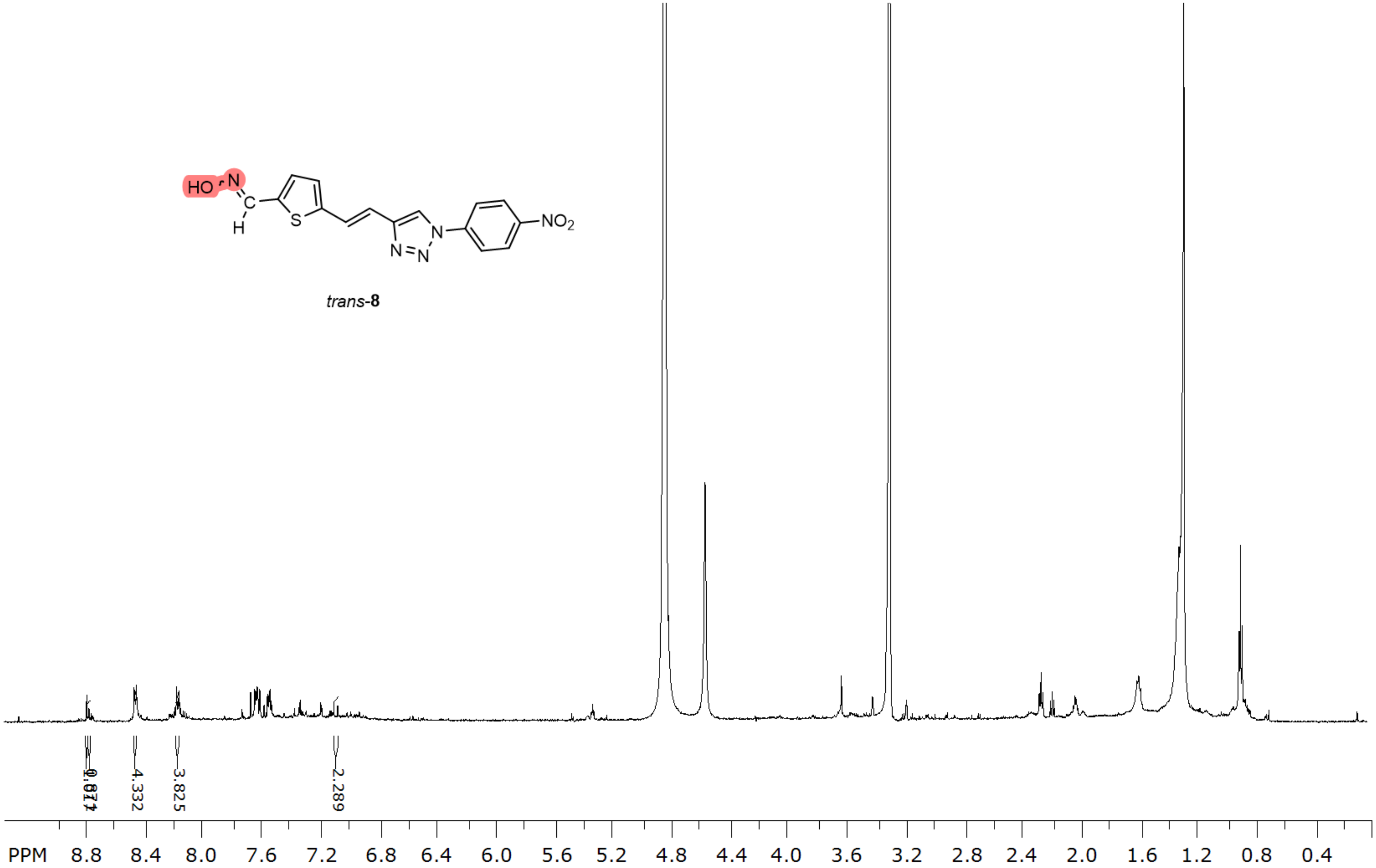

Figure S181. ^{1}H NMR (CD_3OD) spectrum of *trans*-**8**.

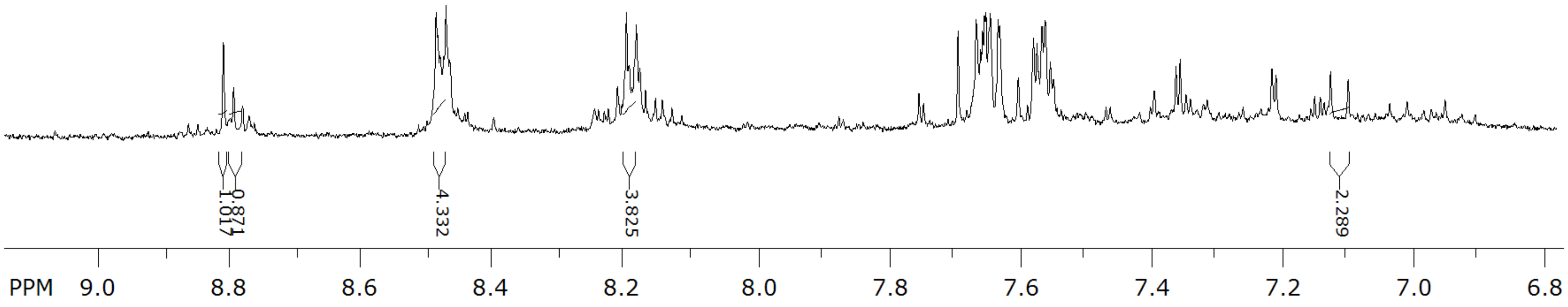

Figure S182. ^1H NMR (CD_3OD) spectrum of aromatic part of *trans*-**8**.

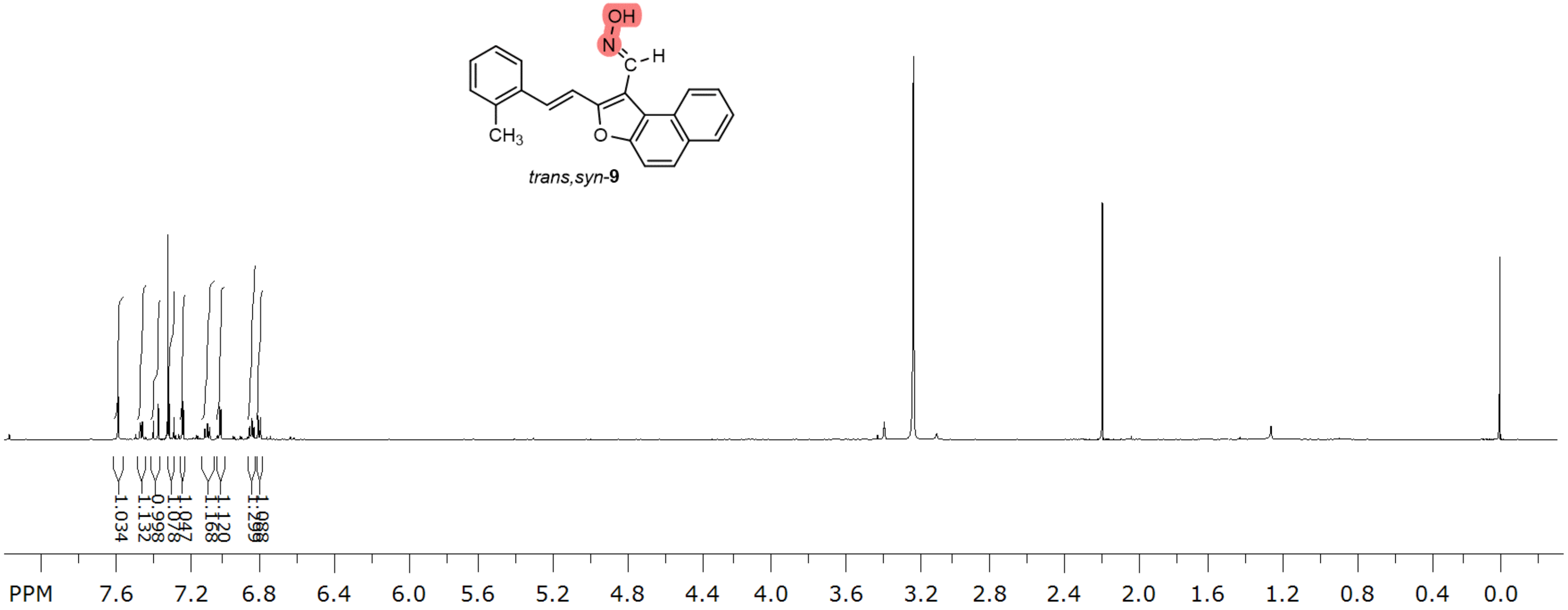

Figure S183. ^{1}H NMR (CD_3OD) spectrum of *trans,syn*-**9**.

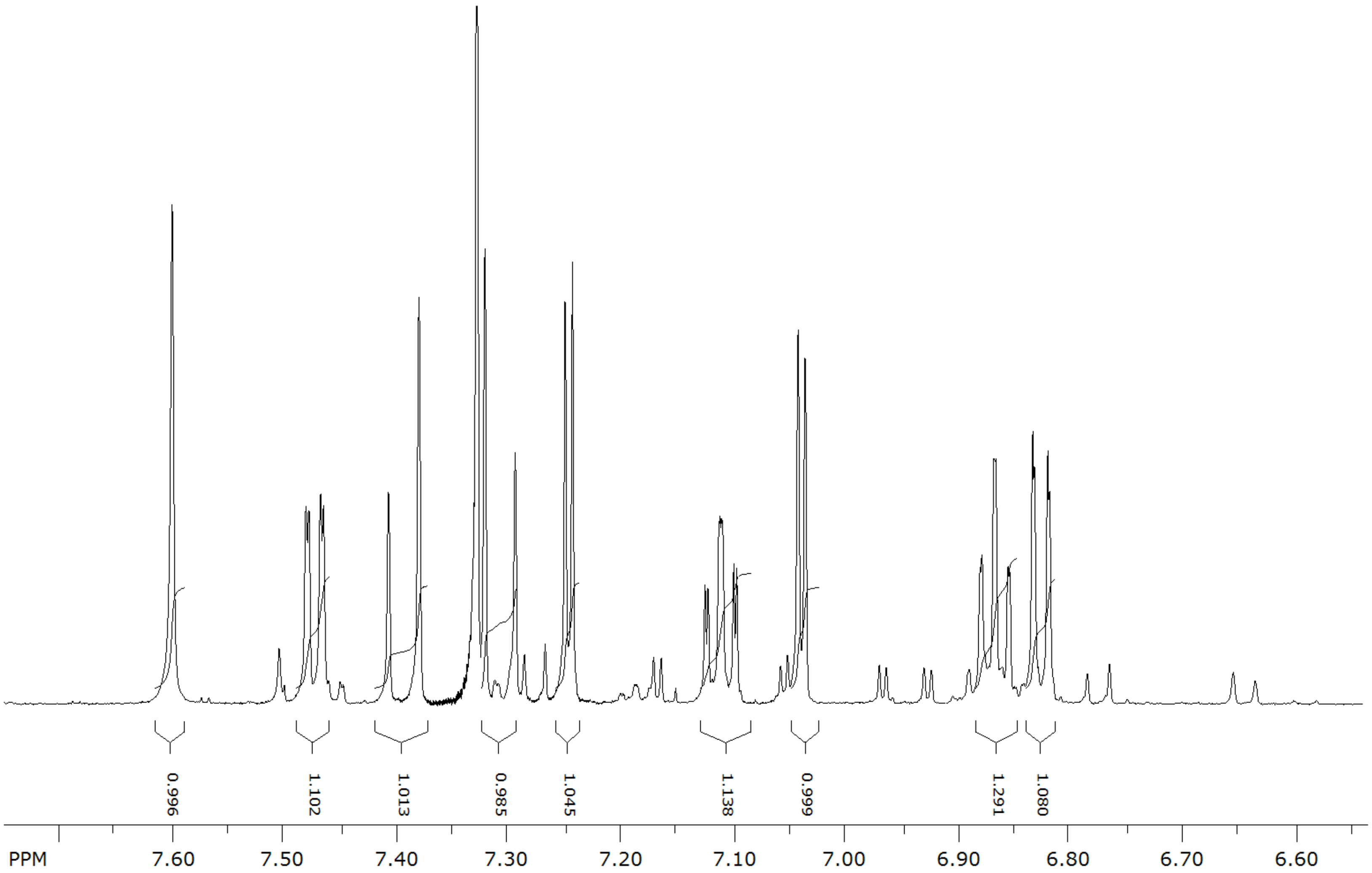

Figure S184. ^{1}H NMR (CD_3OD) spectrum of aromatic part of *trans,syn*-**9**.

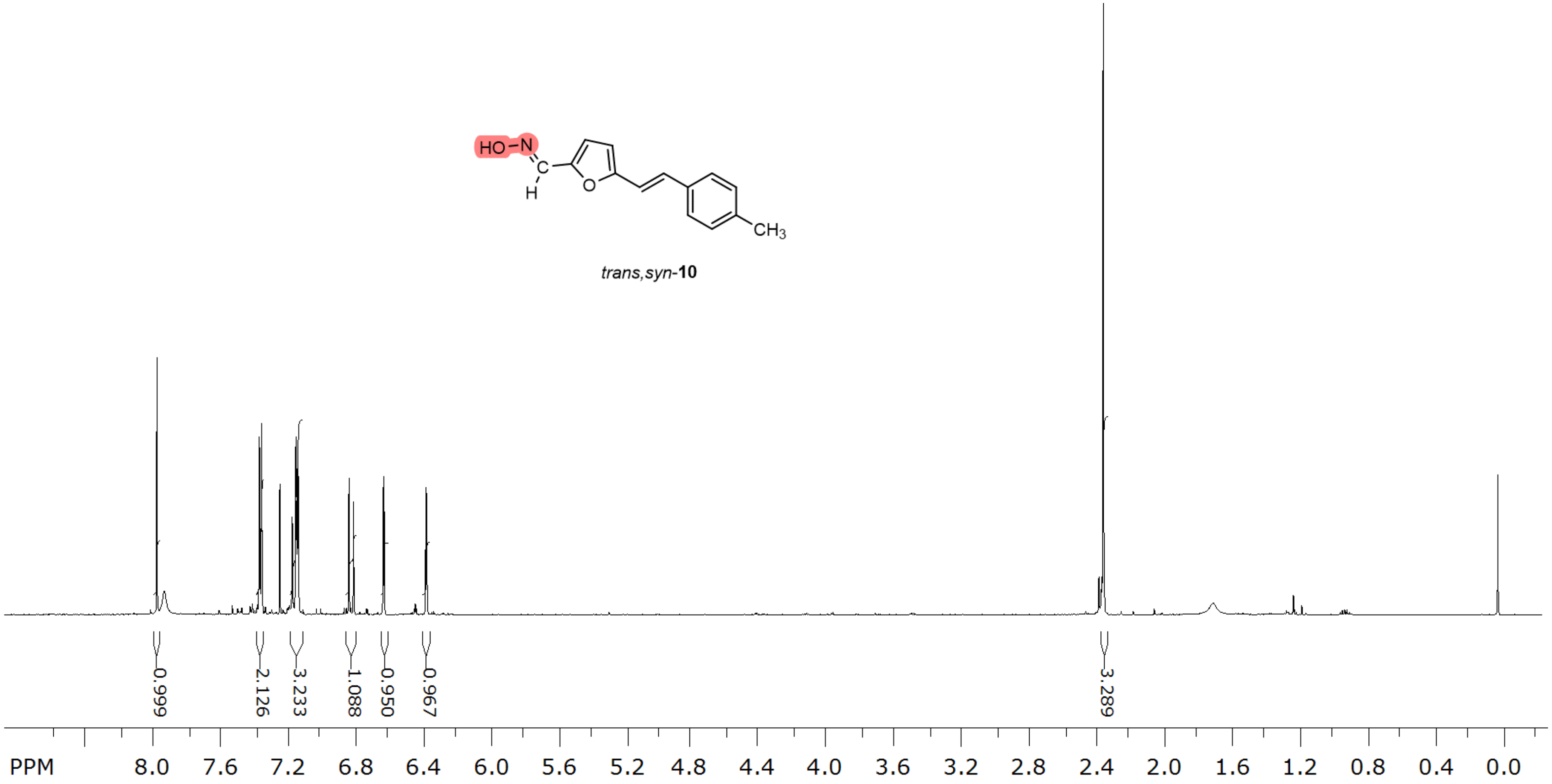

Figure S185. ^{1}H NMR ($CDCl_3$) spectrum of *trans,syn*-**10**.

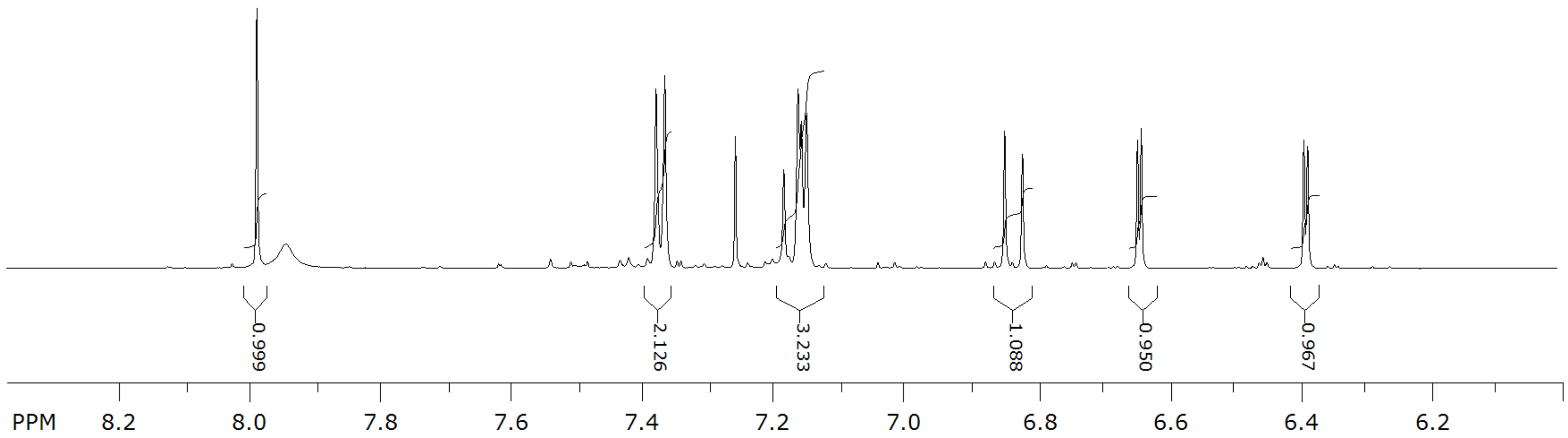

Figure S186. ^{1}H NMR ($CDCl_3$) spectrum of aromatic part of *trans,syn*-**10**.

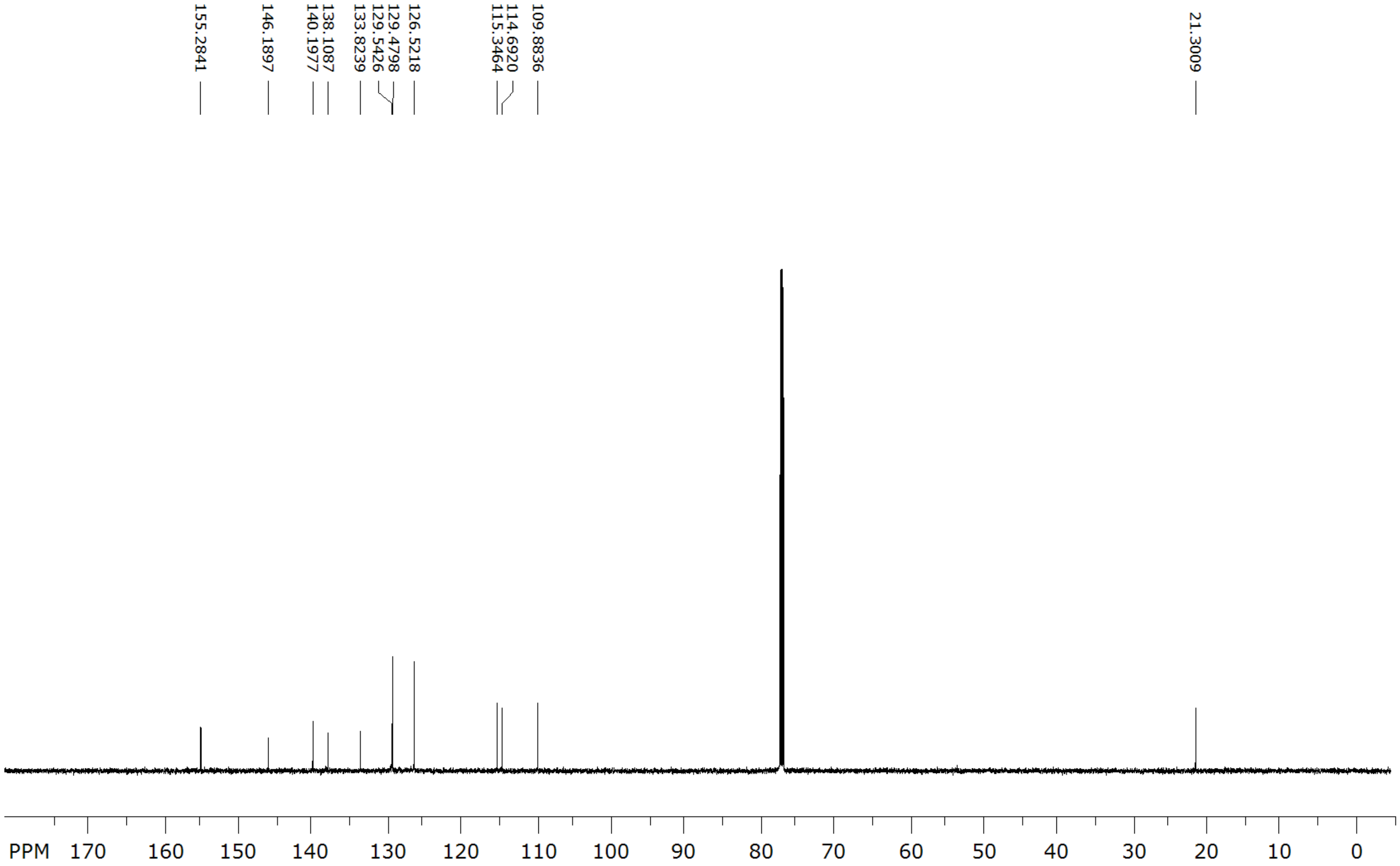

Figure S187. ^{13}C NMR ($CDCl_3$) spectrum of *trans,syn*-**10**.

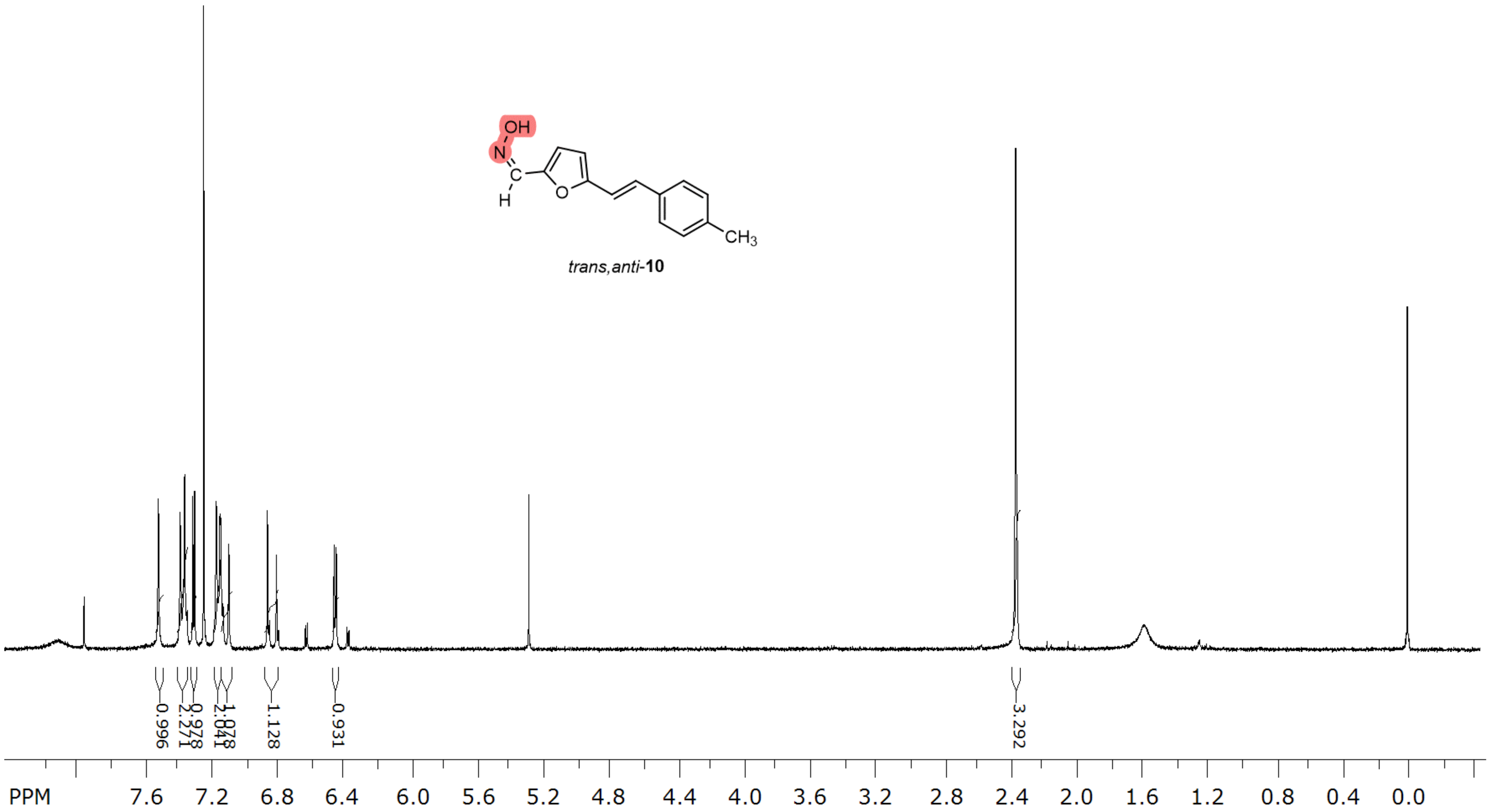

Figure S188. ^1H NMR ($CDCl_3$) spectrum of *trans,anti*-**10**.

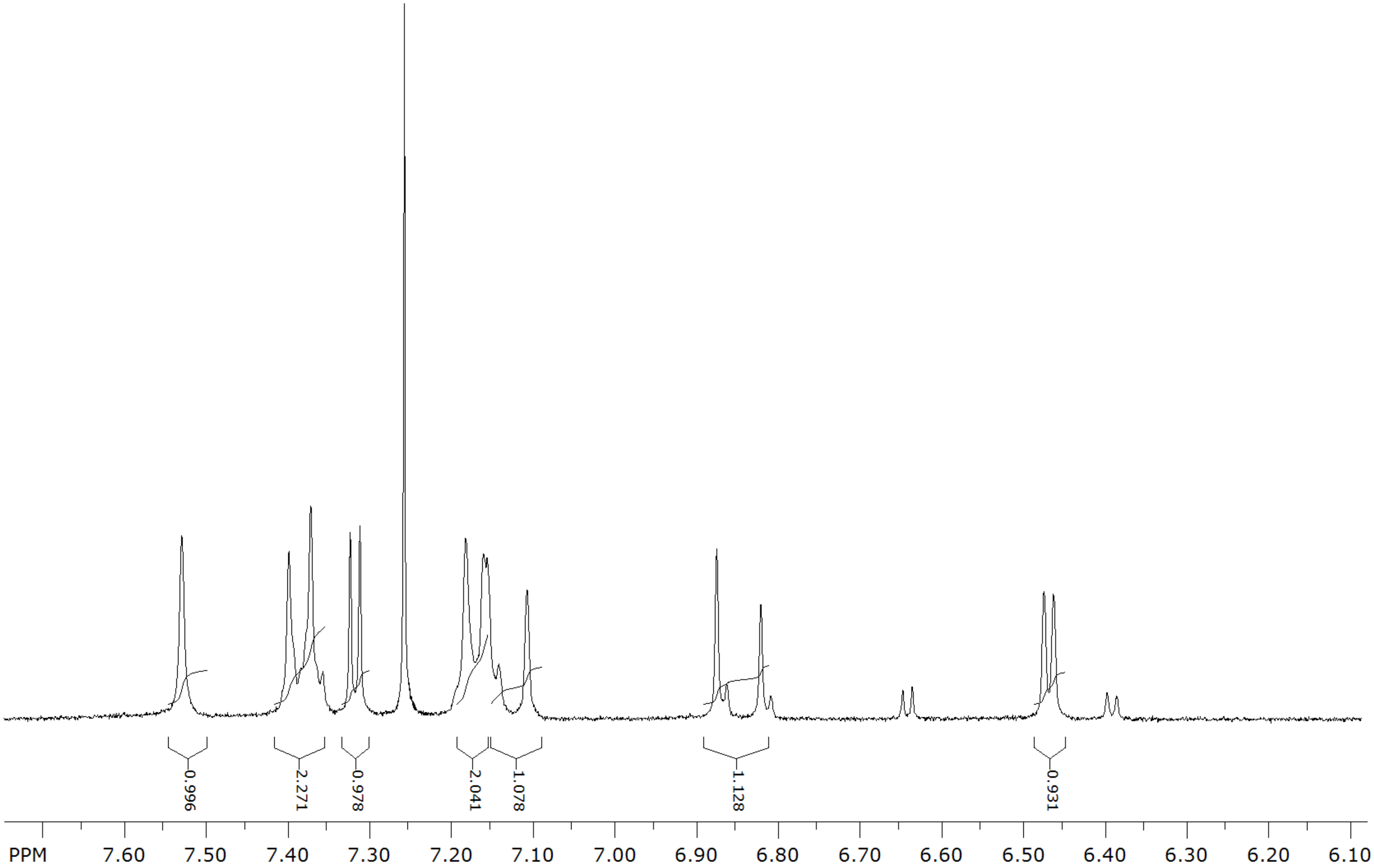

Figure S189. ^{1}H NMR ($CDCl_3$) spectrum of aromatic part of *trans,anti*-**10**.

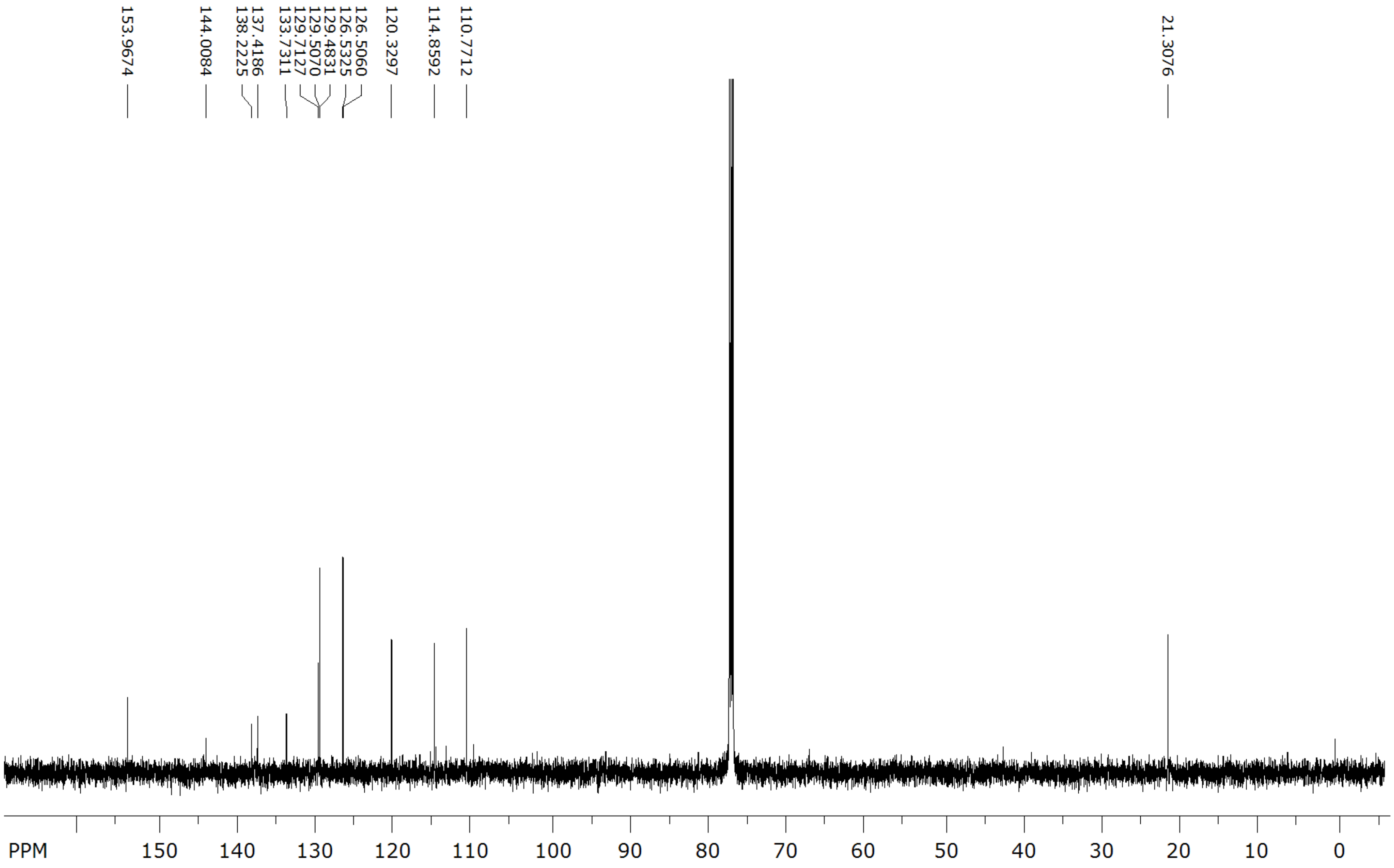

Figure S190. ^{13}C NMR ($CDCl_3$) spectrum of *trans,anti*-**10**.

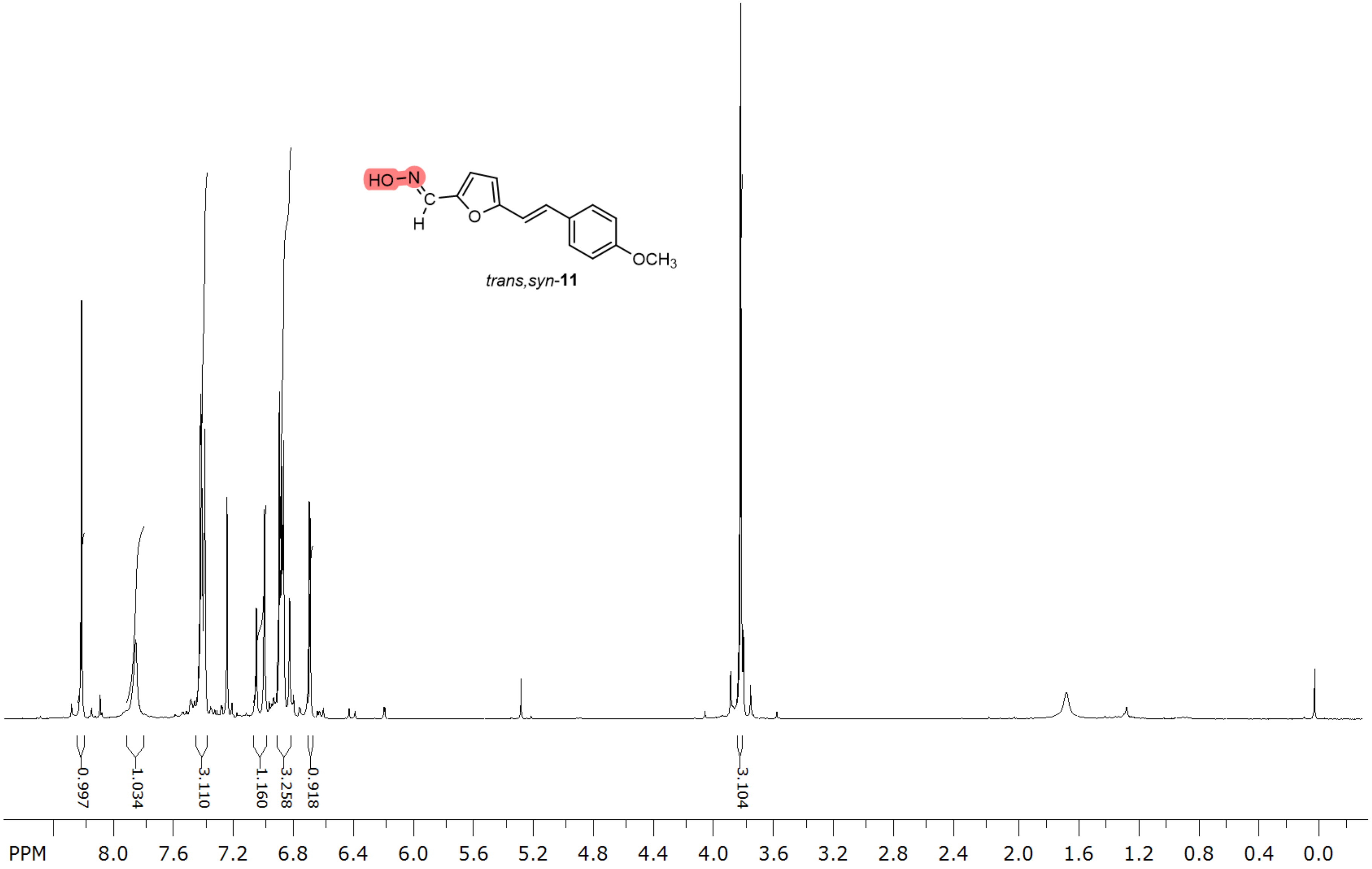

Figure S191. ^{1}H NMR ($CDCl_3$) spectrum of *trans,syn*-**11**.

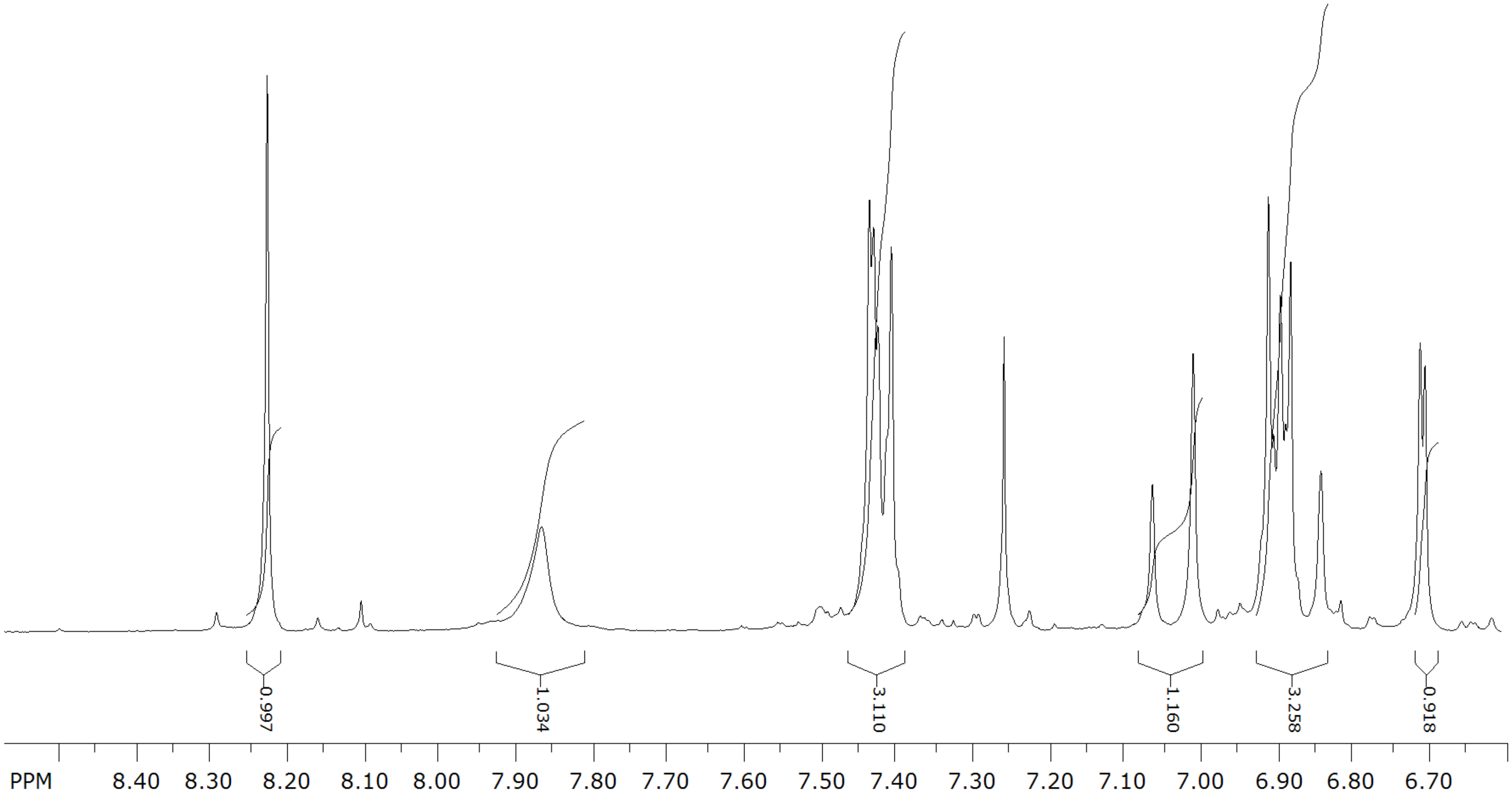

Figure S192. ^{1}H NMR ($CDCl_3$) spectrum of aromatic part of *trans,syn*-**11**.

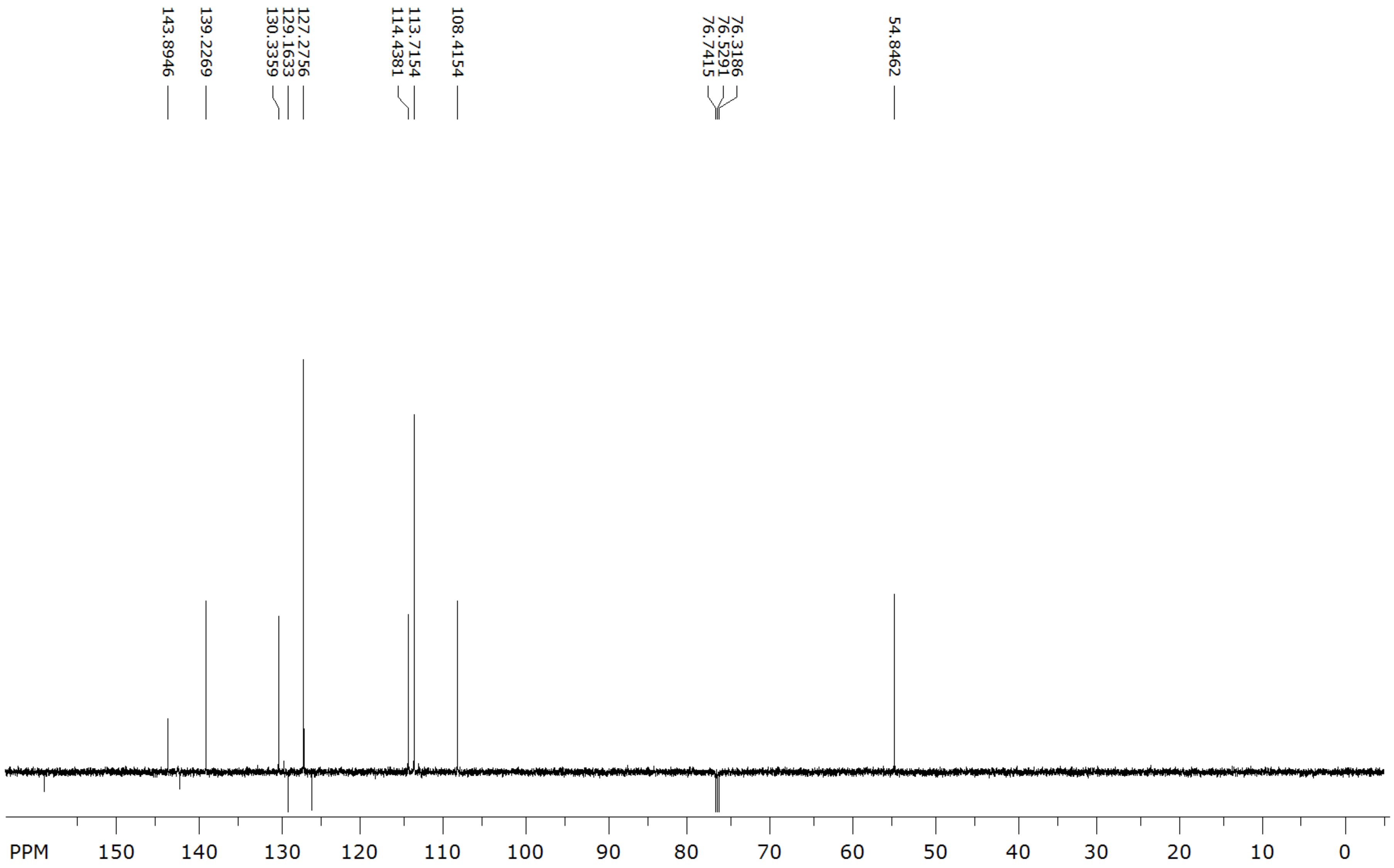

Figure S193. ^{13}C NMR ($CDCl_3$) spectrum of *trans,syn*-**11**.

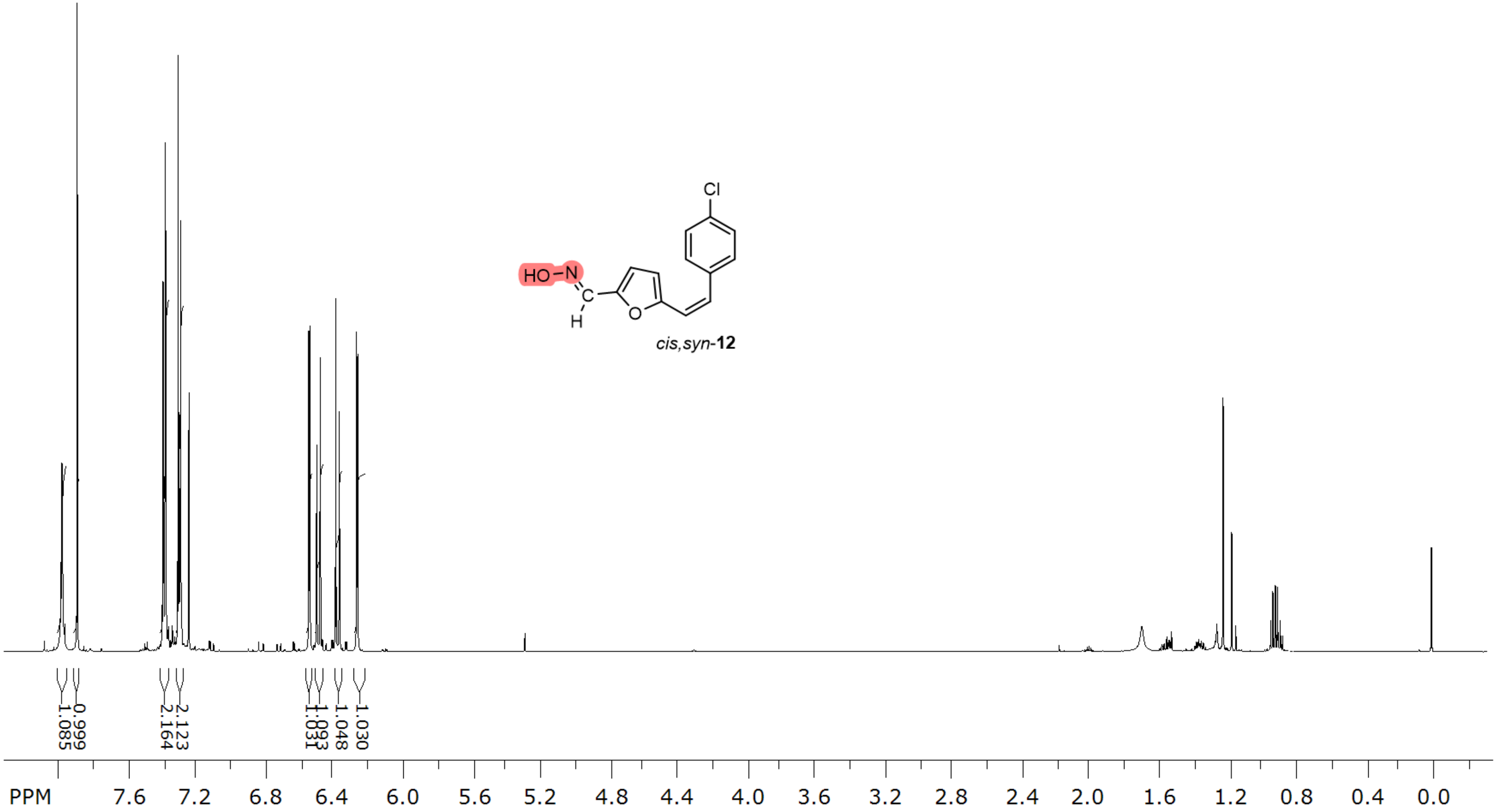

Figure S194. ^{1}H NMR ($CDCl_3$) spectrum of *cis,syn*-**12**.

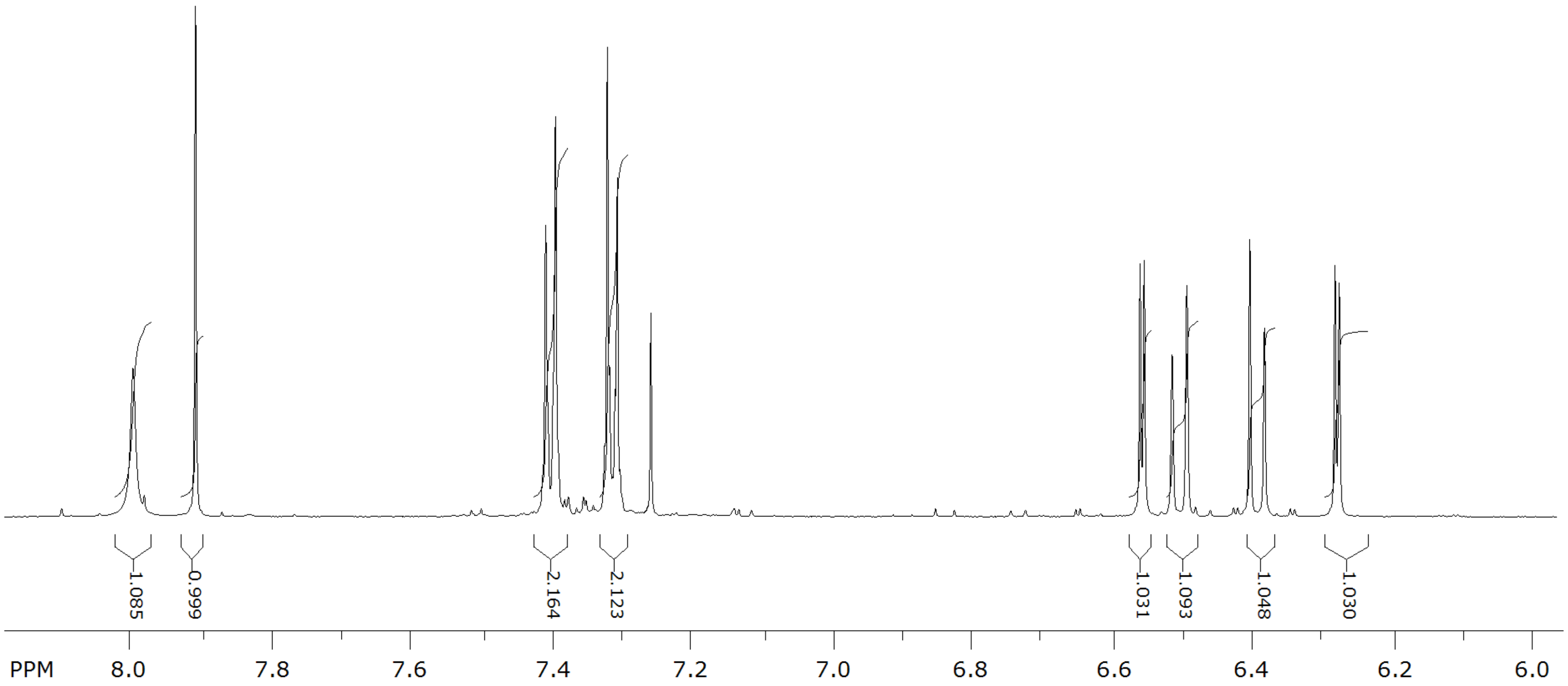

Figure S195. ^{1}H NMR ($CDCl_3$) spectrum of aromatic part of *cis,syn*-**12**.

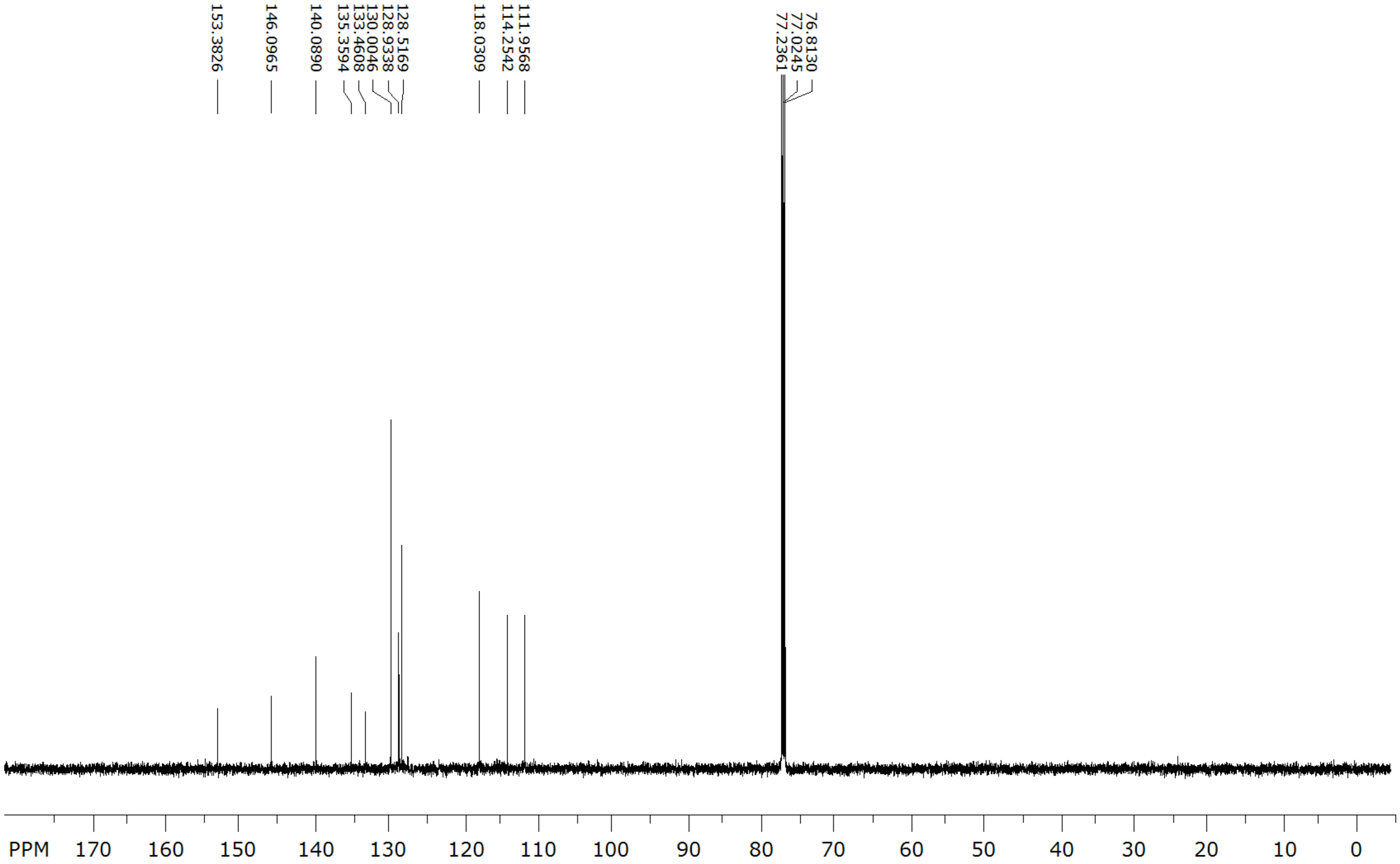

Figure S196. ^{13}C NMR ($CDCl_3$) spectrum of *cis,syn*-**12**.

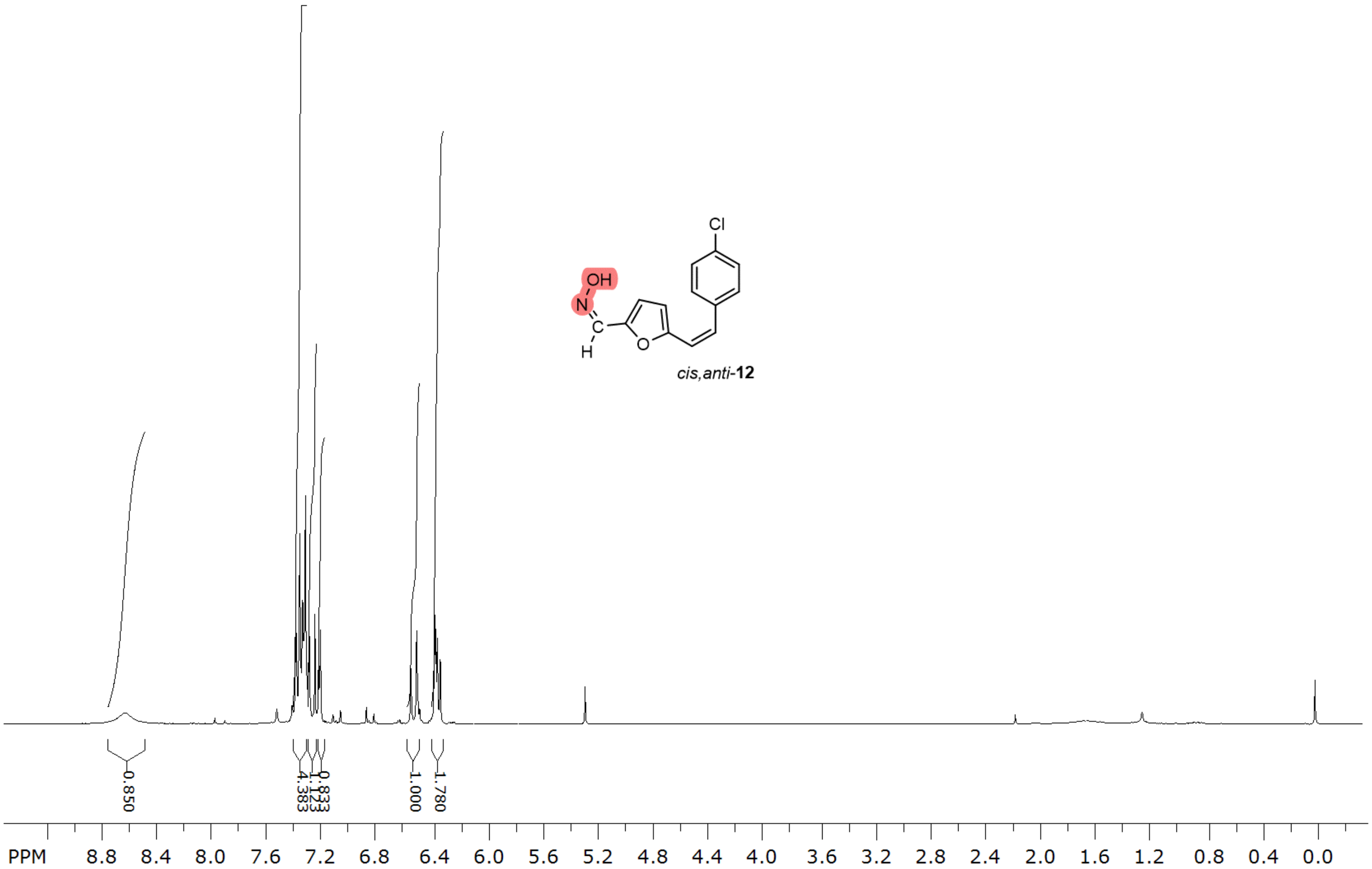

Figure S197. ^{1}H NMR ($CDCl_3$) spectrum of *cis,anti*-**12**.

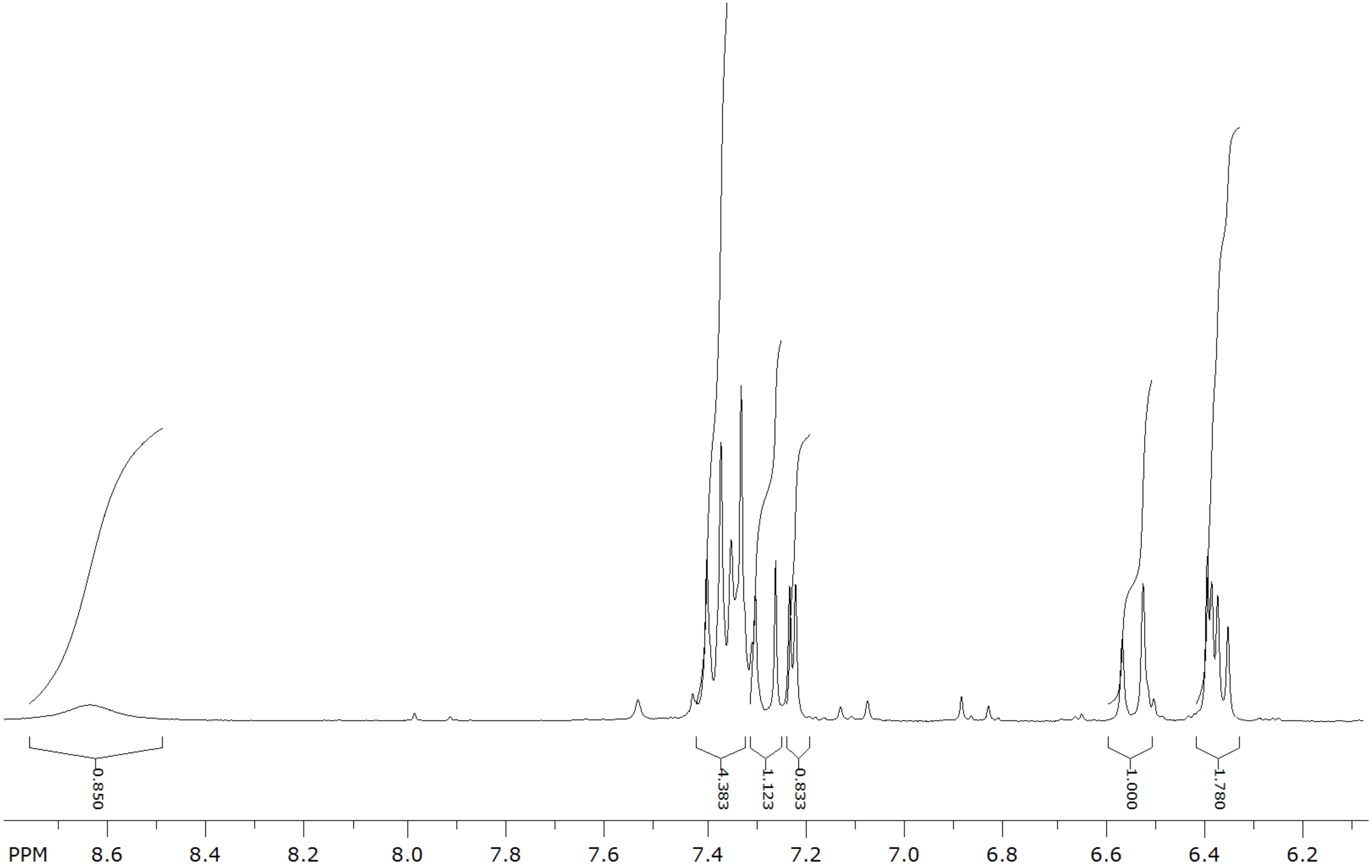

Figure S198. ^{1}H NMR ($CDCl_3$) spectrum of aromatic part of *cis,anti*-**12**.

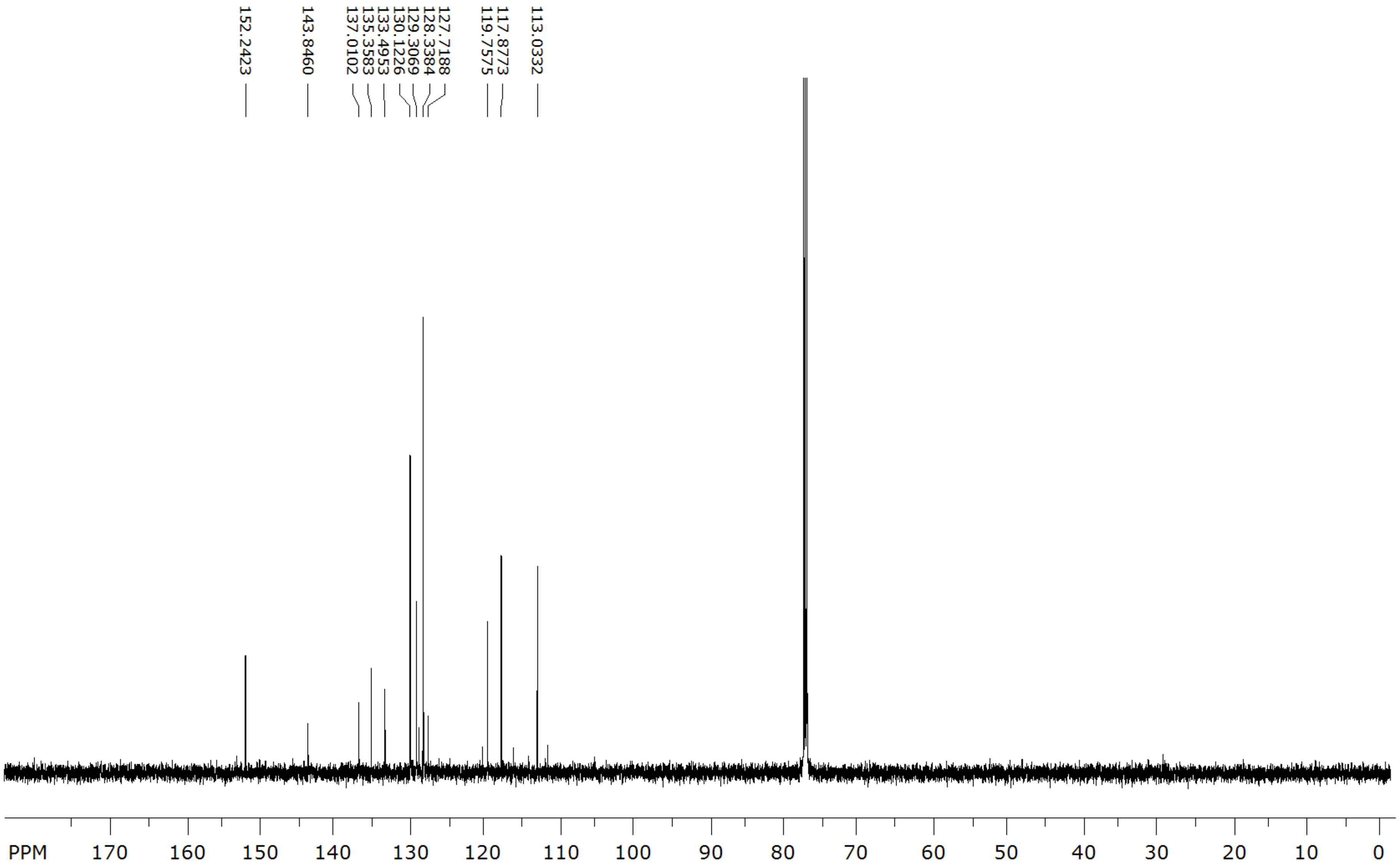

Figure S199. ^{13}C NMR ($CDCl_3$) spectrum of *cis,anti*-**12**.

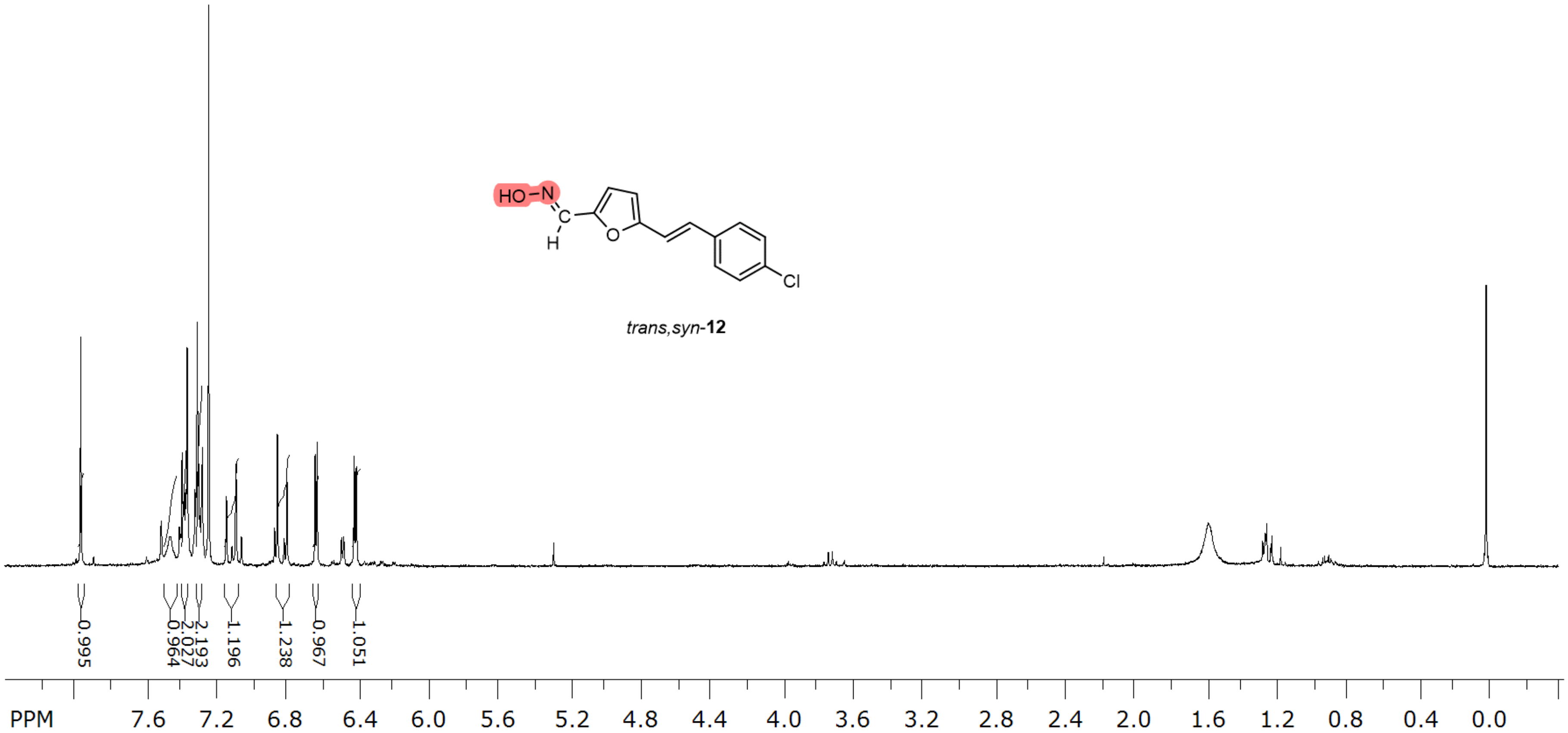

Figure S200. ^{1}H NMR ($CDCl_3$) spectrum of *trans,syn*-**12**.

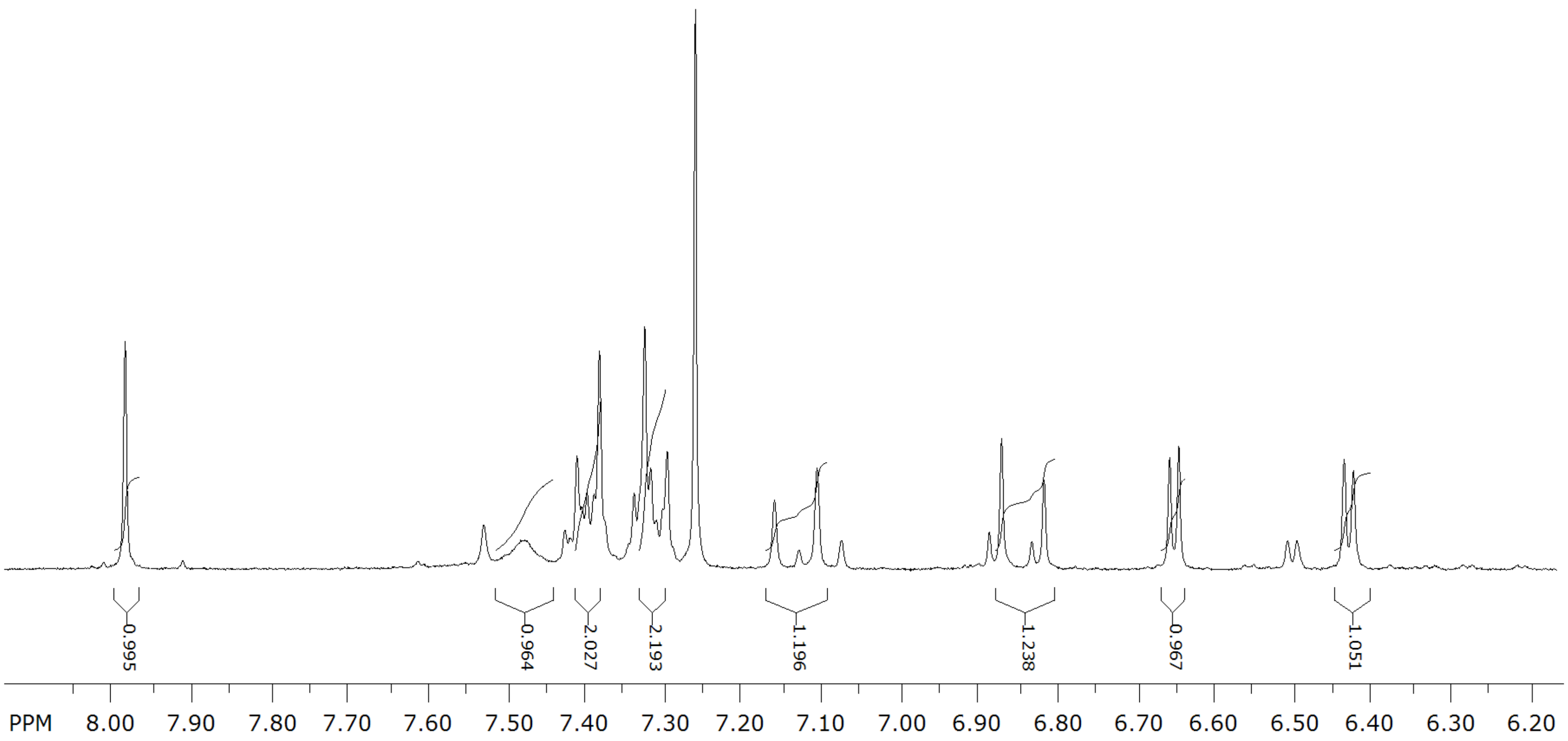

Figure S201. ^{1}H NMR ($CDCl_3$) spectrum of aromatic part of *trans,syn*-**12**.

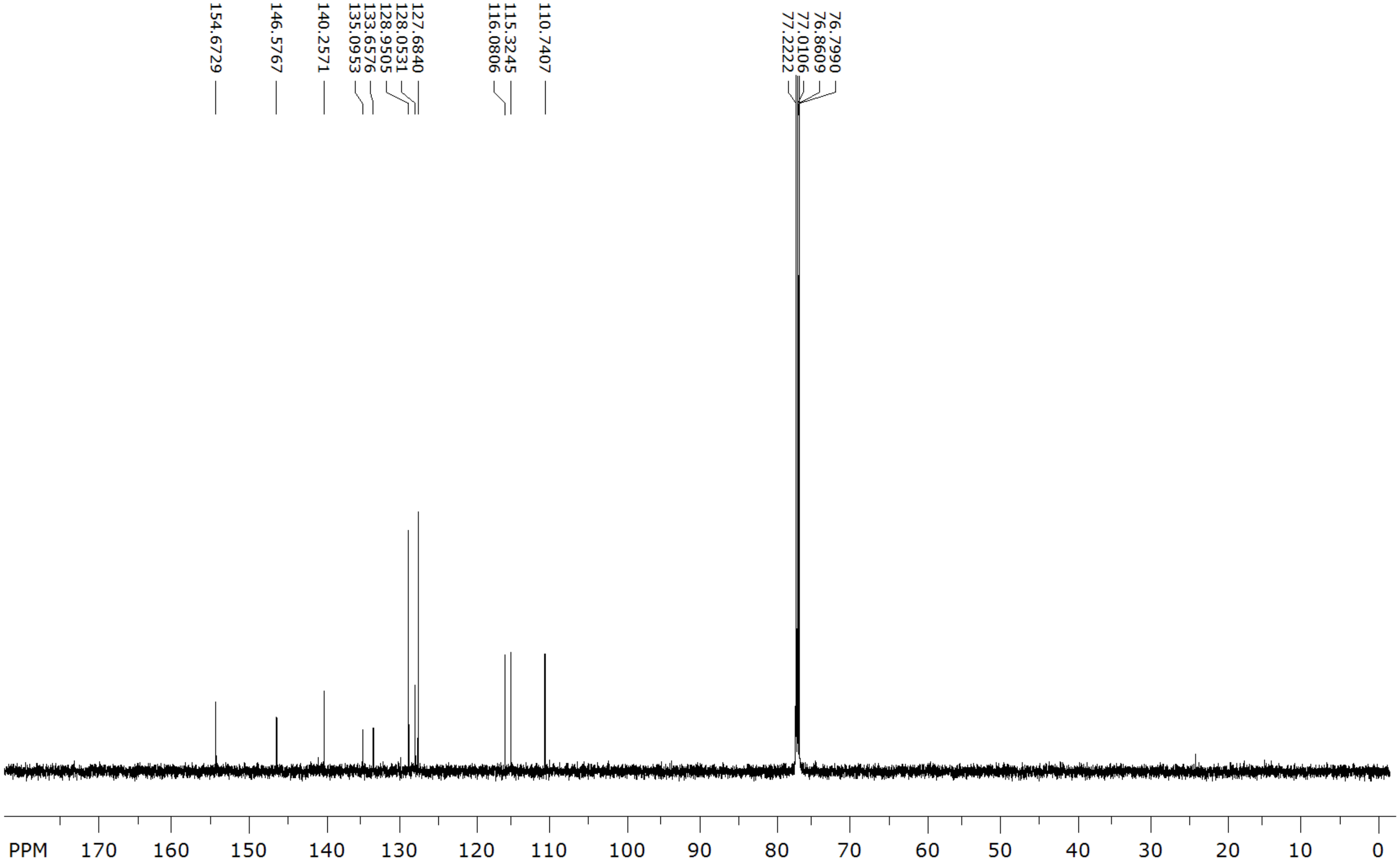

Figure S202. ^{13}C NMR ($CDCl_3$) spectrum of *trans,syn*-**12**.

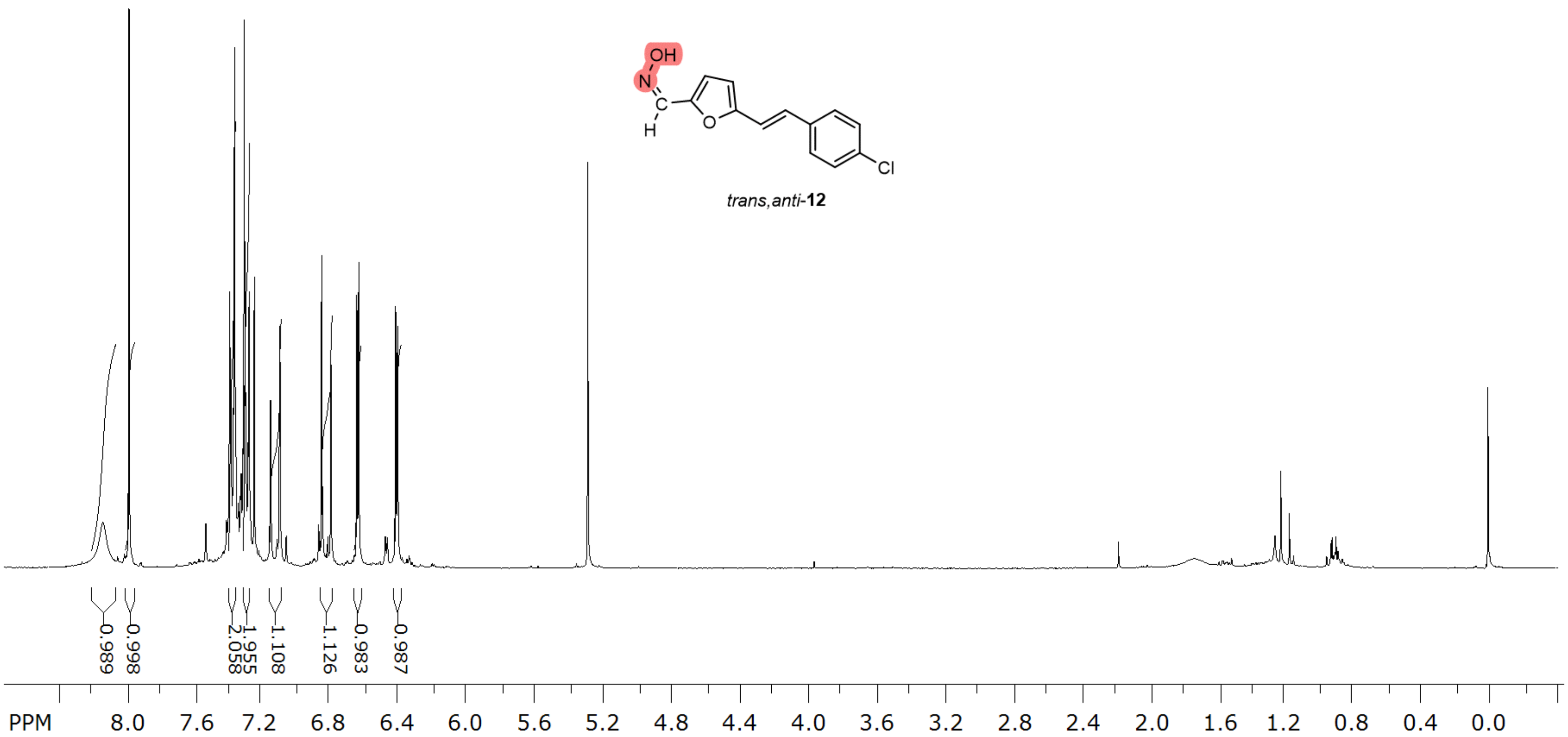

Figure S203. ^{1}H NMR ($CDCl_3$) spectrum of *trans,anti*-**12**.

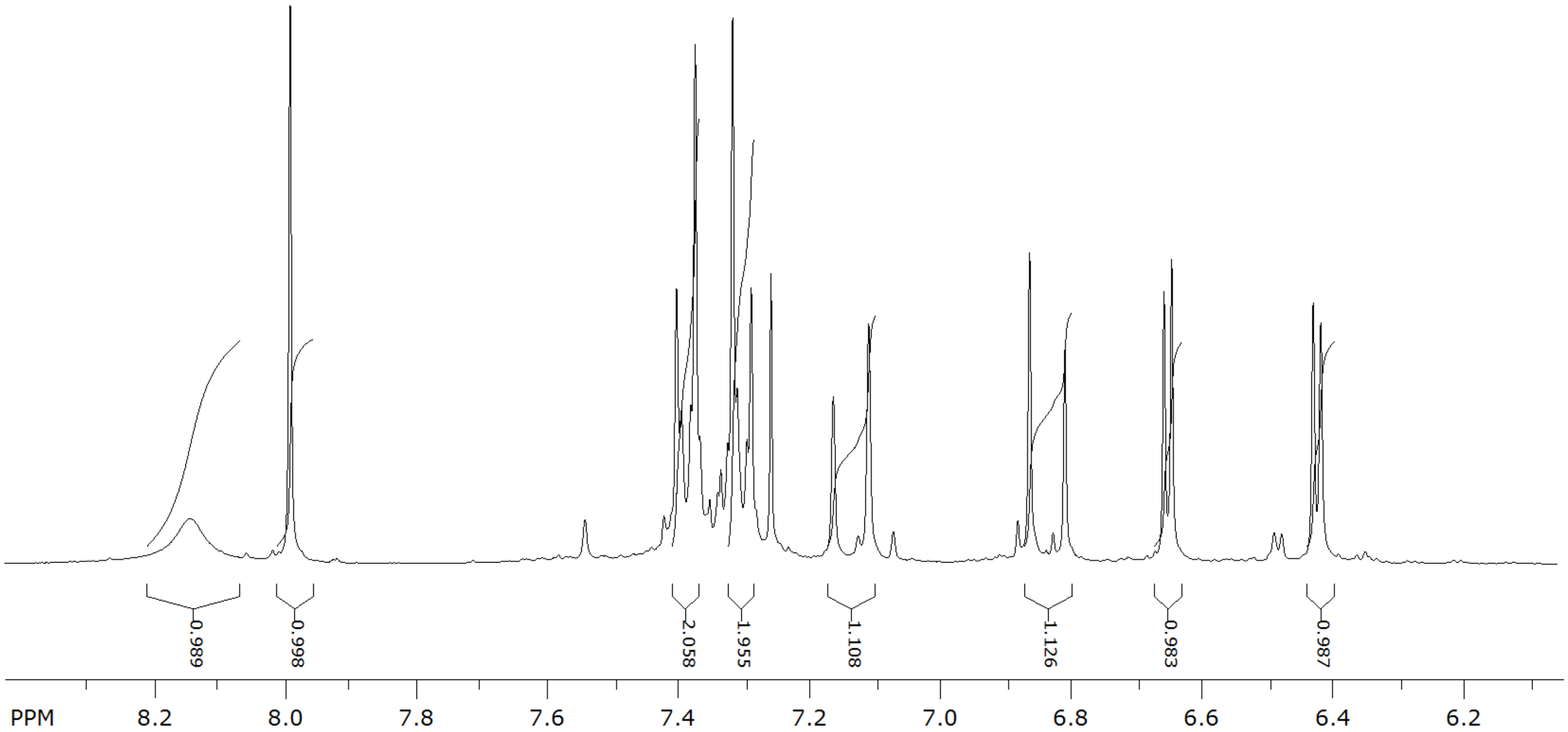

Figure S204. ^{1}H NMR ($CDCl_3$) spectrum of *trans,anti*-**12**.

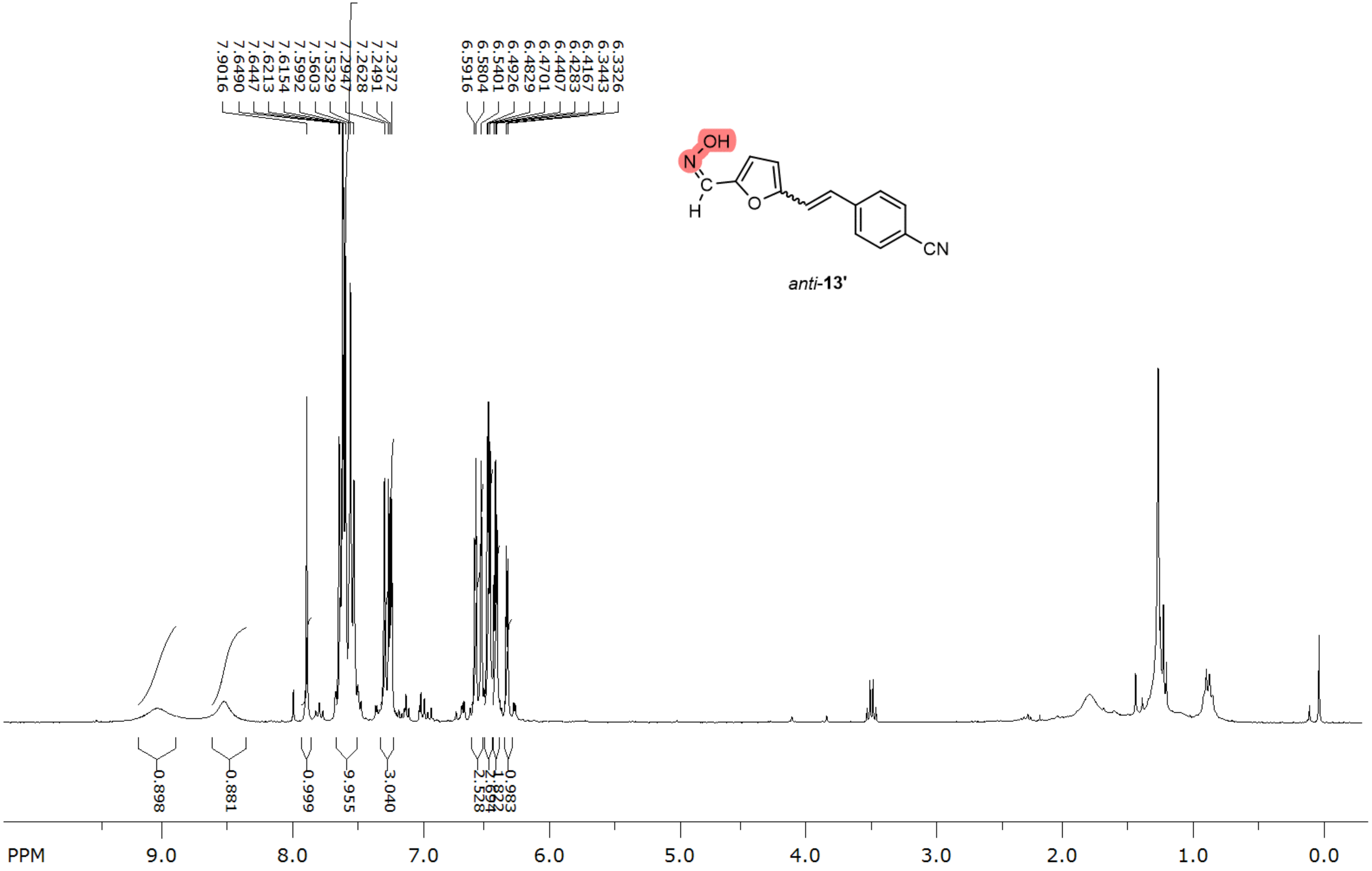

Figure S205. ^{1}H NMR ($CDCl_3$) spectrum of *trans*-**13'**.

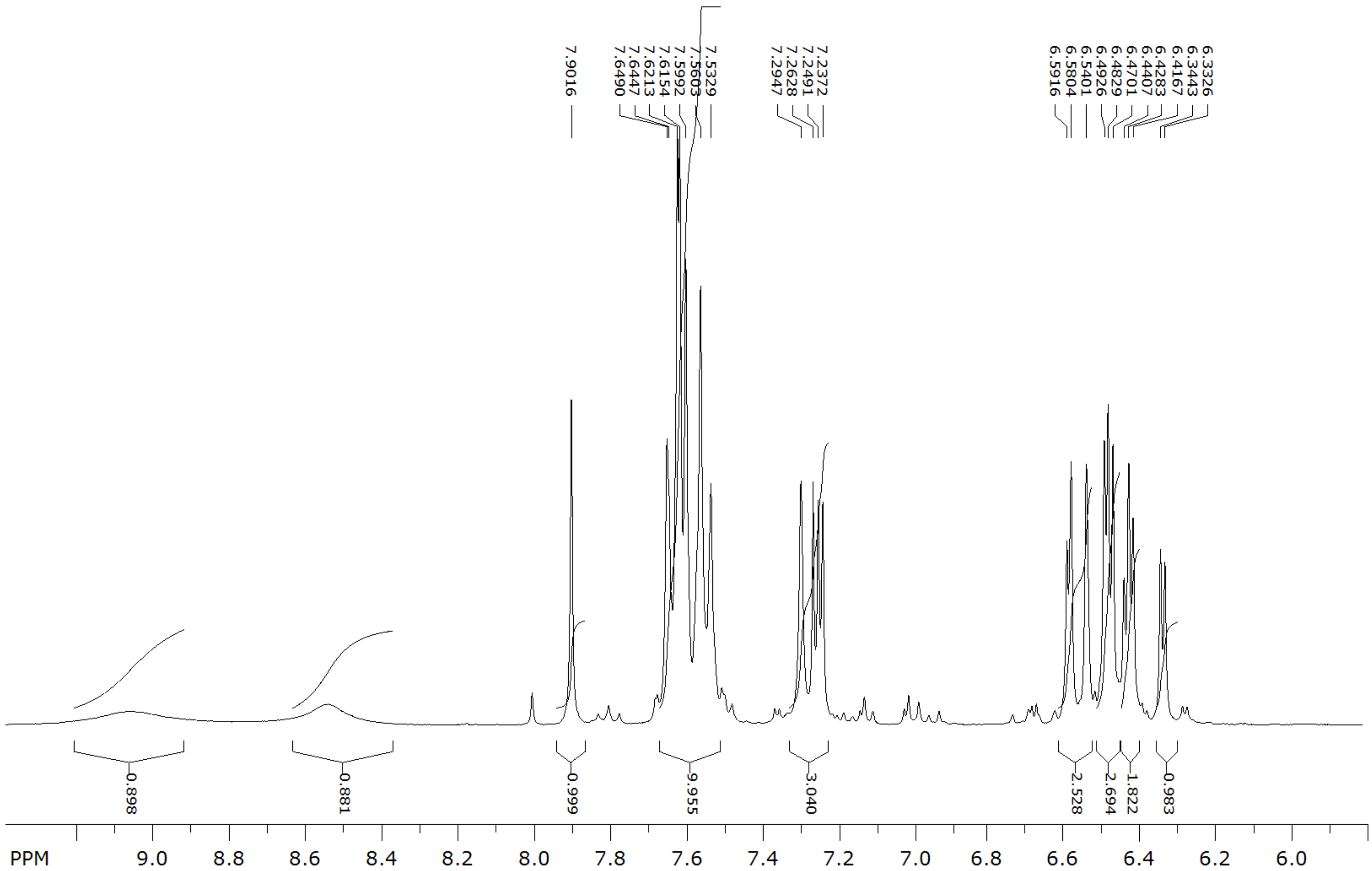

Figure S206. ^{13}C NMR ($CDCl_3$) spectrum of aromatic part of *trans*-**13'**.

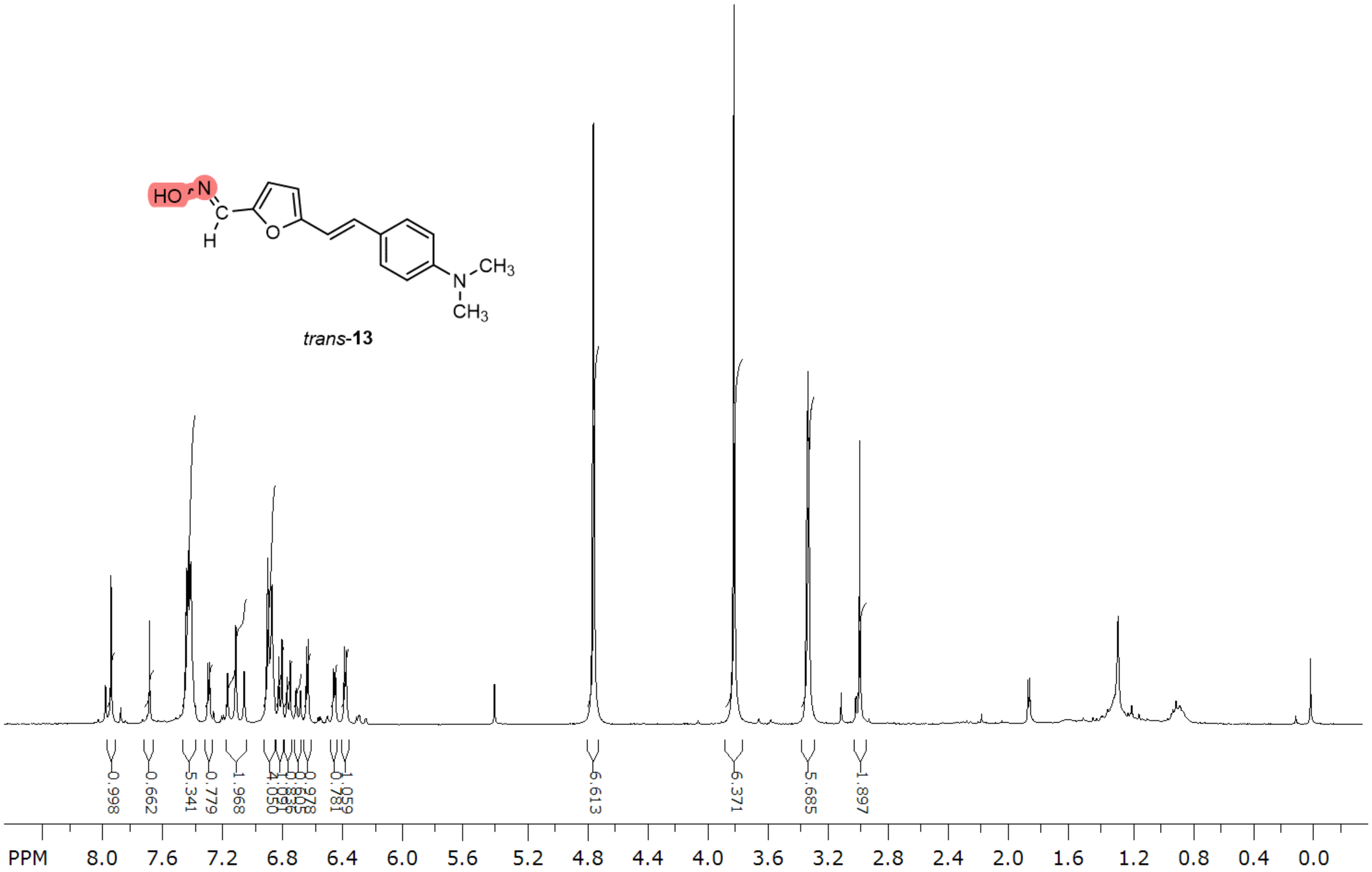

Figure S207. ^{1}H NMR ($CDCl_3$) spectrum of *trans*-**13**.

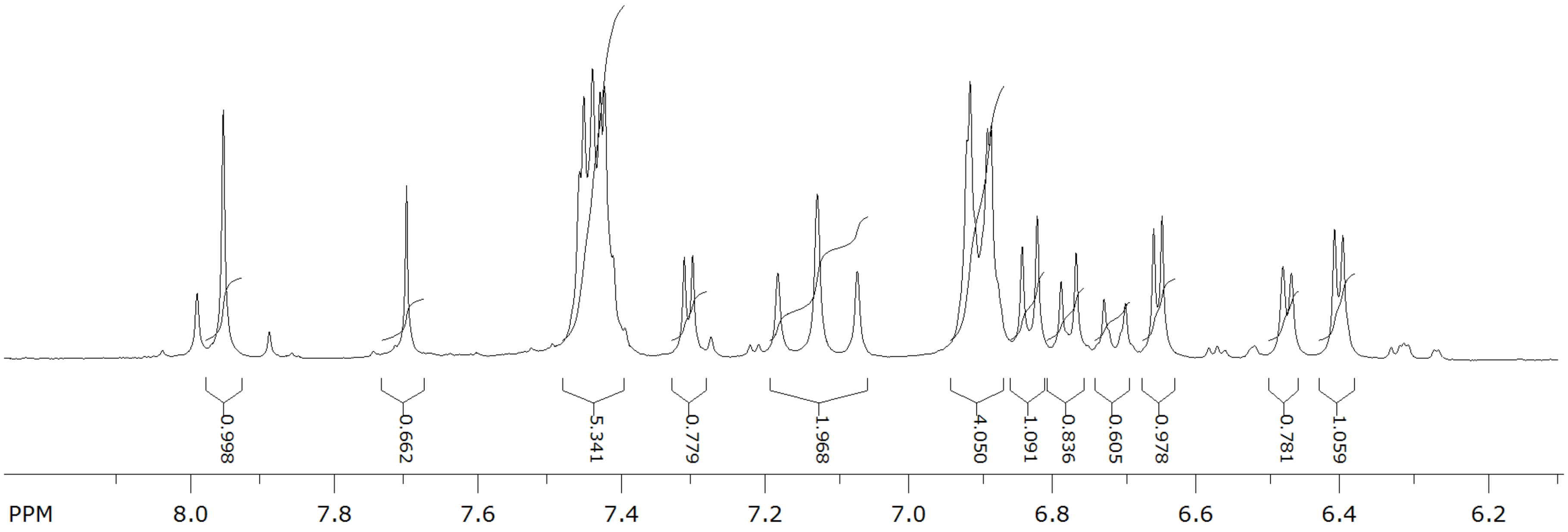

Figure S208. ^{13}C NMR ($CDCl_3$) spectrum of aromatic part of *trans*-**13**.

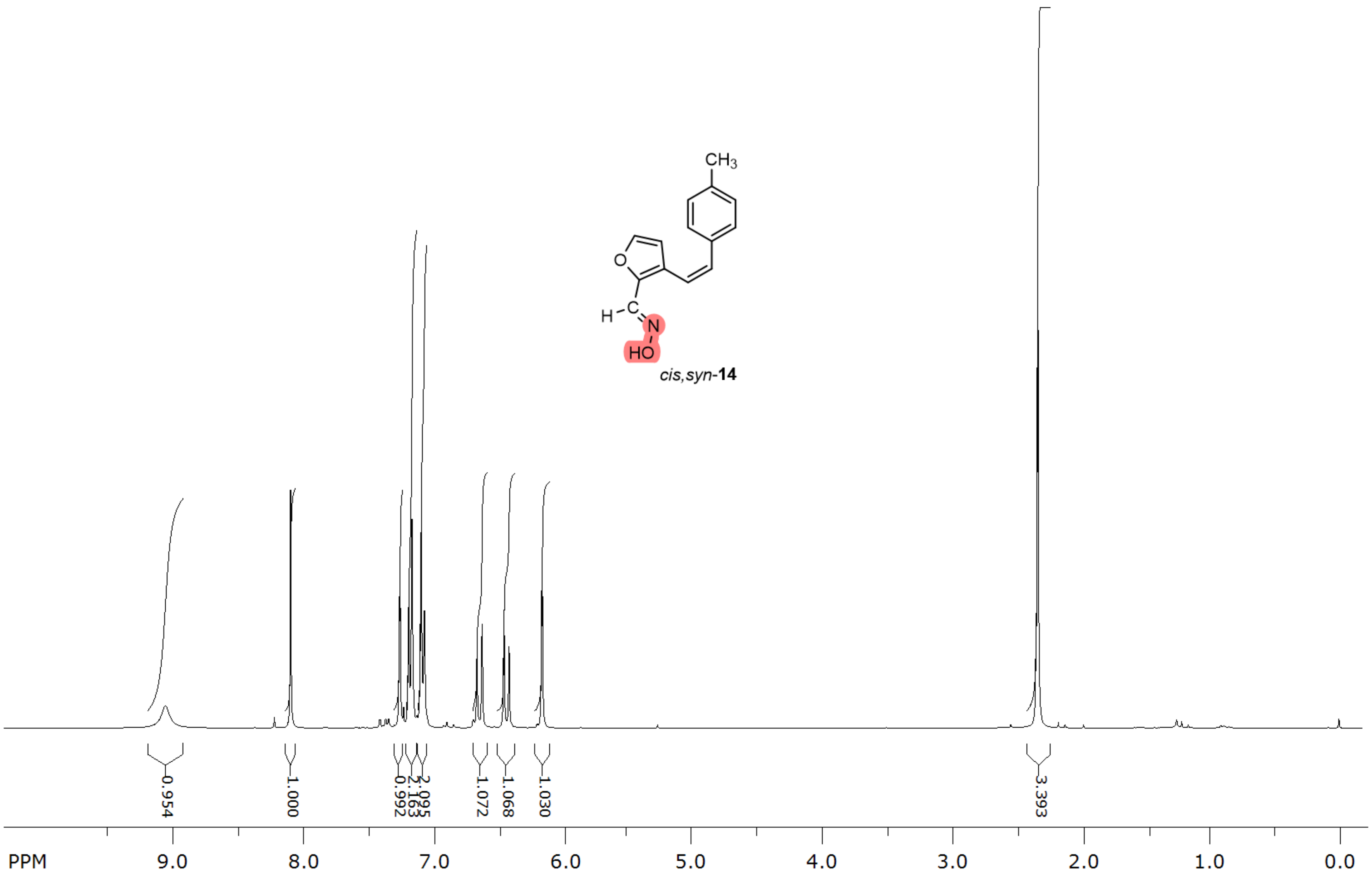

Figure S209. ^{1}H NMR ($CDCl_3$) spectrum of *cis,syn*-**14**.

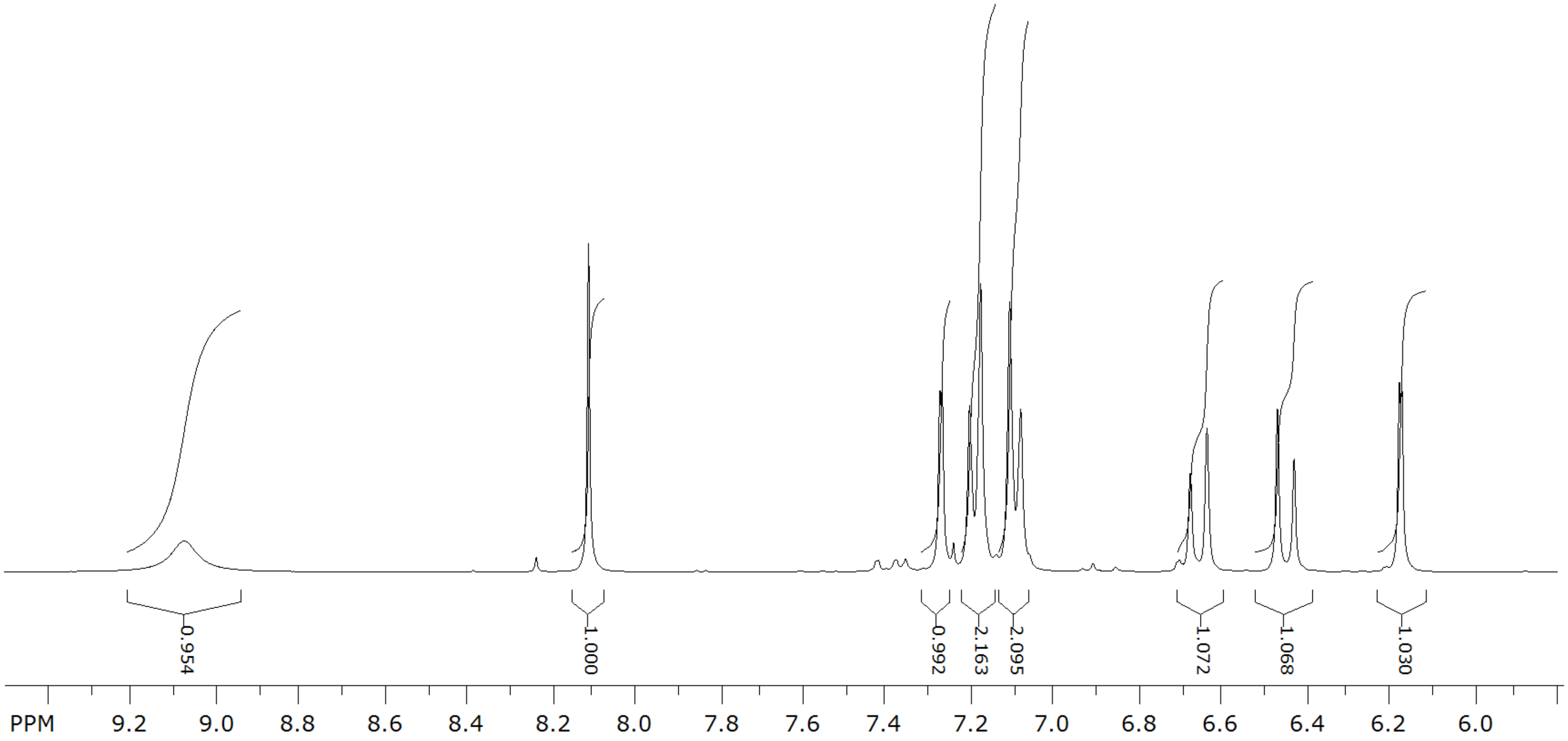

Figure S210. ^1H NMR ($CDCl_3$) spectrum of aromatic part of *cis,syn*-**14**.

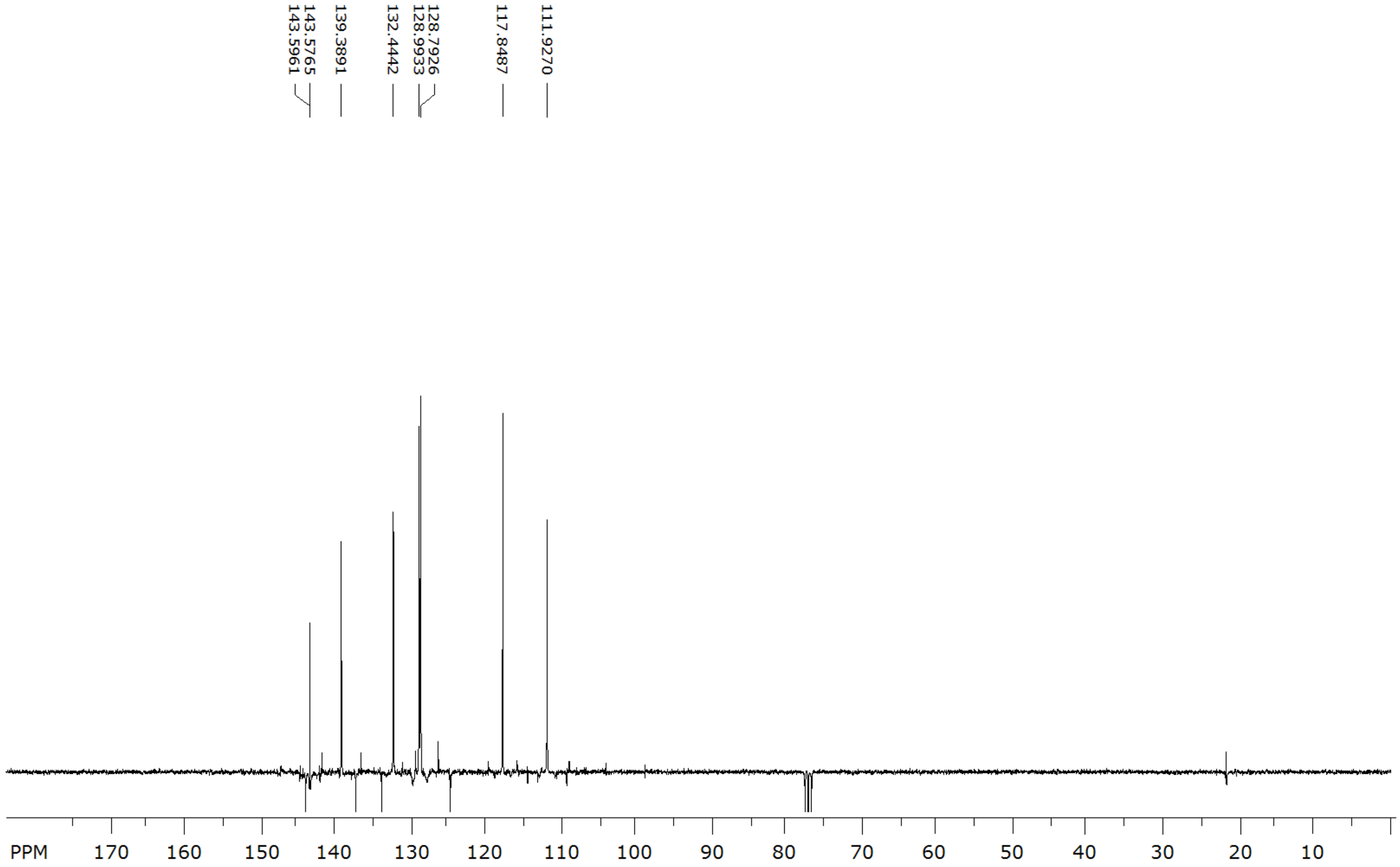

Figure S211. ^{13}C NMR ($CDCl_3$) spectrum of *cis,syn*-**14**.

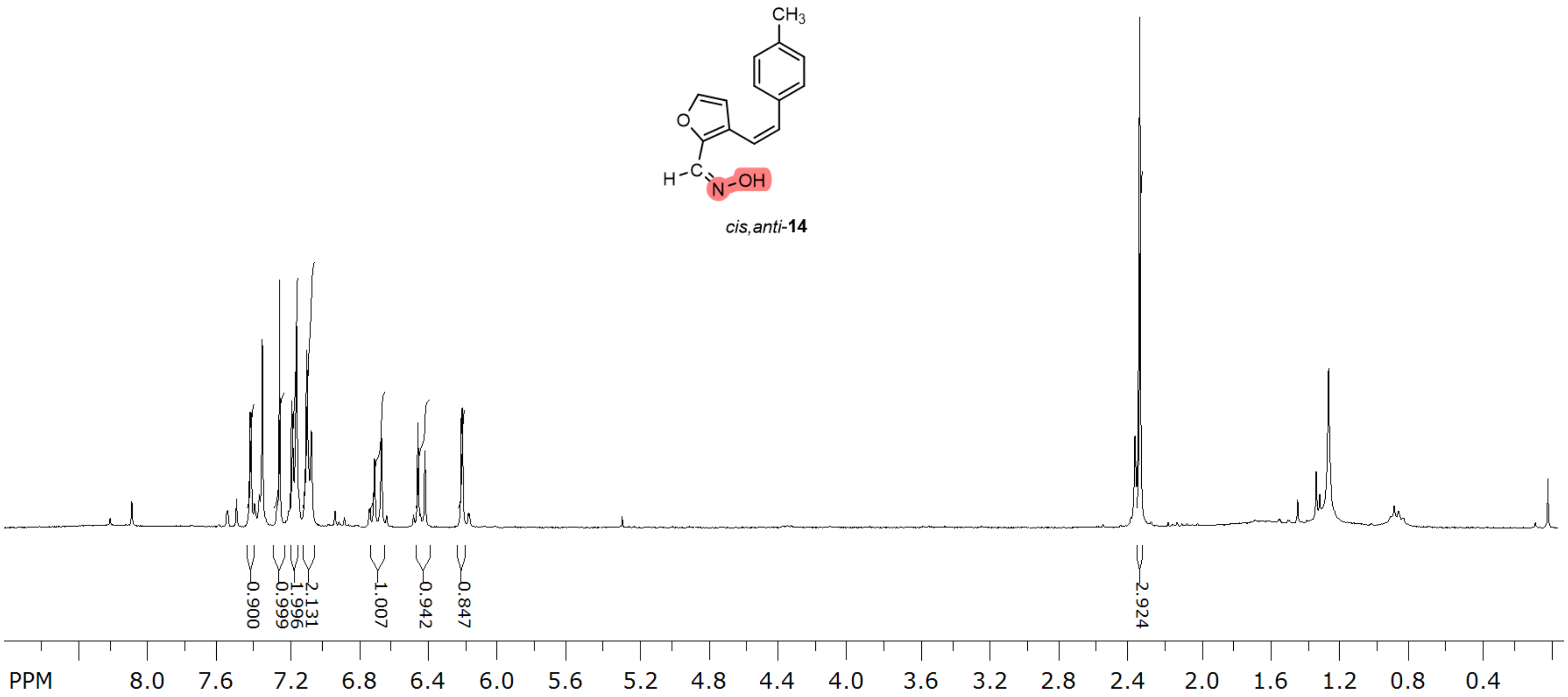

Figure S212. ^{1}H NMR ($CDCl_3$) spectrum of *cis,anti*-**14**.

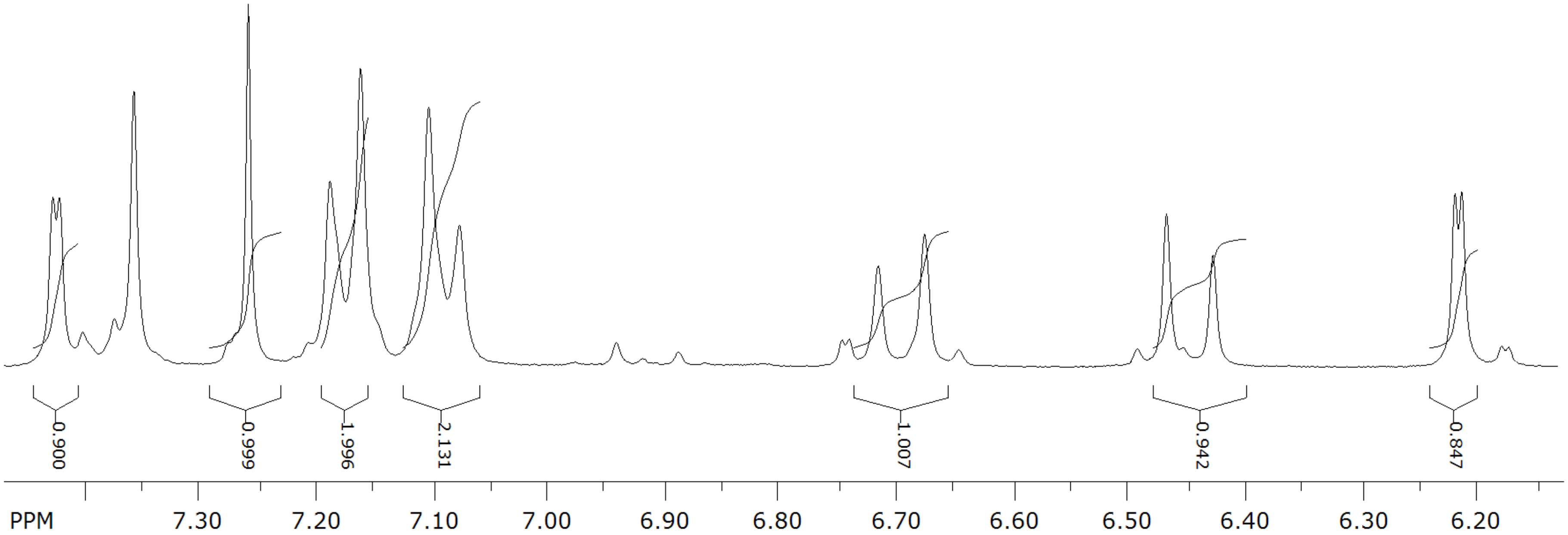

Figure S213. ^{1}H NMR ($CDCl_3$) spectrum of aromatic part of *cis,anti*-**14**.

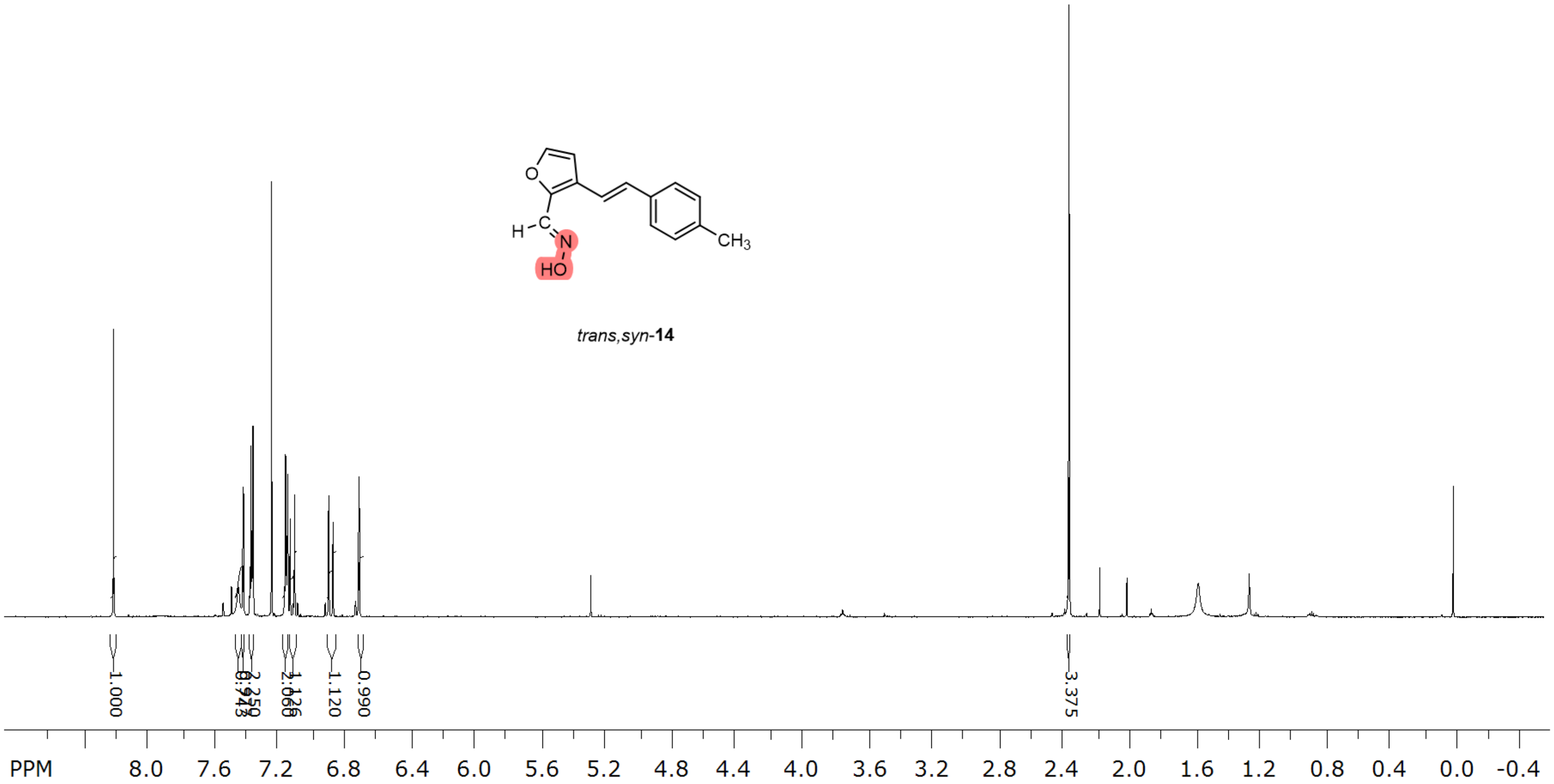

Figure S214. ^{1}H NMR ($CDCl_3$) spectrum of *trans,syn*-**14**.

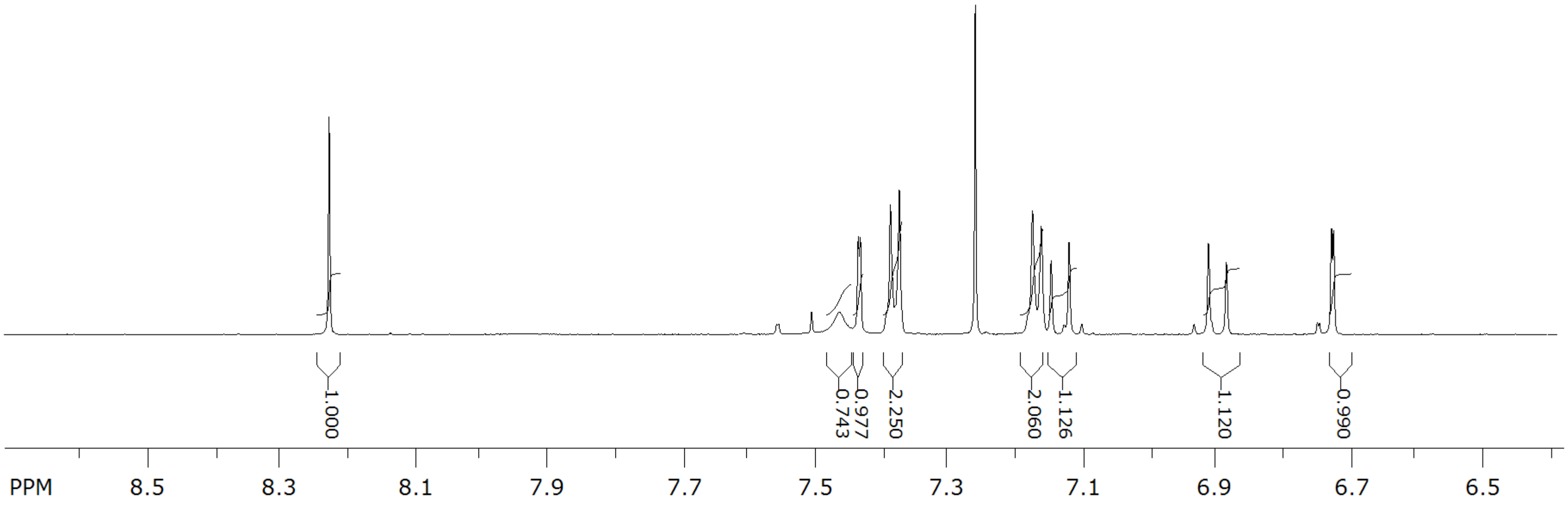

Figure S215. ^{1}H NMR ($CDCl_3$) spectrum of aromatic part of *trans,syn*-**14**.

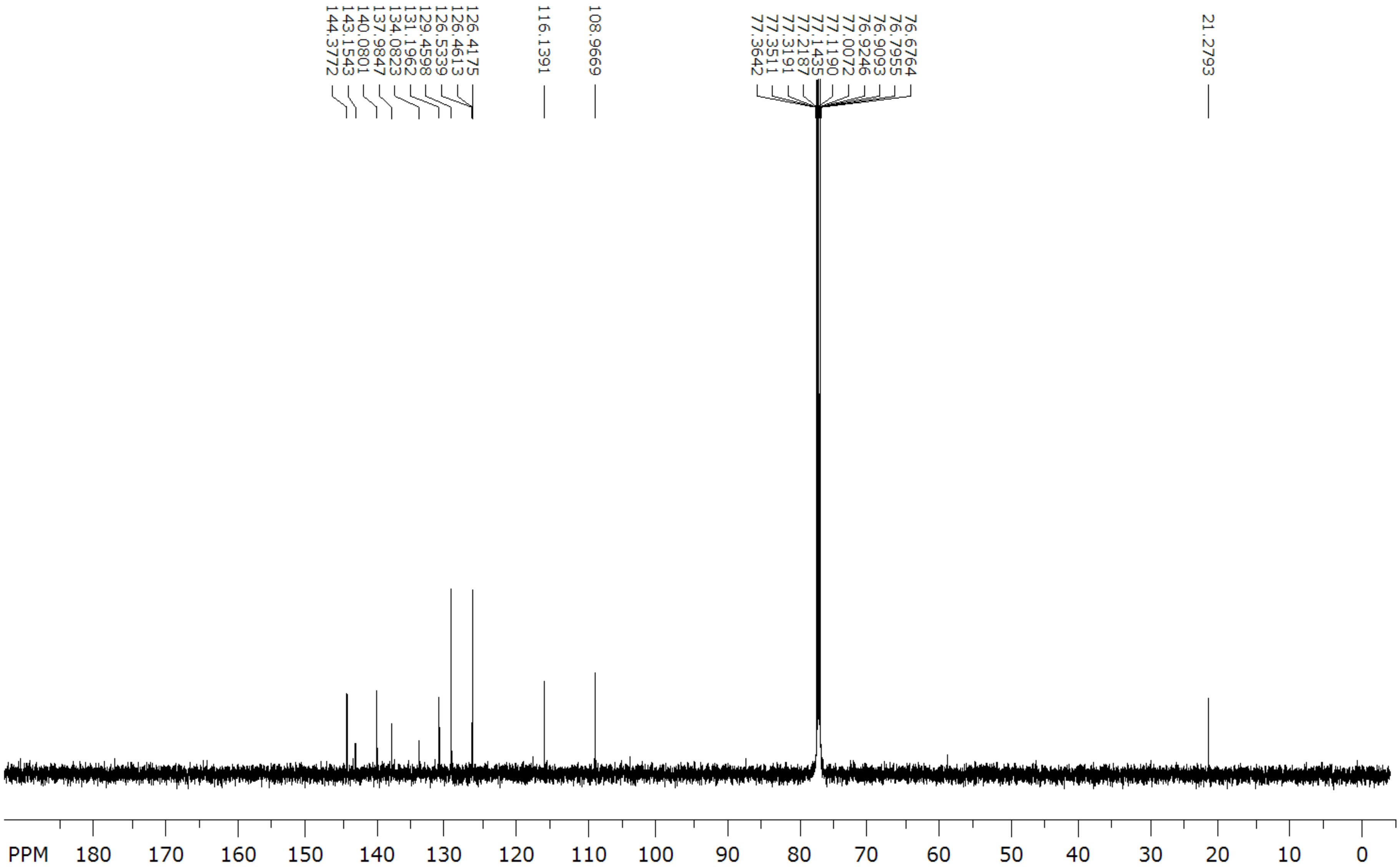

Figure S216. ^{13}C NMR ($CDCl_3$) spectrum of *trans,syn*-**14**.

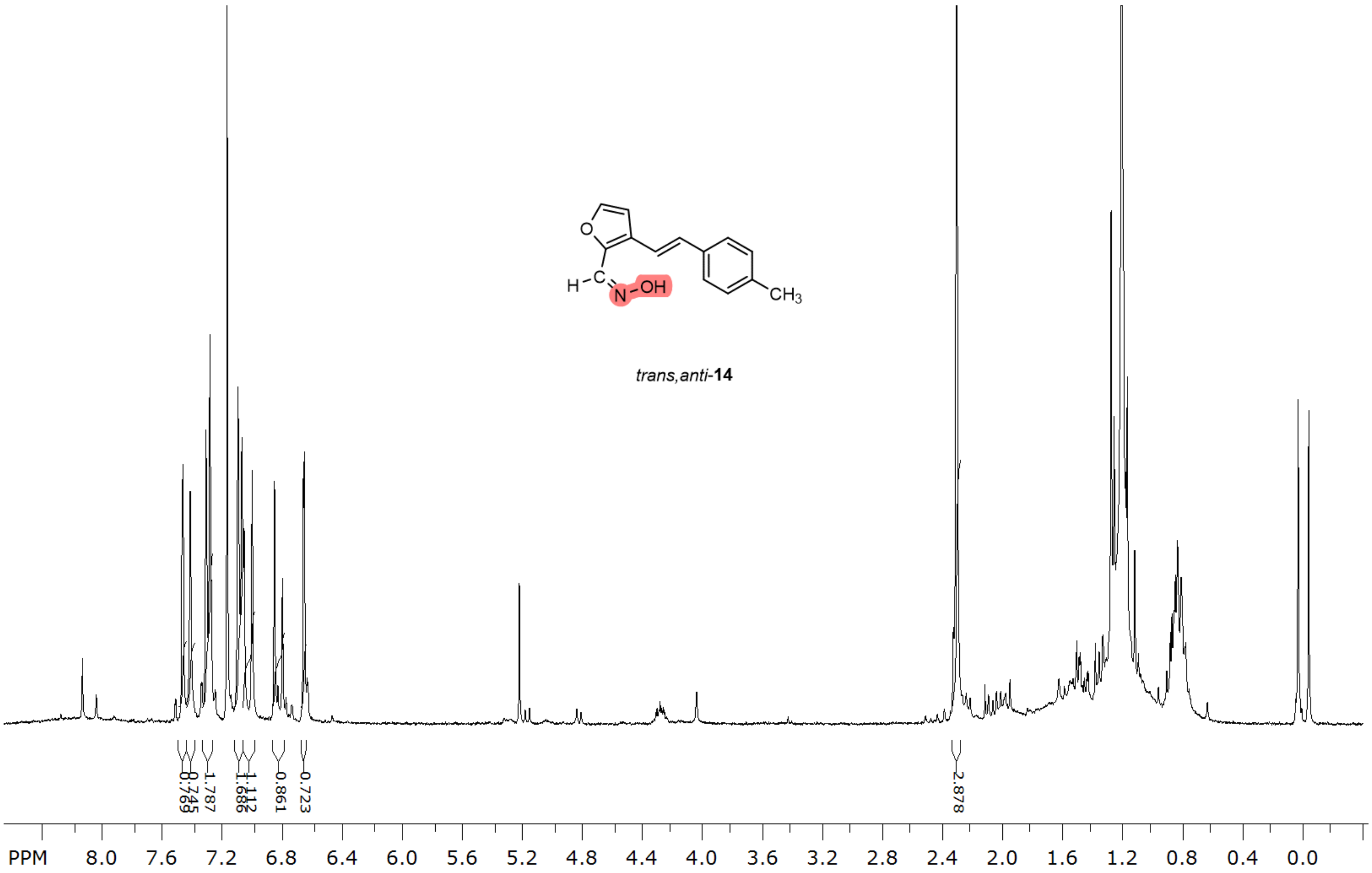

Figure S217. ^1H NMR ($CDCl_3$) spectrum of *trans,anti*-**14**.

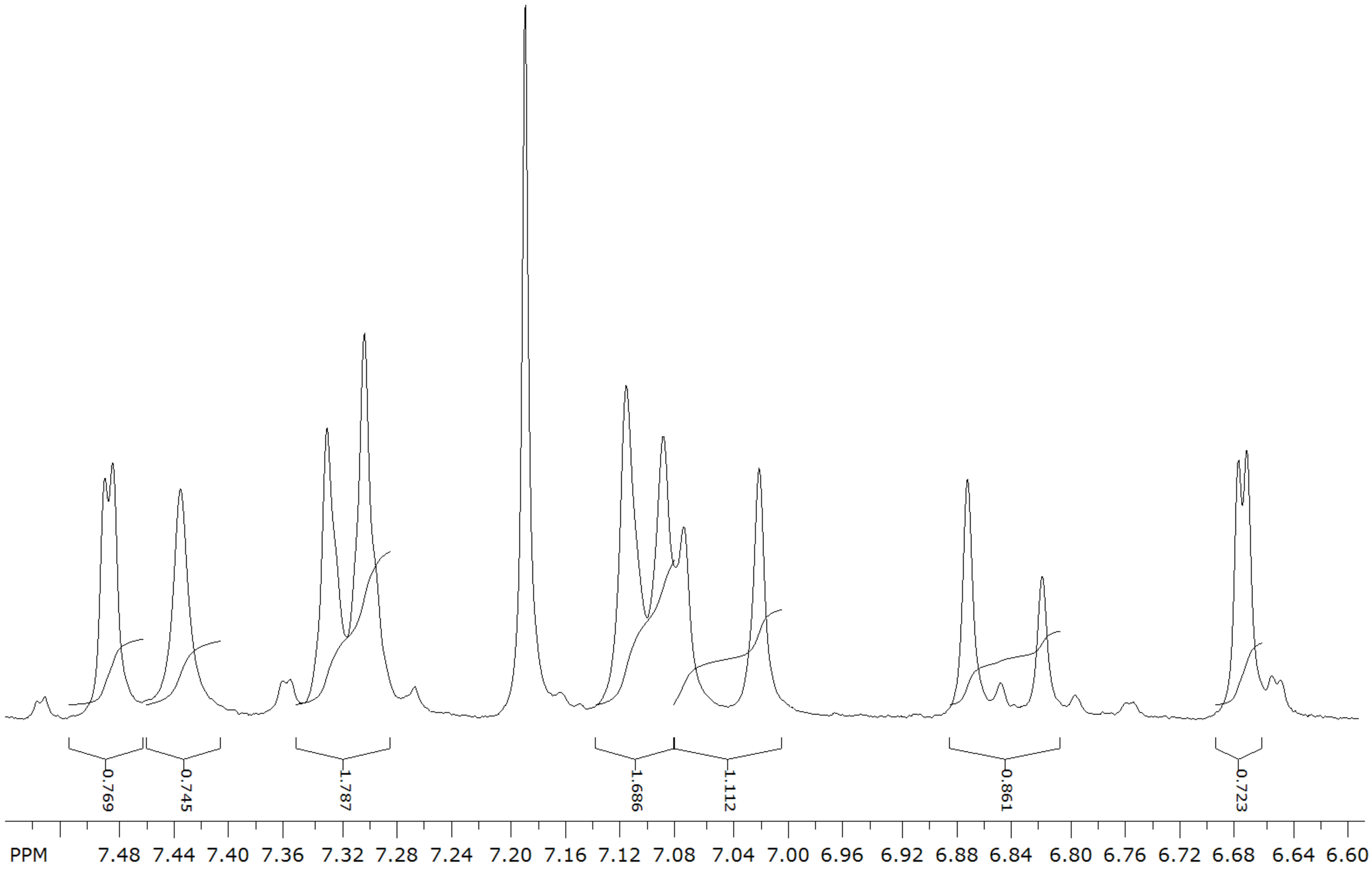

Figure S218. ^{1}H NMR ($CDCl_3$) spectrum of aromatic part of *trans,anti*-**14**.

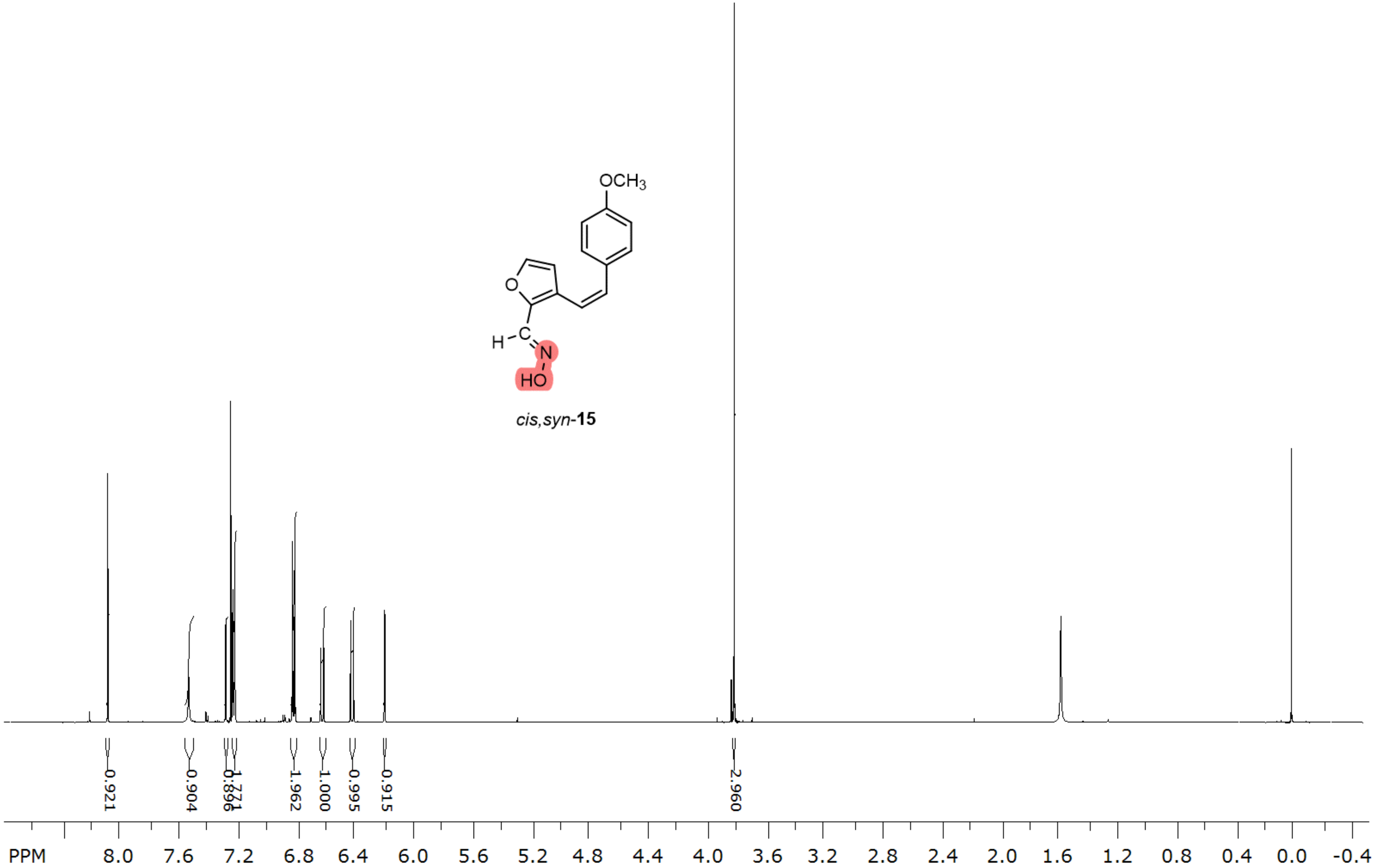

Figure S219. ^{1}H NMR ($CDCl_3$) spectrum of *cis,syn*-**15**.

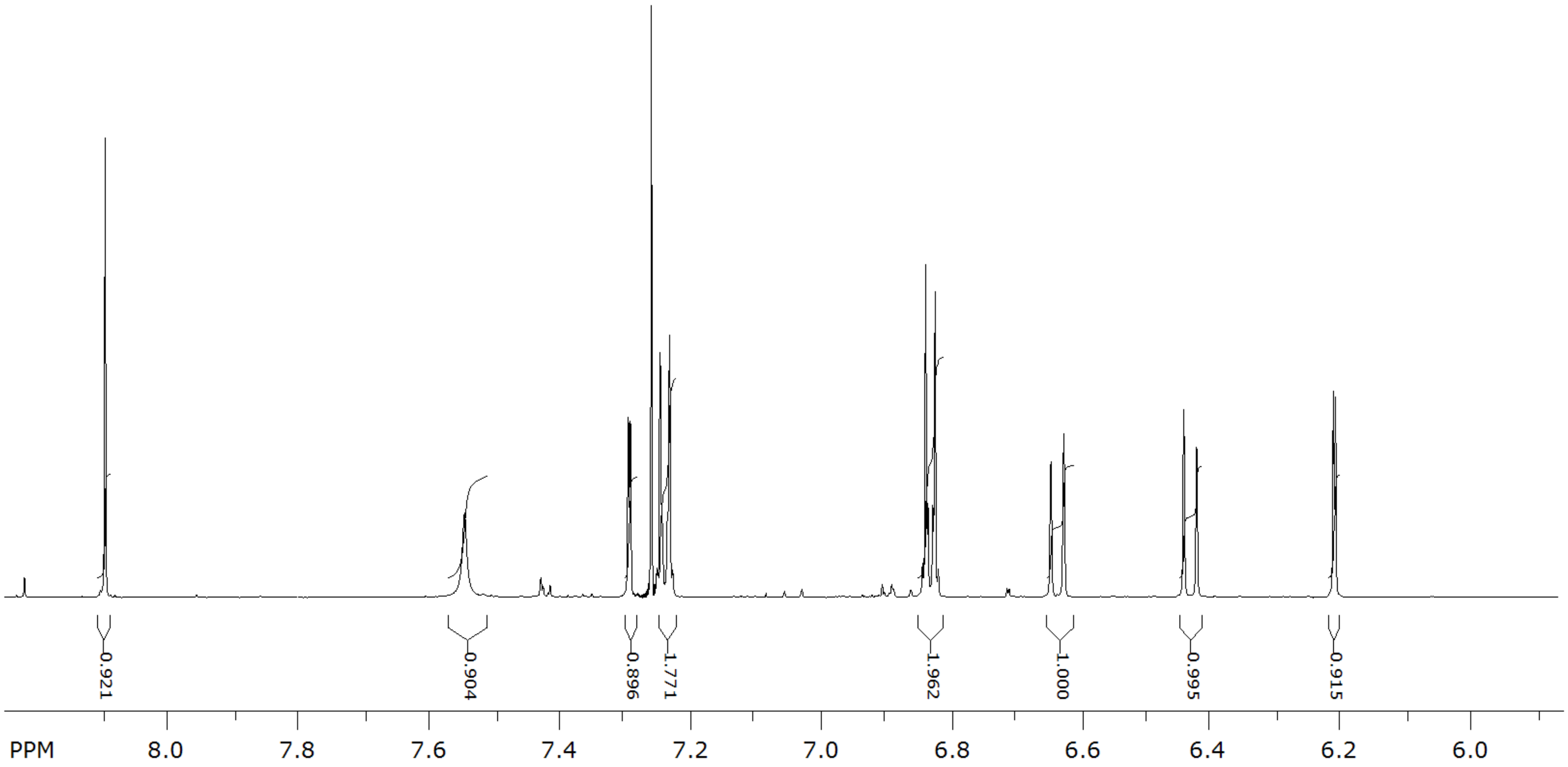

Figure S220. ^{1}H NMR ($CDCl_3$) spectrum of aromatic part of *cis,syn*-**15**.

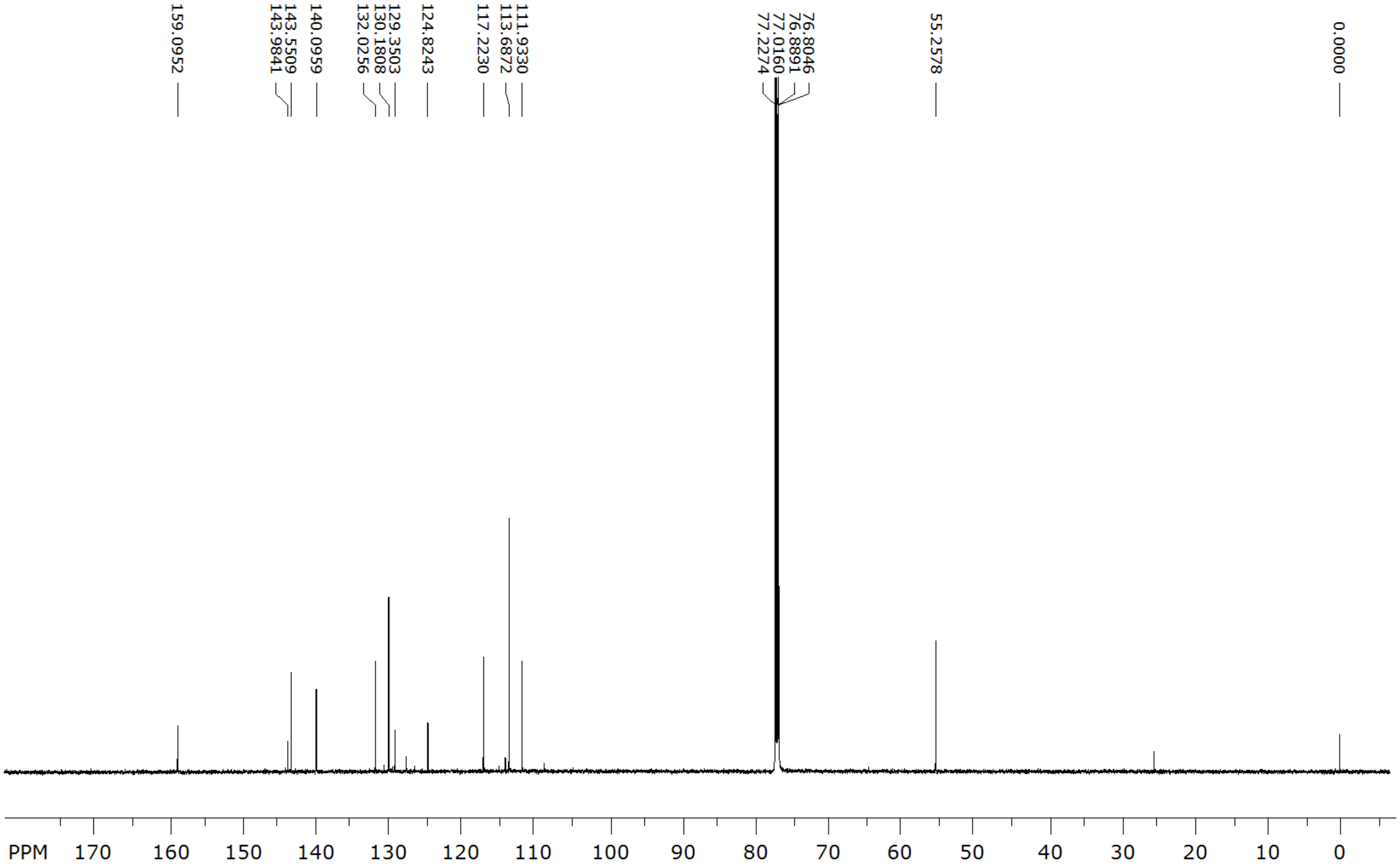

Figure S221. ^{13}C NMR ($CDCl_3$) spectrum of *cis,syn*-**15**.

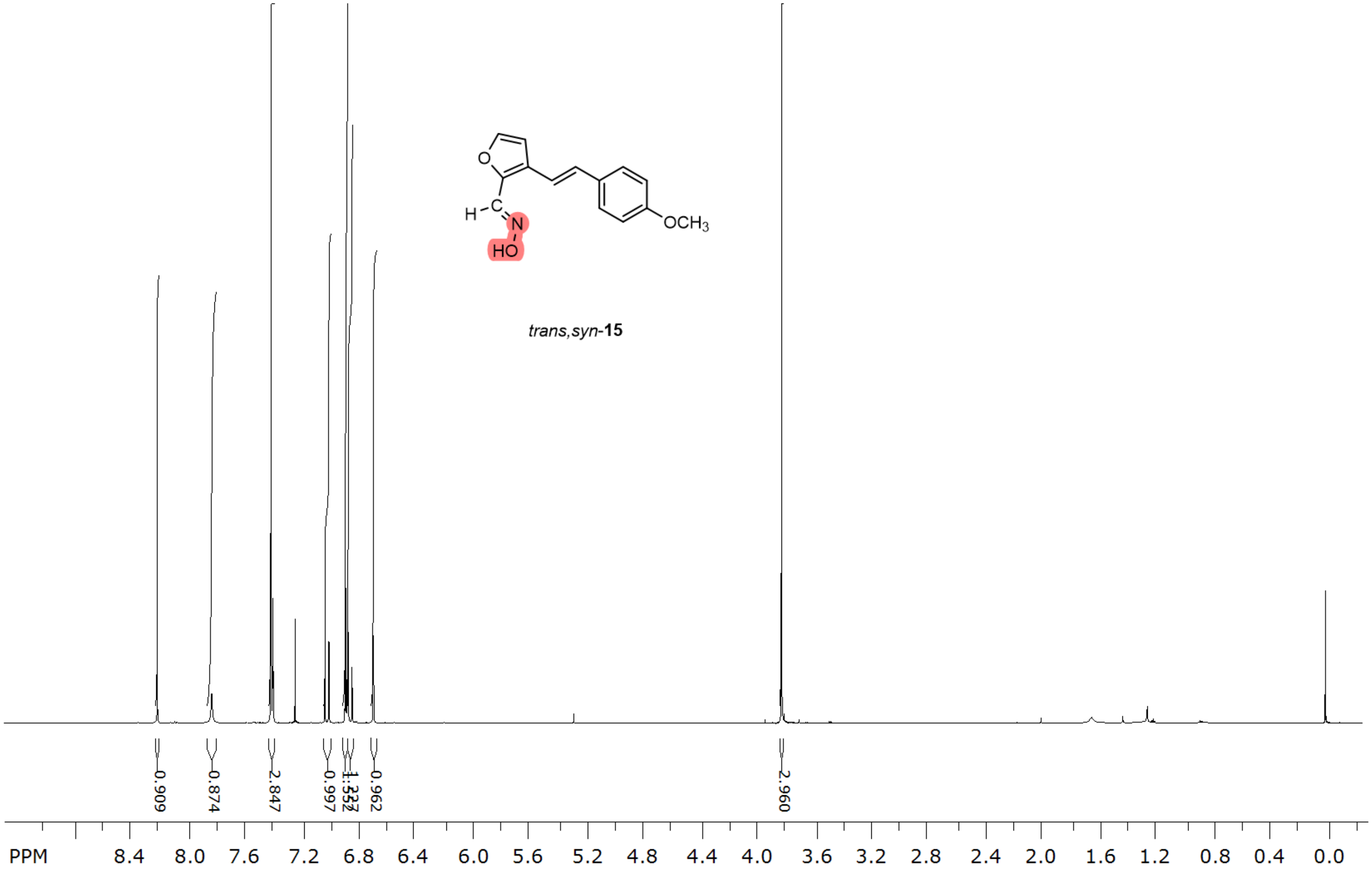

trans,syn-**15**

Figure S222. ^{1}H NMR ($CDCl_3$) spectrum of *trans,syn*-**15**.

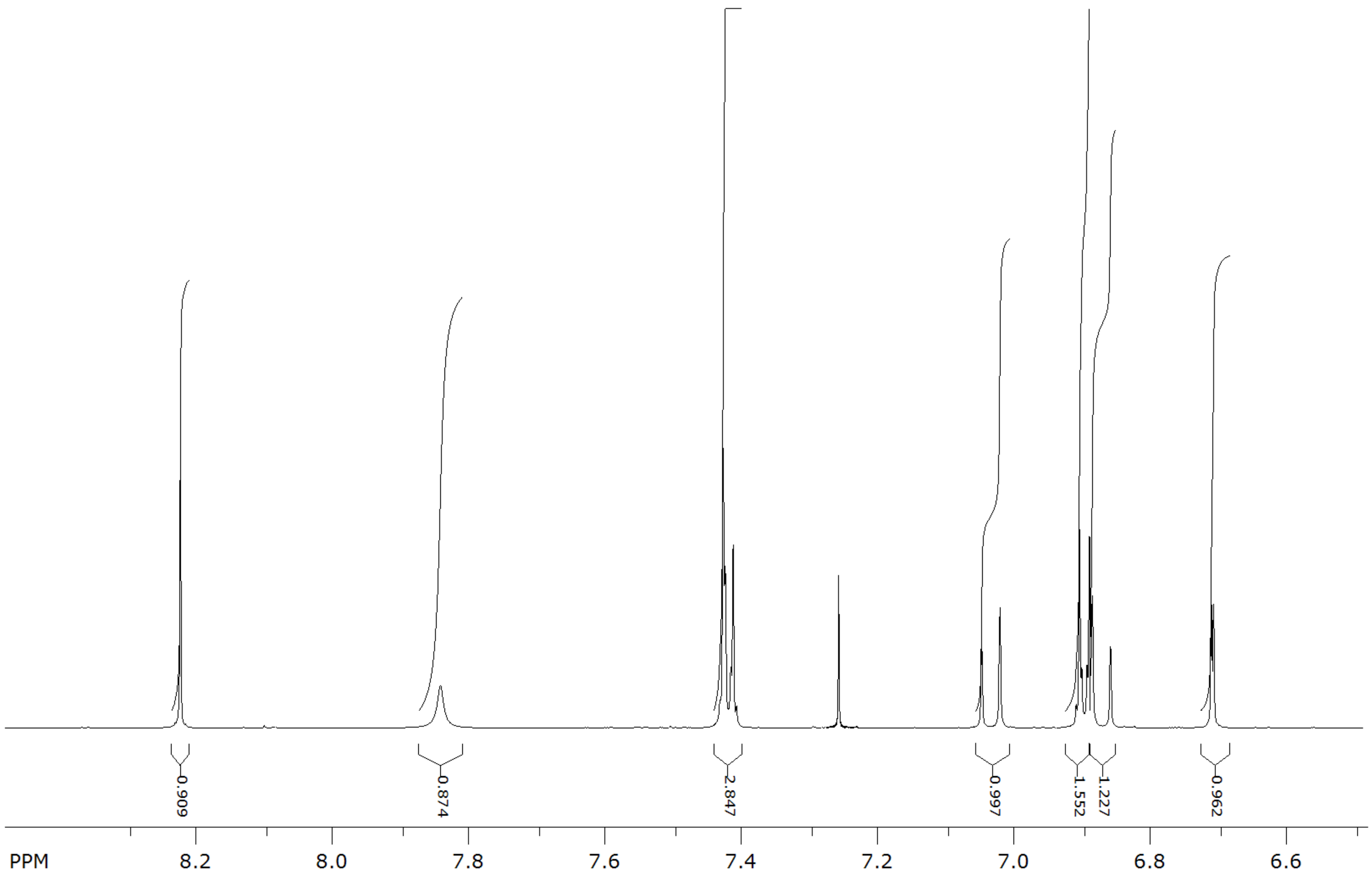

Figure S223. ^{1}H NMR ($CDCl_3$) spectrum of aromatic part of *trans,syn*-**15**.

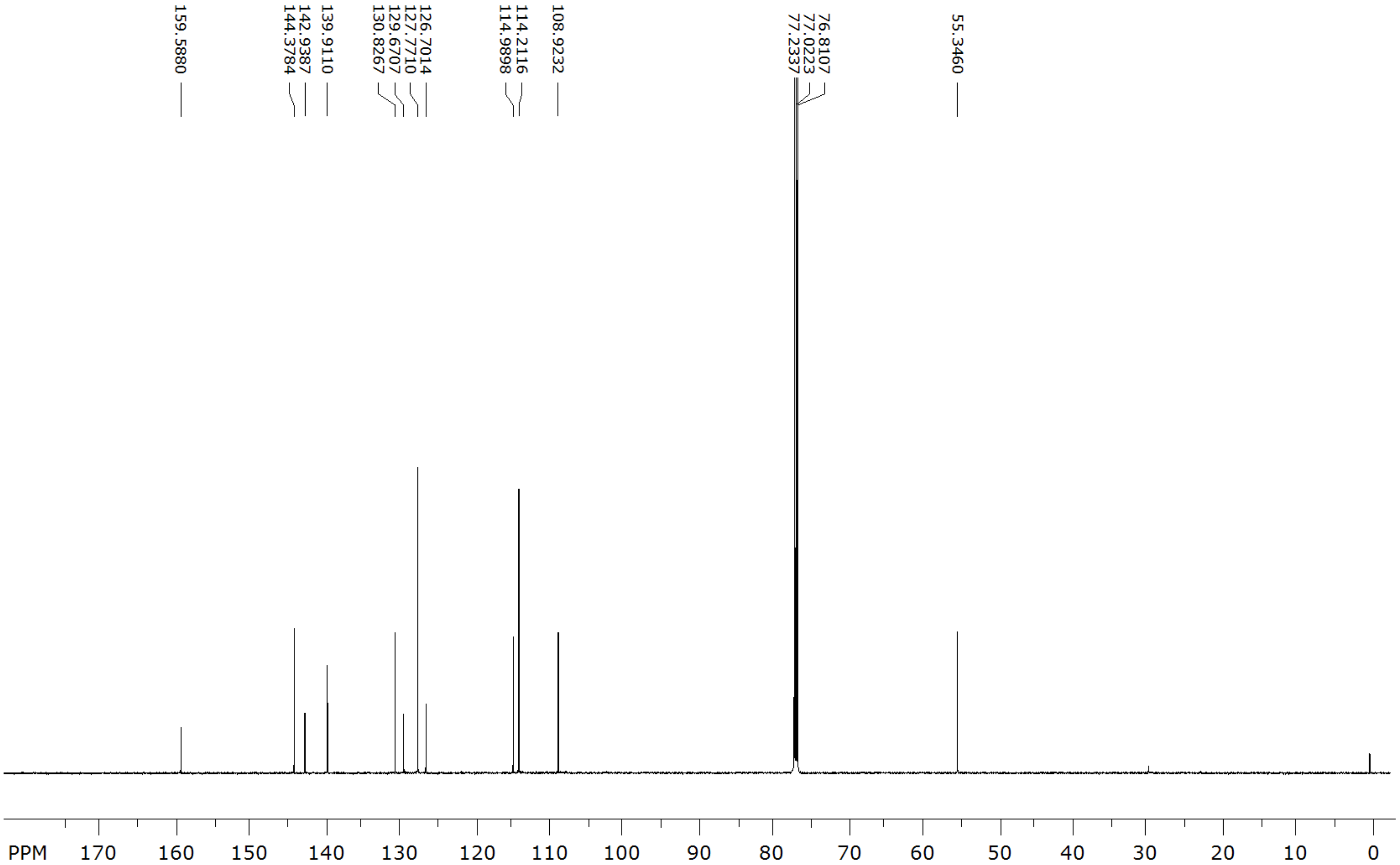

Figure S224. ^{13}C NMR ($CDCl_3$) spectrum of *trans,syn*-**15**.

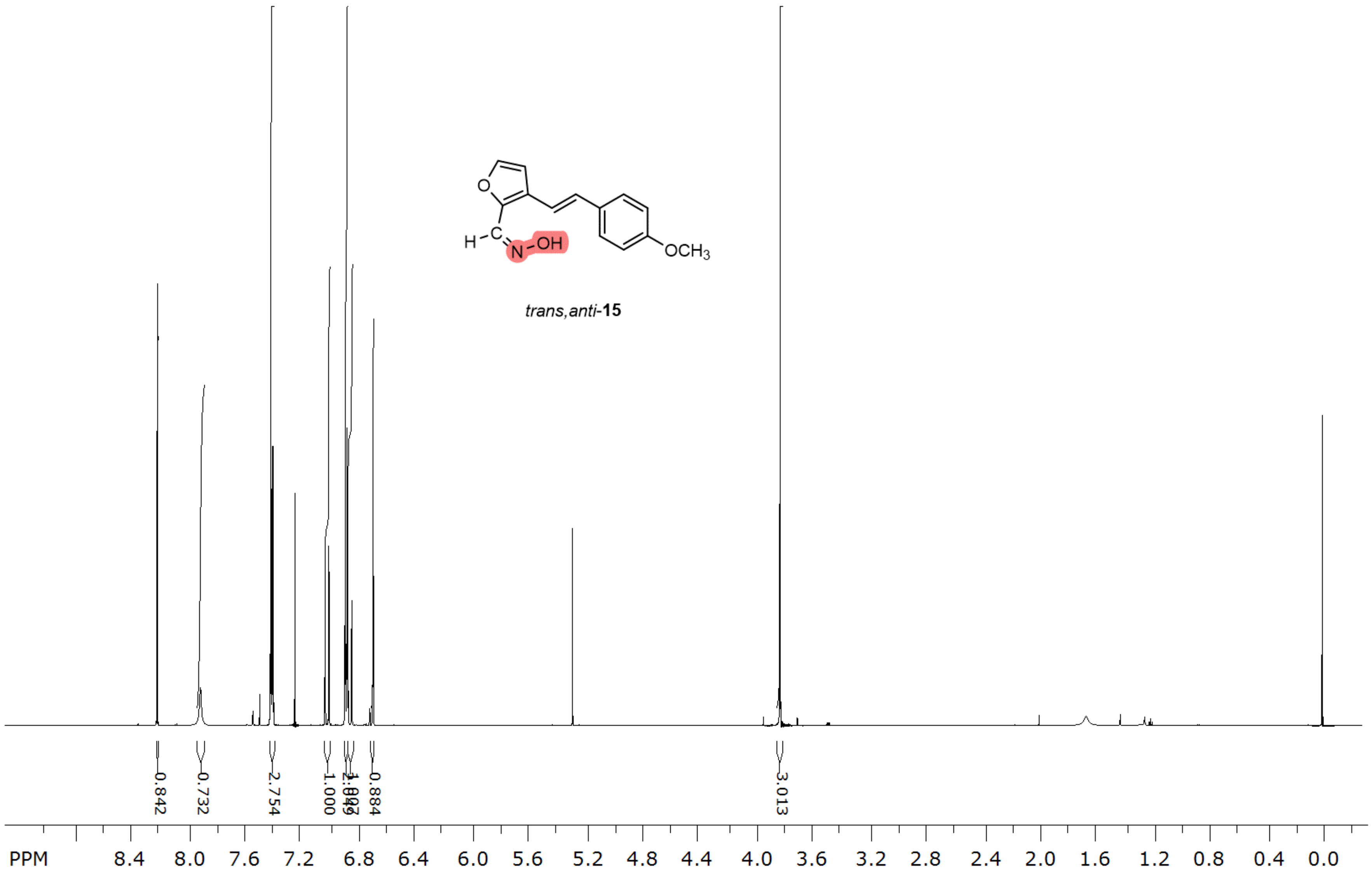

Figure S225. ^{1}H NMR ($CDCl_3$) spectrum of *trans,anti*-**15**.

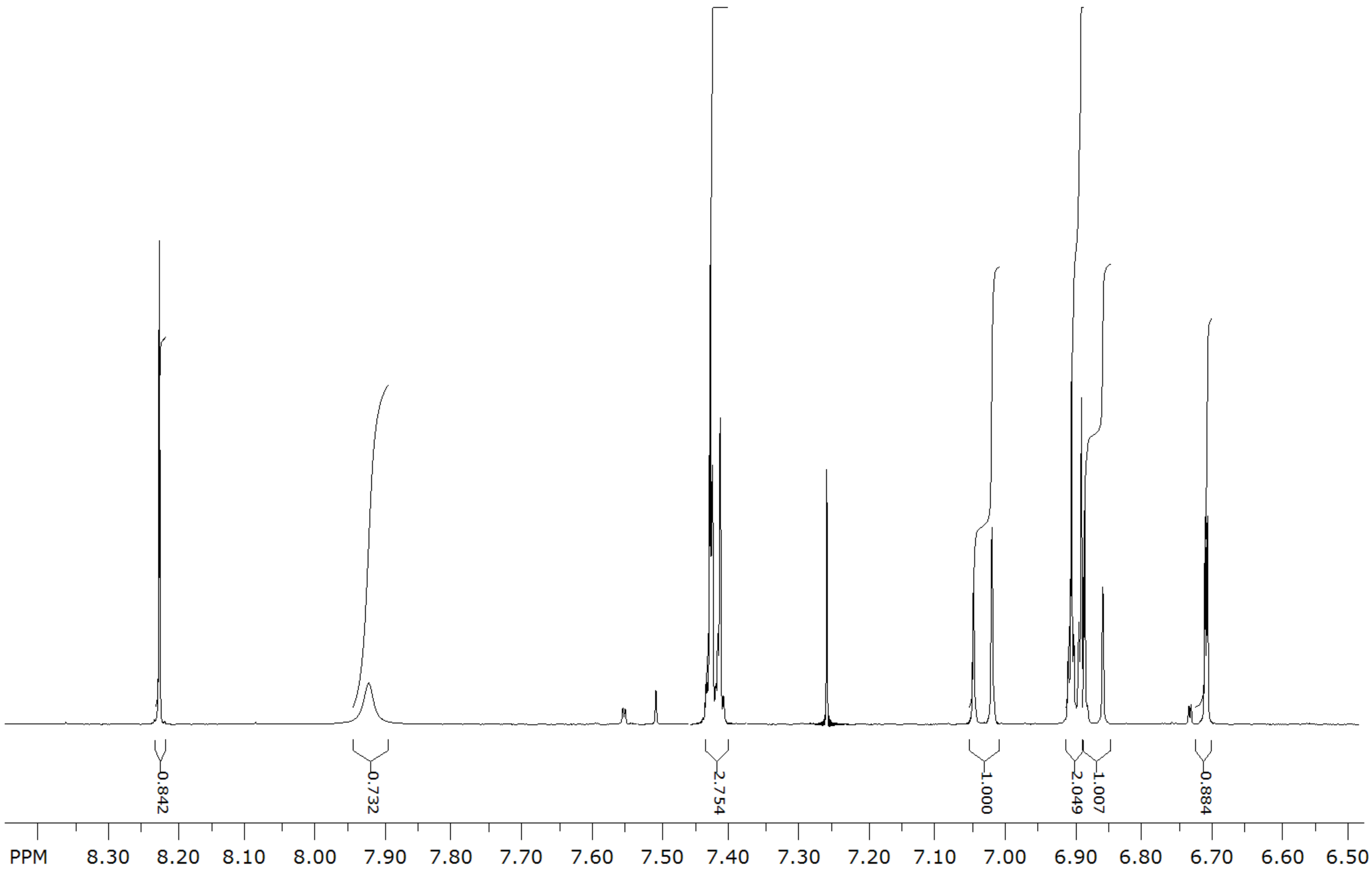

Figure S226. ^{1}H NMR ($CDCl_3$) spectrum of aromatic part of *trans,anti*-**15**.

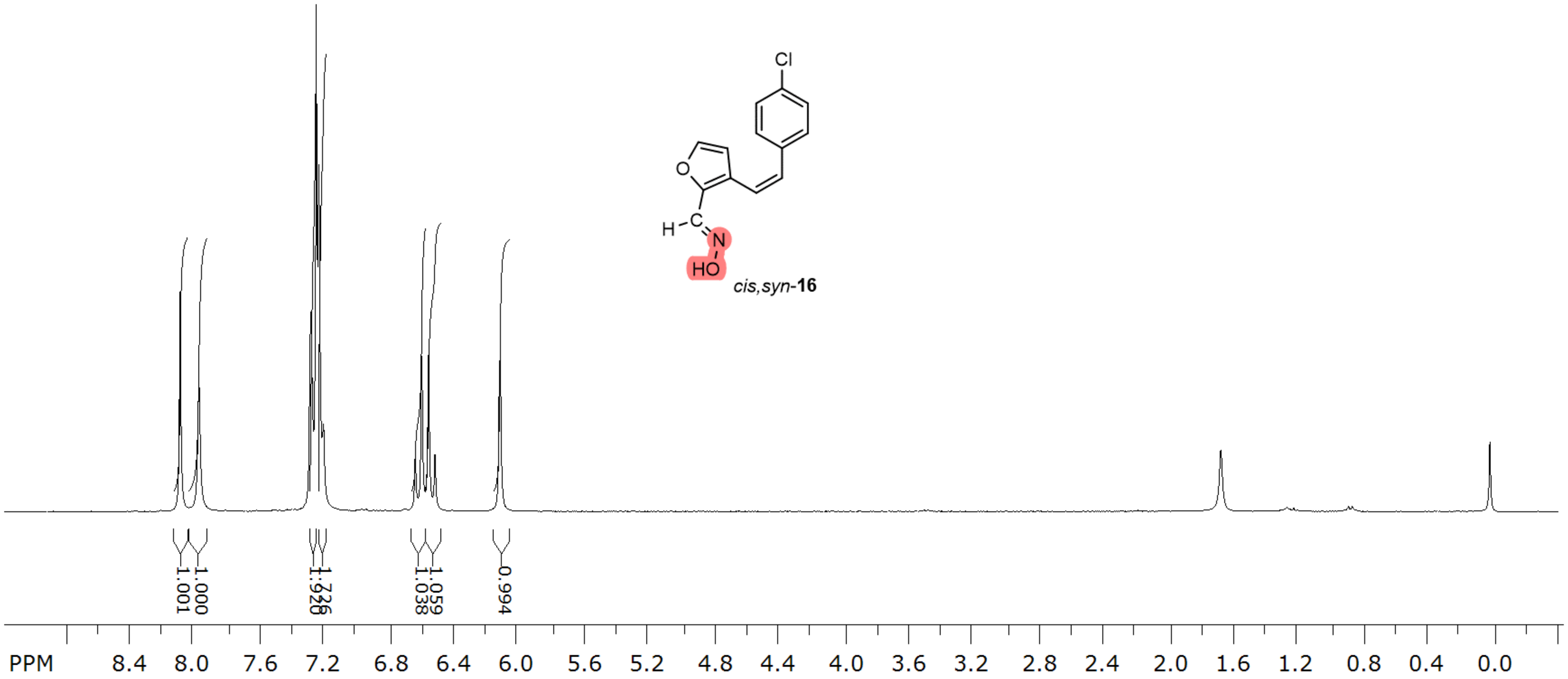

Figure S227. ^{1}H NMR ($CDCl_3$) spectrum of *cis,syn*-**16**.

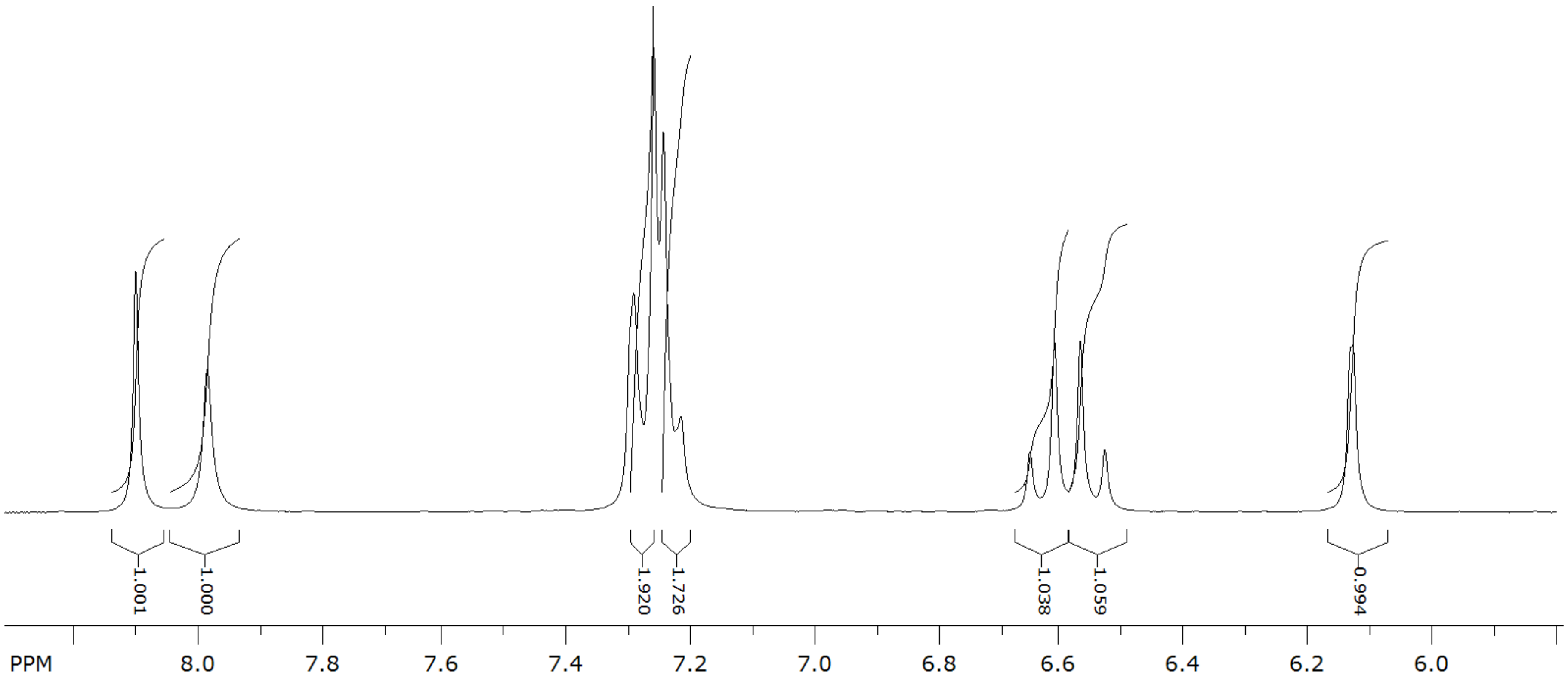

Figure S228. ^{1}H NMR ($CDCl_3$) spectrum of aromatic part of *cis,syn*-**16**.

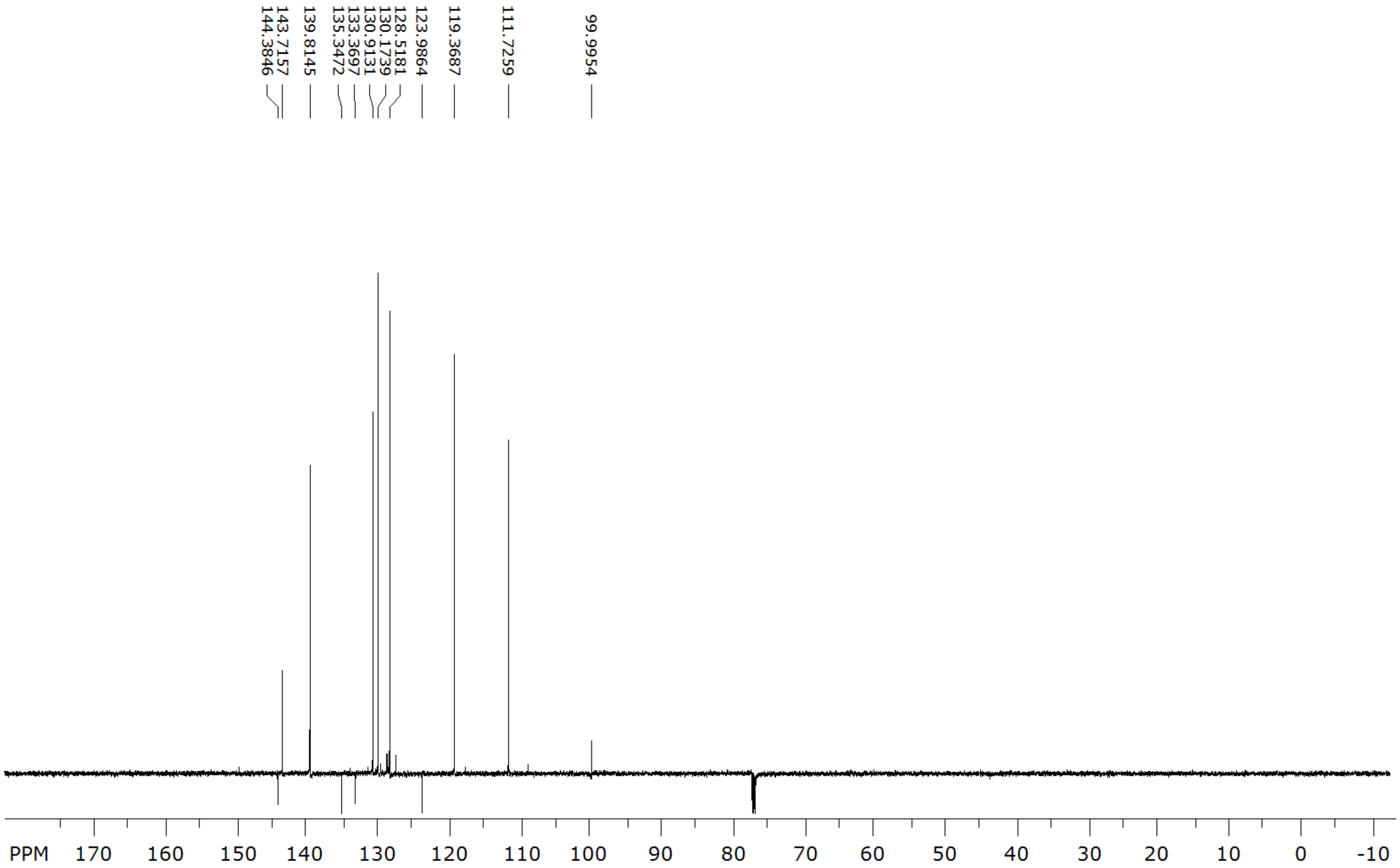

Figure S229. ^{13}C NMR ($CDCl_3$) spectrum of *cis,syn*-**16**.

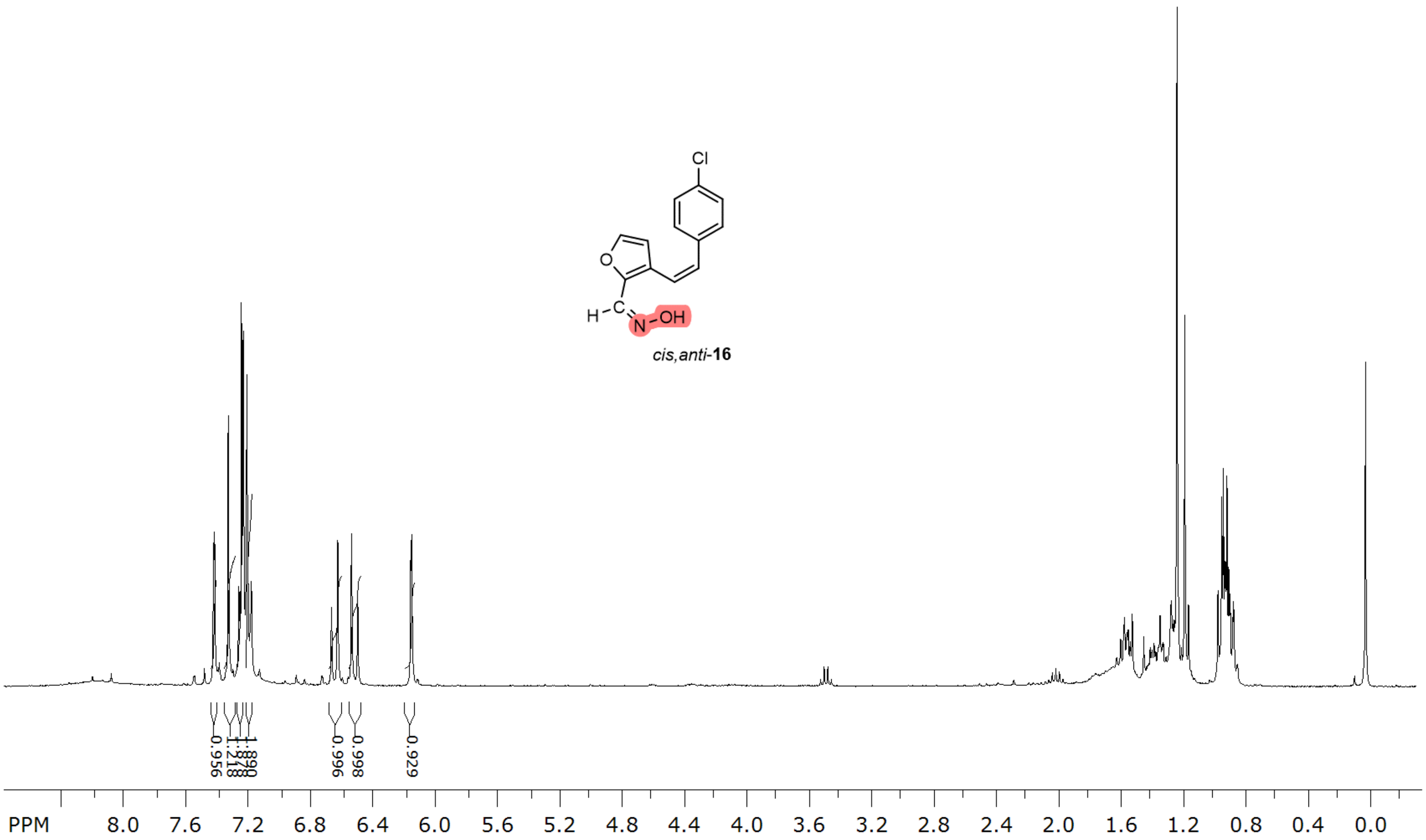

Figure S230. ^{1}H NMR ($CDCl_3$) spectrum of *cis,anti*-**16**.

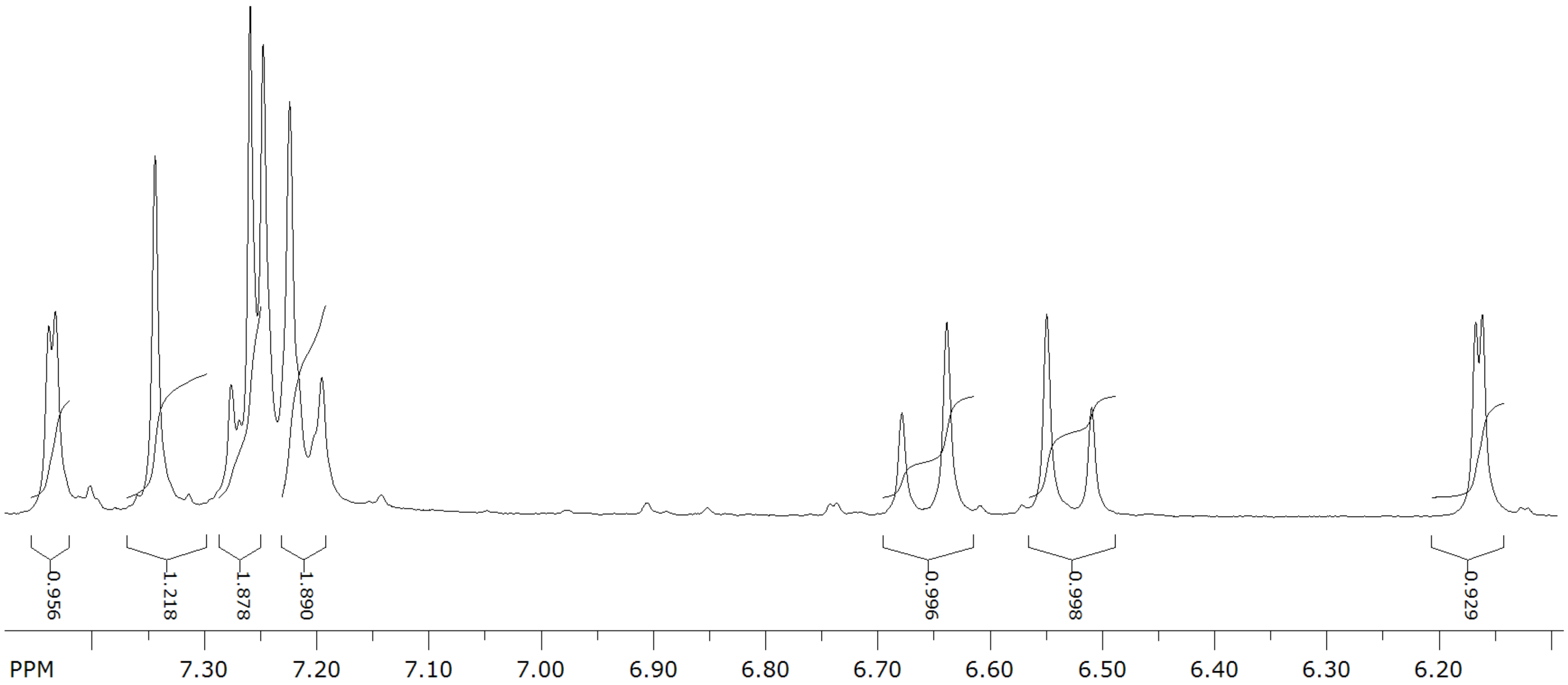

Figure S231. ^{1}H NMR ($CDCl_3$) spectrum of aromatic part of *cis,anti*-**16**.

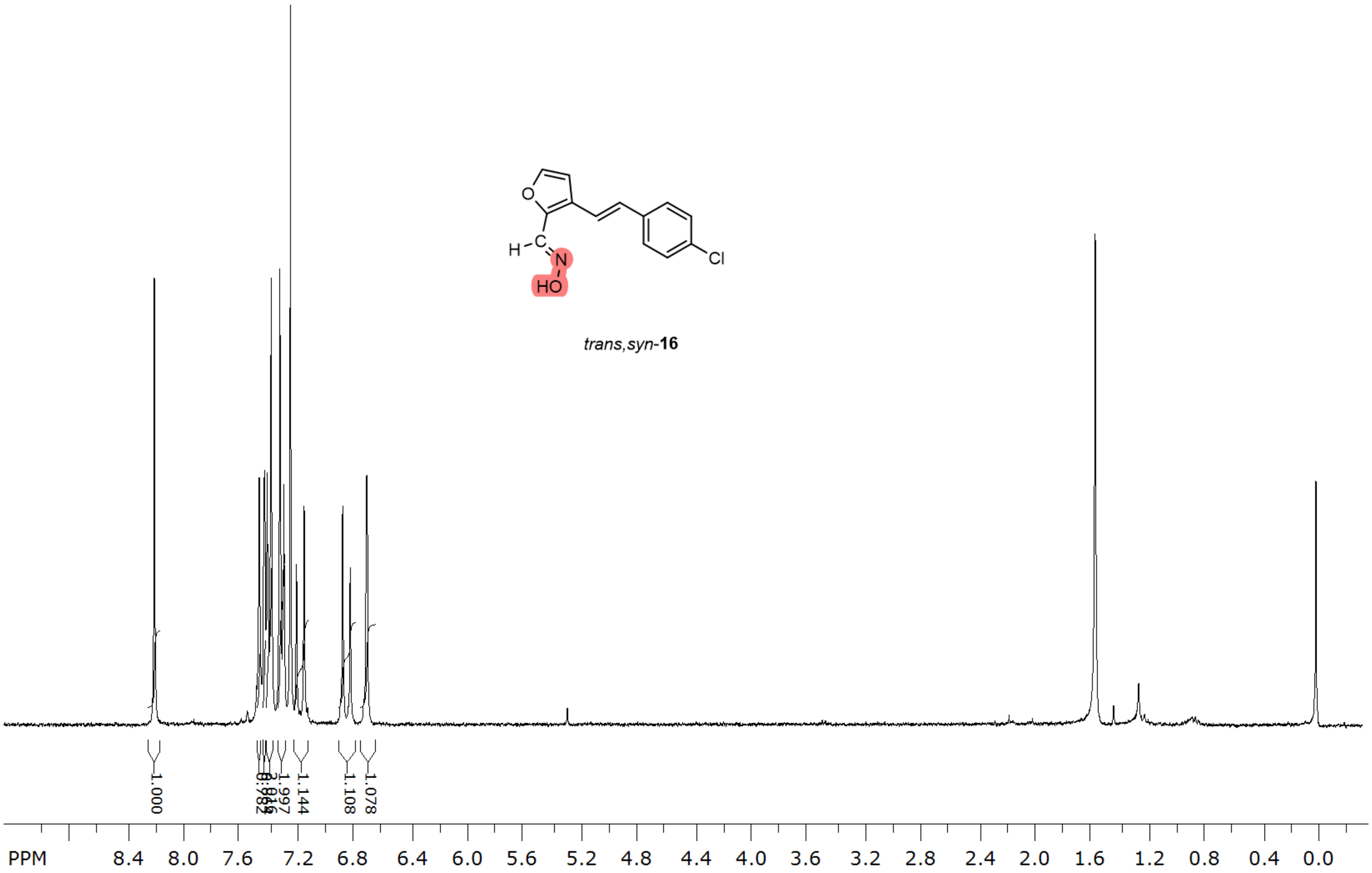

Figure S232. ^{1}H NMR ($CDCl_3$) spectrum of *trans,syn*-**16**.

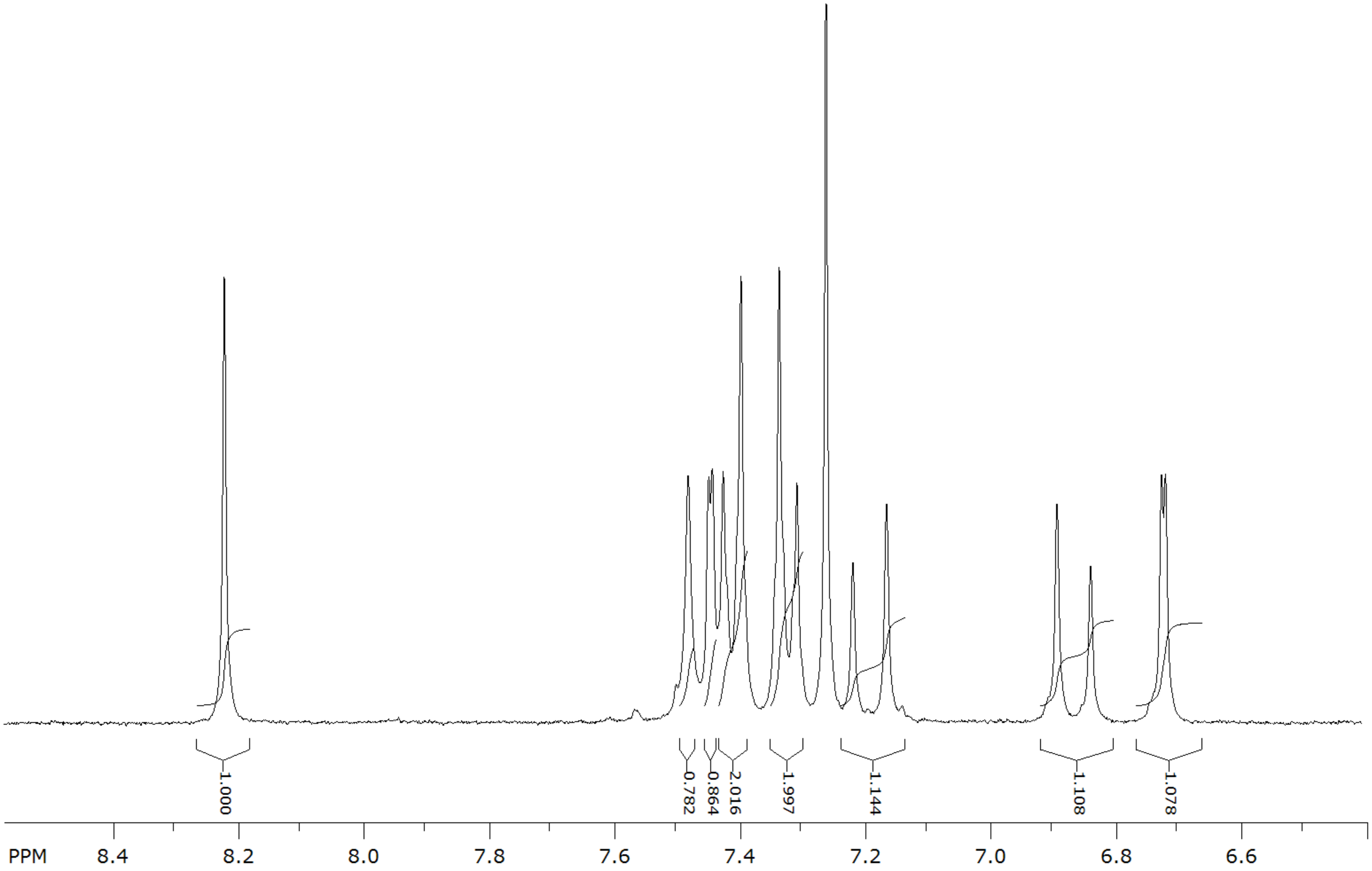

Figure S233. ^{1}H NMR ($CDCl_3$) spectrum of aromatic part of *trans,syn*-**16**.

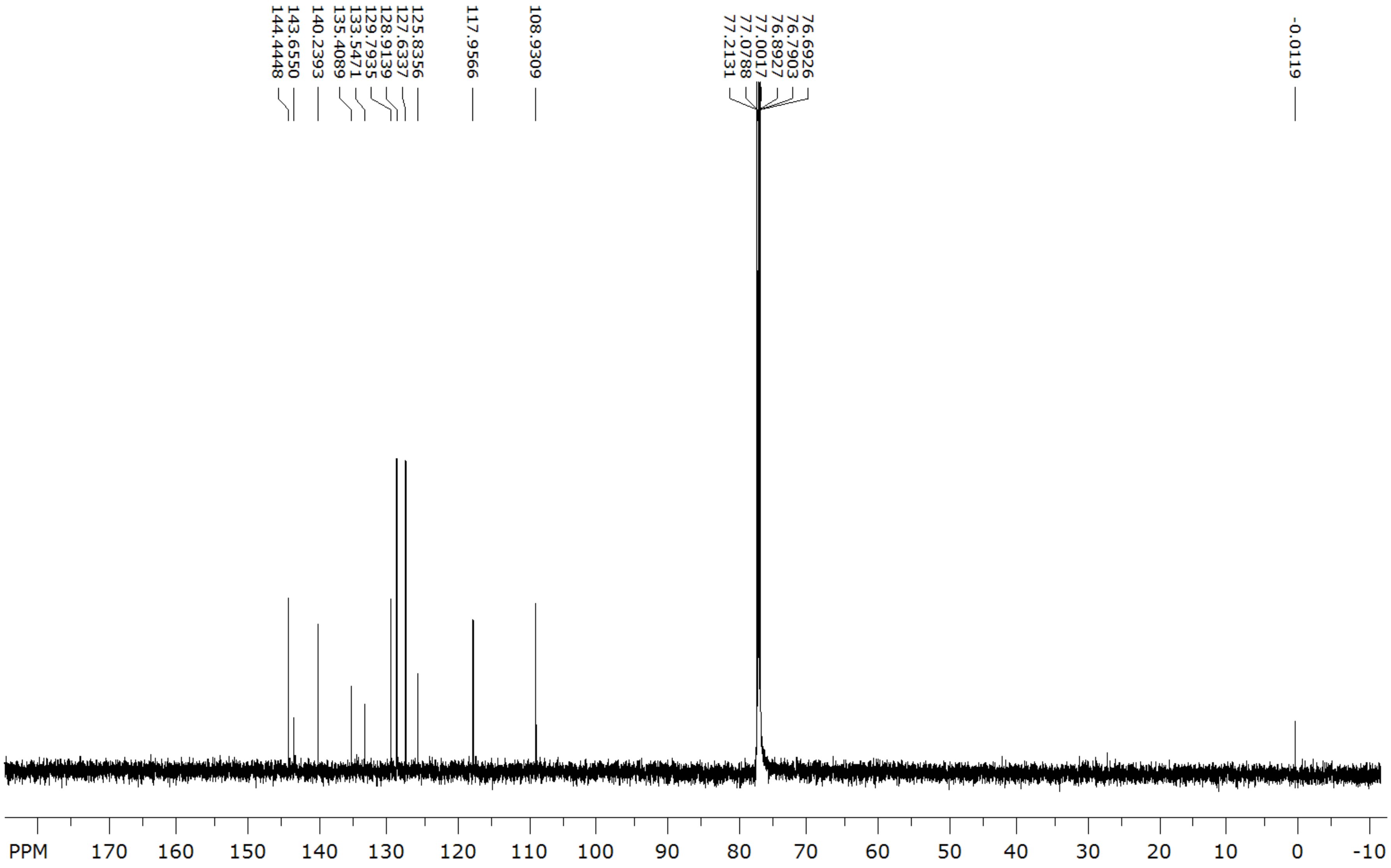

Figure S234. ^{13}C NMR ($CDCl_3$) spectrum of *trans,syn*-**16**.

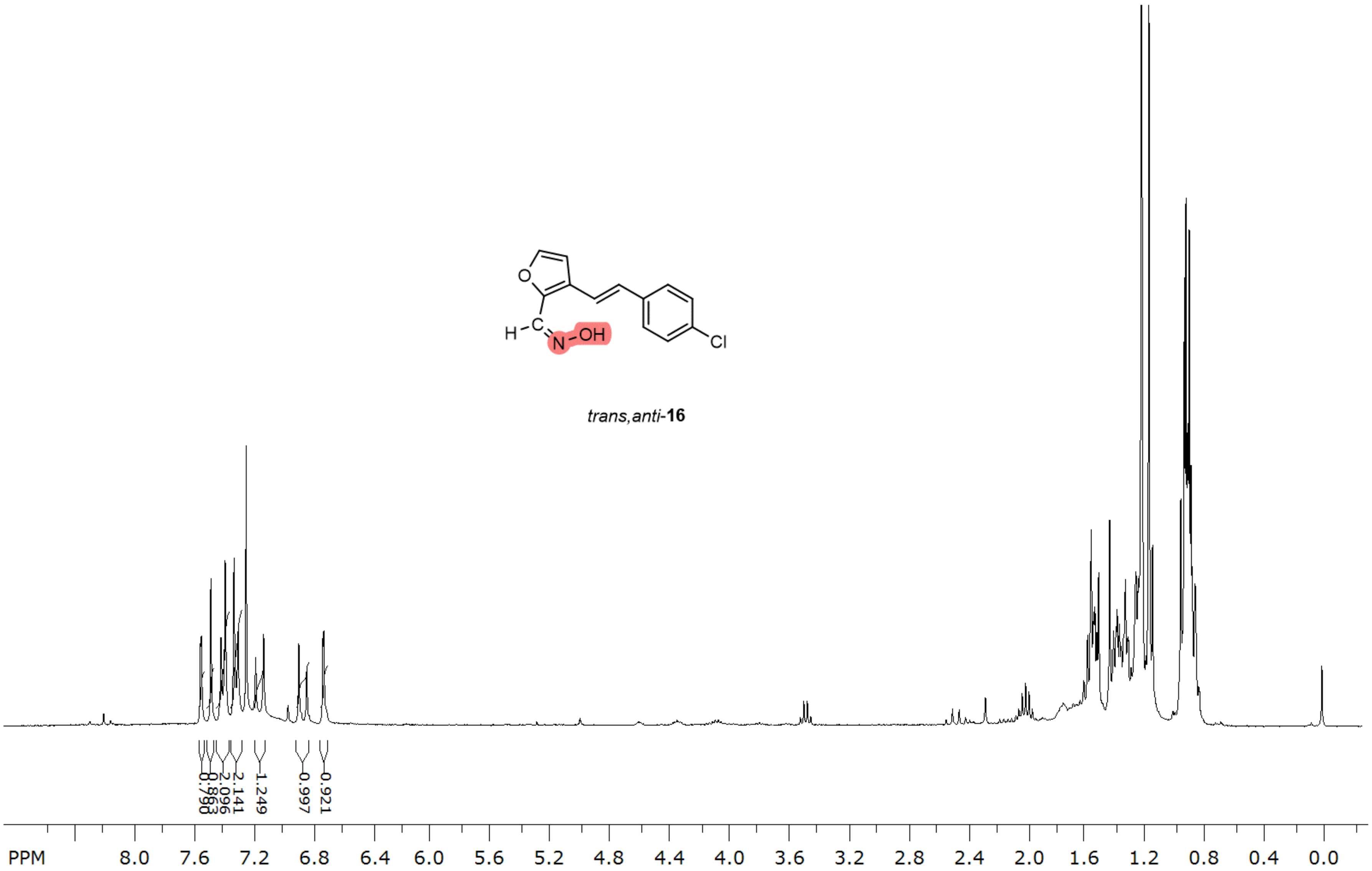

Figure S235. ^{1}H NMR ($CDCl_3$) spectrum of *trans,anti*-**16**.

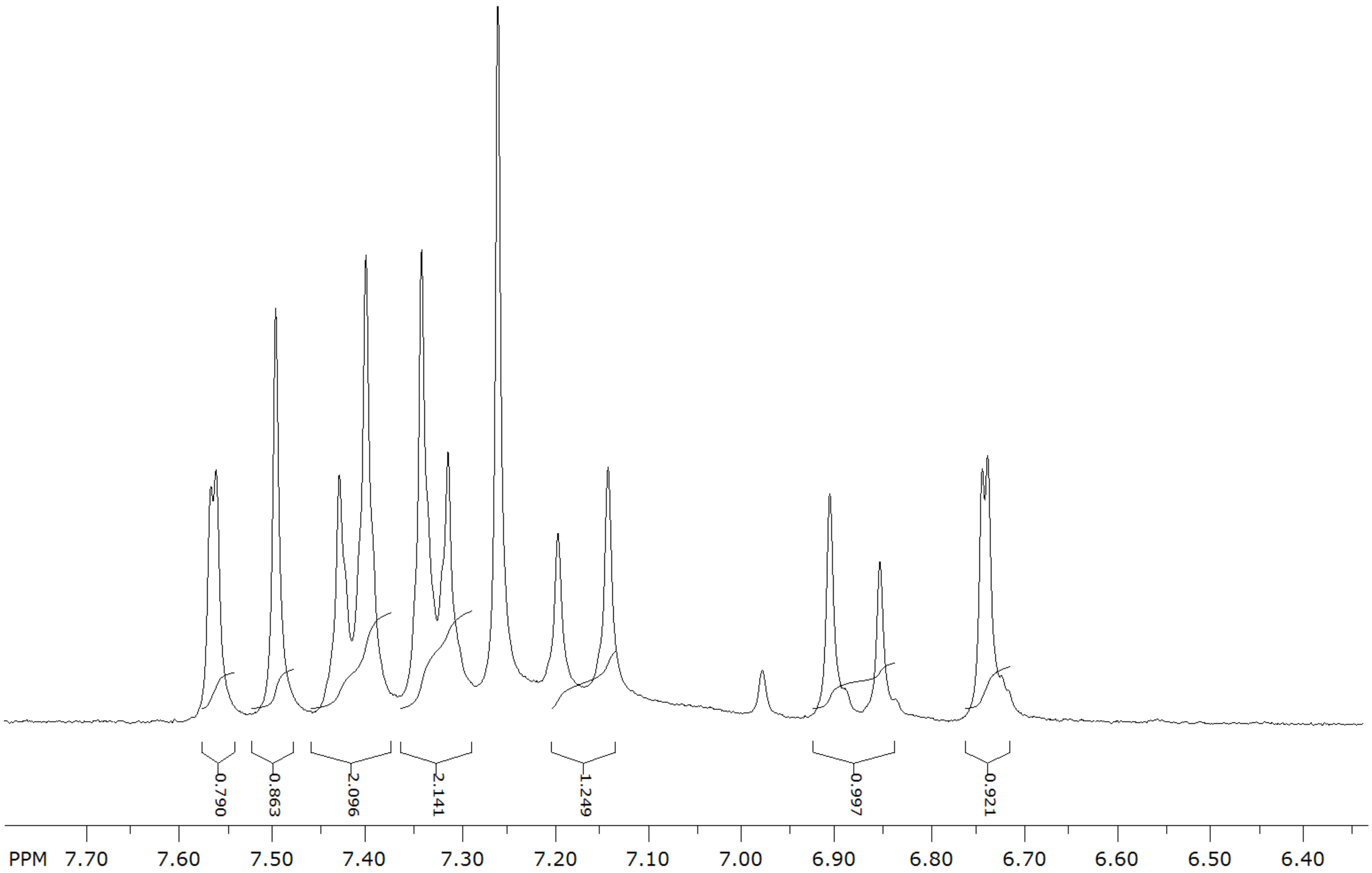

Figure S236. ^{1}H NMR ($CDCl_3$) spectrum of aromatic part of *trans,anti*-**16**.

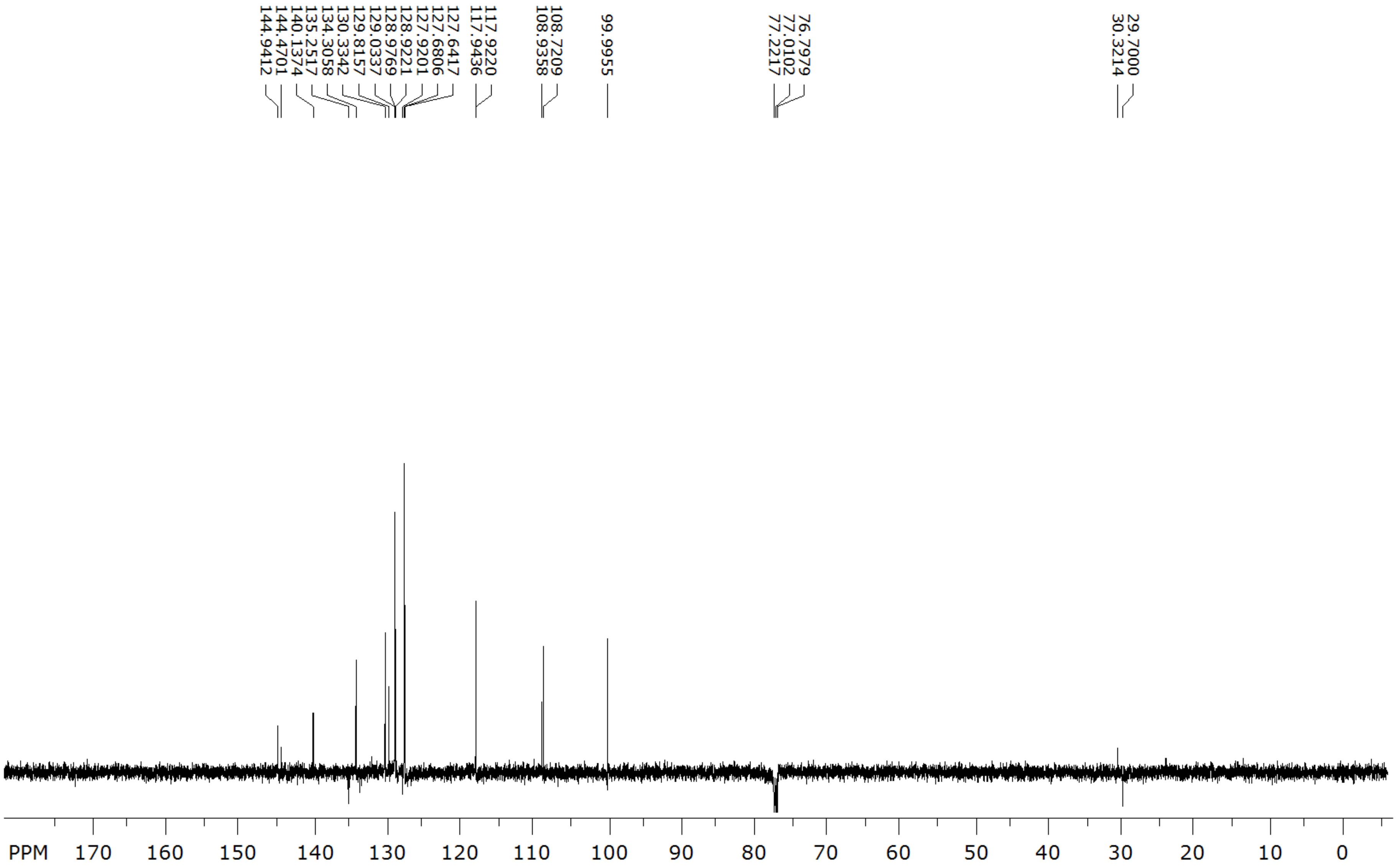

Figure S237. ^{13}C NMR ($CDCl_3$) spectrum of *trans,anti*-**16**.

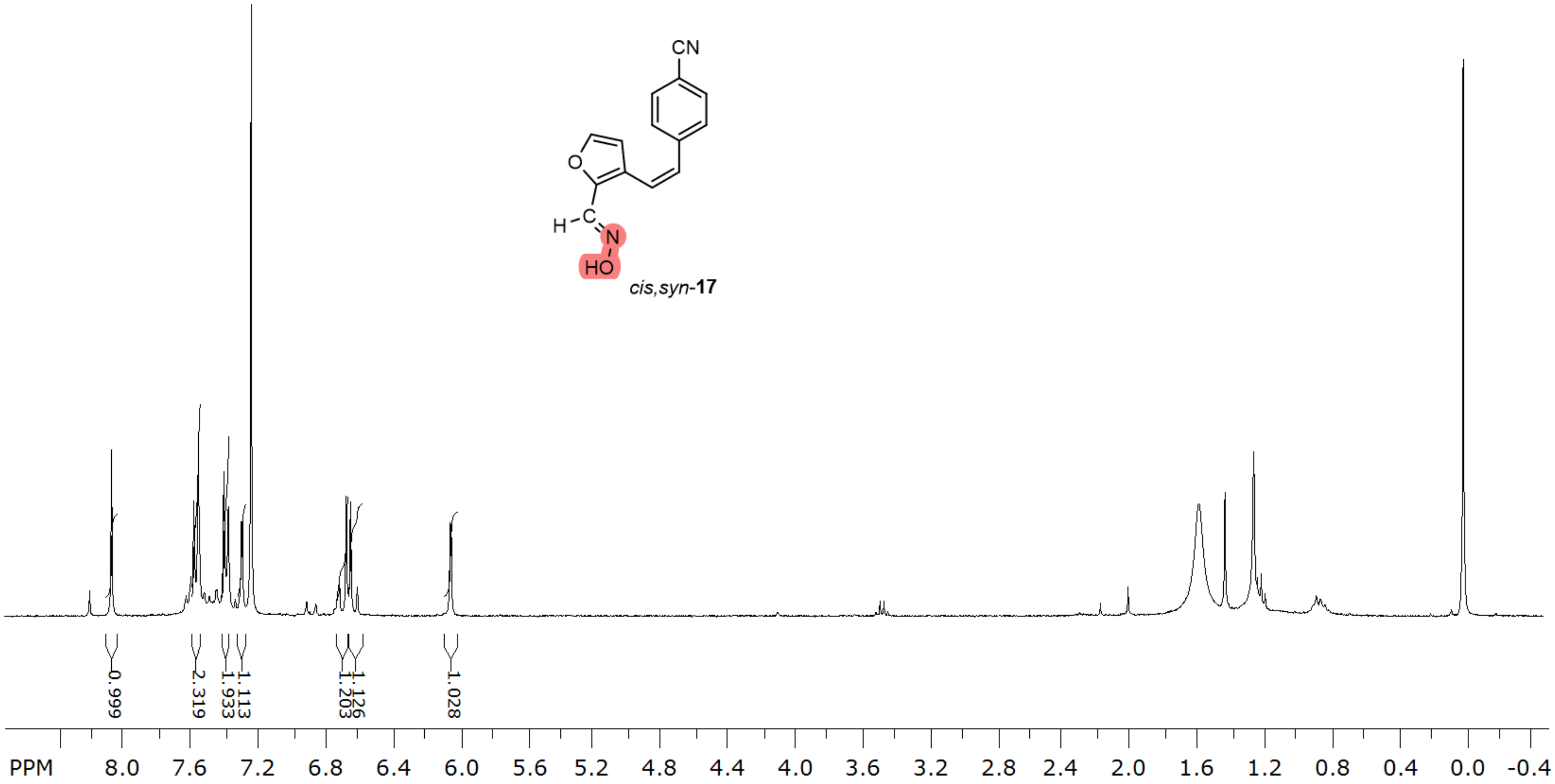

Figure S238. ^{1}H NMR ($CDCl_3$) spectrum of *cis,syn*-**17**.

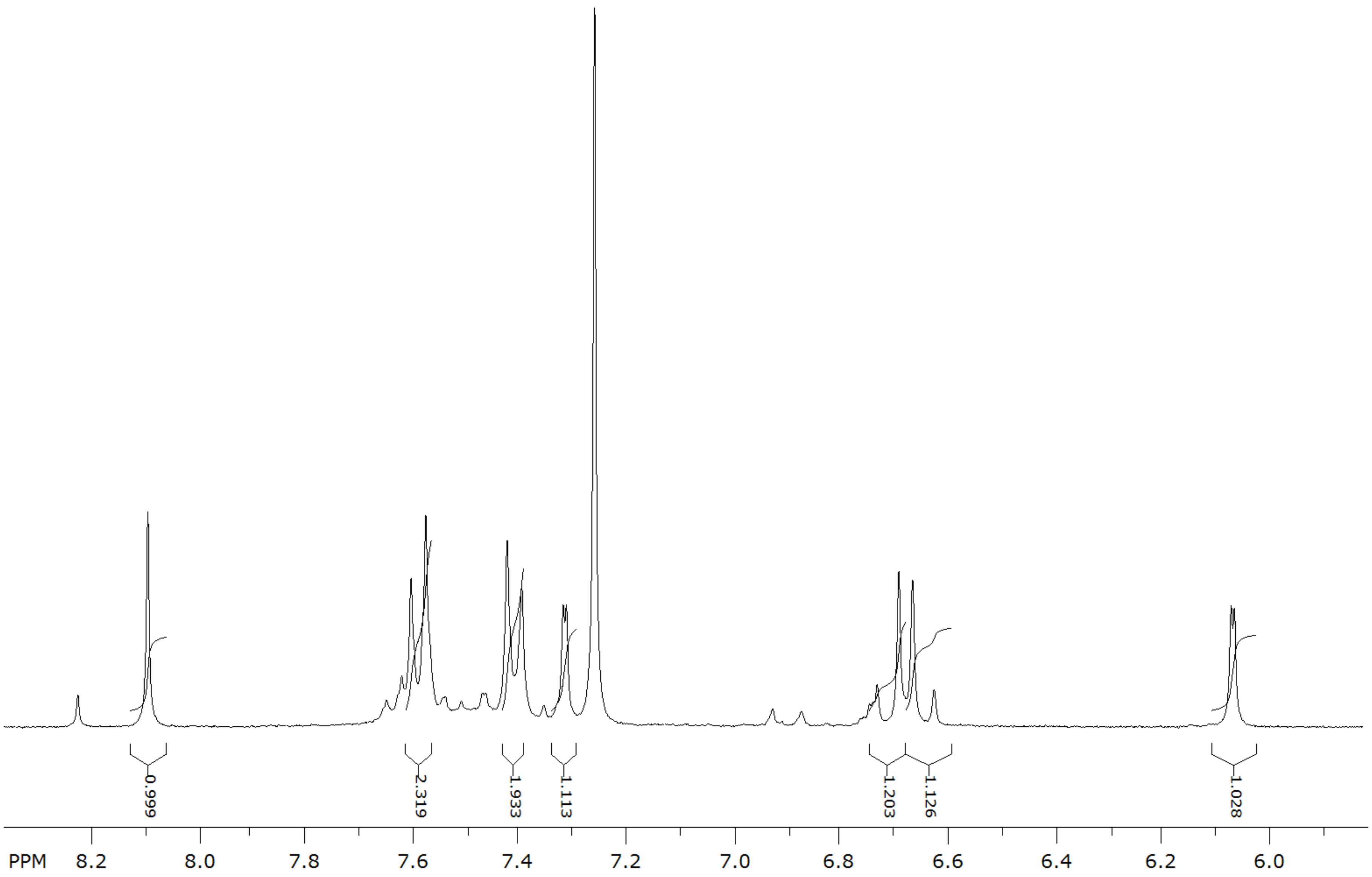

Figure S239. ^{1}H NMR ($CDCl_3$) spectrum of aromatic part of *cis,syn*-**17**.

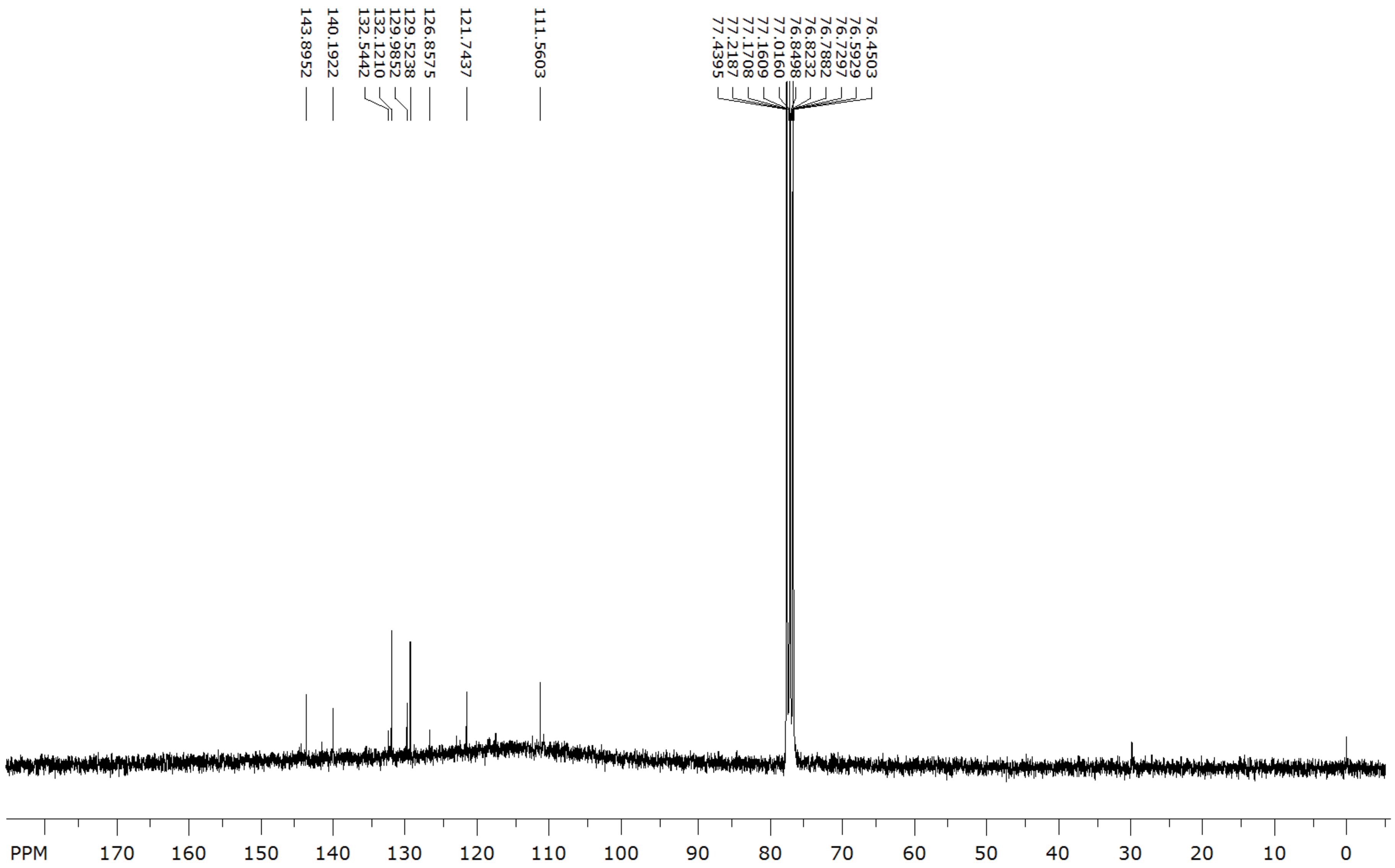

Figure S240. ^{13}C NMR ($CDCl_3$) spectrum of *cis,syn*-**17**.

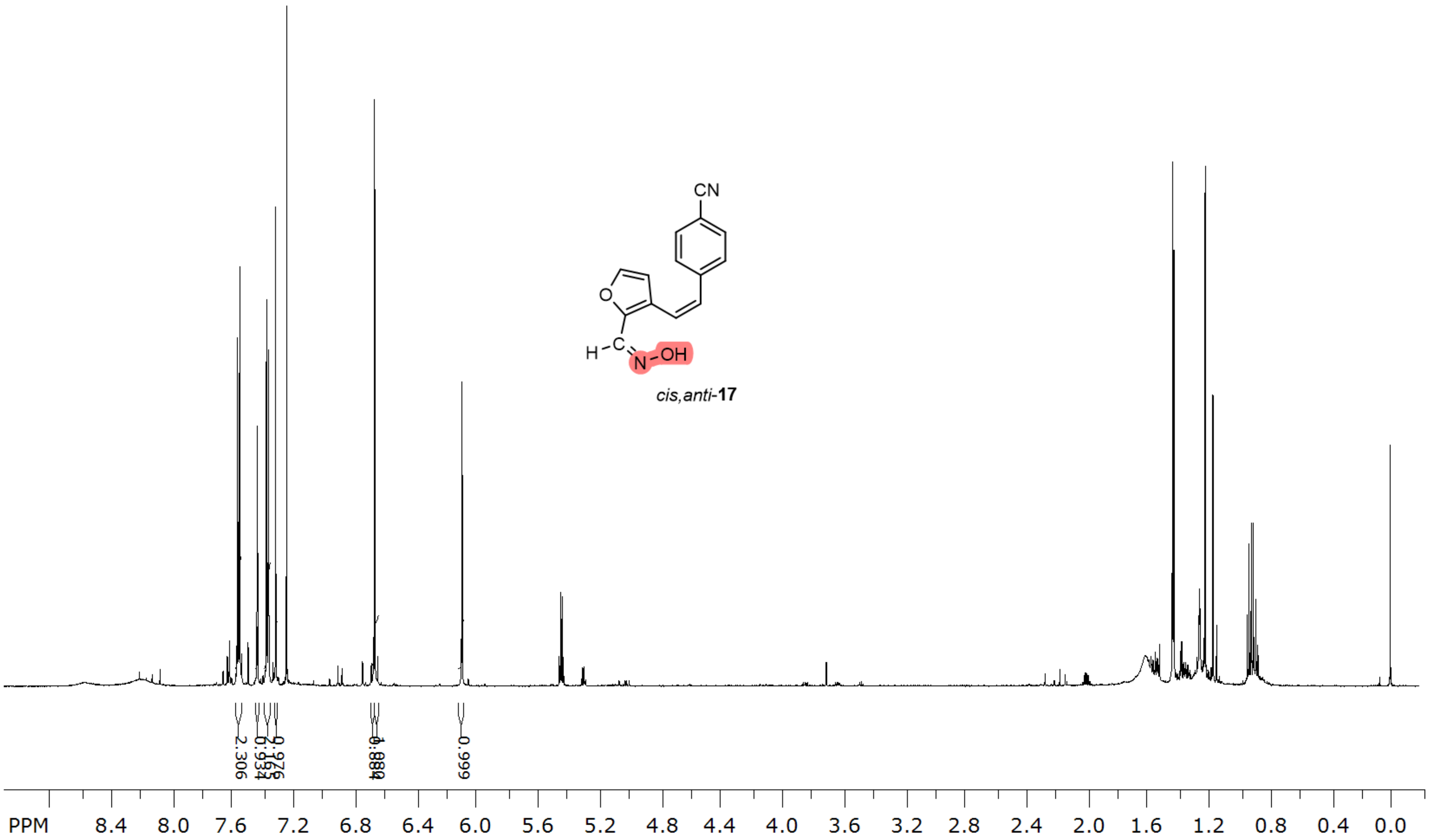

Figure S241. ^{1}H NMR ($CDCl_3$) spectrum of *cis,anti*-**17**.

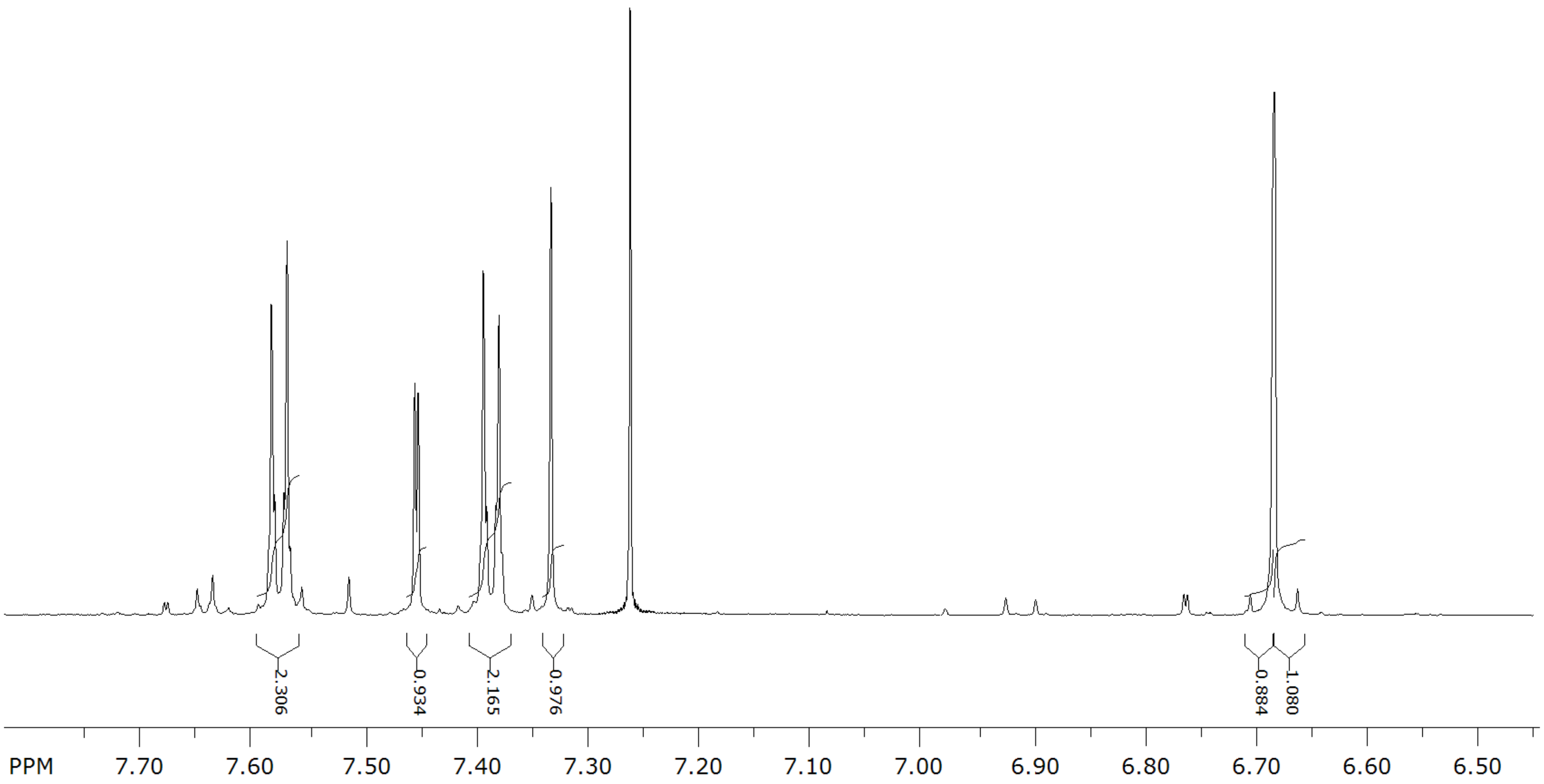

Figure S242. ^{1}H NMR ($CDCl_3$) spectrum of aromatic part of *cis,anti*-**17**.

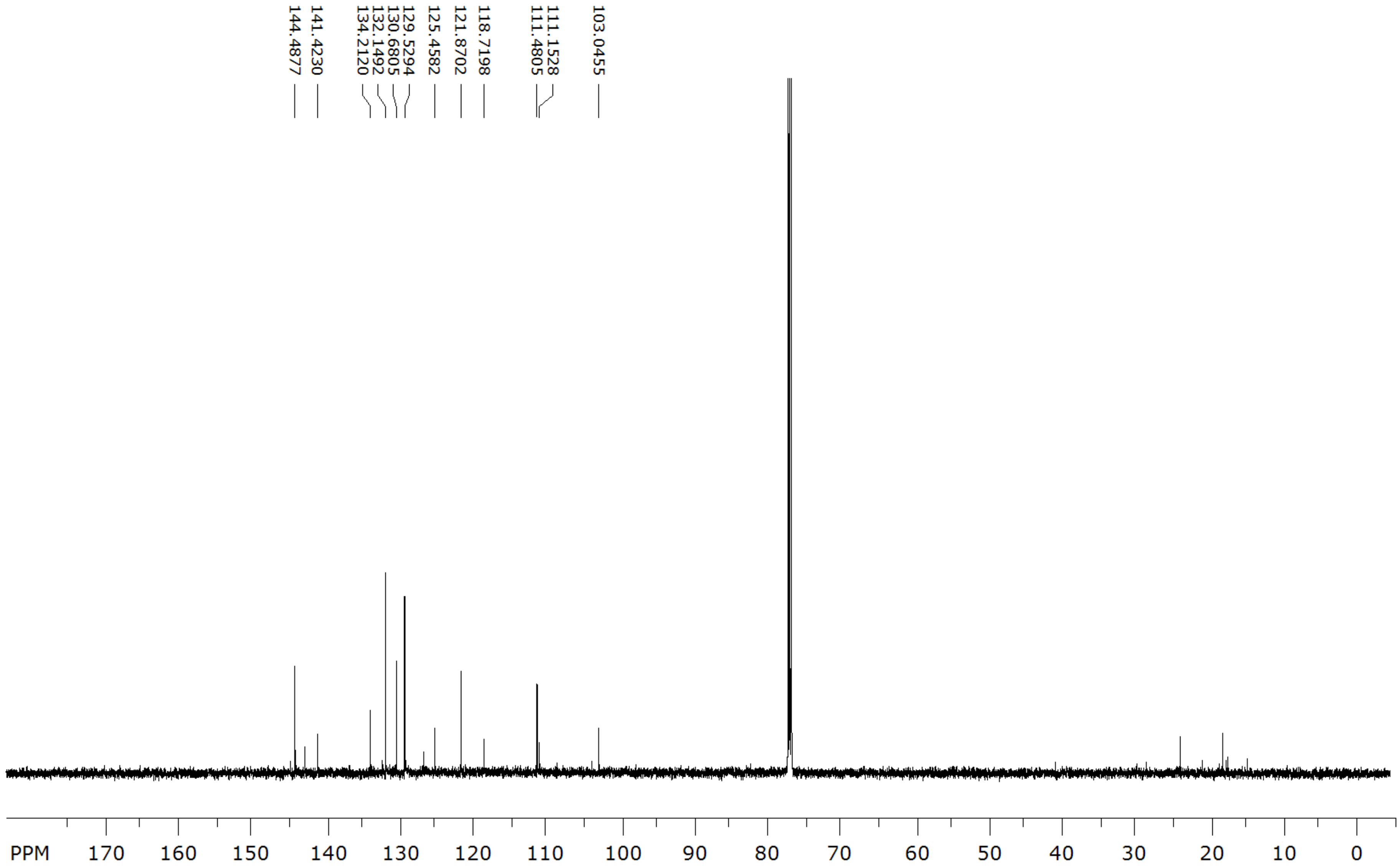

Figure S243. ^{13}C NMR ($CDCl_3$) spectrum of *cis,anti*-**17**.

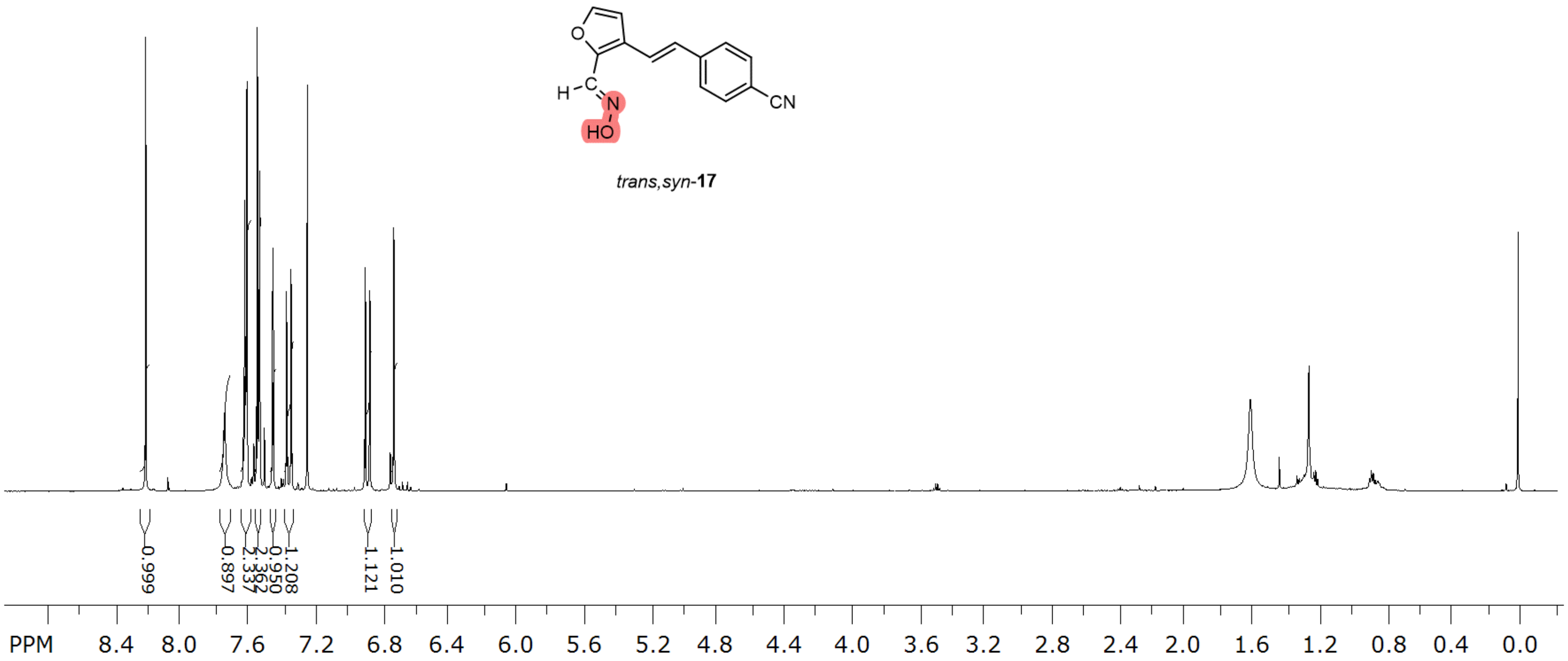

Figure S244. ^{1}H NMR ($CDCl_3$) spectrum of *trans,syn*-**17**.

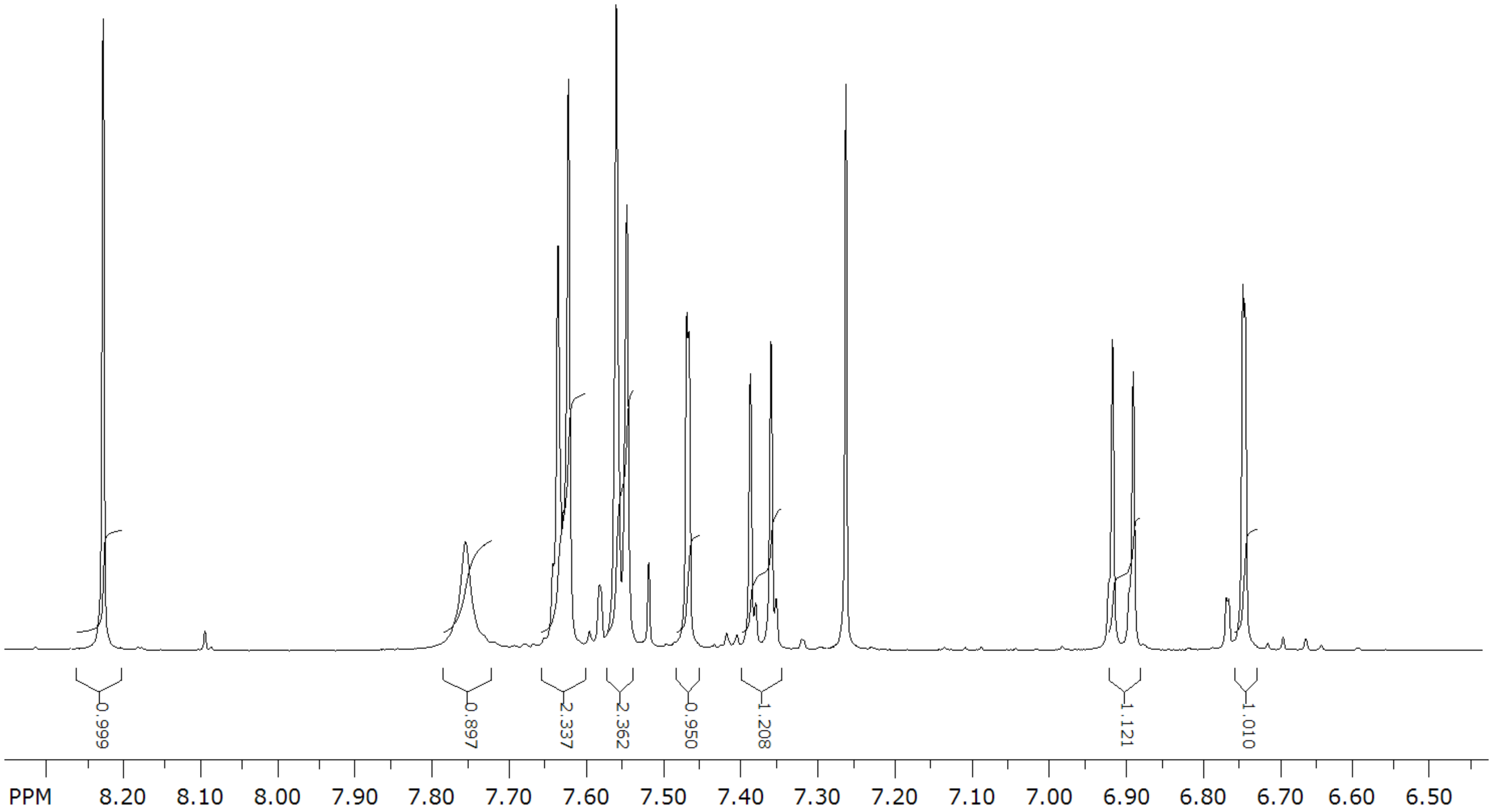

Figure S245. ^{1}H NMR ($CDCl_3$) spectrum of aromatic part of *trans,syn*-**17**.

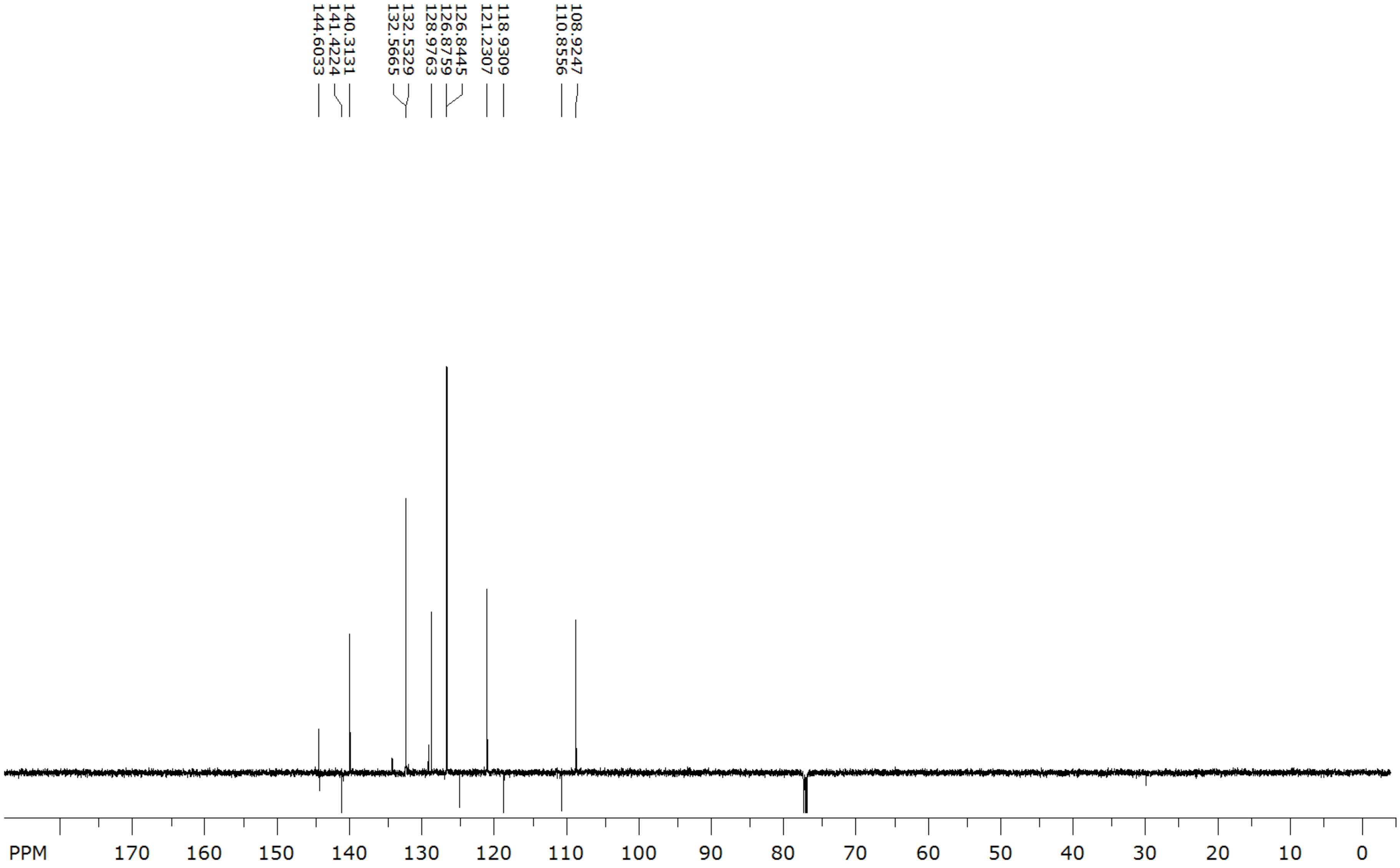

Figure S246. ^{13}C NMR ($CDCl_3$) spectrum of *trans,syn*-**17**.

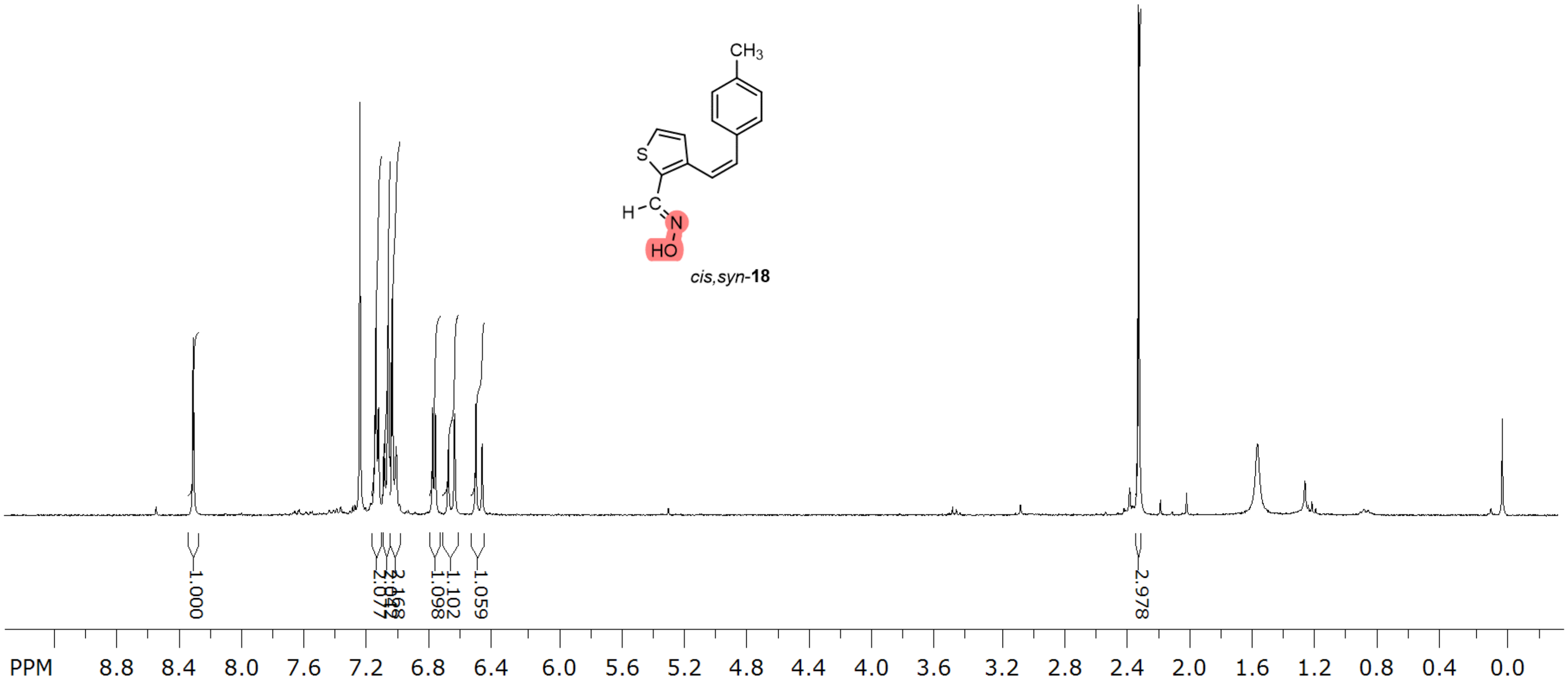

Figure S247. ^{1}H NMR ($CDCl_3$) spectrum of *cis,syn*-**18**.

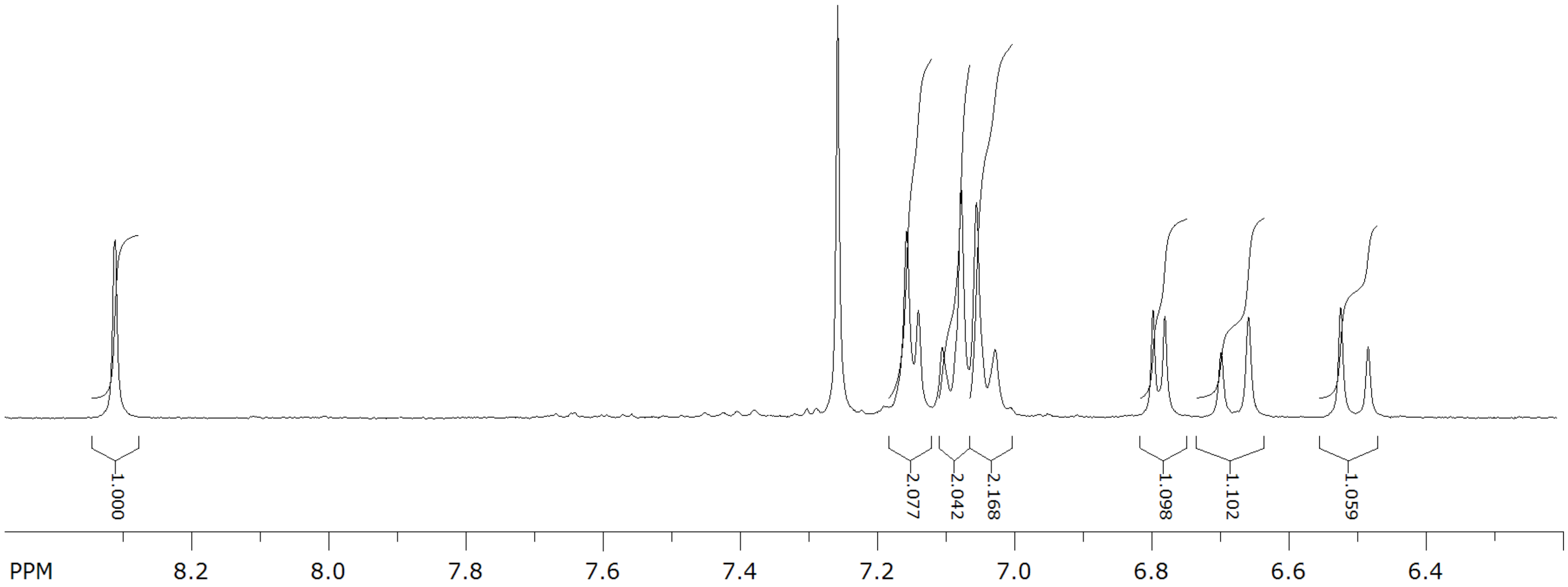

Figure S248. ^1H NMR ($CDCl_3$) spectrum of aromatic part of *cis,syn*-**18**.

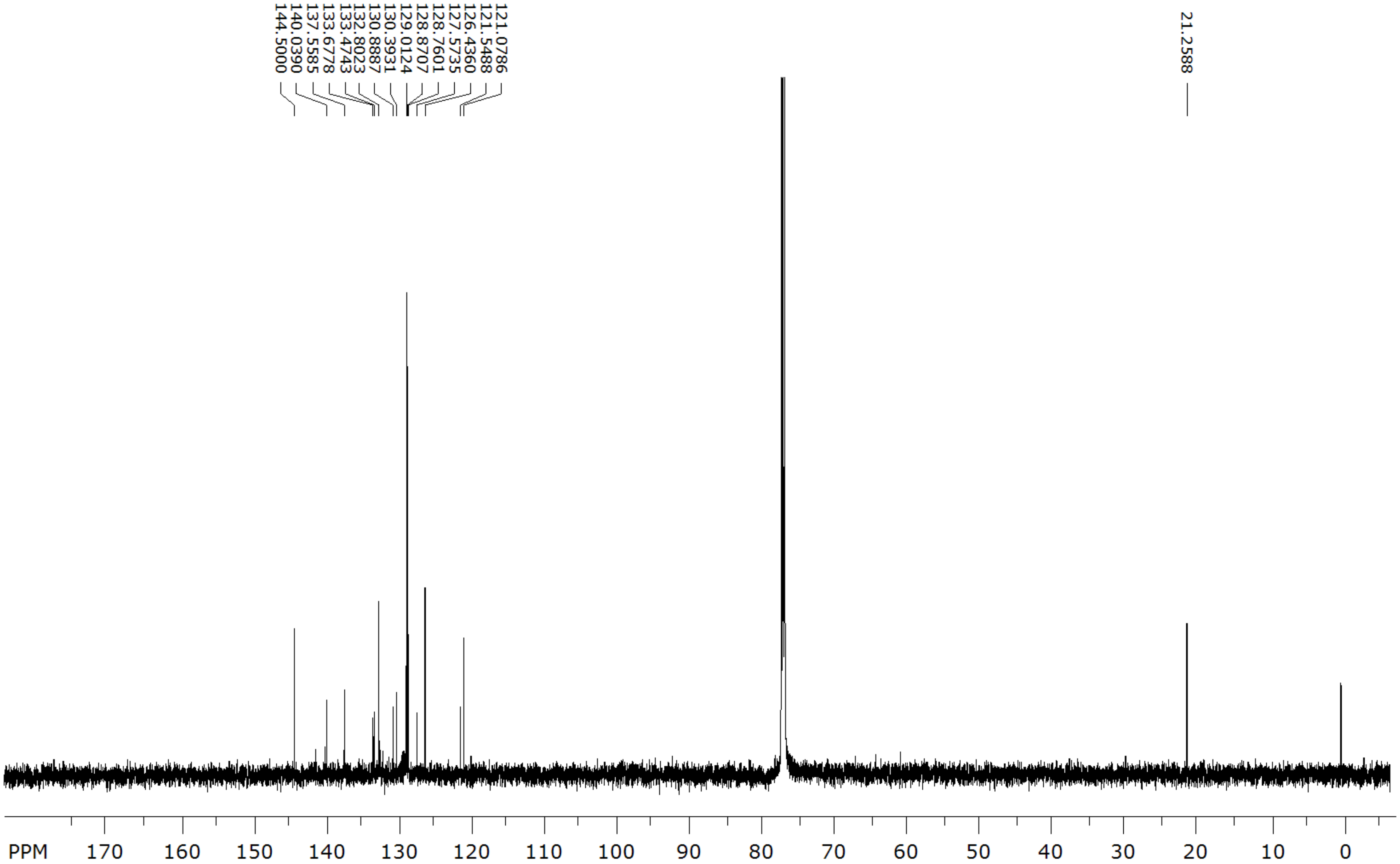

Figure S249. ^{13}C NMR ($CDCl_3$) spectrum of *cis,syn*-**18**.

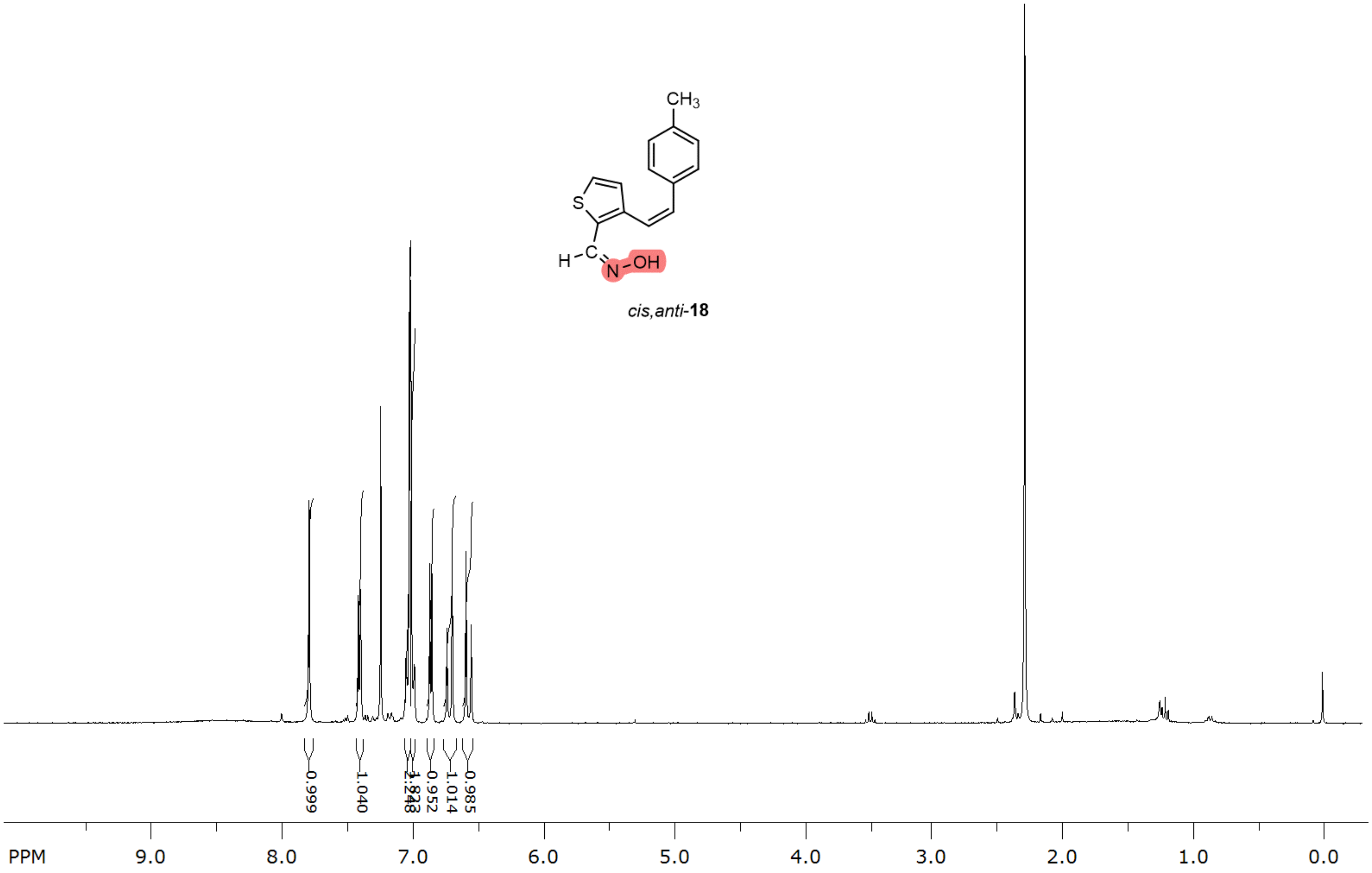

Figure S250. ^{1}H NMR ($CDCl_3$) spectrum of *cis,anti*-**18**.

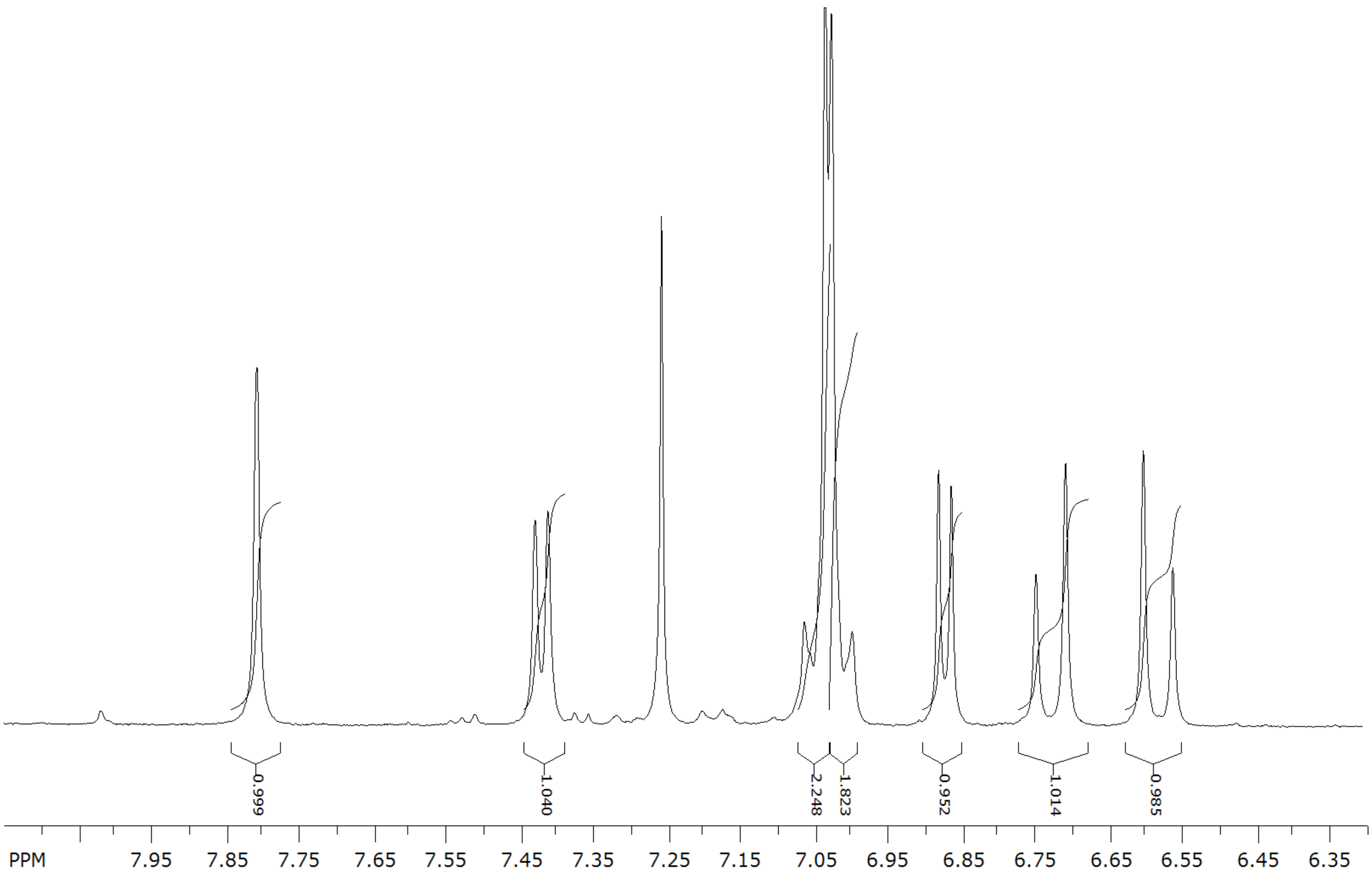

Figure S251. ^{1}H NMR ($CDCl_3$) spectrum of aromatic part of *cis,anti*-**18**.

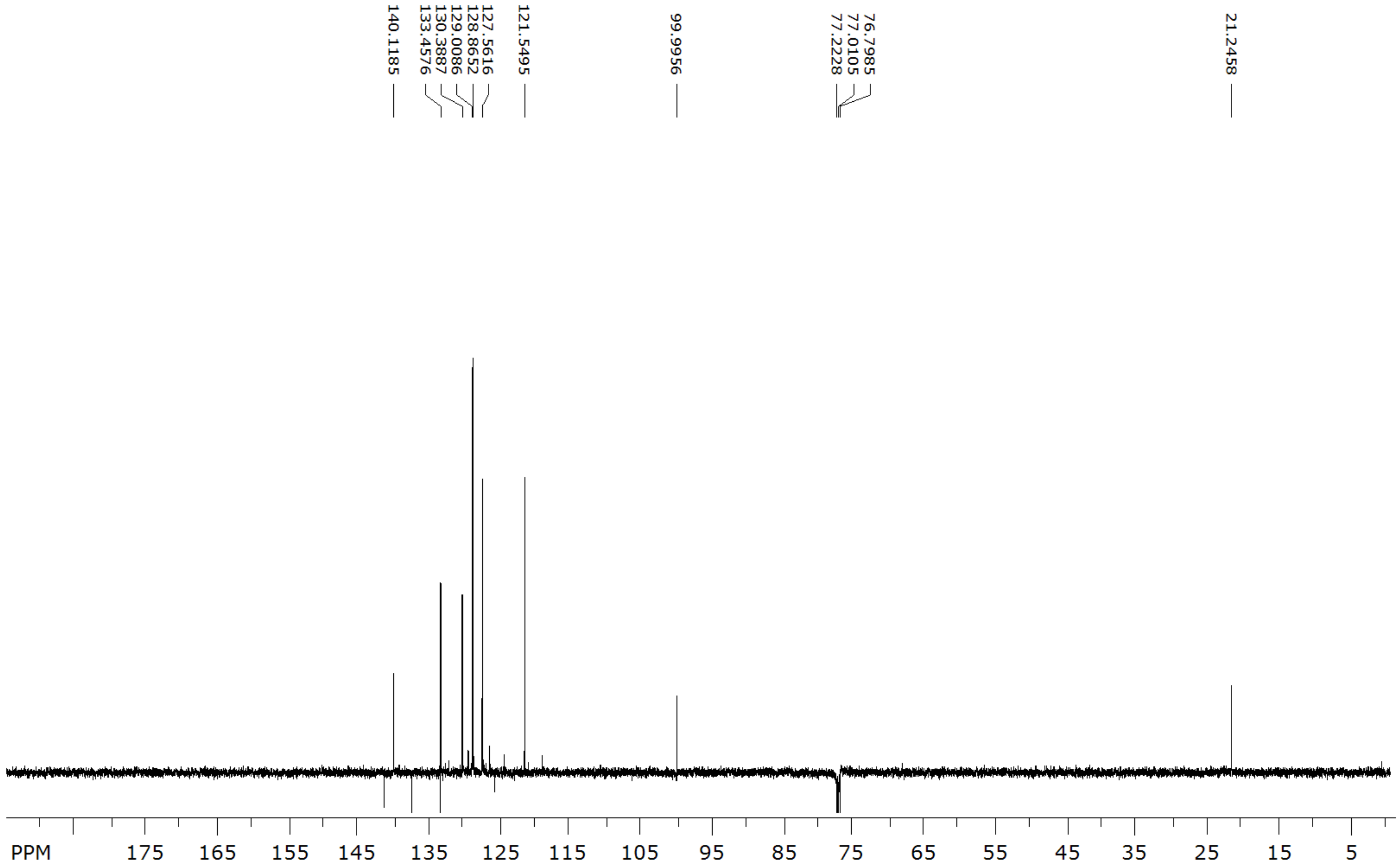

Figure S252. ^{13}C NMR ($CDCl_3$) spectrum of *cis,anti*-**18**.

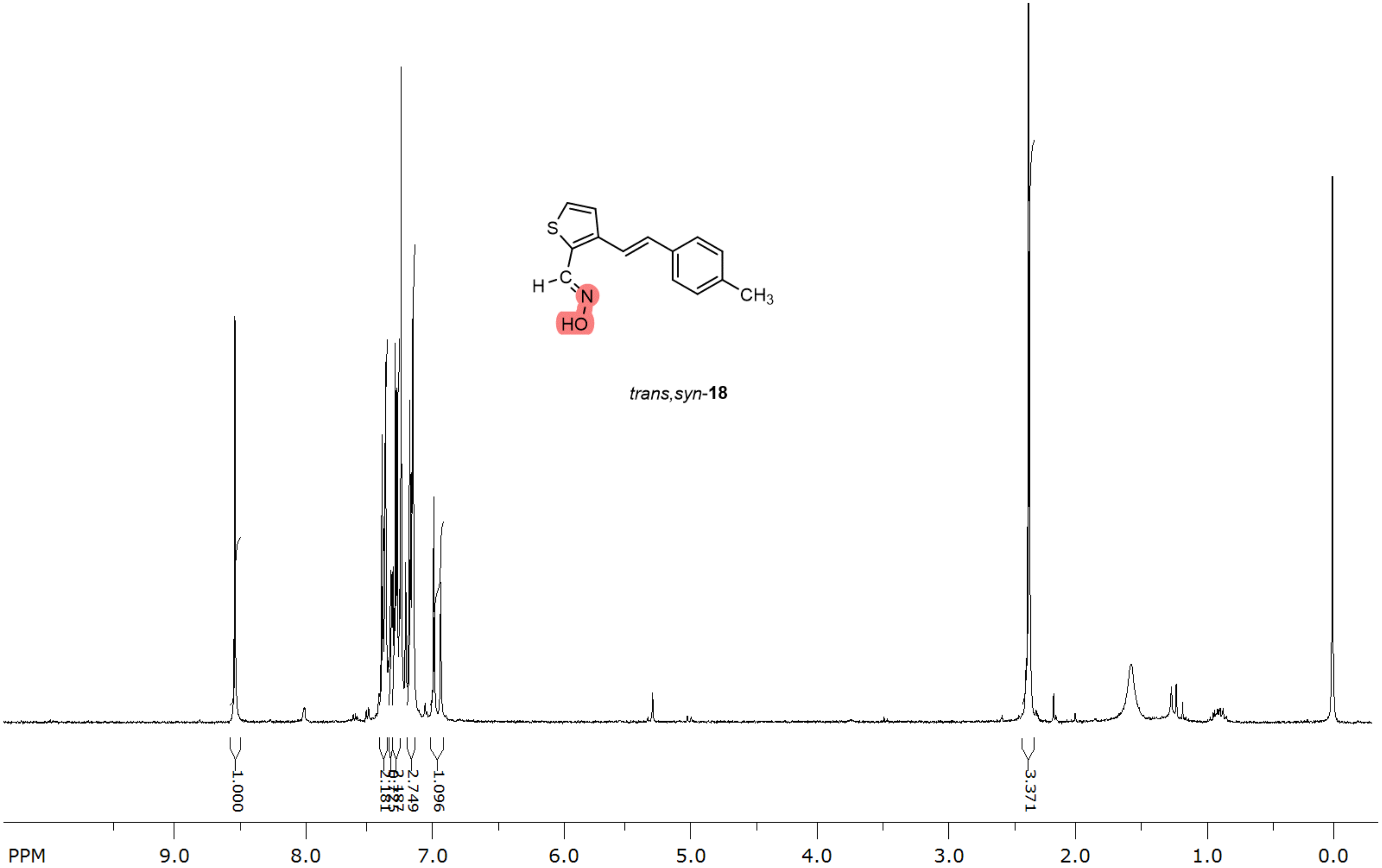

Figure S253. ^{1}H NMR ($CDCl_3$) spectrum of *trans,syn*-**18**.

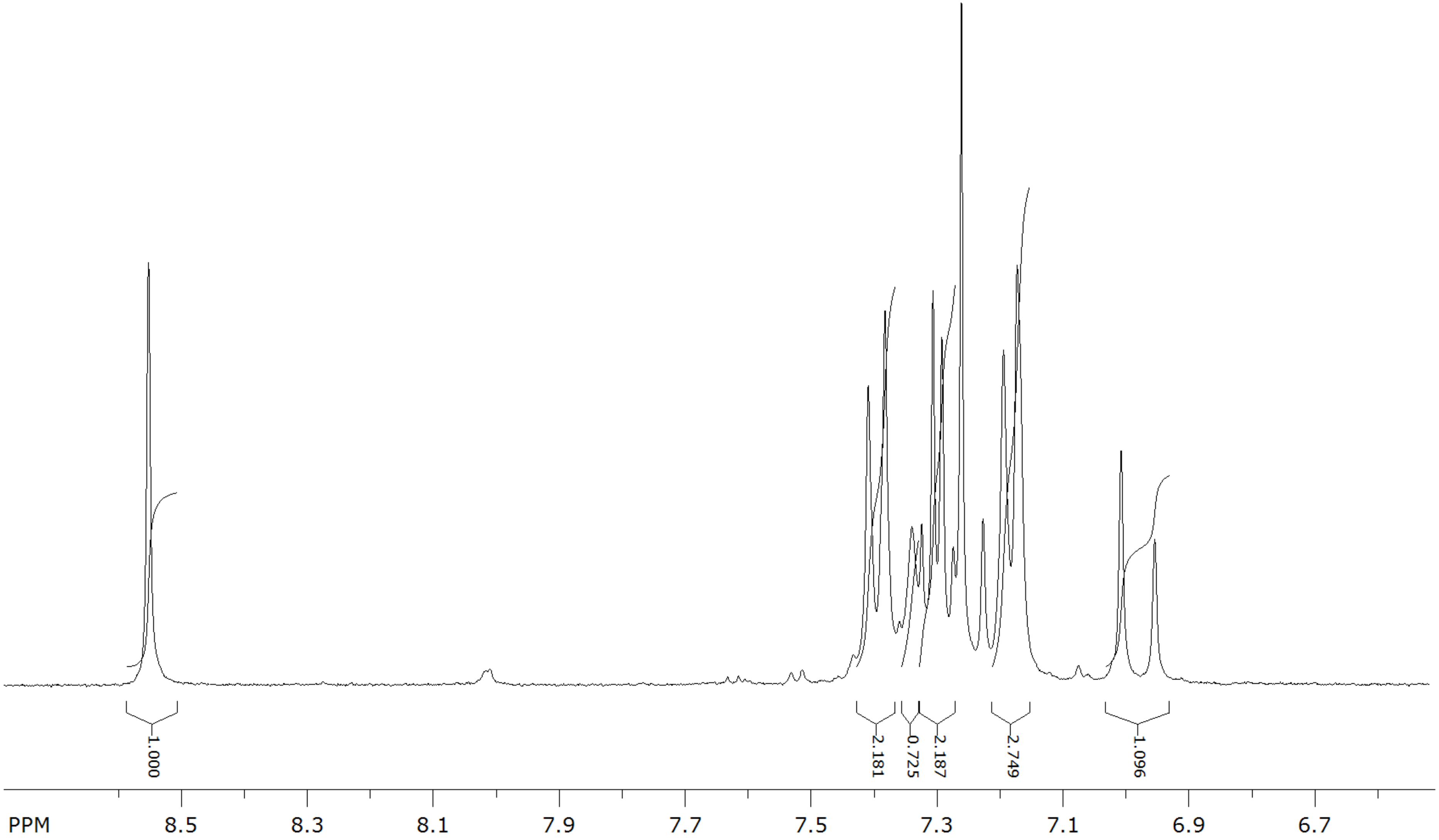

Figure S254. ^{1}H NMR ($CDCl_3$) spectrum of aromatic part of *trans,syn*-**18**.

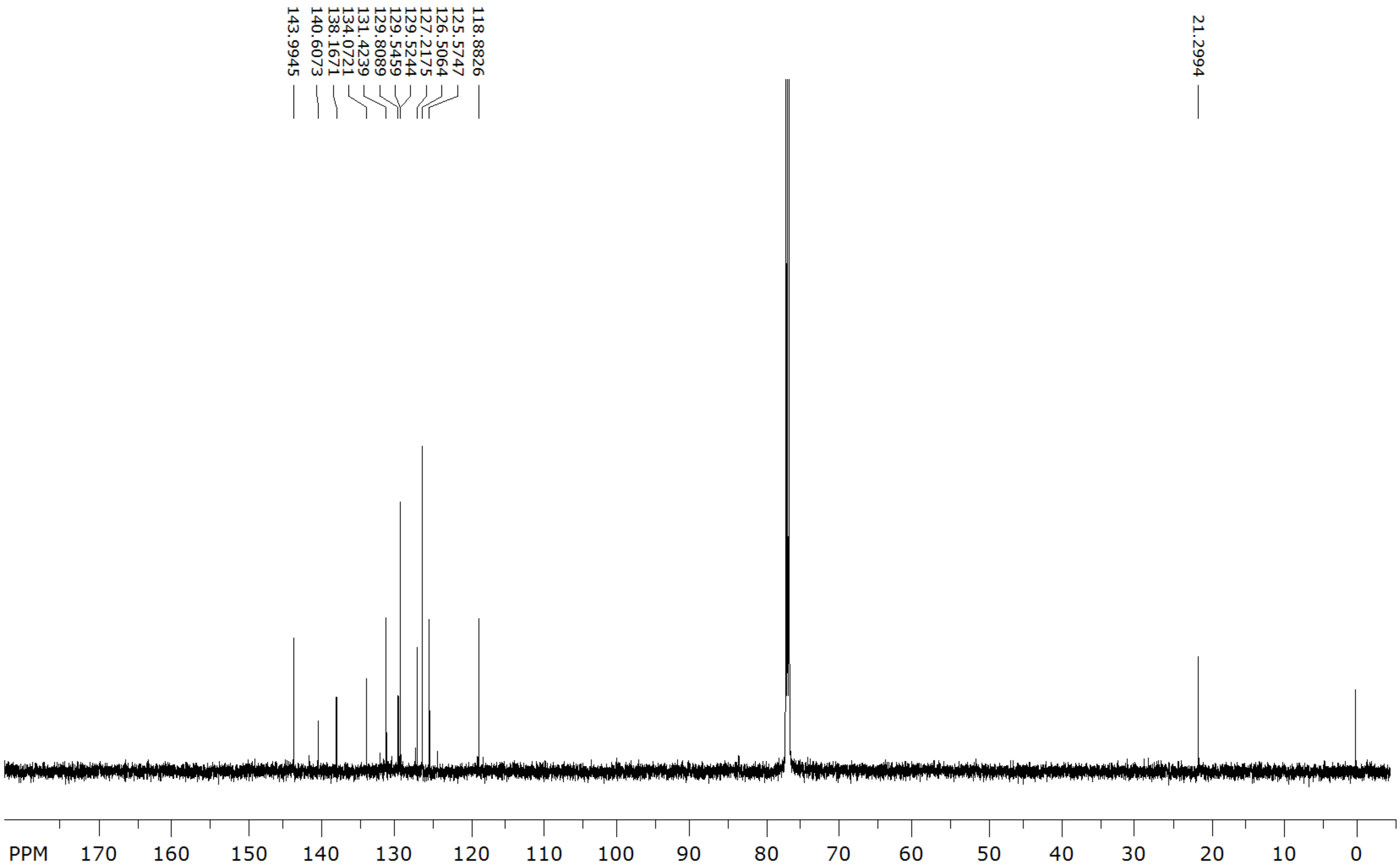

Figure S255. ^{13}C NMR ($CDCl_3$) spectrum of *trans,syn*-**18**.

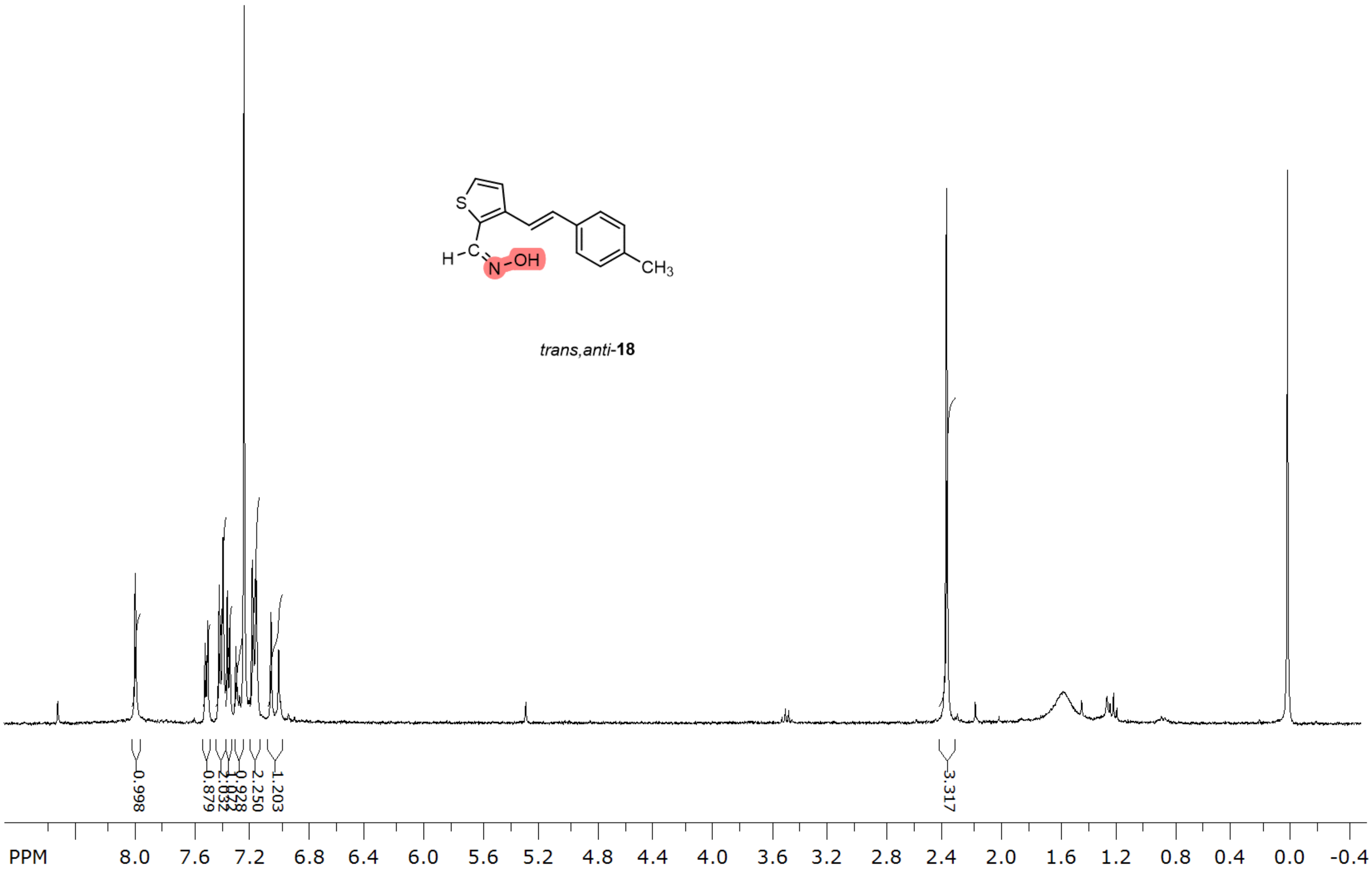

Figure S256. ^{1}H NMR ($CDCl_3$) spectrum of *trans,anti*-**18**.

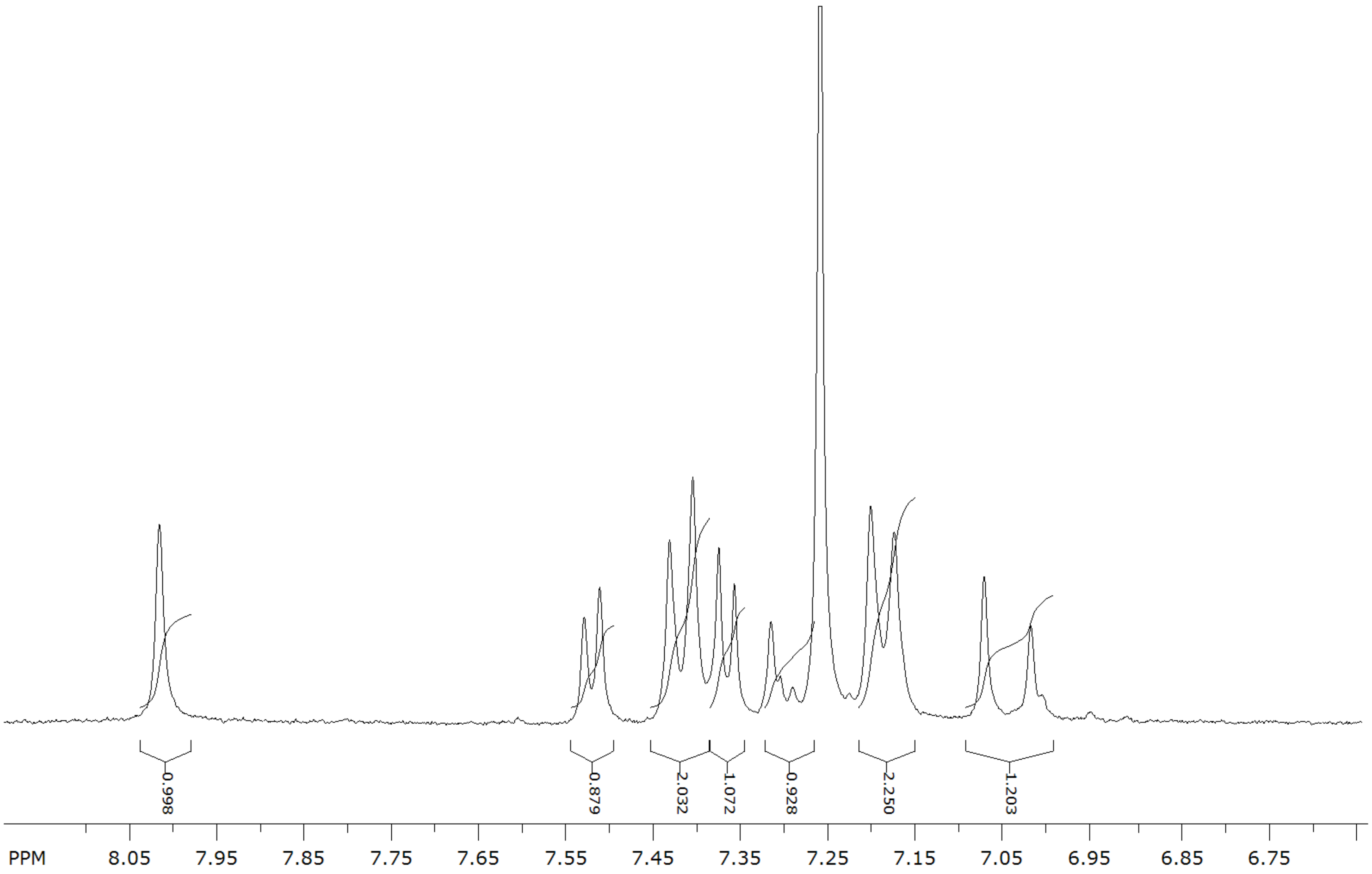

Figure S257. ¹H NMR ($CDCl_3$) spectrum of aromatic part of *trans,anti*-**18**.

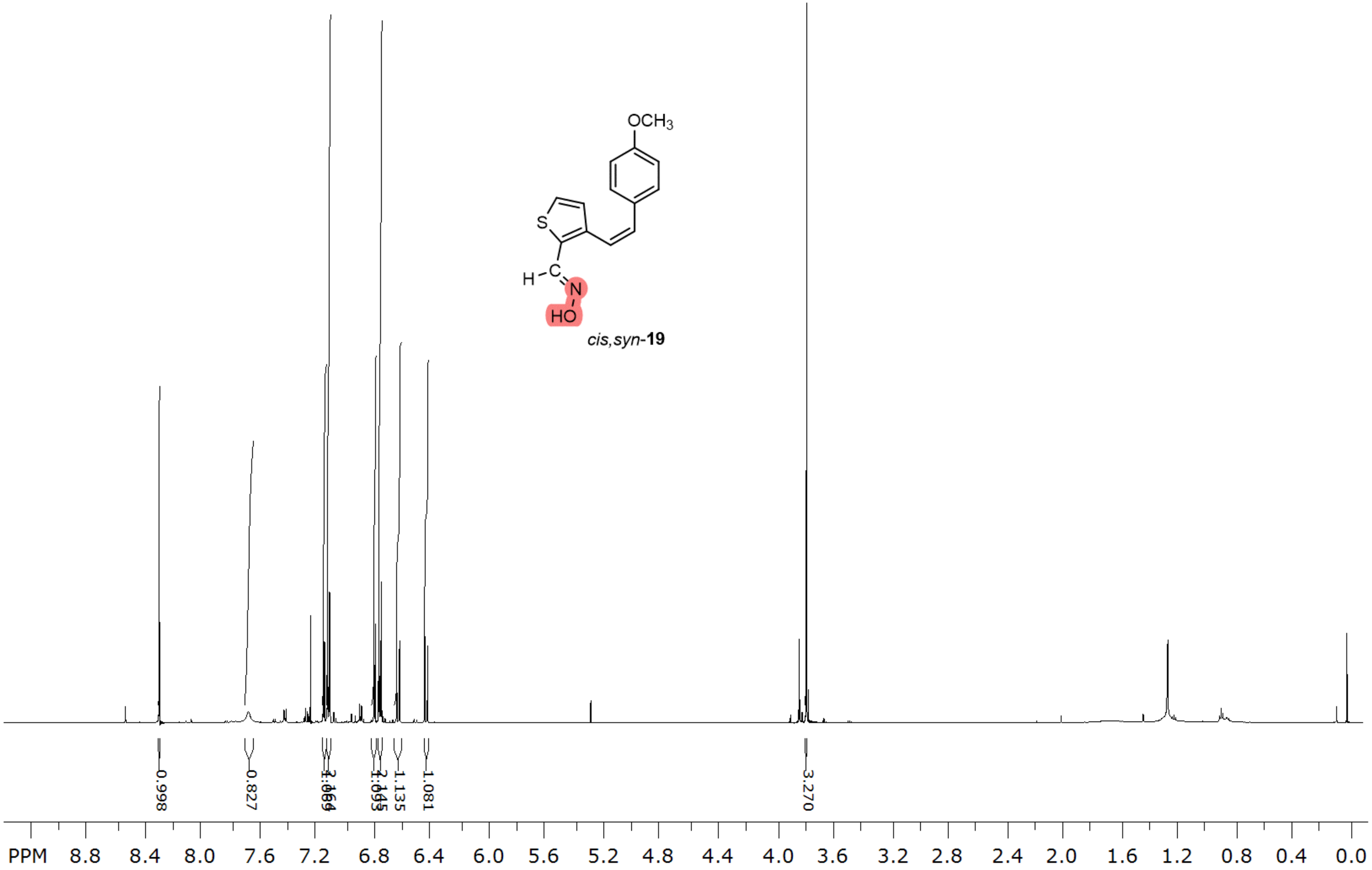

Figure S258. ^{1}H NMR ($CDCl_3$) spectrum of *cis,syn*-**19**.

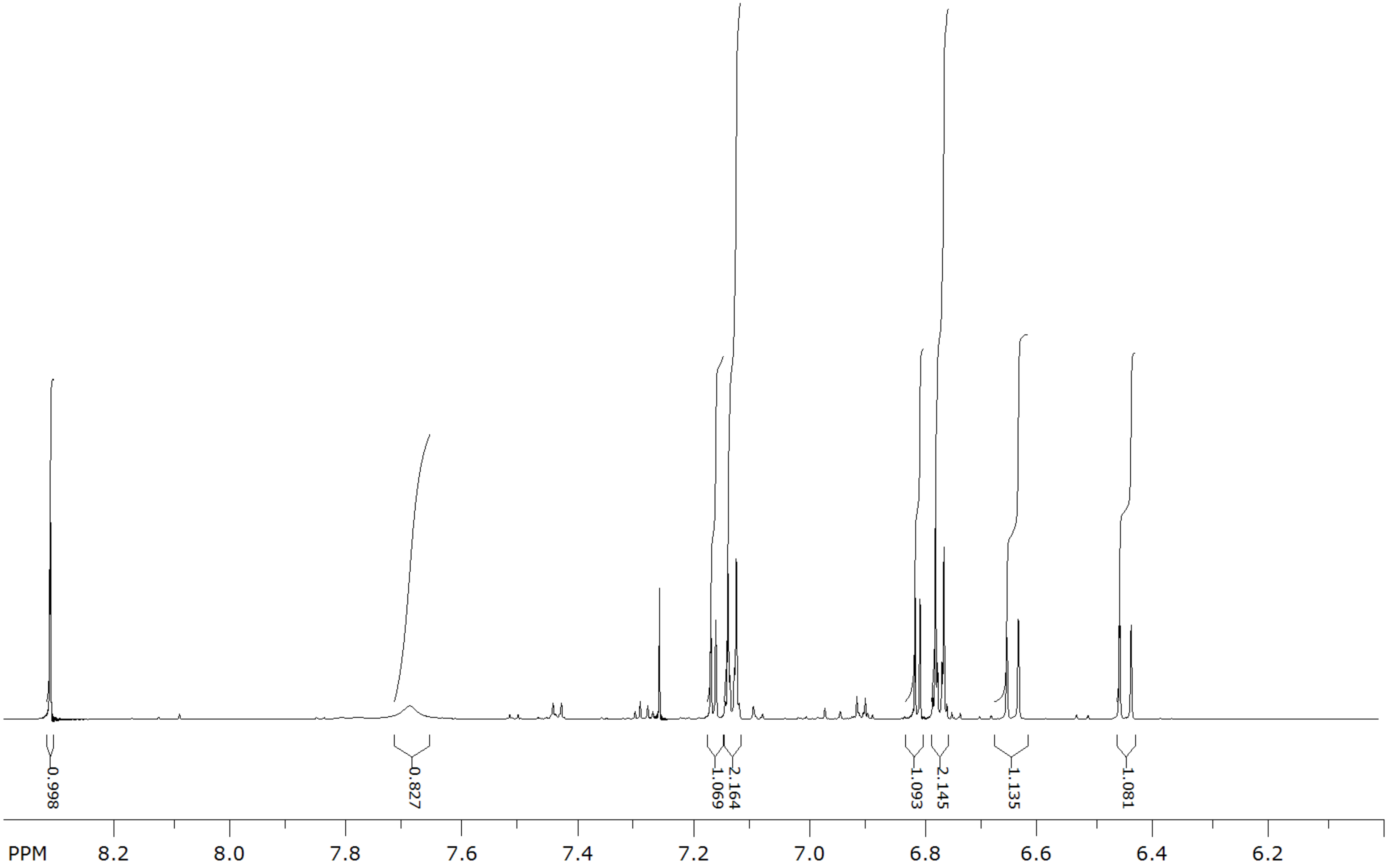

Figure S259. ^{1}H NMR ($CDCl_3$) spectrum of aromatic part of *cis,syn*-**19**.

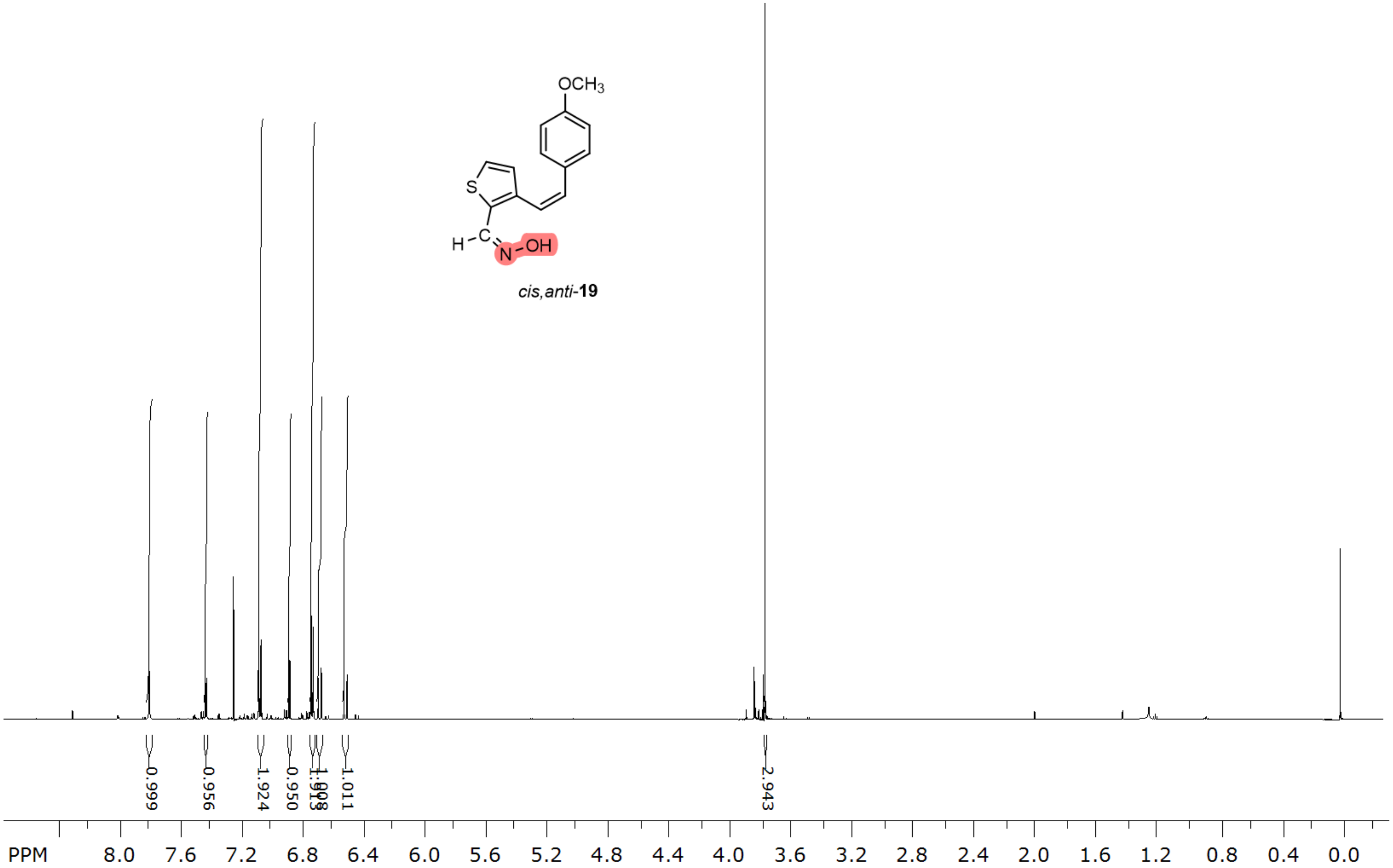

Figure S260. ^{1}H NMR ($CDCl_3$) spectrum of *cis,anti*-**19**.

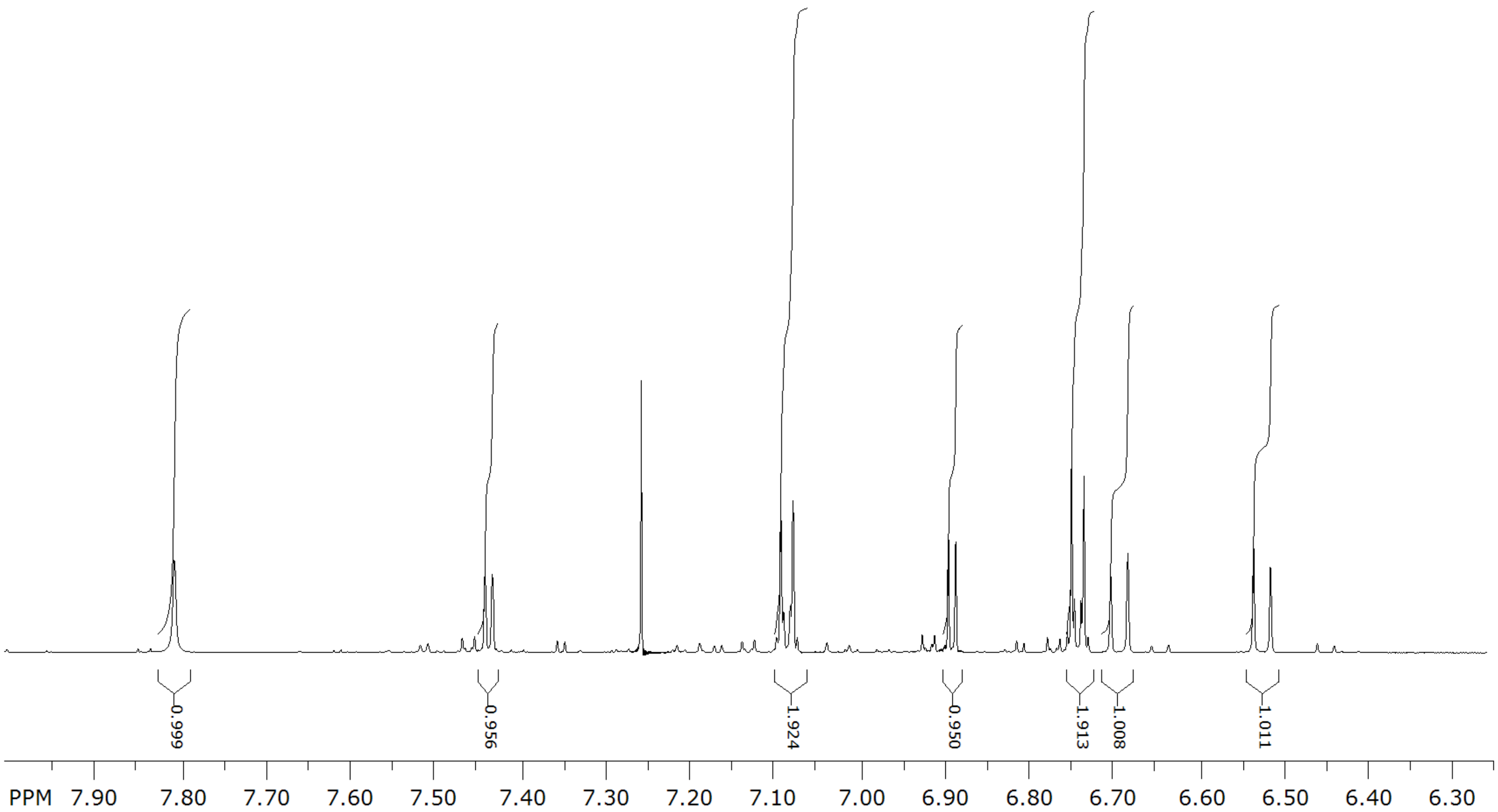

Figure S261. ^{1}H NMR ($CDCl_3$) spectrum of aromatic part of *cis,anti*-**19**.

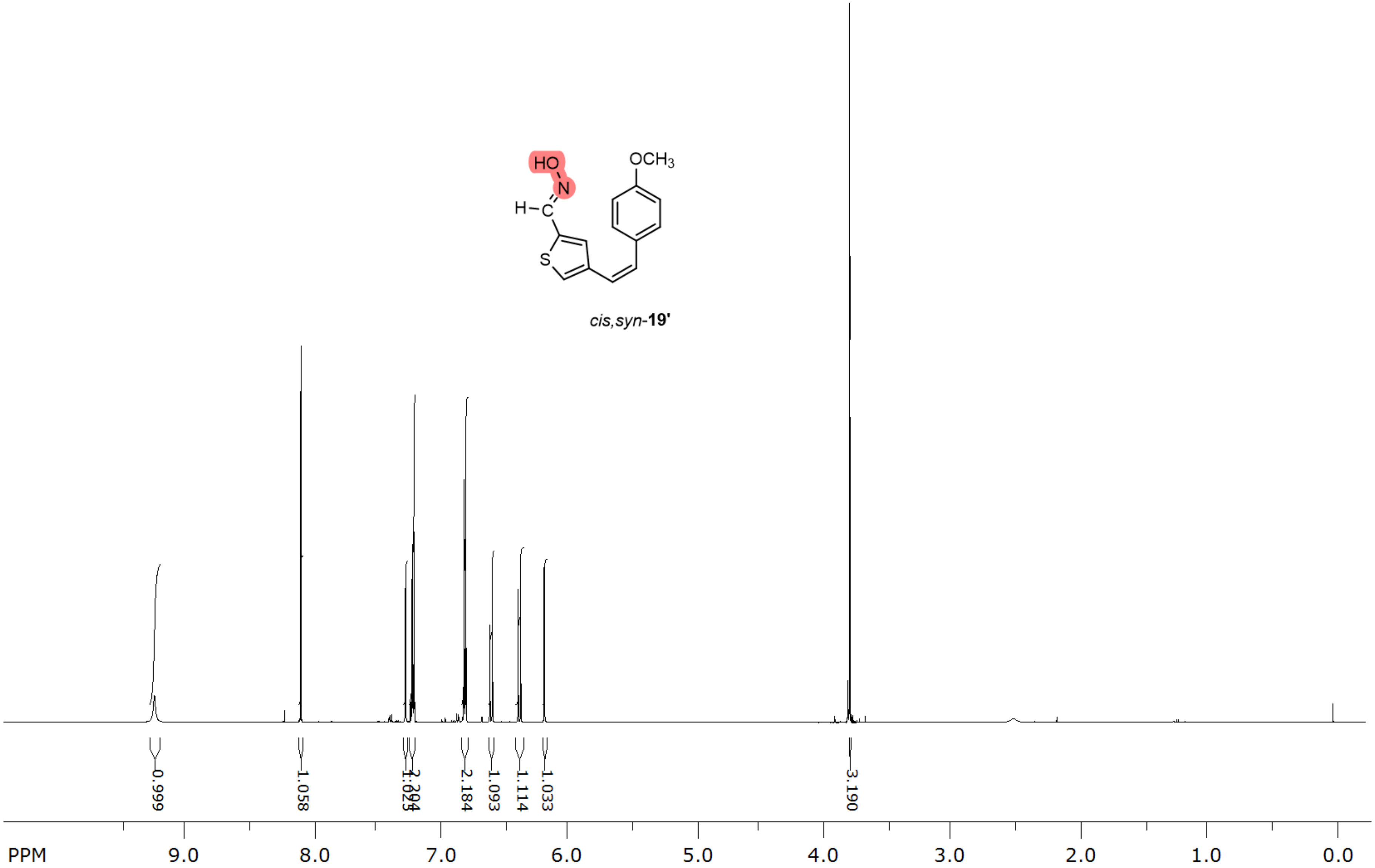

Figure S262. ^{1}H NMR ($CDCl_3$) spectrum of *cis,syn*-**19'**.

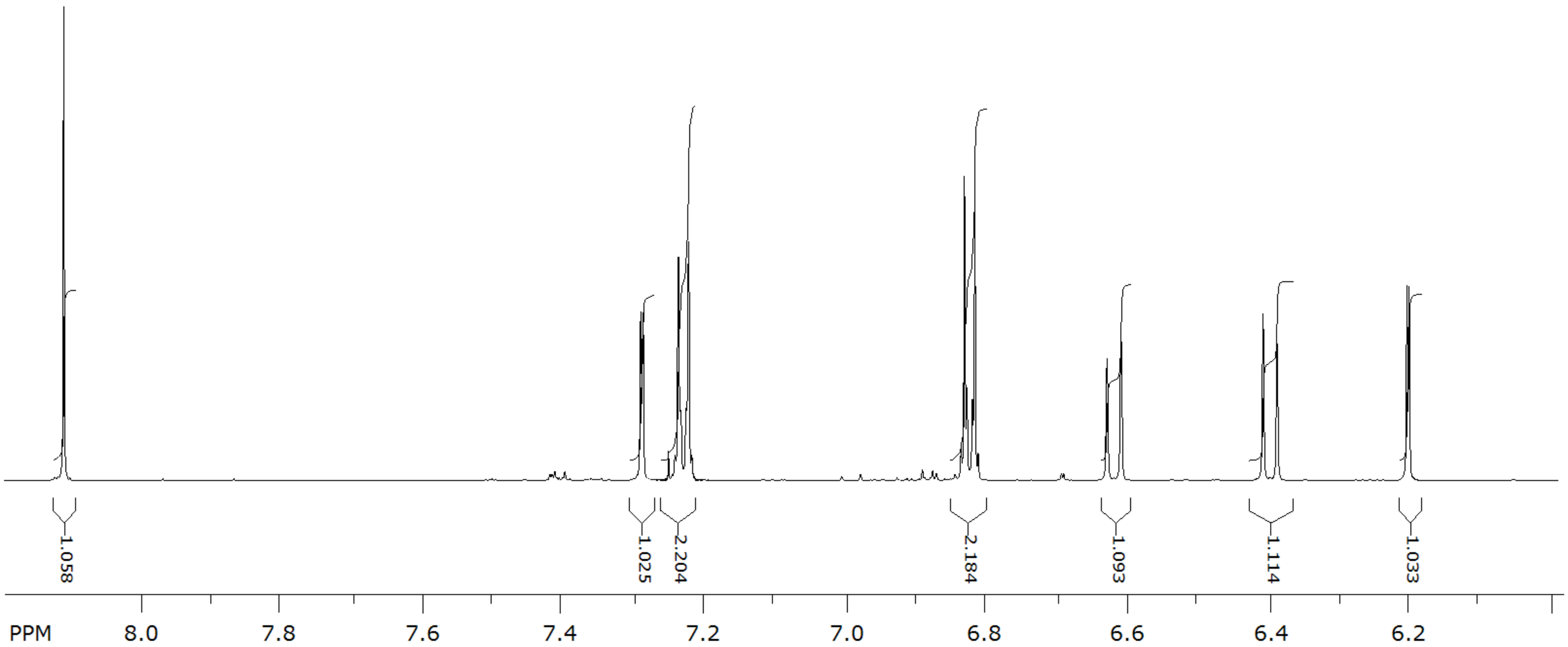

Figure S263. ^{1}H NMR ($CDCl_3$) spectrum of aromatic part of *cis,syn*-**19'**.

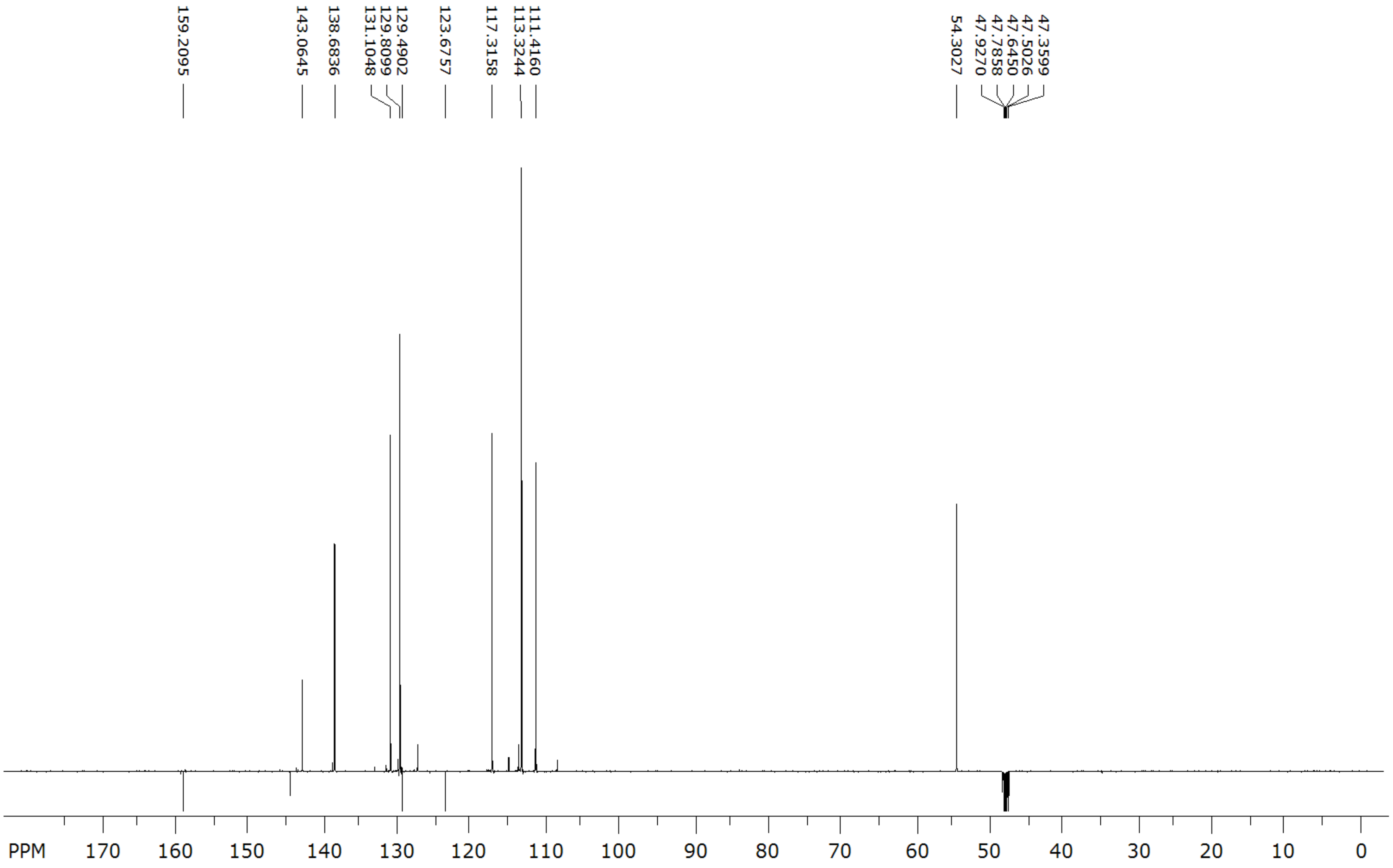

Figure S264. ^{13}C NMR ($CDCl_3$) spectrum of *cis,syn*-**19'**.

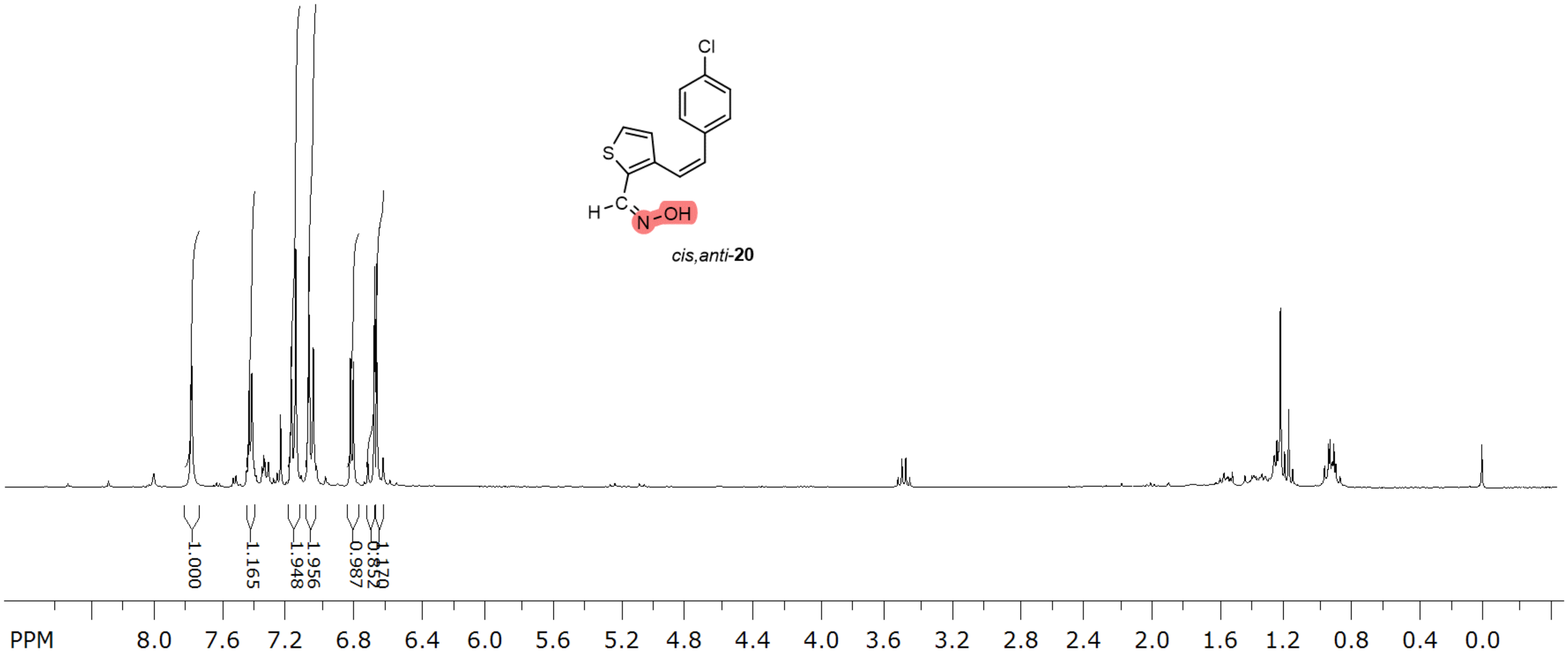

Figure S265. ^{1}H NMR ($CDCl_3$) spectrum of *cis,anti*-**20**.

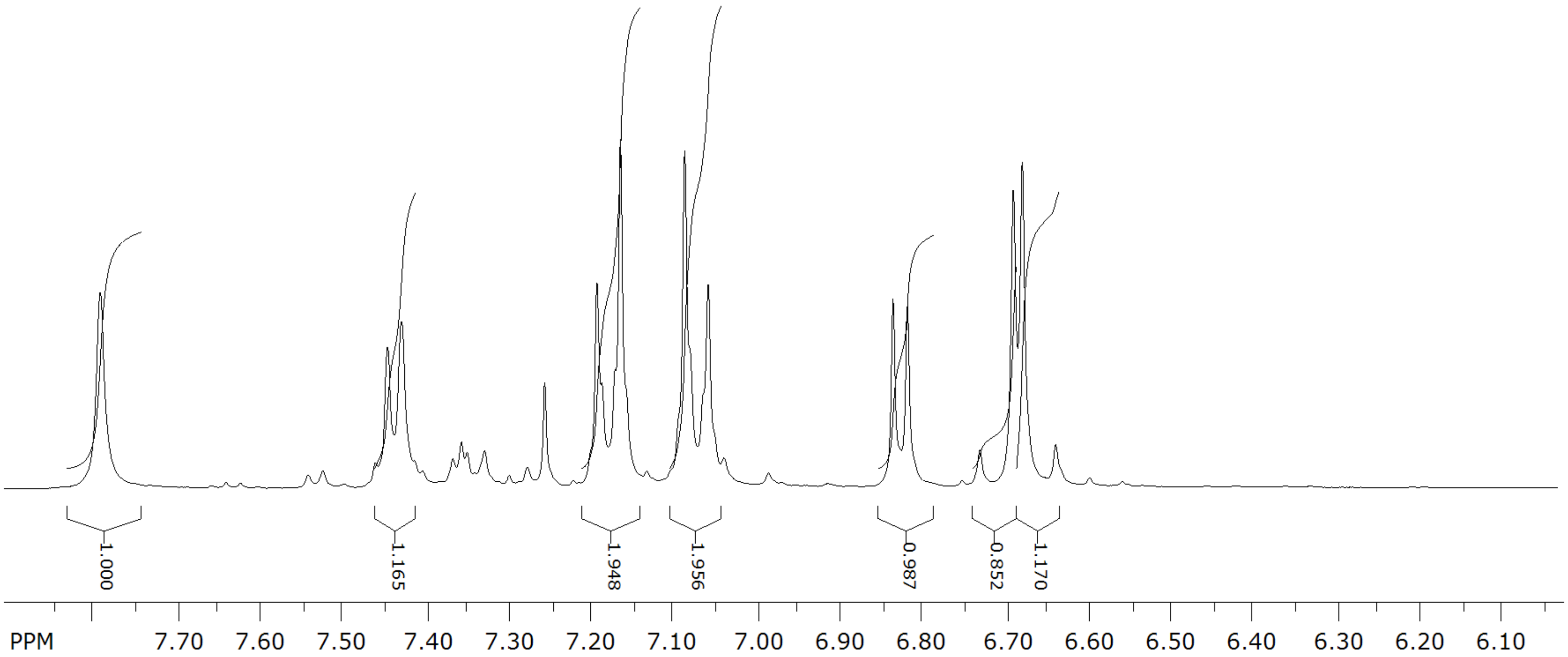

Figure S266. ^{1}H NMR ($CDCl_3$) spectrum of aromatic part of *cis,anti*-**20**.

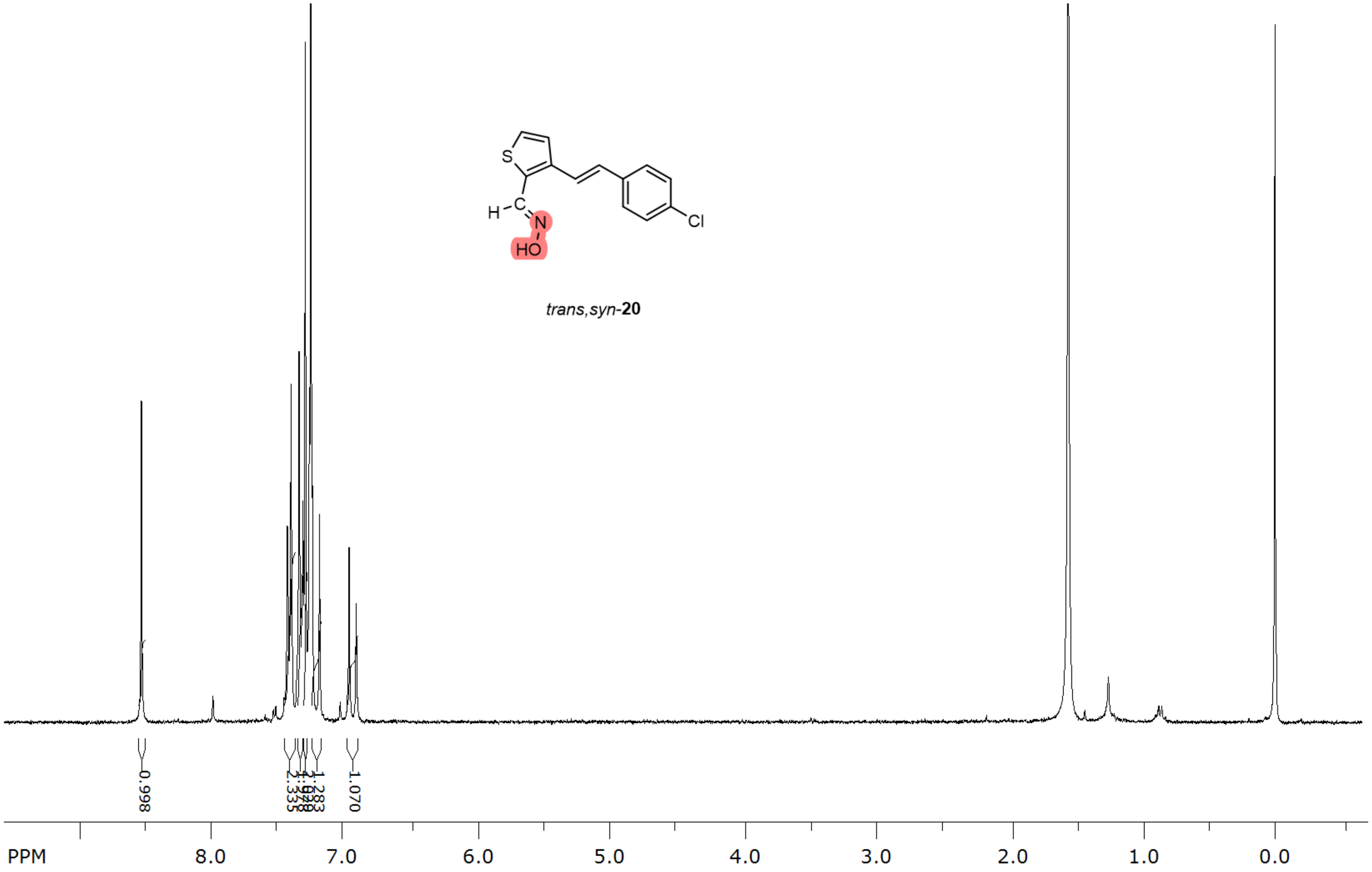

Figure S267. ^1H NMR ($CDCl_3$) spectrum of *trans,syn*-**20**.

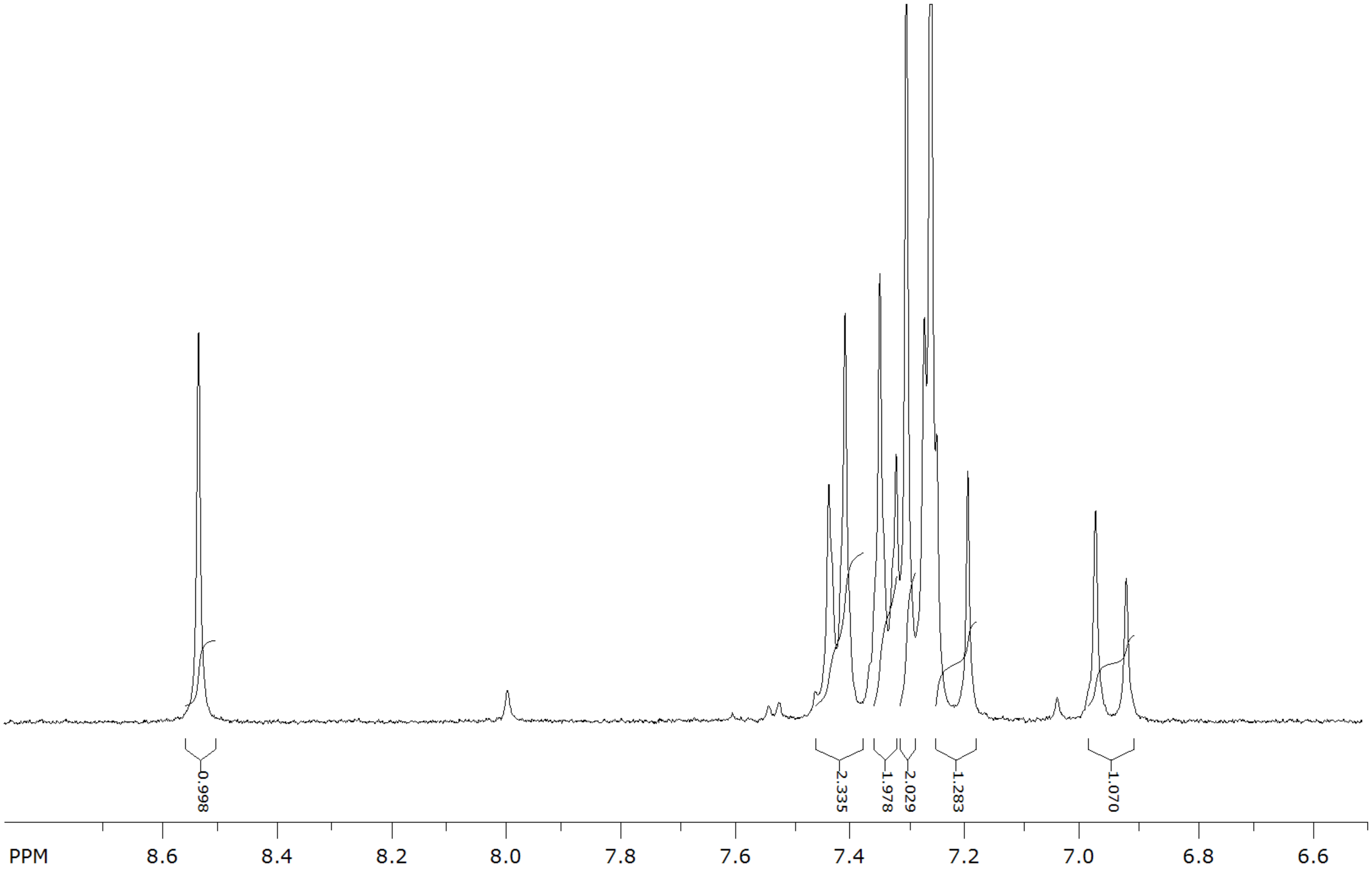

Figure S268. ^{1}H NMR ($CDCl_3$) spectrum of aromatic part of *trans,syn*-**20**.

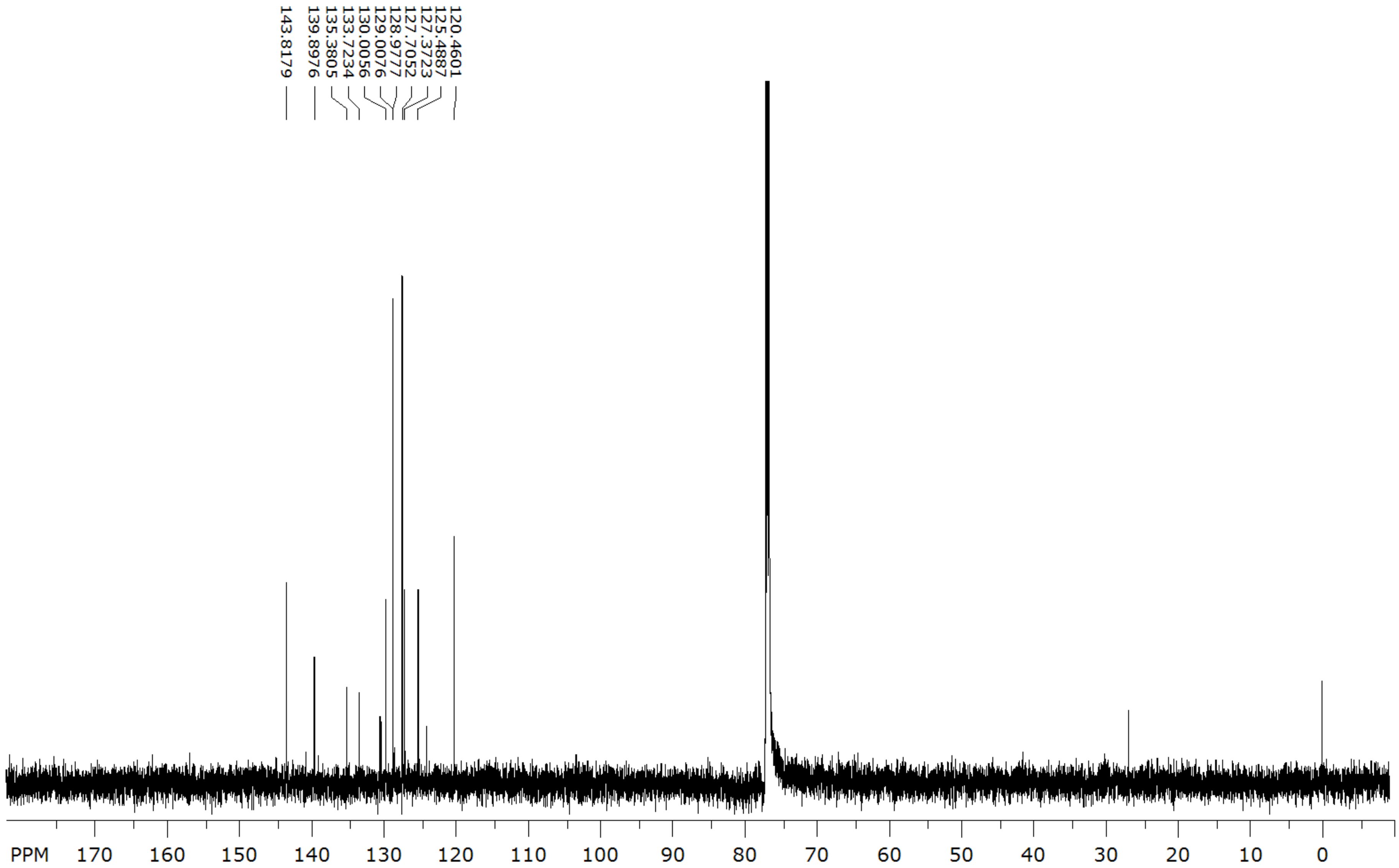

Figure S269. ^{13}C NMR ($CDCl_3$) spectrum of *trans,syn*-**20**.

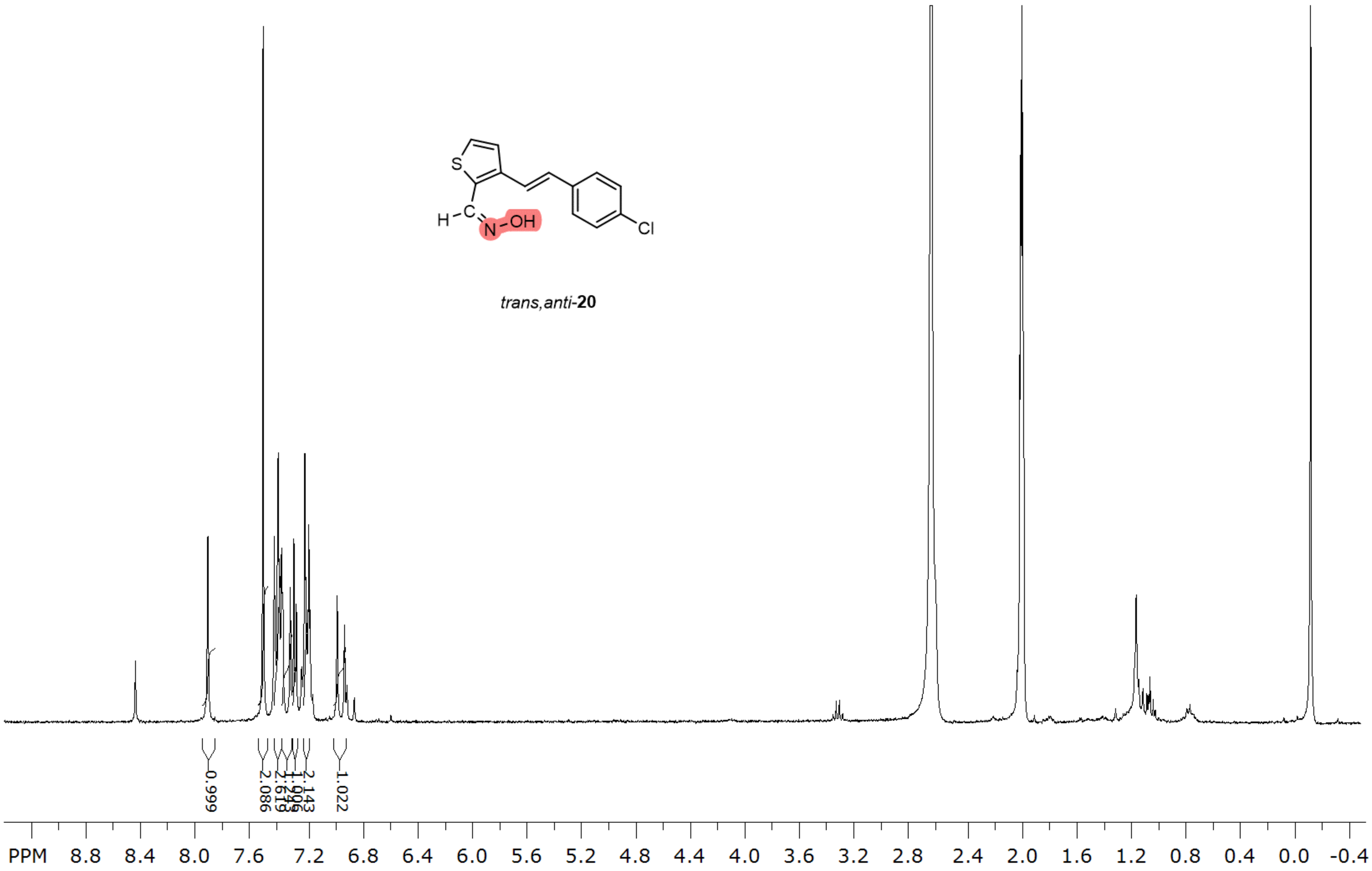

Figure S270. ^{1}H NMR ($CDCl_3$) spectrum of *trans,anti*-**20**.

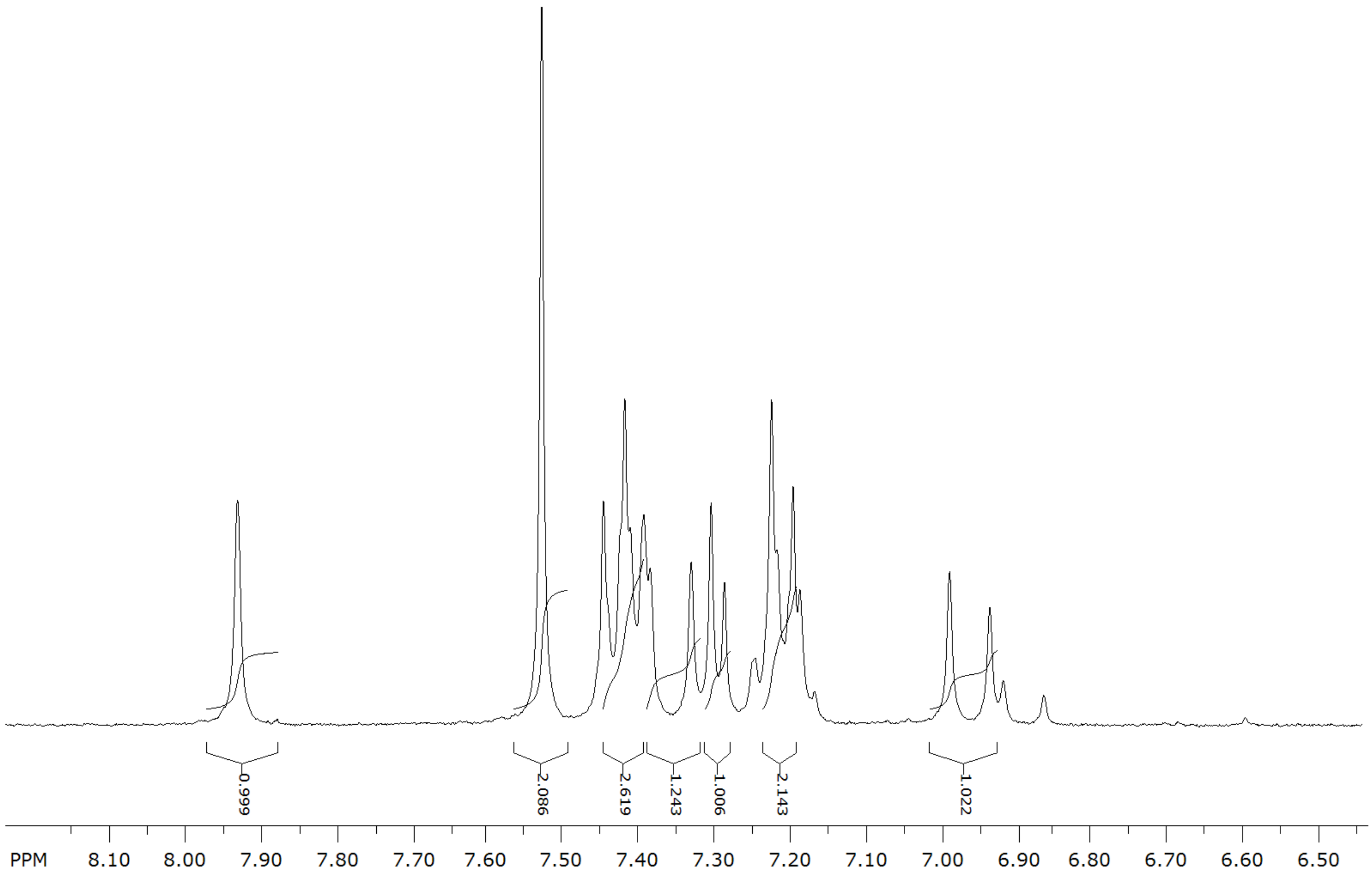

Figure S271. ^{1}H NMR ($CDCl_3$) spectrum of aromatic part of *trans,anti*-**20**.

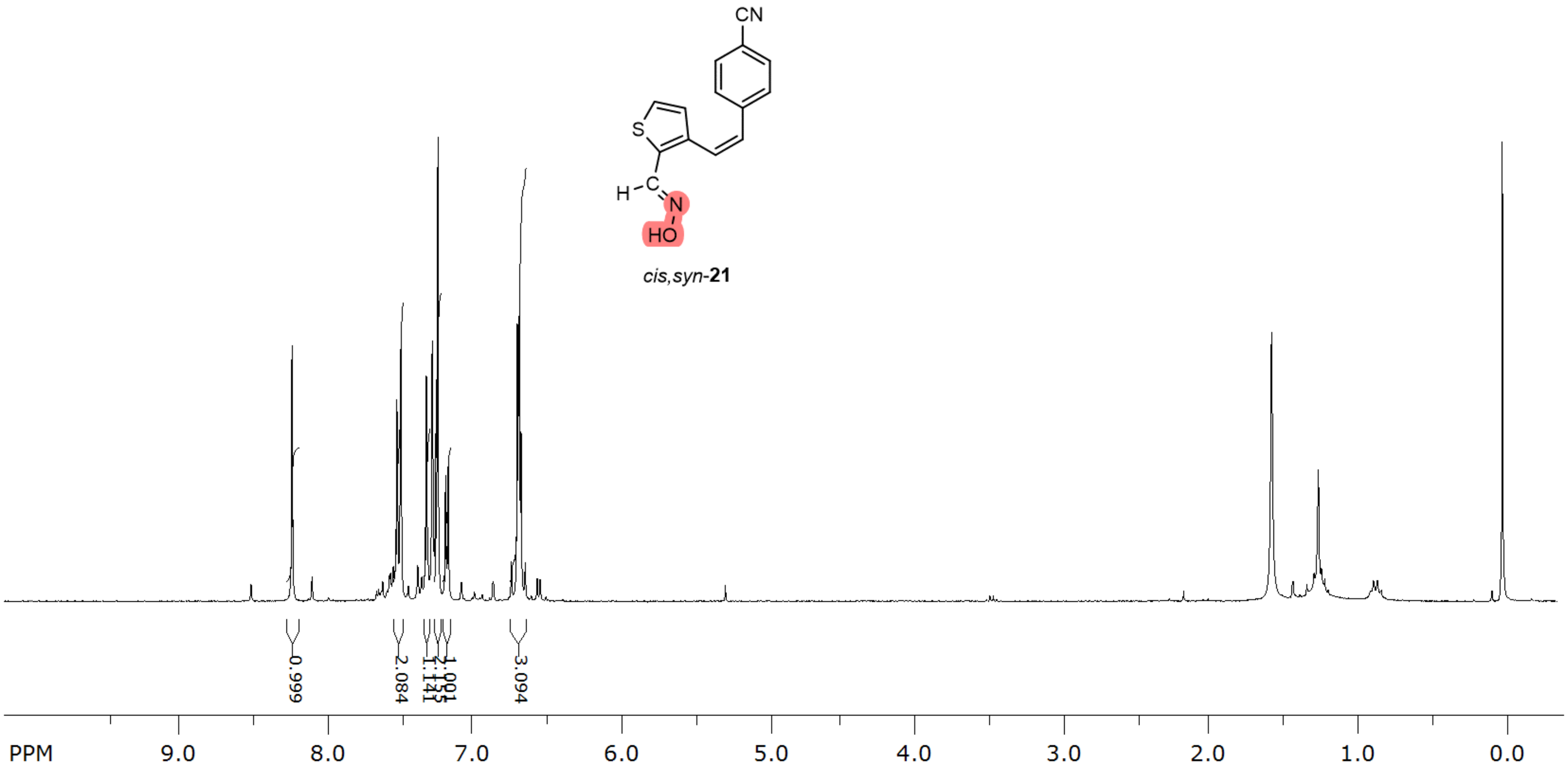

Figure S272. ^{1}H NMR ($CDCl_3$) spectrum of *cis,syn*-**21**.

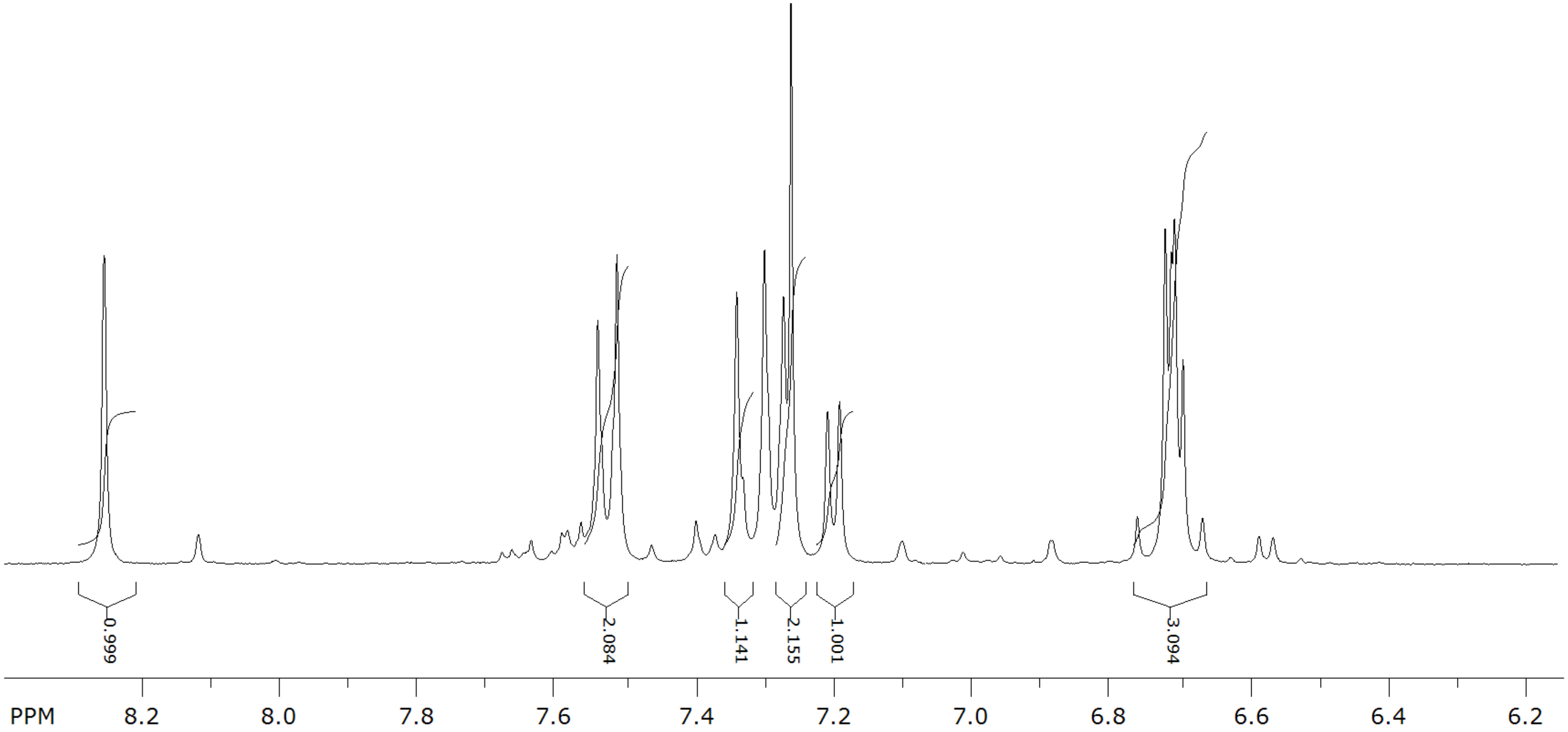

Figure S273. ^{1}H NMR ($CDCl_3$) spectrum of aromatic part of *cis,syn*-**21**.

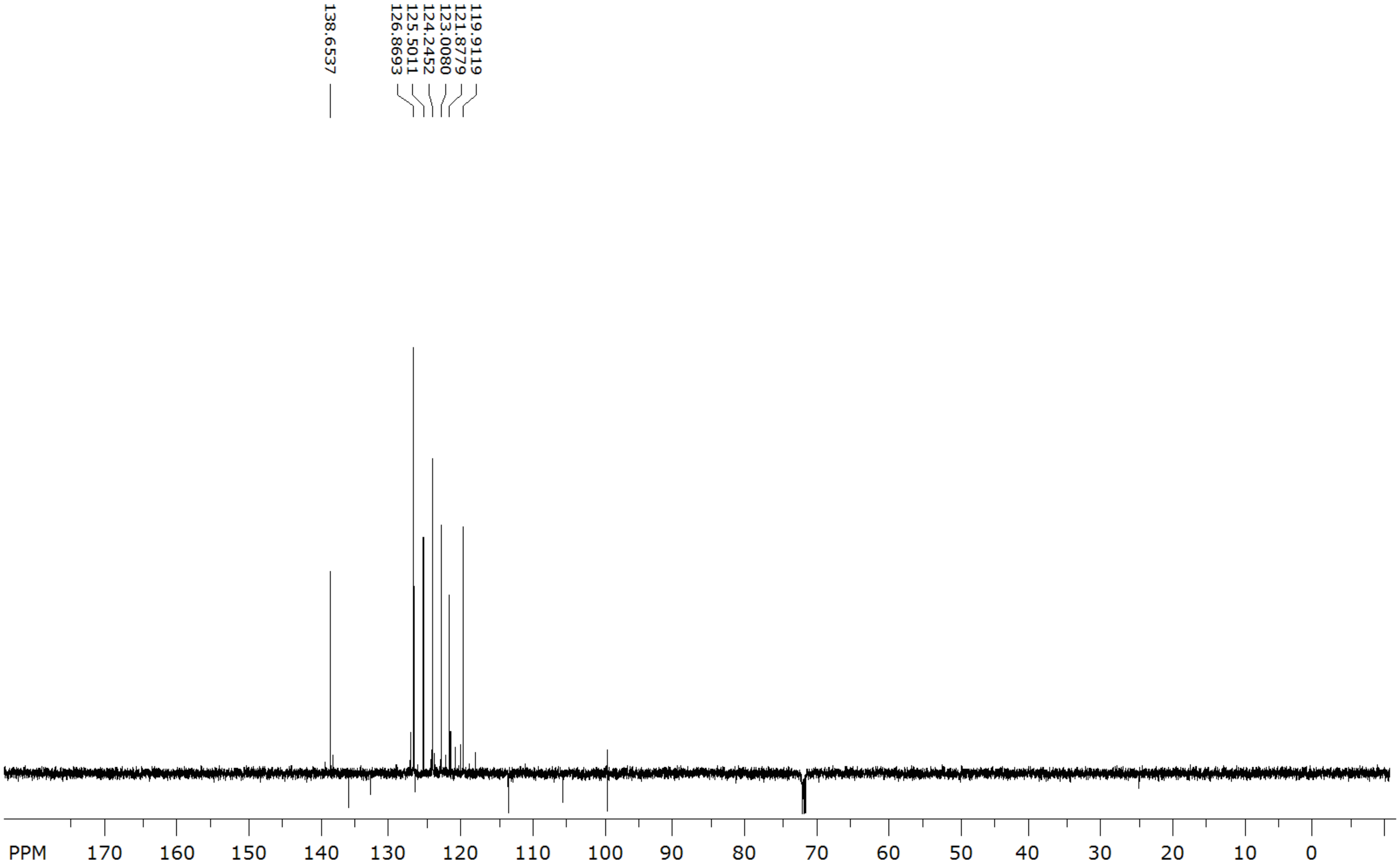

Figure S274. ^{13}C NMR ($CDCl_3$) spectrum of *cis,syn*-**21**.

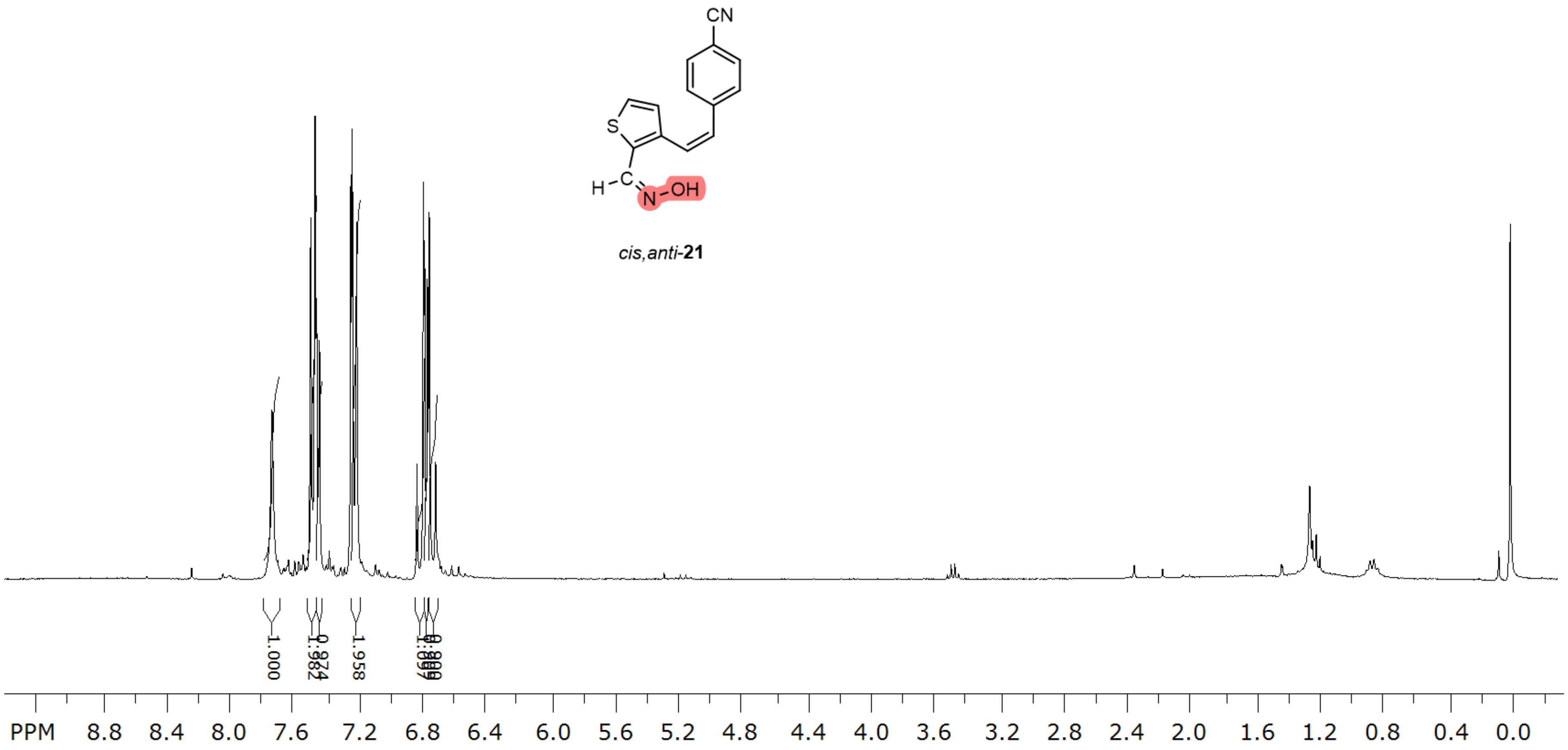

Figure S275. ^{1}H NMR ($CDCl_3$) spectrum of *cis,anti*-**21**.

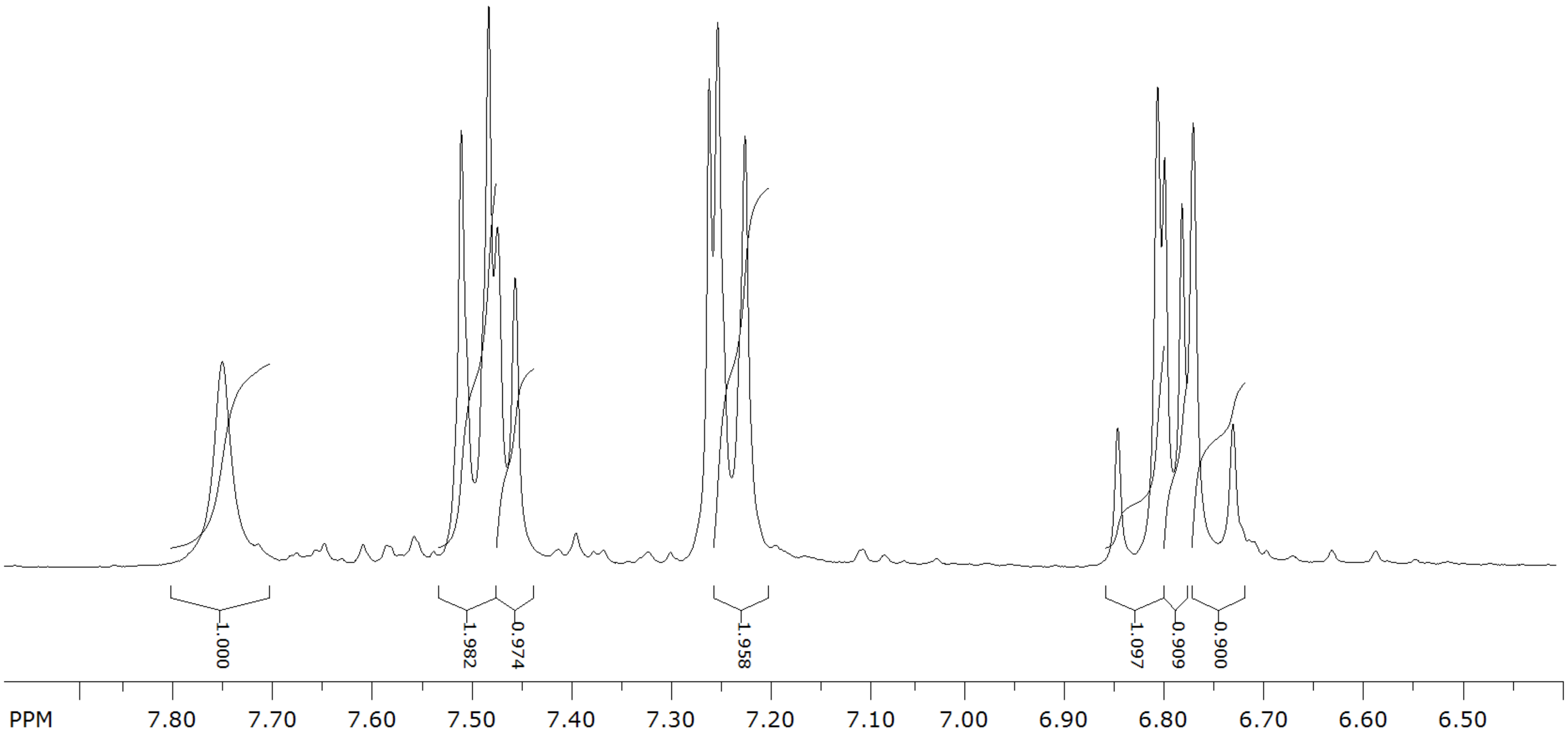

Figure S276. ^{1}H NMR ($CDCl_3$) spectrum of aromatic part of *cis,anti*-**21**.

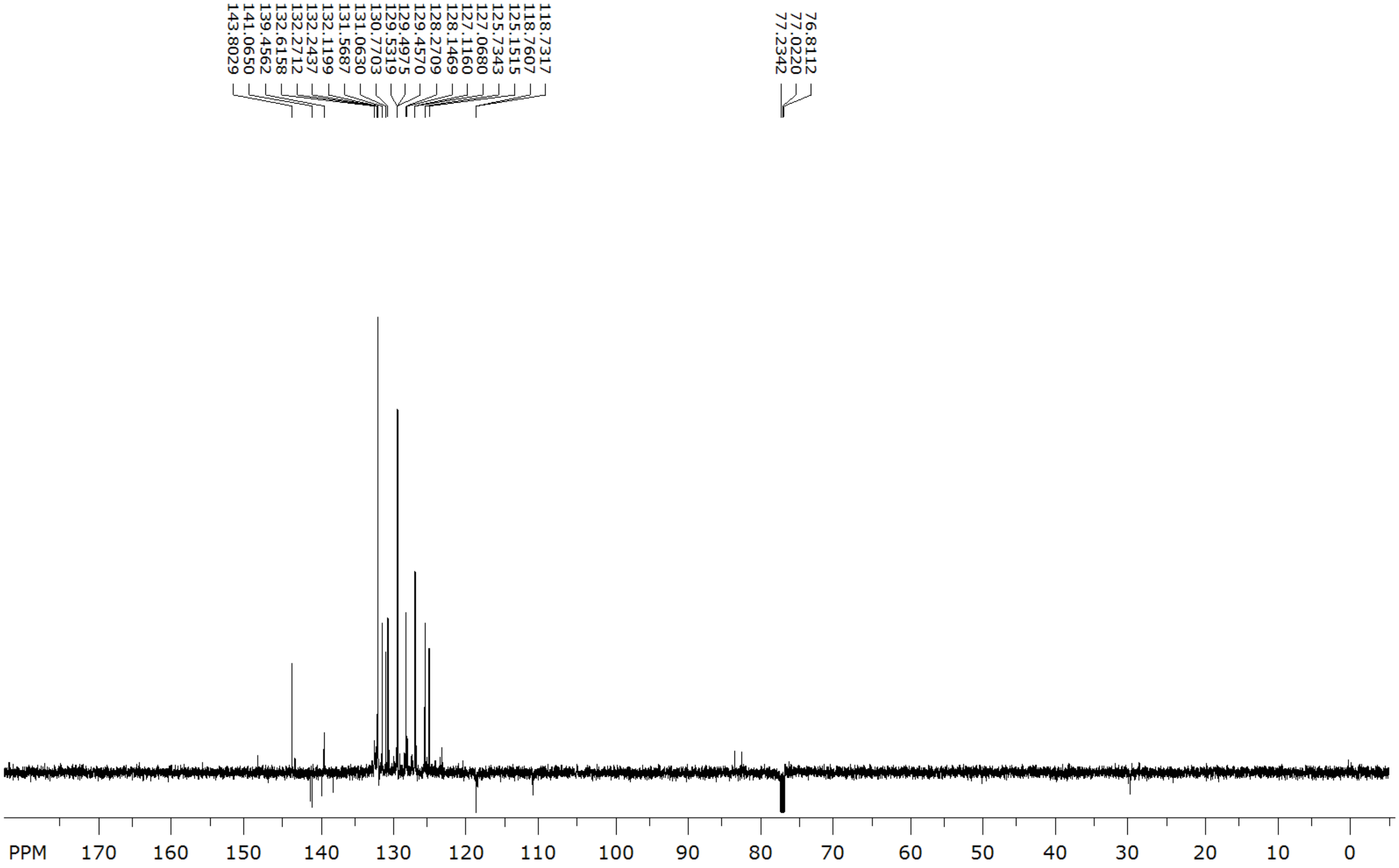

Figure S277. ^{13}C NMR ($CDCl_3$) spectrum of aromatic part of *cis,anti*-**21**.

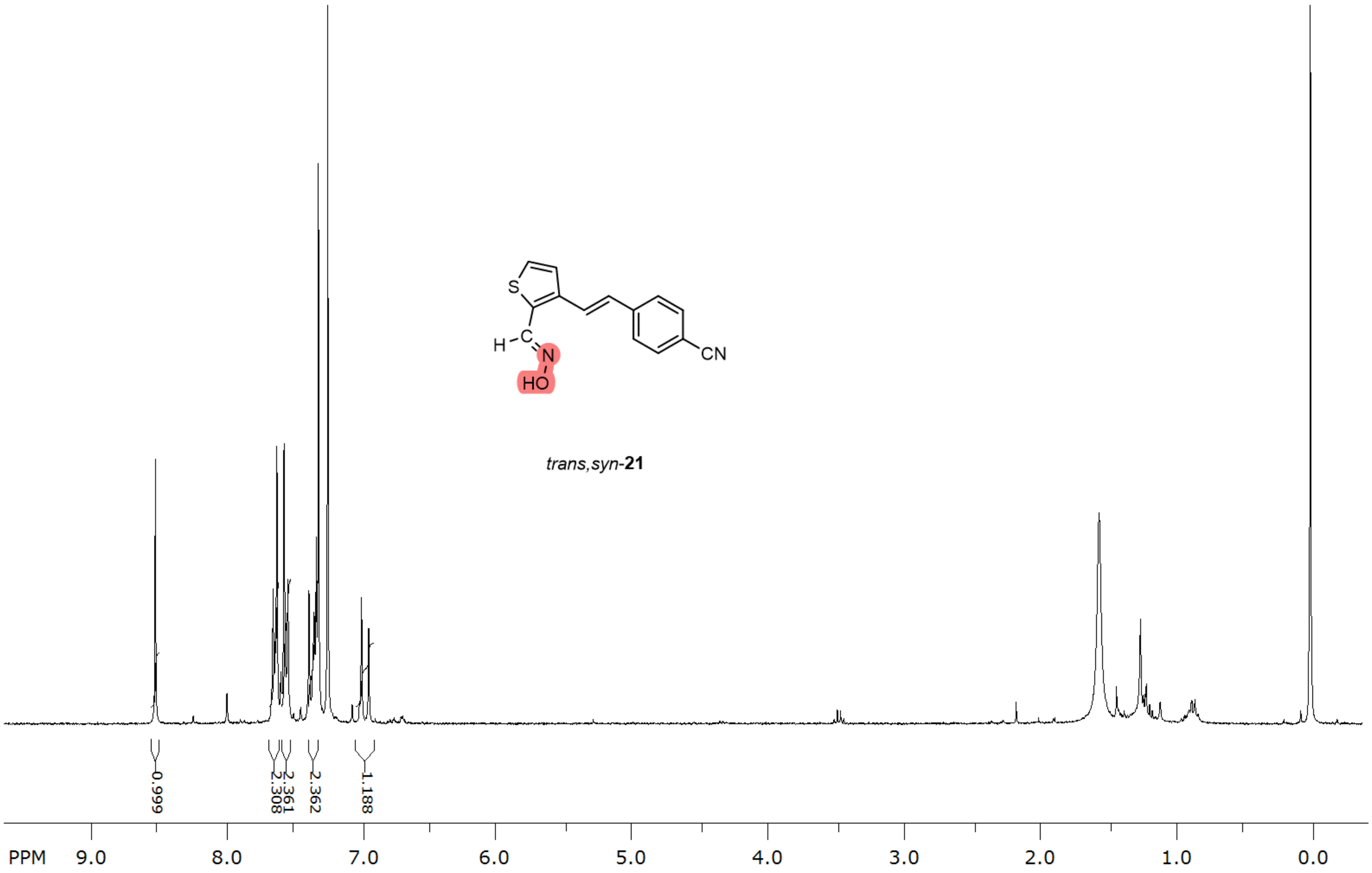

Figure S278. ^{1}H NMR ($CDCl_3$) spectrum of *trans,syn*-**21**.

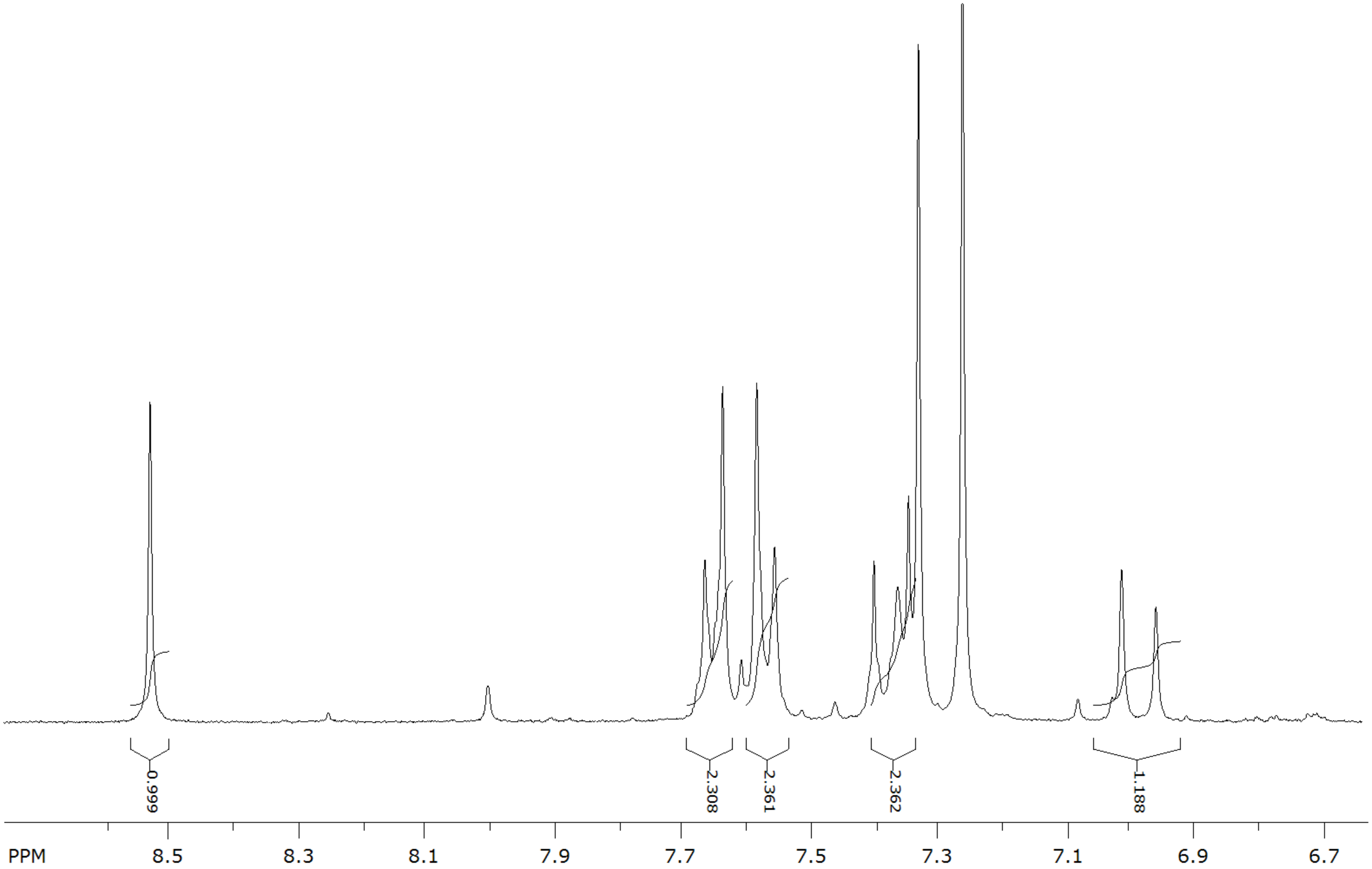

Figure S279. ¹H NMR ($CDCl_3$) spectrum of aromatic part of *trans,syn*-**21**.

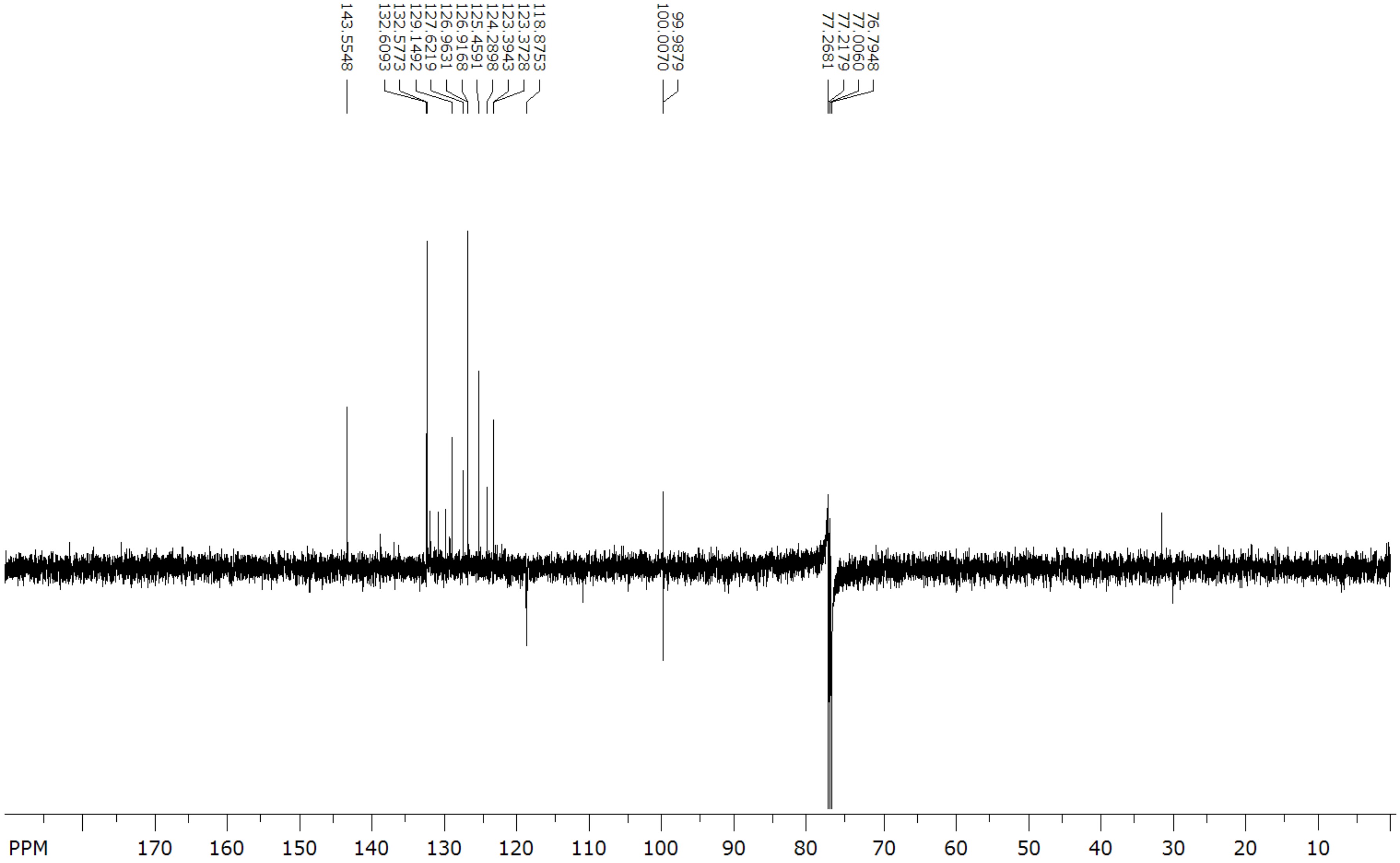

Figure S280. ^{13}C NMR ($CDCl_3$) spectrum of *trans,syn*-**21**.

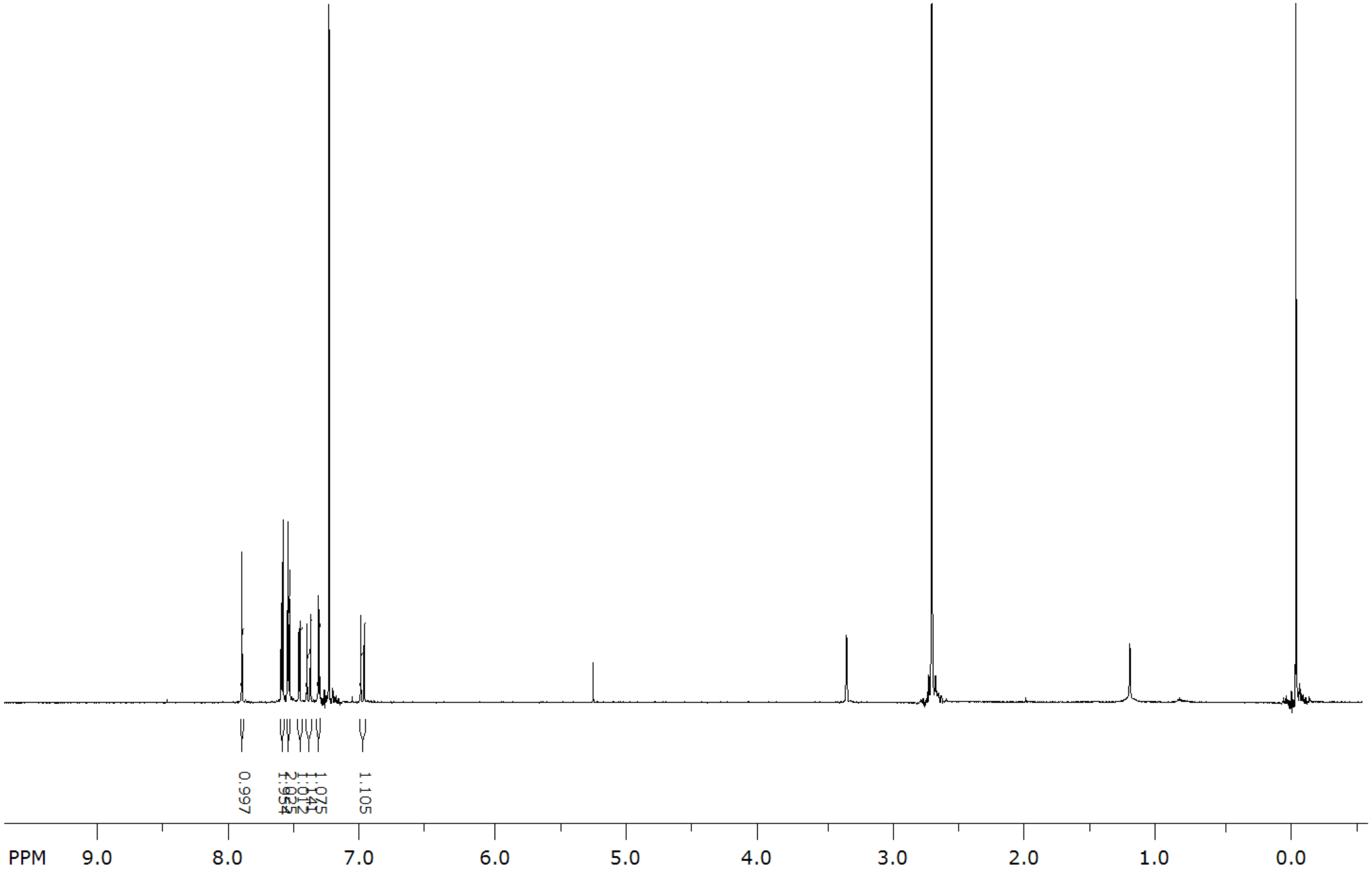

Figure S281. ^{1}H NMR ($CDCl_3$) spectrum of *trans,anti*-**21**.

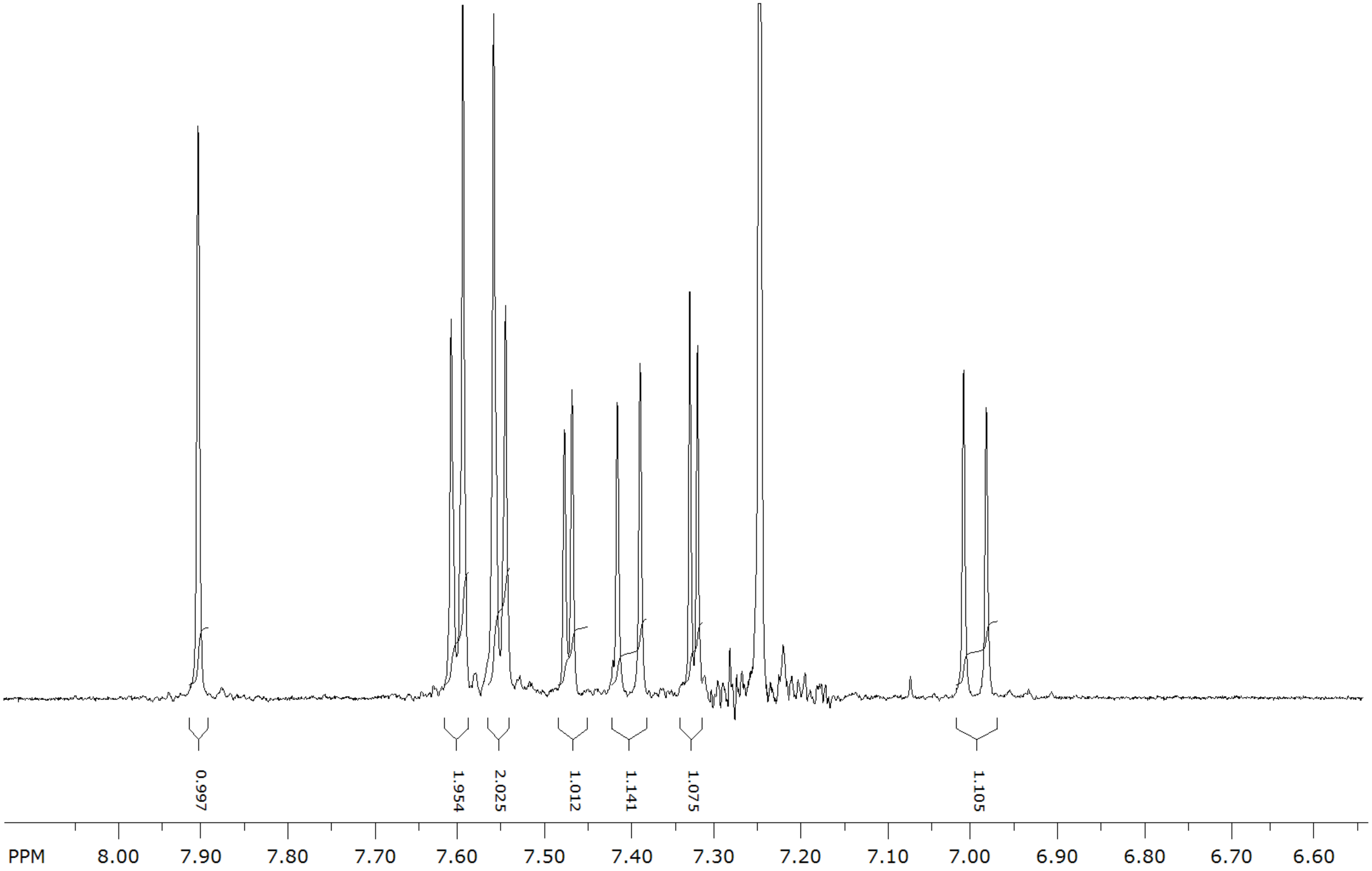

Figure S282. ^{1}H NMR ($CDCl_3$) spectrum of aromatic part of *trans,anti*-**21**.

2. Mass spectra and HRMS analyses of oximes 1-21

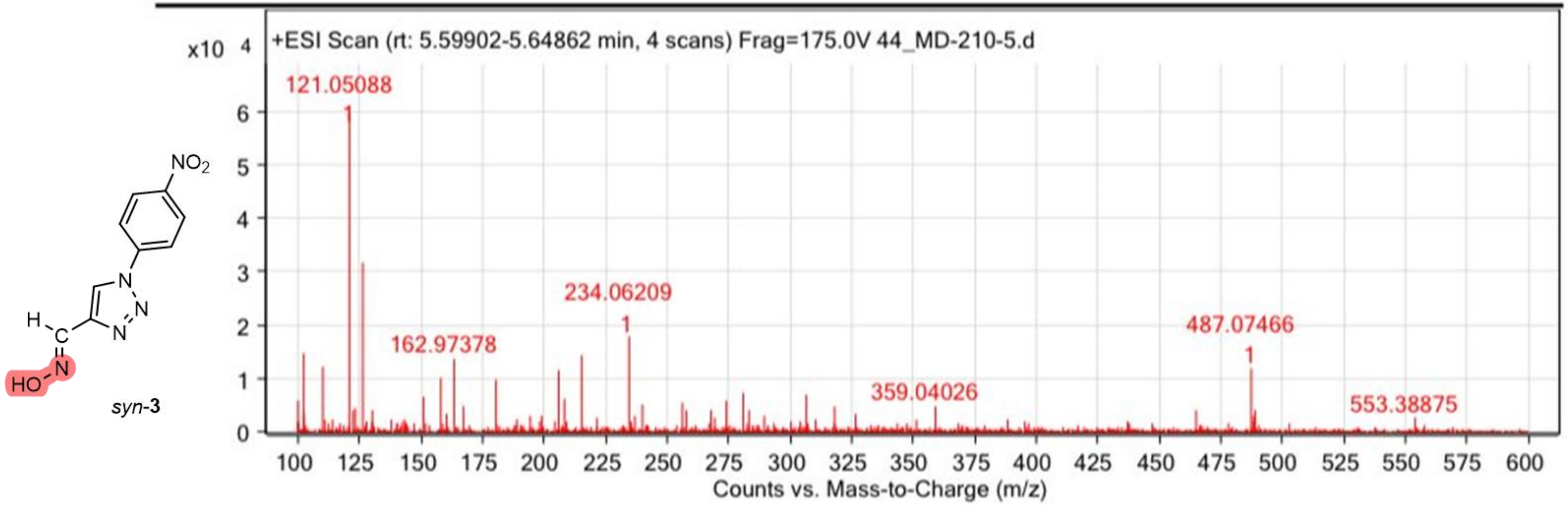

Formula Calculator Results

Formula	Best	Mass	Tgt Mass	Diff (ppm)	Ion Species	Score
C9 H7 N5 O3	True	233.05491	233.05489	-0.09	C9 H8 N5 O3	98.11

Figure S283. HRMS of **3**.

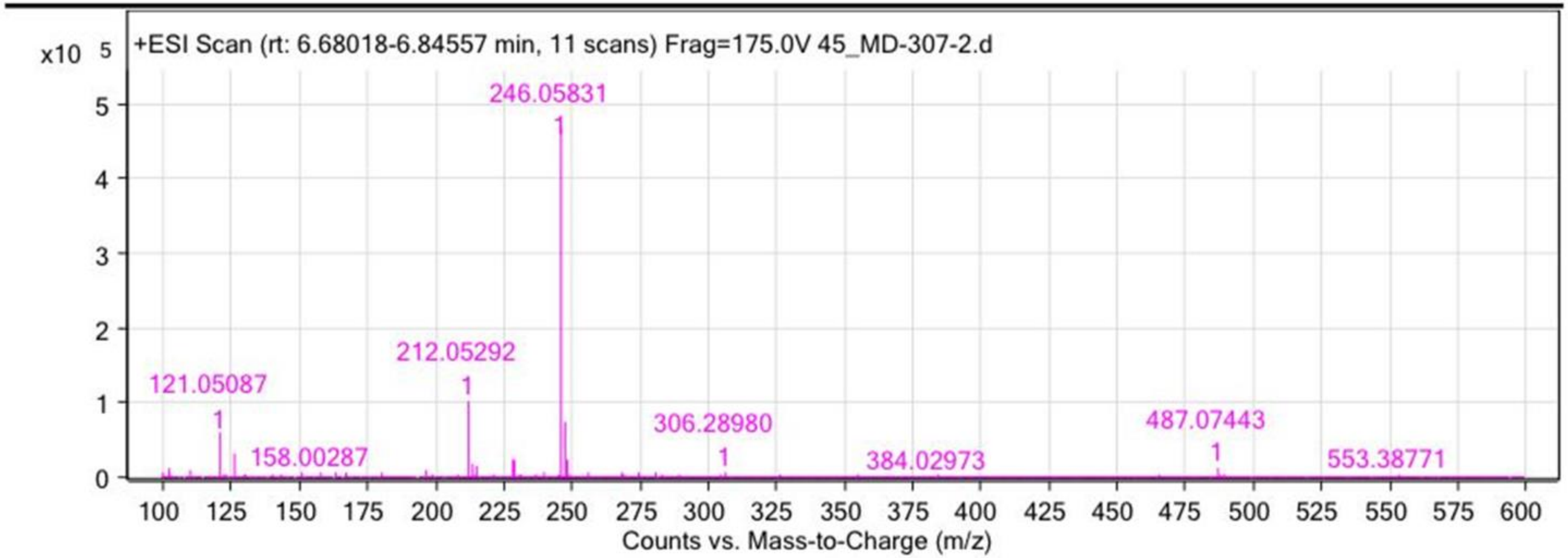

Formula Calculator Results

Formula	Best	Mass	Tgt Mass	Diff (ppm)	Ion Species	Score
C13 H9 N O2 S	True	243.03544	243.0354	-0.17	C13 H10 N O2 S	47.61

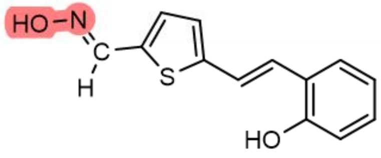

trans,syn-**4**

Figure S284. HRMS of *trans,syn*-**4**.

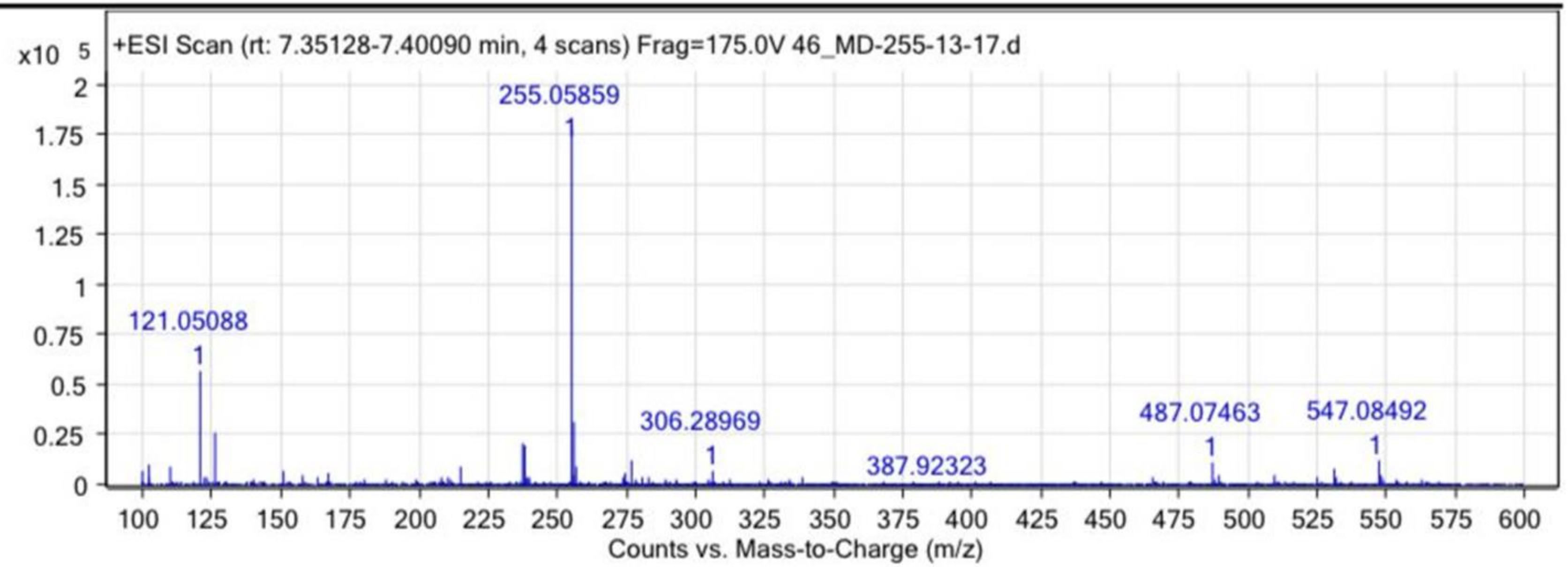

Formula Calculator Results

Formula	Best	Mass	Tgt Mass	Diff (ppm)	Ion Species	Score
C14 H10 N2 O S	True	254.05132	254.05138	0.24	C14 H11 N2 O S	99.3

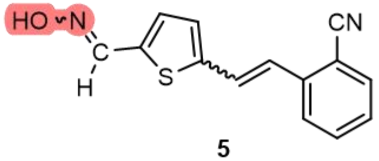

5

Figure S285. HRMS of **5**.

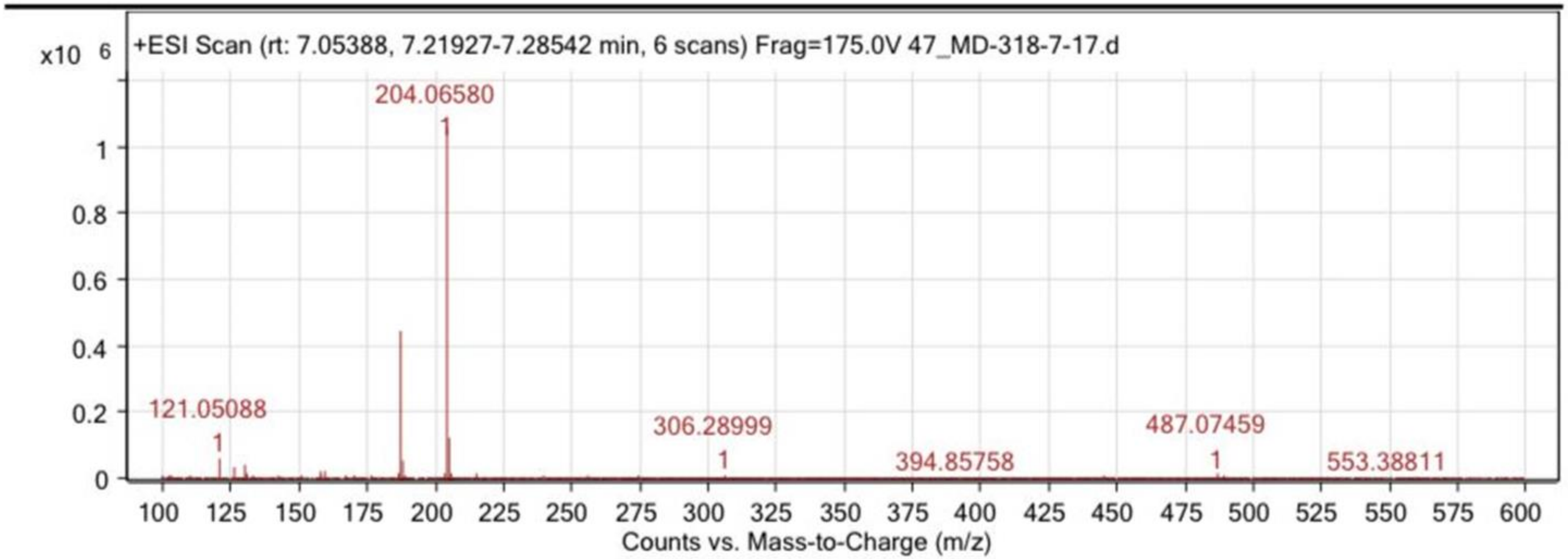

Formula Calculator Results

Formula	Best	Mass	Tgt Mass	Diff (ppm)	Ion Species	Score
C11 H9 N O3	True	203.05853	203.05824	-1.42	C11 H10 N O3	99.05

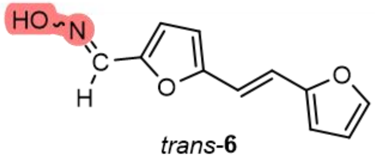

trans-**6**

Figure S286. HRMS of *trans*-**6**.

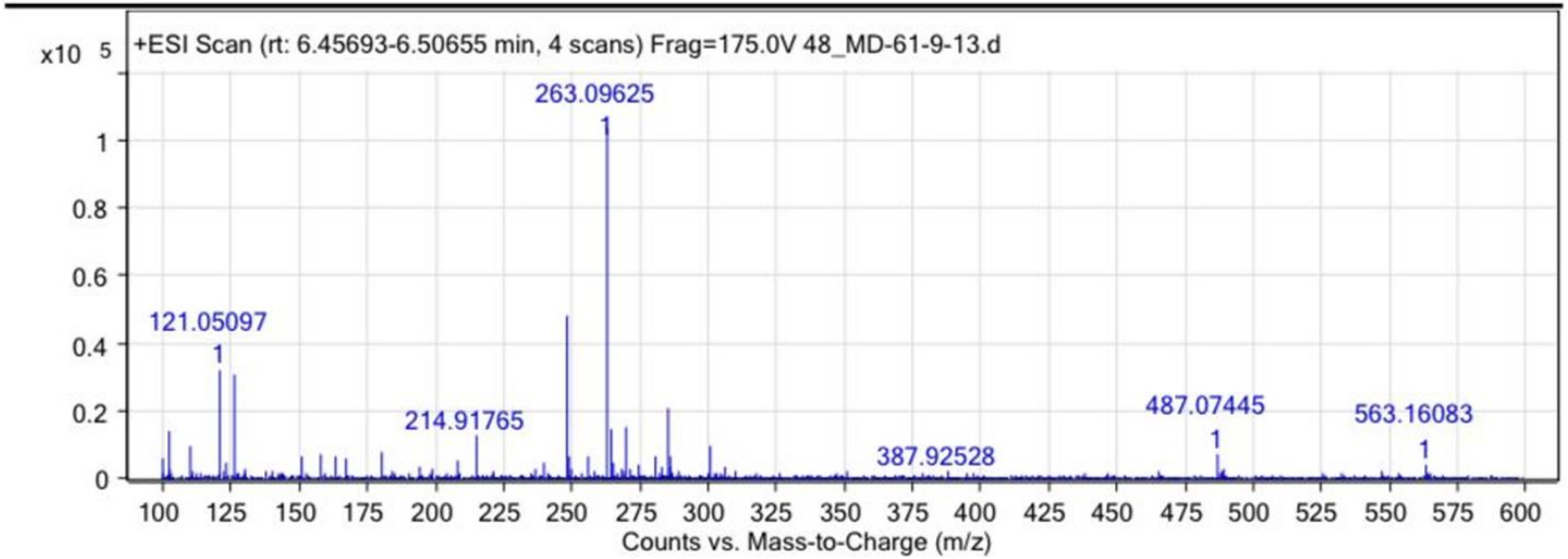

Formula Calculator Results

Formula	Best	Mass	Tgt Mass	Diff (ppm)	Ion Species	Score
C12 H14 N4 O S	True	262.08898	262.08883	-0.58	C12 H15 N4 O S	98.78

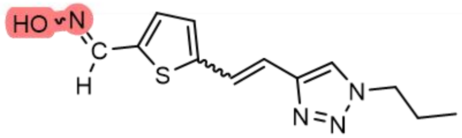

7

Figure S287. HRMS of **7**.

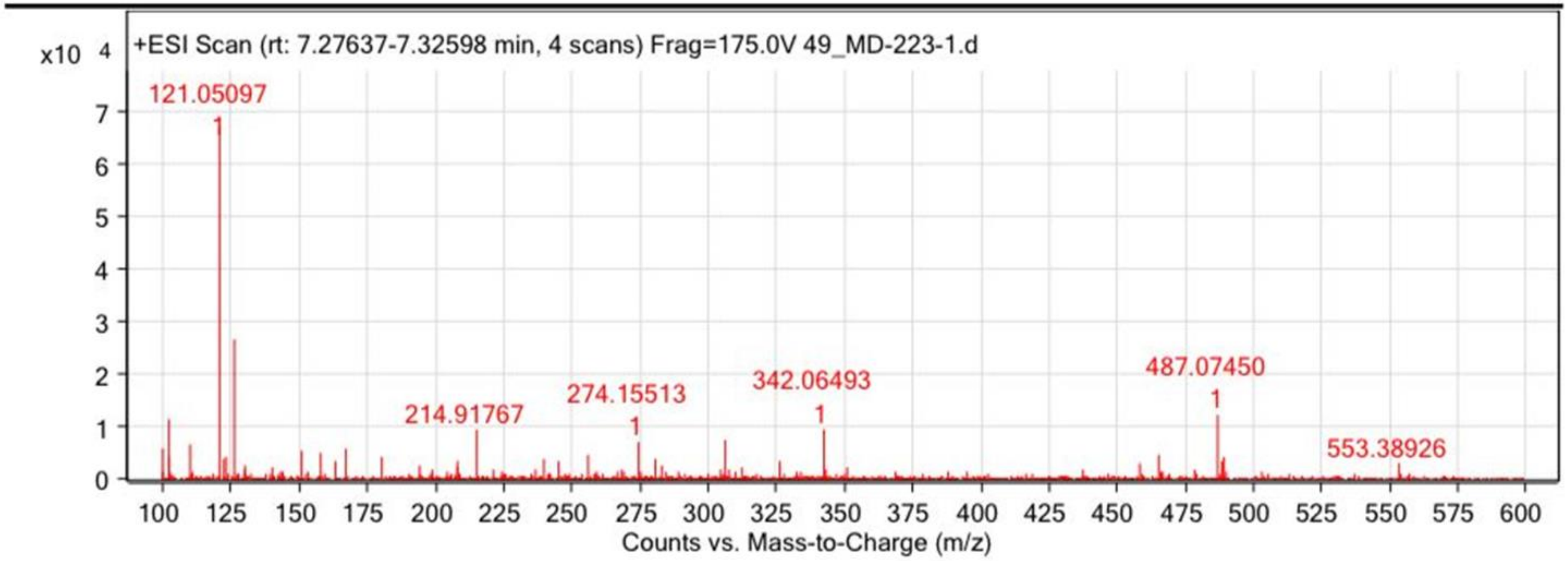

Formula Calculator Results

Formula	Best	Mass	Tgt Mass	Diff (ppm)	Ion Species	Score
C15 H11 N5 O3 S	True	341.05771	341.05826	1.62	C15 H12 N5 O3 S	98.14

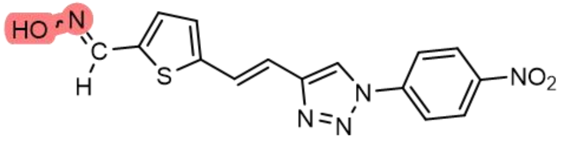

trans-**8**

Figure S288. HRMS of *trans*-**8**.

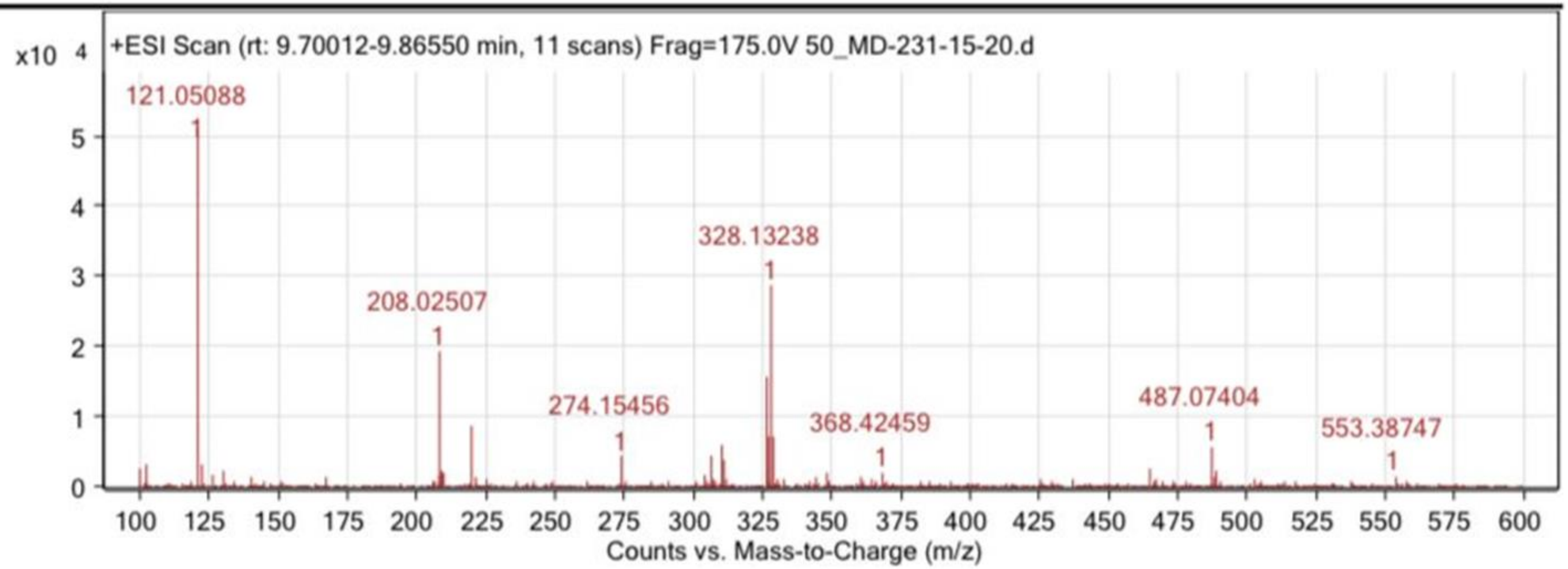

Formula Calculator Results

Formula	Best	Mass	Tgt Mass	Diff (ppm)	Ion Species	Score
C22 H17 N O2	True	327.12512	327.12593	2.46	C22 H18 N O2	97.8

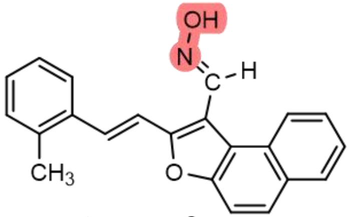

trans,syn-**9**

Figure S289. HRMS of *trans,syn*-**9**.

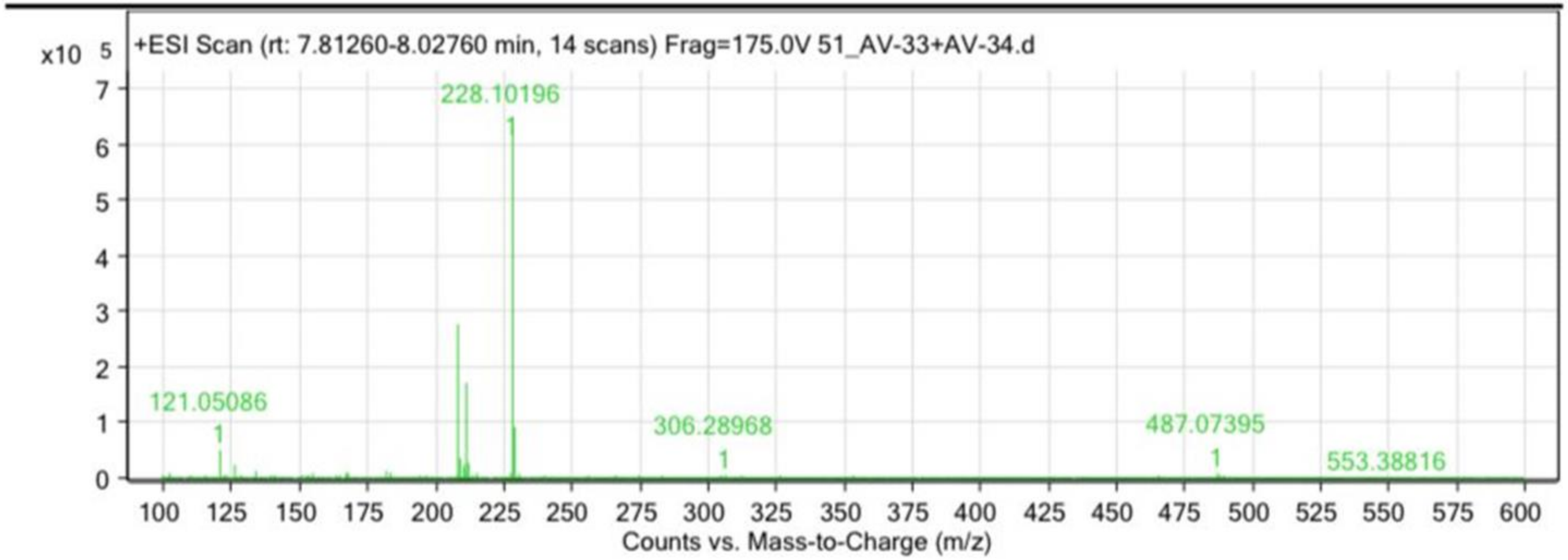

Formula Calculator Results

Formula	Best	Mass	Tgt Mass	Diff (ppm)	Ion Species	Score
C14 H13 N O2	True	227.09469	227.09463	-0.28	C14 H14 N O2	99.65

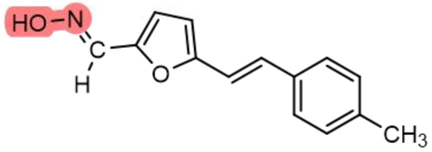

trans,syn-**10**

Figure S290. HRMS of *trans,syn*-**10**.

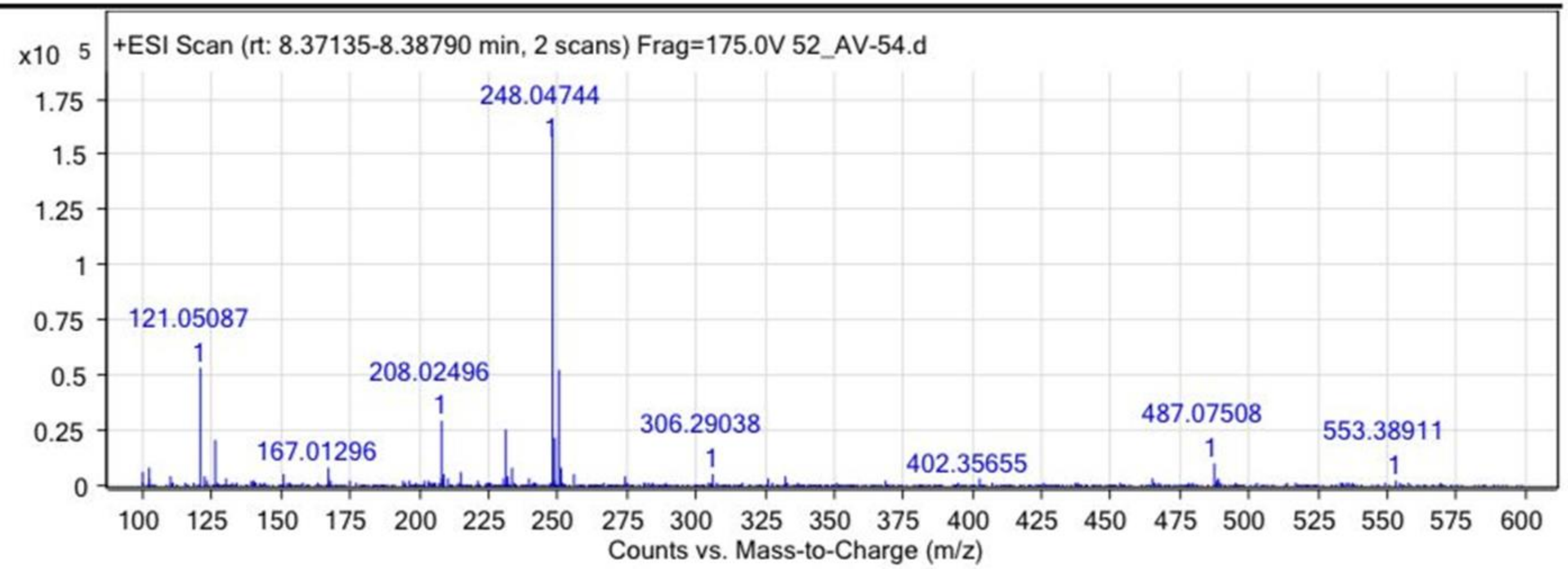

Formula Calculator Results

Formula	Best	Mass	Tgt Mass	Diff (ppm)	Ion Species	Score
C13 H10 Cl N O2	True	247.04013	247.04001	-0.48	C13 H11 Cl N O2	99.3

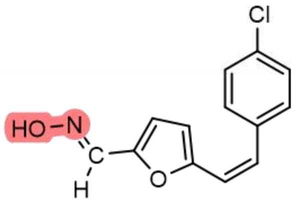

cis,syn-**12**

Figure S291. HRMS of *cis,syn*-**12**.

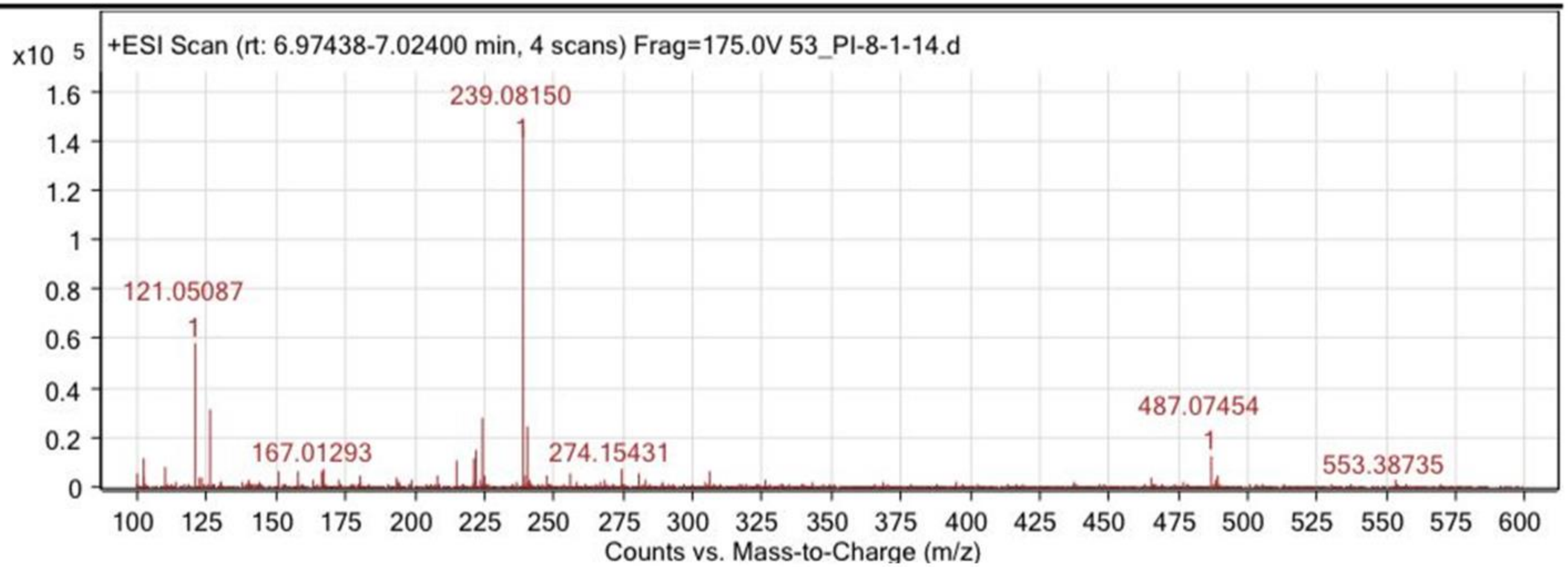

Formula Calculator Results

Formula	Best	Mass	Tgt Mass	Diff (ppm)	Ion Species	Score
C14 H10 N2 O2	True	238.07424	238.07423	-0.04	C14 H11 N2 O2	99.72

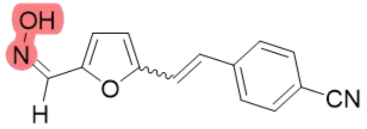

trans,anti-**13'**

Figure S292. HRMS of *trans,anti*-**13'**.

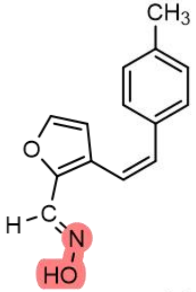

cis,syn-**14**

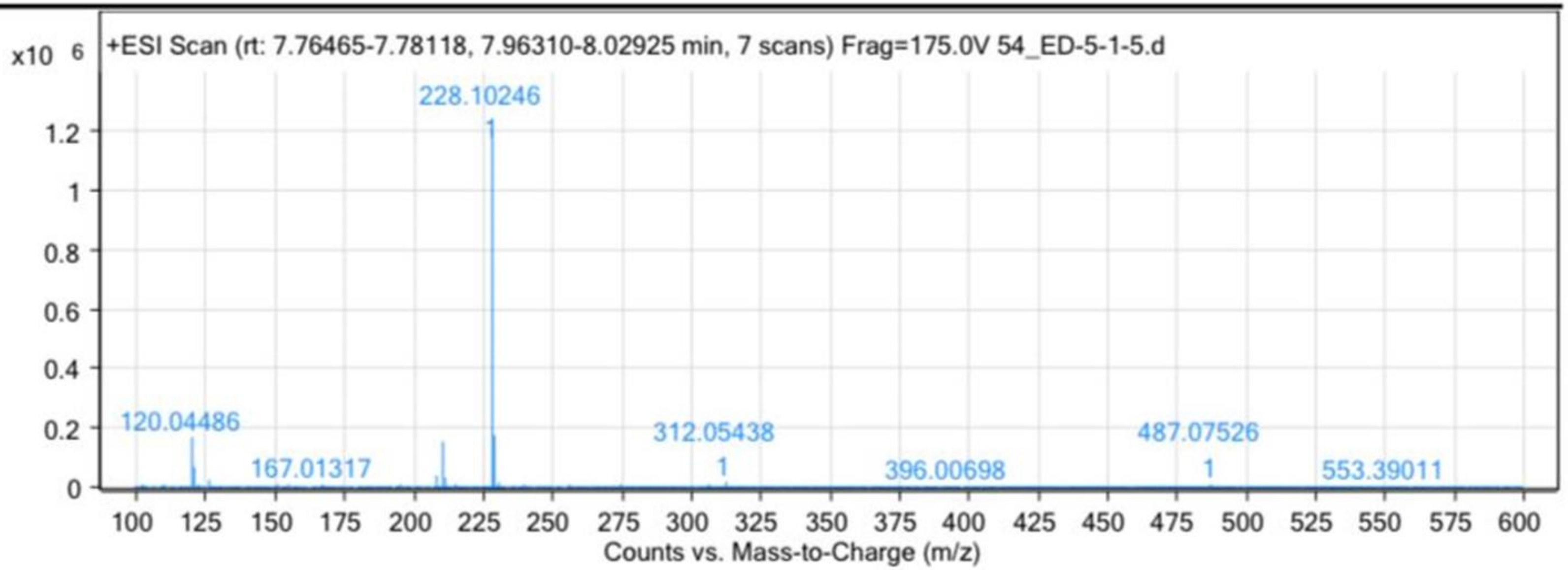

Formula Calculator Results

Formula	Best	Mass	Tgt Mass	Diff (ppm)	Ion Species	Score
C14 H13 N O2	True	227.09521	227.09463	-2.55	C14 H14 N O2	97.85

Figure S293. HRMS of *cis,syn*-**14**.

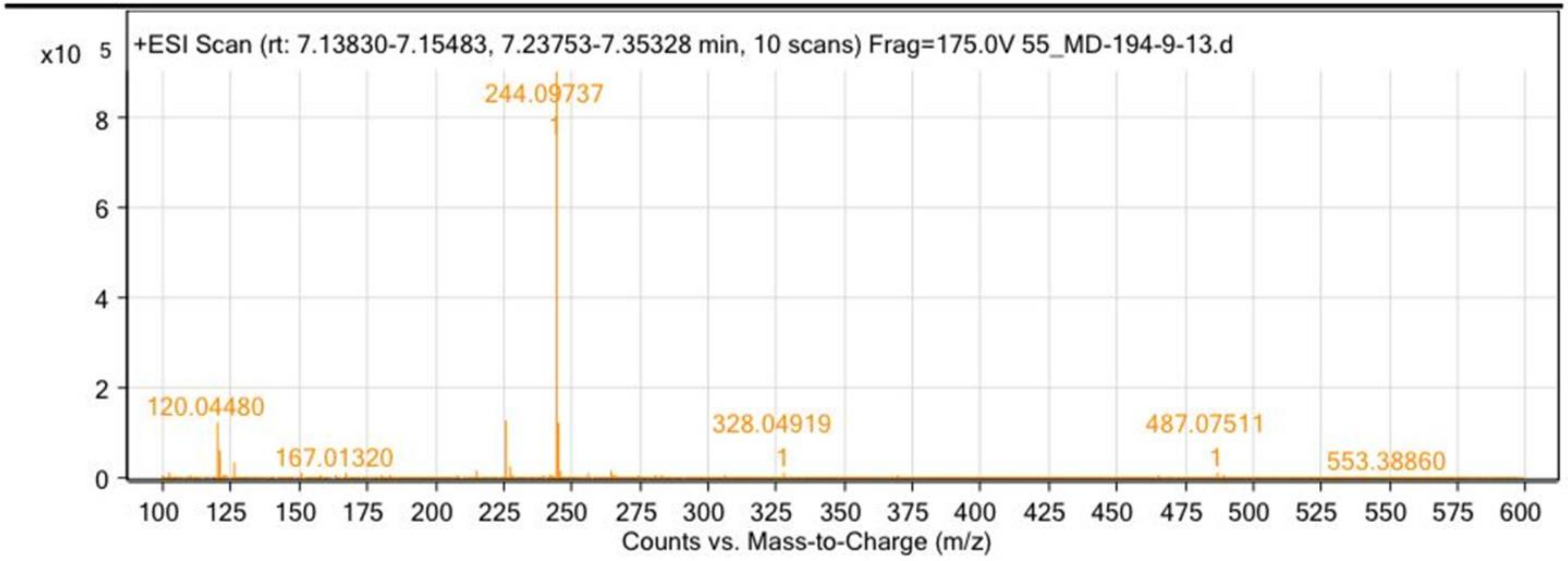

Formula Calculator Results

Formula	Best	Mass	Tgt Mass	Diff (ppm)	Ion Species	Score
C14 H13 N O3	True	243.0901	243.08954	-2.3	C14 H14 N O3	97.34

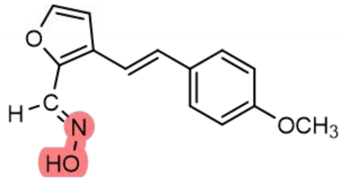

trans,syn-**15**

Figure S294. HRMS of *trans,syn*-**15**.

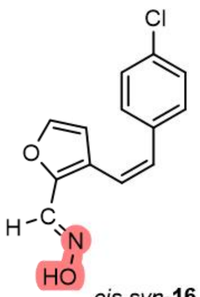

cis,syn-**16**

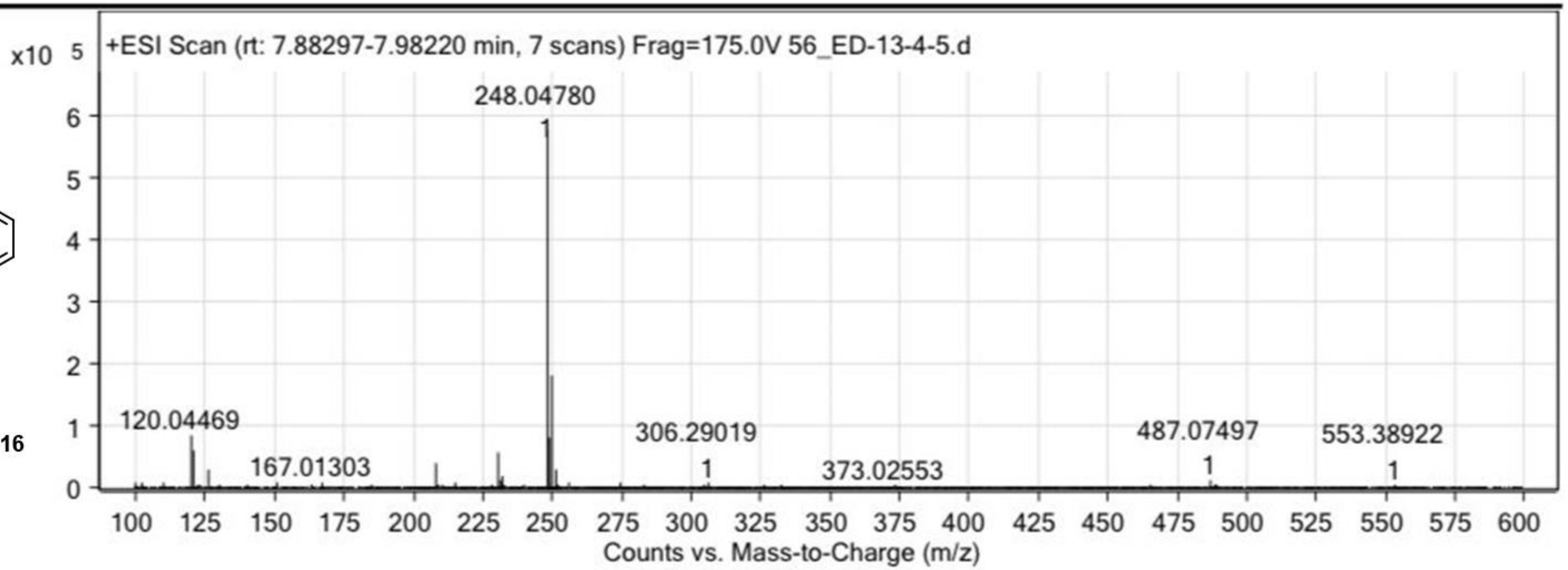

Formula Calculator Results

Formula	Best	Mass	Tgt Mass	Diff (ppm)	Ion Species	Score
C13 H10 Cl N O2	True	247.04048	247.04001	-1.9	C13 H11 Cl N O2	98.46

Figure S295. HRMS of *cis,syn*-**16**.

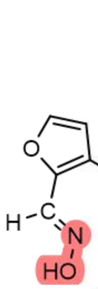

cis,syn-**17**

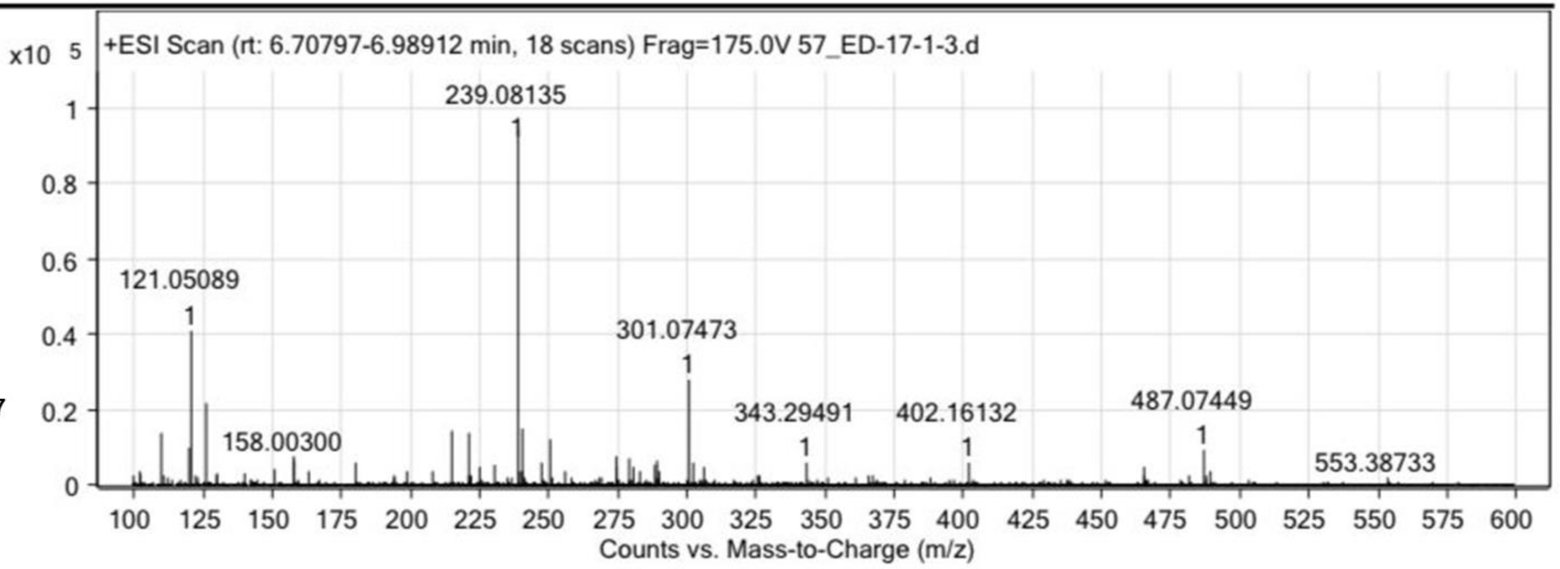

Formula Calculator Results

Formula	Best	Mass	Tgt Mass	Diff (ppm)	Ion Species	Score
C14 H10 N2 O2	True	238.07411	238.07423	0.49	C14 H11 N2 O2	99.75

Figure S296. HRMS of *cis,syn*-**17**.

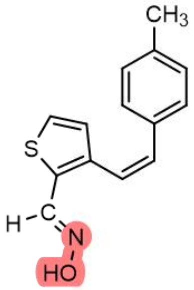

cis,syn-**18**

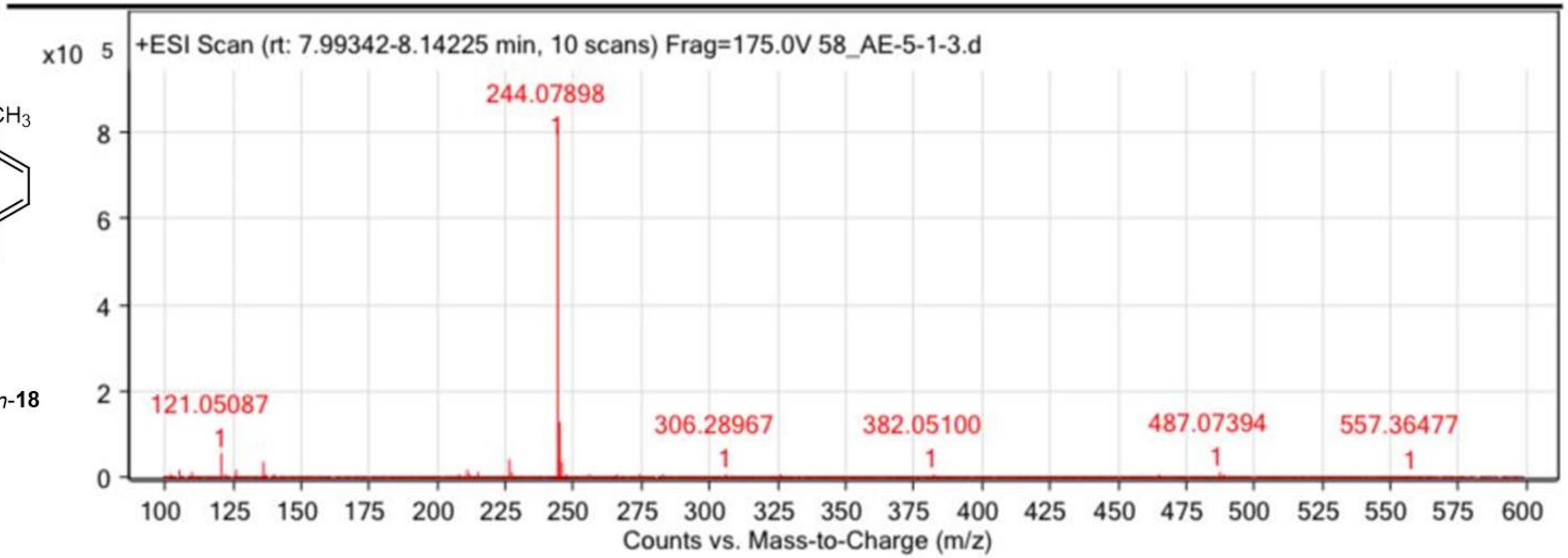

Formula Calculator Results

Formula	Best	Mass	Tgt Mass	Diff (ppm)	Ion Species	Score
C14 H13 N O S	True	243.07169	243.07178	0.39	C14 H14 N O S	98.89

Figure S297. HRMS of *cis,syn*-**18**.

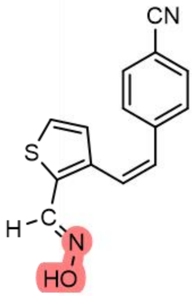

cis,syn-**21**

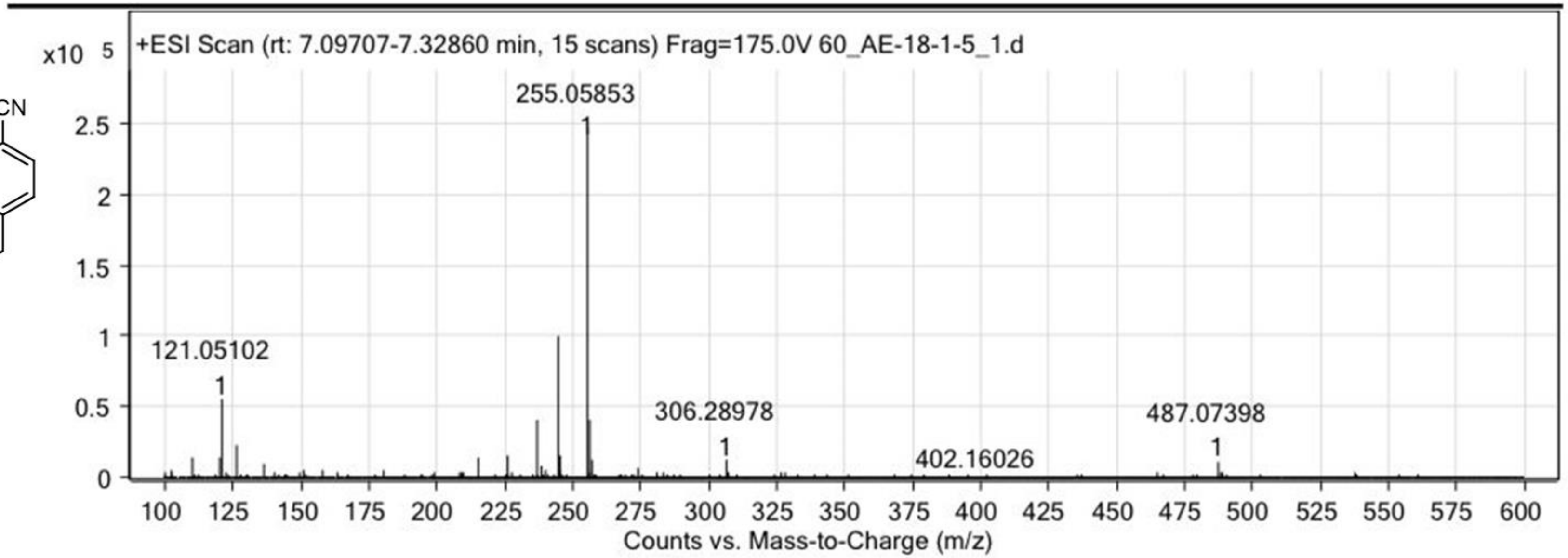

Formula Calculator Results

Formula	Best	Mass	Tgt Mass	Diff (ppm)	Ion Species	Score
C14 H10 N2 O S	True	254.05124	254.05138	0.57	C14 H11 N2 O S	99.26

Figure S298. HRMS of *cis,syn*-**21**.

3. Evaluation of cytotoxicity

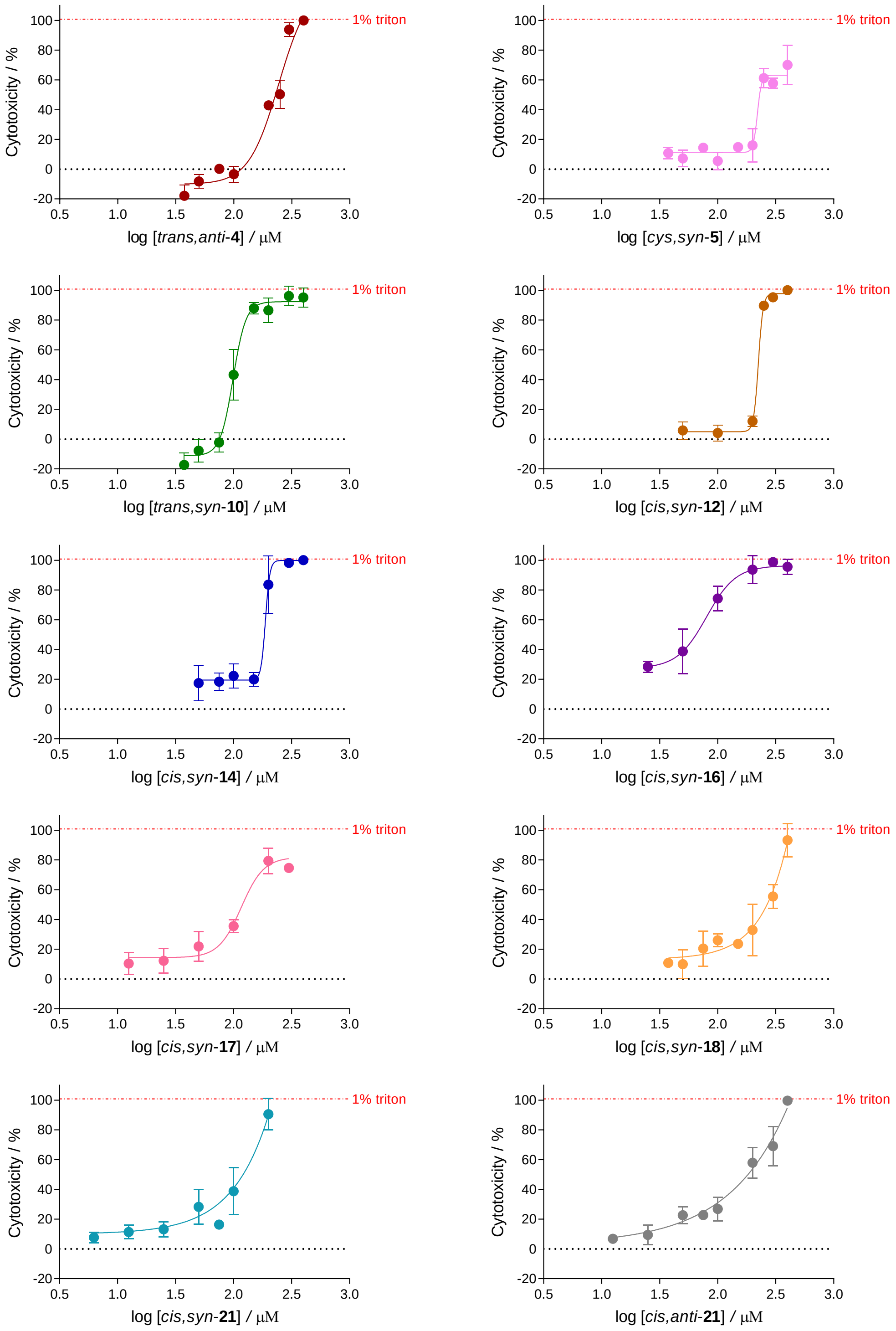

Figure S299. Cytotoxicity of oximes on liver cell line in 24 h exposure time plotted as IC_{50} curves.

4. Inhibition of AChE and BChE by selected oximes

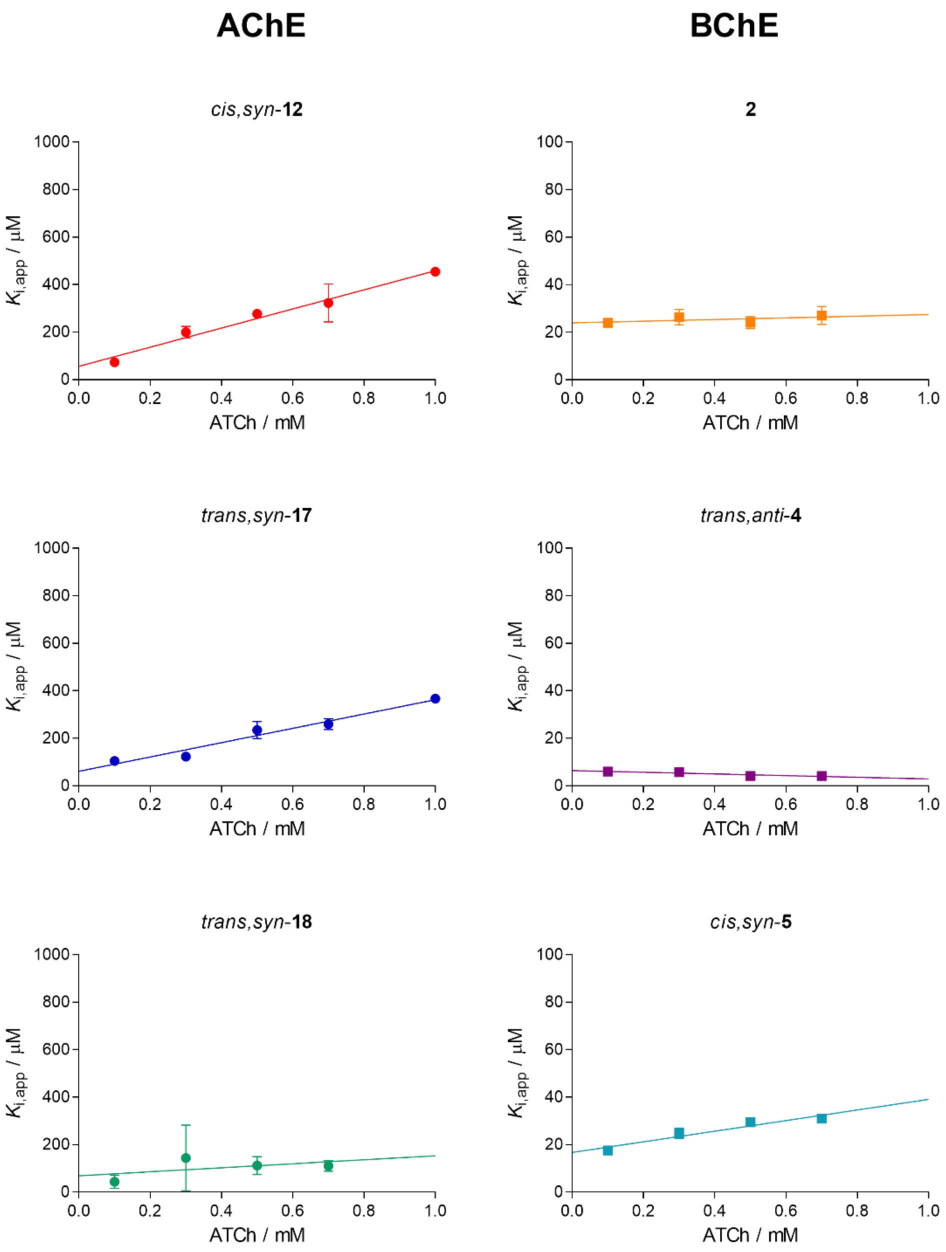

Figure S300. Hunter-Downs plots for determination of inhibition dissociation constant.

5. Molecullar docking of human AChE and BChE

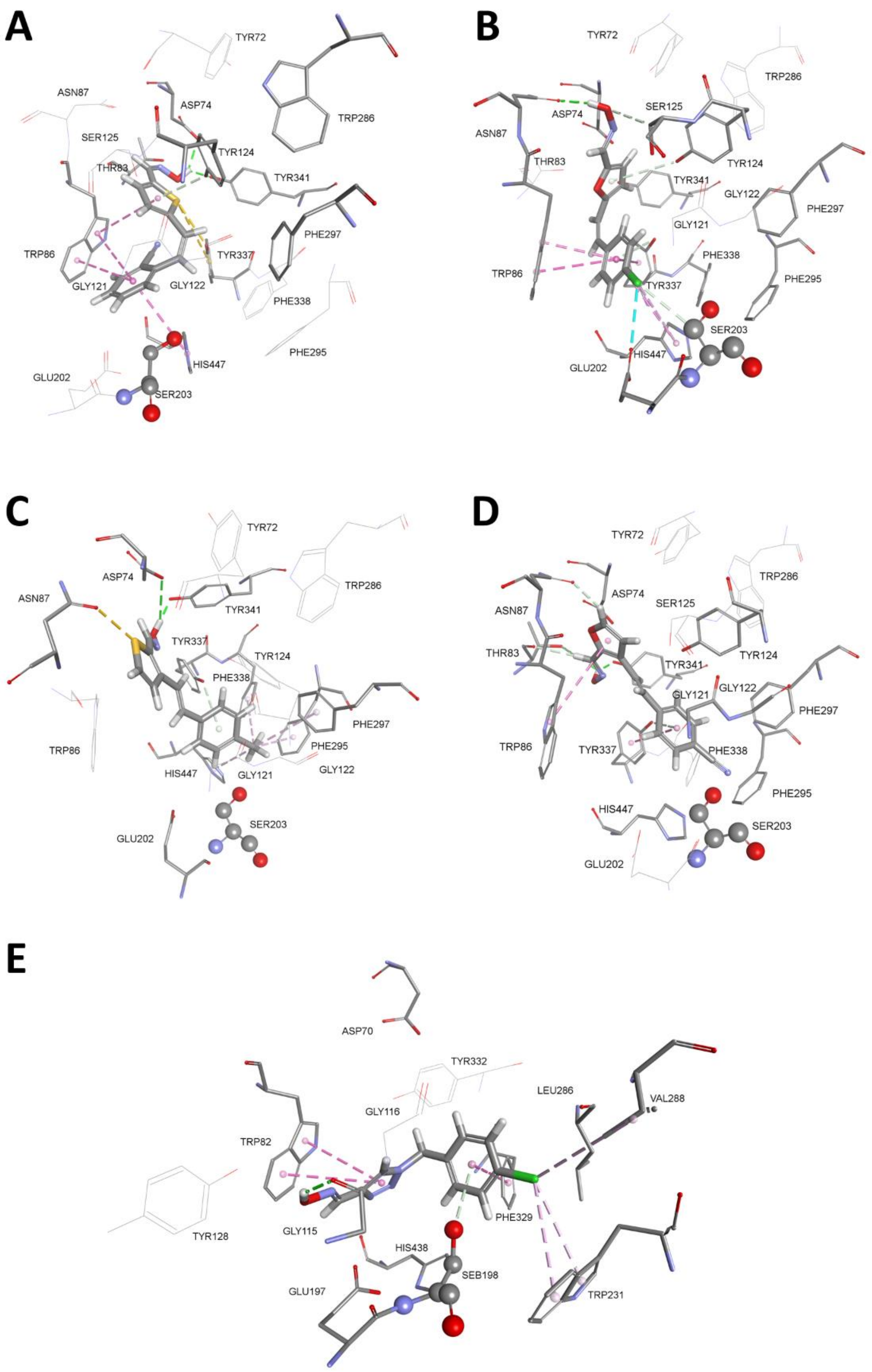

Figure S301. Conformation of complex between heterostilbene oxime molecule and native human AChE and BChE. **A)** Compound *cis,syn*-**5** in AChE. **B**) Compound *cis,syn*-**12** in AChE. **C)** Compound *trans,syn*-**18** in AChE. **D)** Compound *trans,syn*-**17** in AChE. **E)** Compound **2** in BChE. Interactions with amino acid residues are represented as dashed lines: hydrophobic (purple), hydrogen bonds (green) and electrostatic (orange). Crystal structure of human AChE was used (PDB code 4PQE) and human BChE was used (PDB code 2PM8).

6. Molecular dynamics simulation of oxime near-attack conformation

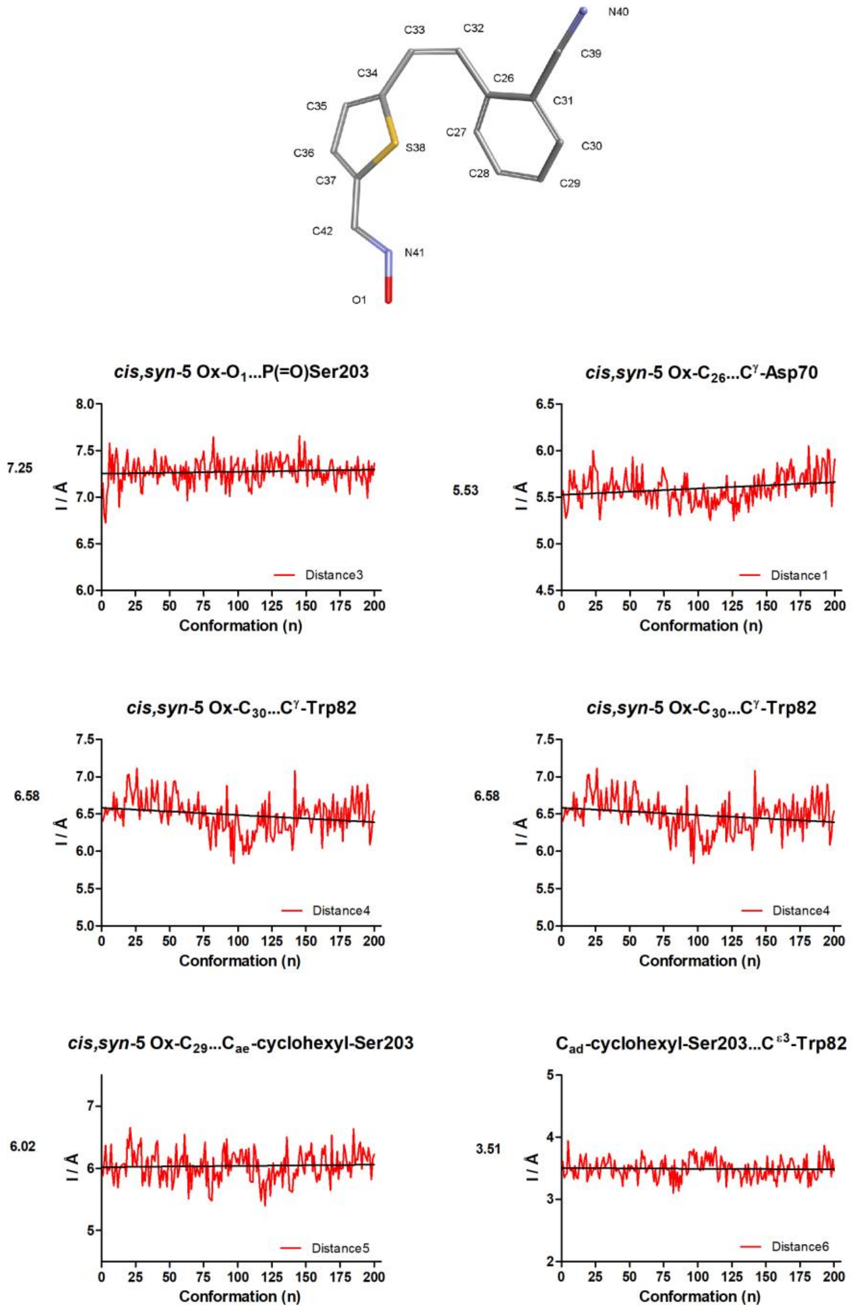

Figure S302. Molecular dynamics simulation (t=20 ns) of complex between an oxime *cis,syn*-5 and cyclosarin-inhibited BChE. Distances between atoms of *cis,syn*-5 and selected atoms of BChE active site residues are shown. Distance average value (Å) is listed left of Y-axes.

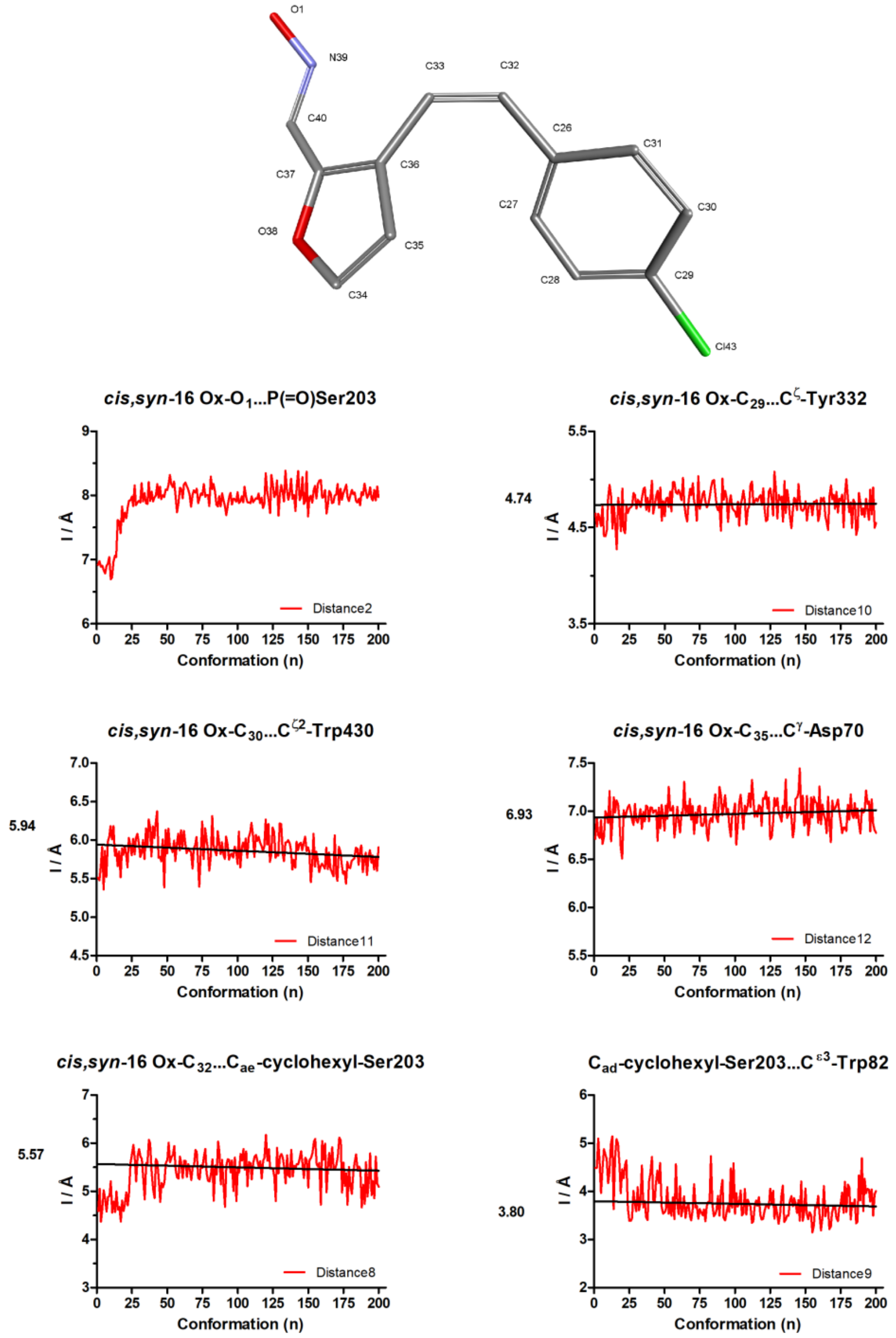

Figure S303. Molecular dynamics simulation (t=20 ns) of complex between an oxime *cis,syn*-16 and cyclosarin-inhibited BChE. Distances between atoms of *cis,syn*-16 and selected atoms of BChE active site residues are shown. Distance average value (Å) is listed left of Y-axes.
